# Analysis of MicroRNA Expression in the Prepubertal Testis

**DOI:** 10.1371/journal.pone.0015317

**Published:** 2010-12-29

**Authors:** Gregory M. Buchold, Cristian Coarfa, Jong Kim, Aleksandar Milosavljevic, Preethi H. Gunaratne, Martin M. Matzuk

**Affiliations:** 1 Department of Pathology, Baylor College of Medicine, Houston, Texas, United States of America; 2 Department of Molecular and Cellular Biology, Baylor College of Medicine, Houston, Texas, United States of America; 3 Department of Molecular and Human Genetics, Baylor College of Medicine, Houston, Texas, United States of America; 4 Human Genome Sequencing Center, Baylor College of Medicine, Houston, Texas, United States of America; 5 Department of Biology and Biochemistry, University of Houston, Houston, Texas, United States of America; University of Otago, New Zealand

## Abstract

Only thirteen microRNAs are conserved between *D. melanogaster* and the mouse; however, conditional loss of miRNA function through mutation of Dicer causes defects in proliferation of premeiotic germ cells in both species. This highlights the potentially important, but uncharacterized, role of miRNAs during early spermatogenesis. The goal of this study was to characterize on postnatal day 7, 10, and 14 the content and editing of murine testicular miRNAs, which predominantly arise from spermatogonia and spermatocytes, in contrast to prior descriptions of miRNAs in the adult mouse testis which largely reflects the content of spermatids. Previous studies have shown miRNAs to be abundant in the mouse testis by postnatal day 14; however, through Next Generation Sequencing of testes from a B6;129 background we found abundant earlier expression of miRNAs and describe shifts in the miRNA signature during this period. We detected robust expression of miRNAs encoded on the X chromosome in postnatal day 14 testes, consistent with prior studies showing their resistance to meiotic sex chromosome inactivation. Unexpectedly, we also found a similar positional enrichment for most miRNAs on chromosome 2 at postnatal day 14 and for those on chromosome 12 at postnatal day 7. We quantified *in vivo* developmental changes in three types of miRNA variation including 5′ heterogeneity, editing, and 3′ nucleotide addition. We identified eleven putative novel pubertal testis miRNAs whose developmental expression suggests a possible role in early male germ cell development. These studies provide a foundation for interpretation of miRNA changes associated with testicular pathology and identification of novel components of the miRNA editing machinery in the testis.

## Introduction

The expression and modification of miRNAs have been an area of intense interest. In brief, miRNA biosynthesis involves primary miRNA (pri-miRNA) transcription by RNA polymerase II and folding of the pri-miRNA into a secondary structure that is recognized and cleaved by the microprocessor complex, Drosha and DGCR8, to yield a stem-loop or pre-miRNA. This pre-miRNA is exported from the nucleus by exportin 5 and cleaved by Dicer in the cytoplasm to yield a double-stranded RNA of 21–22 nts containing both strands of the hairpin, designated 5p and 3p [1], [2], [3], [4]. Subsequently, the two strands are separated and generally one of the two (the guide strand) is incorporated into the RISC effector complex, containing Argonaute proteins, while the passenger or star strand is degraded. However, some star strands may be stable and functional. Using the specificity contained within nucleotides 2–7 (5′seed) and 13–16 (anchor) of the guide strand, the RISC complex targets mRNAs through complementary sequences in their 3′ UTR for cleavage or translational repression [5], [6]. During miRNA biosynthesis, RNA-binding proteins, such as LIN28, can associate with the small RNA, preventing or altering its processing [7].

Genetic studies disrupting miRNA functions in mammals by targeting Dicer, Drosha, DGCR8, or individual miRNAs have revealed specific and global roles of miRNAs a variety of developmental processes and pathologic states. Germ cell-specific deletion of Dicer^−/−^ shows that miRNAs are required for regulation of male gonocyte proliferation [8]. MicroRNAs have also been implicated in the pathogenesis of human germ cell tumors (e.g., mir-372 and mir-373) [9] or several cancers including testicular cancer (e.g. let-7c) [10].

Localization studies of miRNAs and their associated enzymes suggest that they may contribute to post-meiotic male germ cell function. Complexes of miRNAs and their targets as well as Dicer accumulate in the chromatoid body of spermatids [11]; however, their function and localization have not been described in earlier spermatogenic cells. A number of mRNAs are associated with the chromatoid body that are first transcribed in spermatocytes but have no detectable protein expression until some days later [12]. Therefore, this translational delay may result from the action of miRNAs localized in other germ cell RNA granules such as intermitochondrial cement, MIWI2 pi-P bodies, chromatoid bodies, etc. [13]. This correlates with the dramatic increase in overall miRNAs at postnatal day 14 (P14) when pachytene cells are first abundant in the testis. Several groups have described the complement of miRNAs present in the adult mouse testis [14], [15], [16] or human testicular tumors [10], [17], [18] using low-throughput assays and more recently Next Generation Sequencing methods [19]. Next Generation Sequencing of female tissues has uncovered novel small RNAs missed by prior analyses and allows the identification of sequence differences reflective of potential post-transcriptional modification relevant to target regulation [20].

Editing of a variety of RNAs occurs frequently in mammals with the majority of modifications caused by A-to-I editing and 3′ terminal A and U additions with C-to-U editing occurring less frequently [21], [22]. Sixteen percent of miRNAs are also modified [23], [24], [25], predominantly by adenosine deamination of precursor miRNAs by ADARs [26], [27], [28], [29], [30]. ADAR-dependent editing can control targeting specificity and the stability and processing of miRNA precursor transcripts [30], [31], [32]. The editing of nucleotides in the vicinity of Dicer or Drosha processing sites can prevent the further maturation and expression of the miRNA [31], [33], [34], [35]. In the rat, the highest degree of ADAR-dependent editing occurs in brain followed by testis [36]. The second major class of miRNA editing events (C-to-U) depends on the APOBEC (apolipoprotein B mRNA editing enzyme, catalytic polypeptide) family of cytidine deaminases which can prevent translational inhibition of miRNA targets [37]. Additional types of editing yielding variant miRNAs, sometimes called isomiRs [38], have been described in the adult ovaries and testes including variations in the 5′ end cleavage by Drosha or Dicer and nontemplated nucleotide addition to the mature miRNA [19], [20], [39], [40]. Variations in the miRNA 5′ end alter their 5′seed sequence, lowering the affinity of miRNA to target mRNAs. Uridylation of the miRNA 3′ end results in their destabilization. LIN28-dependent uridylation of let-7 miRNAs by polyuridine polymerase ZCCHC11 is the most notable example of this modification [41], [42], [43]. Thus, editing events act to oppose or modulate the action of miRNAs.

Previously, we reported the presence of miRNAs in the testis prior to P14 [44] but did not describe the profile in detail. Our current studies demonstrate that the miRNAs most enriched at P7 and P14 derive predominantly from chromosome 12, and then chromosomes 2 and X, in contrast to those from the adult that are expressed from more diverse chromosomal locations. To date, the description of miRNA editing from deep sequencing in a developmental context has only been described *in vitro* [45]; our current study is the first to analyze editing in the *in vivo* context of pubertal spermatogenesis, preceding the high degree of editing described in the adult testis. All types of editing events are modestly higher at P7, overlapping with only a fraction of the editing events in the adult testis. We believe that profiling miRNA changes during normal testicular development will aid in the interpretation of significant changes in miRNAs occurring in pathologic states (i.e., infertility and cancer) and may suggest novel regulation of miRNAs in male germ cells.

## Methods

### Animal husbandry and tissue isolation

Mice were generated from our mating colony bearing the Gasz mutant allele, *Gasz^tm1Zuk^*, by intercrossing heterozygous sires and homozygous null dams of B6;129 mixed background (129S7/AB2.2×C57BL6/J) [44]. Breeders were housed as trios (one male and two females) with pups in microisolator cages on a 12 hr light/dark cycle (7am-7pm) at 70°F±2°F. Mice were provided Harland Teklad 2919 (breeder chow), acidified water; and nestlets for environmental enrichment. Testes were collected under inhaled anesthesia from two GASZ^+/−^ mice with litter-matched GASZ null controls from two different litters on postnatal day 7, 10, and 14. Additional details of the animal husbandry and experimental design can be found in the ARRIVE checklist (Checklist S1). These studies were carried out in accordance with the NIH Guide for the Care and Use of Laboratory Animals under Baylor College of Medicine IACUC approved protocol AN-716.

### Small RNA isolation and sequencing

Testicular small RNAs were isolated using the mirVana kit (Ambion, Austin, TX) and sequenced by Illumina-Solexa sequencing as described previously [44]. RNA quality and the presence of small RNAs were evaluated on a 2100 Bioanalyzer (Agilent). After passing the quality controls, 15 ug of total RNA was used in the Illumina DGE small RNA sample prep kit to synthesize a small RNA library. Small RNA populations were sequenced on the Illumina 1 G Genome Analyzer (University of Houston).

### Identification of alternatively processed miRNAs

Reads which differed from the mature miRNA sequences as described in microrna.org by 1 nucleotide at the 5′ end and mapped to the pre-miRNA sequence were identified as alternative 5′ isoforms. Those reads which matched 100% to the mature miRNA sequence but contained additional nucleotides A(n) or U(n) that did not match the pre-miRNA sequence were identified as alternative 3′ adenylated or uridylated miRNAs. Those reads which contained a single A to G mismatch within the mature miRNA sequence were identified as candidate ADAR-edited miRNAs. Finally those reads which contained additional uridines, but the surrounding sequence matched 100% to the mature miRNA (similar to an insertional polymorphism) were identified as miRNAs affected by internal uridylation. The ratio of alternative reads to canonical mature miRNA reads were analyzed for developmental trends.

### Identification of putative novel testicular miRNAs

Reads which do not match to mature miRNAs or other ncRNAs including snoRNAs, and tRNAs were mapped and contigs were assembled through the annotation pipeline described previously [39]. Criteria, including the presence of a stem-loop, reads indicating the presence of a miRNA star strand, and consistent 5′ end processing, were used to rank candidate miRNAs. We initially evaluated candidates independent of size; however, to achieve high confidence miRNAs we ultimately chose to subject candidates to a size cutoff of <25 nt present in *Gasz^−/−^* controls to exclude possible novel piRNAs.

## Results

### Developmental analysis of testicular miRNAs by deep sequencing

**Figure 1:**
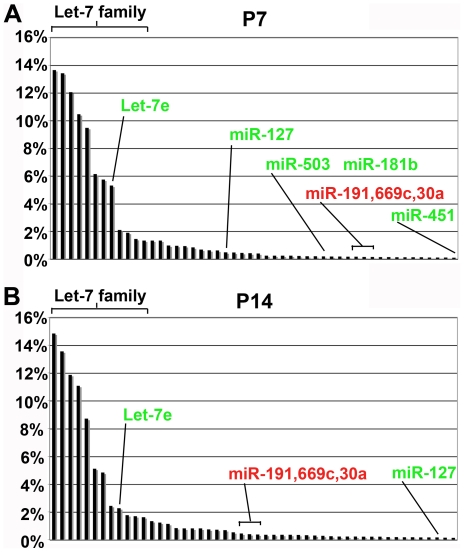
The MicroRNA signature of the testis differs over prepubertal development. MicroRNA signature of postnatal day 7 (P7) (A) and 14 (P14) (B) testes. The top 25 miRNAs (by percent read abundance) at each age were plotted. Representative miRNAs are labeled with those with greater abundance at P7 (putative spermatogonial role) in green and at P14 (putative spermatocyte role) in red. Levels at P10 were intermediate between P7 and P14 indicating a lack of miRNAs specific to meiotic initiation. ∼80% of all miRNAs in the testis at all three time-points were let-7 family miRNAs.

Using Illumina-Solexa deep sequencing, we analyzed the small RNA populations in testes of mice on postnatal day 7 (P7), 10 (P10), and 14 (P14) to assess piRNA populations in GASZ null mice compared to controls [44]. In the course of the analyses of our piRNA findings we recognized that analysis of the miRNA population in controls might provide an insight into their roles in pubertal spermatogenesis. We identified reads belonging to the 682 mature miRNAs (608 pre-miRNAs) present in miRBase 13.0 version (http://www.mirbase.org/) and used these to describe miRNA signatures for these ages, similar to our characterization of human ovarian miRNAs [39]. Since biologically effective changes in miRNA suppression of respective mRNA targets appears to have some minimal threshold, we initially focused on the top 50 miRNAs by read abundance at these three time-points (Figure 1A-B, Table S1). We initially hypothesized that meiotic initiation at P10 may be correlated with a unique miRNA signature; however, we found a lack of evidence for such an enrichment arguing against possible miRNA-mediated initiation of meiosis at P10. Therefore, we focused our subsequent analysis on a comparison between P7 and P14, identifying those miRNAs that showed greater than a two-fold enrichment at P7 or P14 as potential candidate miRNAs important for spermatogonial and spermatocyte functions.

**Figure 2:**
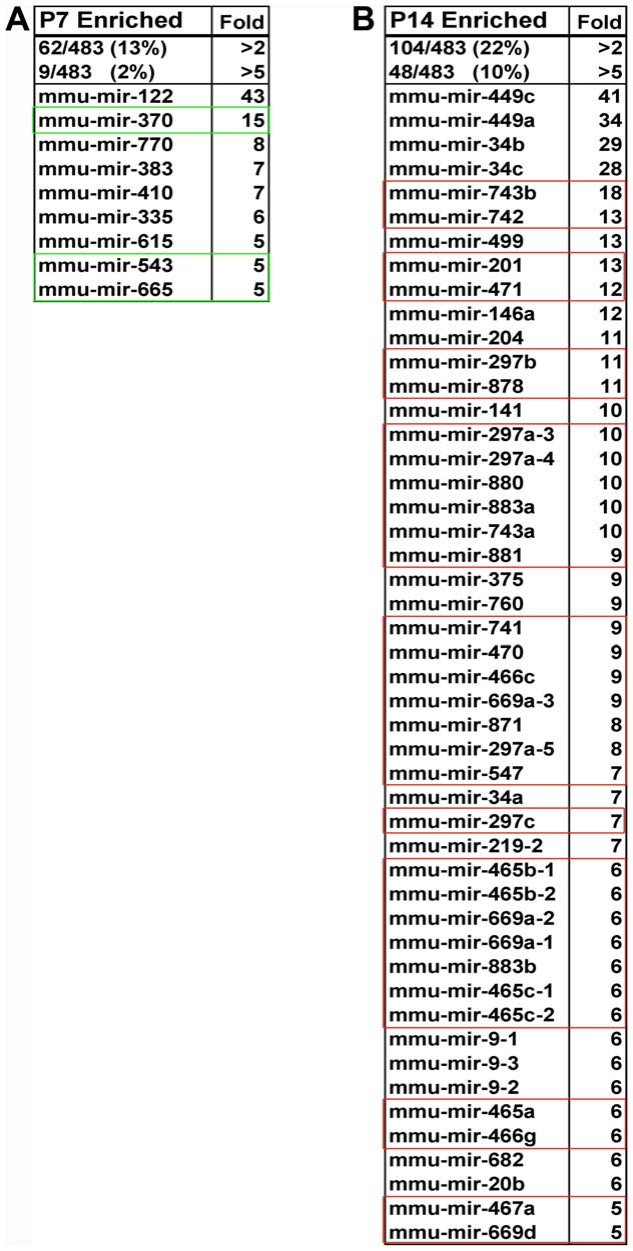
MicroRNAs with the most dramatic enrichment are associated with chromosomes 2, 12, and X. Roughly 2% of miRNAs were enriched more than five-fold at P7 (left). Of these miR-122 was the most enriched (43-fold). By contrast, nearly 10% of miRNAs were enriched to the same degree at P14 (right). The most enriched was miR-449c (41-fold). A number of miRNAs most induced at P7 are located on chromsome 12 (boxed in green) while those most induced at P14 cluster on chromosomes 2 and X (boxed in red).

Several abundant miRNAs showed greater than a two-fold enrichment at P7 (i.e., let-7e, 127, 181b-2, 503, and 181b-1), while those showing the greatest fold enrichment (i.e., mir-122, 370, 770, 383, 410, 335, 615, 543, 665) were generally expressed at modest levels (Figure 2A). By contrast, more than half of the miRNAs most enriched at P14 (i.e., 449c, 34b, 34c, 743b, 471, 204, 878, 880, 883a, 743a, 881, 375, 760, 741, 470, 871, 465b-1, 465b-2, 883b, 465c-1, 465c2, 465a, 467a) were abundantly expressed (Figure 2B). It is also notable that those miRNAs with the most dramatic enrichment at P14 were clustered on a single region of the X chromosome (discussed in greater detail below). MicroRNAs are often enriched either early (in stem cells/early transit amplifying cells) or late (in differentiating cells) in the differentiation process. Consistent with the latter, many of the miRNAs abundant at P14 were also previously reported as abundant at P21 and adult testes by low throughput methods [15] although the profiles were distinct. Thus the miRNA pools within pre-meiotic and meiotic are likely to be different, and a subset of miRNAs arising from the X chromosome during meiosis may continue to remain prominent in post-meiotic spermatogenesis.

### X chromosome miRNAs escape meiotic sex chromosome inactivation

**Figure 3:**
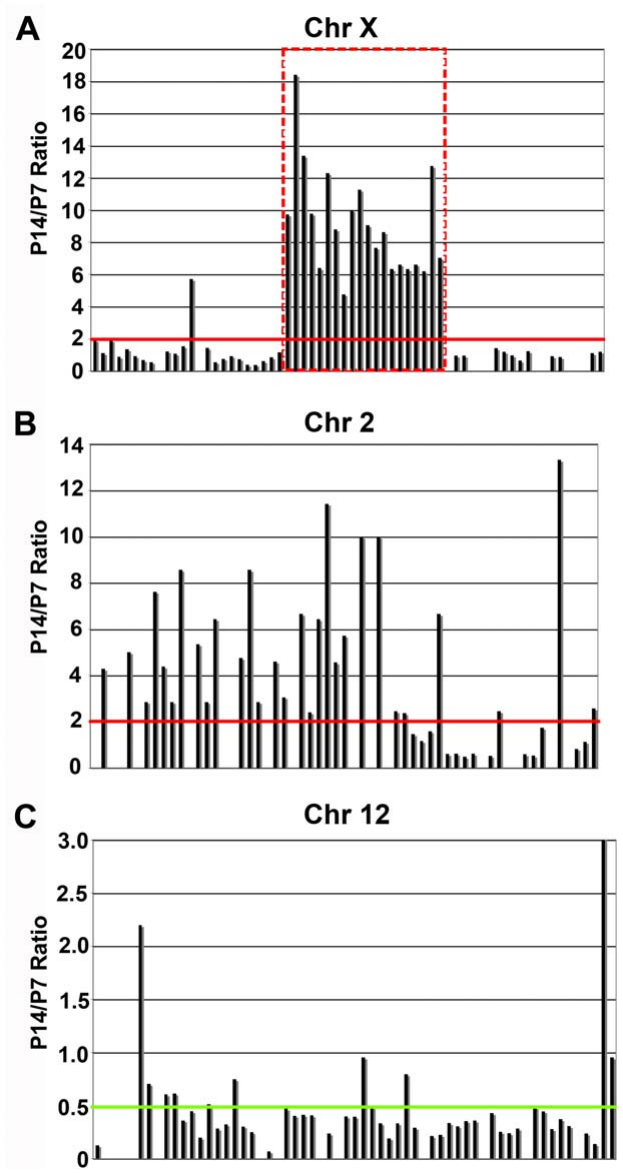
Chromosomes 2, 12, and X predominant source of pubertal-associated testis miRNAs. (A–C) Plots of miRNA expression ratios (P14/P7). (A) A large cluster miRNAs on chromosome X (ChrX) and most miRNAs on chromosome 2 (Chr2) are enriched for expression at P14 (ratio >2, red line). While those from chromosome 12 (Chr12) are enriched for expression at P7 (ratio <0.5, green line). Those on the distal arms of the X chromosome show enrichment at P7, as expected for genes showing the classical meiotic sex chromosome inactivation (MSCI) pattern of repression on P14. The large number of miRNAs on the mid-arm of chromosome X that escape this repressive process, displaying a greater abundance at P14, is in agreement with prior reports. We found a number of miRNAs falling within this cluster were ones that we had previously determined were misidentified as piRNAs in public databases (red dashed box).

Part of the dosage compensation that occurs between X- and Y-bearing male germ cells is a spermatocyte-specific alteration of sex chromatin through a process similar to X chromosome inactivation. The loss of mRNA transcription on the X chromsome during male meiosis is often compensated by intronless retrogene autosomal paralogs [46]. Prior reports by Yan and colleagues demonstrated that nearly all miRNAs on the X chromosome display continuous expression during meiosis in contrast to most protein-coding genes on the X chromosome [47]. We performed similar analysis using deep sequencing and found abundant reads for many X chromosome miRNAs. Intriguingly, when their position was mapped on the X chromosome, domains of developmental expression patterns were seen in which transcription predominated at either P7 or P14. The intergenic distances for most are large enough (>5 kb) to indicate that they are not transcribed from a common primary transcript unlike the miR-17∼92 cluster. In our prior analysis of piRNAs [44], we had observed a number of miRNA variants, previously missanotated as piRNAs, derived from this cluster on Xq. This region (including mir-743a to mir-547) represents a discontinuity in the synteny between rodents and primates. However, the primate X chromosome contains an analogous cluster of clade-specific miRNAs (hsa-mir-890 to hsa-mir-514-3). In addition to the X chromosome, we also found enrichment for miRNAs derived from chromosome 2 at P14 (Figure 3B) and for chromosome 12 at P7 (Figure 3C). In the mouse, miRNAs are non-randomly distributed over the genome with 40% deriving from these three chromosomes (Table 1). During mouse pubertal spermatogenesis the miRNA complement comes predominantly from limited chromosomal domains, shifting from expression of chromosome 12 at P7 to chromosomes 2 and X at P14.

**Table 1:** Positional analysis of pubertal miRNA expression.

Chr	Total	% Total	P7 high	% P7 high	P14 high	% P14 high
**1**	21	4%	2	3%	7	7%
**2**	**59**	**12%**	1	2%	**29**	**29%**
**3**	20	4%	0	0%	5	5%
**4**	22	5%	0	0%	6	6%
**5**	15	3%	1	2%	1	1%
**6**	21	4%	1	2%	3	3%
**7**	26	5%	5	8%	5	5%
**8**	15	3%	3	5%	0	0%
**9**	19	4%	0	0%	6	6%
**10**	10	2%	1	2%	0	0%
**11**	28	6%	2	3%	3	3%
**12**	**61**	**13%**	**37**	**61%**	0	0%
**13**	20	4%	0	0%	5	5%
**14**	21	4%	1	2%	3	3%
**15**	14	3%	1	2%	0	0%
**16**	17	4%	2	3%	1	1%
**17**	11	3%	1	2%	1	1%
**18**	11	2%	1	2%	1	1%
**19**	7	1%	0	0%	4	4%
**X**	**64**	**13%**	2	3%	**21**	**21%**
**Total**	482		61		101	

MicroRNAs are distributed nonrandomly over the mouse genome with predominant enrichment on chromosomes 2, 12, and X (in bold). Most miRNAs on chromosome 12 were enriched at P7, while those on chromosomes 2 and X are enriched at P14.

### Analysis of miRNA hairpin cleavage products

**Figure 4:**
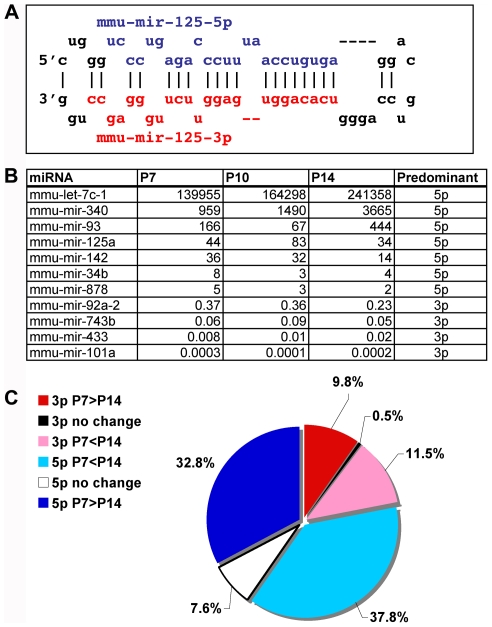
Mature miRNAs and star strands are differentially expressed in the testis. (A) Example miRNA identifying the 5p (red) and 3p (blue) miRNAs that will result from Dicer cleavage of the mmu-mir-125a pre-miRNA hairpin. Levels of 5p and 3p often are dramatically different in abundance in non-reproductive tissues, producing a 5p/3p ratio different from 1. (B) Shown are representative miRNAs in which reads were detected for both mature 5p and 3p sequences. Contrary to one report arguing for equivalent levels of both strands in the testis, we found that the pubertal testis was similar to other non-reproductive tissues. 73–87% of miRNAs displayed a 5p/3p ratio <0.5 or >2 (range 0.0001–240,000), consistent with differential stability of most 5p/3p miRNA pairs. (C) Most miRNAs showed predominant expression of either 5p or 3p reads. In 87% of miRNAs the 5p miRNA was more abundant compared to 3p (13%). Among the 5p miRNAs roughly one third were enriched at P7 (blue), P14 (teal), or unchanged (gray). Equal amounts of the 3p miRNAs were enriched at P7 (red) and P14 (pink).

Dicer cleavage of the pre-miRNA yields two products, the 5′ and 3′ portions of the base-paired regions of the stem-loop. Initially, it was believed that only one strand (guide) is incorporated into the RISC effector complex while the other strand (star) was nonfunctional and degraded. Now both mature miRNAs derived from the pre-miRNA are believed may have activity against targets. Due to speculation that there might be differential processing during testicular development or differences in the relative stability of the two strands in the testis, we analyzed the 5p and 3p miRNA strands during prepubertal testis development. The representative 5p (blue) and 3p (red) sequences from mouse miR-125 are shown (Figure 4A). We found that 359 of the 465 mouse pre-miRNAs, produced reads from one or both strands during P7-P14 of testicular development. After calculating the ratio of 5p to 3p reads from each processed pre-miRNA, we determined that the majority (>86%, Figure 4B and Table S2) showed a ratio that was significantly different from one at all three ages (range 0.0001–780,000). This indicates that the testis is not distinct from other organs with respect to pre-miRNA processing, showing differential abundance of the hairpin cleavage products (5p or 3p) for nearly all pre-miRNAs. Most reads derive from the 5p half of their respective pre-miRNAs. Since the majority of reads in the testis at the ages assessed are composed of let-7 family miRNAs, predominantly represented by their 5p reads, this further inflates this bias. In very rare cases (<1%), the strand preference changed over development, but most showed the same preference at all three time-points. We further assessed the developmental differences (P7 vs. P14) in the abundance of 5p and 3p miRNAs individually. This demonstrated that roughly an equal number of 5p miRNAs were increasing with age (32.8%) as decreasing (37.8%) with a small amount (7.6%) remaining constant over time (Figure 4C). A similar distribution of developmental patterns was seen on 3p miRNAs. We also detected 24 examples (7%) in which the developmental pattern of 5p and 3p miRNAs from the same pre-miRNA were in discordance. Thus we reject the hypotheses that a) the selection of the more abundant strand (5p vs. 3p) may shift and b) the stability of all miRNAs are coordinately regulated over pubertal testicular development.

### Alternatively processed miRNAs

We determined to capture developmental miRNA variation in multiple locations relative to the mature miRNA including 5′ end variation, internal editing, and nontemplated nucleotide addition to the 3′ end. We summarize the major events in which >5% of the reads are of the edited form (Table S3). To address 5′ end variation, we took an approach similar to the discovery of genomic insertions or deletions. The reads mapping with up to two nucleotide mismatches to mature miRNAs, but which matched the pre-miRNA (and genomic sequence), resulting from the utilization of distinct 5′ end cleavage sites by Dicer or Drosha were identified. Prior reports of adult mammalian tissues, including testis, describe 5′ end heterogeneity as affecting 8% of all miRNA reads, with greater variability on star strands [19]. We found that (Table S4) alternative 5′ end processing was rare with the exception of twenty miRNAs in which the 5′ cleavage variant was elevated at P7 relative to P14. Reads which matched the miRNA precursor with 1–3 mismatches were designated internally edited miRNA isoforms (Table S5). Most high abundance (10^4^ reads) miRNAs such as let-7 family member let-7b-5p showed very little internal editing. However its less abundant (10^3^ reads) star strand let-7b-3p, showed a developmental shift in editing at position 15 (A>G), likely to represent an ADAR-dependent event. At P7 the levels were 26% of the total let-7e-3p reads, but this declined on P14 to 15%. The effect of this editing event would be to enhance the inhibition of target at P14 relative to P7. Since Let-7 family members are believed to restrain proliferation reduced effectiveness of let-7 against its targets due to editing may be beneficial in the predominantly mitotically active testis at P7. In contrast to the editing of let-7b-3p, 34b-5p and 376b-3p, four of eighteen miRNAs showed a peak of editing at P10 and the eleven others showed a developmental increase from P7 to P14. Reads matching the mature miRNA sequence with additional 3′ (A)n or (U)n that did not match the pre-miRNA sequence were designated as 3′ edited miRNAs (Table S6). Slightly more than half (61%) of the thirty four miRNAs in this category showed a greater proportion of their edited forms at P14. Overall variant miRNAs compose a greater proportion of the miRNA pool at later developmental stages, suggesting that miRNA effects may weaken with increasing pubertal testicular development.

### Discovery of novel testicular miRNAs

We subjected our reads that mapped to pre-miRNA sequences (putative star strands) as well as those that did not map to a known mature miRNA to a miRNA discovery pipeline described in Creighton et al. 2009 that identified those reads whose surrounding 200 nucleotides had some capacity to fold into a double-stranded RNA using the Vienna secondary structure prediction package. We screened for those hairpins that captured a number of reads typically mapping to one side of the stem, rejecting those which did not cluster tightly, overlapped with the loop, or mapped to known non-coding RNAs. We identified 198 putative novel miRNAs and determined their developmental abundance (Tables S7, S8, S9). These were assigned to several categories based on a) their ability to be produced by predicted Drosha and Dicer cut sites and b) the presence of a putative star strand. We found 107 high confidence novel miRNAs, 104 lacking star strands, and three (76, 131, 143) with a corresponding star strand. Thirteen of 107 (12%) of the high confidence class mapped to repetitive elements including SINEs, LINEs, LTRs, and DNA transposons. These candidates likely represent endo-siRNAs, piRNAs, or SINE-associated small RNAs rather than true miRNAs. Since 17 of the 52 (32%) that met fewer criteria - “potential miRNAs” - were also mapped to repetitive elements, this provides some validation for the cut site criteria. Most of the candidates were expressed at very low levels while a significant fraction of those expressed at a higher level were associated with repeats (Table S9). Greater than 75% of all high confidence candidates were expressed at P7 or P10 alone or P7-P10, which would have been most likely undetectable in the prior sequencing study involving adult testis [19]. The least differentiated cells in the testis may be the source of undiscovered miRNAs just as embryonic stem cells possess miRNAs distinct from those in adult tissues [48]. An additional 143 pre-miRNAs have been described in the latest miRBase 15.0 version but only candidate 185 has been identified as mir-3099-3p, first isolated in newborn ovaries [40]. We assessed evolutionary conservation of the 5′seed sequence of our candidates to rat and human syntenic regions using the UCSC genome browser and found relatively few were conserved to human (26/198 or 13%) although nearly half (45%) were conserved to rat. Most candidates had 5′seed sequences distinct from known miRNAs; however the following candidates, conserved with humans, were similar to those in parentheses: 5 (miR-190/190b), 19 (miR-29b-2), 92 (miR-1195), 93 (miR-134), 132 (miR-486), and 183 (miR-345-3p). Those that were abundant but not conserved with humans (34, 69, 76, 88, 94, 100, 105, 110, 144, 176) had novel 5′seeds with the exception of 110, which was similar to miR-411. Another criteria that may increase our confidence in candidates representing miRNAs rather than other types of ncRNA could include close linkage to known miRNAs (<5000 bp). Two of our candidates show this association (85 to miR-700 and 90 to miR-805). Nine pairs of candidate reads displayed close linkage to other candidates, all mapped to mRNA exons. We assigned these candidates overlapping with exons as gene-associated piRNAs rather than miRNAs, which are more commonly contained within introns when they overlap an mRNA. After excluding those candidates mapping to exons or repetitive elements, 75 candidates remained. Of these, 12 were 24 nt or less while the remainder were 25–30 nt in length. A length assessment of all mouse mature miRNAs identifies only 1.3% of miRBase miRNAs larger than 24 nts (4.5%<20 nt and 94.2% 20–24 nt). Since we have observed that the number of miRNA reads is proportionally increased in GASZ null testes lacking piRNAs [44], we tested the value of excluding those candidates which are absent or not increased in GASZ null testes as probable piRNAs. Eighty-three percent (10/13) of the candidates <25 nt long were increased in GASZ null testes relative to controls, while of the larger size category only candidates 5 and 90 (3%) showed a similar increase. Forty percent of the candidates in the larger size were determined to partially overlap with known piRNAs or map within piRNA clusters. Thus, we identified 11 putative novel testis miRNAs with high confidence and detected miR-3099-3p previously described in newborn ovary (Table 2). We believe that the remaining 64 are likely piRNAs. Using the 5′seed sequence target prediction analysis in TargetScan 5.1, we analyzed the targets of the 11 putative novel candidates. Most showed a very restricted target repertoire between 4–100 targets. A small fraction of targets overlapped between the novels, but was not correlated with similar developmental pattern.

**Table 2:** Identification of putative novel testis-expressed miRNAs.

Candidate miRNA	Mature sequence	length	chr:start-stop (strand)	Intronic to mRNA
149	UGGACACUGGAGAGAGAGCUUUU	23	chr4:58453895-58453917 (−)	Lpar1
139	UGGGUAGACUGAGCCUGGCUGA	22	chr4:133904334-133904355 (+)	Slc30a2
138	UGUUGAUCGGCGUUCUUGGUUAUG	24	chr7:6594138-6594161 (−)	-
185/mir-3099-3p	UAGGCUAGAGAGAGGUUGGGGA	22	chr7:6756349-6756370 (+)	Usp29
19	GAGCACCCCAUUGGCUACCCACA	23	chr7:108030821-108030843 (−)	Arhgef17
91	ACCGGGGACUGCGGCUUUCAGU	22	chr7:133723020-133723041 (−)	Cln3
71	GCUGGAGGAUGAAGUAAGGAGUGA	24	chr8:112363200-112363223 (+)	Ap1g1
190	UGGCGGCAGUCAGGAUACCUGU	22	chr11:117863593-117863614 (+)	Pgs1
186	CUUAGAUCGAUGUGGUGCU	19	chr12:37542836-37542855 (+)	-
148	AACUGAGUUGAAGGCAAAGGU	21	chr15:40862391-40862411 (+)	Zfpm2
120	ACGCCCUUCCCCCCCUUCUUCA	22	chr15:84781975-84781996 (−)	5031439G07Rik
196	AGGGGAGCUAGGUAGAAAGCCA	22	chr19:4623929-4623950 (−)	Rce1

After excluding known miRNAs, clusters of Solexa reads showing evidence of hairpin formation were identified (203 candidates). We set strong inclusion criteria for maximal length (<24 nt) and enrichment in GASZ^−/−^ testes due to piRNA depletion. Seventeen putative novel miRNAs were identified, 12 of which displayed enrichment in GASZ null testes. Most are expressed at relatively low level, have limited conservation, and map to introns of autosomal genes.

## Discussion

The mouse testis displays a distinct microRNA profile during prepubertal development with miRNAs enriched putatively in spermatogonia (e.g., miR-122) and spermatocytes (e.g., miR-409c). MiRNAs from particular chromosomes are active at specific times during spermatogenesis (e.g., chromosome 12 at P7 and chromosomes 2 and X at P14). We have identified 11 putative novel testis-expressed miRNAs in addition to the miRNA variants on the X chromosome described previously [44]. Compared to adult testis sequencing results, the complexity of the juvenile miRNA profiles are much lower. Let-7 family members contribute 80% of juvenile miRNA reads but compose only 11% of adult testis reads. While the top 50 miRNAs by abundance in the adult testis include let-7 family members and many X chromosomally encoded miRNAs, 167 miRNAs are >5-fold enriched in adult testes compared to P14 testes. Many of these miRNAs are also enriched at P14 compared to P7, but a number of miRNAs are specifically enriched in the adult including the mir-17 to -92a-1 cluster on chromosome 14, mir-135a, mir-135b, mir-190, and mir-215.

It is intriguing that newborn ovaries, predominantly composed of meiotic oocytes, show a similar enrichment for expression of miRNAs from chromosomes 2 and X, potentially indicating some common regulation of meiotic miRNAs in both sexes [40]. However, the particular cluster on the X chromosome highly expressed in ovaries (miR-450b to miR-322) is centromeric to a similar cluster in testes (miR-743a to miR-465a). Furthermore, the majority of the ovarian cluster is conserved with human, with the exception of mir-322, nearest to the male cluster, whereas, the male cluster is almost entirely rodent-specific miRNAs. One might be tempted to speculate that the enrichment of miRNAs on mouse chromosomes 2 and X may suggest a possible role in spermatocyte function.

Although i12p, duplication, or increased expression of pluripotency factors encoded on the p arm of human chromosome 12 is common to seminomas [49], [50], the enrichment of mouse chromosome 12 miRNAs at P7 does not have an obvious importance to testicular cancer since this mouse chromosome is syntenic to human chromosome 14. The only gene mapped to mouse chromosome 12 and human chromosome 14 with polymorphisms associated with azoospermia is MLH3, which causes a meiotic arrest [51]. However, we speculate that miRNAs from human chromosome 14 may be abundant in differentiated spermatogonia and deficient in testicular tumors arrested at an earlier stage of germ cell development. Assessment of possible mechanistic action of miRNAs abundant in human testicular cancer would be greatly benefited by comparison of the miRNA complement from newborn mouse testis or isolated gonocytes with their corresponding mouse testicular cancer models.

Differential processing of some pre-miRNAs, leading to tissue-specific differences in the relative abundance of their 5p and 3p mature miRNAs, has been described by deep sequencing [19]. These differences could theoretically reflect differential pre-miRNA processing in dividing versus post-mitotic cells or cell cycle-specific processing, the abundance of which differs by tissue. Such regulation could derive from regulation of pre-miRNA-binding proteins. The relatively synchronous development of the first wave of spermatogenesis should allow detection of such regulation during the shift in the germ cell compartment from a mitotic to meiotic state and be favorable to purification of the processing regulator. However, we found that the mature miRNA strand (5p versus 3p) that was predominant does not appear to differ during mitotic or meiotic testicular development, nor does destabilization of microRNA star strands appear to be developmentally regulated in the testis during this interval. By contrast, about 20% of miRNAs expressed in the adult testis switch from the strand predominant at P14 (Table S10). Seventy-five percent of the shifts favor the increase of the star strand in the adult testis, independent of the predominant strand (5p or 3p) at P14. Although there is no evidence for a global shift of processing (i.e., favoring the 5p at earlier and the 3p at later time-points), the same pre-miRNA may be processed distinctly in early and late spermatogenesis. Were transcription of the pre-miRNA to remain constant, preferential accumulation of the star strand could favor translational de-repression due to loss of the corresponding strand or inhibition of a distinct set of targets.

Analysis of miRNA editing in the testis showed that most types of editing events were higher at P14 than P7. Questions have been raised about the possibility that apparent editing of miRNAs outside the 5′ and 3′ ends could derive from Dicer-dependent processing of other ncRNAs, such as tRNAs, followed by 3′ uridylation or adenylation [45]. We were able to detect several miRNAs, which showed evidence of significant internal editing (>5%) in juvenile mice. Presumptive ADAR- or APOBEC-dependent affects were detected affecting adenosines and cytidines, respectively, but nearly half of internal editing events in juvenile testis miRNAs affects uridines and occurs at sites near the 3′ end. The necessity of ADAR-dependent editing is unclear since no reproductive defects have been described in ADAR1- or ADAR2-deficient mice, but SPNR (spermatid perinuclear RNA-binding protein) has similarity to ADARs outside the deaminase domain and is essential to spermatid function [52], [53].

Twenty juvenile mouse testis miRNAs display variation in 5′ end cleavage including let-7a-1-5p. However, we did not identify those from adult testis such as miR-133, -223, or -155 [19]. Confounding our analysis of 3′ variation, poly(A) or poly(U) tracts in the pre-miRNA follow the canonical 3′ cleavage site in 17% and 35% of mouse miRNAs. 3′ end cleavage resulting in longer miRNAs may mimic post-transcriptional adenylation or uridylation, conferring inherent instability upon 3′ cleavage variants. For those miRNAs in which 3′ end adenylation or uridylation could be distinguished from differential cleavage site selection, we found a relative increase in this modification from P7 to P14. Ninety-one miRNAs in the adult testis display 3′ nucleotide addition (A or U, range 10%–100%) [19]. The majority of miRNAs uridylated in juvenile testes were distinct from those modified in adult testis, but some were affected in both including mir-24-1, -24-2, -103-1, 103-2, -199a, and -342 [19]. Abundant P14 X-chromosomally encoded miRNAs do not display 3′ uridylation with the exception of let-7f-2. The increase in uridylation at P14, decreasing target specificity and miRNA stability, argue for a relatively lower effectiveness of these miRNAs against target at this time. However, differences in miRNA uridylation between P7 and P14 are modest compared to the high degree of uridylation of specific miRNAs in the adult testis. Since the majority of cells in the adult testis are spermatids, these modifications may be targeted to pre-miRNAs by spermatid proteins that bind to the loop domain of pre-miRNAs similar to LIN28 binding to let-7 pre-miRNAs. The semi-synchronous nature of the first wave of spermatogenesis may facilitate in the identification of possible testicular cofactors for miRNA modifying enzymes and such factors may be associated with the chromatoid body.

By utilizing Next Generation Sequencing, our studies characterized the complete miRNAome and its editing *in vivo* during prepubertal testicular development. The peculiar behaviors of a large number of miRNAs expressed in the germline (i.e., resistance to meiotic sex chromosome inactivation and strong chromosomal association) argues for the need to identify additional miRNA transcriptional regulators capable of acting in cis on chromosomes 2, 12, and X. We expect that the developmental associations with these three chromosomes in this study will frame future investigations of translational control of spermatogonial or spermatocyte mRNAs. The very low copy number and poor evolutionary conservation of the novel miRNAs identified in this study is consistent with other attempts to identify tissue-specific miRNAs, suggesting that few additional abundant conserved novel miRNAs remain undiscovered in the mouse genome. While our miRNA catalog did not identify a class of miRNAs that regulates meiosis initiation, they do provide a normative control by which to evaluate miRNA changes in murine and human testicular cancers. Comparison of these findings in the pubertal mouse testis to published studies of adult mouse testis highlights the need to focus future studies of the regulatory consequences of miRNA editing during the terminal differentiation of spermatids and their potential physical connection to the chromatoid body and its associated translationally-regulated spermatid mRNAs. Our findings of miRNA regulation in spermatogenesis may be generalized to other somatic cell types, most notably to neurons. Analogous to the translational regulation required to assemble structures accessory to the sperm tail axoneme, the brain displays a similar temporal-spatial compartmentalization of translation associated with neuronal axons and an even greater fraction of edited miRNAs. Future efforts to identify the mechanism for selectivity in editing of miRNAs during testicular development may ultimately offer potential new avenues to therapeutic intervention in human infertility and neurologic disorders.

## Supporting Information

Table S1 **Developmental analysis of miRNA expression in the testis (P7-P14).** Excel table containing the number of reads, percent of total miRNA reads, chromosomal position, and quantification of modified reads at internal and 3′ positions. The number of reads at each age were normalized by calculation of their percentage in the miRNA pool at each age. Ratios were calculated to compare the relative expression. Those miRNAs with significant effects (>2-fold enrichment at P7 or at P14) are bolded. Str, strand; Chr, chrosomosome.
(PDF)

Table S2 **Developmental analysis of 5p vs. 3p miRNAs in the testis (P7-P14).** Excel table containing the number of reads, percent of total miRNA reads, chromosomal position, and predicted secondary structures of candidate novel testicular miRNAs. The number of reads at each age were normalized by calculation of their percentage in the miRNA pool at each age. Ratios were calculated to determine the bias of pre-miRNA processing (5p/3p) at each age. The predominant miRNA (i.e. the non-star strand) was identified. Developmental change of both 5p and 3p mature miRNAs was also calculated using ratio calculation and annotated as increasing (i) or decreasing (d) from P7 to P14.
(PDF)

Table S3 **Editing of miRNAs during prepubertal testicular development.** Four types of editing were evaluated by assigning reads that were not an exact match to the mature miRNA sequence: a) alteration of 5′ end cleavage, b) A to G transitions representing putative editing by ADARs, c) internal insertions of uridine by an unknown process, and d) 3′ addition of A(n) or (U)n. [Indel, insertion/deletion]
(PDF)

Table S4 **5′ cleavage variants of miRNAs during prepubertal testicular development.** 5′ variants are generally highest at P7 in the juvenile testis but represent a small fraction of total reads.
(PDF)

Table S5 **Internal editing of miRNAs during prepubertal testicular development.** Most internal editing events are highest at P14 in the juvenile testis, but other patterns are detected less frequently. Increased editing is associated with declining levels of the miRNA in 55% of cases. Uridine was the most common base affected. The affected positions are bolded within the mature miRNA sequence.
(PDF)

Table S6 **3′ nuclotide addition to miRNAs during prepubertal testicular development.** 3′ nucleotide addition increased over juvenile testis development (61% of cases), but the portion of modified reads represent a small fraction of total reads.
(PDF)

Table S7 **Hairpin candidate evaluation.** All testicular small RNA reads not identified as known miRNAs were analyzed for 1) their ability to form a stem loop structure, 2) minimum free energy less than -20 kcal/mol, 3) strong clean signal in the specific region of the 15–25 nt reference hairpin, 4) signals should not fall in the loop, 5) predicted Drosha and Dicer cut sites must be able to yield a mature miR sequence that matches the read, 6) stable 5′ end (±1 nt), 7) highly variable 3′ end, 8) presence of star sequence at lower copy number and matching miR with 3′ 2 nucleotide overhang, 9) does not map to rRNA, tRNA, snoRNA, or snRNA. Those matching all nine criteria were designated high-confidence novel miRNAs with star sequences. Those reads that passed all but criteria 6–8 were designated high-confidence miRNA without star sequence. Those that also did not pass criteria 5 were designated as potential miRNAs and the reads that failed criteria 1–4 were identified as non-candidates. Manually curated Dicer and Drosha cleavage sites on the hairpins were marked in blue and red lines.
(PDF)

Table S8 **Description of novel testicular candidate miRNAs.** Hairpin candidates were mapped to the mouse genome (mm9) using the UCSC Blat tool. Those that overlapped with other genomic elements were identified including repetitive elements (LINE, SINE, LTR). Conservation of the SEED sequence with syntenic sequences on the rat (R.n.) and human (H.s.) was assessed through the UCSC genome browser (Y, yes; N, no; M, mutated).
(PDF)

Table S9 **Developmental expression of candidate miRNAs.** The number of reads from two testes of each age and genotype (GASZ^+/−^ or GASZ^−/−^) are shown with the reads assigned to the 5p and 3p sections of the hairpin. A summary of their developmental pattern is given. The majority of those expressed at 50 reads or greater had some similarity to repetitive elements, suggestive they represent piRNAs, endosiRNAs, or SINE-associated small RNAs.
(PDF)

Table S10 **Comparison of predominance of 5p vs. 3p between P14 and adult testes.** The expression of 5p versus 3p mature miRNAs were compared between this study and adult testes. Those miRNAs which shifted from a high to low 5p/3p ratio or vice-versa are shown with the abundant mature miRNA at each time-point.
(PDF)

Checklist S1 **The ARRIVE Checklist.**
(DOC)

## References

[1] Ambros V (2001). microRNAs: tiny regulators with great potential.. Cell.

[2] Bartel DP, Chen CZ (2004). Micromanagers of gene expression: the potentially widespread influence of metazoan microRNAs.. Nat Rev Genet.

[3] Wienholds E, Plasterk RH (2005). MicroRNA function in animal development.. FEBS Lett.

[4] Carthew RW (2006). Molecular biology. A new RNA dimension to genome control.. Science.

[5] Rajewsky N (2006). microRNA target predictions in animals.. Nat Genet.

[6] Grimson A, Farh KK, Johnston WK, Garrett-Engele P, Lim LP (2007). MicroRNA targeting specificity in mammals: determinants beyond seed pairing.. Mol Cell.

[7] Viswanathan SR, Daley GQ, Gregory RI (2008). Selective blockade of microRNA processing by Lin28.. Science.

[8] Hayashi K, Chuva de Sousa Lopes SM, Kaneda M, Tang F, Hajkova P (2008). MicroRNA Biogenesis Is Required for Mouse Primordial Germ Cell Development and Spermatogenesis.. PLoS ONE.

[9] Gillis AJ, Stoop HJ, Hersmus R, Oosterhuis JW, Sun Y (2007). High-throughput microRNAome analysis in human germ cell tumours.. J Pathol.

[10] Looijenga LH, Gillis AJ, Stoop H, Hersmus R, Oosterhuis JW (2007). Relevance of microRNAs in normal and malignant development, including human testicular germ cell tumours.. Int J Androl.

[11] Kotaja N, Bhattacharyya SN, Jaskiewicz L, Kimmins S, Parvinen M (2006). The chromatoid body of male germ cells: similarity with processing bodies and presence of Dicer and microRNA pathway components.. Proc Natl Acad Sci U S A.

[12] Kleene KC (2003). Patterns, mechanisms, and functions of translation regulation in mammalian spermatogenic cells.. Cytogenetic & Genome Research.

[13] Kotaja N, Sassone-Corsi P (2007). The chromatoid body: a germ-cell-specific RNA-processing centre.. Nat Rev Mol Cell Biol.

[14] Yu Z, Raabe T, Hecht NB (2005). MicroRNA Mirn122a reduces expression of the posttranscriptionally regulated germ cell transition protein 2 (Tnp2) messenger RNA (mRNA) by mRNA cleavage.. Biol Reprod.

[15] Ro S, Park C, Sanders KM, McCarrey JR, Yan W (2007). Cloning and expression profiling of testis-expressed microRNAs.. Dev Biol.

[16] Mishima T, Takizawa T, Luo SS, Ishibashi O, Kawahigashi Y (2008). MicroRNA (miRNA) cloning analysis reveals sex differences in miRNA expression profiles between adult mouse testis and ovary.. Reproduction.

[17] Novotny GW, Nielsen JE, Sonne SB, Skakkebaek NE, Rajpert-De Meyts E (2007). Analysis of gene expression in normal and neoplastic human testis: new roles of RNA.. Int J Androl.

[18] Landgraf P, Rusu M, Sheridan R, Sewer A, Iovino N (2007). A mammalian microRNA expression atlas based on small RNA library sequencing.. Cell.

[19] Chiang HR, Schoenfeld LW, Ruby JG, Auyeung VC, Spies N (2010). Mammalian microRNAs: experimental evaluation of novel and previously annotated genes.. Genes Dev.

[20] Reid JG, Nagaraja AK, Lynn FC, Drabek RB, Muzny DM (2008). Mouse let-7 miRNA populations exhibit RNA editing that is constrained in the 5′-seed/cleavage/anchor regions and stabilize predicted mmu-let-7a:mRNA duplexes.. Genome Res.

[21] Kim DD, Kim TT, Walsh T, Kobayashi Y, Matise TC (2004). Widespread RNA editing of embedded alu elements in the human transcriptome.. Genome Res.

[22] Eisenberg E, Adamsky K, Cohen L, Amariglio N, Hirshberg A (2005). Identification of RNA editing sites in the SNP database.. Nucleic Acids Res.

[23] Slezak-Prochazka I, Durmus S, Kroesen BJ, van den Berg A (2010). MicroRNAs, macrocontrol: regulation of miRNA processing.. RNA.

[24] Nishikura K (2010). Functions and regulation of RNA editing by ADAR deaminases.. Annu Rev Biochem.

[25] Bass BL (2002). RNA editing by adenosine deaminases that act on RNA.. Annu Rev Biochem.

[26] Luciano DJ, Mirsky H, Vendetti NJ, Maas S (2004). RNA editing of a miRNA precursor.. RNA.

[27] Pfeffer S, Lagos-Quintana M, Tuschl T (2005). Cloning of small RNA molecules.. Curr Protoc Mol Biol.

[28] Blow MJ, Grocock RJ, van Dongen S, Enright AJ, Dicks E (2006). RNA editing of human microRNAs.. Genome Biol.

[29] Kawahara Y, Zinshteyn B, Sethupathy P, Iizasa H, Hatzigeorgiou AG (2007). Redirection of silencing targets by adenosine-to-inosine editing of miRNAs.. Science.

[30] Gottwein E, Cai X, Cullen BR (2006). A novel assay for viral microRNA function identifies a single nucleotide polymorphism that affects Drosha processing.. J Virol.

[31] Yang W, Chendrimada TP, Wang Q, Higuchi M, Seeburg PH (2006). Modulation of microRNA processing and expression through RNA editing by ADAR deaminases.. Nat Struct Mol Biol.

[32] Kawahara Y, Megraw M, Kreider E, Iizasa H, Valente L (2008). Frequency and fate of microRNA editing in human brain.. Nucleic Acids Res.

[33] Kawahara Y, Zinshteyn B, Chendrimada TP, Shiekhattar R, Nishikura K (2007). RNA editing of the microRNA-151 precursor blocks cleavage by the Dicer-TRBP complex.. EMBO Rep.

[34] Habig JW, Dale T, Bass BL (2007). miRNA editing–we should have inosine this coming.. Mol Cell.

[35] Ohman M (2007). A-to-I editing challenger or ally to the microRNA process.. Biochimie.

[36] Linsen SE, de Wit E, de Bruijn E, Cuppen E (2010). Small RNA expression and strain specificity in the rat.. BMC Genomics.

[37] Huang J, Liang Z, Yang B, Tian H, Ma J (2007). Derepression of microRNA-mediated protein translation inhibition by apolipoprotein B mRNA-editing enzyme catalytic polypeptide-like 3G (APOBEC3G) and its family members.. J Biol Chem.

[38] Morin RD, O'Connor MD, Griffith M, Kuchenbauer F, Delaney A (2008). Application of massively parallel sequencing to microRNA profiling and discovery in human embryonic stem cells.. Genome Res.

[39] Creighton CJ, Benham AL, Zhu H, Khan MF, Reid JG (2010). Discovery of novel microRNAs in female reproductive tract using next generation sequencing.. PLoS One.

[40] Ahn HW, Morin RD, Zhao H, Harris RA, Coarfa C (2010). MicroRNA transcriptome in the newborn mouse ovaries determined by massive parallel sequencing.. Mol Hum Reprod.

[41] Heo I, Joo C, Cho J, Ha M, Han J (2008). Lin28 mediates the terminal uridylation of let-7 precursor MicroRNA.. Mol Cell.

[42] Heo I, Joo C, Kim YK, Ha M, Yoon MJ (2009). TUT4 in concert with Lin28 suppresses microRNA biogenesis through pre-microRNA uridylation.. Cell.

[43] Hagan JP, Piskounova E, Gregory RI (2009). Lin28 recruits the TUTase Zcchc11 to inhibit let-7 maturation in mouse embryonic stem cells.. Nat Struct Mol Biol.

[44] Ma L, Buchold GM, Greenbaum MP, Roy A, Burns KH (2009). GASZ Is Essential for Male Meiosis and Suppression of Retrotransposon Expression in the Male Germline.. PLoS Genet.

[45] de Hoon MJ, Taft RJ, Hashimoto T, Kanamori-Katayama M, Kawaji H (2010). Cross-mapping and the identification of editing sites in mature microRNAs in high-throughput sequencing libraries.. Genome Res.

[46] Wang PJ (2004). X chromosomes, retrogenes and their role in male reproduction.. Trends Endocrinol Metab.

[47] Song R, Ro S, Michaels JD, Park C, McCarrey JR (2009). Many X-linked microRNAs escape meiotic sex chromosome inactivation.. Nat Genet.

[48] Gu P, Reid JG, Gao X, Shaw CA, Creighton C (2008). Novel microRNA candidates and miRNA-mRNA pairs in embryonic stem (ES) cells.. PLoS One.

[49] Reuter VE (2005). Origins and molecular biology of testicular germ cell tumors.. Mod Pathol.

[50] Tanaka K, Okamoto S, Ishikawa Y, Tamura H, Hara T (2009). DDX1 is required for testicular tumorigenesis, partially through the transcriptional activation of 12p stem cell genes.. Oncogene.

[51] Ferras C, Zhou XL, Sousa M, Lindblom A, Barros A (2007). DNA mismatch repair gene hMLH3 variants in meiotic arrest.. Fertil Steril.

[52] Schumacher JM, Lee K, Edelhoff S, Braun RE (1995). Spnr, a murine RNA-binding protein that is localized to cytoplasmic microtubules.. J Cell Biol.

[53] Pires-daSilva A, Nayernia K, Engel W, Torres M, Stoykova A (2001). Mice deficient for spermatid perinuclear RNA-binding protein show neurologic, spermatogenic, and sperm morphological abnormalities.. Dev Biol.

