# Supplementary material for: Analysis of MicroRNA Expression in the Prepubertal Testis
Source: PLoS One. 2010 Dec 29;5(12):e15317. doi: 10.1371/journal.pone.0015317 (PMC3012074; doi:10.1371/journal.pone.0015317)
Supplement: Table S1 — Developmental analysis of miRNA expression in the testis (P7-P14). Excel table containing the number of reads, percent of total miRNA reads, chromosomal position, and quantification of modified reads at internal and 3′ positions. The number of reads at each age were normalized by calculation of their percentage in the miRNA pool at each age. Ratios were calculated to compare the relative expression. Those miRNAs with significant effects (>2-fold enrichment at P7 or at P14) are bolded. Str, strand; Chr, chrosomosome. (PDF) [file pone.0015317.s001.pdf]

Mouse	Chr	start	end	Str	Pre-miRNA Accession	P7 Reads	P7 %	P10 Reads	P10 %	P14 Reads	P14 %	P10/P7	P10/P14	P14/P7
mmu-mir-0206	1	20669091	20669163	+	MI0000249	353	0.01%	92	0.00%	33	0.00%	0.43	1.61	**0.27**
mmu-mir-0133b	1	20672850	20672968	+	MI0000821	1	0.00%	0	0.00%	0	0.00%	0.00	0.00	0.00
mmu-mir-0030a	1	23279108	23279178	+	MI0000144	9148	0.16%	6695	0.19%	7414	0.36%	1.21	0.52	**2.31**
mmu-mir-0030c-2	1	23298540	23298623	+	MI0000548	813	0.01%	607	0.02%	484	0.02%	1.23	0.72	1.70
mmu-mir-0026b	1	74440884	74440968	+	MI0000575	3401	0.06%	2145	0.06%	1141	0.06%	1.04	1.09	0.96
mmu-mir-0375	1	74947232	74947295	-	MI0000792	70	0.00%	23	0.00%	219	0.01%	0.54	0.06	**8.93**
mmu-mir-0149	1	94746955	94747020	+	MI0000171	32	0.00%	6	0.00%	0	0.00%	0.31	0.00	0.00
mmu-mir-0128-1	1	130098938	130099007	+	MI0000155	720	0.01%	634	0.02%	387	0.02%	1.45	0.95	1.54
mmu-mir-0135b	1	134094665	134094761	+	MI0000646	1	0.00%	1	0.00%	1	0.00%	1.65	0.58	**2.86**
mmu-mir-0181a-1	1	139863032	139863118	+	MI0000697	8337	0.14%	3420	0.10%	1829	0.09%	0.68	1.08	0.63
mmu-mir-0181b-1	1	139863216	139863295	+	MI0000723	5025	0.09%	1718	0.05%	889	0.04%	0.56	1.12	0.51
mmu-mir-0488	1	160435756	160435864	+	MI0004633	1	0.00%	0	0.00%	1	0.00%	0.00	0.00	**2.86**
mmu-mir-0199a-2	1	164147945	164148054	+	MI0000713	77290	1.33%	29868	0.85%	16460	0.81%	0.64	1.05	0.61
mmu-mir-0214	1	164153499	164153608	+	MI0000698	1185	0.02%	411	0.01%	252	0.01%	0.57	0.94	0.61
mmu-mir-0689-1	1	169270532	169270640	-	MI0004654	7	0.00%	4	0.00%	1	0.00%	0.94	2.31	**0.41**
mmu-mir-0350	1	178702456	178702554	-	MI0000640	74	0.00%	102	0.00%	65	0.00%	2.27	0.91	**2.51**
mmu-mir-0194-1	1	187137198	187137264	+	MI0000236	3	0.00%	4	0.00%	0	0.00%	2.20	0.00	0.00
mmu-mir-0215	1	187137460	187137571	+	MI0000974	3	0.00%	3	0.00%	2	0.00%	1.65	0.87	1.90
mmu-mir-0205	1	195333657	195333724	-	MI0000248	28	0.00%	2	0.00%	7	0.00%	0.12	0.17	0.71
mmu-mir-0029b-2	1	196863234	196863314	+	MI0000712	32	0.00%	66	0.00%	50	0.00%	3.40	0.76	**4.46**
mmu-mir-0029c	1	196863741	196863828	+	MI0000577	193	0.00%	295	0.01%	250	0.01%	2.52	0.68	**3.70**
mmu-mir-0466f-1	2	10388571	10388664	+	MI0005507	0	0.00%	0	0.00%	1	0.00%	0.00	0.00	1.00
mmu-mir-0669f	2	10388856	10388976	+	MI0006287	2	0.00%	3	0.00%	3	0.00%	2.47	0.58	**4.28**
mmu-mir-0669e	2	10389122	10389240	+	MI0006300	0	0.00%	0	0.00%	0	0.00%	0.00	0.00	0.00
mmu-mir-0669b	2	10389417	10389513	+	MI0004666	0	0.00%	0	0.00%	0	0.00%	0.00	0.00	0.00
mmu-mir-0669d	2	10389970	10390090	+	MI0006281	4	0.00%	8	0.00%	7	0.00%	3.30	0.66	**5.00**
mmu-mir-0466f-2	2	10390302	10390395	+	MI0005508	0	0.00%	2	0.00%	2	0.00%	0.00	0.58	**2.00**
mmu-mir-0466f-3	2	10393580	10393673	+	MI0005509	1	0.00%	1	0.00%	1	0.00%	1.65	0.58	**2.86**
mmu-mir-0297a-5	2	10393881	10393970	+	MI0005490	3	0.00%	5	0.00%	8	0.00%	2.75	0.36	**7.62**
mmu-mir-0467c	2	10395558	10395654	+	MI0005512	43	0.00%	75	0.00%	66	0.00%	2.88	0.66	**4.38**
mmu-mir-0466b-1	2	10395846	10395927	+	MI0005502	6	0.00%	0	0.00%	6	0.00%	0.00	0.00	**2.86**
mmu-mir-0669a-3	2	10396060	10396168	+	MI0004668	1	0.00%	4	0.00%	3	0.00%	6.60	0.77	**8.57**
mmu-mir-0669k	2	10396927	10397053	+	MI0006279	0	0.00%	0	0.00%	0	0.00%	0.00	0.00	0.00
mmu-mir-0467a	2	10397973	10398045	+	MI0002402	64	0.00%	96	0.00%	120	0.01%	2.47	0.46	**5.35**
mmu-mir-0466e	2	10398257	10398340	+	MI0005506	2	0.00%	0	0.00%	2	0.00%	0.00	0.00	**2.86**
mmu-mir-0669a-1	2	10398480	10398576	+	MI0004523	44	0.00%	88	0.00%	99	0.00%	3.30	0.51	**6.43**
mmu-mir-0669g	2	10398777	10398899	+	MI0006280	0	0.00%	0	0.00%	0	0.00%	0.00	0.00	0.00
mmu-mir-0669j	2	10399537	10399657	+	MI0006286	0	0.00%	0	0.00%	0	0.00%	0.00	0.00	0.00
mmu-mir-0467b	2	10402875	10402947	+	MI0004671	6	0.00%	9	0.00%	10	0.00%	2.47	0.52	**4.76**

mmu-mir-0466c	2	10403161	10403244	+	MI0005505	3	0.00%	3	0.00%	9	0.00%	1.65	0.19	**8.57**
mmu-mir-0466b-2	2	10420312	10420393	+	MI0005503	1	0.00%	0	0.00%	1	0.00%	0.00	0.00	**2.86**
mmu-mir-0466b-3	2	10425192	10425272	+	MI0005504	0	0.00%	0	0.00%	0	0.00%	0.00	0.00	0.00
mmu-mir-0467e	2	10427348	10427434	+	MI0006128	74	0.00%	101	0.00%	119	0.01%	2.25	0.49	**4.59**
mmu-mir-0467d	2	10429257	10429341	+	MI0005513	29	0.00%	41	0.00%	31	0.00%	2.33	0.76	**3.05**
mmu-mir-0466a	2	10429545	10429617	+	MI0002401	0	0.00%	0	0.00%	0	0.00%	0.00	0.00	0.00
mmu-mir-0297c	2	10430643	10430740	+	MI0005492	3	0.00%	8	0.00%	7	0.00%	4.40	0.66	**6.66**
mmu-mir-0669c	2	10430923	10431031	+	MI0004673	9480	0.16%	10842	0.31%	7987	0.39%	1.89	0.78	**2.41**
mmu-mir-0669a-2	2	10431791	10431887	+	MI0004667	4	0.00%	8	0.00%	9	0.00%	3.30	0.51	**6.43**
mmu-mir-0297b	2	10433294	10433402	+	MI0004674	2	0.00%	8	0.00%	8	0.00%	6.60	0.58	**11.42**
mmu-mir-0466d	2	10433594	10433689	+	MI0005546	5	0.00%	9	0.00%	8	0.00%	2.97	0.65	**4.57**
mmu-mir-0466g	2	10436222	10436301	+	MI0005510	1	0.00%	0	0.00%	2	0.00%	0.00	0.00	**5.71**
mmu-mir-0466h	2	10436518	10436598	+	MI0005511	0	0.00%	1	0.00%	0	0.00%	0.00	0.00	0.00
mmu-mir-0297a-3	2	10437447	10437548	+	MI0005488	2	0.00%	7	0.00%	7	0.00%	5.77	0.58	**10.00**
mmu-mir-0466l	2	10437724	10437844	+	MI0006278	0	0.00%	0	0.00%	0	0.00%	0.00	0.00	0.00
mmu-mir-0297a-4	2	10438694	10438791	+	MI0005489	2	0.00%	8	0.00%	7	0.00%	6.60	0.66	**10.00**
mmu-mir-0669i	2	10439231	10439357	+	MI0006288	0	0.00%	0	0.00%	0	0.00%	0.00	0.00	0.00
mmu-mir-0669h	2	10439782	10439906	+	MI0006289	7	0.00%	6	0.00%	6	0.00%	1.41	0.58	**2.45**
mmu-mir-0511	2	14182630	14182708	+	MI0005554	47	0.00%	65	0.00%	39	0.00%	2.28	0.96	**2.37**
mmu-mir-0001-1	2	18123753	18123829	+	MI0000139	14184	0.24%	9554	0.27%	7245	0.36%	1.11	0.76	1.46
mmu-mir-0133a-2	2	18133084	18133187	+	MI0000820	22	0.00%	16	0.00%	9	0.00%	1.20	1.03	1.17
mmu-mir-0126	2	26446877	26446949	+	MI0000153	105	0.00%	50	0.00%	58	0.00%	0.79	0.50	1.58
mmu-mir-0219-2	2	29701151	29701247	-	MI0000741	3	0.00%	3	0.00%	7	0.00%	1.65	0.25	**6.66**
mmu-mir-0199b	2	32173980	32174089	+	MI0000714	77125	1.33%	29805	0.85%	16419	0.81%	0.64	1.05	0.61
mmu-mir-0181a-2	2	38708255	38708330	+	MI0000223	8454	0.15%	3427	0.10%	1825	0.09%	0.67	1.08	0.62
mmu-mir-0181b-2	2	38709350	38709438	+	MI0000823	10037	0.17%	3469	0.10%	1711	0.08%	0.57	1.17	**0.49**
mmu-mir-0010b	2	74564127	74564194	+	MI0000221	3257	0.06%	1313	0.04%	696	0.03%	0.67	1.09	0.61
mmu-mir-0684-1	2	80468862	80468947	-	MI0004647	0	0.00%	0	0.00%	0	0.00%	0.00	0.00	0.00
mmu-mir-0130a	2	84581272	84581335	-	MI0000156	5143	0.09%	1750	0.05%	936	0.05%	0.56	1.08	0.52
mmu-mir-0129-2	2	94081521	94081610	-	MI0000585	71	0.00%	31	0.00%	61	0.00%	0.72	0.29	**2.45**
mmu-mir-0670	2	94101457	94101556	-	MI0004295	0	0.00%	0	0.00%	0	0.00%	0.00	0.00	0.00
mmu-mir-0674	2	117010863	117010962	+	MI0004611	985	0.02%	317	0.01%	205	0.01%	0.53	0.89	0.59
mmu-mir-0147	2	122466539	122466617	+	MI0005482	91	0.00%	19	0.00%	17	0.00%	0.34	0.65	0.53
mmu-mir-0103-2	2	131113788	131113873	+	MI0000588	56520	0.97%	39017	1.11%	34380	1.69%	1.14	0.66	1.74
mmu-mir-0695	2	155182553	155182661	+	MI0004675	0	0.00%	0	0.00%	0	0.00%	0.00	0.00	0.00
mmu-mir-0499	2	155448616	155448694	+	MI0004676	15	0.00%	47	0.00%	70	0.00%	5.17	0.39	**13.33**
mmu-mir-0297a-6	2	169144385	169144466	-	MI0005491	1	0.00%	0	0.00%	0	0.00%	0.00	0.00	0.00
mmu-mir-0296	2	174092548	174092626	-	MI0000394	505	0.01%	180	0.01%	146	0.01%	0.59	0.71	0.83
mmu-mir-0298	2	174093005	174093086	-	MI0000398	11505	0.20%	5252	0.15%	4538	0.22%	0.75	0.67	1.13
mmu-mir-0124-3	2	180628745	180628812	+	MI0000150	10	0.00%	7	0.00%	9	0.00%	1.15	0.45	**2.57**
mmu-mir-0009-1	3	8819520	8819608	+	MI0000720	11	0.00%	21	0.00%	24	0.00%	3.15	0.51	**6.23**
mmu-mir-0124-2	3	17695662	17695770	+	MI0000717	10	0.00%	7	0.00%	9	0.00%	1.15	0.45	**2.57**

mmu-mir-0551b	3	29315745	29315842	+	MI0004131	0	0.00%	0	0.00%	0	0.00%	0.00	0.00	0.00
mmu-mir-0015b	3	68813694	68813757	+	MI0000140	163	0.00%	138	0.00%	81	0.00%	1.40	0.98	1.42
mmu-mir-0016-2	3	68813824	68813918	+	MI0000566	427	0.01%	410	0.01%	230	0.01%	1.58	1.03	1.54
mmu-mir-0466k	3	85271299	85271420	+	MI0006292	0	0.00%	0	0.00%	0	0.00%	0.00	0.00	0.00
mmu-mir-0720	3	88920531	88920594	-	MI0004678	0	0.00%	0	0.00%	0	0.00%	0.00	0.00	0.00
mmu-mir-0092b	3	89031038	89031120	-	MI0005521	337	0.01%	298	0.01%	260	0.01%	1.46	0.66	**2.20**
mmu-mir-0190b	3	89873942	89874021	+	MI0005478	5	0.00%	3	0.00%	7	0.00%	0.99	0.25	**4.00**
mmu-mir-0137	3	118136775	118136847	+	MI0000163	0	0.00%	0	0.00%	0	0.00%	0.00	0.00	0.00
mmu-mir-0760	3	121996503	121996621	-	MI0004605	182	0.00%	685	0.02%	566	0.03%	6.21	0.70	**8.88**
mmu-mir-0302b	3	127248146	127248219	+	MI0003716	3	0.00%	0	0.00%	0	0.00%	0.00	0.00	0.00
mmu-mir-0302c	3	127248281	127248348	+	MI0003717	1	0.00%	0	0.00%	0	0.00%	0.00	0.00	0.00
mmu-mir-0302a	3	127248414	127248482	+	MI0000402	6	0.00%	0	0.00%	3	0.00%	0.00	0.00	1.43
mmu-mir-0302d	3	127248542	127248607	+	MI0003718	1	0.00%	0	0.00%	0	0.00%	0.00	0.00	0.00
mmu-mir-0367	3	127248651	127248725	+	MI0003531	1	0.00%	0	0.00%	0	0.00%	0.00	0.00	0.00
mmu-mir-0186	3	157207243	157207313	+	MI0000228	282	0.00%	149	0.00%	127	0.01%	0.87	0.68	1.29
mmu-mir-0684-2	4	11061244	11061329	+	MI0004648	0	0.00%	0	0.00%	0	0.00%	0.00	0.00	0.00
mmu-mir-0429	4	15542814	155428096	-	MI0001642	38	0.00%	57	0.00%	40	0.00%	2.47	0.82	**3.01**
mmu-mir-0876	4	36592406	36592486	-	MI0005480	0	0.00%	0	0.00%	0	0.00%	0.00	0.00	0.00
mmu-mir-0873	4	36615543	36615619	-	MI0005550	1	0.00%	0	0.00%	0	0.00%	0.00	0.00	0.00
mmu-mir-0207	4	40669950	40670028	+	MI0000250	0	0.00%	0	0.00%	0	0.00%	0.00	0.00	0.00
mmu-mir-0699	4	43505690	43505752	-	MI0004683	11	0.00%	11	0.00%	5	0.00%	1.65	1.27	1.30
mmu-mir-0032	4	56908101	56908170	-	MI0000691	68	0.00%	47	0.00%	39	0.00%	1.14	0.70	1.64
mmu-mir-0455	4	62917885	62917966	+	MI0004679	296	0.01%	118	0.00%	66	0.00%	0.66	1.03	0.64
mmu-mir-0491	4	87767944	87768029	+	MI0004680	0	0.00%	0	0.00%	0	0.00%	0.00	0.00	0.00
mmu-mir-0031	4	88556461	88556566	-	MI0000579	260	0.00%	316	0.01%	189	0.01%	2.00	0.97	**2.08**
mmu-mir-0872	4	94331848	94331928	+	MI0005549	1903	0.03%	1261	0.04%	1047	0.05%	1.09	0.70	1.57
mmu-mir-0101a	4	101019550	101019632	-	MI0000148	7468	0.13%	7365	0.21%	4625	0.23%	1.63	0.92	1.77
mmu-mir-0761	4	108690260	108690335	+	MI0004306	0	0.00%	0	0.00%	0	0.00%	0.00	0.00	0.00
mmu-mir-0030c-1	4	120442139	120442227	-	MI0000547	399	0.01%	340	0.01%	216	0.01%	1.41	0.91	1.55
mmu-mir-0030e	4	120445211	120445302	-	MI0000259	3823	0.07%	2175	0.06%	1798	0.09%	0.94	0.70	1.34
mmu-mir-0697	4	124408938	124409046	+	MI0004681	0	0.00%	0	0.00%	0	0.00%	0.00	0.00	0.00
mmu-mir-0698	4	124421025	124421133	+	MI0004682	0	0.00%	1	0.00%	0	0.00%	0.00	0.00	0.00
mmu-mir-0692-2	4	125181993	125182101	+	MI0004661	2	0.00%	4	0.00%	2	0.00%	3.30	1.16	**2.86**
mmu-mir-0700	4	134972470	134972548	-	MI0004684	36	0.00%	44	0.00%	22	0.00%	2.02	1.16	1.75
mmu-mir-0034a	4	149442563	149442664	+	MI0000584	30	0.00%	127	0.00%	70	0.00%	6.98	1.05	**6.66**
mmu-mir-0200a	4	155429005	155429094	-	MI0000554	285	0.00%	297	0.01%	247	0.01%	1.72	0.69	**2.48**
mmu-mir-0200b	4	155429790	155429859	-	MI0000243	111	0.00%	132	0.00%	95	0.00%	1.96	0.80	**2.44**
mmu-mir-0879	5	9375704	9375779	+	MI0005472	13	0.00%	10	0.00%	12	0.00%	1.27	0.48	**2.64**
mmu-mir-0671	5	24097932	24098029	+	MI0004133	13	0.00%	12	0.00%	5	0.00%	1.52	1.39	1.10
mmu-mir-0467g	5	35075502	35075621	-	MI0006301	0	0.00%	0	0.00%	0	0.00%	0.00	0.00	0.00
mmu-mir-0218-1	5	48615181	48615290	+	MI0000700	15	0.00%	6	0.00%	2	0.00%	0.66	1.73	**0.38**
mmu-mir-0574	5	65361557	65361634	+	MI0005518	69	0.00%	39	0.00%	18	0.00%	0.93	1.25	0.74

mmu-mir-1187	5	83227969	83228090	-	MI0006285	0	0.00%	0	0.00%	0	0.00%	0.00	0.00	0.00
mmu-mir-0703	5	98904575	98904683	-	MI0004687	0	0.00%	1	0.00%	0	0.00%	0.00	0.00	0.00
mmu-mir-0701	5	111433163	111433271	+	MI0004685	0	0.00%	1	0.00%	0	0.00%	0.00	0.00	0.00
mmu-mir-0469	5	113772106	113772179	+	MI0002404	0	0.00%	0	0.00%	11	0.00%	0.00	0.00	**11.00**
mmu-mir-0721	5	136851586	136851673	-	MI0004708	0	0.00%	0	0.00%	0	0.00%	0.00	0.00	0.00
mmu-mir-0702	5	137467303	137467411	+	MI0004686	17	0.00%	15	0.00%	6	0.00%	1.46	1.44	1.01
mmu-mir-0025	5	138606549	138606632	-	MI0000689	35521	0.61%	16838	0.48%	14072	0.69%	0.78	0.69	1.13
mmu-mir-0093	5	138606751	138606838	-	MI0000581	1338	0.02%	743	0.02%	445	0.02%	0.92	0.96	0.95
mmu-mir-0106b	5	138606965	138607046	-	MI0000407	936	0.02%	464	0.01%	301	0.01%	0.82	0.89	0.92
mmu-mir-0339	5	139845604	139845699	-	MI0000621	69	0.00%	75	0.00%	42	0.00%	1.79	1.03	1.74
mmu-mir-0653	6	3671301	3671385	-	MI0005557	0	0.00%	0	0.00%	0	0.00%	0.00	0.00	0.00
mmu-mir-0489	6	3671897	3672003	-	MI0003476	0	0.00%	0	0.00%	0	0.00%	0.00	0.00	0.00
mmu-mir-0592	6	27886655	27886750	-	MI0004127	0	0.00%	0	0.00%	0	0.00%	0.00	0.00	0.00
mmu-mir-0129-1	6	28972619	28972691	+	MI0000222	50	0.00%	27	0.00%	27	0.00%	0.89	0.58	1.54
mmu-mir-0182	6	30115918	30115992	-	MI0000224	143	0.00%	60	0.00%	30	0.00%	0.69	1.16	0.60
mmu-mir-0096	6	30119446	30119551	-	MI0000583	7	0.00%	4	0.00%	2	0.00%	0.94	1.16	0.82
mmu-mir-0183	6	30119668	30119737	-	MI0000225	45	0.00%	8	0.00%	19	0.00%	0.29	0.24	1.21
mmu-mir-0335	6	30691299	30691396	+	MI0000817	363	0.01%	89	0.00%	22	0.00%	0.40	2.34	**0.17**
mmu-mir-0029a	6	31012660	31012747	-	MI0000576	1696	0.03%	2697	0.08%	2578	0.13%	2.62	0.60	**4.34**
mmu-mir-0029b-1	6	31013023	31013093	-	MI0000143	28	0.00%	55	0.00%	38	0.00%	3.24	0.84	**3.88**
mmu-mir-0490	6	36371742	36371825	+	MI0005002	8	0.00%	0	0.00%	2	0.00%	0.00	0.00	0.71
mmu-mir-0704	6	47753575	47753651	-	MI0004688	5	0.00%	5	0.00%	1	0.00%	1.65	2.89	0.57
mmu-mir-0148a	6	51219811	51219909	-	MI0000550	1785	0.03%	1240	0.04%	736	0.04%	1.15	0.97	1.18
mmu-mir-0196b	6	52180080	5218164	-	MI0001151	103	0.00%	22	0.00%	26	0.00%	0.35	0.49	0.72
mmu-mir-0468	6	81846593	81846670	-	MI0002403	0	0.00%	2	0.00%	200	0.01%	0.00	0.01	**200.00**
mmu-mir-0705	6	85286286	85286367	-	MI0004689	0	0.00%	0	0.00%	0	0.00%	0.00	0.00	0.00
mmu-mir-0706	6	119984246	119984329	-	MI0004690	0	0.00%	0	0.00%	0	0.00%	0.00	0.00	0.00
mmu-mir-0141	6	124667932	124668003	-	MI0000166	3	0.00%	1	0.00%	11	0.00%	0.55	0.05	**10.47**
mmu-mir-0200c	6	124668340	124668408	-	MI0000694	47	0.00%	7	0.00%	20	0.00%	0.25	0.20	1.22
mmu-mir-0680-1	6	129641553	129641661	+	MI0004640	0	0.00%	0	0.00%	0	0.00%	0.00	0.00	0.00
mmu-mir-0220	6	136348946	136349031	-	MI0005487	0	0.00%	0	0.00%	0	0.00%	0.00	0.00	0.00
mmu-mir-0290	7	3218627	3218709	+	MI0000388	108	0.00%	21	0.00%	12	0.00%	0.32	1.01	**0.32**
mmu-mir-0291a	7	3218920	3219001	+	MI0000389	18	0.00%	8	0.00%	3	0.00%	0.73	1.54	**0.48**
mmu-mir-0292	7	3219190	3219271	+	MI0000390	34	0.00%	11	0.00%	3	0.00%	0.53	2.12	**0.25**
mmu-mir-0291b	7	3219483	3219561	+	MI0003539	0	0.00%	2	0.00%	0	0.00%	0.00	0.00	0.00
mmu-mir-0293	7	3220344	3220423	+	MI0000391	141	0.00%	55	0.00%	26	0.00%	0.64	1.22	0.53
mmu-mir-0294	7	3220642	3220725	+	MI0000392	18	0.00%	14	0.00%	7	0.00%	1.28	1.16	1.11
mmu-mir-0295	7	3220774	3220842	+	MI0000393	35	0.00%	13	0.00%	7	0.00%	0.61	1.07	0.57
mmu-mir-0297a-1	7	11543786	11543861	-	MI0000395	0	0.00%	0	0.00%	0	0.00%	0.00	0.00	0.00
mmu-mir-0330	7	19766814	19766911	+	MI0000607	217	0.00%	128	0.00%	54	0.00%	0.97	1.37	0.71
mmu-mir-0343	7	19971992	19972066	+	MI0005494	0	0.00%	1	0.00%	0	0.00%	0.00	0.00	0.00
mmu-mir-1191	7	27990555	27990674	+	MI0006296	7	0.00%	17	0.00%	6	0.00%	4.01	1.64	**2.45**

mmu-mir-0707	7	52105069	52105141	+	MI0004691	1	0.00%	2	0.00%	1	0.00%	3.30	1.16	**2.86**
mmu-mir-0150	7	52377127	52377191	+	MI0000172	22	0.00%	12	0.00%	8	0.00%	0.90	0.87	1.04
mmu-mir-0344-1	7	69022656	69022750	-	MI0000630	4	0.00%	1	0.00%	0	0.00%	0.41	0.00	0.00
mmu-mir-0344-2	7	69084913	69084992	-	MI0005495	4	0.00%	1	0.00%	0	0.00%	0.41	0.00	0.00
mmu-mir-0211	7	71350692	71350797	+	MI0000708	0	0.00%	0	0.00%	1	0.00%	0.00	0.00	1.00
mmu-mir-0007a-2	7	86033163	86033259	+	MI0000729	302	0.01%	512	0.01%	240	0.01%	2.80	1.23	**2.27**
mmu-mir-0009-3	7	86650150	86650239	+	MI0000721	11	0.00%	21	0.00%	24	0.00%	3.15	0.51	**6.23**
mmu-mir-0708	7	103397934	103398042	+	MI0004692	894	0.02%	230	0.01%	112	0.01%	0.42	1.19	**0.36**
mmu-mir-0326	7	106700779	106700873	+	MI0000598	9	0.00%	7	0.00%	10	0.00%	1.28	0.40	**3.17**
mmu-mir-0139	7	108623890	108623957	+	MI0000693	109	0.00%	101	0.00%	39	0.00%	1.53	1.50	1.02
mmu-mir-0762	7	134852001	134852076	+	MI0004215	0	0.00%	0	0.00%	0	0.00%	0.00	0.00	0.00
mmu-mir-0202	7	147143588	147143659	-	MI0000245	26735	0.46%	31010	0.88%	14525	0.71%	1.91	1.23	1.55
mmu-mir-0210	7	148407283	148407392	-	MI0000695	881	0.02%	399	0.01%	159	0.01%	0.75	1.45	0.52
mmu-mir-0675	7	149762969	149763052	-	MI0004123	3	0.00%	3	0.00%	0	0.00%	1.65	0.00	0.00
mmu-mir-0483	7	149840829	149840901	-	MI0003484	664	0.01%	121	0.00%	68	0.00%	0.30	1.03	**0.29**
mmu-mir-0486	8	24253027	24253154	+	MI0003493	193	0.00%	95	0.00%	32	0.00%	0.81	1.71	**0.47**
mmu-mir-1186	8	32213393	32213514	-	MI0006284	0	0.00%	0	0.00%	0	0.00%	0.00	0.00	0.00
mmu-mir-0383	8	39315187	39315256	-	MI0000800	64	0.00%	9	0.00%	3	0.00%	0.23	1.73	**0.13**
mmu-mir-0710	8	66993129	66993238	-	MI0004694	0	0.00%	0	0.00%	0	0.00%	0.00	0.00	0.00
mmu-mir-1199	8	86535414	86535532	-	MI0006307	1	0.00%	0	0.00%	0	0.00%	0.00	0.00	0.00
mmu-mir-0709	8	86609998	86610085	+	MI0004693	0	0.00%	0	0.00%	0	0.00%	0.00	0.00	0.00
mmu-mir-0181d	8	86702615	86702686	-	MI0005450	23303	0.40%	6628	0.19%	4199	0.21%	0.47	0.91	0.51
mmu-mir-0181c	8	86702772	86702860	-	MI0000724	922	0.02%	359	0.01%	152	0.01%	0.64	1.36	**0.47**
mmu-mir-0023a	8	86732417	86732491	+	MI0000571	812	0.01%	369	0.01%	349	0.02%	0.75	0.61	1.23
mmu-mir-0027a	8	86732571	86732657	+	MI0000578	250	0.00%	180	0.01%	81	0.00%	1.19	1.28	0.93
mmu-mir-0024-2	8	86732714	86732820	+	MI0000572	1266	0.02%	919	0.03%	400	0.02%	1.20	1.33	0.90
mmu-mir-0138-2	8	96848211	96848281	+	MI0000164	35	0.00%	13	0.00%	12	0.00%	0.61	0.63	0.98
mmu-mir-0328	8	107832264	107832360	-	MI0000603	143	0.00%	101	0.00%	63	0.00%	1.17	0.93	1.26
mmu-mir-0140	8	110075144	110075213	+	MI0000165	83975	1.45%	45700	1.30%	23078	1.13%	0.90	1.14	0.78
mmu-mir-0199a-1	9	21300939	21301008	-	MI0000241	77532	1.33%	29943	0.85%	16495	0.81%	0.64	1.05	0.61
mmu-mir-0100	9	41339508	41339587	+	MI0000692	622	0.01%	562	0.02%	451	0.02%	1.49	0.72	**2.07**
mmu-let-7a-2	9	41344799	41344894	+	MI0000557	333447	5.74%	185126	5.26%	98376	4.83%	0.92	1.09	0.84
mmu-mir-0125b-1	9	41390009	41390085	+	MI0000725	3404	0.06%	2546	0.07%	1279	0.06%	1.23	1.15	1.07
mmu-mir-0034c	9	50911139	50911215	-	MI0000403	2719	0.05%	2070	0.06%	27087	1.33%	1.26	0.04	**28.45**
mmu-mir-0034b	9	50911667	50911750	-	MI0000404	63	0.00%	49	0.00%	660	0.03%	1.28	0.04	**29.92**
mmu-mir-0190	9	67084467	67084533	-	MI0000232	1	0.00%	0	0.00%	1	0.00%	0.00	0.00	**2.86**
mmu-mir-0184	9	89697098	89697166	-	MI0000226	122	0.00%	70	0.00%	78	0.00%	0.95	0.52	1.83
mmu-mir-0135a-1	9	106056455	106056544	+	MI0000161	15	0.00%	7	0.00%	6	0.00%	0.77	0.67	1.14
mmu-let-7g	9	106081171	106081258	+	MI0000137	110416	1.90%	87323	2.48%	49601	2.44%	1.30	1.02	1.28
mmu-mir-0191	9	108470650	108470723	+	MI0000233	9956	0.17%	5514	0.16%	9210	0.45%	0.91	0.35	**2.64**
mmu-mir-0425	9	108471108	108471192	+	MI0001447	87	0.00%	63	0.00%	101	0.00%	1.19	0.36	**3.32**
mmu-mir-0711	9	108871967	108872048	+	MI0004695	0	0.00%	0	0.00%	0	0.00%	0.00	0.00	0.00

mmu-mir-0128-2	9	112021140	112021215	-	MI0000726	498	0.01%	469	0.01%	260	0.01%	1.55	1.04	1.49
mmu-mir-0467h	9	115290937	115291057	+	MI0006302	0	0.00%	0	0.00%	0	0.00%	0.00	0.00	0.00
mmu-mir-0026a-1	9	118940914	118941003	+	MI0000573	6209	0.11%	4204	0.12%	2222	0.11%	1.12	1.09	1.02
mmu-mir-0138-1	9	122591994	122592092	+	MI0000722	44	0.00%	17	0.00%	12	0.00%	0.64	0.82	0.78
mmu-mir-0297a-2	10	42855276	42855339	+	MI0000397	1	0.00%	0	0.00%	0	0.00%	0.00	0.00	0.00
mmu-mir-0466j	10	60423471	60423592	+	MI0006295	0	0.00%	0	0.00%	0	0.00%	0.00	0.00	0.00
mmu-mir-0678	10	75670071	75670154	-	MI0004635	0	0.00%	0	0.00%	0	0.00%	0.00	0.00	0.00
mmu-mir-0135a-2	10	91534831	91534930	-	MI0000715	130	0.00%	35	0.00%	19	0.00%	0.44	1.06	**0.42**
mmu-mir-0331	10	93426513	93426608	-	MI0000609	181	0.00%	138	0.00%	91	0.00%	1.26	0.88	1.44
mmu-mir-0763	10	119885047	119885166	-	MI0004516	0	0.00%	0	0.00%	0	0.00%	0.00	0.00	0.00
mmu-let-7i	10	122422696	122422780	-	MI0000138	55685	0.96%	46896	1.33%	25136	1.24%	1.39	1.08	1.29
mmu-mir-0026a-2	10	126432586	126432669	+	MI0000706	6270	0.11%	4264	0.12%	2249	0.11%	1.12	1.10	1.02
mmu-mir-0546	10	126435496	126435616	+	MI0003517	68	0.00%	136	0.00%	42	0.00%	3.30	1.87	1.76
mmu-mir-0677	10	127522342	127522450	+	MI0004634	10	0.00%	4	0.00%	6	0.00%	0.66	0.39	1.71
mmu-mir-0216b	11	28646191	28646276	+	MI0004126	1	0.00%	1	0.00%	0	0.00%	1.65	0.00	0.00
mmu-mir-0216a	11	28657012	28657083	+	MI0000699	0	0.00%	0	0.00%	0	0.00%	0.00	0.00	0.00
mmu-mir-0217	11	28663728	28663835	+	MI0000731	14	0.00%	4	0.00%	4	0.00%	0.47	0.58	0.82
mmu-mir-0218-2	11	35430318	35430427	+	MI0000701	37	0.00%	22	0.00%	4	0.00%	0.98	3.18	**0.31**
mmu-mir-0103-1	11	35595898	35595983	+	MI0000587	54315	0.93%	38102	1.08%	32979	1.62%	1.16	0.67	1.73
mmu-mir-0146a	11	43187899	43187963	-	MI0000170	7	0.00%	62	0.00%	30	0.00%	14.61	1.19	**12.24**
mmu-mir-0340	11	49883204	49883301	+	MI0000623	6729	0.12%	4479	0.13%	3668	0.18%	1.10	0.71	1.56
mmu-mir-0804	11	50171287	50171381	-	MI0005203	0	0.00%	0	0.00%	0	0.00%	0.00	0.00	0.00
mmu-mir-0744	11	65548235	65548334	-	MI0004124	7165	0.12%	6289	0.18%	2512	0.12%	1.45	1.45	1.00
mmu-mir-0467f	11	69448902	69449021	-	MI0006293	0	0.00%	0	0.00%	0	0.00%	0.00	0.00	0.00
mmu-mir-0324	11	69825545	69825633	+	MI0000595	103	0.00%	68	0.00%	50	0.00%	1.09	0.79	1.39
mmu-mir-0497	11	70048219	70048302	+	MI0004636	340	0.01%	257	0.01%	179	0.01%	1.25	0.83	1.50
mmu-mir-0195	11	70048544	70048637	+	MI0000237	104	0.00%	110	0.00%	73	0.00%	1.74	0.87	**2.00**
mmu-mir-0212	11	74986890	74986980	+	MI0000696	30	0.00%	23	0.00%	36	0.00%	1.26	0.37	**3.43**
mmu-mir-0132	11	74987184	74987249	+	MI0000158	23	0.00%	20	0.00%	16	0.00%	1.43	0.72	1.99
mmu-mir-0022	11	75277218	75277312	+	MI0000570	1380	0.02%	1082	0.03%	686	0.03%	1.29	0.91	1.42
mmu-mir-0423	11	76891566	76891674	-	MI0004637	35922	0.62%	22220	0.63%	15030	0.74%	1.02	0.85	1.19
mmu-mir-0144	11	77886507	77886572	+	MI0000168	73	0.00%	16	0.00%	16	0.00%	0.36	0.58	0.63
mmu-mir-0451	11	77886672	77886743	+	MI0001730	396	0.01%	103	0.00%	155	0.01%	0.43	0.38	1.12
mmu-mir-0193	11	79525471	79525536	+	MI0000235	137	0.00%	88	0.00%	53	0.00%	1.06	0.96	1.10
mmu-mir-0365-2	11	79539902	79540013	+	MI0001645	864	0.01%	306	0.01%	200	0.01%	0.58	0.88	0.66
mmu-mir-0021	11	86397569	86397660	-	MI0000569	6717	0.12%	4991	0.14%	3200	0.16%	1.23	0.90	1.36
mmu-mir-0301a	11	86926506	86926591	+	MI0000401	183	0.00%	82	0.00%	68	0.00%	0.74	0.70	1.06
mmu-mir-0142	11	87570366	87570429	+	MI0000167	112	0.00%	65	0.00%	77	0.00%	0.96	0.49	1.96
mmu-mir-0196a-1	11	96126478	96126579	+	MI0000552	451	0.01%	214	0.01%	87	0.00%	0.78	1.42	0.55
mmu-mir-0010a	11	96178479	96178588	+	MI0000685	4601	0.08%	821	0.02%	804	0.04%	0.29	0.59	**0.50**
mmu-mir-0152	11	96711707	96711779	+	MI0000174	25474	0.44%	14709	0.42%	6900	0.34%	0.95	1.23	0.77
mmu-mir-0338	11	119876079	119876176	-	MI0000619	3	0.00%	5	0.00%	1	0.00%	2.75	2.89	0.95

mmu-mir-0770	12	11081902	11081995	+	MI0004203	68	0.00%	23	0.00%	3	0.00%	0.56	4.43	**0.13**
mmu-mir-0680-3	12	35879483	35879569	-	MI0004642	0	0.00%	0	0.00%	0	0.00%	0.00	0.00	0.00
mmu-mir-0681	12	70864822	70864931	-	MI0004643	0	0.00%	0	0.00%	0	0.00%	0.00	0.00	0.00
mmu-mir-1190	12	102259883	102260003	-	MI0006294	1	0.00%	0	0.00%	0	0.00%	0.00	0.00	0.00
mmu-mir-0342	12	109896830	109896928	+	MI0000627	1035	0.02%	1322	0.04%	796	0.04%	2.11	0.96	**2.20**
mmu-mir-0345	12	110075183	110075278	+	MI0000632	1050	0.02%	367	0.01%	259	0.01%	0.58	0.82	0.70
mmu-mir-0673	12	110810200	110810290	+	MI0004601	66	0.00%	20	0.00%	14	0.00%	0.50	0.83	0.61
mmu-mir-0493	12	110818443	110818525	+	MI0005514	14	0.00%	12	0.00%	3	0.00%	1.41	2.31	0.61
mmu-mir-0337	12	110823999	110824095	+	MI0000615	420	0.01%	162	0.00%	53	0.00%	0.64	1.77	**0.36**
mmu-mir-0540	12	110824290	110824356	+	MI0003518	236	0.00%	116	0.00%	37	0.00%	0.81	1.81	**0.45**
mmu-mir-0665	12	110824524	110824617	+	MI0004171	160	0.00%	44	0.00%	11	0.00%	0.45	2.31	**0.20**
mmu-mir-0431	12	110828657	110828747	+	MI0001524	28	0.00%	12	0.00%	5	0.00%	0.71	1.39	0.51
mmu-mir-0433	12	110829925	110830048	+	MI0001525	2907	0.05%	768	0.02%	287	0.01%	0.44	1.55	**0.28**
mmu-mir-0127	12	110831056	110831125	+	MI0000154	28043	0.48%	10284	0.29%	3166	0.16%	0.60	1.88	**0.32**
mmu-mir-0434	12	110832716	110832809	+	MI0001526	1138	0.02%	493	0.01%	298	0.01%	0.71	0.96	0.75
mmu-mir-0136	12	110833537	110833598	+	MI0000162	442	0.01%	152	0.00%	47	0.00%	0.57	1.87	**0.30**
mmu-mir-0341	12	110849710	110849805	+	MI0000625	2420	0.04%	931	0.03%	211	0.01%	0.63	2.55	**0.25**
mmu-mir-1188	12	110850032	110850151	+	MI0006290	11	0.00%	1	0.00%	0	0.00%	0.15	0.00	0.00
mmu-mir-0370	12	110856468	110856546	+	MI0001165	169	0.00%	29	0.00%	4	0.00%	0.28	4.19	**0.07**
mmu-mir-0882	12	110920407	110920483	+	MI0005475	0	0.00%	0	0.00%	0	0.00%	0.00	0.00	0.00
mmu-mir-0379	12	110947270	110947335	+	MI0000796	4507	0.08%	1797	0.05%	740	0.04%	0.66	1.40	**0.47**
mmu-mir-0411	12	110948385	110948466	+	MI0001163	1397	0.02%	507	0.01%	197	0.01%	0.60	1.49	**0.40**
mmu-mir-0299	12	110948848	110948910	+	MI0000399	325	0.01%	116	0.00%	47	0.00%	0.59	1.43	**0.41**
mmu-mir-0380	12	110950013	110950073	+	MI0000797	98	0.00%	37	0.00%	14	0.00%	0.62	1.53	**0.41**
mmu-mir-1197	12	110950527	110950646	+	MI0006305	1	0.00%	0	0.00%	0	0.00%	0.00	0.00	0.00
mmu-mir-0323	12	110950718	110950803	+	MI0000592	447	0.01%	104	0.00%	37	0.00%	0.38	1.62	**0.24**
mmu-mir-0758	12	110951020	110951100	+	MI0004129	3	0.00%	0	0.00%	0	0.00%	0.00	0.00	0.00
mmu-mir-0329	12	110951691	110951787	+	MI0000605	692	0.01%	178	0.01%	96	0.00%	0.42	1.07	**0.40**
mmu-mir-0494	12	110953528	110953612	+	MI0003532	174	0.00%	48	0.00%	24	0.00%	0.46	1.16	**0.39**
mmu-mir-0679	12	110953787	110953860	+	MI0004638	3	0.00%	1	0.00%	1	0.00%	0.55	0.58	0.95
mmu-mir-1193	12	110953881	110954001	+	MI0006298	53	0.00%	19	0.00%	9	0.00%	0.59	1.22	**0.48**
mmu-mir-0666	12	110955295	110955393	+	MI0004553	60	0.00%	20	0.00%	7	0.00%	0.55	1.65	**0.33**
mmu-mir-0543	12	110955468	110955543	+	MI0003519	1367	0.02%	288	0.01%	91	0.00%	0.35	1.83	**0.19**
mmu-mir-0495	12	110956964	110957026	+	MI0004639	437	0.01%	116	0.00%	51	0.00%	0.44	1.31	**0.33**
mmu-mir-0667	12	110958216	110958307	+	MI0004196	18	0.00%	12	0.00%	5	0.00%	1.10	1.39	0.79
mmu-mir-0376c	12	110960928	110961013	+	MI0003533	88	0.00%	33	0.00%	9	0.00%	0.62	2.12	**0.29**
mmu-mir-0654	12	110961428	110961511	+	MI0005520	0	0.00%	0	0.00%	0	0.00%	0.00	0.00	0.00
mmu-mir-0376b	12	110961668	110961749	+	MI0001162	187	0.00%	28	0.00%	14	0.00%	0.25	1.16	**0.21**
mmu-mir-0376a	12	110961991	110962058	+	MI0000793	1326	0.02%	335	0.01%	104	0.01%	0.42	1.86	**0.22**
mmu-mir-0300	12	110962523	110962601	+	MI0000400	102	0.00%	26	0.00%	12	0.00%	0.42	1.25	**0.34**
mmu-mir-0381	12	110965032	110965106	+	MI0000798	47	0.00%	15	0.00%	5	0.00%	0.53	1.73	**0.30**
mmu-mir-0487b	12	110965543	110965624	+	MI0003534	73	0.00%	38	0.00%	9	0.00%	0.86	2.44	**0.35**

mmu-mir-0539	12	110966339	110966412	+	MI0003520	16	0.00%	3	0.00%	2	0.00%	0.31	0.87	**0.36**
mmu-mir-0544	12	110967535	110967612	+	MI0005555	2	0.00%	0	0.00%	0	0.00%	0.00	0.00	0.00
mmu-mir-0382	12	110971981	110972056	+	MI0000799	3977	0.07%	1251	0.04%	597	0.03%	0.52	1.21	**0.43**
mmu-mir-0134	12	110972349	110972419	+	MI0000160	759	0.01%	210	0.01%	67	0.00%	0.46	1.81	**0.25**
mmu-mir-0668	12	110972942	110973007	+	MI0004134	192	0.00%	86	0.00%	16	0.00%	0.74	3.10	**0.24**
mmu-mir-0485	12	110973112	110973184	+	MI0003492	3759	0.06%	862	0.02%	372	0.02%	0.38	1.34	**0.28**
mmu-mir-0453	12	110973829	110973910	+	MI0005497	0	0.00%	0	0.00%	0	0.00%	0.00	0.00	0.00
mmu-mir-0154	12	110976643	110976708	+	MI0000176	195	0.00%	72	0.00%	33	0.00%	0.61	1.26	**0.48**
mmu-mir-0496	12	110977329	110977407	+	MI0004589	96	0.00%	37	0.00%	15	0.00%	0.64	1.42	**0.45**
mmu-mir-0377	12	110978720	110978787	+	MI0000794	41	0.00%	9	0.00%	4	0.00%	0.36	1.30	**0.28**
mmu-mir-0541	12	110980619	110980708	+	MI0003521	5538	0.10%	1540	0.04%	718	0.04%	0.46	1.24	**0.37**
mmu-mir-0409	12	110981368	110981446	+	MI0001160	567	0.01%	161	0.00%	61	0.00%	0.47	1.52	**0.31**
mmu-mir-0412	12	110981499	110981578	+	MI0001164	15	0.00%	1	0.00%	0	0.00%	0.11	0.00	0.00
mmu-mir-0369	12	110981628	110981706	+	MI0003535	218	0.00%	64	0.00%	18	0.00%	0.48	2.05	**0.24**
mmu-mir-0410	12	110981925	110982005	+	MI0001161	249	0.00%	56	0.00%	12	0.00%	0.37	2.70	**0.14**
mmu-mir-0203	12	113369091	113369166	+	MI0000246	517	0.01%	663	0.02%	553	0.03%	2.12	0.69	**3.05**
mmu-mir-0153	12	118489290	118489358	+	MI0000175	3	0.00%	2	0.00%	1	0.00%	1.10	1.16	0.95
mmu-mir-0466i	13	17839307	17839427	+	MI0006282	0	0.00%	0	0.00%	0	0.00%	0.00	0.00	0.00
mmu-let-7d	13	48631381	48631483	-	MI0000405	121665	2.09%	60122	1.71%	35763	1.76%	0.82	0.97	0.84
mmu-let-7f-1	13	48633198	48633286	-	MI0000562	607040	10.45%	352136	10.00%	225358	11.08%	0.96	0.90	1.06
mmu-let-7a-1	13	48633548	48633641	-	MI0000556	356499	6.14%	193614	5.50%	103864	5.10%	0.90	1.08	0.83
mmu-mir-0683	13	50639995	50640103	-	MI0004646	0	0.00%	0	0.00%	0	0.00%	0.00	0.00	0.00
mmu-mir-0874	13	58124486	58124561	-	MI0005479	3	0.00%	1	0.00%	1	0.00%	0.55	0.58	0.95
mmu-mir-0007a-1	13	58494140	58494247	-	MI0000728	353	0.01%	596	0.02%	292	0.01%	2.79	1.18	**2.36**
mmu-mir-0713	13	62862099	62862206	+	MI0004698	0	0.00%	0	0.00%	0	0.00%	0.00	0.00	0.00
mmu-mir-0027b	13	63402020	63402092	+	MI0000142	1121	0.02%	671	0.02%	275	0.01%	0.99	1.41	0.70
mmu-mir-0024-1	13	63402516	63402583	+	MI0000231	1255	0.02%	919	0.03%	403	0.02%	1.21	1.32	0.92
mmu-mir-0466f-4	13	71245967	71246087	+	MI0006291	0	0.00%	0	0.00%	1	0.00%	0.00	0.00	1.00
mmu-mir-0682	13	75782494	75782589	+	MI0004644	1	0.00%	6	0.00%	2	0.00%	9.90	1.73	**5.71**
mmu-mir-0009-2	13	83878419	83878490	+	MI0000157	11	0.00%	20	0.00%	24	0.00%	3.00	0.48	**6.23**
mmu-mir-0582	13	110114938	110115018	+	MI0006127	7	0.00%	3	0.00%	4	0.00%	0.71	0.43	1.63
mmu-mir-0449c	13	113826191	113826299	+	MI0004645	64	0.00%	43	0.00%	912	0.04%	1.11	0.03	**40.70**
mmu-mir-0449b	13	113827627	113827706	+	MI0005547	0	0.00%	0	0.00%	3	0.00%	0.00	0.00	3.00
mmu-mir-0449a	13	113827742	113827832	+	MI0001649	41	0.00%	27	0.00%	495	0.02%	1.09	0.03	**34.48**
mmu-mir-0023b	13	63401791	63401865	+	MI0000141	1433	0.02%	644	0.02%	351	0.02%	0.74	1.06	0.70
mmu-mir-0759	14	8138238	8138335	+	MI0004554	0	0.00%	0	0.00%	0	0.00%	0.00	0.00	0.00
mmu-mir-0346	14	35707795	35707892	+	MI0000634	1	0.00%	0	0.00%	0	0.00%	0.00	0.00	0.00
mmu-mir-0327	14	45567118	45567186	-	MI0005493	0	0.00%	0	0.00%	0	0.00%	0.00	0.00	0.00
mmu-mir-0685	14	51427159	51427267	-	MI0004649	36	0.00%	64	0.00%	29	0.00%	2.93	1.27	**2.30**
mmu-mir-0686	14	55235514	55235622	-	MI0004650	6	0.00%	4	0.00%	1	0.00%	1.10	2.31	**0.48**
mmu-mir-0208a	14	55567897	55567979	-	MI0000555	0	0.00%	0	0.00%	0	0.00%	0.00	0.00	0.00
mmu-mir-0208b	14	55594537	55594613	-	MI0005552	1	0.00%	0	0.00%	0	0.00%	0.00	0.00	0.00

mmu-mir-0719	14	60847643	60847752	-	MI0004651	0	0.00%	0	0.00%	0	0.00%	0.00	0.00	0.00
mmu-mir-0016-1	14	62250717	62250809	-	MI0000565	593	0.01%	531	0.02%	292	0.01%	1.48	1.05	1.41
mmu-mir-0015a	14	62250864	62250947	-	MI0000564	175	0.00%	157	0.00%	106	0.01%	1.48	0.86	1.73
mmu-mir-1196	14	62371057	62371175	-	MI0006304	0	0.00%	0	0.00%	0	0.00%	0.00	0.00	0.00
mmu-mir-0598	14	64346026	64346104	+	MI0005556	616	0.01%	574	0.02%	575	0.03%	1.54	0.58	**2.67**
mmu-mir-0124-1	14	65209494	65209578	+	MI0000716	10	0.00%	7	0.00%	9	0.00%	1.15	0.45	**2.57**
mmu-mir-0320	14	70843317	70843398	+	MI0000704	48550	0.84%	20700	0.59%	10643	0.52%	0.70	1.12	0.63
mmu-mir-0687	14	73606570	73606658	-	MI0004652	0	0.00%	0	0.00%	0	0.00%	0.00	0.00	0.00
mmu-mir-0017	14	115442893	115442976	+	MI0000687	1045	0.02%	639	0.02%	423	0.02%	1.01	0.87	1.16
mmu-mir-0018a	14	115443073	115443168	+	MI0000567	34	0.00%	23	0.00%	15	0.00%	1.12	0.89	1.26
mmu-mir-0019a	14	115443222	115443303	+	MI0000688	7	0.00%	5	0.00%	3	0.00%	1.18	0.96	1.22
mmu-mir-0020a	14	115443379	115443485	+	MI0000568	85	0.00%	57	0.00%	53	0.00%	1.11	0.62	1.78
mmu-mir-0019b-1	14	115443527	115443613	+	MI0000718	30	0.00%	21	0.00%	14	0.00%	1.15	0.87	1.33
mmu-mir-0092a-1	14	115443649	115443728	+	MI0000719	2326	0.04%	1584	0.04%	1102	0.05%	1.12	0.83	1.35
mmu-mir-0875	15	35590725	35590802	-	MI0005551	0	0.00%	0	0.00%	0	0.00%	0.00	0.00	0.00
mmu-mir-0030b	15	68168977	68169072	-	MI0000145	240	0.00%	223	0.01%	154	0.01%	1.53	0.84	1.83
mmu-mir-0030d	15	68172770	68172851	-	MI0000549	7120	0.12%	5031	0.14%	3307	0.16%	1.17	0.88	1.33
mmu-mir-0151	15	73085245	73085312	-	MI0000173	2413	0.04%	1317	0.04%	1013	0.05%	0.90	0.75	1.20
mmu-mir-0464	15	74476179	74476249	+	MI0002399	0	0.00%	1	0.00%	37	0.00%	0.00	0.02	**37.00**
mmu-mir-0033	15	82028552	82028620	+	MI0000707	893	0.02%	586	0.02%	220	0.01%	1.08	1.54	0.70
mmu-let-7c-2	15	85537033	85537127	+	MI0000560	792575	13.64%	527623	14.98%	275784	13.55%	1.10	1.11	0.99
mmu-let-7b	15	85537749	85537833	+	MI0000558	550172	9.47%	411028	11.67%	177097	8.70%	1.23	1.34	0.92
mmu-mir-0688	15	102502223	102502297	-	MI0004653	1	0.00%	1	0.00%	0	0.00%	1.65	0.00	0.00
mmu-mir-0196a-2	15	102803781	102803865	+	MI0000553	417	0.01%	219	0.01%	97	0.00%	0.87	1.30	0.66
mmu-mir-0615	15	102845341	102845432	+	MI0005004	64	0.00%	19	0.00%	4	0.00%	0.49	2.74	**0.18**
mmu-mir-0148b	15	103115556	103115652	+	MI0000617	373	0.01%	325	0.01%	189	0.01%	1.44	0.99	1.45
mmu-mir-0689-2	16	11144133	11144241	+	MI0004655	36	0.00%	31	0.00%	5	0.00%	1.42	3.58	**0.40**
mmu-mir-0193b	16	13449616	13449694	+	MI0005484	669	0.01%	637	0.02%	440	0.02%	1.57	0.84	1.88
mmu-mir-0365-1	16	13453933	13454019	+	MI0000768	402	0.01%	249	0.01%	267	0.01%	1.02	0.54	1.90
mmu-mir-0484	16	14159719	14159785	+	MI0003491	55	0.00%	53	0.00%	17	0.00%	1.59	1.80	0.88
mmu-mir-0130b	16	17124154	17124235	-	MI0000408	187	0.00%	97	0.00%	81	0.00%	0.86	0.69	1.24
mmu-mir-0301b	16	17124493	17124589	-	MI0004122	4	0.00%	1	0.00%	2	0.00%	0.41	0.29	1.43
mmu-mir-0185	16	18327494	18327558	-	MI0000227	6492	0.11%	5039	0.14%	2604	0.13%	1.28	1.12	1.15
mmu-mir-1224	16	20604525	20604609	+	MI0004118	99	0.00%	27	0.00%	11	0.00%	0.45	1.42	**0.32**
mmu-mir-0028	16	24827941	24828026	+	MI0000690	401	0.01%	171	0.00%	114	0.01%	0.70	0.87	0.81
mmu-mir-0690	16	28600021	28600129	-	MI0004658	0	0.00%	0	0.00%	0	0.00%	0.00	0.00	0.00
mmu-mir-0568	16	43640768	43640850	+	MI0005517	0	0.00%	0	0.00%	0	0.00%	0.00	0.00	0.00
mmu-mir-0691	16	74342235	74342312	-	MI0004659	0	0.00%	0	0.00%	0	0.00%	0.00	0.00	0.00
mmu-mir-0099a	16	77599181	77599245	+	MI0000146	12049	0.21%	6290	0.18%	3769	0.19%	0.86	0.96	0.89
mmu-let-7c-1	16	77599902	77599995	+	MI0000559	699782	12.04%	492897	13.99%	241359	11.86%	1.16	1.18	0.98
mmu-mir-0125b-2	16	77646518	77646588	+	MI0000152	2975	0.05%	2227	0.06%	1047	0.05%	1.23	1.23	1.01
mmu-mir-0155	16	84714385	84714449	+	MI0000177	1	0.00%	0	0.00%	1	0.00%	0.00	0.00	**2.86**

mmu-mir-0802	16	93369965	93370061	+	MI0004249	0	0.00%	0	0.00%	0	0.00%	0.00	0.00	0.00
mmu-mir-0692-1	17	7099578	7099686	-	MI0004660	0	0.00%	0	0.00%	0	0.00%	0.00	0.00	0.00
mmu-mir-0099b	17	17967152	17967221	+	MI0000147	14108	0.24%	5130	0.15%	2751	0.14%	0.60	1.08	0.56
mmu-let-7e	17	17967316	17967408	+	MI0000561	308164	5.30%	97531	2.77%	45991	2.26%	0.52	1.22	**0.43**
mmu-mir-0125a	17	17967776	17967843	+	MI0000151	4177	0.07%	2282	0.06%	806	0.04%	0.90	1.64	0.55
mmu-mir-0219-1	17	34161928	34162037	-	MI0000702	123	0.00%	73	0.00%	53	0.00%	0.98	0.80	1.23
mmu-mir-0877	17	36097675	36097759	-	MI0005553	498	0.01%	353	0.01%	302	0.01%	1.17	0.68	1.73
mmu-mir-0715	17	39981081	39981190	+	MI0004700	19	0.00%	8	0.00%	4	0.00%	0.69	1.16	0.60
mmu-mir-0693	17	46368480	46368568	+	MI0004662	0	0.00%	0	0.00%	0	0.00%	0.00	0.00	0.00
mmu-mir-0007b	17	56382411	56382521	+	MI0000730	7	0.00%	3	0.00%	2	0.00%	0.71	0.87	0.82
mmu-mir-1195	17	71209818	71209940	-	MI0006303	14	0.00%	54	0.00%	21	0.00%	6.36	1.49	**4.28**
mmu-mir-0133a-1	18	10782907	10782974	-	MI0000159	22	0.00%	16	0.00%	12	0.00%	1.20	0.77	1.56
mmu-mir-0001-2-as	18	10785444	10785565	+	MI0006283	14204	0.24%	9554	0.27%	7240	0.36%	1.11	0.76	1.46
mmu-mir-0001-2	18	10785479	10785550	-	MI0000652	14204	0.24%	9554	0.27%	7240	0.36%	1.11	0.76	1.46
mmu-mir-0187	18	24587611	24587671	-	MI0000229	105	0.00%	61	0.00%	27	0.00%	0.96	1.31	0.73
mmu-mir-0378	18	61557489	61557554	-	MI0000795	2317	0.04%	654	0.02%	594	0.03%	0.47	0.64	0.73
mmu-mir-0145	18	61807479	61807548	-	MI0000169	1124	0.02%	791	0.02%	837	0.04%	1.16	0.55	**2.13**
mmu-mir-0143	18	61808850	61808912	-	MI0000257	11430	0.20%	9521	0.27%	6542	0.32%	1.37	0.84	1.63
mmu-mir-0122	18	65408515	65408580	+	MI0000256	739	0.01%	25	0.00%	6	0.00%	0.06	2.41	**0.02**
mmu-mir-0694	18	66378918	66378987	-	MI0004664	0	0.00%	1	0.00%	1	0.00%	0.00	0.58	1.00
mmu-mir-0194-2	19	6264643	6264728	+	MI0000733	20	0.00%	18	0.00%	29	0.00%	1.48	0.36	**4.14**
mmu-mir-0192	19	6264844	6264932	+	MI0000551	2983	0.05%	2233	0.06%	3390	0.17%	1.23	0.38	**3.25**
mmu-mir-0204	19	22825095	22825162	+	MI0000247	96	0.00%	377	0.01%	385	0.02%	6.48	0.57	**11.45**
mmu-mir-1192	19	23223921	23224041	+	MI0006297	0	0.00%	0	0.00%	0	0.00%	0.00	0.00	0.00
mmu-mir-0101b	19	29209769	29209865	+	MI0000649	4780	0.08%	4228	0.12%	3090	0.15%	1.46	0.79	1.85
mmu-mir-0107	19	34895177	34895263	-	MI0000684	39177	0.67%	26185	0.74%	17080	0.84%	1.10	0.89	1.25
mmu-mir-0146b	19	46417252	46417360	+	MI0004665	288	0.00%	228	0.01%	297	0.01%	1.31	0.44	**2.95**
mmu-mir-0500	X	6814809	6814900	-	MI0004702	43	0.00%	44	0.00%	29	0.00%	1.69	0.88	1.93
mmu-mir-0501	X	6818369	6818477	-	MI0004703	208	0.00%	118	0.00%	80	0.00%	0.94	0.85	1.10
mmu-mir-0362	X	6819108	6819172	-	MI0000763	151	0.00%	117	0.00%	101	0.00%	1.28	0.67	1.91
mmu-mir-0188	X	6825115	6825182	-	MI0000230	39	0.00%	20	0.00%	12	0.00%	0.85	0.96	0.88
mmu-mir-0532	X	6825528	6825623	-	MI0003206	779	0.01%	370	0.01%	366	0.02%	0.78	0.58	1.34
mmu-mir-1198	X	7384227	7384347	+	MI0006306	25	0.00%	12	0.00%	8	0.00%	0.79	0.87	0.91
mmu-mir-0221	X	18723420	18723514	-	MI0000709	2095	0.04%	580	0.02%	496	0.02%	0.46	0.68	0.68
mmu-mir-0222	X	18724019	18724097	-	MI0000710	365	0.01%	125	0.00%	71	0.00%	0.56	1.02	0.56
mmu-mir-0717	X	49775584	49775692	-	MI0004704	0	0.00%	0	0.00%	0	0.00%	0.00	0.00	0.00
mmu-mir-0363	X	50094870	50094944	-	MI0000765	73	0.00%	34	0.00%	31	0.00%	0.77	0.63	1.21
mmu-mir-0092a-2	X	50095015	50095105	-	MI0000580	130	0.00%	75	0.00%	49	0.00%	0.95	0.88	1.08
mmu-mir-0019b-2	X	50095160	50095243	-	MI0000546	28	0.00%	21	0.00%	15	0.00%	1.24	0.81	1.53
mmu-mir-0020b	X	50095290	50095369	-	MI0003536	1	0.00%	1	0.00%	2	0.00%	1.65	0.29	**5.71**
mmu-mir-0018b	X	50095508	50095590	-	MI0005483	0	0.00%	0	0.00%	1	0.00%	0.00	0.00	1.00
mmu-mir-0106a	X	50095680	50095744	-	MI0000406	2	0.00%	0	0.00%	1	0.00%	0.00	0.00	1.43

mmu-mir-0450b	X	50401174	50401255	-	MI0004705	266	0.00%	125	0.00%	51	0.00%	0.78	1.42	0.55
mmu-mir-0450a-1	X	50401331	50401421	-	MI0001653	111	0.00%	80	0.00%	29	0.00%	1.19	1.59	0.75
mmu-mir-0450a-2	X	50401476	50401544	-	MI0003537	111	0.00%	77	0.00%	36	0.00%	1.14	1.24	0.93
mmu-mir-0542	X	50402580	50402664	-	MI0003522	526	0.01%	237	0.01%	137	0.01%	0.74	1.00	0.74
mmu-mir-0351	X	50406432	50406530	-	MI0000643	1598	0.03%	640	0.02%	217	0.01%	0.66	1.70	**0.39**
mmu-mir-0503	X	50407161	50407231	-	MI0003538	10588	0.18%	4551	0.13%	1385	0.07%	0.71	1.90	**0.37**
mmu-mir-0322	X	50407432	50407526	-	MI0000590	23834	0.41%	8550	0.24%	5024	0.25%	0.59	0.98	0.60
mmu-mir-0504	X	56350835	56350913	-	MI0005515	157	0.00%	65	0.00%	47	0.00%	0.68	0.80	0.85
mmu-mir-0505	X	57647578	57647667	-	MI0004706	37	0.00%	27	0.00%	15	0.00%	1.20	1.04	1.16
mmu-mir-0743a	X	64029932	64029993	-	MI0005207	207	0.00%	455	0.01%	705	0.03%	3.63	0.37	**9.73**
mmu-mir-0743b	X	64030431	64030507	-	MI0005470	1114	0.02%	3547	0.10%	7182	0.35%	5.25	0.29	**18.41**
mmu-mir-0742	X	64033548	64033612	-	MI0005206	25	0.00%	82	0.00%	117	0.01%	5.41	0.40	**13.37**
mmu-mir-0883a	X	64033933	64034008	-	MI0005476	1373	0.02%	3146	0.09%	4697	0.23%	3.78	0.39	**9.77**
mmu-mir-0883b	X	64043065	64043142	-	MI0005477	277	0.00%	628	0.02%	621	0.03%	3.74	0.58	**6.40**
mmu-mir-0471	X	64045770	64045836	-	MI0002406	126	0.00%	518	0.01%	542	0.03%	6.78	0.55	**12.28**
mmu-mir-0741	X	64049980	64050050	-	MI0005205	336	0.01%	951	0.03%	1036	0.05%	4.67	0.53	**8.81**
mmu-mir-0463	X	64052398	64052472	-	MI0002398	42	0.00%	70	0.00%	70	0.00%	2.75	0.58	**4.76**
mmu-mir-0880	X	64053705	64053782	-	MI0005473	197	0.00%	742	0.02%	689	0.03%	6.21	0.62	**9.99**
mmu-mir-0878	X	64054683	64054760	-	MI0005548	150	0.00%	429	0.01%	591	0.03%	4.72	0.42	**11.25**
mmu-mir-0881	X	64055119	64055196	-	MI0005474	1743	0.03%	4031	0.11%	5529	0.27%	3.82	0.42	**9.06**
mmu-mir-0871	X	64063603	64063679	-	MI0005471	567	0.01%	1415	0.04%	1520	0.07%	4.12	0.54	**7.66**
mmu-mir-0470	X	64067126	64067200	-	MI0002405	2096	0.04%	5754	0.16%	6328	0.31%	4.53	0.53	**8.62**
mmu-mir-0465c-1	X	64079130	64079210	-	MI0005500	1946	0.03%	5213	0.15%	4312	0.21%	4.42	0.70	**6.33**
mmu-mir-0465b-1	X	64082377	64082455	-	MI0005498	951	0.02%	2406	0.07%	2204	0.11%	4.17	0.63	**6.62**
mmu-mir-0465c-2	X	64085692	64085772	-	MI0005501	1946	0.03%	5213	0.15%	4312	0.21%	4.42	0.70	**6.33**
mmu-mir-0465b-2	X	64088939	64089017	-	MI0005499	951	0.02%	2406	0.07%	2204	0.11%	4.17	0.63	**6.62**
mmu-mir-0465a	X	64092227	64092300	-	MI0002400	1207	0.02%	2951	0.08%	2617	0.13%	4.03	0.65	**6.19**
mmu-mir-0201	X	65241271	65241336	-	MI0000244	13	0.00%	98	0.00%	58	0.00%	12.44	0.98	**12.74**
mmu-mir-0547	X	65241549	65241626	-	MI0003523	41	0.00%	120	0.00%	101	0.00%	4.83	0.69	**7.04**
mmu-mir-0509	X	65263280	65263354	-	MI0005516	0	0.00%	0	0.00%	0	0.00%	0.00	0.00	0.00
mmu-mir-0224	X	69506370	69506451	-	MI0000711	36	0.00%	32	0.00%	12	0.00%	1.47	1.54	0.95
mmu-mir-0452	X	69507563	69507647	-	MI0001734	30	0.00%	24	0.00%	10	0.00%	1.32	1.39	0.95
mmu-mir-0105	X	69836839	69836918	-	MI0005481	0	0.00%	0	0.00%	0	0.00%	0.00	0.00	0.00
mmu-mir-0718	X	71269188	71269275	-	MI0004707	0	0.00%	0	0.00%	0	0.00%	0.00	0.00	0.00
mmu-mir-0223	X	93438156	93438265	+	MI0000703	101	0.00%	97	0.00%	50	0.00%	1.58	1.12	1.41
mmu-mir-0676	X	97576436	97576524	+	MI0005003	1260	0.02%	728	0.02%	526	0.03%	0.95	0.80	1.19
mmu-mir-0421	X	100768260	100768335	-	MI0005496	41	0.00%	18	0.00%	14	0.00%	0.72	0.74	0.98
mmu-mir-0374	X	100768399	100768493	-	MI0004125	157	0.00%	31	0.00%	35	0.00%	0.33	0.51	0.64
mmu-mir-0672	X	101311514	101311613	-	MI0004258	7968	0.14%	6729	0.19%	3429	0.17%	1.39	1.13	1.23
mmu-mir-0384	X	102539621	102539708	-	MI0001146	2	0.00%	0	0.00%	0	0.00%	0.00	0.00	0.00
mmu-mir-0325	X	102574421	102574518	-	MI0000597	0	0.00%	1	0.00%	0	0.00%	0.00	0.00	0.00
mmu-mir-0361	X	110188433	110188502	-	MI0000761	109	0.00%	61	0.00%	35	0.00%	0.92	1.01	0.92

mmu-mir-0652	X	139173543	139173640	+	MI0004965	167	0.00%	94	0.00%	51	0.00%	0.93	1.06	0.87
mmu-mir-0680-2	X	140732333	140732442	+	MI0004641	0	0.00%	0	0.00%	0	0.00%	0.00	0.00	0.00
mmu-mir-0764	X	143436802	143436909	+	MI0004310	0	0.00%	0	0.00%	0	0.00%	0.00	0.00	0.00
mmu-mir-0448	X	143592753	143592864	+	MI0001638	3	0.00%	1	0.00%	0	0.00%	0.55	0.00	0.00
mmu-let-7f-2	X	148346889	148346971	+	MI0000563	779322	13.41%	437857	12.43%	301811	14.83%	0.93	0.84	1.11
mmu-mir-0098	X	148347757	148347864	+	MI0000586	1918	0.03%	1262	0.04%	808	0.04%	1.09	0.90	1.20
