# Supplementary material for: Analysis of MicroRNA Expression in the Prepubertal Testis
Source: PLoS One. 2010 Dec 29;5(12):e15317. doi: 10.1371/journal.pone.0015317 (PMC3012074; doi:10.1371/journal.pone.0015317)
Supplement: Table S3 — Editing of miRNAs during prepubertal testicular development. Four types of editing were evaluated by assigning reads that were not an exact match to the mature miRNA sequence: a) alteration of 5′ end cleavage, b) A to G transitions representing putative editing by ADARs, c) internal insertions of uridine by an unknown process, and d) 3′ addition of A(n) or (U)n. [Indel, insertion/deletion] (PDF) [file pone.0015317.s003.pdf]

miRNA	Chr	Start	Stop	Strand	Type	Position	P7 reads	P7%	P10 reads	P10%	P14 reads	P14%
mmu-let-7a-1-5p	13	48633608	48633629	-	5' Edit	5'	1595	0.4%	0	0.0%	696	0.6%
mmu-let-7a-1-5p	13	48633608	48633629	-	5' Indel	5'	104	0.0%	0	0.0%	36	0.0%
mmu-let-7a-1-5p	13	48633608	48633629	-	Indel	21	10	0.0%	0	0.0%	5	0.0%
mmu-let-7a-1-5p	13	48633608	48633629	-	edit	1	0	0.0%	0	0.0%	0	0.0%
mmu-let-7a-1-5p	13	48633608	48633629	-	edit	2	0	0.0%	0	0.0%	0	0.0%
mmu-let-7a-1-5p	13	48633608	48633629	-	edit	3	0	0.0%	0	0.0%	0	0.0%
mmu-let-7a-1-5p	13	48633608	48633629	-	edit	4	64	0.0%	46	0.0%	21	0.0%
mmu-let-7a-1-5p	13	48633608	48633629	-	edit	5	7	0.0%	0	0.0%	0	0.0%
mmu-let-7a-1-5p	13	48633608	48633629	-	edit	6	1321	0.4%	386	0.2%	798	0.7%
mmu-let-7a-1-5p	13	48633608	48633629	-	edit	7	0	0.0%	0	0.0%	0	0.0%
mmu-let-7a-1-5p	13	48633608	48633629	-	edit	8	233	0.1%	48	0.0%	27	0.0%
mmu-let-7a-1-5p	13	48633608	48633629	-	edit	9	1256	0.3%	186	0.1%	245	0.2%
mmu-let-7a-1-5p	13	48633608	48633629	-	edit	10	22	0.0%	23	0.0%	9	0.0%
mmu-let-7a-1-5p	13	48633608	48633629	-	edit	11	8	0.0%	0	0.0%	6	0.0%
mmu-let-7a-1-5p	13	48633608	48633629	-	edit	12	0	0.0%	0	0.0%	0	0.0%
mmu-let-7a-1-5p	13	48633608	48633629	-	edit	13	351	0.1%	48	0.0%	167	0.2%
mmu-let-7a-1-5p	13	48633608	48633629	-	edit	14	3	0.0%	0	0.0%	3	0.0%
mmu-let-7a-1-5p	13	48633608	48633629	-	edit	15	321	0.1%	168	0.1%	79	0.1%
mmu-let-7a-1-5p	13	48633608	48633629	-	edit	16	304	0.1%	110	0.1%	101	0.1%
mmu-let-7a-1-5p	13	48633608	48633629	-	edit	17	3999	1.1%	2929	1.5%	1159	1.1%
mmu-let-7a-1-5p	13	48633608	48633629	-	edit	18	51	0.0%	20	0.0%	10	0.0%
mmu-let-7a-1-5p	13	48633608	48633629	-	edit	19	1579	0.4%	1233	0.6%	471	0.4%
mmu-let-7a-1-5p	13	48633608	48633629	-	edit	20	48	0.0%	28	0.0%	14	0.0%
mmu-let-7a-1-5p	13	48633608	48633629	-	edit	21	556	0.2%	87	0.0%	258	0.2%
mmu-let-7a-1-5p	13	48633608	48633629	-	edit	22	185	0.1%	54	0.0%	55	0.1%
EDITED							12012	3.3%	5364	2.7%	4159	3.8%
CANONICAL							356489	96.7%	193610	97.3%	103860	96.2%
TOTAL							368501		198974		108019	

miRNA	Chr	Start	Stop	Strand	Type	Position	P7 reads	P7%	P10 reads	P10%	P14 reads	P14%
mmu-let-7a-2-5p	9	41344815	41344836	+	3' Edit	3'	92	0.0%	0	0.0%	30	0.0%
mmu-let-7a-2-5p	9	41344815	41344836	+	3' Indel	3'	20	0.0%	0	0.0%	7	0.0%
mmu-let-7a-2-5p	9	41344815	41344836	+	Indel	20	29	0.0%	0	0.0%	9	0.0%
mmu-let-7a-2-5p	9	41344815	41344836	+	Indel	22	122	0.0%	24	0.0%	50	0.0%
mmu-let-7a-2-5p	9	41344815	41344836	+	edit	1	0	0.0%	0	0.0%	0	0.0%
mmu-let-7a-2-5p	9	41344815	41344836	+	edit	2	0	0.0%	0	0.0%	0	0.0%
mmu-let-7a-2-5p	9	41344815	41344836	+	edit	3	0	0.0%	0	0.0%	0	0.0%
mmu-let-7a-2-5p	9	41344815	41344836	+	edit	4	62	0.0%	45	0.0%	19	0.0%
mmu-let-7a-2-5p	9	41344815	41344836	+	edit	5	6	0.0%	0	0.0%	0	0.0%
mmu-let-7a-2-5p	9	41344815	41344836	+	edit	6	1284	0.4%	380	0.2%	790	0.8%
mmu-let-7a-2-5p	9	41344815	41344836	+	edit	7	0	0.0%	0	0.0%	0	0.0%
mmu-let-7a-2-5p	9	41344815	41344836	+	edit	8	219	0.1%	50	0.0%	21	0.0%
mmu-let-7a-2-5p	9	41344815	41344836	+	edit	9	2520	0.7%	361	0.2%	536	0.5%
mmu-let-7a-2-5p	9	41344815	41344836	+	edit	10	35	0.0%	42	0.0%	14	0.0%
mmu-let-7a-2-5p	9	41344815	41344836	+	edit	11	12	0.0%	0	0.0%	9	0.0%
mmu-let-7a-2-5p	9	41344815	41344836	+	edit	12	1104	0.3%	215	0.1%	570	0.6%
mmu-let-7a-2-5p	9	41344815	41344836	+	edit	13	259	0.1%	38	0.0%	187	0.2%
mmu-let-7a-2-5p	9	41344815	41344836	+	edit	14	20	0.0%	9	0.0%	14	0.0%
mmu-let-7a-2-5p	9	41344815	41344836	+	edit	15	301	0.1%	159	0.1%	71	0.1%
mmu-let-7a-2-5p	9	41344815	41344836	+	edit	16	306	0.1%	114	0.1%	104	0.1%
mmu-let-7a-2-5p	9	41344815	41344836	+	edit	17	2256	0.7%	2332	1.2%	685	0.7%
mmu-let-7a-2-5p	9	41344815	41344836	+	edit	18	49	0.0%	19	0.0%	9	0.0%
mmu-let-7a-2-5p	9	41344815	41344836	+	edit	19	1388	0.4%	1135	0.6%	402	0.4%
mmu-let-7a-2-5p	9	41344815	41344836	+	edit	20	88	0.0%	31	0.0%	34	0.0%
mmu-let-7a-2-5p	9	41344815	41344836	+	edit	21	921	0.3%	112	0.1%	389	0.4%
mmu-let-7a-2-5p	9	41344815	41344836	+	edit	22	418	0.1%	80	0.0%	183	0.2%
EDITED							11507	3.3%	5142	2.7%	4129	4.0%
CANONICAL							333447	96.7%	185125	97.3%	98375	96.0%
TOTAL							344954		190267		102504	

miRNA	Chr	Start	Stop	Strand	Type	Position	P7 reads	P7%	P10 reads	P10%	P14 reads	P14%
mmu-let-7b-3p	15	85537809	85537830	+	edit	1	0	0.0%	0	0.0%	0	0.0%
mmu-let-7b-3p	15	85537809	85537830	+	edit	2	13	1.6%	9	3.3%	7	3.1%
mmu-let-7b-3p	15	85537809	85537830	+	edit	3	0	0.0%	0	0.0%	0	0.0%
mmu-let-7b-3p	15	85537809	85537830	+	edit	4	0	0.0%	0	0.0%	0	0.0%
mmu-let-7b-3p	15	85537809	85537830	+	edit	5	0	0.0%	0	0.0%	0	0.0%
mmu-let-7b-3p	15	85537809	85537830	+	edit	6	0	0.0%	0	0.0%	0	0.0%
mmu-let-7b-3p	15	85537809	85537830	+	edit	7	0	0.0%	0	0.0%	0	0.0%
mmu-let-7b-3p	15	85537809	85537830	+	edit	8	0	0.0%	0	0.0%	0	0.0%
mmu-let-7b-3p	15	85537809	85537830	+	edit	9	0	0.0%	0	0.0%	0	0.0%
mmu-let-7b-3p	15	85537809	85537830	+	edit	10	0	0.0%	0	0.0%	0	0.0%
mmu-let-7b-3p	15	85537809	85537830	+	edit	11	0	0.0%	3	1.0%	0	0.0%
mmu-let-7b-3p	15	85537809	85537830	+	edit	12	0	0.0%	0	0.0%	0	0.0%
mmu-let-7b-3p	15	85537809	85537830	+	edit	13	0	0.0%	0	0.0%	0	0.0%
mmu-let-7b-3p	15	85537809	85537830	+	edit	14	0	0.0%	0	0.0%	0	0.0%
mmu-let-7b-3p	15	85537809	85537830	+	edit	15	**211**	**26.3%**	**52**	**18.2%**	**35**	**14.9%**
mmu-let-7b-3p	15	85537809	85537830	+	edit	16	0	0.0%	0	0.0%	0	0.0%
mmu-let-7b-3p	15	85537809	85537830	+	edit	17	0	0.0%	0	0.0%	0	0.0%
mmu-let-7b-3p	15	85537809	85537830	+	edit	18	0	0.0%	0	0.0%	0	0.0%
mmu-let-7b-3p	15	85537809	85537830	+	edit	19	0	0.0%	0	0.0%	0	0.0%
mmu-let-7b-3p	15	85537809	85537830	+	edit	20	0	0.0%	0	0.0%	0	0.0%
mmu-let-7b-3p	15	85537809	85537830	+	edit	21	0	0.0%	0	0.0%	0	0.0%
mmu-let-7b-3p	15	85537809	85537830	+	edit	22	0	0.0%	0	0.0%	0	0.0%
EDITED							224	27.9%	65	22.6%	43	18.0%
CANONICAL							578	72.1%	222	77.4%	195	82.0%
TOTAL							802		287		238	

miRNA	Chr	Start	Stop	Strand	Type	Position	P7 reads	P7%	P10 reads	P10%	P14 reads	P14%
mmu-let-7b-5p	15	85537755	85537776	+	3' Edit	3'	120	0.0%	0	0.0%	59	0.0%
mmu-let-7b-5p	15	85537755	85537776	+	3' Indel	3'	15	0.0%	0	0.0%	12	0.0%
mmu-let-7b-5p	15	85537755	85537776	+	Indel	17	28	0.0%	11	0.0%	5	0.0%
mmu-let-7b-5p	15	85537755	85537776	+	Indel	18	206	0.0%	104	0.0%	116	0.1%
mmu-let-7b-5p	15	85537755	85537776	+	Indel	19	0	0.0%	0	0.0%	0	0.0%
mmu-let-7b-5p	15	85537755	85537776	+	Indel	20	75	0.0%	40	0.0%	27	0.0%
mmu-let-7b-5p	15	85537755	85537776	+	Indel	21	15	0.0%	0	0.0%	0	0.0%
mmu-let-7b-5p	15	85537755	85537776	+	Indel	22	24	0.0%	9	0.0%	12	0.0%
mmu-let-7b-5p	15	85537755	85537776	+	edit	1	0	0.0%	0	0.0%	0	0.0%
mmu-let-7b-5p	15	85537755	85537776	+	edit	2	0	0.0%	0	0.0%	0	0.0%
mmu-let-7b-5p	15	85537755	85537776	+	edit	3	0	0.0%	0	0.0%	0	0.0%
mmu-let-7b-5p	15	85537755	85537776	+	edit	4	132	0.0%	138	0.0%	48	0.0%
mmu-let-7b-5p	15	85537755	85537776	+	edit	5	0	0.0%	0	0.0%	0	0.0%
mmu-let-7b-5p	15	85537755	85537776	+	edit	6	1551	0.3%	804	0.2%	919	0.5%
mmu-let-7b-5p	15	85537755	85537776	+	edit	7	0	0.0%	0	0.0%	0	0.0%
mmu-let-7b-5p	15	85537755	85537776	+	edit	8	413	0.1%	135	0.0%	52	0.0%
mmu-let-7b-5p	15	85537755	85537776	+	edit	9	0	0.0%	0	0.0%	0	0.0%
mmu-let-7b-5p	15	85537755	85537776	+	edit	10	18	0.0%	32	0.0%	0	0.0%
mmu-let-7b-5p	15	85537755	85537776	+	edit	11	0	0.0%	0	0.0%	0	0.0%
mmu-let-7b-5p	15	85537755	85537776	+	edit	12	33	0.0%	19	0.0%	6	0.0%
mmu-let-7b-5p	15	85537755	85537776	+	edit	13	12	0.0%	3	0.0%	11	0.0%
mmu-let-7b-5p	15	85537755	85537776	+	edit	14	0	0.0%	0	0.0%	0	0.0%
mmu-let-7b-5p	15	85537755	85537776	+	edit	15	552	0.1%	484	0.1%	145	0.1%
mmu-let-7b-5p	15	85537755	85537776	+	edit	16	0	0.0%	0	0.0%	0	0.0%
mmu-let-7b-5p	15	85537755	85537776	+	edit	17	399	0.1%	980	0.2%	134	0.1%
mmu-let-7b-5p	15	85537755	85537776	+	edit	18	4	0.0%	3	0.0%	0	0.0%
mmu-let-7b-5p	15	85537755	85537776	+	edit	19	4202	0.8%	2845	0.7%	1151	0.6%
mmu-let-7b-5p	15	85537755	85537776	+	edit	20	28	0.0%	17	0.0%	5	0.0%
mmu-let-7b-5p	15	85537755	85537776	+	edit	21	641	0.1%	306	0.1%	345	0.2%
mmu-let-7b-5p	15	85537755	85537776	+	edit	22	36	0.0%	9	0.0%	12	0.0%
EDITED							8502	1.5%	5938	1.4%	3058	1.7%
CANONICAL							549565	98.5%	410791	98.6%	176895	98.3%
TOTAL							558067		416729		179953	

miRNA	Chr	Start	Stop	Strand	Type	Position	P7 reads	P7%	P10 reads	P10%	P14 reads	P14%
mmu-let-7c-1-5p	16	77599917	77599938	+	3' Edit	3'	232	0.0%	0	0.0%	82	0.0%
mmu-let-7c-1-5p	16	77599917	77599938	+	3' Indel	3'	25	0.0%	0	0.0%	19	0.0%
mmu-let-7c-1-5p	16	77599917	77599938	+	Indel	18	110	0.0%	19	0.0%	50	0.0%
mmu-let-7c-1-5p	16	77599917	77599938	+	Indel	19	0	0.0%	0	0.0%	0	0.0%
mmu-let-7c-1-5p	16	77599917	77599938	+	Indel	20	196	0.0%	48	0.0%	91	0.0%
mmu-let-7c-1-5p	16	77599917	77599938	+	Indel	21	150	0.0%	49	0.0%	125	0.1%
mmu-let-7c-1-5p	16	77599917	77599938	+	Indel	22	701	0.1%	176	0.0%	397	0.2%
mmu-let-7c-1-5p	16	77599917	77599938	+	edit	1	0	0.0%	0	0.0%	0	0.0%
mmu-let-7c-1-5p	16	77599917	77599938	+	edit	2	0	0.0%	0	0.0%	0	0.0%
mmu-let-7c-1-5p	16	77599917	77599938	+	edit	3	0	0.0%	0	0.0%	0	0.0%
mmu-let-7c-1-5p	16	77599917	77599938	+	edit	4	182	0.0%	174	0.0%	77	0.0%
mmu-let-7c-1-5p	16	77599917	77599938	+	edit	5	0	0.0%	0	0.0%	0	0.0%
mmu-let-7c-1-5p	16	77599917	77599938	+	edit	6	2889	0.4%	1270	0.3%	2037	0.8%
mmu-let-7c-1-5p	16	77599917	77599938	+	edit	7	0	0.0%	0	0.0%	0	0.0%
mmu-let-7c-1-5p	16	77599917	77599938	+	edit	8	532	0.1%	141	0.0%	64	0.0%
mmu-let-7c-1-5p	16	77599917	77599938	+	edit	9	2163	0.3%	445	0.1%	314	0.1%
mmu-let-7c-1-5p	16	77599917	77599938	+	edit	10	22	0.0%	20	0.0%	5	0.0%
mmu-let-7c-1-5p	16	77599917	77599938	+	edit	11	7	0.0%	3	0.0%	9	0.0%
mmu-let-7c-1-5p	16	77599917	77599938	+	edit	12	268	0.0%	238	0.0%	72	0.0%
mmu-let-7c-1-5p	16	77599917	77599938	+	edit	13	449	0.1%	112	0.0%	344	0.1%
mmu-let-7c-1-5p	16	77599917	77599938	+	edit	14	3	0.0%	3	0.0%	7	0.0%
mmu-let-7c-1-5p	16	77599917	77599938	+	edit	15	653	0.1%	466	0.1%	163	0.1%
mmu-let-7c-1-5p	16	77599917	77599938	+	edit	16	36	0.0%	16	0.0%	10	0.0%
mmu-let-7c-1-5p	16	77599917	77599938	+	edit	17	222	0.0%	237	0.0%	105	0.0%
mmu-let-7c-1-5p	16	77599917	77599938	+	edit	18	3	0.0%	3	0.0%	0	0.0%
mmu-let-7c-1-5p	16	77599917	77599938	+	edit	19	1385	0.2%	1134	0.2%	398	0.2%
mmu-let-7c-1-5p	16	77599917	77599938	+	edit	20	38	0.0%	9	0.0%	25	0.0%
mmu-let-7c-1-5p	16	77599917	77599938	+	edit	21	1905	0.3%	386	0.1%	989	0.4%
mmu-let-7c-1-5p	16	77599917	77599938	+	edit	22	1101	0.2%	363	0.1%	798	0.3%
EDITED							13270	1.9%	5311	1.1%	6180	2.5%
CANONICAL							699775	98.1%	492893	98.9%	241358	97.5%
TOTAL							713045		498204		247538	

miRNA	Chr	Start	Stop	Strand	Type	Position	P7 reads	P7%	P10 reads	P10%	P14 reads	P14%
mmu-let-7c-2-5p	15	85537046	85537067	+	3' Edit	3'	5169	0.6%	0	0.0%	1791	0.6%
mmu-let-7c-2-5p	15	85537046	85537067	+	3' Indel	3'	4	0.0%	0	0.0%	3	0.0%
mmu-let-7c-2-5p	15	85537046	85537067	+	Indel	18	231	0.0%	51	0.0%	106	0.0%
mmu-let-7c-2-5p	15	85537046	85537067	+	Indel	19	0	0.0%	0	0.0%	0	0.0%
mmu-let-7c-2-5p	15	85537046	85537067	+	Indel	20	57	0.0%	20	0.0%	28	0.0%
mmu-let-7c-2-5p	15	85537046	85537067	+	Indel	21	132	0.0%	34	0.0%	87	0.0%
mmu-let-7c-2-5p	15	85537046	85537067	+	Indel	22	583	0.1%	256	0.0%	232	0.1%
mmu-let-7c-2-5p	15	85537046	85537067	+	edit	1	0	0.0%	0	0.0%	0	0.0%
mmu-let-7c-2-5p	15	85537046	85537067	+	edit	2	0	0.0%	0	0.0%	0	0.0%
mmu-let-7c-2-5p	15	85537046	85537067	+	edit	3	0	0.0%	0	0.0%	0	0.0%
mmu-let-7c-2-5p	15	85537046	85537067	+	edit	4	199	0.0%	181	0.0%	84	0.0%
mmu-let-7c-2-5p	15	85537046	85537067	+	edit	5	0	0.0%	0	0.0%	0	0.0%
mmu-let-7c-2-5p	15	85537046	85537067	+	edit	6	3254	0.4%	1321	0.2%	2317	0.8%
mmu-let-7c-2-5p	15	85537046	85537067	+	edit	7	0	0.0%	0	0.0%	0	0.0%
mmu-let-7c-2-5p	15	85537046	85537067	+	edit	8	595	0.1%	147	0.0%	67	0.0%
mmu-let-7c-2-5p	15	85537046	85537067	+	edit	9	5864	0.7%	968	0.2%	724	0.3%
mmu-let-7c-2-5p	15	85537046	85537067	+	edit	10	78	0.0%	65	0.0%	25	0.0%
mmu-let-7c-2-5p	15	85537046	85537067	+	edit	11	10	0.0%	5	0.0%	10	0.0%
mmu-let-7c-2-5p	15	85537046	85537067	+	edit	12	583	0.1%	317	0.1%	118	0.0%
mmu-let-7c-2-5p	15	85537046	85537067	+	edit	13	536	0.1%	133	0.0%	356	0.1%
mmu-let-7c-2-5p	15	85537046	85537067	+	edit	14	8	0.0%	0	0.0%	3	0.0%
mmu-let-7c-2-5p	15	85537046	85537067	+	edit	15	760	0.1%	526	0.1%	187	0.1%
mmu-let-7c-2-5p	15	85537046	85537067	+	edit	16	290	0.0%	98	0.0%	71	0.0%
mmu-let-7c-2-5p	15	85537046	85537067	+	edit	17	5645	0.7%	3913	0.7%	2076	0.7%
mmu-let-7c-2-5p	15	85537046	85537067	+	edit	18	0	0.0%	0	0.0%	0	0.0%
mmu-let-7c-2-5p	15	85537046	85537067	+	edit	19	2516	0.3%	1552	0.3%	769	0.3%
mmu-let-7c-2-5p	15	85537046	85537067	+	edit	20	35	0.0%	24	0.0%	13	0.0%
mmu-let-7c-2-5p	15	85537046	85537067	+	edit	21	2431	0.3%	584	0.1%	1242	0.4%
mmu-let-7c-2-5p	15	85537046	85537067	+	edit	22	957	0.1%	347	0.1%	534	0.2%
mmu-let-7c-2-5p							29935	3.6%	10540	2.0%	10843	3.8%
mmu-let-7c-2-5p							792556	96.4%	527611	98.0%	275775	96.2%
mmu-let-7c-2-5p							822491		538151		286618	

miRNA	Chr	Start	Stop	Strand	Type	Position	P7 reads	P7%	P10 reads	P10%	P14 reads	P14%
mmu-let-7d-5p	13	48631447	48631468	-	5' Edit	5'	219	0.2%	0	0.0%	92	0.3%
mmu-let-7d-5p	13	48631447	48631468	-	5' Indel	5'	12	0.0%	0	0.0%	3	0.0%
mmu-let-7d-5p	13	48631447	48631468	-	edit	1	0	0.0%	0	0.0%	0	0.0%
mmu-let-7d-5p	13	48631447	48631468	-	edit	2	0	0.0%	0	0.0%	0	0.0%
mmu-let-7d-5p	13	48631447	48631468	-	edit	3	0	0.0%	0	0.0%	0	0.0%
mmu-let-7d-5p	13	48631447	48631468	-	edit	4	30	0.0%	7	0.0%	3	0.0%
mmu-let-7d-5p	13	48631447	48631468	-	edit	5	0	0.0%	0	0.0%	0	0.0%
mmu-let-7d-5p	13	48631447	48631468	-	edit	6	26	0.0%	10	0.0%	19	0.1%
mmu-let-7d-5p	13	48631447	48631468	-	edit	7	0	0.0%	0	0.0%	0	0.0%
mmu-let-7d-5p	13	48631447	48631468	-	edit	8	97	0.1%	17	0.0%	7	0.0%
mmu-let-7d-5p	13	48631447	48631468	-	edit	9	49	0.0%	11	0.0%	6	0.0%
mmu-let-7d-5p	13	48631447	48631468	-	edit	10	3	0.0%	0	0.0%	0	0.0%
mmu-let-7d-5p	13	48631447	48631468	-	edit	11	0	0.0%	0	0.0%	0	0.0%
mmu-let-7d-5p	13	48631447	48631468	-	edit	12	8	0.0%	0	0.0%	0	0.0%
mmu-let-7d-5p	13	48631447	48631468	-	edit	13	101	0.1%	23	0.0%	62	0.2%
mmu-let-7d-5p	13	48631447	48631468	-	edit	14	0	0.0%	0	0.0%	0	0.0%
mmu-let-7d-5p	13	48631447	48631468	-	edit	15	155	0.1%	69	0.1%	23	0.1%
mmu-let-7d-5p	13	48631447	48631468	-	edit	16	164	0.1%	179	0.3%	91	0.2%
mmu-let-7d-5p	13	48631447	48631468	-	edit	17	933	0.7%	1893	3.0%	290	0.8%
mmu-let-7d-5p	13	48631447	48631468	-	edit	18	28	0.0%	8	0.0%	3	0.0%
mmu-let-7d-5p	13	48631447	48631468	-	edit	19	1326	1.1%	1289	2.0%	270	0.7%
mmu-let-7d-5p	13	48631447	48631468	-	edit	20	13	0.0%	0	0.0%	4	0.0%
mmu-let-7d-5p	13	48631447	48631468	-	edit	21	136	0.1%	22	0.0%	54	0.1%
mmu-let-7d-5p	13	48631447	48631468	-	edit	22	36	0.0%	6	0.0%	6	0.0%
EDITED							3335	2.7%	3531	5.6%	932	2.5%
CANONICAL							121532	97.3%	60054	94.4%	35740	97.5%
TOTAL							124867		63585		36672	

miRNA	Chr	Start	Stop	Strand	Type	Position	P7 reads	P7%	P10 reads	P10%	P14 reads	P14%
mmu-let-7e-5p	17	17967330	17967351	+	3' Edit	3'	954	0.3%	0	0.0%	160	0.3%
mmu-let-7e-5p	17	17967330	17967351	+	3' Indel	3'	128	0.0%	0	0.0%	29	0.1%
mmu-let-7e-5p	17	17967330	17967351	+	Indel	19	6	0.0%	0	0.0%	0	0.0%
mmu-let-7e-5p	17	17967330	17967351	+	Indel	20	20	0.0%	0	0.0%	4	0.0%
mmu-let-7e-5p	17	17967330	17967351	+	Indel	21	4	0.0%	0	0.0%	0	0.0%
mmu-let-7e-5p	17	17967330	17967351	+	Indel	22	57	0.0%	5	0.0%	6	0.0%
mmu-let-7e-5p	17	17967330	17967351	+	edit	1	0	0.0%	0	0.0%	0	0.0%
mmu-let-7e-5p	17	17967330	17967351	+	edit	2	0	0.0%	0	0.0%	0	0.0%
mmu-let-7e-5p	17	17967330	17967351	+	edit	3	0	0.0%	0	0.0%	0	0.0%
mmu-let-7e-5p	17	17967330	17967351	+	edit	4	53	0.0%	20	0.0%	13	0.0%
mmu-let-7e-5p	17	17967330	17967351	+	edit	5	0	0.0%	0	0.0%	0	0.0%
mmu-let-7e-5p	17	17967330	17967351	+	edit	6	1392	0.4%	295	0.3%	497	1.0%
mmu-let-7e-5p	17	17967330	17967351	+	edit	7	0	0.0%	0	0.0%	0	0.0%
mmu-let-7e-5p	17	17967330	17967351	+	edit	8	173	0.1%	20	0.0%	5	0.0%
mmu-let-7e-5p	17	17967330	17967351	+	edit	9	16	0.0%	3	0.0%	10	0.0%
mmu-let-7e-5p	17	17967330	17967351	+	edit	10	0	0.0%	0	0.0%	0	0.0%
mmu-let-7e-5p	17	17967330	17967351	+	edit	11	0	0.0%	0	0.0%	0	0.0%
mmu-let-7e-5p	17	17967330	17967351	+	edit	12	10	0.0%	0	0.0%	0	0.0%
mmu-let-7e-5p	17	17967330	17967351	+	edit	13	3	0.0%	0	0.0%	0	0.0%
mmu-let-7e-5p	17	17967330	17967351	+	edit	14	0	0.0%	0	0.0%	0	0.0%
mmu-let-7e-5p	17	17967330	17967351	+	edit	15	177	0.1%	43	0.0%	19	0.0%
mmu-let-7e-5p	17	17967330	17967351	+	edit	16	16	0.0%	0	0.0%	0	0.0%
mmu-let-7e-5p	17	17967330	17967351	+	edit	17	25	0.0%	0	0.0%	0	0.0%
mmu-let-7e-5p	17	17967330	17967351	+	edit	18	0	0.0%	0	0.0%	0	0.0%
mmu-let-7e-5p	17	17967330	17967351	+	edit	19	7360	2.3%	2135	2.1%	1094	2.3%
mmu-let-7e-5p	17	17967330	17967351	+	edit	20	0	0.0%	0	0.0%	0	0.0%
mmu-let-7e-5p	17	17967330	17967351	+	edit	21	84	0.0%	10	0.0%	14	0.0%
mmu-let-7e-5p	17	17967330	17967351	+	edit	22	17	0.0%	7	0.0%	0	0.0%
EDITED							10493	3.3%	2539	2.5%	1851	3.9%
CANONICAL							308133	96.7%	97522	97.5%	45983	96.1%
TOTAL							318626		100061		47834	

miRNA	Chr	Start	Stop	Strand	Type	Position	P7 reads	P7%	P10 reads	P10%	P14 reads	P14%
mmu-let-7f-1-5p	13	48633258	48633279	-	5' Edit	5'	1028	0.2%	0	0.0%	400	0.2%
mmu-let-7f-1-5p	13	48633258	48633279	-	5' Indel	5'	33	0.0%	0	0.0%	19	0.0%
mmu-let-7f-1-5p	13	48633258	48633279	-	Indel	21	99	0.0%	22	0.0%	43	0.0%
mmu-let-7f-1-5p	13	48633258	48633279	-	Indel	22	0	0.0%	0	0.0%	5	0.0%
mmu-let-7f-1-5p	13	48633258	48633279	-	edit	1	0	0.0%	0	0.0%	0	0.0%
mmu-let-7f-1-5p	13	48633258	48633279	-	edit	2	0	0.0%	0	0.0%	0	0.0%
mmu-let-7f-1-5p	13	48633258	48633279	-	edit	3	0	0.0%	0	0.0%	0	0.0%
mmu-let-7f-1-5p	13	48633258	48633279	-	edit	4	136	0.0%	86	0.0%	49	0.0%
mmu-let-7f-1-5p	13	48633258	48633279	-	edit	5	0	0.0%	0	0.0%	0	0.0%
mmu-let-7f-1-5p	13	48633258	48633279	-	edit	6	2330	0.4%	737	0.2%	1673	0.7%
mmu-let-7f-1-5p	13	48633258	48633279	-	edit	7	0	0.0%	0	0.0%	0	0.0%
mmu-let-7f-1-5p	13	48633258	48633279	-	edit	8	401	0.1%	85	0.0%	53	0.0%
mmu-let-7f-1-5p	13	48633258	48633279	-	edit	9	11	0.0%	0	0.0%	0	0.0%
mmu-let-7f-1-5p	13	48633258	48633279	-	edit	10	0	0.0%	0	0.0%	0	0.0%
mmu-let-7f-1-5p	13	48633258	48633279	-	edit	11	0	0.0%	0	0.0%	0	0.0%
mmu-let-7f-1-5p	13	48633258	48633279	-	edit	12	4	0.0%	0	0.0%	0	0.0%
mmu-let-7f-1-5p	13	48633258	48633279	-	edit	13	0	0.0%	0	0.0%	6	0.0%
mmu-let-7f-1-5p	13	48633258	48633279	-	edit	14	0	0.0%	0	0.0%	0	0.0%
mmu-let-7f-1-5p	13	48633258	48633279	-	edit	15	552	0.1%	295	0.1%	152	0.1%
mmu-let-7f-1-5p	13	48633258	48633279	-	edit	16	571	0.1%	245	0.1%	178	0.1%
mmu-let-7f-1-5p	13	48633258	48633279	-	edit	17	838	0.1%	2061	0.6%	308	0.1%
mmu-let-7f-1-5p	13	48633258	48633279	-	edit	18	7	0.0%	0	0.0%	4	0.0%
mmu-let-7f-1-5p	13	48633258	48633279	-	edit	19	1885	0.3%	2227	0.6%	457	0.2%
mmu-let-7f-1-5p	13	48633258	48633279	-	edit	20	3	0.0%	0	0.0%	0	0.0%
mmu-let-7f-1-5p	13	48633258	48633279	-	edit	21	36	0.0%	25	0.0%	36	0.0%
mmu-let-7f-1-5p	13	48633258	48633279	-	edit	22	223	0.0%	417	0.1%	173	0.1%
EDITED							8155	1.3%	6198	1.7%	3557	1.6%
CANONICAL							607029	98.7%	352131	98.3%	225350	98.4%
TOTAL							615184		358329		228907	

miRNA	Chr	Start	Stop	Strand	Type	Position	P7 reads	P7%	P10 reads	P10%	P14 reads	P14%
mmu-let-7f-2-5p	X	148346896	148346917	+	3' Edit	3'	2385	0.3%	0	0.0%	1243	0.4%
mmu-let-7f-2-5p	X	148346896	148346917	+	3' Indel	3'	11	0.0%	0	0.0%	4	0.0%
mmu-let-7f-2-5p	X	148346896	148346917	+	Indel	19	5	0.0%	0	0.0%	0	0.0%
mmu-let-7f-2-5p	X	148346896	148346917	+	Indel	20	13	0.0%	0	0.0%	0	0.0%
mmu-let-7f-2-5p	X	148346896	148346917	+	Indel	21	5	0.0%	0	0.0%	0	0.0%
mmu-let-7f-2-5p	X	148346896	148346917	+	Indel	22	53	0.0%	13	0.0%	24	0.0%
mmu-let-7f-2-5p	X	148346896	148346917	+	edit	1	0	0.0%	0	0.0%	0	0.0%
mmu-let-7f-2-5p	X	148346896	148346917	+	edit	2	0	0.0%	0	0.0%	0	0.0%
mmu-let-7f-2-5p	X	148346896	148346917	+	edit	3	0	0.0%	0	0.0%	0	0.0%
mmu-let-7f-2-5p	X	148346896	148346917	+	edit	4	186	0.0%	107	0.0%	68	0.0%
mmu-let-7f-2-5p	X	148346896	148346917	+	edit	5	0	0.0%	0	0.0%	0	0.0%
mmu-let-7f-2-5p	X	148346896	148346917	+	edit	6	3586	0.5%	1008	0.2%	2562	0.8%
mmu-let-7f-2-5p	X	148346896	148346917	+	edit	7	0	0.0%	0	0.0%	0	0.0%
mmu-let-7f-2-5p	X	148346896	148346917	+	edit	8	498	0.1%	105	0.0%	67	0.0%
mmu-let-7f-2-5p	X	148346896	148346917	+	edit	9	0	0.0%	0	0.0%	0	0.0%
mmu-let-7f-2-5p	X	148346896	148346917	+	edit	10	117	0.0%	126	0.0%	53	0.0%
mmu-let-7f-2-5p	X	148346896	148346917	+	edit	11	0	0.0%	0	0.0%	0	0.0%
mmu-let-7f-2-5p	X	148346896	148346917	+	edit	12	286	0.0%	168	0.0%	77	0.0%
mmu-let-7f-2-5p	X	148346896	148346917	+	edit	13	6	0.0%	0	0.0%	9	0.0%
mmu-let-7f-2-5p	X	148346896	148346917	+	edit	14	3	0.0%	0	0.0%	0	0.0%
mmu-let-7f-2-5p	X	148346896	148346917	+	edit	15	879	0.1%	427	0.1%	279	0.1%
mmu-let-7f-2-5p	X	148346896	148346917	+	edit	16	906	0.1%	370	0.1%	261	0.1%
mmu-let-7f-2-5p	X	148346896	148346917	+	edit	17	1558	0.2%	3028	0.7%	558	0.2%
mmu-let-7f-2-5p	X	148346896	148346917	+	edit	18	0	0.0%	0	0.0%	0	0.0%
mmu-let-7f-2-5p	X	148346896	148346917	+	edit	19	2939	0.4%	3560	0.8%	832	0.3%
mmu-let-7f-2-5p	X	148346896	148346917	+	edit	20	31	0.0%	4	0.0%	19	0.0%
mmu-let-7f-2-5p	X	148346896	148346917	+	edit	21	897	0.1%	119	0.0%	489	0.2%
mmu-let-7f-2-5p	X	148346896	148346917	+	edit	22	290	0.0%	73	0.0%	109	0.0%
EDITED							14653	1.8%	9107	2.0%	6650	2.2%
CANONICAL							779320	98.2%	437857	98.0%	301811	97.8%
TOTAL							793973		446964		308461	

miRNA	Chr	Start	Stop	Strand	Type	Position	P7 reads	P7%	P10 reads	P10%	P14 reads	P14%
mmu-let-7g-5p	9	106081177	106081198	+	Indel	19	7	0.0%	3	0.0%	5	0.0%
mmu-let-7g-5p	9	106081177	106081198	+	Indel	20	22	0.0%	5	0.0%	17	0.0%
mmu-let-7g-5p	9	106081177	106081198	+	edit	1	0	0.0%	0	0.0%	0	0.0%
mmu-let-7g-5p	9	106081177	106081198	+	edit	2	0	0.0%	0	0.0%	0	0.0%
mmu-let-7g-5p	9	106081177	106081198	+	edit	3	0	0.0%	0	0.0%	0	0.0%
mmu-let-7g-5p	9	106081177	106081198	+	edit	4	12	0.0%	10	0.0%	4	0.0%
mmu-let-7g-5p	9	106081177	106081198	+	edit	5	0	0.0%	0	0.0%	0	0.0%
mmu-let-7g-5p	9	106081177	106081198	+	edit	6	432	0.4%	176	0.2%	358	0.7%
mmu-let-7g-5p	9	106081177	106081198	+	edit	7	0	0.0%	0	0.0%	0	0.0%
mmu-let-7g-5p	9	106081177	106081198	+	edit	8	98	0.1%	36	0.0%	14	0.0%
mmu-let-7g-5p	9	106081177	106081198	+	edit	9	0	0.0%	0	0.0%	0	0.0%
mmu-let-7g-5p	9	106081177	106081198	+	edit	10	0	0.0%	0	0.0%	0	0.0%
mmu-let-7g-5p	9	106081177	106081198	+	edit	11	0	0.0%	0	0.0%	0	0.0%
mmu-let-7g-5p	9	106081177	106081198	+	edit	12	186	0.2%	53	0.1%	92	0.2%
mmu-let-7g-5p	9	106081177	106081198	+	edit	13	0	0.0%	0	0.0%	0	0.0%
mmu-let-7g-5p	9	106081177	106081198	+	edit	14	0	0.0%	0	0.0%	0	0.0%
mmu-let-7g-5p	9	106081177	106081198	+	edit	15	291	0.3%	309	0.3%	100	0.2%
mmu-let-7g-5p	9	106081177	106081198	+	edit	16	0	0.0%	0	0.0%	0	0.0%
mmu-let-7g-5p	9	106081177	106081198	+	edit	17	102	0.1%	129	0.1%	41	0.1%
mmu-let-7g-5p	9	106081177	106081198	+	edit	18	1498	1.3%	991	1.1%	393	0.8%
mmu-let-7g-5p	9	106081177	106081198	+	edit	19	562	0.5%	1581	1.7%	229	0.5%
mmu-let-7g-5p	9	106081177	106081198	+	edit	20	0	0.0%	0	0.0%	0	0.0%
mmu-let-7g-5p	9	106081177	106081198	+	edit	21	7	0.0%	6	0.0%	7	0.0%
mmu-let-7g-5p	9	106081177	106081198	+	edit	22	0	0.0%	0	0.0%	0	0.0%
EDITED							3217	2.8%	3298	3.6%	1260	2.5%
CANONICAL							110414	97.2%	87323	96.4%	49598	97.5%
TOTAL							113631		90621		50858	

miRNA	Chr	Start	Stop	Strand	Type	Position	P7 reads	P7%	P10 reads	P10%	P14 reads	P14%
mmu-let-7i-5p	10	122422754	122422775	-	5' Indel	5'	0	0.0%	0	0.0%	3	0.0%
mmu-let-7i-5p	10	122422754	122422775	-	edit	1	0	0.0%	0	0.0%	0	0.0%
mmu-let-7i-5p	10	122422754	122422775	-	edit	2	0	0.0%	0	0.0%	0	0.0%
mmu-let-7i-5p	10	122422754	122422775	-	edit	3	0	0.0%	0	0.0%	0	0.0%
mmu-let-7i-5p	10	122422754	122422775	-	edit	4	4	0.0%	3	0.0%	0	0.0%
mmu-let-7i-5p	10	122422754	122422775	-	edit	5	0	0.0%	0	0.0%	0	0.0%
mmu-let-7i-5p	10	122422754	122422775	-	edit	6	0	0.0%	0	0.0%	0	0.0%
mmu-let-7i-5p	10	122422754	122422775	-	edit	7	0	0.0%	0	0.0%	0	0.0%
mmu-let-7i-5p	10	122422754	122422775	-	edit	8	0	0.0%	0	0.0%	0	0.0%
mmu-let-7i-5p	10	122422754	122422775	-	edit	9	0	0.0%	0	0.0%	0	0.0%
mmu-let-7i-5p	10	122422754	122422775	-	edit	10	0	0.0%	0	0.0%	0	0.0%
mmu-let-7i-5p	10	122422754	122422775	-	edit	11	0	0.0%	0	0.0%	0	0.0%
mmu-let-7i-5p	10	122422754	122422775	-	edit	12	0	0.0%	0	0.0%	0	0.0%
mmu-let-7i-5p	10	122422754	122422775	-	edit	13	0	0.0%	0	0.0%	0	0.0%
mmu-let-7i-5p	10	122422754	122422775	-	edit	14	0	0.0%	0	0.0%	0	0.0%
mmu-let-7i-5p	10	122422754	122422775	-	edit	15	84	0.1%	103	0.2%	27	0.1%
mmu-let-7i-5p	10	122422754	122422775	-	edit	16	46	0.1%	39	0.1%	23	0.1%
mmu-let-7i-5p	10	122422754	122422775	-	edit	17	107	0.2%	112	0.2%	34	0.1%
mmu-let-7i-5p	10	122422754	122422775	-	edit	18	0	0.0%	4	0.0%	0	0.0%
mmu-let-7i-5p	10	122422754	122422775	-	edit	19	107	0.2%	145	0.3%	34	0.1%
mmu-let-7i-5p	10	122422754	122422775	-	edit	20	0	0.0%	0	0.0%	0	0.0%
mmu-let-7i-5p	10	122422754	122422775	-	edit	21	0	0.0%	0	0.0%	0	0.0%
mmu-let-7i-5p	10	122422754	122422775	-	edit	22	0	0.0%	0	0.0%	0	0.0%
EDITED							347	0.6%	406	0.9%	120	0.5%
CANONICAL							55670	99.4%	46889	99.1%	25130	99.5%
TOTAL							56017		47295		25250	

miRNA	Chr	Start	Stop	Strand	Type	Position	P7 reads	P7%	P10 reads	P10%	P14 reads	P14%
mmu-mir-1-2-5p	18	10785483	10785504	-	edit	1	0	0.0%	0	0.0%	0	0.0%
mmu-mir-1-2-5p	18	10785483	10785504	-	edit	2	0	0.0%	0	0.0%	0	0.0%
mmu-mir-1-2-5p	18	10785483	10785504	-	edit	3	0	0.0%	0	0.0%	0	0.0%
mmu-mir-1-2-5p	18	10785483	10785504	-	edit	4	8	0.1%	8	0.1%	4	0.1%
mmu-mir-1-2-5p	18	10785483	10785504	-	edit	5	0	0.0%	0	0.0%	0	0.0%
mmu-mir-1-2-5p	18	10785483	10785504	-	edit	6	0	0.0%	0	0.0%	0	0.0%
mmu-mir-1-2-5p	18	10785483	10785504	-	edit	7	0	0.0%	0	0.0%	0	0.0%
mmu-mir-1-2-5p	18	10785483	10785504	-	edit	8	52	0.4%	11	0.1%	30	0.4%
mmu-mir-1-2-5p	18	10785483	10785504	-	edit	9	0	0.0%	0	0.0%	0	0.0%
mmu-mir-1-2-5p	18	10785483	10785504	-	edit	10	0	0.0%	0	0.0%	0	0.0%
mmu-mir-1-2-5p	18	10785483	10785504	-	edit	11	0	0.0%	0	0.0%	0	0.0%
mmu-mir-1-2-5p	18	10785483	10785504	-	edit	12	0	0.0%	0	0.0%	0	0.0%
mmu-mir-1-2-5p	18	10785483	10785504	-	edit	13	0	0.0%	0	0.0%	0	0.0%
mmu-mir-1-2-5p	18	10785483	10785504	-	edit	14	0	0.0%	0	0.0%	0	0.0%
mmu-mir-1-2-5p	18	10785483	10785504	-	edit	15	5	0.0%	0	0.0%	0	0.0%
mmu-mir-1-2-5p	18	10785483	10785504	-	edit	16	6	0.0%	0	0.0%	4	0.1%
mmu-mir-1-2-5p	18	10785483	10785504	-	edit	17	16	0.1%	60	0.6%	6	0.1%
mmu-mir-1-2-5p	18	10785483	10785504	-	edit	18	0	0.0%	0	0.0%	0	0.0%
mmu-mir-1-2-5p	18	10785483	10785504	-	edit	19	25	0.2%	11	0.1%	9	0.1%
mmu-mir-1-2-5p	18	10785483	10785504	-	edit	20	0	0.0%	0	0.0%	0	0.0%
mmu-mir-1-2-5p	18	10785483	10785504	-	edit	21	0	0.0%	0	0.0%	0	0.0%
mmu-mir-1-2-5p	18	10785483	10785504	-	edit	22	0	0.0%	0	0.0%	0	0.0%
EDITED							112	0.8%	90	0.9%	53	0.7%
CANONICAL							14204	99.2%	9553	99.1%	7240	99.3%
TOTAL							14316		9643		7293	

miRNA	Chr	Start	Stop	Strand	Type	Position	P7 reads	P7%	P10 reads	P10%	P14 reads	P14%
mmu-mir-7a-2-5p	7	86033181	86033203	+	edit	1	0	0.0%	0	0.0%	0	0.0%
mmu-mir-7a-2-5p	7	86033181	86033203	+	edit	2	0	0.0%	0	0.0%	0	0.0%
mmu-mir-7a-2-5p	7	86033181	86033203	+	edit	3	0	0.0%	0	0.0%	0	0.0%
mmu-mir-7a-2-5p	7	86033181	86033203	+	edit	4	0	0.0%	0	0.0%	0	0.0%
mmu-mir-7a-2-5p	7	86033181	86033203	+	edit	5	0	0.0%	0	0.0%	0	0.0%
mmu-mir-7a-2-5p	7	86033181	86033203	+	edit	6	0	0.0%	0	0.0%	0	0.0%
mmu-mir-7a-2-5p	7	86033181	86033203	+	edit	7	0	0.0%	0	0.0%	0	0.0%
mmu-mir-7a-2-5p	7	86033181	86033203	+	edit	8	0	0.0%	0	0.0%	0	0.0%
mmu-mir-7a-2-5p	7	86033181	86033203	+	edit	9	0	0.0%	0	0.0%	0	0.0%
mmu-mir-7a-2-5p	7	86033181	86033203	+	edit	10	0	0.0%	0	0.0%	0	0.0%
mmu-mir-7a-2-5p	7	86033181	86033203	+	edit	11	0	0.0%	0	0.0%	0	0.0%
mmu-mir-7a-2-5p	7	86033181	86033203	+	edit	12	0	0.0%	0	0.0%	0	0.0%
mmu-mir-7a-2-5p	7	86033181	86033203	+	edit	13	0	0.0%	0	0.0%	0	0.0%
mmu-mir-7a-2-5p	7	86033181	86033203	+	edit	14	0	0.0%	6	1.2%	0	0.0%
mmu-mir-7a-2-5p	7	86033181	86033203	+	edit	15	0	0.0%	0	0.0%	0	0.0%
mmu-mir-7a-2-5p	7	86033181	86033203	+	edit	16	0	0.0%	0	0.0%	0	0.0%
mmu-mir-7a-2-5p	7	86033181	86033203	+	edit	17	0	0.0%	0	0.0%	0	0.0%
mmu-mir-7a-2-5p	7	86033181	86033203	+	edit	18	0	0.0%	0	0.0%	0	0.0%
mmu-mir-7a-2-5p	7	86033181	86033203	+	edit	19	0	0.0%	0	0.0%	0	0.0%
mmu-mir-7a-2-5p	7	86033181	86033203	+	edit	20	0	0.0%	0	0.0%	0	0.0%
mmu-mir-7a-2-5p	7	86033181	86033203	+	edit	21	0	0.0%	0	0.0%	0	0.0%
mmu-mir-7a-2-5p	7	86033181	86033203	+	edit	22	0	0.0%	0	0.0%	0	0.0%
EDITED							0	0.0%	6	1.2%	0	0.0%
CANONICAL							302	100.0%	510	98.8%	238	100.0%
TOTAL							302		516		238	

miRNA	Chr	Start	Stop	Strand	Type	Position	P7 reads	P7%	P10 reads	P10%	P14 reads	P14%
mmu-mir-10a-5p	11	96178500	96178522	+	edit	1	0	0.0%	0	0.0%	0	0.0%
mmu-mir-10a-5p	11	96178500	96178522	+	edit	2	0	0.0%	0	0.0%	0	0.0%
mmu-mir-10a-5p	11	96178500	96178522	+	edit	3	0	0.0%	0	0.0%	0	0.0%
mmu-mir-10a-5p	11	96178500	96178522	+	edit	4	6	0.1%	0	0.0%	0	0.0%
mmu-mir-10a-5p	11	96178500	96178522	+	edit	5	0	0.0%	0	0.0%	0	0.0%
mmu-mir-10a-5p	11	96178500	96178522	+	edit	6	4	0.1%	0	0.0%	0	0.0%
mmu-mir-10a-5p	11	96178500	96178522	+	edit	7	0	0.0%	0	0.0%	0	0.0%
mmu-mir-10a-5p	11	96178500	96178522	+	edit	8	9	0.2%	0	0.0%	0	0.0%
mmu-mir-10a-5p	11	96178500	96178522	+	edit	9	0	0.0%	0	0.0%	0	0.0%
mmu-mir-10a-5p	11	96178500	96178522	+	edit	10	0	0.0%	0	0.0%	0	0.0%
mmu-mir-10a-5p	11	96178500	96178522	+	edit	11	0	0.0%	0	0.0%	0	0.0%
mmu-mir-10a-5p	11	96178500	96178522	+	edit	12	4	0.1%	0	0.0%	0	0.0%
mmu-mir-10a-5p	11	96178500	96178522	+	edit	13	4	0.1%	0	0.0%	0	0.0%
mmu-mir-10a-5p	11	96178500	96178522	+	edit	14	0	0.0%	0	0.0%	0	0.0%
mmu-mir-10a-5p	11	96178500	96178522	+	edit	15	0	0.0%	0	0.0%	0	0.0%
mmu-mir-10a-5p	11	96178500	96178522	+	edit	16	0	0.0%	4	0.5%	0	0.0%
mmu-mir-10a-5p	11	96178500	96178522	+	edit	17	0	0.0%	0	0.0%	0	0.0%
mmu-mir-10a-5p	11	96178500	96178522	+	edit	18	0	0.0%	0	0.0%	0	0.0%
mmu-mir-10a-5p	11	96178500	96178522	+	edit	19	7	0.2%	6	0.7%	0	0.0%
mmu-mir-10a-5p	11	96178500	96178522	+	edit	20	0	0.0%	0	0.0%	0	0.0%
mmu-mir-10a-5p	11	96178500	96178522	+	edit	21	4	0.1%	0	0.0%	0	0.0%
mmu-mir-10a-5p	11	96178500	96178522	+	edit	22	0	0.0%	0	0.0%	0	0.0%
EDITED							38	0.8%	10	1.2%	0	0.0%
CANONICAL							4592	99.2%	821	98.8%	803	100.0%
TOTAL							4630		831		803	

miRNA	Chr	Start	Stop	Strand	Type	Position	P7 reads	P7%	P10 reads	P10%	P14 reads	P14%
mmu-mir-10b-5p	2	74564131	74564153	+	3' Edit	3'	7	0.2%	0	0.0%	3	0.4%
mmu-mir-10b-5p	2	74564131	74564153	+	Indel	22	5	0.2%	8	0.6%	0	0.0%
mmu-mir-10b-5p	2	74564131	74564153	+	edit	1	0	0.0%	0	0.0%	0	0.0%
mmu-mir-10b-5p	2	74564131	74564153	+	edit	2	0	0.0%	0	0.0%	0	0.0%
mmu-mir-10b-5p	2	74564131	74564153	+	edit	3	0	0.0%	0	0.0%	0	0.0%
mmu-mir-10b-5p	2	74564131	74564153	+	edit	4	3	0.1%	0	0.0%	0	0.0%
mmu-mir-10b-5p	2	74564131	74564153	+	edit	5	0	0.0%	0	0.0%	0	0.0%
mmu-mir-10b-5p	2	74564131	74564153	+	edit	6	0	0.0%	0	0.0%	0	0.0%
mmu-mir-10b-5p	2	74564131	74564153	+	edit	7	0	0.0%	0	0.0%	0	0.0%
mmu-mir-10b-5p	2	74564131	74564153	+	edit	8	16	0.5%	0	0.0%	0	0.0%
mmu-mir-10b-5p	2	74564131	74564153	+	edit	9	0	0.0%	0	0.0%	0	0.0%
mmu-mir-10b-5p	2	74564131	74564153	+	edit	10	0	0.0%	0	0.0%	0	0.0%
mmu-mir-10b-5p	2	74564131	74564153	+	edit	11	0	0.0%	0	0.0%	0	0.0%
mmu-mir-10b-5p	2	74564131	74564153	+	edit	12	0	0.0%	0	0.0%	3	0.4%
mmu-mir-10b-5p	2	74564131	74564153	+	edit	13	0	0.0%	0	0.0%	0	0.0%
mmu-mir-10b-5p	2	74564131	74564153	+	edit	14	0	0.0%	0	0.0%	0	0.0%
mmu-mir-10b-5p	2	74564131	74564153	+	edit	15	0	0.0%	0	0.0%	0	0.0%
mmu-mir-10b-5p	2	74564131	74564153	+	edit	16	3	0.1%	10	0.8%	0	0.0%
mmu-mir-10b-5p	2	74564131	74564153	+	edit	17	0	0.0%	0	0.0%	0	0.0%
mmu-mir-10b-5p	2	74564131	74564153	+	edit	18	0	0.0%	0	0.0%	0	0.0%
mmu-mir-10b-5p	2	74564131	74564153	+	edit	19	11	0.3%	3	0.2%	0	0.0%
mmu-mir-10b-5p	2	74564131	74564153	+	edit	20	0	0.0%	0	0.0%	0	0.0%
mmu-mir-10b-5p	2	74564131	74564153	+	edit	21	0	0.0%	0	0.0%	0	0.0%
mmu-mir-10b-5p	2	74564131	74564153	+	edit	22	0	0.0%	0	0.0%	0	0.0%
mmu-mir-10b-5p	2	74564131	74564153	+	edit	23	0	0.0%	0	0.0%	3	0.4%
EDITED							45	1.4%	21	1.6%	9	1.3%
CANONICAL							3215	98.6%	1301	98.4%	689	98.7%
TOTAL							3260		1322		698	

miRNA	Chr	Start	Stop	Strand	Type	Position	P7 reads	P7%	P10 reads	P10%	P14 reads	P14%
mmu-mir-16-2-5p	3	68813840	68813861	+	Indel	22	3	0.7%	4	1.0%	0	0.0%
mmu-mir-16-2-5p	3	68813840	68813861	+	edit	1	0	0.0%	0	0.0%	0	0.0%
mmu-mir-16-2-5p	3	68813840	68813861	+	edit	2	0	0.0%	0	0.0%	0	0.0%
mmu-mir-16-2-5p	3	68813840	68813861	+	edit	3	0	0.0%	0	0.0%	0	0.0%
mmu-mir-16-2-5p	3	68813840	68813861	+	edit	4	0	0.0%	0	0.0%	0	0.0%
mmu-mir-16-2-5p	3	68813840	68813861	+	edit	5	0	0.0%	0	0.0%	0	0.0%
mmu-mir-16-2-5p	3	68813840	68813861	+	edit	6	0	0.0%	0	0.0%	0	0.0%
mmu-mir-16-2-5p	3	68813840	68813861	+	edit	7	0	0.0%	0	0.0%	0	0.0%
mmu-mir-16-2-5p	3	68813840	68813861	+	edit	8	0	0.0%	0	0.0%	0	0.0%
mmu-mir-16-2-5p	3	68813840	68813861	+	edit	9	0	0.0%	0	0.0%	0	0.0%
mmu-mir-16-2-5p	3	68813840	68813861	+	edit	10	0	0.0%	0	0.0%	0	0.0%
mmu-mir-16-2-5p	3	68813840	68813861	+	edit	11	0	0.0%	0	0.0%	0	0.0%
mmu-mir-16-2-5p	3	68813840	68813861	+	edit	12	0	0.0%	0	0.0%	0	0.0%
mmu-mir-16-2-5p	3	68813840	68813861	+	edit	13	0	0.0%	0	0.0%	0	0.0%
mmu-mir-16-2-5p	3	68813840	68813861	+	edit	14	0	0.0%	0	0.0%	0	0.0%
mmu-mir-16-2-5p	3	68813840	68813861	+	edit	15	0	0.0%	0	0.0%	0	0.0%
mmu-mir-16-2-5p	3	68813840	68813861	+	edit	16	0	0.0%	0	0.0%	0	0.0%
mmu-mir-16-2-5p	3	68813840	68813861	+	edit	17	0	0.0%	0	0.0%	0	0.0%
mmu-mir-16-2-5p	3	68813840	68813861	+	edit	18	0	0.0%	0	0.0%	0	0.0%
mmu-mir-16-2-5p	3	68813840	68813861	+	edit	19	0	0.0%	0	0.0%	0	0.0%
mmu-mir-16-2-5p	3	68813840	68813861	+	edit	20	0	0.0%	0	0.0%	0	0.0%
mmu-mir-16-2-5p	3	68813840	68813861	+	edit	21	0	0.0%	0	0.0%	4	1.8%
mmu-mir-16-2-5p	3	68813840	68813861	+	edit	22	0	0.0%	0	0.0%	0	0.0%
EDITED							3	0.7%	4	1.0%	4	1.8%
CANONICAL							406	99.3%	393	99.0%	222	98.2%
TOTAL							409		397		226	

miRNA	Chr	Start	Stop	Strand	Type	Position	P7 reads	P7%	P10 reads	P10%	P14 reads	P14%
mmu-mir-17-3p	14	115442943	115442964	+	3' Edit	3'	10	1.2%	0	0.0%	3	1.0%
mmu-mir-17-3p	14	115442943	115442964	+	Indel	22	3	0.4%	0	0.0%	0	0.0%
mmu-mir-17-3p	14	115442943	115442964	+	edit	1	0	0.0%	0	0.0%	0	0.0%
mmu-mir-17-3p	14	115442943	115442964	+	edit	2	0	0.0%	0	0.0%	0	0.0%
mmu-mir-17-3p	14	115442943	115442964	+	edit	3	0	0.0%	0	0.0%	0	0.0%
mmu-mir-17-3p	14	115442943	115442964	+	edit	4	0	0.0%	0	0.0%	0	0.0%
mmu-mir-17-3p	14	115442943	115442964	+	edit	5	0	0.0%	0	0.0%	0	0.0%
mmu-mir-17-3p	14	115442943	115442964	+	edit	6	0	0.0%	0	0.0%	0	0.0%
mmu-mir-17-3p	14	115442943	115442964	+	edit	7	0	0.0%	0	0.0%	0	0.0%
mmu-mir-17-3p	14	115442943	115442964	+	edit	8	3	0.4%	0	0.0%	0	0.0%
mmu-mir-17-3p	14	115442943	115442964	+	edit	9	0	0.0%	0	0.0%	0	0.0%
mmu-mir-17-3p	14	115442943	115442964	+	edit	10	0	0.0%	0	0.0%	0	0.0%
mmu-mir-17-3p	14	115442943	115442964	+	edit	11	0	0.0%	0	0.0%	0	0.0%
mmu-mir-17-3p	14	115442943	115442964	+	edit	12	0	0.0%	0	0.0%	0	0.0%
mmu-mir-17-3p	14	115442943	115442964	+	edit	13	0	0.0%	0	0.0%	0	0.0%
mmu-mir-17-3p	14	115442943	115442964	+	edit	14	0	0.0%	0	0.0%	0	0.0%
mmu-mir-17-3p	14	115442943	115442964	+	edit	15	0	0.0%	4	0.7%	0	0.0%
mmu-mir-17-3p	14	115442943	115442964	+	edit	16	0	0.0%	0	0.0%	0	0.0%
mmu-mir-17-3p	14	115442943	115442964	+	edit	17	0	0.0%	0	0.0%	0	0.0%
mmu-mir-17-3p	14	115442943	115442964	+	edit	18	0	0.0%	0	0.0%	0	0.0%
mmu-mir-17-3p	14	115442943	115442964	+	edit	19	0	0.0%	0	0.0%	0	0.0%
mmu-mir-17-3p	14	115442943	115442964	+	edit	20	0	0.0%	0	0.0%	0	0.0%
mmu-mir-17-3p	14	115442943	115442964	+	edit	21	0	0.0%	0	0.0%	0	0.0%
mmu-mir-17-3p	14	115442943	115442964	+	edit	22	0	0.0%	0	0.0%	0	0.0%
EDITED							16	2.0%	4	0.7%	3	1.0%
CANONICAL							792	98.0%	464	99.3%	293	99.0%
TOTAL							808		468		296	

miRNA	Chr	Start	Stop	Strand	Type	Position	P7 reads	P7%	P10 reads	P10%	P14 reads	P14%
mmu-mir-21-5p	11	86397622	86397643	-	5' Edit	5'	17	0.3%	0	0.0%	10	0.3%
mmu-mir-21-5p	11	86397622	86397643	-	edit	1	0	0.0%	0	0.0%	0	0.0%
mmu-mir-21-5p	11	86397622	86397643	-	edit	2	0	0.0%	0	0.0%	0	0.0%
mmu-mir-21-5p	11	86397622	86397643	-	edit	3	0	0.0%	0	0.0%	0	0.0%
mmu-mir-21-5p	11	86397622	86397643	-	edit	4	0	0.0%	0	0.0%	0	0.0%
mmu-mir-21-5p	11	86397622	86397643	-	edit	5	0	0.0%	0	0.0%	0	0.0%
mmu-mir-21-5p	11	86397622	86397643	-	edit	6	0	0.0%	0	0.0%	0	0.0%
mmu-mir-21-5p	11	86397622	86397643	-	edit	7	0	0.0%	0	0.0%	0	0.0%
mmu-mir-21-5p	11	86397622	86397643	-	edit	8	7	0.1%	0	0.0%	0	0.0%
mmu-mir-21-5p	11	86397622	86397643	-	edit	9	3	0.0%	0	0.0%	0	0.0%
mmu-mir-21-5p	11	86397622	86397643	-	edit	10	0	0.0%	0	0.0%	0	0.0%
mmu-mir-21-5p	11	86397622	86397643	-	edit	11	0	0.0%	0	0.0%	0	0.0%
mmu-mir-21-5p	11	86397622	86397643	-	edit	12	0	0.0%	0	0.0%	0	0.0%
mmu-mir-21-5p	11	86397622	86397643	-	edit	13	0	0.0%	0	0.0%	0	0.0%
mmu-mir-21-5p	11	86397622	86397643	-	edit	14	0	0.0%	0	0.0%	0	0.0%
mmu-mir-21-5p	11	86397622	86397643	-	edit	15	0	0.0%	5	0.1%	0	0.0%
mmu-mir-21-5p	11	86397622	86397643	-	edit	16	0	0.0%	27	0.5%	3	0.1%
mmu-mir-21-5p	11	86397622	86397643	-	edit	17	13	0.2%	0	0.0%	5	0.1%
mmu-mir-21-5p	11	86397622	86397643	-	edit	18	5	0.1%	0	0.0%	3	0.1%
mmu-mir-21-5p	11	86397622	86397643	-	edit	19	37	0.6%	8	0.2%	14	0.4%
mmu-mir-21-5p	11	86397622	86397643	-	edit	20	0	0.0%	0	0.0%	0	0.0%
mmu-mir-21-5p	11	86397622	86397643	-	edit	21	7	0.1%	0	0.0%	0	0.0%
mmu-mir-21-5p	11	86397622	86397643	-	edit	22	0	0.0%	0	0.0%	0	0.0%
EDITED							89	1.3%	40	0.8%	34	1.1%
CANONICAL							6659	98.7%	4952	99.2%	3182	98.9%
TOTAL							6748		4992		3216	

miRNA	Chr	Start	Stop	Strand	Type	Position	P7 reads	P7%	P10 reads	P10%	P14 reads	P14%
mmu-mir-22-3p	11	75277274	75277295	+	edit	1	0	0.0%	0	0.0%	0	0.0%
mmu-mir-22-3p	11	75277274	75277295	+	edit	2	0	0.0%	0	0.0%	0	0.0%
mmu-mir-22-3p	11	75277274	75277295	+	edit	3	0	0.0%	0	0.0%	0	0.0%
mmu-mir-22-3p	11	75277274	75277295	+	edit	4	0	0.0%	0	0.0%	0	0.0%
mmu-mir-22-3p	11	75277274	75277295	+	edit	5	0	0.0%	0	0.0%	0	0.0%
mmu-mir-22-3p	11	75277274	75277295	+	edit	6	0	0.0%	0	0.0%	0	0.0%
mmu-mir-22-3p	11	75277274	75277295	+	edit	7	0	0.0%	0	0.0%	0	0.0%
mmu-mir-22-3p	11	75277274	75277295	+	edit	8	0	0.0%	3	0.3%	0	0.0%
mmu-mir-22-3p	11	75277274	75277295	+	edit	9	0	0.0%	0	0.0%	0	0.0%
mmu-mir-22-3p	11	75277274	75277295	+	edit	10	0	0.0%	0	0.0%	0	0.0%
mmu-mir-22-3p	11	75277274	75277295	+	edit	11	0	0.0%	0	0.0%	0	0.0%
mmu-mir-22-3p	11	75277274	75277295	+	edit	12	0	0.0%	0	0.0%	0	0.0%
mmu-mir-22-3p	11	75277274	75277295	+	edit	13	0	0.0%	0	0.0%	0	0.0%
mmu-mir-22-3p	11	75277274	75277295	+	edit	14	0	0.0%	0	0.0%	0	0.0%
mmu-mir-22-3p	11	75277274	75277295	+	edit	15	0	0.0%	4	0.4%	0	0.0%
mmu-mir-22-3p	11	75277274	75277295	+	edit	16	0	0.0%	0	0.0%	0	0.0%
mmu-mir-22-3p	11	75277274	75277295	+	edit	17	0	0.0%	0	0.0%	0	0.0%
mmu-mir-22-3p	11	75277274	75277295	+	edit	18	0	0.0%	0	0.0%	0	0.0%
mmu-mir-22-3p	11	75277274	75277295	+	edit	19	6	0.5%	8	0.8%	6	1.0%
mmu-mir-22-3p	11	75277274	75277295	+	edit	20	0	0.0%	0	0.0%	0	0.0%
mmu-mir-22-3p	11	75277274	75277295	+	edit	21	0	0.0%	0	0.0%	0	0.0%
mmu-mir-22-3p	11	75277274	75277295	+	edit	22	0	0.0%	0	0.0%	0	0.0%
EDITED							6	0.5%	15	1.5%	6	1.0%
CANONICAL							1217	99.5%	992	98.5%	600	99.0%
TOTAL							1223		1007		606	

miRNA	Chr	Start	Stop	Strand	Type	Position	P7 reads	P7%	P10 reads	P10%	P14 reads	P14%
mmu-mir-24-1-3p	13	63402559	63402580	+	3' Edit	3'	5	0.4%	0	0.0%	0	0.0%
mmu-mir-24-1-3p	13	63402559	63402580	+	edit	1	0	0.0%	0	0.0%	0	0.0%
mmu-mir-24-1-3p	13	63402559	63402580	+	edit	2	0	0.0%	0	0.0%	0	0.0%
mmu-mir-24-1-3p	13	63402559	63402580	+	edit	3	0	0.0%	0	0.0%	0	0.0%
mmu-mir-24-1-3p	13	63402559	63402580	+	edit	4	0	0.0%	0	0.0%	0	0.0%
mmu-mir-24-1-3p	13	63402559	63402580	+	edit	5	0	0.0%	0	0.0%	0	0.0%
mmu-mir-24-1-3p	13	63402559	63402580	+	edit	6	0	0.0%	0	0.0%	0	0.0%
mmu-mir-24-1-3p	13	63402559	63402580	+	edit	7	0	0.0%	0	0.0%	0	0.0%
mmu-mir-24-1-3p	13	63402559	63402580	+	edit	8	0	0.0%	0	0.0%	0	0.0%
mmu-mir-24-1-3p	13	63402559	63402580	+	edit	9	0	0.0%	0	0.0%	0	0.0%
mmu-mir-24-1-3p	13	63402559	63402580	+	edit	10	0	0.0%	0	0.0%	0	0.0%
mmu-mir-24-1-3p	13	63402559	63402580	+	edit	11	0	0.0%	0	0.0%	0	0.0%
mmu-mir-24-1-3p	13	63402559	63402580	+	edit	12	0	0.0%	0	0.0%	0	0.0%
mmu-mir-24-1-3p	13	63402559	63402580	+	edit	13	0	0.0%	0	0.0%	0	0.0%
mmu-mir-24-1-3p	13	63402559	63402580	+	edit	14	0	0.0%	0	0.0%	0	0.0%
mmu-mir-24-1-3p	13	63402559	63402580	+	edit	15	0	0.0%	3	0.3%	0	0.0%
mmu-mir-24-1-3p	13	63402559	63402580	+	edit	16	0	0.0%	0	0.0%	0	0.0%
mmu-mir-24-1-3p	13	63402559	63402580	+	edit	17	0	0.0%	0	0.0%	0	0.0%
mmu-mir-24-1-3p	13	63402559	63402580	+	edit	18	0	0.0%	0	0.0%	0	0.0%
mmu-mir-24-1-3p	13	63402559	63402580	+	edit	19	0	0.0%	5	0.5%	0	0.0%
mmu-mir-24-1-3p	13	63402559	63402580	+	edit	20	0	0.0%	0	0.0%	0	0.0%
mmu-mir-24-1-3p	13	63402559	63402580	+	edit	21	0	0.0%	0	0.0%	0	0.0%
mmu-mir-24-1-3p	13	63402559	63402580	+	edit	22	0	0.0%	0	0.0%	0	0.0%
EDITED							5	0.4%	8	0.9%	0	0.0%
CANONICAL							1248	99.6%	912	99.1%	398	100.0%
TOTAL							1253		920		398	

miRNA	Chr	Start	Stop	Strand	Type	Position	P7 reads	P7%	P10 reads	P10%	P14 reads	P14%
mmu-mir-24-2-3p	8	86732774	86732795	+	3' Edit	3'	4	0.3%	0	0.0%	0	0.0%
mmu-mir-24-2-3p	8	86732774	86732795	+	edit	1	0	0.0%	0	0.0%	0	0.0%
mmu-mir-24-2-3p	8	86732774	86732795	+	edit	2	0	0.0%	0	0.0%	0	0.0%
mmu-mir-24-2-3p	8	86732774	86732795	+	edit	3	0	0.0%	0	0.0%	0	0.0%
mmu-mir-24-2-3p	8	86732774	86732795	+	edit	4	0	0.0%	0	0.0%	0	0.0%
mmu-mir-24-2-3p	8	86732774	86732795	+	edit	5	0	0.0%	0	0.0%	0	0.0%
mmu-mir-24-2-3p	8	86732774	86732795	+	edit	6	0	0.0%	0	0.0%	0	0.0%
mmu-mir-24-2-3p	8	86732774	86732795	+	edit	7	0	0.0%	0	0.0%	0	0.0%
mmu-mir-24-2-3p	8	86732774	86732795	+	edit	8	0	0.0%	0	0.0%	0	0.0%
mmu-mir-24-2-3p	8	86732774	86732795	+	edit	9	0	0.0%	0	0.0%	0	0.0%
mmu-mir-24-2-3p	8	86732774	86732795	+	edit	10	0	0.0%	0	0.0%	0	0.0%
mmu-mir-24-2-3p	8	86732774	86732795	+	edit	11	0	0.0%	0	0.0%	0	0.0%
mmu-mir-24-2-3p	8	86732774	86732795	+	edit	12	0	0.0%	0	0.0%	0	0.0%
mmu-mir-24-2-3p	8	86732774	86732795	+	edit	13	0	0.0%	0	0.0%	0	0.0%
mmu-mir-24-2-3p	8	86732774	86732795	+	edit	14	0	0.0%	0	0.0%	0	0.0%
mmu-mir-24-2-3p	8	86732774	86732795	+	edit	15	0	0.0%	3	0.3%	0	0.0%
mmu-mir-24-2-3p	8	86732774	86732795	+	edit	16	0	0.0%	0	0.0%	0	0.0%
mmu-mir-24-2-3p	8	86732774	86732795	+	edit	17	0	0.0%	0	0.0%	0	0.0%
mmu-mir-24-2-3p	8	86732774	86732795	+	edit	18	0	0.0%	0	0.0%	0	0.0%
mmu-mir-24-2-3p	8	86732774	86732795	+	edit	19	0	0.0%	5	0.5%	0	0.0%
mmu-mir-24-2-3p	8	86732774	86732795	+	edit	20	0	0.0%	0	0.0%	0	0.0%
mmu-mir-24-2-3p	8	86732774	86732795	+	edit	21	0	0.0%	0	0.0%	0	0.0%
mmu-mir-24-2-3p	8	86732774	86732795	+	edit	22	0	0.0%	0	0.0%	0	0.0%
EDITED							4	0.3%	8	0.9%	0	0.0%
CANONICAL							1247	99.7%	911	99.1%	397	100.0%
TOTAL							1251		919		397	

miRNA	Chr	Start	Stop	Strand	Type	Position	P7 reads	P7%	P10 reads	P10%	P14 reads	P14%
mmu-mir-25-5p	5	138606560	138606581	-	5' Edit	5'	618	1.9%	0	0.0%	405	3.0%
mmu-mir-25-5p	5	138606560	138606581	-	edit	1	0	0.0%	0	0.0%	0	0.0%
mmu-mir-25-5p	5	138606560	138606581	-	edit	2	0	0.0%	0	0.0%	0	0.0%
mmu-mir-25-5p	5	138606560	138606581	-	edit	3	0	0.0%	0	0.0%	0	0.0%
mmu-mir-25-5p	5	138606560	138606581	-	edit	4	36	0.1%	13	0.1%	9	0.1%
mmu-mir-25-5p	5	138606560	138606581	-	edit	5	0	0.0%	0	0.0%	0	0.0%
mmu-mir-25-5p	5	138606560	138606581	-	edit	6	0	0.0%	0	0.0%	0	0.0%
mmu-mir-25-5p	5	138606560	138606581	-	edit	7	0	0.0%	0	0.0%	0	0.0%
mmu-mir-25-5p	5	138606560	138606581	-	edit	8	28	0.1%	3	0.0%	10	0.1%
mmu-mir-25-5p	5	138606560	138606581	-	edit	9	0	0.0%	0	0.0%	0	0.0%
mmu-mir-25-5p	5	138606560	138606581	-	edit	10	0	0.0%	0	0.0%	0	0.0%
mmu-mir-25-5p	5	138606560	138606581	-	edit	11	0	0.0%	0	0.0%	0	0.0%
mmu-mir-25-5p	5	138606560	138606581	-	edit	12	0	0.0%	0	0.0%	0	0.0%
mmu-mir-25-5p	5	138606560	138606581	-	edit	13	0	0.0%	0	0.0%	0	0.0%
mmu-mir-25-5p	5	138606560	138606581	-	edit	14	0	0.0%	0	0.0%	0	0.0%
mmu-mir-25-5p	5	138606560	138606581	-	edit	15	57	0.2%	29	0.2%	43	0.3%
mmu-mir-25-5p	5	138606560	138606581	-	edit	16	0	0.0%	0	0.0%	0	0.0%
mmu-mir-25-5p	5	138606560	138606581	-	edit	17	71	0.2%	43	0.3%	26	0.2%
mmu-mir-25-5p	5	138606560	138606581	-	edit	18	0	0.0%	0	0.0%	0	0.0%
mmu-mir-25-5p	5	138606560	138606581	-	edit	19	645	2.0%	266	1.9%	385	2.9%
mmu-mir-25-5p	5	138606560	138606581	-	edit	20	0	0.0%	0	0.0%	0	0.0%
mmu-mir-25-5p	5	138606560	138606581	-	edit	21	0	0.0%	0	0.0%	0	0.0%
mmu-mir-25-5p	5	138606560	138606581	-	edit	22	0	0.0%	0	0.0%	0	0.0%
EDITED							1455	4.5%	354	2.5%	877	6.6%
CANONICAL							30958	95.5%	13809	97.5%	12435	93.4%
TOTAL							32413		14163		13312	

miRNA	Chr	Start	Stop	Strand	Type	Position	P7 reads	P7%	P10 reads	P10%	P14 reads	P14%
mmu-mir-26a-1-5p	9	118940929	118940950	+	3' Edit	3'	6	0.1%	0	0.0%	0	0.0%
mmu-mir-26a-1-5p	9	118940929	118940950	+	edit	1	0	0.0%	0	0.0%	0	0.0%
mmu-mir-26a-1-5p	9	118940929	118940950	+	edit	2	0	0.0%	0	0.0%	0	0.0%
mmu-mir-26a-1-5p	9	118940929	118940950	+	edit	3	0	0.0%	0	0.0%	0	0.0%
mmu-mir-26a-1-5p	9	118940929	118940950	+	edit	4	5	0.1%	0	0.0%	0	0.0%
mmu-mir-26a-1-5p	9	118940929	118940950	+	edit	5	0	0.0%	0	0.0%	0	0.0%
mmu-mir-26a-1-5p	9	118940929	118940950	+	edit	6	0	0.0%	0	0.0%	0	0.0%
mmu-mir-26a-1-5p	9	118940929	118940950	+	edit	7	0	0.0%	0	0.0%	0	0.0%
mmu-mir-26a-1-5p	9	118940929	118940950	+	edit	8	5	0.1%	0	0.0%	0	0.0%
mmu-mir-26a-1-5p	9	118940929	118940950	+	edit	9	0	0.0%	0	0.0%	0	0.0%
mmu-mir-26a-1-5p	9	118940929	118940950	+	edit	10	0	0.0%	0	0.0%	0	0.0%
mmu-mir-26a-1-5p	9	118940929	118940950	+	edit	11	0	0.0%	0	0.0%	0	0.0%
mmu-mir-26a-1-5p	9	118940929	118940950	+	edit	12	0	0.0%	0	0.0%	0	0.0%
mmu-mir-26a-1-5p	9	118940929	118940950	+	edit	13	0	0.0%	0	0.0%	0	0.0%
mmu-mir-26a-1-5p	9	118940929	118940950	+	edit	14	0	0.0%	0	0.0%	0	0.0%
mmu-mir-26a-1-5p	9	118940929	118940950	+	edit	15	8	0.1%	9	0.2%	0	0.0%
mmu-mir-26a-1-5p	9	118940929	118940950	+	edit	16	0	0.0%	0	0.0%	0	0.0%
mmu-mir-26a-1-5p	9	118940929	118940950	+	edit	17	0	0.0%	0	0.0%	0	0.0%
mmu-mir-26a-1-5p	9	118940929	118940950	+	edit	18	0	0.0%	0	0.0%	0	0.0%
mmu-mir-26a-1-5p	9	118940929	118940950	+	edit	19	18	0.3%	16	0.4%	4	0.2%
mmu-mir-26a-1-5p	9	118940929	118940950	+	edit	20	0	0.0%	0	0.0%	0	0.0%
mmu-mir-26a-1-5p	9	118940929	118940950	+	edit	21	0	0.0%	0	0.0%	0	0.0%
mmu-mir-26a-1-5p	9	118940929	118940950	+	edit	22	0	0.0%	0	0.0%	0	0.0%
EDITED							41	0.7%	24	0.6%	4	0.2%
CANONICAL							6209	99.3%	4204	99.4%	2217	99.8%
TOTAL							6250		4228		2221	

miRNA	Chr	Start	Stop	Strand	Type	Position	P7 reads	P7%	P10 reads	P10%	P14 reads	P14%
mmu-mir-26a-2-5p	10	126432599	126432620	+	3' Edit	3'	59	0.9%	0	0.0%	33	1.4%
mmu-mir-26a-2-5p	10	126432599	126432620	+	Indel	22	0	0.0%	12	0.3%	0	0.0%
mmu-mir-26a-2-5p	10	126432599	126432620	+	edit	1	0	0.0%	0	0.0%	0	0.0%
mmu-mir-26a-2-5p	10	126432599	126432620	+	edit	2	0	0.0%	0	0.0%	0	0.0%
mmu-mir-26a-2-5p	10	126432599	126432620	+	edit	3	0	0.0%	0	0.0%	0	0.0%
mmu-mir-26a-2-5p	10	126432599	126432620	+	edit	4	5	0.1%	0	0.0%	0	0.0%
mmu-mir-26a-2-5p	10	126432599	126432620	+	edit	5	0	0.0%	0	0.0%	0	0.0%
mmu-mir-26a-2-5p	10	126432599	126432620	+	edit	6	0	0.0%	0	0.0%	0	0.0%
mmu-mir-26a-2-5p	10	126432599	126432620	+	edit	7	0	0.0%	0	0.0%	0	0.0%
mmu-mir-26a-2-5p	10	126432599	126432620	+	edit	8	5	0.1%	0	0.0%	0	0.0%
mmu-mir-26a-2-5p	10	126432599	126432620	+	edit	9	0	0.0%	0	0.0%	0	0.0%
mmu-mir-26a-2-5p	10	126432599	126432620	+	edit	10	0	0.0%	0	0.0%	0	0.0%
mmu-mir-26a-2-5p	10	126432599	126432620	+	edit	11	0	0.0%	0	0.0%	0	0.0%
mmu-mir-26a-2-5p	10	126432599	126432620	+	edit	12	0	0.0%	0	0.0%	0	0.0%
mmu-mir-26a-2-5p	10	126432599	126432620	+	edit	13	0	0.0%	0	0.0%	0	0.0%
mmu-mir-26a-2-5p	10	126432599	126432620	+	edit	14	0	0.0%	0	0.0%	0	0.0%
mmu-mir-26a-2-5p	10	126432599	126432620	+	edit	15	8	0.1%	9	0.2%	0	0.0%
mmu-mir-26a-2-5p	10	126432599	126432620	+	edit	16	0	0.0%	0	0.0%	0	0.0%
mmu-mir-26a-2-5p	10	126432599	126432620	+	edit	17	0	0.0%	0	0.0%	0	0.0%
mmu-mir-26a-2-5p	10	126432599	126432620	+	edit	18	0	0.0%	0	0.0%	0	0.0%
mmu-mir-26a-2-5p	10	126432599	126432620	+	edit	19	19	0.3%	16	0.4%	4	0.2%
mmu-mir-26a-2-5p	10	126432599	126432620	+	edit	20	0	0.0%	0	0.0%	0	0.0%
mmu-mir-26a-2-5p	10	126432599	126432620	+	edit	21	0	0.0%	0	0.0%	0	0.0%
mmu-mir-26a-2-5p	10	126432599	126432620	+	edit	22	0	0.0%	0	0.0%	0	0.0%
EDITED							95	1.5%	36	0.8%	37	1.6%
CANONICAL							6270	98.5%	4259	99.2%	2249	98.4%
TOTAL							6365		4295		2286	

miRNA	Chr	Start	Stop	Strand	Type	Position	P7 reads	P7%	P10 reads	P10%	P14 reads	P14%
mmu-mir-26b-5p	1	74440898	74440918	+	3' Edit	3'	3	0.1%	0	0.0%	5	0.4%
mmu-mir-26b-5p	1	74440898	74440918	+	3' Indel	3'	5	0.1%	0	0.0%	6	0.5%
mmu-mir-26b-5p	1	74440898	74440918	+	edit	1	0	0.0%	0	0.0%	0	0.0%
mmu-mir-26b-5p	1	74440898	74440918	+	edit	2	0	0.0%	0	0.0%	0	0.0%
mmu-mir-26b-5p	1	74440898	74440918	+	edit	3	0	0.0%	0	0.0%	0	0.0%
mmu-mir-26b-5p	1	74440898	74440918	+	edit	4	4	0.1%	3	0.1%	0	0.0%
mmu-mir-26b-5p	1	74440898	74440918	+	edit	5	0	0.0%	0	0.0%	0	0.0%
mmu-mir-26b-5p	1	74440898	74440918	+	edit	6	0	0.0%	0	0.0%	0	0.0%
mmu-mir-26b-5p	1	74440898	74440918	+	edit	7	0	0.0%	0	0.0%	0	0.0%
mmu-mir-26b-5p	1	74440898	74440918	+	edit	8	0	0.0%	0	0.0%	0	0.0%
mmu-mir-26b-5p	1	74440898	74440918	+	edit	9	0	0.0%	0	0.0%	0	0.0%
mmu-mir-26b-5p	1	74440898	74440918	+	edit	10	0	0.0%	0	0.0%	0	0.0%
mmu-mir-26b-5p	1	74440898	74440918	+	edit	11	0	0.0%	0	0.0%	0	0.0%
mmu-mir-26b-5p	1	74440898	74440918	+	edit	12	0	0.0%	0	0.0%	0	0.0%
mmu-mir-26b-5p	1	74440898	74440918	+	edit	13	0	0.0%	0	0.0%	0	0.0%
mmu-mir-26b-5p	1	74440898	74440918	+	edit	14	0	0.0%	0	0.0%	0	0.0%
mmu-mir-26b-5p	1	74440898	74440918	+	edit	15	0	0.0%	7	0.3%	0	0.0%
mmu-mir-26b-5p	1	74440898	74440918	+	edit	16	0	0.0%	0	0.0%	0	0.0%
mmu-mir-26b-5p	1	74440898	74440918	+	edit	17	0	0.0%	0	0.0%	0	0.0%
mmu-mir-26b-5p	1	74440898	74440918	+	edit	18	0	0.0%	0	0.0%	0	0.0%
mmu-mir-26b-5p	1	74440898	74440918	+	edit	19	0	0.0%	3	0.1%	0	0.0%
mmu-mir-26b-5p	1	74440898	74440918	+	edit	20	0	0.0%	0	0.0%	0	0.0%
mmu-mir-26b-5p	1	74440898	74440918	+	edit	21	0	0.0%	0	0.0%	0	0.0%
mmu-mir-26b-5p	1	74440898	74440918	+	edit	22	0	0.0%	0	0.0%	0	0.0%
EDITED							12	0.4%	13	0.6%	11	1.0%
CANONICAL							3401	99.6%	2145	99.4%	1141	99.0%
TOTAL							3413		2158		1152	

miRNA	Chr	Start	Stop	Strand	Type	Position	P7 reads	P7%	P10 reads	P10%	P14 reads	P14%
mmu-mir-29a-3p	6	31012673	31012694	-	edit	1	0	0.0%	0	0.0%	0	0.0%
mmu-mir-29a-3p	6	31012673	31012694	-	edit	2	0	0.0%	0	0.0%	0	0.0%
mmu-mir-29a-3p	6	31012673	31012694	-	edit	3	0	0.0%	0	0.0%	0	0.0%
mmu-mir-29a-3p	6	31012673	31012694	-	edit	4	0	0.0%	0	0.0%	0	0.0%
mmu-mir-29a-3p	6	31012673	31012694	-	edit	5	0	0.0%	0	0.0%	0	0.0%
mmu-mir-29a-3p	6	31012673	31012694	-	edit	6	0	0.0%	0	0.0%	0	0.0%
mmu-mir-29a-3p	6	31012673	31012694	-	edit	7	0	0.0%	0	0.0%	0	0.0%
mmu-mir-29a-3p	6	31012673	31012694	-	edit	8	0	0.0%	0	0.0%	0	0.0%
mmu-mir-29a-3p	6	31012673	31012694	-	edit	9	0	0.0%	0	0.0%	0	0.0%
mmu-mir-29a-3p	6	31012673	31012694	-	edit	10	0	0.0%	0	0.0%	0	0.0%
mmu-mir-29a-3p	6	31012673	31012694	-	edit	11	0	0.0%	0	0.0%	0	0.0%
mmu-mir-29a-3p	6	31012673	31012694	-	edit	12	0	0.0%	0	0.0%	0	0.0%
mmu-mir-29a-3p	6	31012673	31012694	-	edit	13	0	0.0%	0	0.0%	0	0.0%
mmu-mir-29a-3p	6	31012673	31012694	-	edit	14	0	0.0%	0	0.0%	0	0.0%
mmu-mir-29a-3p	6	31012673	31012694	-	edit	15	0	0.0%	16	0.6%	0	0.0%
mmu-mir-29a-3p	6	31012673	31012694	-	edit	16	0	0.0%	0	0.0%	0	0.0%
mmu-mir-29a-3p	6	31012673	31012694	-	edit	17	0	0.0%	0	0.0%	16	0.6%
mmu-mir-29a-3p	6	31012673	31012694	-	edit	18	0	0.0%	0	0.0%	0	0.0%
mmu-mir-29a-3p	6	31012673	31012694	-	edit	19	0	0.0%	4	#REF!	3	0.1%
mmu-mir-29a-3p	6	31012673	31012694	-	edit	20	0	0.0%	0	0.0%	0	0.0%
mmu-mir-29a-3p	6	31012673	31012694	-	edit	21	0	0.0%	0	0.0%	3	0.1%
mmu-mir-29a-3p	6	31012673	31012694	-	edit	22	0	0.0%	0	0.0%	0	0.0%
EDITED							0	0.0%	20	0.7%	22	0.8%
CANONICAL							1694	100.0%	2696	99.3%	2577	99.2%
TOTAL							1694		2716		2599	

miRNA	Chr	Start	Stop	Strand	Type	Position	P7 reads	P7%	P10 reads	P10%	P14 reads	P14%
mmu-mir-29c-3p	1	196863794	196863815	+	edit	1	0	0.0%	0	0.0%	0	0.0%
mmu-mir-29c-3p	1	196863794	196863815	+	edit	2	0	0.0%	0	0.0%	0	0.0%
mmu-mir-29c-3p	1	196863794	196863815	+	edit	3	0	0.0%	0	0.0%	0	0.0%
mmu-mir-29c-3p	1	196863794	196863815	+	edit	4	0	0.0%	0	0.0%	0	0.0%
mmu-mir-29c-3p	1	196863794	196863815	+	edit	5	0	0.0%	0	0.0%	0	0.0%
mmu-mir-29c-3p	1	196863794	196863815	+	edit	6	0	0.0%	0	0.0%	0	0.0%
mmu-mir-29c-3p	1	196863794	196863815	+	edit	7	0	0.0%	0	0.0%	0	0.0%
mmu-mir-29c-3p	1	196863794	196863815	+	edit	8	0	0.0%	0	0.0%	0	0.0%
mmu-mir-29c-3p	1	196863794	196863815	+	edit	9	0	0.0%	0	0.0%	0	0.0%
mmu-mir-29c-3p	1	196863794	196863815	+	edit	10	0	0.0%	0	0.0%	0	0.0%
mmu-mir-29c-3p	1	196863794	196863815	+	edit	11	0	0.0%	0	0.0%	0	0.0%
mmu-mir-29c-3p	1	196863794	196863815	+	edit	12	0	0.0%	0	0.0%	0	0.0%
mmu-mir-29c-3p	1	196863794	196863815	+	edit	13	0	0.0%	0	0.0%	0	0.0%
mmu-mir-29c-3p	1	196863794	196863815	+	edit	14	0	0.0%	0	0.0%	0	0.0%
mmu-mir-29c-3p	1	196863794	196863815	+	edit	15	0	0.0%	3	1.0%	0	0.0%
mmu-mir-29c-3p	1	196863794	196863815	+	edit	16	0	0.0%	0	0.0%	0	0.0%
mmu-mir-29c-3p	1	196863794	196863815	+	edit	17	0	0.0%	0	0.0%	0	0.0%
mmu-mir-29c-3p	1	196863794	196863815	+	edit	18	0	0.0%	0	0.0%	0	0.0%
mmu-mir-29c-3p	1	196863794	196863815	+	edit	19	0	0.0%	0	0.0%	0	0.0%
mmu-mir-29c-3p	1	196863794	196863815	+	edit	20	0	0.0%	0	0.0%	0	0.0%
mmu-mir-29c-3p	1	196863794	196863815	+	edit	21	0	0.0%	0	0.0%	0	0.0%
mmu-mir-29c-3p	1	196863794	196863815	+	edit	22	0	0.0%	0	0.0%	0	0.0%
EDITED							0	0.0%	3	1.0%	0	0.0%
CANONICAL							188	100.0%	293	99.0%	248	100.0%
TOTAL							188		296		248	

miRNA	Chr	Start	Stop	Strand	Type	Position	P7 reads	P7%	P10 reads	P10%	P14 reads	P14%
mmu-mir-30a-3p	1	23279154	23279175	+	edit	1	0	0.0%	0	0.0%	0	0.0%
mmu-mir-30a-3p	1	23279154	23279175	+	edit	2	0	0.0%	0	0.0%	0	0.0%
mmu-mir-30a-3p	1	23279154	23279175	+	edit	3	0	0.0%	0	0.0%	0	0.0%
mmu-mir-30a-3p	1	23279154	23279175	+	edit	4	0	0.0%	0	0.0%	0	0.0%
mmu-mir-30a-3p	1	23279154	23279175	+	edit	5	0	0.0%	0	0.0%	0	0.0%
mmu-mir-30a-3p	1	23279154	23279175	+	edit	6	0	0.0%	0	0.0%	0	0.0%
mmu-mir-30a-3p	1	23279154	23279175	+	edit	7	0	0.0%	0	0.0%	0	0.0%
mmu-mir-30a-3p	1	23279154	23279175	+	edit	8	0	0.0%	0	0.0%	0	0.0%
mmu-mir-30a-3p	1	23279154	23279175	+	edit	9	0	0.0%	0	0.0%	0	0.0%
mmu-mir-30a-3p	1	23279154	23279175	+	edit	10	0	0.0%	0	0.0%	0	0.0%
mmu-mir-30a-3p	1	23279154	23279175	+	edit	11	0	0.0%	0	0.0%	0	0.0%
mmu-mir-30a-3p	1	23279154	23279175	+	edit	12	0	0.0%	0	0.0%	0	0.0%
mmu-mir-30a-3p	1	23279154	23279175	+	edit	13	0	0.0%	0	0.0%	0	0.0%
mmu-mir-30a-3p	1	23279154	23279175	+	edit	14	0	0.0%	0	0.0%	0	0.0%
mmu-mir-30a-3p	1	23279154	23279175	+	edit	15	0	0.0%	0	0.0%	9	0.6%
mmu-mir-30a-3p	1	23279154	23279175	+	edit	16	0	0.0%	0	0.0%	0	0.0%
mmu-mir-30a-3p	1	23279154	23279175	+	edit	17	0	0.0%	0	0.0%	0	0.0%
mmu-mir-30a-3p	1	23279154	23279175	+	edit	18	0	0.0%	0	0.0%	0	0.0%
mmu-mir-30a-3p	1	23279154	23279175	+	edit	19	8	0.5%	0	0.0%	16	1.0%
mmu-mir-30a-3p	1	23279154	23279175	+	edit	20	0	0.0%	0	0.0%	0	0.0%
mmu-mir-30a-3p	1	23279154	23279175	+	edit	21	0	0.0%	0	0.0%	0	0.0%
mmu-mir-30a-3p	1	23279154	23279175	+	edit	22	0	0.0%	0	0.0%	0	0.0%
EDITED							8	0.5%	0	0.0%	25	1.6%
CANONICAL							1583	99.5%	907	100.0%	1532	98.4%
TOTAL							1591		907		1557	

miRNA	Chr	Start	Stop	Strand	Type	Position	P7 reads	P7%	P10 reads	P10%	P14 reads	P14%
mmu-mir-30a-5p	1	23279113	23279134	+	3' Edit	3'	6	0.1%	0	0.0%	7	0.1%
mmu-mir-30a-5p	1	23279113	23279134	+	edit	1	0	0.0%	0	0.0%	0	0.0%
mmu-mir-30a-5p	1	23279113	23279134	+	edit	2	0	0.0%	0	0.0%	0	0.0%
mmu-mir-30a-5p	1	23279113	23279134	+	edit	3	0	0.0%	0	0.0%	0	0.0%
mmu-mir-30a-5p	1	23279113	23279134	+	edit	4	0	0.0%	4	0.1%	4	0.1%
mmu-mir-30a-5p	1	23279113	23279134	+	edit	5	0	0.0%	0	0.0%	0	0.0%
mmu-mir-30a-5p	1	23279113	23279134	+	edit	6	0	0.0%	4	0.1%	0	0.0%
mmu-mir-30a-5p	1	23279113	23279134	+	edit	7	0	0.0%	0	0.0%	0	0.0%
mmu-mir-30a-5p	1	23279113	23279134	+	edit	8	3	0.0%	0	0.0%	0	0.0%
mmu-mir-30a-5p	1	23279113	23279134	+	edit	9	0	0.0%	0	0.0%	0	0.0%
mmu-mir-30a-5p	1	23279113	23279134	+	edit	10	0	0.0%	0	0.0%	0	0.0%
mmu-mir-30a-5p	1	23279113	23279134	+	edit	11	0	0.0%	0	0.0%	0	0.0%
mmu-mir-30a-5p	1	23279113	23279134	+	edit	12	15	0.2%	4	0.1%	0	0.0%
mmu-mir-30a-5p	1	23279113	23279134	+	edit	13	0	0.0%	0	0.0%	0	0.0%
mmu-mir-30a-5p	1	23279113	23279134	+	edit	14	0	0.0%	0	0.0%	0	0.0%
mmu-mir-30a-5p	1	23279113	23279134	+	edit	15	11	0.1%	60	1.0%	10	0.2%
mmu-mir-30a-5p	1	23279113	23279134	+	edit	16	0	0.0%	0	0.0%	0	0.0%
mmu-mir-30a-5p	1	23279113	23279134	+	edit	17	11	0.1%	0	0.0%	6	0.1%
mmu-mir-30a-5p	1	23279113	23279134	+	edit	18	0	0.0%	0	0.0%	0	0.0%
mmu-mir-30a-5p	1	23279113	23279134	+	edit	19	19	0.2%	16	0.3%	7	0.1%
mmu-mir-30a-5p	1	23279113	23279134	+	edit	20	0	0.0%	0	0.0%	0	0.0%
mmu-mir-30a-5p	1	23279113	23279134	+	edit	21	12	0.2%	56	0.9%	8	0.1%
mmu-mir-30a-5p	1	23279113	23279134	+	edit	22	0	0.0%	0	0.0%	0	0.0%
EDITED							77	1.0%	143	2.4%	42	0.7%
CANONICAL							7565	99.0%	5788	97.6%	5882	99.3%
TOTAL							7642		5931		5924	

miRNA	Chr	Start	Stop	Strand	Type	Position	P7 reads	P7%	P10 reads	P10%	P14 reads	P14%
mmu-mir-30c-1-5p	4	120442189	120442211	-	edit	1	0	0.0%	0	0.0%	0	0.0%
mmu-mir-30c-1-5p	4	120442189	120442211	-	edit	2	0	0.0%	0	0.0%	0	0.0%
mmu-mir-30c-1-5p	4	120442189	120442211	-	edit	3	0	0.0%	0	0.0%	0	0.0%
mmu-mir-30c-1-5p	4	120442189	120442211	-	edit	4	0	0.0%	0	0.0%	0	0.0%
mmu-mir-30c-1-5p	4	120442189	120442211	-	edit	5	0	0.0%	0	0.0%	0	0.0%
mmu-mir-30c-1-5p	4	120442189	120442211	-	edit	6	0	0.0%	0	0.0%	0	0.0%
mmu-mir-30c-1-5p	4	120442189	120442211	-	edit	7	0	0.0%	0	0.0%	0	0.0%
mmu-mir-30c-1-5p	4	120442189	120442211	-	edit	8	0	0.0%	0	0.0%	0	0.0%
mmu-mir-30c-1-5p	4	120442189	120442211	-	edit	9	0	0.0%	0	0.0%	0	0.0%
mmu-mir-30c-1-5p	4	120442189	120442211	-	edit	10	0	0.0%	0	0.0%	0	0.0%
mmu-mir-30c-1-5p	4	120442189	120442211	-	edit	11	0	0.0%	0	0.0%	0	0.0%
mmu-mir-30c-1-5p	4	120442189	120442211	-	edit	12	0	0.0%	0	0.0%	0	0.0%
mmu-mir-30c-1-5p	4	120442189	120442211	-	edit	13	0	0.0%	0	0.0%	0	0.0%
mmu-mir-30c-1-5p	4	120442189	120442211	-	edit	14	0	0.0%	0	0.0%	0	0.0%
mmu-mir-30c-1-5p	4	120442189	120442211	-	edit	15	0	0.0%	0	0.0%	0	0.0%
mmu-mir-30c-1-5p	4	120442189	120442211	-	edit	16	0	0.0%	0	0.0%	0	0.0%
mmu-mir-30c-1-5p	4	120442189	120442211	-	edit	17	0	0.0%	0	0.0%	0	0.0%
mmu-mir-30c-1-5p	4	120442189	120442211	-	edit	18	0	0.0%	0	0.0%	0	0.0%
mmu-mir-30c-1-5p	4	120442189	120442211	-	edit	19	0	0.0%	0	0.0%	0	0.0%
mmu-mir-30c-1-5p	4	120442189	120442211	-	edit	20	0	0.0%	0	0.0%	0	0.0%
mmu-mir-30c-1-5p	4	120442189	120442211	-	edit	21	0	0.0%	10	3.0%	0	0.0%
mmu-mir-30c-1-5p	4	120442189	120442211	-	edit	22	0	0.0%	0	0.0%	0	0.0%
EDITED							0	0.0%	10	3.0%	0	0.0%
CANONICAL							323	100.0%	306	97.0%	193	100.0%
TOTAL							323		316		193	

miRNA	Chr	Start	Stop	Strand	Type	Position	P7 reads	P7%	P10 reads	P10%	P14 reads	P14%
mmu-mir-30c-2-3p	1	23298593	23298614	+	edit	1	0	0.0%	0	0.0%	0	0.0%
mmu-mir-30c-2-3p	1	23298593	23298614	+	edit	2	0	0.0%	0	0.0%	0	0.0%
mmu-mir-30c-2-3p	1	23298593	23298614	+	edit	3	0	0.0%	0	0.0%	0	0.0%
mmu-mir-30c-2-3p	1	23298593	23298614	+	edit	4	0	0.0%	0	0.0%	0	0.0%
mmu-mir-30c-2-3p	1	23298593	23298614	+	edit	5	0	0.0%	0	0.0%	0	0.0%
mmu-mir-30c-2-3p	1	23298593	23298614	+	edit	6	0	0.0%	0	0.0%	0	0.0%
mmu-mir-30c-2-3p	1	23298593	23298614	+	edit	7	0	0.0%	0	0.0%	0	0.0%
mmu-mir-30c-2-3p	1	23298593	23298614	+	edit	8	0	0.0%	0	0.0%	0	0.0%
mmu-mir-30c-2-3p	1	23298593	23298614	+	edit	9	0	0.0%	0	0.0%	0	0.0%
mmu-mir-30c-2-3p	1	23298593	23298614	+	edit	10	0	0.0%	0	0.0%	0	0.0%
mmu-mir-30c-2-3p	1	23298593	23298614	+	edit	11	0	0.0%	0	0.0%	0	0.0%
mmu-mir-30c-2-3p	1	23298593	23298614	+	edit	12	0	0.0%	0	0.0%	0	0.0%
mmu-mir-30c-2-3p	1	23298593	23298614	+	edit	13	0	0.0%	0	0.0%	0	0.0%
mmu-mir-30c-2-3p	1	23298593	23298614	+	edit	14	0	0.0%	0	0.0%	0	0.0%
mmu-mir-30c-2-3p	1	23298593	23298614	+	edit	15	0	0.0%	0	0.0%	0	0.0%
mmu-mir-30c-2-3p	1	23298593	23298614	+	edit	16	0	0.0%	0	0.0%	0	0.0%
mmu-mir-30c-2-3p	1	23298593	23298614	+	edit	17	0	0.0%	0	0.0%	0	0.0%
mmu-mir-30c-2-3p	1	23298593	23298614	+	edit	18	0	0.0%	0	0.0%	0	0.0%
mmu-mir-30c-2-3p	1	23298593	23298614	+	edit	19	5	0.9%	3	1.0%	3	1.0%
mmu-mir-30c-2-3p	1	23298593	23298614	+	edit	20	0	0.0%	0	0.0%	0	0.0%
mmu-mir-30c-2-3p	1	23298593	23298614	+	edit	21	0	0.0%	10	3.2%	0	0.0%
mmu-mir-30c-2-3p	1	23298593	23298614	+	edit	22	0	0.0%	0	0.0%	0	0.0%
EDITED							5	0.9%	13	4.1%	3	1.0%
CANONICAL							487	99.1%	289	95.9%	289	99.0%
TOTAL							492		302		292	

miRNA	Chr	Start	Stop	Strand	Type	Position	P7 reads	P7%	P10 reads	P10%	P14 reads	P14%
mmu-mir-30d-5p	15	68172819	68172840	-	5' Edit	5'	9	0.1%	0	0.0%	13	0.4%
mmu-mir-30d-5p	15	68172819	68172840	-	edit	1	0	0.0%	0	0.0%	0	0.0%
mmu-mir-30d-5p	15	68172819	68172840	-	edit	2	0	0.0%	0	0.0%	0	0.0%
mmu-mir-30d-5p	15	68172819	68172840	-	edit	3	0	0.0%	0	0.0%	0	0.0%
mmu-mir-30d-5p	15	68172819	68172840	-	edit	4	0	0.0%	0	0.0%	0	0.0%
mmu-mir-30d-5p	15	68172819	68172840	-	edit	5	0	0.0%	0	0.0%	0	0.0%
mmu-mir-30d-5p	15	68172819	68172840	-	edit	6	0	0.0%	0	0.0%	0	0.0%
mmu-mir-30d-5p	15	68172819	68172840	-	edit	7	0	0.0%	0	0.0%	0	0.0%
mmu-mir-30d-5p	15	68172819	68172840	-	edit	8	4	0.1%	0	0.0%	0	0.0%
mmu-mir-30d-5p	15	68172819	68172840	-	edit	9	0	0.0%	0	0.0%	0	0.0%
mmu-mir-30d-5p	15	68172819	68172840	-	edit	10	0	0.0%	0	0.0%	3	0.1%
mmu-mir-30d-5p	15	68172819	68172840	-	edit	11	0	0.0%	0	0.0%	0	0.0%
mmu-mir-30d-5p	15	68172819	68172840	-	edit	12	0	0.0%	0	0.0%	0	0.0%
mmu-mir-30d-5p	15	68172819	68172840	-	edit	13	8	0.1%	8	0.2%	12	0.4%
mmu-mir-30d-5p	15	68172819	68172840	-	edit	14	0	0.0%	0	0.0%	0	0.0%
mmu-mir-30d-5p	15	68172819	68172840	-	edit	15	34	0.5%	119	2.2%	20	0.6%
mmu-mir-30d-5p	15	68172819	68172840	-	edit	16	6	0.1%	9	0.2%	6	0.2%
mmu-mir-30d-5p	15	68172819	68172840	-	edit	17	52	0.7%	9	0.2%	29	0.8%
mmu-mir-30d-5p	15	68172819	68172840	-	edit	18	5	0.1%	0	0.0%	0	0.0%
mmu-mir-30d-5p	15	68172819	68172840	-	edit	19	20	0.3%	14	0.3%	11	0.3%
mmu-mir-30d-5p	15	68172819	68172840	-	edit	20	5	0.1%	20	0.4%	0	0.0%
mmu-mir-30d-5p	15	68172819	68172840	-	edit	21	24	0.3%	90	1.7%	13	0.4%
mmu-mir-30d-5p	15	68172819	68172840	-	edit	22	0	0.0%	0	0.0%	0	0.0%
EDITED							166	2.3%	269	5.1%	106	3.1%
CANONICAL							7116	97.7%	5031	94.9%	3302	96.9%
TOTAL							7282		5300		3408	

miRNA	Chr	Start	Stop	Strand	Type	Position	P7 reads	P7%	P10 reads	P10%	P14 reads	P14%
mmu-mir-30e-3p	4	120445223	120445244	-	edit	1	0	0.0%	0	0.0%	0	0.0%
mmu-mir-30e-3p	4	120445223	120445244	-	edit	2	0	0.0%	0	0.0%	0	0.0%
mmu-mir-30e-3p	4	120445223	120445244	-	edit	3	0	0.0%	0	0.0%	0	0.0%
mmu-mir-30e-3p	4	120445223	120445244	-	edit	4	0	0.0%	0	0.0%	0	0.0%
mmu-mir-30e-3p	4	120445223	120445244	-	edit	5	0	0.0%	0	0.0%	0	0.0%
mmu-mir-30e-3p	4	120445223	120445244	-	edit	6	0	0.0%	0	0.0%	0	0.0%
mmu-mir-30e-3p	4	120445223	120445244	-	edit	7	0	0.0%	0	0.0%	0	0.0%
mmu-mir-30e-3p	4	120445223	120445244	-	edit	8	0	0.0%	0	0.0%	0	0.0%
mmu-mir-30e-3p	4	120445223	120445244	-	edit	9	0	0.0%	0	0.0%	0	0.0%
mmu-mir-30e-3p	4	120445223	120445244	-	edit	10	0	0.0%	0	0.0%	0	0.0%
mmu-mir-30e-3p	4	120445223	120445244	-	edit	11	0	0.0%	0	0.0%	0	0.0%
mmu-mir-30e-3p	4	120445223	120445244	-	edit	12	0	0.0%	0	0.0%	0	0.0%
mmu-mir-30e-3p	4	120445223	120445244	-	edit	13	0	0.0%	0	0.0%	0	0.0%
mmu-mir-30e-3p	4	120445223	120445244	-	edit	14	0	0.0%	0	0.0%	0	0.0%
mmu-mir-30e-3p	4	120445223	120445244	-	edit	15	7	0.5%	0	0.0%	0	0.0%
mmu-mir-30e-3p	4	120445223	120445244	-	edit	16	0	0.0%	0	0.0%	0	0.0%
mmu-mir-30e-3p	4	120445223	120445244	-	edit	17	0	0.0%	0	0.0%	0	0.0%
mmu-mir-30e-3p	4	120445223	120445244	-	edit	18	0	0.0%	0	0.0%	0	0.0%
mmu-mir-30e-3p	4	120445223	120445244	-	edit	19	5	0.4%	0	0.0%	8	1.3%
mmu-mir-30e-3p	4	120445223	120445244	-	edit	20	0	0.0%	0	0.0%	0	0.0%
mmu-mir-30e-3p	4	120445223	120445244	-	edit	21	0	0.0%	0	0.0%	0	0.0%
mmu-mir-30e-3p	4	120445223	120445244	-	edit	22	0	0.0%	0	0.0%	0	0.0%
EDITED							11	0.9%	0	0.0%	8	1.3%
CANONICAL							1217	99.1%	481	100.0%	587	98.7%
TOTAL							1228		481		595	

miRNA	Chr	Start	Stop	Strand	Type	Position	P7 reads	P7%	P10 reads	P10%	P14 reads	P14%
mmu-mir-30e-5p	4	120445265	120445286	-	edit	1	0	0.0%	0	0.0%	0	0.0%
mmu-mir-30e-5p	4	120445265	120445286	-	edit	2	0	0.0%	0	0.0%	0	0.0%
mmu-mir-30e-5p	4	120445265	120445286	-	edit	3	0	0.0%	0	0.0%	0	0.0%
mmu-mir-30e-5p	4	120445265	120445286	-	edit	4	0	0.0%	0	0.0%	0	0.0%
mmu-mir-30e-5p	4	120445265	120445286	-	edit	5	0	0.0%	0	0.0%	0	0.0%
mmu-mir-30e-5p	4	120445265	120445286	-	edit	6	0	0.0%	0	0.0%	0	0.0%
mmu-mir-30e-5p	4	120445265	120445286	-	edit	7	0	0.0%	0	0.0%	0	0.0%
mmu-mir-30e-5p	4	120445265	120445286	-	edit	8	0	0.0%	0	0.0%	0	0.0%
mmu-mir-30e-5p	4	120445265	120445286	-	edit	9	0	0.0%	0	0.0%	0	0.0%
mmu-mir-30e-5p	4	120445265	120445286	-	edit	10	0	0.0%	0	0.0%	0	0.0%
mmu-mir-30e-5p	4	120445265	120445286	-	edit	11	0	0.0%	0	0.0%	0	0.0%
mmu-mir-30e-5p	4	120445265	120445286	-	edit	12	0	0.0%	0	0.0%	0	0.0%
mmu-mir-30e-5p	4	120445265	120445286	-	edit	13	4	0.2%	0	0.0%	3	0.2%
mmu-mir-30e-5p	4	120445265	120445286	-	edit	14	0	0.0%	0	0.0%	0	0.0%
mmu-mir-30e-5p	4	120445265	120445286	-	edit	15	3	0.1%	6	0.4%	0	0.0%
mmu-mir-30e-5p	4	120445265	120445286	-	edit	16	0	0.0%	0	0.0%	0	0.0%
mmu-mir-30e-5p	4	120445265	120445286	-	edit	17	4	0.1%	0	0.0%	0	0.0%
mmu-mir-30e-5p	4	120445265	120445286	-	edit	18	0	0.0%	0	0.0%	0	0.0%
mmu-mir-30e-5p	4	120445265	120445286	-	edit	19	3	0.1%	0	0.0%	0	0.0%
mmu-mir-30e-5p	4	120445265	120445286	-	edit	20	3	0.1%	0	0.0%	0	0.0%
mmu-mir-30e-5p	4	120445265	120445286	-	edit	21	4	0.2%	13	0.8%	0	0.0%
mmu-mir-30e-5p	4	120445265	120445286	-	edit	22	0	0.0%	0	0.0%	0	0.0%
EDITED							21	0.8%	19	1.1%	3	0.2%
CANONICAL							2605	99.2%	1694	98.9%	1211	99.8%
TOTAL							2626		1713		1214	

miRNA	Chr	Start	Stop	Strand	Type	Position	P7 reads	P7%	P10 reads	P10%	P14 reads	P14%
mmu-mir-34b-5p	9	50911715	50911737	-	edit	1	0	0.0%	0	0.0%	0	0.0%
mmu-mir-34b-5p	9	50911715	50911737	-	edit	2	0	0.0%	0	0.0%	0	0.0%
mmu-mir-34b-5p	9	50911715	50911737	-	edit	3	0	0.0%	0	0.0%	0	0.0%
mmu-mir-34b-5p	9	50911715	50911737	-	edit	4	0	0.0%	0	0.0%	0	0.0%
mmu-mir-34b-5p	9	50911715	50911737	-	edit	5	0	0.0%	0	0.0%	0	0.0%
mmu-mir-34b-5p	9	50911715	50911737	-	edit	6	0	0.0%	0	0.0%	0	0.0%
mmu-mir-34b-5p	9	50911715	50911737	-	edit	7	0	0.0%	0	0.0%	0	0.0%
mmu-mir-34b-5p	9	50911715	50911737	-	edit	8	0	0.0%	0	0.0%	0	0.0%
mmu-mir-34b-5p	9	50911715	50911737	-	edit	9	0	0.0%	0	0.0%	0	0.0%
mmu-mir-34b-5p	9	50911715	50911737	-	edit	10	0	0.0%	0	0.0%	0	0.0%
mmu-mir-34b-5p	9	50911715	50911737	-	edit	11	**10**	**15.2%**	**4**	**9.8%**	**77**	**12.9%**
mmu-mir-34b-5p	9	50911715	50911737	-	edit	12	0	0.0%	0	0.0%	0	0.0%
mmu-mir-34b-5p	9	50911715	50911737	-	edit	13	0	0.0%	0	0.0%	0	0.0%
mmu-mir-34b-5p	9	50911715	50911737	-	edit	14	0	0.0%	0	0.0%	0	0.0%
mmu-mir-34b-5p	9	50911715	50911737	-	edit	15	0	0.0%	0	0.0%	0	0.0%
mmu-mir-34b-5p	9	50911715	50911737	-	edit	16	0	0.0%	0	0.0%	0	0.0%
mmu-mir-34b-5p	9	50911715	50911737	-	edit	17	0	0.0%	0	0.0%	0	0.0%
mmu-mir-34b-5p	9	50911715	50911737	-	edit	18	0	0.0%	0	0.0%	0	0.0%
mmu-mir-34b-5p	9	50911715	50911737	-	edit	19	0	0.0%	0	0.0%	3	0.5%
mmu-mir-34b-5p	9	50911715	50911737	-	edit	20	0	0.0%	0	0.0%	0	0.0%
mmu-mir-34b-5p	9	50911715	50911737	-	edit	21	0	0.0%	0	0.0%	0	0.0%
mmu-mir-34b-5p	9	50911715	50911737	-	edit	22	0	0.0%	0	0.0%	0	0.0%
EDITED							10	15.2%	4	9.8%	80	13.4%
CANONICAL							56	84.8%	37	90.2%	519	86.6%
TOTAL							66		41		599	

miRNA	Chr	Start	Stop	Strand	Type	Position	P7 reads	P7%	P10 reads	P10%	P14 reads	P14%
mmu-mir-34c-5p	9	50911181	50911203	-	5' Edit	5'	0	0.0%	0	0.0%	4	0.0%
mmu-mir-34c-5p	9	50911181	50911203	-	5' Indel	5'	0	0.0%	0	0.0%	7	0.0%
mmu-mir-34c-5p	9	50911181	50911203	-	edit	1	0	0.0%	0	0.0%	0	0.0%
mmu-mir-34c-5p	9	50911181	50911203	-	edit	2	0	0.0%	0	0.0%	0	0.0%
mmu-mir-34c-5p	9	50911181	50911203	-	edit	3	0	0.0%	0	0.0%	0	0.0%
mmu-mir-34c-5p	9	50911181	50911203	-	edit	4	0	0.0%	0	0.0%	9	0.0%
mmu-mir-34c-5p	9	50911181	50911203	-	edit	5	0	0.0%	0	0.0%	9	0.0%
mmu-mir-34c-5p	9	50911181	50911203	-	edit	6	0	0.0%	0	0.0%	0	0.0%
mmu-mir-34c-5p	9	50911181	50911203	-	edit	7	0	0.0%	0	0.0%	0	0.0%
mmu-mir-34c-5p	9	50911181	50911203	-	edit	8	0	0.0%	0	0.0%	6	0.0%
mmu-mir-34c-5p	9	50911181	50911203	-	edit	9	3	0.1%	3	0.1%	67	0.2%
mmu-mir-34c-5p	9	50911181	50911203	-	edit	10	0	0.0%	0	0.0%	4	0.0%
mmu-mir-34c-5p	9	50911181	50911203	-	edit	11	0	0.0%	0	0.0%	0	0.0%
mmu-mir-34c-5p	9	50911181	50911203	-	edit	12	0	0.0%	0	0.0%	0	0.0%
mmu-mir-34c-5p	9	50911181	50911203	-	edit	13	0	0.0%	0	0.0%	15	0.1%
mmu-mir-34c-5p	9	50911181	50911203	-	edit	14	0	0.0%	0	0.0%	0	0.0%
mmu-mir-34c-5p	9	50911181	50911203	-	edit	15	4	0.1%	4	0.2%	50	0.2%
mmu-mir-34c-5p	9	50911181	50911203	-	edit	16	18	0.7%	31	1.4%	283	1.0%
mmu-mir-34c-5p	9	50911181	50911203	-	edit	17	0	0.0%	0	0.0%	74	0.3%
mmu-mir-34c-5p	9	50911181	50911203	-	edit	18	0	0.0%	5	0.2%	23	0.1%
mmu-mir-34c-5p	9	50911181	50911203	-	edit	19	13	0.5%	36	1.7%	191	0.7%
mmu-mir-34c-5p	9	50911181	50911203	-	edit	20	4	0.1%	0	0.0%	83	0.3%
mmu-mir-34c-5p	9	50911181	50911203	-	edit	21	6	0.2%	0	0.0%	61	0.2%
mmu-mir-34c-5p	9	50911181	50911203	-	edit	22	0	0.0%	0	0.0%	36	0.1%
mmu-mir-34c-5p	9	50911181	50911203	-	edit	23	0	0.0%	0	0.0%	6	0.0%
EDITED							48	1.7%	78	3.6%	928	3.3%
CANONICAL							2719	98.3%	2069	96.4%	27073	96.7%
TOTAL							2767		2147		28001	

miRNA	Chr	Start	Stop	Strand	Type	Position	P7 reads	P7%	P10 reads	P10%	P14 reads	P14%
mmu-mir-92a-1-5p	14	115443698	115443718	+	edit	1	0	0.0%	0	0.0%	0	0.0%
mmu-mir-92a-1-5p	14	115443698	115443718	+	edit	2	0	0.0%	0	0.0%	0	0.0%
mmu-mir-92a-1-5p	14	115443698	115443718	+	edit	3	0	0.0%	0	0.0%	0	0.0%
mmu-mir-92a-1-5p	14	115443698	115443718	+	edit	4	3	0.2%	0	0.0%	3	0.3%
mmu-mir-92a-1-5p	14	115443698	115443718	+	edit	5	0	0.0%	0	0.0%	0	0.0%
mmu-mir-92a-1-5p	14	115443698	115443718	+	edit	6	0	0.0%	0	0.0%	0	0.0%
mmu-mir-92a-1-5p	14	115443698	115443718	+	edit	7	0	0.0%	0	0.0%	0	0.0%
mmu-mir-92a-1-5p	14	115443698	115443718	+	edit	8	0	0.0%	0	0.0%	0	0.0%
mmu-mir-92a-1-5p	14	115443698	115443718	+	edit	9	0	0.0%	0	0.0%	0	0.0%
mmu-mir-92a-1-5p	14	115443698	115443718	+	edit	10	0	0.0%	0	0.0%	0	0.0%
mmu-mir-92a-1-5p	14	115443698	115443718	+	edit	11	0	0.0%	0	0.0%	0	0.0%
mmu-mir-92a-1-5p	14	115443698	115443718	+	edit	12	0	0.0%	0	0.0%	0	0.0%
mmu-mir-92a-1-5p	14	115443698	115443718	+	edit	13	0	0.0%	0	0.0%	0	0.0%
mmu-mir-92a-1-5p	14	115443698	115443718	+	edit	14	0	0.0%	0	0.0%	0	0.0%
mmu-mir-92a-1-5p	14	115443698	115443718	+	edit	15	0	0.0%	4	0.3%	0	0.0%
mmu-mir-92a-1-5p	14	115443698	115443718	+	edit	16	0	0.0%	0	0.0%	0	0.0%
mmu-mir-92a-1-5p	14	115443698	115443718	+	edit	17	0	0.0%	0	0.0%	0	0.0%
mmu-mir-92a-1-5p	14	115443698	115443718	+	edit	18	0	0.0%	0	0.0%	0	0.0%
mmu-mir-92a-1-5p	14	115443698	115443718	+	edit	19	46	2.5%	40	3.3%	31	3.1%
mmu-mir-92a-1-5p	14	115443698	115443718	+	edit	20	0	0.0%	0	0.0%	0	0.0%
mmu-mir-92a-1-5p	14	115443698	115443718	+	edit	21	0	0.0%	0	0.0%	0	0.0%
mmu-mir-92a-1-5p	14	115443698	115443718	+	edit	22	0	0.0%	0	0.0%	0	0.0%
EDITED							49	2.7%	44	3.5%	34	3.4%
CANONICAL							1794	97.3%	1185	96.5%	952	96.6%
TOTAL							1843		1229		986	

miRNA	Chr	Start	Stop	Strand	Type	Position	P7 reads	P7%	P10 reads	P10%	P14 reads	P14%
mmu-mir-92b-5p	3	89031048	89031069	-	edit	1	0	0.0%	0	0.0%	0	0.0%
mmu-mir-92b-5p	3	89031048	89031069	-	edit	2	0	0.0%	0	0.0%	0	0.0%
mmu-mir-92b-5p	3	89031048	89031069	-	edit	3	0	0.0%	0	0.0%	0	0.0%
mmu-mir-92b-5p	3	89031048	89031069	-	edit	4	0	0.0%	0	0.0%	0	0.0%
mmu-mir-92b-5p	3	89031048	89031069	-	edit	5	0	0.0%	0	0.0%	0	0.0%
mmu-mir-92b-5p	3	89031048	89031069	-	edit	6	0	0.0%	0	0.0%	0	0.0%
mmu-mir-92b-5p	3	89031048	89031069	-	edit	7	0	0.0%	0	0.0%	0	0.0%
mmu-mir-92b-5p	3	89031048	89031069	-	edit	8	0	0.0%	0	0.0%	0	0.0%
mmu-mir-92b-5p	3	89031048	89031069	-	edit	9	0	0.0%	0	0.0%	0	0.0%
mmu-mir-92b-5p	3	89031048	89031069	-	edit	10	0	0.0%	0	0.0%	0	0.0%
mmu-mir-92b-5p	3	89031048	89031069	-	edit	11	0	0.0%	0	0.0%	0	0.0%
mmu-mir-92b-5p	3	89031048	89031069	-	edit	12	0	0.0%	0	0.0%	0	0.0%
mmu-mir-92b-5p	3	89031048	89031069	-	edit	13	0	0.0%	0	0.0%	0	0.0%
mmu-mir-92b-5p	3	89031048	89031069	-	edit	14	0	0.0%	0	0.0%	0	0.0%
mmu-mir-92b-5p	3	89031048	89031069	-	edit	15	0	0.0%	0	0.0%	0	0.0%
mmu-mir-92b-5p	3	89031048	89031069	-	edit	16	0	0.0%	0	0.0%	0	0.0%
mmu-mir-92b-5p	3	89031048	89031069	-	edit	17	0	0.0%	0	0.0%	0	0.0%
mmu-mir-92b-5p	3	89031048	89031069	-	edit	18	0	0.0%	0	0.0%	0	0.0%
mmu-mir-92b-5p	3	89031048	89031069	-	edit	19	0	0.0%	0	0.0%	6	2.8%
mmu-mir-92b-5p	3	89031048	89031069	-	edit	20	0	0.0%	0	0.0%	0	0.0%
mmu-mir-92b-5p	3	89031048	89031069	-	edit	21	0	0.0%	0	0.0%	0	0.0%
mmu-mir-92b-5p	3	89031048	89031069	-	edit	22	0	0.0%	0	0.0%	0	0.0%
EDITED							0	0.0%	0	0.0%	6	2.8%
CANONICAL							208	100.0%	239	100.0%	206	97.2%
TOTAL							208		239		212	

miRNA	Chr	Start	Stop	Strand	Type	Position	P7 reads	P7%	P10 reads	P10%	P14 reads	P14%
mmu-mir-93-5p	5	138606802	138606824	-	edit	1	0	0.0%	0	0.0%	0	0.0%
mmu-mir-93-5p	5	138606802	138606824	-	edit	2	0	0.0%	0	0.0%	0	0.0%
mmu-mir-93-5p	5	138606802	138606824	-	edit	3	0	0.0%	0	0.0%	0	0.0%
mmu-mir-93-5p	5	138606802	138606824	-	edit	4	0	0.0%	0	0.0%	0	0.0%
mmu-mir-93-5p	5	138606802	138606824	-	edit	5	0	0.0%	0	0.0%	0	0.0%
mmu-mir-93-5p	5	138606802	138606824	-	edit	6	0	0.0%	0	0.0%	0	0.0%
mmu-mir-93-5p	5	138606802	138606824	-	edit	7	0	0.0%	0	0.0%	0	0.0%
mmu-mir-93-5p	5	138606802	138606824	-	edit	8	0	0.0%	0	0.0%	0	0.0%
mmu-mir-93-5p	5	138606802	138606824	-	edit	9	0	0.0%	0	0.0%	0	0.0%
mmu-mir-93-5p	5	138606802	138606824	-	edit	10	0	0.0%	0	0.0%	0	0.0%
mmu-mir-93-5p	5	138606802	138606824	-	edit	11	0	0.0%	0	0.0%	0	0.0%
mmu-mir-93-5p	5	138606802	138606824	-	edit	12	0	0.0%	0	0.0%	0	0.0%
mmu-mir-93-5p	5	138606802	138606824	-	edit	13	0	0.0%	0	0.0%	0	0.0%
mmu-mir-93-5p	5	138606802	138606824	-	edit	14	0	0.0%	0	0.0%	0	0.0%
mmu-mir-93-5p	5	138606802	138606824	-	edit	15	0	0.0%	0	0.0%	5	1.0%
mmu-mir-93-5p	5	138606802	138606824	-	edit	16	0	0.0%	0	0.0%	0	0.0%
mmu-mir-93-5p	5	138606802	138606824	-	edit	17	6	0.4%	8	1.1%	4	0.9%
mmu-mir-93-5p	5	138606802	138606824	-	edit	18	0	0.0%	4	0.5%	0	0.0%
mmu-mir-93-5p	5	138606802	138606824	-	edit	19	0	0.0%	0	0.0%	0	0.0%
mmu-mir-93-5p	5	138606802	138606824	-	edit	20	0	0.0%	0	0.0%	0	0.0%
mmu-mir-93-5p	5	138606802	138606824	-	edit	21	13	1.0%	6	0.8%	17	3.6%
mmu-mir-93-5p	5	138606802	138606824	-	edit	22	0	0.0%	0	0.0%	0	0.0%
EDITED							19	1.4%	18	2.4%	26	5.4%
CANONICAL							1330	98.6%	732	97.6%	444	94.6%
TOTAL							1349		750		470	

**miRNA**	**Chr**	**Start**	**Stop**	**Strand**	**Type**	**Position**	**P7 reads**	**P7%**	**P10 reads**	**P10%**	**P14 reads**	**P14%**
mmu-mir-98-5p	X	148347772	148347793	+	edit	1	0	0.0%	0	0.0%	0	0.0%
mmu-mir-98-5p	X	148347772	148347793	+	edit	2	0	0.0%	0	0.0%	0	0.0%
mmu-mir-98-5p	X	148347772	148347793	+	edit	3	0	0.0%	0	0.0%	0	0.0%
mmu-mir-98-5p	X	148347772	148347793	+	edit	4	0	0.0%	0	0.0%	0	0.0%
mmu-mir-98-5p	X	148347772	148347793	+	edit	5	0	0.0%	0	0.0%	0	0.0%
mmu-mir-98-5p	X	148347772	148347793	+	edit	6	0	0.0%	0	0.0%	0	0.0%
mmu-mir-98-5p	X	148347772	148347793	+	edit	7	0	0.0%	0	0.0%	0	0.0%
mmu-mir-98-5p	X	148347772	148347793	+	edit	8	0	0.0%	0	0.0%	0	0.0%
mmu-mir-98-5p	X	148347772	148347793	+	edit	9	0	0.0%	0	0.0%	0	0.0%
mmu-mir-98-5p	X	148347772	148347793	+	edit	10	0	0.0%	0	0.0%	0	0.0%
mmu-mir-98-5p	X	148347772	148347793	+	edit	11	21	1.0%	8	0.6%	9	1.0%
mmu-mir-98-5p	X	148347772	148347793	+	edit	12	0	0.0%	0	0.0%	0	0.0%
mmu-mir-98-5p	X	148347772	148347793	+	edit	13	0	0.0%	0	0.0%	0	0.0%
mmu-mir-98-5p	X	148347772	148347793	+	edit	14	0	0.0%	0	0.0%	0	0.0%
mmu-mir-98-5p	X	148347772	148347793	+	edit	15	0	0.0%	0	0.0%	0	0.0%
mmu-mir-98-5p	X	148347772	148347793	+	edit	16	0	0.0%	0	0.0%	0	0.0%
mmu-mir-98-5p	X	148347772	148347793	+	edit	17	0	0.0%	0	0.0%	0	0.0%
mmu-mir-98-5p	X	148347772	148347793	+	edit	18	0	0.0%	0	0.0%	0	0.0%
mmu-mir-98-5p	X	148347772	148347793	+	edit	19	**176**	**8.3%**	**78**	**5.8%**	**97**	**10.6%**
mmu-mir-98-5p	X	148347772	148347793	+	edit	20	0	0.0%	0	0.0%	0	0.0%
mmu-mir-98-5p	X	148347772	148347793	+	edit	21	0	0.0%	0	0.0%	0	0.0%
mmu-mir-98-5p	X	148347772	148347793	+	edit	22	0	0.0%	0	0.0%	0	0.0%
EDITED							197	9.3%	86	6.4%	106	11.6%
CANONICAL							1916	90.7%	1261	93.6%	806	88.4%
TOTAL							2113		1347		912	

miRNA	Chr	Start	Stop	Strand	Type	Position	P7 reads	P7%	P10 reads	P10%	P14 reads	P14%
mmu-mir-99a-5p	16	77599185	77599206	+	3' Edit	3'	3	0.0%	0	0.0%	3	0.1%
mmu-mir-99a-5p	16	77599185	77599206	+	edit	1	0	0.0%	0	0.0%	0	0.0%
mmu-mir-99a-5p	16	77599185	77599206	+	edit	2	0	0.0%	0	0.0%	0	0.0%
mmu-mir-99a-5p	16	77599185	77599206	+	edit	3	0	0.0%	0	0.0%	0	0.0%
mmu-mir-99a-5p	16	77599185	77599206	+	edit	4	3	0.0%	0	0.0%	6	0.2%
mmu-mir-99a-5p	16	77599185	77599206	+	edit	5	0	0.0%	0	0.0%	0	0.0%
mmu-mir-99a-5p	16	77599185	77599206	+	edit	6	10	0.1%	0	0.0%	0	0.0%
mmu-mir-99a-5p	16	77599185	77599206	+	edit	7	0	0.0%	0	0.0%	0	0.0%
mmu-mir-99a-5p	16	77599185	77599206	+	edit	8	9	0.1%	0	0.0%	0	0.0%
mmu-mir-99a-5p	16	77599185	77599206	+	edit	9	0	0.0%	0	0.0%	0	0.0%
mmu-mir-99a-5p	16	77599185	77599206	+	edit	10	0	0.0%	0	0.0%	0	0.0%
mmu-mir-99a-5p	16	77599185	77599206	+	edit	11	0	0.0%	0	0.0%	0	0.0%
mmu-mir-99a-5p	16	77599185	77599206	+	edit	12	0	0.0%	0	0.0%	0	0.0%
mmu-mir-99a-5p	16	77599185	77599206	+	edit	13	0	0.0%	0	0.0%	0	0.0%
mmu-mir-99a-5p	16	77599185	77599206	+	edit	14	0	0.0%	0	0.0%	0	0.0%
mmu-mir-99a-5p	16	77599185	77599206	+	edit	15	43	0.4%	113	1.8%	15	0.4%
mmu-mir-99a-5p	16	77599185	77599206	+	edit	16	0	0.0%	0	0.0%	0	0.0%
mmu-mir-99a-5p	16	77599185	77599206	+	edit	17	0	0.0%	0	0.0%	0	0.0%
mmu-mir-99a-5p	16	77599185	77599206	+	edit	18	0	0.0%	0	0.0%	0	0.0%
mmu-mir-99a-5p	16	77599185	77599206	+	edit	19	45	0.4%	28	0.4%	7	0.2%
EDITED							113	0.9%	141	2.2%	31	0.8%
CANONICAL							11936	99.1%	6245	97.8%	3743	99.2%
TOTAL							12049		6386		3774	

miRNA	Chr	Start	Stop	Strand	Type	Position	P7 reads	P7%	P10 reads	P10%	P14 reads	P14%
mmu-mir-99b-3p	17	17967196	17967217	+	3' Edit	3'	17	0.9%	0	0.0%	0	0.0%
mmu-mir-99b-3p	17	17967196	17967217	+	3' Indel	3'	9	0.5%	0	0.0%	0	0.0%
mmu-mir-99b-3p	17	17967196	17967217	+	Indel	22	8	0.4%	9	1.6%	3	1.0%
mmu-mir-99b-3p	17	17967196	17967217	+	edit	1	0	0.0%	0	0.0%	0	0.0%
mmu-mir-99b-3p	17	17967196	17967217	+	edit	2	0	0.0%	0	0.0%	0	0.0%
mmu-mir-99b-3p	17	17967196	17967217	+	edit	3	0	0.0%	0	0.0%	0	0.0%
mmu-mir-99b-3p	17	17967196	17967217	+	edit	4	0	0.0%	0	0.0%	0	0.0%
mmu-mir-99b-3p	17	17967196	17967217	+	edit	5	0	0.0%	0	0.0%	0	0.0%
mmu-mir-99b-3p	17	17967196	17967217	+	edit	6	0	0.0%	0	0.0%	0	0.0%
mmu-mir-99b-3p	17	17967196	17967217	+	edit	7	0	0.0%	0	0.0%	0	0.0%
mmu-mir-99b-3p	17	17967196	17967217	+	edit	8	3	0.2%	0	0.0%	0	0.0%
mmu-mir-99b-3p	17	17967196	17967217	+	edit	9	0	0.0%	0	0.0%	0	0.0%
mmu-mir-99b-3p	17	17967196	17967217	+	edit	10	0	0.0%	0	0.0%	0	0.0%
mmu-mir-99b-3p	17	17967196	17967217	+	edit	11	0	0.0%	0	0.0%	0	0.0%
mmu-mir-99b-3p	17	17967196	17967217	+	edit	12	0	0.0%	0	0.0%	0	0.0%
mmu-mir-99b-3p	17	17967196	17967217	+	edit	13	0	0.0%	0	0.0%	0	0.0%
mmu-mir-99b-3p	17	17967196	17967217	+	edit	14	0	0.0%	0	0.0%	0	0.0%
mmu-mir-99b-3p	17	17967196	17967217	+	edit	15	13	0.7%	7	1.3%	3	1.0%
mmu-mir-99b-3p	17	17967196	17967217	+	edit	16	0	0.0%	0	0.0%	0	0.0%
mmu-mir-99b-3p	17	17967196	17967217	+	edit	17	0	0.0%	0	0.0%	0	0.0%
mmu-mir-99b-3p	17	17967196	17967217	+	edit	18	0	0.0%	0	0.0%	0	0.0%
mmu-mir-99b-3p	17	17967196	17967217	+	edit	19	**98**	**5.4%**	**31**	**5.5%**	**29**	**10.1%**
mmu-mir-99b-3p	17	17967196	17967217	+	edit	20	0	0.0%	0	0.0%	0	0.0%
mmu-mir-99b-3p	17	17967196	17967217	+	edit	21	0	0.0%	0	0.0%	0	0.0%
mmu-mir-99b-3p	17	17967196	17967217	+	edit	22	0	0.0%	0	0.0%	0	0.0%
EDITED							147	8.1%	47	8.4%	35	12.2%
CANONICAL							1672	91.9%	509	91.6%	251	87.8%
TOTAL							1819		556		286	

miRNA	Chr	Start	Stop	Strand	Type	Position	P7 reads	P7%	P10 reads	P10%	P14 reads	P14%
mmu-mir-99b-5p	17	17967158	17967179	+	edit	1	0	0.0%	0	0.0%	0	0.0%
mmu-mir-99b-5p	17	17967158	17967179	+	edit	2	0	0.0%	0	0.0%	0	0.0%
mmu-mir-99b-5p	17	17967158	17967179	+	edit	3	0	0.0%	0	0.0%	0	0.0%
mmu-mir-99b-5p	17	17967158	17967179	+	edit	4	13	0.1%	18	0.4%	8	0.3%
mmu-mir-99b-5p	17	17967158	17967179	+	edit	5	0	0.0%	0	0.0%	0	0.0%
mmu-mir-99b-5p	17	17967158	17967179	+	edit	6	4	0.0%	3	0.1%	4	0.2%
mmu-mir-99b-5p	17	17967158	17967179	+	edit	7	0	0.0%	0	0.0%	0	0.0%
mmu-mir-99b-5p	17	17967158	17967179	+	edit	8	11	0.1%	0	0.0%	0	0.0%
mmu-mir-99b-5p	17	17967158	17967179	+	edit	9	0	0.0%	0	0.0%	0	0.0%
mmu-mir-99b-5p	17	17967158	17967179	+	edit	10	0	0.0%	0	0.0%	0	0.0%
mmu-mir-99b-5p	17	17967158	17967179	+	edit	11	0	0.0%	0	0.0%	0	0.0%
mmu-mir-99b-5p	17	17967158	17967179	+	edit	12	0	0.0%	0	0.0%	0	0.0%
mmu-mir-99b-5p	17	17967158	17967179	+	edit	13	0	0.0%	0	0.0%	0	0.0%
mmu-mir-99b-5p	17	17967158	17967179	+	edit	14	0	0.0%	0	0.0%	0	0.0%
mmu-mir-99b-5p	17	17967158	17967179	+	edit	15	40	0.3%	93	2.0%	6	0.2%
mmu-mir-99b-5p	17	17967158	17967179	+	edit	16	0	0.0%	0	0.0%	0	0.0%
mmu-mir-99b-5p	17	17967158	17967179	+	edit	17	0	0.0%	0	0.0%	0	0.0%
mmu-mir-99b-5p	17	17967158	17967179	+	edit	18	0	0.0%	0	0.0%	0	0.0%
mmu-mir-99b-5p	17	17967158	17967179	+	edit	19	71	0.6%	31	0.7%	16	0.6%
mmu-mir-99b-5p	17	17967158	17967179	+	edit	20	0	0.0%	0	0.0%	0	0.0%
mmu-mir-99b-5p	17	17967158	17967179	+	edit	21	0	0.0%	0	0.0%	0	0.0%
mmu-mir-99b-5p	17	17967158	17967179	+	edit	22	3	0.0%	0	0.0%	0	0.0%
EDITED							142	1.1%	145	3.0%	34	1.3%
CANONICAL							12436	98.9%	4621	97.0%	2500	98.7%
TOTAL							12578		4766		2534	

miRNA	Chr	Start	Stop	Strand	Type	Position	P7 reads	P7%	P10 reads	P10%	P14 reads	P14%
mmu-mir-100-5p	9	41339520	41339541	+	edit	1	0	0.0%	0	0.0%	0	0.0%
mmu-mir-100-5p	9	41339520	41339541	+	edit	2	0	0.0%	0	0.0%	0	0.0%
mmu-mir-100-5p	9	41339520	41339541	+	edit	3	0	0.0%	0	0.0%	0	0.0%
mmu-mir-100-5p	9	41339520	41339541	+	edit	4	0	0.0%	0	0.0%	0	0.0%
mmu-mir-100-5p	9	41339520	41339541	+	edit	5	0	0.0%	0	0.0%	0	0.0%
mmu-mir-100-5p	9	41339520	41339541	+	edit	6	0	0.0%	0	0.0%	0	0.0%
mmu-mir-100-5p	9	41339520	41339541	+	edit	7	0	0.0%	0	0.0%	0	0.0%
mmu-mir-100-5p	9	41339520	41339541	+	edit	8	0	0.0%	0	0.0%	0	0.0%
mmu-mir-100-5p	9	41339520	41339541	+	edit	9	0	0.0%	0	0.0%	0	0.0%
mmu-mir-100-5p	9	41339520	41339541	+	edit	10	0	0.0%	0	0.0%	0	0.0%
mmu-mir-100-5p	9	41339520	41339541	+	edit	11	0	0.0%	0	0.0%	0	0.0%
mmu-mir-100-5p	9	41339520	41339541	+	edit	12	0	0.0%	0	0.0%	0	0.0%
mmu-mir-100-5p	9	41339520	41339541	+	edit	13	0	0.0%	0	0.0%	0	0.0%
mmu-mir-100-5p	9	41339520	41339541	+	edit	14	0	0.0%	0	0.0%	0	0.0%
mmu-mir-100-5p	9	41339520	41339541	+	edit	15	0	0.0%	16	2.8%	4	0.8%
mmu-mir-100-5p	9	41339520	41339541	+	edit	16	4	0.6%	0	0.0%	0	0.0%
mmu-mir-100-5p	9	41339520	41339541	+	edit	17	0	0.0%	0	0.0%	0	0.0%
mmu-mir-100-5p	9	41339520	41339541	+	edit	18	0	0.0%	0	0.0%	0	0.0%
mmu-mir-100-5p	9	41339520	41339541	+	edit	19	3	0.5%	4	0.7%	0	0.0%
mmu-mir-100-5p	9	41339520	41339541	+	edit	20	0	0.0%	0	0.0%	0	0.0%
mmu-mir-100-5p	9	41339520	41339541	+	edit	21	0	0.0%	0	0.0%	0	0.0%
mmu-mir-100-5p	9	41339520	41339541	+	edit	22	0	0.0%	0	0.0%	0	0.0%
EDITED							7	1.0%	20	3.5%	4	0.8%
CANONICAL							620	99.0%	558	96.5%	449	99.2%
TOTAL							627		578		453	

miRNA	Chr	Start	Stop	Strand	Type	Position	P7 reads	P7%	P10 reads	P10%	P14 reads	P14%
mmu-mir-101a-3p	4	101019562	101019582	-	5' Edit	5'	0	0.0%	0	0.0%	4	0.1%
mmu-mir-101a-3p	4	101019562	101019582	-	Indel	21	0	0.0%	8	0.1%	4	0.1%
mmu-mir-101a-3p	4	101019562	101019582	-	edit	1	0	0.0%	0	0.0%	0	0.0%
mmu-mir-101a-3p	4	101019562	101019582	-	edit	2	0	0.0%	0	0.0%	0	0.0%
mmu-mir-101a-3p	4	101019562	101019582	-	edit	3	0	0.0%	0	0.0%	0	0.0%
mmu-mir-101a-3p	4	101019562	101019582	-	edit	4	0	0.0%	0	0.0%	0	0.0%
mmu-mir-101a-3p	4	101019562	101019582	-	edit	5	0	0.0%	0	0.0%	0	0.0%
mmu-mir-101a-3p	4	101019562	101019582	-	edit	6	0	0.0%	0	0.0%	0	0.0%
mmu-mir-101a-3p	4	101019562	101019582	-	edit	7	0	0.0%	0	0.0%	0	0.0%
mmu-mir-101a-3p	4	101019562	101019582	-	edit	8	0	0.0%	0	0.0%	0	0.0%
mmu-mir-101a-3p	4	101019562	101019582	-	edit	9	0	0.0%	0	0.0%	0	0.0%
mmu-mir-101a-3p	4	101019562	101019582	-	edit	10	0	0.0%	0	0.0%	0	0.0%
mmu-mir-101a-3p	4	101019562	101019582	-	edit	11	0	0.0%	0	0.0%	0	0.0%
mmu-mir-101a-3p	4	101019562	101019582	-	edit	12	0	0.0%	0	0.0%	0	0.0%
mmu-mir-101a-3p	4	101019562	101019582	-	edit	13	0	0.0%	0	0.0%	0	0.0%
mmu-mir-101a-3p	4	101019562	101019582	-	edit	14	0	0.0%	4	0.0%	5	0.1%
mmu-mir-101a-3p	4	101019562	101019582	-	edit	15	0	0.0%	22	0.3%	0	0.0%
mmu-mir-101a-3p	4	101019562	101019582	-	edit	16	0	0.0%	9	0.1%	0	0.0%
mmu-mir-101a-3p	4	101019562	101019582	-	edit	17	4	0.1%	0	0.0%	0	0.0%
mmu-mir-101a-3p	4	101019562	101019582	-	edit	18	10	0.1%	11	0.1%	10	0.2%
mmu-mir-101a-3p	4	101019562	101019582	-	edit	19	0	0.0%	0	0.0%	0	0.0%
mmu-mir-101a-3p	4	101019562	101019582	-	edit	20	0	0.0%	0	0.0%	0	0.0%
mmu-mir-101a-3p	4	101019562	101019582	-	edit	21	0	0.0%	6	0.1%	3	0.1%
mmu-mir-101a-3p	4	101019562	101019582	-	edit	22	0	0.0%	0	0.0%	0	0.0%
EDITED							14	0.2%	59	0.8%	25	0.5%
CANONICAL							7466	99.8%	7364	99.2%	4625	99.5%
TOTAL							7480		7423		4650	

miRNA	Chr	Start	Stop	Strand	Type	Position	P7 reads	P7%	P10 reads	P10%	P14 reads	P14%
mmu-mir-101b-5p	19	29209829	29209849	+	3' Edit	3'	6	0.1%	0	0.0%	5	0.1%
mmu-mir-101b-5p	19	29209829	29209849	+	Indel	20	4	0.1%	0	0.0%	13	0.4%
mmu-mir-101b-5p	19	29209829	29209849	+	edit	1	0	0.0%	0	0.0%	0	0.0%
mmu-mir-101b-5p	19	29209829	29209849	+	edit	2	0	0.0%	0	0.0%	0	0.0%
mmu-mir-101b-5p	19	29209829	29209849	+	edit	3	0	0.0%	0	0.0%	3	0.1%
mmu-mir-101b-5p	19	29209829	29209849	+	edit	4	0	0.0%	4	0.1%	0	0.0%
mmu-mir-101b-5p	19	29209829	29209849	+	edit	5	0	0.0%	0	0.0%	0	0.0%
mmu-mir-101b-5p	19	29209829	29209849	+	edit	6	3	0.1%	7	0.2%	12	0.4%
mmu-mir-101b-5p	19	29209829	29209849	+	edit	7	0	0.0%	0	0.0%	0	0.0%
mmu-mir-101b-5p	19	29209829	29209849	+	edit	8	0	0.0%	0	0.0%	0	0.0%
mmu-mir-101b-5p	19	29209829	29209849	+	edit	9	0	0.0%	0	0.0%	0	0.0%
mmu-mir-101b-5p	19	29209829	29209849	+	edit	10	0	0.0%	0	0.0%	0	0.0%
mmu-mir-101b-5p	19	29209829	29209849	+	edit	11	0	0.0%	0	0.0%	0	0.0%
mmu-mir-101b-5p	19	29209829	29209849	+	edit	12	0	0.0%	0	0.0%	0	0.0%
mmu-mir-101b-5p	19	29209829	29209849	+	edit	13	0	0.0%	0	0.0%	0	0.0%
mmu-mir-101b-5p	19	29209829	29209849	+	edit	14	0	0.0%	0	0.0%	0	0.0%
mmu-mir-101b-5p	19	29209829	29209849	+	edit	15	0	0.0%	11	0.3%	0	0.0%
mmu-mir-101b-5p	19	29209829	29209849	+	edit	16	0	0.0%	10	0.2%	7	0.2%
mmu-mir-101b-5p	19	29209829	29209849	+	edit	17	0	0.0%	0	0.0%	3	0.1%
mmu-mir-101b-5p	19	29209829	29209849	+	edit	18	4	0.1%	3	0.1%	5	0.2%
mmu-mir-101b-5p	19	29209829	29209849	+	edit	19	4	0.1%	3	0.1%	0	0.0%
mmu-mir-101b-5p	19	29209829	29209849	+	edit	20	0	0.0%	0	0.0%	0	0.0%
mmu-mir-101b-5p	19	29209829	29209849	+	edit	21	0	0.0%	0	0.0%	0	0.0%
mmu-mir-101b-5p	19	29209829	29209849	+	edit	22	0	0.0%	0	0.0%	0	0.0%
EDITED							21	0.4%	38	0.9%	47	1.5%
CANONICAL							4779	99.6%	4228	99.1%	3090	98.5%
TOTAL							4800		4266		3137	

miRNA	Chr	Start	Stop	Strand	Type	Position	P7 reads	P7%	P10 reads	P10%	P14 reads	P14%
mmu-mir-103-1-5p	11	35595949	35595971	+	3' Edit	3'	54	0.1%	0	0.0%	45	0.1%
mmu-mir-103-1-5p	11	35595949	35595971	+	3' Indel	3'	0	0.0%	0	0.0%	4	0.0%
mmu-mir-103-1-5p	11	35595949	35595971	+	Indel	21	5	0.0%	0	0.0%	7	0.0%
mmu-mir-103-1-5p	11	35595949	35595971	+	Indel	22	0	0.0%	0	0.0%	0	0.0%
mmu-mir-103-1-5p	11	35595949	35595971	+	Indel	23	0	0.0%	0	0.0%	3	0.0%
mmu-mir-103-1-5p	11	35595949	35595971	+	edit	1	0	0.0%	0	0.0%	0	0.0%
mmu-mir-103-1-5p	11	35595949	35595971	+	edit	2	0	0.0%	0	0.0%	0	0.0%
mmu-mir-103-1-5p	11	35595949	35595971	+	edit	3	0	0.0%	0	0.0%	0	0.0%
mmu-mir-103-1-5p	11	35595949	35595971	+	edit	4	10	0.0%	27	0.1%	6	0.0%
mmu-mir-103-1-5p	11	35595949	35595971	+	edit	5	0	0.0%	0	0.0%	0	0.0%
mmu-mir-103-1-5p	11	35595949	35595971	+	edit	6	34	0.1%	24	0.1%	30	0.1%
mmu-mir-103-1-5p	11	35595949	35595971	+	edit	7	0	0.0%	0	0.0%	0	0.0%
mmu-mir-103-1-5p	11	35595949	35595971	+	edit	8	59	0.1%	13	0.0%	15	0.0%
mmu-mir-103-1-5p	11	35595949	35595971	+	edit	9	0	0.0%	0	0.0%	0	0.0%
mmu-mir-103-1-5p	11	35595949	35595971	+	edit	10	0	0.0%	0	0.0%	0	0.0%
mmu-mir-103-1-5p	11	35595949	35595971	+	edit	11	0	0.0%	0	0.0%	0	0.0%
mmu-mir-103-1-5p	11	35595949	35595971	+	edit	12	0	0.0%	0	0.0%	0	0.0%
mmu-mir-103-1-5p	11	35595949	35595971	+	edit	13	0	0.0%	0	0.0%	3	0.0%
mmu-mir-103-1-5p	11	35595949	35595971	+	edit	14	0	0.0%	0	0.0%	0	0.0%
mmu-mir-103-1-5p	11	35595949	35595971	+	edit	15	125	0.2%	91	0.2%	81	0.2%
mmu-mir-103-1-5p	11	35595949	35595971	+	edit	16	78	0.1%	36	0.1%	16	0.0%
mmu-mir-103-1-5p	11	35595949	35595971	+	edit	17	8	0.0%	0	0.0%	6	0.0%
mmu-mir-103-1-5p	11	35595949	35595971	+	edit	18	0	0.0%	0	0.0%	0	0.0%
mmu-mir-103-1-5p	11	35595949	35595971	+	edit	19	339	0.6%	153	0.4%	299	0.9%
mmu-mir-103-1-5p	11	35595949	35595971	+	edit	20	0	0.0%	0	0.0%	0	0.0%
mmu-mir-103-1-5p	11	35595949	35595971	+	edit	21	44	0.1%	4	0.0%	35	0.1%
mmu-mir-103-1-5p	11	35595949	35595971	+	edit	22	6	0.0%	0	0.0%	7	0.0%
mmu-mir-103-1-5p	11	35595949	35595971	+	edit	23	14	0.0%	3	0.0%	18	0.1%
EDITED							774	1.4%	351	0.9%	574	1.7%
CANONICAL							54312	98.6%	38098	99.1%	32978	98.3%
TOTAL							55086		38449		33552	

miRNA	Chr	Start	Stop	Strand	Type	Position	P7 reads	P7%	P10 reads	P10%	P14 reads	P14%
mmu-mir-103-2-5p	2	131113839	131113861	+	3' Edit	3'	390	0.7%	0	0.0%	479	1.3%
mmu-mir-103-2-5p	2	131113839	131113861	+	3' Indel	3'	21	0.0%	0	0.0%	12	0.0%
mmu-mir-103-2-5p	2	131113839	131113861	+	Indel	20	4	0.0%	0	0.0%	0	0.0%
mmu-mir-103-2-5p	2	131113839	131113861	+	Indel	21	10	0.0%	0	0.0%	3	0.0%
mmu-mir-103-2-5p	2	131113839	131113861	+	Indel	22	0	0.0%	0	0.0%	0	0.0%
mmu-mir-103-2-5p	2	131113839	131113861	+	Indel	23	14	0.0%	0	0.0%	8	0.0%
mmu-mir-103-2-5p	2	131113839	131113861	+	edit	1	0	0.0%	0	0.0%	0	0.0%
mmu-mir-103-2-5p	2	131113839	131113861	+	edit	2	0	0.0%	0	0.0%	0	0.0%
mmu-mir-103-2-5p	2	131113839	131113861	+	edit	3	0	0.0%	0	0.0%	0	0.0%
mmu-mir-103-2-5p	2	131113839	131113861	+	edit	4	12	0.0%	30	0.1%	6	0.0%
mmu-mir-103-2-5p	2	131113839	131113861	+	edit	5	0	0.0%	0	0.0%	0	0.0%
mmu-mir-103-2-5p	2	131113839	131113861	+	edit	6	34	0.1%	24	0.1%	35	0.1%
mmu-mir-103-2-5p	2	131113839	131113861	+	edit	7	0	0.0%	0	0.0%	0	0.0%
mmu-mir-103-2-5p	2	131113839	131113861	+	edit	8	58	0.1%	14	0.0%	17	0.0%
mmu-mir-103-2-5p	2	131113839	131113861	+	edit	9	0	0.0%	0	0.0%	0	0.0%
mmu-mir-103-2-5p	2	131113839	131113861	+	edit	10	3	0.0%	0	0.0%	0	0.0%
mmu-mir-103-2-5p	2	131113839	131113861	+	edit	11	4	0.0%	0	0.0%	0	0.0%
mmu-mir-103-2-5p	2	131113839	131113861	+	edit	12	0	0.0%	0	0.0%	0	0.0%
mmu-mir-103-2-5p	2	131113839	131113861	+	edit	13	9	0.0%	3	0.0%	6	0.0%
mmu-mir-103-2-5p	2	131113839	131113861	+	edit	14	0	0.0%	0	0.0%	0	0.0%
mmu-mir-103-2-5p	2	131113839	131113861	+	edit	15	134	0.2%	96	0.2%	102	0.3%
mmu-mir-103-2-5p	2	131113839	131113861	+	edit	16	79	0.1%	36	0.1%	16	0.0%
mmu-mir-103-2-5p	2	131113839	131113861	+	edit	17	20	0.0%	3	0.0%	21	0.1%
mmu-mir-103-2-5p	2	131113839	131113861	+	edit	18	0	0.0%	0	0.0%	0	0.0%
mmu-mir-103-2-5p	2	131113839	131113861	+	edit	19	381	0.7%	159	0.4%	334	0.9%
mmu-mir-103-2-5p	2	131113839	131113861	+	edit	20	3	0.0%	0	0.0%	0	0.0%
mmu-mir-103-2-5p	2	131113839	131113861	+	edit	21	131	0.2%	15	0.0%	105	0.3%
mmu-mir-103-2-5p	2	131113839	131113861	+	edit	22	61	0.1%	14	0.0%	38	0.1%
mmu-mir-103-2-5p	2	131113839	131113861	+	edit	23	9	0.0%	0	0.0%	12	0.0%
EDITED							1375	2.4%	394	1.0%	1192	3.4%
CANONICAL							56517	97.6%	39005	99.0%	34378	96.6%
TOTAL							57892		39399		35570	

miRNA	Chr	Start	Stop	Strand	Type	Position	P7 reads	P7%	P10 reads	P10%	P14 reads	P14%
mmu-mir-106b-3p	5	138606974	138606995	-	edit	1	0	0.0%	0	0.0%	0	0.0%
mmu-mir-106b-3p	5	138606974	138606995	-	edit	2	0	0.0%	0	0.0%	0	0.0%
mmu-mir-106b-3p	5	138606974	138606995	-	edit	3	0	0.0%	0	0.0%	0	0.0%
mmu-mir-106b-3p	5	138606974	138606995	-	edit	4	0	0.0%	0	0.0%	0	0.0%
mmu-mir-106b-3p	5	138606974	138606995	-	edit	5	0	0.0%	0	0.0%	0	0.0%
mmu-mir-106b-3p	5	138606974	138606995	-	edit	6	0	0.0%	0	0.0%	0	0.0%
mmu-mir-106b-3p	5	138606974	138606995	-	edit	7	0	0.0%	0	0.0%	0	0.0%
mmu-mir-106b-3p	5	138606974	138606995	-	edit	8	0	0.0%	0	0.0%	0	0.0%
mmu-mir-106b-3p	5	138606974	138606995	-	edit	9	0	0.0%	0	0.0%	0	0.0%
mmu-mir-106b-3p	5	138606974	138606995	-	edit	10	0	0.0%	0	0.0%	0	0.0%
mmu-mir-106b-3p	5	138606974	138606995	-	edit	11	0	0.0%	0	0.0%	0	0.0%
mmu-mir-106b-3p	5	138606974	138606995	-	edit	12	0	0.0%	0	0.0%	0	0.0%
mmu-mir-106b-3p	5	138606974	138606995	-	edit	13	4	0.9%	0	0.0%	0	0.0%
mmu-mir-106b-3p	5	138606974	138606995	-	edit	14	0	0.0%	0	0.0%	0	0.0%
mmu-mir-106b-3p	5	138606974	138606995	-	edit	15	8	1.8%	0	0.0%	0	0.0%
mmu-mir-106b-3p	5	138606974	138606995	-	edit	16	0	0.0%	0	0.0%	0	0.0%
mmu-mir-106b-3p	5	138606974	138606995	-	edit	17	0	0.0%	0	0.0%	0	0.0%
mmu-mir-106b-3p	5	138606974	138606995	-	edit	18	0	0.0%	0	0.0%	0	0.0%
mmu-mir-106b-3p	5	138606974	138606995	-	edit	19	0	0.0%	4	2.3%	0	0.0%
mmu-mir-106b-3p	5	138606974	138606995	-	edit	20	0	0.0%	0	0.0%	0	0.0%
mmu-mir-106b-3p	5	138606974	138606995	-	edit	21	0	0.0%	0	0.0%	0	0.0%
mmu-mir-106b-3p	5	138606974	138606995	-	edit	22	0	0.0%	0	0.0%	0	0.0%
EDITED							12	2.7%	4	2.3%	0	0.0%
CANONICAL							417	97.3%	170	97.7%	116	100.0%
TOTAL							429		174		116	

miRNA	Chr	Start	Stop	Strand	Type	Position	P7 reads	P7%	P10 reads	P10%	P14 reads	P14%
mmu-mir-107-5p	19	34895190	34895212	-	5' Edit	5'	23	0.1%	0	0.0%	18	0.1%
mmu-mir-107-5p	19	34895190	34895212	-	Indel	21	8	0.0%	0	0.0%	3	0.0%
mmu-mir-107-5p	19	34895190	34895212	-	Indel	22	19	0.0%	0	0.0%	25	0.1%
mmu-mir-107-5p	19	34895190	34895212	-	Indel	23	6	0.0%	0	0.0%	0	0.0%
mmu-mir-107-5p	19	34895190	34895212	-	edit	1	0	0.0%	0	0.0%	0	0.0%
mmu-mir-107-5p	19	34895190	34895212	-	edit	2	0	0.0%	0	0.0%	0	0.0%
mmu-mir-107-5p	19	34895190	34895212	-	edit	3	0	0.0%	0	0.0%	0	0.0%
mmu-mir-107-5p	19	34895190	34895212	-	edit	4	0	0.0%	8	0.0%	0	0.0%
mmu-mir-107-5p	19	34895190	34895212	-	edit	5	4	0.0%	5	0.0%	0	0.0%
mmu-mir-107-5p	19	34895190	34895212	-	edit	6	29	0.1%	14	0.1%	14	0.1%
mmu-mir-107-5p	19	34895190	34895212	-	edit	7	0	0.0%	0	0.0%	0	0.0%
mmu-mir-107-5p	19	34895190	34895212	-	edit	8	7	0.0%	0	0.0%	0	0.0%
mmu-mir-107-5p	19	34895190	34895212	-	edit	9	0	0.0%	0	0.0%	0	0.0%
mmu-mir-107-5p	19	34895190	34895212	-	edit	10	0	0.0%	0	0.0%	0	0.0%
mmu-mir-107-5p	19	34895190	34895212	-	edit	11	0	0.0%	0	0.0%	0	0.0%
mmu-mir-107-5p	19	34895190	34895212	-	edit	12	0	0.0%	0	0.0%	0	0.0%
mmu-mir-107-5p	19	34895190	34895212	-	edit	13	3	0.0%	0	0.0%	5	0.0%
mmu-mir-107-5p	19	34895190	34895212	-	edit	14	0	0.0%	0	0.0%	0	0.0%
mmu-mir-107-5p	19	34895190	34895212	-	edit	15	72	0.2%	53	0.2%	47	0.3%
mmu-mir-107-5p	19	34895190	34895212	-	edit	16	73	0.2%	29	0.1%	9	0.0%
mmu-mir-107-5p	19	34895190	34895212	-	edit	17	71	0.2%	24	0.1%	32	0.2%
mmu-mir-107-5p	19	34895190	34895212	-	edit	18	95	0.2%	105	0.4%	68	0.4%
mmu-mir-107-5p	19	34895190	34895212	-	edit	19	120	0.3%	38	0.1%	67	0.4%
mmu-mir-107-5p	19	34895190	34895212	-	edit	20	21	0.1%	68	0.3%	14	0.1%
mmu-mir-107-5p	19	34895190	34895212	-	edit	21	44	0.1%	3	0.0%	15	0.1%
mmu-mir-107-5p	19	34895190	34895212	-	edit	22	26	0.1%	6	0.0%	17	0.1%
EDITED							621	1.6%	352	1.3%	333	1.9%
CANONICAL							39174	98.4%	26183	98.7%	17078	98.1%
TOTAL							39795		26535		17411	

miRNA	Chr	Start	Stop	Strand	Type	Position	P7 reads	P7%	P10 reads	P10%	P14 reads	P14%
mmu-mir-122-5p	18	65408520	65408541	+	edit	1	0	0.0%	0	0.0%	0	0.0%
mmu-mir-122-5p	18	65408520	65408541	+	edit	2	0	0.0%	0	0.0%	0	0.0%
mmu-mir-122-5p	18	65408520	65408541	+	edit	3	0	0.0%	0	0.0%	0	0.0%
mmu-mir-122-5p	18	65408520	65408541	+	edit	4	0	0.0%	0	0.0%	0	0.0%
mmu-mir-122-5p	18	65408520	65408541	+	edit	5	0	0.0%	0	0.0%	0	0.0%
mmu-mir-122-5p	18	65408520	65408541	+	edit	6	0	0.0%	0	0.0%	0	0.0%
mmu-mir-122-5p	18	65408520	65408541	+	edit	7	0	0.0%	0	0.0%	0	0.0%
mmu-mir-122-5p	18	65408520	65408541	+	edit	8	3	0.4%	0	0.0%	0	0.0%
mmu-mir-122-5p	18	65408520	65408541	+	edit	9	0	0.0%	0	0.0%	0	0.0%
mmu-mir-122-5p	18	65408520	65408541	+	edit	10	0	0.0%	0	0.0%	0	0.0%
mmu-mir-122-5p	18	65408520	65408541	+	edit	11	0	0.0%	0	0.0%	0	0.0%
mmu-mir-122-5p	18	65408520	65408541	+	edit	12	0	0.0%	0	0.0%	0	0.0%
mmu-mir-122-5p	18	65408520	65408541	+	edit	13	0	0.0%	0	0.0%	0	0.0%
mmu-mir-122-5p	18	65408520	65408541	+	edit	14	0	0.0%	0	0.0%	0	0.0%
mmu-mir-122-5p	18	65408520	65408541	+	edit	15	0	0.0%	0	0.0%	0	0.0%
mmu-mir-122-5p	18	65408520	65408541	+	edit	16	0	0.0%	0	0.0%	0	0.0%
mmu-mir-122-5p	18	65408520	65408541	+	edit	17	0	0.0%	0	0.0%	0	0.0%
mmu-mir-122-5p	18	65408520	65408541	+	edit	18	0	0.0%	0	0.0%	0	0.0%
mmu-mir-122-5p	18	65408520	65408541	+	edit	19	7	0.9%	0	0.0%	0	0.0%
mmu-mir-122-5p	18	65408520	65408541	+	edit	20	0	0.0%	0	0.0%	0	0.0%
mmu-mir-122-5p	18	65408520	65408541	+	edit	21	0	0.0%	0	0.0%	0	0.0%
mmu-mir-122-5p	18	65408520	65408541	+	edit	22	8	1.1%	0	0.0%	0	0.0%
EDITED							18	2.4%	0	0.0%	0	0.0%
CANONICAL							739	97.6%	25	100.0%	6	100.0%
TOTAL							757		25		6	

miRNA	Chr	Start	Stop	Strand	Type	Position	P7 reads	P7%	P10 reads	P10%	P14 reads	P14%
mmu-mir-125a-5p	17	17967781	17967804	+	edit	1	0	0.0%	0	0.0%	0	0.0%
mmu-mir-125a-5p	17	17967781	17967804	+	edit	2	0	0.0%	0	0.0%	0	0.0%
mmu-mir-125a-5p	17	17967781	17967804	+	edit	3	0	0.0%	0	0.0%	0	0.0%
mmu-mir-125a-5p	17	17967781	17967804	+	edit	4	0	0.0%	0	0.0%	0	0.0%
mmu-mir-125a-5p	17	17967781	17967804	+	edit	5	0	0.0%	0	0.0%	0	0.0%
mmu-mir-125a-5p	17	17967781	17967804	+	edit	6	0	0.0%	3	0.1%	0	0.0%
mmu-mir-125a-5p	17	17967781	17967804	+	edit	7	0	0.0%	0	0.0%	0	0.0%
mmu-mir-125a-5p	17	17967781	17967804	+	edit	8	3	0.1%	0	0.0%	0	0.0%
mmu-mir-125a-5p	17	17967781	17967804	+	edit	9	0	0.0%	0	0.0%	0	0.0%
mmu-mir-125a-5p	17	17967781	17967804	+	edit	10	0	0.0%	0	0.0%	0	0.0%
mmu-mir-125a-5p	17	17967781	17967804	+	edit	11	0	0.0%	0	0.0%	0	0.0%
mmu-mir-125a-5p	17	17967781	17967804	+	edit	12	0	0.0%	0	0.0%	0	0.0%
mmu-mir-125a-5p	17	17967781	17967804	+	edit	13	0	0.0%	0	0.0%	0	0.0%
mmu-mir-125a-5p	17	17967781	17967804	+	edit	14	0	0.0%	0	0.0%	0	0.0%
mmu-mir-125a-5p	17	17967781	17967804	+	edit	15	9	0.2%	0	0.0%	0	0.0%
mmu-mir-125a-5p	17	17967781	17967804	+	edit	16	0	0.0%	0	0.0%	0	0.0%
mmu-mir-125a-5p	17	17967781	17967804	+	edit	17	0	0.0%	7	0.3%	0	0.0%
mmu-mir-125a-5p	17	17967781	17967804	+	edit	18	0	0.0%	0	0.0%	0	0.0%
mmu-mir-125a-5p	17	17967781	17967804	+	edit	19	33	0.8%	12	0.5%	7	0.8%
mmu-mir-125a-5p	17	17967781	17967804	+	edit	20	0	0.0%	0	0.0%	0	0.0%
mmu-mir-125a-5p	17	17967781	17967804	+	edit	21	0	0.0%	0	0.0%	0	0.0%
mmu-mir-125a-5p	17	17967781	17967804	+	edit	22	0	0.0%	0	0.0%	0	0.0%
							45	1.1%	22	0.9%	7	0.8%
							4081	98.9%	2253	99.1%	782	99.2%
							4126		2275		789	

miRNA	Chr	Start	Stop	Strand	Type	Position	P7 reads	P7%	P10 reads	P10%	P14 reads	P14%
mmu-mir-125b-1-5p	9	41390023	41390044	+	3' Indel	3'	17	0.5%	0	0.0%	15	1.2%
mmu-mir-125b-1-5p	9	41390023	41390044	+	edit	1	0	0.0%	0	0.0%	0	0.0%
mmu-mir-125b-1-5p	9	41390023	41390044	+	edit	2	0	0.0%	0	0.0%	0	0.0%
mmu-mir-125b-1-5p	9	41390023	41390044	+	edit	3	0	0.0%	0	0.0%	0	0.0%
mmu-mir-125b-1-5p	9	41390023	41390044	+	edit	4	3	0.1%	0	0.0%	0	0.0%
mmu-mir-125b-1-5p	9	41390023	41390044	+	edit	5	0	0.0%	0	0.0%	0	0.0%
mmu-mir-125b-1-5p	9	41390023	41390044	+	edit	6	0	0.0%	0	0.0%	0	0.0%
mmu-mir-125b-1-5p	9	41390023	41390044	+	edit	7	0	0.0%	0	0.0%	0	0.0%
mmu-mir-125b-1-5p	9	41390023	41390044	+	edit	8	0	0.0%	0	0.0%	0	0.0%
mmu-mir-125b-1-5p	9	41390023	41390044	+	edit	9	0	0.0%	0	0.0%	0	0.0%
mmu-mir-125b-1-5p	9	41390023	41390044	+	edit	10	0	0.0%	0	0.0%	0	0.0%
mmu-mir-125b-1-5p	9	41390023	41390044	+	edit	11	0	0.0%	0	0.0%	0	0.0%
mmu-mir-125b-1-5p	9	41390023	41390044	+	edit	12	0	0.0%	0	0.0%	0	0.0%
mmu-mir-125b-1-5p	9	41390023	41390044	+	edit	13	0	0.0%	0	0.0%	0	0.0%
mmu-mir-125b-1-5p	9	41390023	41390044	+	edit	14	0	0.0%	0	0.0%	0	0.0%
mmu-mir-125b-1-5p	9	41390023	41390044	+	edit	15	0	0.0%	18	0.7%	0	0.0%
mmu-mir-125b-1-5p	9	41390023	41390044	+	edit	16	0	0.0%	0	0.0%	0	0.0%
mmu-mir-125b-1-5p	9	41390023	41390044	+	edit	17	0	0.0%	0	0.0%	0	0.0%
mmu-mir-125b-1-5p	9	41390023	41390044	+	edit	18	0	0.0%	0	0.0%	0	0.0%
mmu-mir-125b-1-5p	9	41390023	41390044	+	edit	19	7	0.2%	0	0.0%	0	0.0%
mmu-mir-125b-1-5p	9	41390023	41390044	+	edit	20	0	0.0%	0	0.0%	0	0.0%
mmu-mir-125b-1-5p	9	41390023	41390044	+	edit	21	0	0.0%	0	0.0%	0	0.0%
mmu-mir-125b-1-5p	9	41390023	41390044	+	edit	22	9	0.3%	0	0.0%	6	0.5%
EDITED							36	1.1%	18	0.7%	21	1.7%
CANONICAL							3329	98.9%	2483	99.3%	1237	98.3%
TOTAL							3365		2501		1258	

miRNA	Chr	Start	Stop	Strand	Type	Position	P7 reads	P7%	P10 reads	P10%	P14 reads	P14%
mmu-mir-125b-2-5p	16	77646524	77646545	+	Indel	22	6	0.2%	6	0.2%	0	0.0%
mmu-mir-125b-2-5p	16	77646524	77646545	+	edit	1	0	0.0%	0	0.0%	0	0.0%
mmu-mir-125b-2-5p	16	77646524	77646545	+	edit	2	0	0.0%	0	0.0%	0	0.0%
mmu-mir-125b-2-5p	16	77646524	77646545	+	edit	3	0	0.0%	0	0.0%	0	0.0%
mmu-mir-125b-2-5p	16	77646524	77646545	+	edit	4	3	0.1%	0	0.0%	0	0.0%
mmu-mir-125b-2-5p	16	77646524	77646545	+	edit	5	0	0.0%	0	0.0%	0	0.0%
mmu-mir-125b-2-5p	16	77646524	77646545	+	edit	6	0	0.0%	0	0.0%	0	0.0%
mmu-mir-125b-2-5p	16	77646524	77646545	+	edit	7	0	0.0%	0	0.0%	0	0.0%
mmu-mir-125b-2-5p	16	77646524	77646545	+	edit	8	0	0.0%	0	0.0%	0	0.0%
mmu-mir-125b-2-5p	16	77646524	77646545	+	edit	9	0	0.0%	0	0.0%	0	0.0%
mmu-mir-125b-2-5p	16	77646524	77646545	+	edit	10	0	0.0%	0	0.0%	0	0.0%
mmu-mir-125b-2-5p	16	77646524	77646545	+	edit	11	0	0.0%	0	0.0%	0	0.0%
mmu-mir-125b-2-5p	16	77646524	77646545	+	edit	12	0	0.0%	0	0.0%	0	0.0%
mmu-mir-125b-2-5p	16	77646524	77646545	+	edit	13	0	0.0%	0	0.0%	0	0.0%
mmu-mir-125b-2-5p	16	77646524	77646545	+	edit	14	0	0.0%	0	0.0%	0	0.0%
mmu-mir-125b-2-5p	16	77646524	77646545	+	edit	15	0	0.0%	14	0.6%	0	0.0%
mmu-mir-125b-2-5p	16	77646524	77646545	+	edit	16	0	0.0%	0	0.0%	0	0.0%
mmu-mir-125b-2-5p	16	77646524	77646545	+	edit	17	0	0.0%	0	0.0%	0	0.0%
mmu-mir-125b-2-5p	16	77646524	77646545	+	edit	18	0	0.0%	0	0.0%	0	0.0%
mmu-mir-125b-2-5p	16	77646524	77646545	+	edit	19	4	0.1%	0	0.0%	0	0.0%
mmu-mir-125b-2-5p	16	77646524	77646545	+	edit	20	0	0.0%	0	0.0%	0	0.0%
mmu-mir-125b-2-5p	16	77646524	77646545	+	edit	21	0	0.0%	0	0.0%	0	0.0%
mmu-mir-125b-2-5p	16	77646524	77646545	+	edit	22	0	0.0%	3	0.1%	3	0.3%
EDITED							12	0.4%	36	1.6%	36	3.4%
CANONICAL							2922	99.6%	2195	98.4%	1028	96.6%
TOTAL							2934		2231		1064	

miRNA	Chr	Start	Stop	Strand	Type	Position	P7 reads	P7%	P10 reads	P10%	P14 reads	P14%
mmu-mir-127-3p	12	110831098	110831119	+	3' Edit	3'	136	0.5%	0	0.0%	19	0.6%
mmu-mir-127-3p	12	110831098	110831119	+	3' Indel	3'	4	0.0%	0	0.0%	0	0.0%
mmu-mir-127-3p	12	110831098	110831119	+	5' Indel	5'	3	0.0%	0	0.0%	0	0.0%
mmu-mir-127-3p	12	110831098	110831119	+	Indel	20	3	0.0%	0	0.0%	0	0.0%
mmu-mir-127-3p	12	110831098	110831119	+	Indel	21	8	0.0%	9	0.1%	4	0.1%
mmu-mir-127-3p	12	110831098	110831119	+	Indel	22	13	0.0%	0	0.0%	0	0.0%
mmu-mir-127-3p	12	110831098	110831119	+	edit	1	0	0.0%	0	0.0%	0	0.0%
mmu-mir-127-3p	12	110831098	110831119	+	edit	2	0	0.0%	0	0.0%	0	0.0%
mmu-mir-127-3p	12	110831098	110831119	+	edit	3	0	0.0%	0	0.0%	0	0.0%
mmu-mir-127-3p	12	110831098	110831119	+	edit	4	3	0.0%	0	0.0%	0	0.0%
mmu-mir-127-3p	12	110831098	110831119	+	edit	5	0	0.0%	0	0.0%	0	0.0%
mmu-mir-127-3p	12	110831098	110831119	+	edit	6	10	0.0%	0	0.0%	0	0.0%
mmu-mir-127-3p	12	110831098	110831119	+	edit	7	0	0.0%	0	0.0%	0	0.0%
mmu-mir-127-3p	12	110831098	110831119	+	edit	8	24	0.1%	7	0.1%	0	0.0%
mmu-mir-127-3p	12	110831098	110831119	+	edit	9	0	0.0%	0	0.0%	0	0.0%
mmu-mir-127-3p	12	110831098	110831119	+	edit	10	0	0.0%	0	0.0%	0	0.0%
mmu-mir-127-3p	12	110831098	110831119	+	edit	11	0	0.0%	0	0.0%	0	0.0%
mmu-mir-127-3p	12	110831098	110831119	+	edit	12	0	0.0%	0	0.0%	0	0.0%
mmu-mir-127-3p	12	110831098	110831119	+	edit	13	0	0.0%	0	0.0%	0	0.0%
mmu-mir-127-3p	12	110831098	110831119	+	edit	14	0	0.0%	0	0.0%	0	0.0%
mmu-mir-127-3p	12	110831098	110831119	+	edit	15	39	0.1%	15	0.1%	0	0.0%
mmu-mir-127-3p	12	110831098	110831119	+	edit	16	0	0.0%	0	0.0%	0	0.0%
mmu-mir-127-3p	12	110831098	110831119	+	edit	17	0	0.0%	0	0.0%	0	0.0%
mmu-mir-127-3p	12	110831098	110831119	+	edit	18	0	0.0%	0	0.0%	0	0.0%
mmu-mir-127-3p	12	110831098	110831119	+	edit	19	65	0.2%	16	0.2%	4	0.1%
mmu-mir-127-3p	12	110831098	110831119	+	edit	20	0	0.0%	0	0.0%	0	0.0%
mmu-mir-127-3p	12	110831098	110831119	+	edit	21	6	0.0%	0	0.0%	3	0.1%
mmu-mir-127-3p	12	110831098	110831119	+	edit	22	0	0.0%	0	0.0%	0	0.0%
EDITED							314	1.1%	47	0.5%	30	1.0%
CANONICAL							27461	98.9%	10153	99.5%	3109	99.0%
TOTAL							27775		10200		3139	

miRNA	Chr	Start	Stop	Strand	Type	Position	P7 reads	P7%	P10 reads	P10%	P14 reads	P14%
mmu-mir-127-5p	12	110831064	110831085	+	edit	1	0	0.0%	0	0.0%	0	0.0%
mmu-mir-127-5p	12	110831064	110831085	+	edit	2	0	0.0%	0	0.0%	0	0.0%
mmu-mir-127-5p	12	110831064	110831085	+	edit	3	0	0.0%	0	0.0%	0	0.0%
mmu-mir-127-5p	12	110831064	110831085	+	edit	4	0	0.0%	0	0.0%	0	0.0%
mmu-mir-127-5p	12	110831064	110831085	+	edit	5	0	0.0%	0	0.0%	0	0.0%
mmu-mir-127-5p	12	110831064	110831085	+	edit	6	0	0.0%	0	0.0%	0	0.0%
mmu-mir-127-5p	12	110831064	110831085	+	edit	7	0	0.0%	0	0.0%	0	0.0%
mmu-mir-127-5p	12	110831064	110831085	+	edit	8	0	0.0%	0	0.0%	0	0.0%
mmu-mir-127-5p	12	110831064	110831085	+	edit	9	0	0.0%	0	0.0%	0	0.0%
mmu-mir-127-5p	12	110831064	110831085	+	edit	10	0	0.0%	0	0.0%	0	0.0%
mmu-mir-127-5p	12	110831064	110831085	+	edit	11	0	0.0%	0	0.0%	0	0.0%
mmu-mir-127-5p	12	110831064	110831085	+	edit	12	0	0.0%	0	0.0%	0	0.0%
mmu-mir-127-5p	12	110831064	110831085	+	edit	13	0	0.0%	0	0.0%	0	0.0%
mmu-mir-127-5p	12	110831064	110831085	+	edit	14	0	0.0%	0	0.0%	0	0.0%
mmu-mir-127-5p	12	110831064	110831085	+	edit	15	0	0.0%	0	0.0%	0	0.0%
mmu-mir-127-5p	12	110831064	110831085	+	edit	16	0	0.0%	0	0.0%	0	0.0%
mmu-mir-127-5p	12	110831064	110831085	+	edit	17	0	0.0%	0	0.0%	0	0.0%
mmu-mir-127-5p	12	110831064	110831085	+	edit	18	0	0.0%	0	0.0%	0	0.0%
mmu-mir-127-5p	12	110831064	110831085	+	edit	19	0	0.0%	0	0.0%	0	0.0%
mmu-mir-127-5p	12	110831064	110831085	+	edit	20	0	0.0%	0	0.0%	0	0.0%
mmu-mir-127-5p	12	110831064	110831085	+	edit	21	3	0.5%	0	0.0%	0	0.0%
mmu-mir-127-5p	12	110831064	110831085	+	edit	22	0	0.0%	0	0.0%	0	0.0%
EDITED							3	0.5%	0	0.0%	0	0.0%
CANONICAL							582	99.5%	134	100.0%	58	100.0%
TOTAL							585		134		58	

miRNA	Chr	Start	Stop	Strand	Type	Position	P7 reads	P7%	P10 reads	P10%	P14 reads	P14%
mmu-mir-128-1-5p	1	130098981	130099001	+	edit	1	0	0.0%	0	0.0%	0	0.0%
mmu-mir-128-1-5p	1	130098981	130099001	+	edit	2	0	0.0%	0	0.0%	0	0.0%
mmu-mir-128-1-5p	1	130098981	130099001	+	edit	3	0	0.0%	0	0.0%	0	0.0%
mmu-mir-128-1-5p	1	130098981	130099001	+	edit	4	0	0.0%	0	0.0%	0	0.0%
mmu-mir-128-1-5p	1	130098981	130099001	+	edit	5	0	0.0%	0	0.0%	0	0.0%
mmu-mir-128-1-5p	1	130098981	130099001	+	edit	6	0	0.0%	0	0.0%	0	0.0%
mmu-mir-128-1-5p	1	130098981	130099001	+	edit	7	0	0.0%	0	0.0%	0	0.0%
mmu-mir-128-1-5p	1	130098981	130099001	+	edit	8	0	0.0%	0	0.0%	0	0.0%
mmu-mir-128-1-5p	1	130098981	130099001	+	edit	9	0	0.0%	0	0.0%	0	0.0%
mmu-mir-128-1-5p	1	130098981	130099001	+	edit	10	0	0.0%	0	0.0%	0	0.0%
mmu-mir-128-1-5p	1	130098981	130099001	+	edit	11	0	0.0%	0	0.0%	0	0.0%
mmu-mir-128-1-5p	1	130098981	130099001	+	edit	12	0	0.0%	0	0.0%	0	0.0%
mmu-mir-128-1-5p	1	130098981	130099001	+	edit	13	0	0.0%	0	0.0%	0	0.0%
mmu-mir-128-1-5p	1	130098981	130099001	+	edit	14	0	0.0%	0	0.0%	0	0.0%
mmu-mir-128-1-5p	1	130098981	130099001	+	edit	15	0	0.0%	4	0.5%	0	0.0%
mmu-mir-128-1-5p	1	130098981	130099001	+	edit	16	0	0.0%	4	0.6%	4	1.0%
mmu-mir-128-1-5p	1	130098981	130099001	+	edit	17	0	0.0%	0	0.0%	0	0.0%
mmu-mir-128-1-5p	1	130098981	130099001	+	edit	18	0	0.0%	0	0.0%	0	0.0%
mmu-mir-128-1-5p	1	130098981	130099001	+	edit	19	0	0.0%	0	0.0%	0	0.0%
mmu-mir-128-1-5p	1	130098981	130099001	+	edit	20	0	0.0%	0	0.0%	0	0.0%
mmu-mir-128-1-5p	1	130098981	130099001	+	edit	21	0	0.0%	0	0.0%	0	0.0%
mmu-mir-128-1-5p	1	130098981	130099001	+	edit	22	0	0.0%	0	0.0%	0	0.0%
EDITED							0	0.0%	8	1.2%	4	1.0%
CANONICAL							716	100.0%	631	98.8%	385	99.0%
TOTAL							716		639		389	

miRNA	Chr	Start	Stop	Strand	Type	Position	P7 reads	P7%	P10 reads	P10%	P14 reads	P14%
mmu-mir-128-2-5p	9	112021148	112021168	-	edit	1	0	0.0%	0	0.0%	0	0.0%
mmu-mir-128-2-5p	9	112021148	112021168	-	edit	2	0	0.0%	0	0.0%	0	0.0%
mmu-mir-128-2-5p	9	112021148	112021168	-	edit	3	0	0.0%	0	0.0%	0	0.0%
mmu-mir-128-2-5p	9	112021148	112021168	-	edit	4	0	0.0%	0	0.0%	0	0.0%
mmu-mir-128-2-5p	9	112021148	112021168	-	edit	5	0	0.0%	0	0.0%	0	0.0%
mmu-mir-128-2-5p	9	112021148	112021168	-	edit	6	0	0.0%	0	0.0%	0	0.0%
mmu-mir-128-2-5p	9	112021148	112021168	-	edit	7	0	0.0%	0	0.0%	0	0.0%
mmu-mir-128-2-5p	9	112021148	112021168	-	edit	8	0	0.0%	0	0.0%	0	0.0%
mmu-mir-128-2-5p	9	112021148	112021168	-	edit	9	0	0.0%	0	0.0%	0	0.0%
mmu-mir-128-2-5p	9	112021148	112021168	-	edit	10	0	0.0%	0	0.0%	0	0.0%
mmu-mir-128-2-5p	9	112021148	112021168	-	edit	11	0	0.0%	0	0.0%	0	0.0%
mmu-mir-128-2-5p	9	112021148	112021168	-	edit	12	0	0.0%	0	0.0%	0	0.0%
mmu-mir-128-2-5p	9	112021148	112021168	-	edit	13	0	0.0%	0	0.0%	0	0.0%
mmu-mir-128-2-5p	9	112021148	112021168	-	edit	14	0	0.0%	0	0.0%	0	0.0%
mmu-mir-128-2-5p	9	112021148	112021168	-	edit	15	0	0.0%	0	0.0%	0	0.0%
mmu-mir-128-2-5p	9	112021148	112021168	-	edit	16	0	0.0%	4	0.8%	4	1.5%
mmu-mir-128-2-5p	9	112021148	112021168	-	edit	17	0	0.0%	0	0.0%	0	0.0%
mmu-mir-128-2-5p	9	112021148	112021168	-	edit	18	0	0.0%	0	0.0%	0	0.0%
mmu-mir-128-2-5p	9	112021148	112021168	-	edit	19	0	0.0%	0	0.0%	0	0.0%
mmu-mir-128-2-5p	9	112021148	112021168	-	edit	20	0	0.0%	0	0.0%	0	0.0%
mmu-mir-128-2-5p	9	112021148	112021168	-	edit	21	0	0.0%	0	0.0%	0	0.0%
mmu-mir-128-2-5p	9	112021148	112021168	-	edit	22	0	0.0%	0	0.0%	0	0.0%
EDITED							0	0.0%	4	0.8%	4	1.5%
CANONICAL							498	100.0%	469	99.2%	260	98.5%
TOTAL							498		473		264	

miRNA	Chr	Start	Stop	Strand	Type	Position	P7 reads	P7%	P10 reads	P10%	P14 reads	P14%
mmu-mir-130a-5p	2	84581273	84581294	-	edit	1	0	0.0%	0	0.0%	0	0.0%
mmu-mir-130a-5p	2	84581273	84581294	-	edit	2	0	0.0%	0	0.0%	0	0.0%
mmu-mir-130a-5p	2	84581273	84581294	-	edit	3	0	0.0%	0	0.0%	0	0.0%
mmu-mir-130a-5p	2	84581273	84581294	-	edit	4	29	0.6%	5	0.3%	6	0.6%
mmu-mir-130a-5p	2	84581273	84581294	-	edit	5	0	0.0%	0	0.0%	0	0.0%
mmu-mir-130a-5p	2	84581273	84581294	-	edit	6	0	0.0%	0	0.0%	0	0.0%
mmu-mir-130a-5p	2	84581273	84581294	-	edit	7	0	0.0%	0	0.0%	0	0.0%
mmu-mir-130a-5p	2	84581273	84581294	-	edit	8	0	0.0%	0	0.0%	0	0.0%
mmu-mir-130a-5p	2	84581273	84581294	-	edit	9	0	0.0%	0	0.0%	0	0.0%
mmu-mir-130a-5p	2	84581273	84581294	-	edit	10	0	0.0%	0	0.0%	0	0.0%
mmu-mir-130a-5p	2	84581273	84581294	-	edit	11	0	0.0%	0	0.0%	0	0.0%
mmu-mir-130a-5p	2	84581273	84581294	-	edit	12	0	0.0%	0	0.0%	0	0.0%
mmu-mir-130a-5p	2	84581273	84581294	-	edit	13	0	0.0%	0	0.0%	0	0.0%
mmu-mir-130a-5p	2	84581273	84581294	-	edit	14	0	0.0%	0	0.0%	0	0.0%
mmu-mir-130a-5p	2	84581273	84581294	-	edit	15	0	0.0%	13	0.8%	3	0.3%
mmu-mir-130a-5p	2	84581273	84581294	-	edit	16	0	0.0%	0	0.0%	0	0.0%
mmu-mir-130a-5p	2	84581273	84581294	-	edit	17	6	0.1%	0	0.0%	0	0.0%
mmu-mir-130a-5p	2	84581273	84581294	-	edit	18	0	0.0%	0	0.0%	0	0.0%
mmu-mir-130a-5p	2	84581273	84581294	-	edit	19	16	0.3%	4	0.2%	0	0.0%
mmu-mir-130a-5p	2	84581273	84581294	-	edit	20	0	0.0%	0	0.0%	0	0.0%
mmu-mir-130a-5p	2	84581273	84581294	-	edit	21	0	0.0%	0	0.0%	0	0.0%
mmu-mir-130a-5p	2	84581273	84581294	-	edit	22	0	0.0%	0	0.0%	0	0.0%
EDITED							51	1.0%	22	1.2%	9	0.9%
CANONICAL							5126	99.0%	1743	98.8%	935	99.1%
TOTAL							5177		1765		944	

miRNA	Chr	Start	Stop	Strand	Type	Position	P7 reads	P7%	P10 reads	P10%	P14 reads	P14%
mmu-mir-134-5p	12	110972355	110972376	+	edit	1	0	0.0%	0	0.0%	0	0.0%
mmu-mir-134-5p	12	110972355	110972376	+	edit	2	0	0.0%	0	0.0%	0	0.0%
mmu-mir-134-5p	12	110972355	110972376	+	edit	3	0	0.0%	0	0.0%	0	0.0%
mmu-mir-134-5p	12	110972355	110972376	+	edit	4	0	0.0%	0	0.0%	0	0.0%
mmu-mir-134-5p	12	110972355	110972376	+	edit	5	0	0.0%	0	0.0%	0	0.0%
mmu-mir-134-5p	12	110972355	110972376	+	edit	6	0	0.0%	0	0.0%	0	0.0%
mmu-mir-134-5p	12	110972355	110972376	+	edit	7	0	0.0%	0	0.0%	0	0.0%
mmu-mir-134-5p	12	110972355	110972376	+	edit	8	0	0.0%	0	0.0%	0	0.0%
mmu-mir-134-5p	12	110972355	110972376	+	edit	9	0	0.0%	0	0.0%	0	0.0%
mmu-mir-134-5p	12	110972355	110972376	+	edit	10	0	0.0%	0	0.0%	0	0.0%
mmu-mir-134-5p	12	110972355	110972376	+	edit	11	0	0.0%	0	0.0%	0	0.0%
mmu-mir-134-5p	12	110972355	110972376	+	edit	12	0	0.0%	0	0.0%	0	0.0%
mmu-mir-134-5p	12	110972355	110972376	+	edit	13	0	0.0%	0	0.0%	0	0.0%
mmu-mir-134-5p	12	110972355	110972376	+	edit	14	0	0.0%	0	0.0%	0	0.0%
mmu-mir-134-5p	12	110972355	110972376	+	edit	15	6	0.7%	0	0.0%	0	0.0%
mmu-mir-134-5p	12	110972355	110972376	+	edit	16	0	0.0%	0	0.0%	0	0.0%
mmu-mir-134-5p	12	110972355	110972376	+	edit	17	0	0.0%	0	0.0%	0	0.0%
mmu-mir-134-5p	12	110972355	110972376	+	edit	18	0	0.0%	0	0.0%	0	0.0%
mmu-mir-134-5p	12	110972355	110972376	+	edit	19	0	0.0%	0	0.0%	0	0.0%
mmu-mir-134-5p	12	110972355	110972376	+	edit	20	0	0.0%	0	0.0%	0	0.0%
mmu-mir-134-5p	12	110972355	110972376	+	edit	21	0	0.0%	0	0.0%	0	0.0%
mmu-mir-134-5p	12	110972355	110972376	+	edit	22	0	0.0%	0	0.0%	0	0.0%
EDITED							6	0.7%	0	0.0%	0	0.0%
CANONICAL							759	99.3%	210	100.0%	67	100.0%
TOTAL							765		210		67	

miRNA	Chr	Start	Stop	Strand	Type	Position	P7 reads	P7%	P10 reads	P10%	P14 reads	P14%
mmu-mir-140-3p	8	110075188	110075208	+	3' Edit	3'	90	0.1%	0	0.0%	59	0.3%
mmu-mir-140-3p	8	110075188	110075208	+	3' Indel	3'	6	0.0%	0	0.0%	7	0.0%
mmu-mir-140-3p	8	110075188	110075208	+	Indel	21	4	0.0%	0	0.0%	5	0.0%
mmu-mir-140-3p	8	110075188	110075208	+	edit	1	0	0.0%	0	0.0%	0	0.0%
mmu-mir-140-3p	8	110075188	110075208	+	edit	2	0	0.0%	0	0.0%	0	0.0%
mmu-mir-140-3p	8	110075188	110075208	+	edit	3	0	0.0%	0	0.0%	0	0.0%
mmu-mir-140-3p	8	110075188	110075208	+	edit	4	31	0.0%	10	0.0%	15	0.1%
mmu-mir-140-3p	8	110075188	110075208	+	edit	5	3	0.0%	6	0.0%	0	0.0%
mmu-mir-140-3p	8	110075188	110075208	+	edit	6	12	0.0%	15	0.0%	8	0.0%
mmu-mir-140-3p	8	110075188	110075208	+	edit	7	16	0.0%	15	0.0%	11	0.0%
mmu-mir-140-3p	8	110075188	110075208	+	edit	8	40	0.0%	10	0.0%	0	0.0%
mmu-mir-140-3p	8	110075188	110075208	+	edit	9	19	0.0%	3	0.0%	5	0.0%
mmu-mir-140-3p	8	110075188	110075208	+	edit	10	0	0.0%	0	0.0%	0	0.0%
mmu-mir-140-3p	8	110075188	110075208	+	edit	11	6	0.0%	0	0.0%	0	0.0%
mmu-mir-140-3p	8	110075188	110075208	+	edit	12	0	0.0%	0	0.0%	0	0.0%
mmu-mir-140-3p	8	110075188	110075208	+	edit	13	0	0.0%	0	0.0%	0	0.0%
mmu-mir-140-3p	8	110075188	110075208	+	edit	14	0	0.0%	0	0.0%	0	0.0%
mmu-mir-140-3p	8	110075188	110075208	+	edit	15	22	0.0%	124	0.3%	7	0.0%
mmu-mir-140-3p	8	110075188	110075208	+	edit	16	151	0.2%	89	0.2%	78	0.3%
mmu-mir-140-3p	8	110075188	110075208	+	edit	17	74	0.1%	46	0.1%	21	0.1%
mmu-mir-140-3p	8	110075188	110075208	+	edit	18	0	0.0%	0	0.0%	0	0.0%
mmu-mir-140-3p	8	110075188	110075208	+	edit	19	428	0.5%	201	0.4%	243	1.0%
mmu-mir-140-3p	8	110075188	110075208	+	edit	20	67	0.1%	40	0.1%	13	0.1%
mmu-mir-140-3p	8	110075188	110075208	+	edit	21	22	0.0%	3	0.0%	5	0.0%
mmu-mir-140-3p	8	110075188	110075208	+	edit	22	0	0.0%	0	0.0%	0	0.0%
EDITED							990	1.2%	562	1.2%	477	2.0%
CANONICAL							83969	98.8%	45691	98.8%	23075	98.0%
TOTAL							84959		46253		23552	

miRNA	Chr	Start	Stop	Strand	Type	Position	P7 reads	P7%	P10 reads	P10%	P14 reads	P14%
mmu-mir-143-5p	18	61808853	61808873	-	5' Edit	5'	4	0.0%	0	0.0%	4	0.1%
mmu-mir-143-5p	18	61808853	61808873	-	5' Indel	5'	8	0.1%	0	0.0%	0	0.0%
mmu-mir-143-5p	18	61808853	61808873	-	edit	1	0	0.0%	0	0.0%	0	0.0%
mmu-mir-143-5p	18	61808853	61808873	-	edit	2	0	0.0%	0	0.0%	0	0.0%
mmu-mir-143-5p	18	61808853	61808873	-	edit	3	0	0.0%	0	0.0%	0	0.0%
mmu-mir-143-5p	18	61808853	61808873	-	edit	4	0	0.0%	0	0.0%	0	0.0%
mmu-mir-143-5p	18	61808853	61808873	-	edit	5	0	0.0%	0	0.0%	0	0.0%
mmu-mir-143-5p	18	61808853	61808873	-	edit	6	0	0.0%	0	0.0%	0	0.0%
mmu-mir-143-5p	18	61808853	61808873	-	edit	7	0	0.0%	0	0.0%	0	0.0%
mmu-mir-143-5p	18	61808853	61808873	-	edit	8	0	0.0%	0	0.0%	0	0.0%
mmu-mir-143-5p	18	61808853	61808873	-	edit	9	0	0.0%	0	0.0%	0	0.0%
mmu-mir-143-5p	18	61808853	61808873	-	edit	10	0	0.0%	0	0.0%	0	0.0%
mmu-mir-143-5p	18	61808853	61808873	-	edit	11	0	0.0%	0	0.0%	0	0.0%
mmu-mir-143-5p	18	61808853	61808873	-	edit	12	0	0.0%	0	0.0%	0	0.0%
mmu-mir-143-5p	18	61808853	61808873	-	edit	13	0	0.0%	0	0.0%	0	0.0%
mmu-mir-143-5p	18	61808853	61808873	-	edit	14	0	0.0%	0	0.0%	0	0.0%
mmu-mir-143-5p	18	61808853	61808873	-	edit	15	6	0.1%	0	0.0%	7	0.1%
mmu-mir-143-5p	18	61808853	61808873	-	edit	16	13	0.1%	11	0.1%	6	0.1%
mmu-mir-143-5p	18	61808853	61808873	-	edit	17	0	0.0%	0	0.0%	0	0.0%
mmu-mir-143-5p	18	61808853	61808873	-	edit	18	0	0.0%	0	0.0%	0	0.0%
mmu-mir-143-5p	18	61808853	61808873	-	edit	19	14	0.1%	10	0.1%	15	0.2%
mmu-mir-143-5p	18	61808853	61808873	-	edit	20	0	0.0%	0	0.0%	4	0.1%
mmu-mir-143-5p	18	61808853	61808873	-	edit	21	0	0.0%	0	0.0%	0	0.0%
mmu-mir-143-5p	18	61808853	61808873	-	edit	22	0	0.0%	0	0.0%	0	0.0%
EDITED							44	0.4%	20	0.2%	35	0.6%
CANONICAL							10776	99.6%	9027	99.8%	6303	99.4%
TOTAL							10820		9047		6338	

miRNA	Chr	Start	Stop	Strand	Type	Position	P7 reads	P7%	P10 reads	P10%	P14 reads	P14%
mmu-mir-145-5p	18	61807520	61807542	-	edit	1	0	0.0%	0	0.0%	0	0.0%
mmu-mir-145-5p	18	61807520	61807542	-	edit	2	0	0.0%	0	0.0%	0	0.0%
mmu-mir-145-5p	18	61807520	61807542	-	edit	3	0	0.0%	0	0.0%	0	0.0%
mmu-mir-145-5p	18	61807520	61807542	-	edit	4	0	0.0%	0	0.0%	0	0.0%
mmu-mir-145-5p	18	61807520	61807542	-	edit	5	0	0.0%	0	0.0%	0	0.0%
mmu-mir-145-5p	18	61807520	61807542	-	edit	6	0	0.0%	0	0.0%	0	0.0%
mmu-mir-145-5p	18	61807520	61807542	-	edit	7	0	0.0%	0	0.0%	0	0.0%
mmu-mir-145-5p	18	61807520	61807542	-	edit	8	0	0.0%	0	0.0%	0	0.0%
mmu-mir-145-5p	18	61807520	61807542	-	edit	9	0	0.0%	0	0.0%	0	0.0%
mmu-mir-145-5p	18	61807520	61807542	-	edit	10	0	0.0%	0	0.0%	0	0.0%
mmu-mir-145-5p	18	61807520	61807542	-	edit	11	0	0.0%	0	0.0%	0	0.0%
mmu-mir-145-5p	18	61807520	61807542	-	edit	12	0	0.0%	0	0.0%	0	0.0%
mmu-mir-145-5p	18	61807520	61807542	-	edit	13	0	0.0%	0	0.0%	0	0.0%
mmu-mir-145-5p	18	61807520	61807542	-	edit	14	0	0.0%	0	0.0%	0	0.0%
mmu-mir-145-5p	18	61807520	61807542	-	edit	15	0	0.0%	0	0.0%	0	0.0%
mmu-mir-145-5p	18	61807520	61807542	-	edit	16	0	0.0%	0	0.0%	0	0.0%
mmu-mir-145-5p	18	61807520	61807542	-	edit	17	0	0.0%	8	1.1%	0	0.0%
mmu-mir-145-5p	18	61807520	61807542	-	edit	18	0	0.0%	0	0.0%	0	0.0%
mmu-mir-145-5p	18	61807520	61807542	-	edit	19	0	0.0%	0	0.0%	0	0.0%
mmu-mir-145-5p	18	61807520	61807542	-	edit	20	0	0.0%	0	0.0%	0	0.0%
mmu-mir-145-5p	18	61807520	61807542	-	edit	21	0	0.0%	3	0.4%	0	0.0%
mmu-mir-145-5p	18	61807520	61807542	-	edit	22	0	0.0%	0	0.0%	0	0.0%
EDITED							0	0.0%	11	1.5%	0	0.0%
CANONICAL							949	100.0%	682	98.5%	769	100.0%
TOTAL							949		693		769	

miRNA	Chr	Start	Stop	Strand	Type	Position	P7 reads	P7%	P10 reads	P10%	P14 reads	P14%
mmu-mir-146b-5p	19	46417280	46417301	+	edit	1	0	0.0%	0	0.0%	0	0.0%
mmu-mir-146b-5p	19	46417280	46417301	+	edit	2	0	0.0%	0	0.0%	0	0.0%
mmu-mir-146b-5p	19	46417280	46417301	+	edit	3	0	0.0%	0	0.0%	0	0.0%
mmu-mir-146b-5p	19	46417280	46417301	+	edit	4	0	0.0%	0	0.0%	0	0.0%
mmu-mir-146b-5p	19	46417280	46417301	+	edit	5	0	0.0%	0	0.0%	0	0.0%
mmu-mir-146b-5p	19	46417280	46417301	+	edit	6	0	0.0%	0	0.0%	0	0.0%
mmu-mir-146b-5p	19	46417280	46417301	+	edit	7	0	0.0%	0	0.0%	0	0.0%
mmu-mir-146b-5p	19	46417280	46417301	+	edit	8	0	0.0%	0	0.0%	0	0.0%
mmu-mir-146b-5p	19	46417280	46417301	+	edit	9	0	0.0%	0	0.0%	0	0.0%
mmu-mir-146b-5p	19	46417280	46417301	+	edit	10	0	0.0%	0	0.0%	0	0.0%
mmu-mir-146b-5p	19	46417280	46417301	+	edit	11	0	0.0%	0	0.0%	0	0.0%
mmu-mir-146b-5p	19	46417280	46417301	+	edit	12	0	0.0%	0	0.0%	0	0.0%
mmu-mir-146b-5p	19	46417280	46417301	+	edit	13	0	0.0%	0	0.0%	0	0.0%
mmu-mir-146b-5p	19	46417280	46417301	+	edit	14	0	0.0%	0	0.0%	0	0.0%
mmu-mir-146b-5p	19	46417280	46417301	+	edit	15	0	0.0%	0	0.0%	0	0.0%
mmu-mir-146b-5p	19	46417280	46417301	+	edit	16	0	0.0%	0	0.0%	0	0.0%
mmu-mir-146b-5p	19	46417280	46417301	+	edit	17	0	0.0%	0	0.0%	0	0.0%
mmu-mir-146b-5p	19	46417280	46417301	+	edit	18	0	0.0%	0	0.0%	0	0.0%
mmu-mir-146b-5p	19	46417280	46417301	+	edit	19	0	0.0%	0	0.0%	0	0.0%
mmu-mir-146b-5p	19	46417280	46417301	+	edit	20	0	0.0%	0	0.0%	0	0.0%
mmu-mir-146b-5p	19	46417280	46417301	+	edit	21	3	1.1%	0	0.0%	0	0.0%
mmu-mir-146b-5p	19	46417280	46417301	+	edit	22	0	0.0%	0	0.0%	0	0.0%
EDITED							3	1.1%	0	0.0%	0	0.0%
CANONICAL							288	98.9%	228	100.0%	297	100.0%
TOTAL							291		228		297	

miRNA	Chr	Start	Stop	Strand	Type	Position	P7 reads	P7%	P10 reads	P10%	P14 reads	P14%
mmu-mir-148a-3p	6	51219828	51219849	-	edit	1	0	0.0%	0	0.0%	0	0.0%
mmu-mir-148a-3p	6	51219828	51219849	-	edit	2	0	0.0%	0	0.0%	0	0.0%
mmu-mir-148a-3p	6	51219828	51219849	-	edit	3	0	0.0%	0	0.0%	0	0.0%
mmu-mir-148a-3p	6	51219828	51219849	-	edit	4	0	0.0%	0	0.0%	0	0.0%
mmu-mir-148a-3p	6	51219828	51219849	-	edit	5	0	0.0%	0	0.0%	0	0.0%
mmu-mir-148a-3p	6	51219828	51219849	-	edit	6	0	0.0%	0	0.0%	0	0.0%
mmu-mir-148a-3p	6	51219828	51219849	-	edit	7	0	0.0%	0	0.0%	0	0.0%
mmu-mir-148a-3p	6	51219828	51219849	-	edit	8	5	0.3%	0	0.0%	0	0.0%
mmu-mir-148a-3p	6	51219828	51219849	-	edit	9	0	0.0%	0	0.0%	0	0.0%
mmu-mir-148a-3p	6	51219828	51219849	-	edit	10	0	0.0%	0	0.0%	0	0.0%
mmu-mir-148a-3p	6	51219828	51219849	-	edit	11	0	0.0%	0	0.0%	0	0.0%
mmu-mir-148a-3p	6	51219828	51219849	-	edit	12	0	0.0%	0	0.0%	0	0.0%
mmu-mir-148a-3p	6	51219828	51219849	-	edit	13	0	0.0%	0	0.0%	0	0.0%
mmu-mir-148a-3p	6	51219828	51219849	-	edit	14	0	0.0%	0	0.0%	0	0.0%
mmu-mir-148a-3p	6	51219828	51219849	-	edit	15	0	0.0%	19	1.5%	0	0.0%
mmu-mir-148a-3p	6	51219828	51219849	-	edit	16	0	0.0%	0	0.0%	0	0.0%
mmu-mir-148a-3p	6	51219828	51219849	-	edit	17	0	0.0%	0	0.0%	0	0.0%
mmu-mir-148a-3p	6	51219828	51219849	-	edit	18	0	0.0%	0	0.0%	0	0.0%
mmu-mir-148a-3p	6	51219828	51219849	-	edit	19	0	0.0%	3	0.2%	0	0.0%
mmu-mir-148a-3p	6	51219828	51219849	-	edit	20	0	0.0%	0	0.0%	0	0.0%
mmu-mir-148a-3p	6	51219828	51219849	-	edit	21	0	0.0%	0	0.0%	0	0.0%
mmu-mir-148a-3p	6	51219828	51219849	-	edit	22	0	0.0%	0	0.0%	0	0.0%
EDITED							5	0.3%	22	1.7%	0	0.0%
CANONICAL							1776	99.7%	1232	98.3%	732	100.0%
TOTAL							1781		1254		732	

miRNA	Chr	Start	Stop	Strand	Type	Position	P7 reads	P7%	P10 reads	P10%	P14 reads	P14%
mmu-mir-151-3p	15	73085250	73085270	-	edit	1	0	0.0%	0	0.0%	0	0.0%
mmu-mir-151-3p	15	73085250	73085270	-	edit	2	0	0.0%	0	0.0%	0	0.0%
mmu-mir-151-3p	15	73085250	73085270	-	edit	3	0	0.0%	0	0.0%	0	0.0%
mmu-mir-151-3p	15	73085250	73085270	-	edit	4	0	0.0%	0	0.0%	0	0.0%
mmu-mir-151-3p	15	73085250	73085270	-	edit	5	0	0.0%	0	0.0%	0	0.0%
mmu-mir-151-3p	15	73085250	73085270	-	edit	6	0	0.0%	0	0.0%	0	0.0%
mmu-mir-151-3p	15	73085250	73085270	-	edit	7	0	0.0%	0	0.0%	0	0.0%
mmu-mir-151-3p	15	73085250	73085270	-	edit	8	0	0.0%	0	0.0%	0	0.0%
mmu-mir-151-3p	15	73085250	73085270	-	edit	9	0	0.0%	0	0.0%	0	0.0%
mmu-mir-151-3p	15	73085250	73085270	-	edit	10	0	0.0%	0	0.0%	0	0.0%
mmu-mir-151-3p	15	73085250	73085270	-	edit	11	0	0.0%	0	0.0%	0	0.0%
mmu-mir-151-3p	15	73085250	73085270	-	edit	12	0	0.0%	0	0.0%	0	0.0%
mmu-mir-151-3p	15	73085250	73085270	-	edit	13	0	0.0%	0	0.0%	0	0.0%
mmu-mir-151-3p	15	73085250	73085270	-	edit	14	0	0.0%	0	0.0%	0	0.0%
mmu-mir-151-3p	15	73085250	73085270	-	edit	15	5	0.2%	5	0.5%	0	0.0%
mmu-mir-151-3p	15	73085250	73085270	-	edit	16	0	0.0%	0	0.0%	0	0.0%
mmu-mir-151-3p	15	73085250	73085270	-	edit	17	4	0.2%	0	0.0%	0	0.0%
mmu-mir-151-3p	15	73085250	73085270	-	edit	18	0	0.0%	0	0.0%	0	0.0%
mmu-mir-151-3p	15	73085250	73085270	-	edit	19	10	0.5%	11	1.1%	8	1.0%
mmu-mir-151-3p	15	73085250	73085270	-	edit	20	0	0.0%	0	0.0%	0	0.0%
mmu-mir-151-3p	15	73085250	73085270	-	edit	21	0	0.0%	0	0.0%	0	0.0%
mmu-mir-151-3p	15	73085250	73085270	-	edit	22	0	0.0%	0	0.0%	0	0.0%
EDITED							18	0.9%	16	1.6%	8	1.0%
CANONICAL							2104	99.1%	1005	98.4%	832	99.0%
TOTAL							2122		1021		840	

miRNA	Chr	Start	Stop	Strand	Type	Position	P7 reads	P7%	P10 reads	P10%	P14 reads	P14%
mmu-mir-152-5p	11	96711753	96711773	+	edit	1	0	0.0%	0	0.0%	0	0.0%
mmu-mir-152-5p	11	96711753	96711773	+	edit	2	0	0.0%	0	0.0%	0	0.0%
mmu-mir-152-5p	11	96711753	96711773	+	edit	3	0	0.0%	0	0.0%	0	0.0%
mmu-mir-152-5p	11	96711753	96711773	+	edit	4	0	0.0%	0	0.0%	0	0.0%
mmu-mir-152-5p	11	96711753	96711773	+	edit	5	0	0.0%	0	0.0%	0	0.0%
mmu-mir-152-5p	11	96711753	96711773	+	edit	6	10	0.0%	3	0.0%	0	0.0%
mmu-mir-152-5p	11	96711753	96711773	+	edit	7	0	0.0%	0	0.0%	0	0.0%
mmu-mir-152-5p	11	96711753	96711773	+	edit	8	0	0.0%	0	0.0%	0	0.0%
mmu-mir-152-5p	11	96711753	96711773	+	edit	9	0	0.0%	0	0.0%	0	0.0%
mmu-mir-152-5p	11	96711753	96711773	+	edit	10	0	0.0%	0	0.0%	0	0.0%
mmu-mir-152-5p	11	96711753	96711773	+	edit	11	0	0.0%	0	0.0%	0	0.0%
mmu-mir-152-5p	11	96711753	96711773	+	edit	12	0	0.0%	0	0.0%	0	0.0%
mmu-mir-152-5p	11	96711753	96711773	+	edit	13	0	0.0%	0	0.0%	0	0.0%
mmu-mir-152-5p	11	96711753	96711773	+	edit	14	0	0.0%	0	0.0%	0	0.0%
mmu-mir-152-5p	11	96711753	96711773	+	edit	15	3	0.0%	8	0.1%	0	0.0%
mmu-mir-152-5p	11	96711753	96711773	+	edit	16	0	0.0%	0	0.0%	0	0.0%
mmu-mir-152-5p	11	96711753	96711773	+	edit	17	0	0.0%	0	0.0%	0	0.0%
mmu-mir-152-5p	11	96711753	96711773	+	edit	18	0	0.0%	0	0.0%	0	0.0%
mmu-mir-152-5p	11	96711753	96711773	+	edit	19	7	0.0%	0	0.0%	0	0.0%
mmu-mir-152-5p	11	96711753	96711773	+	edit	20	0	0.0%	0	0.0%	0	0.0%
mmu-mir-152-5p	11	96711753	96711773	+	edit	21	0	0.0%	0	0.0%	0	0.0%
mmu-mir-152-5p	11	96711753	96711773	+	edit	22	0	0.0%	0	0.0%	0	0.0%
EDITED							20	0.1%	11	0.1%	0	0.0%
CANONICAL							25462	99.9%	14706	99.9%	6897	100.0%
TOTAL							25482		14717		6897	

miRNA	Chr	Start	Stop	Strand	Type	Position	P7 reads	P7%	P10 reads	P10%	P14 reads	P14%
mmu-mir-181a-1-5p	1	139863045	139863067	+	3' Edit	3'	11	0.1%	0	0.0%	3	0.1%
mmu-mir-181a-1-5p	1	139863045	139863067	+	Indel	20	5	0.0%	0	0.0%	0	0.0%
mmu-mir-181a-1-5p	1	139863045	139863067	+	Indel	21	37	0.4%	13	0.3%	13	0.6%
mmu-mir-181a-1-5p	1	139863045	139863067	+	Indel	22	26	0.3%	0	0.0%	12	0.5%
mmu-mir-181a-1-5p	1	139863045	139863067	+	Indel	23	7	0.1%	0	0.0%	0	0.0%
mmu-mir-181a-1-5p	1	139863045	139863067	+	edit	1	0	0.0%	0	0.0%	0	0.0%
mmu-mir-181a-1-5p	1	139863045	139863067	+	edit	2	0	0.0%	0	0.0%	0	0.0%
mmu-mir-181a-1-5p	1	139863045	139863067	+	edit	3	0	0.0%	0	0.0%	0	0.0%
mmu-mir-181a-1-5p	1	139863045	139863067	+	edit	4	0	0.0%	0	0.0%	0	0.0%
mmu-mir-181a-1-5p	1	139863045	139863067	+	edit	5	0	0.0%	0	0.0%	0	0.0%
mmu-mir-181a-1-5p	1	139863045	139863067	+	edit	6	0	0.0%	0	0.0%	0	0.0%
mmu-mir-181a-1-5p	1	139863045	139863067	+	edit	7	0	0.0%	0	0.0%	0	0.0%
mmu-mir-181a-1-5p	1	139863045	139863067	+	edit	8	6	0.1%	0	0.0%	0	0.0%
mmu-mir-181a-1-5p	1	139863045	139863067	+	edit	9	0	0.0%	0	0.0%	0	0.0%
mmu-mir-181a-1-5p	1	139863045	139863067	+	edit	10	3	0.0%	0	0.0%	3	0.1%
mmu-mir-181a-1-5p	1	139863045	139863067	+	edit	11	0	0.0%	0	0.0%	0	0.0%
mmu-mir-181a-1-5p	1	139863045	139863067	+	edit	12	6	0.1%	0	0.0%	0	0.0%
mmu-mir-181a-1-5p	1	139863045	139863067	+	edit	13	6	0.1%	0	0.0%	0	0.0%
mmu-mir-181a-1-5p	1	139863045	139863067	+	edit	14	0	0.0%	0	0.0%	0	0.0%
mmu-mir-181a-1-5p	1	139863045	139863067	+	edit	15	68	0.7%	12	0.3%	9	0.4%
mmu-mir-181a-1-5p	1	139863045	139863067	+	edit	16	0	0.0%	0	0.0%	0	0.0%
mmu-mir-181a-1-5p	1	139863045	139863067	+	edit	17	10	0.1%	0	0.0%	0	0.0%
mmu-mir-181a-1-5p	1	139863045	139863067	+	edit	18	0	0.0%	0	0.0%	0	0.0%
mmu-mir-181a-1-5p	1	139863045	139863067	+	edit	19	**651**	**6.9%**	**211**	**5.3%**	**207**	**9.6%**
mmu-mir-181a-1-5p	1	139863045	139863067	+	edit	20	0	0.0%	0	0.0%	0	0.0%
mmu-mir-181a-1-5p	1	139863045	139863067	+	edit	21	348	3.7%	369	9.3%	93	4.3%
mmu-mir-181a-1-5p	1	139863045	139863067	+	edit	22	11	0.1%	0	0.0%	0	0.0%
mmu-mir-181a-1-5p	1	139863045	139863067	+	edit	23	43	0.4%	8	0.2%	16	0.7%
EDITED							1234	13.0%	614	15.4%	354	16.4%
CANONICAL							8252	87.0%	3368	84.6%	1808	83.6%
TOTAL							9486		3982		2162	

miRNA	Chr	Start	Stop	Strand	Type	Position	P7 reads	P7%	P10 reads	P10%	P14 reads	P14%
mmu-mir-181a-2-5p	2	38708261	38708283	+	3' Edit	3'	4	0.0%	0	0.0%	0	0.0%
mmu-mir-181a-2-5p	2	38708261	38708283	+	Indel	20	4	0.0%	0	0.0%	0	0.0%
mmu-mir-181a-2-5p	2	38708261	38708283	+	Indel	21	39	0.4%	11	0.3%	11	0.5%
mmu-mir-181a-2-5p	2	38708261	38708283	+	Indel	22	12	0.1%	4	0.1%	6	0.3%
mmu-mir-181a-2-5p	2	38708261	38708283	+	Indel	23	4	0.0%	0	0.0%	0	0.0%
mmu-mir-181a-2-5p	2	38708261	38708283	+	edit	1	0	0.0%	0	0.0%	0	0.0%
mmu-mir-181a-2-5p	2	38708261	38708283	+	edit	2	0	0.0%	0	0.0%	0	0.0%
mmu-mir-181a-2-5p	2	38708261	38708283	+	edit	3	0	0.0%	0	0.0%	0	0.0%
mmu-mir-181a-2-5p	2	38708261	38708283	+	edit	4	0	0.0%	0	0.0%	0	0.0%
mmu-mir-181a-2-5p	2	38708261	38708283	+	edit	5	0	0.0%	0	0.0%	0	0.0%
mmu-mir-181a-2-5p	2	38708261	38708283	+	edit	6	0	0.0%	0	0.0%	0	0.0%
mmu-mir-181a-2-5p	2	38708261	38708283	+	edit	7	0	0.0%	0	0.0%	0	0.0%
mmu-mir-181a-2-5p	2	38708261	38708283	+	edit	8	6	0.1%	0	0.0%	0	0.0%
mmu-mir-181a-2-5p	2	38708261	38708283	+	edit	9	0	0.0%	0	0.0%	0	0.0%
mmu-mir-181a-2-5p	2	38708261	38708283	+	edit	10	3	0.0%	0	0.0%	3	0.1%
mmu-mir-181a-2-5p	2	38708261	38708283	+	edit	11	0	0.0%	0	0.0%	0	0.0%
mmu-mir-181a-2-5p	2	38708261	38708283	+	edit	12	6	0.1%	0	0.0%	0	0.0%
mmu-mir-181a-2-5p	2	38708261	38708283	+	edit	13	6	0.1%	0	0.0%	0	0.0%
mmu-mir-181a-2-5p	2	38708261	38708283	+	edit	14	0	0.0%	0	0.0%	0	0.0%
mmu-mir-181a-2-5p	2	38708261	38708283	+	edit	15	67	0.7%	12	0.3%	9	0.4%
mmu-mir-181a-2-5p	2	38708261	38708283	+	edit	16	0	0.0%	0	0.0%	0	0.0%
mmu-mir-181a-2-5p	2	38708261	38708283	+	edit	17	9	0.1%	0	0.0%	0	0.0%
mmu-mir-181a-2-5p	2	38708261	38708283	+	edit	18	0	0.0%	0	0.0%	0	0.0%
mmu-mir-181a-2-5p	2	38708261	38708283	+	edit	19	**649**	**6.9%**	**213**	**5.3%**	**210**	**9.8%**
mmu-mir-181a-2-5p	2	38708261	38708283	+	edit	20	0	0.0%	0	0.0%	0	0.0%
mmu-mir-181a-2-5p	2	38708261	38708283	+	edit	21	346	3.7%	372	9.3%	94	4.4%
mmu-mir-181a-2-5p	2	38708261	38708283	+	edit	22	11	0.1%	0	0.0%	0	0.0%
mmu-mir-181a-2-5p	2	38708261	38708283	+	edit	23	30	0.3%	11	0.3%	13	0.6%
EDITED							1192	12.7%	623	15.6%	344	16.1%
CANONICAL							8208	87.3%	3381	84.4%	1799	83.9%
TOTAL							9400		4004		2143	

miRNA	Chr	Start	Stop	Strand	Type	Position	P7 reads	P7%	P10 reads	P10%	P14 reads	P14%
mmu-mir-181b-1-5p	1	139863227	139863249	+	3' Edit	3'	5	0.1%	0	0.0%	0	0.0%
mmu-mir-181b-1-5p	1	139863227	139863249	+	edit	1	0	0.0%	0	0.0%	0	0.0%
mmu-mir-181b-1-5p	1	139863227	139863249	+	edit	2	0	0.0%	0	0.0%	0	0.0%
mmu-mir-181b-1-5p	1	139863227	139863249	+	edit	3	0	0.0%	0	0.0%	0	0.0%
mmu-mir-181b-1-5p	1	139863227	139863249	+	edit	4	0	0.0%	0	0.0%	0	0.0%
mmu-mir-181b-1-5p	1	139863227	139863249	+	edit	5	0	0.0%	0	0.0%	0	0.0%
mmu-mir-181b-1-5p	1	139863227	139863249	+	edit	6	0	0.0%	0	0.0%	0	0.0%
mmu-mir-181b-1-5p	1	139863227	139863249	+	edit	7	0	0.0%	0	0.0%	0	0.0%
mmu-mir-181b-1-5p	1	139863227	139863249	+	edit	8	3	0.1%	0	0.0%	0	0.0%
mmu-mir-181b-1-5p	1	139863227	139863249	+	edit	9	0	0.0%	0	0.0%	0	0.0%
mmu-mir-181b-1-5p	1	139863227	139863249	+	edit	10	0	0.0%	0	0.0%	0	0.0%
mmu-mir-181b-1-5p	1	139863227	139863249	+	edit	11	0	0.0%	0	0.0%	0	0.0%
mmu-mir-181b-1-5p	1	139863227	139863249	+	edit	12	14	0.3%	0	0.0%	0	0.0%
mmu-mir-181b-1-5p	1	139863227	139863249	+	edit	13	0	0.0%	0	0.0%	0	0.0%
mmu-mir-181b-1-5p	1	139863227	139863249	+	edit	14	0	0.0%	0	0.0%	0	0.0%
mmu-mir-181b-1-5p	1	139863227	139863249	+	edit	15	19	0.4%	4	0.2%	0	0.0%
mmu-mir-181b-1-5p	1	139863227	139863249	+	edit	16	0	0.0%	0	0.0%	0	0.0%
mmu-mir-181b-1-5p	1	139863227	139863249	+	edit	17	4	0.1%	0	0.0%	0	0.0%
mmu-mir-181b-1-5p	1	139863227	139863249	+	edit	18	0	0.0%	0	0.0%	0	0.0%
mmu-mir-181b-1-5p	1	139863227	139863249	+	edit	19	**434**	**7.9%**	**125**	**6.7%**	**98**	**9.9%**
mmu-mir-181b-1-5p	1	139863227	139863249	+	edit	20	0	0.0%	0	0.0%	0	0.0%
mmu-mir-181b-1-5p	1	139863227	139863249	+	edit	21	0	0.0%	5	0.2%	0	0.0%
mmu-mir-181b-1-5p	1	139863227	139863249	+	edit	22	4	0.1%	0	0.0%	0	0.0%
mmu-mir-181b-1-5p	1	139863227	139863249	+	edit	23	4	0.1%	0	0.0%	5	0.5%
EDITED							486	8.9%	133	7.2%	103	10.4%
CANONICAL							5002	91.1%	1713	92.8%	883	89.6%
TOTAL							5488		1846		986	

miRNA	Chr	Start	Stop	Strand	Type	Position	P7 reads	P7%	P10 reads	P10%	P14 reads	P14%
mmu-mir-181b-2-5p	2	38709365	38709387	+	3' Edit	3'	16	0.1%	0	0.0%	7	0.3%
mmu-mir-181b-2-5p	2	38709365	38709387	+	3' Indel	3'	60	0.5%	0	0.0%	12	0.6%
mmu-mir-181b-2-5p	2	38709365	38709387	+	Indel	16	29	0.2%	3	0.1%	9	0.4%
mmu-mir-181b-2-5p	2	38709365	38709387	+	Indel	17	0	0.0%	0	0.0%	0	0.0%
mmu-mir-181b-2-5p	2	38709365	38709387	+	Indel	18	4	0.0%	0	0.0%	0	0.0%
mmu-mir-181b-2-5p	2	38709365	38709387	+	Indel	19	218	1.9%	71	1.8%	74	3.5%
mmu-mir-181b-2-5p	2	38709365	38709387	+	Indel	20	4	0.0%	0	0.0%	0	0.0%
mmu-mir-181b-2-5p	2	38709365	38709387	+	Indel	21	6	0.1%	3	0.1%	0	0.0%
mmu-mir-181b-2-5p	2	38709365	38709387	+	Indel	22	87	0.7%	19	0.5%	11	0.5%
mmu-mir-181b-2-5p	2	38709365	38709387	+	Indel	23	25	0.2%	6	0.2%	5	0.2%
mmu-mir-181b-2-5p	2	38709365	38709387	+	edit	1	0	0.0%	0	0.0%	0	0.0%
mmu-mir-181b-2-5p	2	38709365	38709387	+	edit	2	0	0.0%	0	0.0%	0	0.0%
mmu-mir-181b-2-5p	2	38709365	38709387	+	edit	3	0	0.0%	0	0.0%	0	0.0%
mmu-mir-181b-2-5p	2	38709365	38709387	+	edit	4	0	0.0%	5	0.1%	0	0.0%
mmu-mir-181b-2-5p	2	38709365	38709387	+	edit	5	0	0.0%	0	0.0%	0	0.0%
mmu-mir-181b-2-5p	2	38709365	38709387	+	edit	6	4	0.0%	0	0.0%	0	0.0%
mmu-mir-181b-2-5p	2	38709365	38709387	+	edit	7	0	0.0%	0	0.0%	0	0.0%
mmu-mir-181b-2-5p	2	38709365	38709387	+	edit	8	6	0.1%	0	0.0%	0	0.0%
mmu-mir-181b-2-5p	2	38709365	38709387	+	edit	9	0	0.0%	0	0.0%	0	0.0%
mmu-mir-181b-2-5p	2	38709365	38709387	+	edit	10	7	0.1%	0	0.0%	4	0.2%
mmu-mir-181b-2-5p	2	38709365	38709387	+	edit	11	3	0.0%	0	0.0%	0	0.0%
mmu-mir-181b-2-5p	2	38709365	38709387	+	edit	12	110	0.9%	26	0.7%	21	1.0%
mmu-mir-181b-2-5p	2	38709365	38709387	+	edit	13	0	0.0%	0	0.0%	0	0.0%
mmu-mir-181b-2-5p	2	38709365	38709387	+	edit	14	13	0.1%	0	0.0%	0	0.0%
mmu-mir-181b-2-5p	2	38709365	38709387	+	edit	15	49	0.4%	11	0.3%	5	0.2%
mmu-mir-181b-2-5p	2	38709365	38709387	+	edit	16	0	0.0%	0	0.0%	0	0.0%
mmu-mir-181b-2-5p	2	38709365	38709387	+	edit	17	11	0.1%	4	0.1%	0	0.0%
mmu-mir-181b-2-5p	2	38709365	38709387	+	edit	18	0	0.0%	0	0.0%	0	0.0%
mmu-mir-181b-2-5p	2	38709365	38709387	+	edit	19	**860**	**7.3%**	**275**	**6.9%**	**201**	**9.5%**
mmu-mir-181b-2-5p	2	38709365	38709387	+	edit	20	0	0.0%	0	0.0%	0	0.0%
mmu-mir-181b-2-5p	2	38709365	38709387	+	edit	21	7	0.1%	12	0.3%	0	0.0%
mmu-mir-181b-2-5p	2	38709365	38709387	+	edit	22	24	0.2%	7	0.2%	3	0.1%
mmu-mir-181b-2-5p	2	38709365	38709387	+	edit	23	172	1.5%	68	1.7%	57	2.7%
EDITED							1712	14.6%	508	12.8%	407	19.2%
CANONICAL							10037	85.4%	3469	87.2%	1711	80.8%
TOTAL							11749		3977		2118	

miRNA	Chr	Start	Stop	Strand	Type	Position	P7 reads	P7%	P10 reads	P10%	P14 reads	P14%
mmu-mir-181c-5p	8	86702823	86702844	-	edit	1	0	0.0%	0	0.0%	0	0.0%
mmu-mir-181c-5p	8	86702823	86702844	-	edit	2	0	0.0%	0	0.0%	0	0.0%
mmu-mir-181c-5p	8	86702823	86702844	-	edit	3	0	0.0%	0	0.0%	0	0.0%
mmu-mir-181c-5p	8	86702823	86702844	-	edit	4	0	0.0%	0	0.0%	0	0.0%
mmu-mir-181c-5p	8	86702823	86702844	-	edit	5	0	0.0%	0	0.0%	0	0.0%
mmu-mir-181c-5p	8	86702823	86702844	-	edit	6	0	0.0%	0	0.0%	0	0.0%
mmu-mir-181c-5p	8	86702823	86702844	-	edit	7	0	0.0%	0	0.0%	0	0.0%
mmu-mir-181c-5p	8	86702823	86702844	-	edit	8	0	0.0%	0	0.0%	0	0.0%
mmu-mir-181c-5p	8	86702823	86702844	-	edit	9	0	0.0%	0	0.0%	0	0.0%
mmu-mir-181c-5p	8	86702823	86702844	-	edit	10	0	0.0%	0	0.0%	0	0.0%
mmu-mir-181c-5p	8	86702823	86702844	-	edit	11	0	0.0%	0	0.0%	0	0.0%
mmu-mir-181c-5p	8	86702823	86702844	-	edit	12	0	0.0%	0	0.0%	0	0.0%
mmu-mir-181c-5p	8	86702823	86702844	-	edit	13	0	0.0%	0	0.0%	0	0.0%
mmu-mir-181c-5p	8	86702823	86702844	-	edit	14	0	0.0%	0	0.0%	0	0.0%
mmu-mir-181c-5p	8	86702823	86702844	-	edit	15	0	0.0%	0	0.0%	0	0.0%
mmu-mir-181c-5p	8	86702823	86702844	-	edit	16	0	0.0%	0	0.0%	0	0.0%
mmu-mir-181c-5p	8	86702823	86702844	-	edit	17	0	0.0%	0	0.0%	0	0.0%
mmu-mir-181c-5p	8	86702823	86702844	-	edit	18	0	0.0%	0	0.0%	0	0.0%
mmu-mir-181c-5p	8	86702823	86702844	-	edit	19	0	0.0%	0	0.0%	0	0.0%
mmu-mir-181c-5p	8	86702823	86702844	-	edit	20	0	0.0%	4	1.3%	0	0.0%
mmu-mir-181c-5p	8	86702823	86702844	-	edit	21	0	0.0%	0	0.0%	0	0.0%
mmu-mir-181c-5p	8	86702823	86702844	-	edit	22	0	0.0%	0	0.0%	0	0.0%
EDITED							0	0.0%	4	1.3%	0	0.0%
CANONICAL							759	100.0%	296	98.7%	117	100.0%
TOTAL							759		300		117	

miRNA	Chr	Start	Stop	Strand	Type	Position	P7 reads	P7%	P10 reads	P10%	P14 reads	P14%
mmu-mir-181d-5p	8	86702658	86702680	-	5' Edit	5'	23	0.1%	0	0.0%	4	0.1%
mmu-mir-181d-5p	8	86702658	86702680	-	5' Indel	5'	0	0.0%	0	0.0%	3	0.1%
mmu-mir-181d-5p	8	86702658	86702680	-	Indel	21	3	0.0%	0	0.0%	0	0.0%
mmu-mir-181d-5p	8	86702658	86702680	-	Indel	22	0	0.0%	3	0.0%	0	0.0%
mmu-mir-181d-5p	8	86702658	86702680	-	Indel	23	127	0.5%	30	0.4%	37	0.8%
mmu-mir-181d-5p	8	86702658	86702680	-	edit	1	0	0.0%	0	0.0%	0	0.0%
mmu-mir-181d-5p	8	86702658	86702680	-	edit	2	0	0.0%	0	0.0%	0	0.0%
mmu-mir-181d-5p	8	86702658	86702680	-	edit	3	0	0.0%	0	0.0%	0	0.0%
mmu-mir-181d-5p	8	86702658	86702680	-	edit	4	11	0.0%	11	0.2%	3	0.1%
mmu-mir-181d-5p	8	86702658	86702680	-	edit	5	0	0.0%	0	0.0%	0	0.0%
mmu-mir-181d-5p	8	86702658	86702680	-	edit	6	3	0.0%	0	0.0%	0	0.0%
mmu-mir-181d-5p	8	86702658	86702680	-	edit	7	0	0.0%	0	0.0%	0	0.0%
mmu-mir-181d-5p	8	86702658	86702680	-	edit	8	21	0.1%	7	0.1%	0	0.0%
mmu-mir-181d-5p	8	86702658	86702680	-	edit	9	0	0.0%	0	0.0%	0	0.0%
mmu-mir-181d-5p	8	86702658	86702680	-	edit	10	6	0.0%	0	0.0%	0	0.0%
mmu-mir-181d-5p	8	86702658	86702680	-	edit	11	0	0.0%	0	0.0%	0	0.0%
mmu-mir-181d-5p	8	86702658	86702680	-	edit	12	0	0.0%	0	0.0%	0	0.0%
mmu-mir-181d-5p	8	86702658	86702680	-	edit	13	7	0.0%	0	0.0%	0	0.0%
mmu-mir-181d-5p	8	86702658	86702680	-	edit	14	0	0.0%	0	0.0%	0	0.0%
mmu-mir-181d-5p	8	86702658	86702680	-	edit	15	77	0.3%	18	0.3%	8	0.2%
mmu-mir-181d-5p	8	86702658	86702680	-	edit	16	28	0.1%	14	0.2%	8	0.2%
mmu-mir-181d-5p	8	86702658	86702680	-	edit	17	31	0.1%	16	0.2%	5	0.1%
mmu-mir-181d-5p	8	86702658	86702680	-	edit	18	18	0.1%	4	0.1%	0	0.0%
mmu-mir-181d-5p	8	86702658	86702680	-	edit	19	752	3.1%	131	1.9%	175	3.9%
mmu-mir-181d-5p	8	86702658	86702680	-	edit	20	11	0.0%	3	0.0%	0	0.0%
mmu-mir-181d-5p	8	86702658	86702680	-	edit	21	40	0.2%	23	0.3%	3	0.1%
mmu-mir-181d-5p	8	86702658	86702680	-	edit	22	6	0.0%	0	0.0%	4	0.1%
mmu-mir-181d-5p	8	86702658	86702680	-	edit	23	37	0.2%	8	0.1%	9	0.2%
EDITED							1199	4.9%	267	3.9%	258	5.8%
CANONICAL							23294	95.1%	6626	96.1%	4197	94.2%
TOTAL							24493		6893		4455	

miRNA	Chr	Start	Stop	Strand	Type	Position	P7 reads	P7%	P10 reads	P10%	P14 reads	P14%
mmu-mir-182-5p	6	30115962	30115986	-	edit	1	0	0.0%	0	0.0%	0	0.0%
mmu-mir-182-5p	6	30115962	30115986	-	edit	2	0	0.0%	0	0.0%	0	0.0%
mmu-mir-182-5p	6	30115962	30115986	-	edit	3	0	0.0%	0	0.0%	0	0.0%
mmu-mir-182-5p	6	30115962	30115986	-	edit	4	0	0.0%	0	0.0%	0	0.0%
mmu-mir-182-5p	6	30115962	30115986	-	edit	5	0	0.0%	0	0.0%	0	0.0%
mmu-mir-182-5p	6	30115962	30115986	-	edit	6	0	0.0%	0	0.0%	0	0.0%
mmu-mir-182-5p	6	30115962	30115986	-	edit	7	0	0.0%	0	0.0%	0	0.0%
mmu-mir-182-5p	6	30115962	30115986	-	edit	8	0	0.0%	0	0.0%	0	0.0%
mmu-mir-182-5p	6	30115962	30115986	-	edit	9	0	0.0%	0	0.0%	0	0.0%
mmu-mir-182-5p	6	30115962	30115986	-	edit	10	0	0.0%	0	0.0%	0	0.0%
mmu-mir-182-5p	6	30115962	30115986	-	edit	11	0	0.0%	0	0.0%	0	0.0%
mmu-mir-182-5p	6	30115962	30115986	-	edit	12	0	0.0%	0	0.0%	0	0.0%
mmu-mir-182-5p	6	30115962	30115986	-	edit	13	0	0.0%	0	0.0%	0	0.0%
mmu-mir-182-5p	6	30115962	30115986	-	edit	14	0	0.0%	0	0.0%	0	0.0%
mmu-mir-182-5p	6	30115962	30115986	-	edit	15	0	0.0%	0	0.0%	0	0.0%
mmu-mir-182-5p	6	30115962	30115986	-	edit	16	0	0.0%	0	0.0%	0	0.0%
mmu-mir-182-5p	6	30115962	30115986	-	edit	17	0	0.0%	0	0.0%	0	0.0%
mmu-mir-182-5p	6	30115962	30115986	-	edit	18	0	0.0%	0	0.0%	0	0.0%
mmu-mir-182-5p	6	30115962	30115986	-	edit	19	0	0.0%	0	0.0%	0	0.0%
mmu-mir-182-5p	6	30115962	30115986	-	edit	20	0	0.0%	0	0.0%	0	0.0%
mmu-mir-182-5p	6	30115962	30115986	-	edit	21	0	0.0%	0	0.0%	0	0.0%
mmu-mir-182-5p	6	30115962	30115986	-	edit	22	0	0.0%	0	0.0%	0	0.0%
mmu-mir-182-5p	6	30115962	30115986	-	edit	23	3	2.1%	0	0.0%	0	0.0%
EDITED							3	2.1%	0	0.0%	0	0.0%
CANONICAL							139	97.9%	56	100.0%	27	100.0%
TOTAL							142		56		27	

miRNA	Chr	Start	Stop	Strand	Type	Position	P7 reads	P7%	P10 reads	P10%	P14 reads	P14%
mmu-mir-185-5p	16	18327531	18327552	-	5' Edit	5'	7	0.1%	0	0.0%	0	0.0%
mmu-mir-185-5p	16	18327531	18327552	-	edit	1	0	0.0%	0	0.0%	0	0.0%
mmu-mir-185-5p	16	18327531	18327552	-	edit	2	0	0.0%	0	0.0%	0	0.0%
mmu-mir-185-5p	16	18327531	18327552	-	edit	3	0	0.0%	0	0.0%	0	0.0%
mmu-mir-185-5p	16	18327531	18327552	-	edit	4	0	0.0%	0	0.0%	0	0.0%
mmu-mir-185-5p	16	18327531	18327552	-	edit	5	0	0.0%	0	0.0%	0	0.0%
mmu-mir-185-5p	16	18327531	18327552	-	edit	6	0	0.0%	0	0.0%	0	0.0%
mmu-mir-185-5p	16	18327531	18327552	-	edit	7	0	0.0%	0	0.0%	0	0.0%
mmu-mir-185-5p	16	18327531	18327552	-	edit	8	4	0.1%	0	0.0%	0	0.0%
mmu-mir-185-5p	16	18327531	18327552	-	edit	9	0	0.0%	0	0.0%	0	0.0%
mmu-mir-185-5p	16	18327531	18327552	-	edit	10	0	0.0%	0	0.0%	0	0.0%
mmu-mir-185-5p	16	18327531	18327552	-	edit	11	0	0.0%	0	0.0%	0	0.0%
mmu-mir-185-5p	16	18327531	18327552	-	edit	12	0	0.0%	0	0.0%	0	0.0%
mmu-mir-185-5p	16	18327531	18327552	-	edit	13	0	0.0%	0	0.0%	0	0.0%
mmu-mir-185-5p	16	18327531	18327552	-	edit	14	0	0.0%	0	0.0%	0	0.0%
mmu-mir-185-5p	16	18327531	18327552	-	edit	15	0	0.0%	0	0.0%	0	0.0%
mmu-mir-185-5p	16	18327531	18327552	-	edit	16	28	0.4%	17	0.3%	12	0.5%
mmu-mir-185-5p	16	18327531	18327552	-	edit	17	16	0.2%	8	0.1%	8	0.3%
mmu-mir-185-5p	16	18327531	18327552	-	edit	18	12	0.2%	37	0.7%	26	1.0%
mmu-mir-185-5p	16	18327531	18327552	-	edit	19	95	1.4%	57	1.1%	35	1.3%
mmu-mir-185-5p	16	18327531	18327552	-	edit	20	3	0.0%	0	0.0%	0	0.0%
mmu-mir-185-5p	16	18327531	18327552	-	edit	21	0	0.0%	0	0.0%	0	0.0%
mmu-mir-185-5p	16	18327531	18327552	-	edit	22	0	0.0%	8	0.1%	0	0.0%
EDITED							165	2.5%	126	2.4%	81	3.0%
CANONICAL							6476	97.5%	5028	97.6%	2595	97.0%
TOTAL							6641		5154		2676	

miRNA	Chr	Start	Stop	Strand	Type	Position	P7 reads	P7%	P10 reads	P10%	P14 reads	P14%
mmu-mir-191-5p	9	108470656	108470678	+	3' Edit	3'	0	0.0%	0	0.0%	3	0.0%
mmu-mir-191-5p	9	108470656	108470678	+	3' Indel	3'	54	0.5%	0	0.0%	62	0.7%
mmu-mir-191-5p	9	108470656	108470678	+	Indel	22	4	0.0%	0	0.0%	0	0.0%
mmu-mir-191-5p	9	108470656	108470678	+	Indel	23	0	0.0%	3	0.1%	0	0.0%
mmu-mir-191-5p	9	108470656	108470678	+	edit	1	0	0.0%	0	0.0%	0	0.0%
mmu-mir-191-5p	9	108470656	108470678	+	edit	2	0	0.0%	0	0.0%	0	0.0%
mmu-mir-191-5p	9	108470656	108470678	+	edit	3	0	0.0%	0	0.0%	0	0.0%
mmu-mir-191-5p	9	108470656	108470678	+	edit	4	5	0.0%	3	0.1%	8	0.1%
mmu-mir-191-5p	9	108470656	108470678	+	edit	5	0	0.0%	0	0.0%	0	0.0%
mmu-mir-191-5p	9	108470656	108470678	+	edit	6	4	0.0%	0	0.0%	5	0.1%
mmu-mir-191-5p	9	108470656	108470678	+	edit	7	0	0.0%	0	0.0%	0	0.0%
mmu-mir-191-5p	9	108470656	108470678	+	edit	8	5	0.0%	3	0.1%	0	0.0%
mmu-mir-191-5p	9	108470656	108470678	+	edit	9	0	0.0%	0	0.0%	0	0.0%
mmu-mir-191-5p	9	108470656	108470678	+	edit	10	25	0.2%	5	0.1%	31	0.3%
mmu-mir-191-5p	9	108470656	108470678	+	edit	11	3	0.0%	0	0.0%	6	0.1%
mmu-mir-191-5p	9	108470656	108470678	+	edit	12	4	0.0%	0	0.0%	7	0.1%
mmu-mir-191-5p	9	108470656	108470678	+	edit	13	0	0.0%	0	0.0%	8	0.1%
mmu-mir-191-5p	9	108470656	108470678	+	edit	14	0	0.0%	3	0.1%	0	0.0%
mmu-mir-191-5p	9	108470656	108470678	+	edit	15	15	0.1%	167	2.9%	21	0.2%
mmu-mir-191-5p	9	108470656	108470678	+	edit	16	0	0.0%	3	0.1%	0	0.0%
mmu-mir-191-5p	9	108470656	108470678	+	edit	17	13	0.1%	0	0.0%	10	0.1%
mmu-mir-191-5p	9	108470656	108470678	+	edit	18	0	0.0%	0	0.0%	0	0.0%
mmu-mir-191-5p	9	108470656	108470678	+	edit	19	30	0.3%	56	1.0%	33	0.3%
mmu-mir-191-5p	9	108470656	108470678	+	edit	20	0	0.0%	0	0.0%	0	0.0%
mmu-mir-191-5p	9	108470656	108470678	+	edit	21	47	0.5%	16	0.3%	74	0.8%
mmu-mir-191-5p	9	108470656	108470678	+	edit	22	20	0.2%	0	0.0%	21	0.2%
EDITED							229	2.2%	259	4.5%	288	3.0%
CANONICAL							9954	97.8%	5513	95.5%	9206	97.0%
TOTAL							10183		5772		9494	

miRNA	Chr	Start	Stop	Strand	Type	Position	P7 reads	P7%	P10 reads	P10%	P14 reads	P14%
mmu-mir-192-5p	19	6264857	6264877	+	Indel	21	0	0.0%	3	0.1%	12	0.4%
mmu-mir-192-5p	19	6264857	6264877	+	edit	1	0	0.0%	0	0.0%	0	0.0%
mmu-mir-192-5p	19	6264857	6264877	+	edit	2	0	0.0%	0	0.0%	0	0.0%
mmu-mir-192-5p	19	6264857	6264877	+	edit	3	0	0.0%	0	0.0%	0	0.0%
mmu-mir-192-5p	19	6264857	6264877	+	edit	4	0	0.0%	0	0.0%	0	0.0%
mmu-mir-192-5p	19	6264857	6264877	+	edit	5	0	0.0%	0	0.0%	0	0.0%
mmu-mir-192-5p	19	6264857	6264877	+	edit	6	0	0.0%	0	0.0%	5	0.1%
mmu-mir-192-5p	19	6264857	6264877	+	edit	7	0	0.0%	0	0.0%	0	0.0%
mmu-mir-192-5p	19	6264857	6264877	+	edit	8	0	0.0%	0	0.0%	0	0.0%
mmu-mir-192-5p	19	6264857	6264877	+	edit	9	0	0.0%	0	0.0%	0	0.0%
mmu-mir-192-5p	19	6264857	6264877	+	edit	10	0	0.0%	0	0.0%	0	0.0%
mmu-mir-192-5p	19	6264857	6264877	+	edit	11	0	0.0%	0	0.0%	0	0.0%
mmu-mir-192-5p	19	6264857	6264877	+	edit	12	0	0.0%	0	0.0%	0	0.0%
mmu-mir-192-5p	19	6264857	6264877	+	edit	13	0	0.0%	0	0.0%	0	0.0%
mmu-mir-192-5p	19	6264857	6264877	+	edit	14	0	0.0%	0	0.0%	0	0.0%
mmu-mir-192-5p	19	6264857	6264877	+	edit	15	0	0.0%	0	0.0%	7	0.2%
mmu-mir-192-5p	19	6264857	6264877	+	edit	16	0	0.0%	0	0.0%	0	0.0%
mmu-mir-192-5p	19	6264857	6264877	+	edit	17	0	0.0%	0	0.0%	0	0.0%
mmu-mir-192-5p	19	6264857	6264877	+	edit	18	0	0.0%	0	0.0%	0	0.0%
mmu-mir-192-5p	19	6264857	6264877	+	edit	19	3	0.1%	3	0.1%	5	0.1%
mmu-mir-192-5p	19	6264857	6264877	+	edit	20	0	0.0%	9	0.4%	8	0.2%
mmu-mir-192-5p	19	6264857	6264877	+	edit	21	0	0.0%	0	0.0%	0	0.0%
mmu-mir-192-5p	19	6264857	6264877	+	edit	22	0	0.0%	0	0.0%	0	0.0%
EDITED							3	0.1%	15	0.7%	37	1.1%
CANONICAL							2982	99.9%	2233	99.3%	3389	98.9%
TOTAL							2985		2248		3426	

miRNA	Chr	Start	Stop	Strand	Type	Position	P7 reads	P7%	P10 reads	P10%	P14 reads	P14%
mmu-mir-193-3p	11	79525511	79525532	+	edit	1	0	0.0%	0	0.0%	0	0.0%
mmu-mir-193-3p	11	79525511	79525532	+	edit	2	0	0.0%	0	0.0%	0	0.0%
mmu-mir-193-3p	11	79525511	79525532	+	edit	3	0	0.0%	0	0.0%	0	0.0%
mmu-mir-193-3p	11	79525511	79525532	+	edit	4	0	0.0%	0	0.0%	**3**	**6.0%**
mmu-mir-193-3p	11	79525511	79525532	+	edit	5	0	0.0%	0	0.0%	0	0.0%
mmu-mir-193-3p	11	79525511	79525532	+	edit	6	0	0.0%	0	0.0%	0	0.0%
mmu-mir-193-3p	11	79525511	79525532	+	edit	7	0	0.0%	0	0.0%	0	0.0%
mmu-mir-193-3p	11	79525511	79525532	+	edit	8	0	0.0%	0	0.0%	0	0.0%
mmu-mir-193-3p	11	79525511	79525532	+	edit	9	0	0.0%	0	0.0%	0	0.0%
mmu-mir-193-3p	11	79525511	79525532	+	edit	10	0	0.0%	0	0.0%	0	0.0%
mmu-mir-193-3p	11	79525511	79525532	+	edit	11	0	0.0%	0	0.0%	0	0.0%
mmu-mir-193-3p	11	79525511	79525532	+	edit	12	0	0.0%	0	0.0%	0	0.0%
mmu-mir-193-3p	11	79525511	79525532	+	edit	13	0	0.0%	0	0.0%	0	0.0%
mmu-mir-193-3p	11	79525511	79525532	+	edit	14	0	0.0%	0	0.0%	0	0.0%
mmu-mir-193-3p	11	79525511	79525532	+	edit	15	0	0.0%	0	0.0%	0	0.0%
mmu-mir-193-3p	11	79525511	79525532	+	edit	16	0	0.0%	0	0.0%	0	0.0%
mmu-mir-193-3p	11	79525511	79525532	+	edit	17	0	0.0%	0	0.0%	0	0.0%
mmu-mir-193-3p	11	79525511	79525532	+	edit	18	0	0.0%	0	0.0%	0	0.0%
mmu-mir-193-3p	11	79525511	79525532	+	edit	19	0	0.0%	0	0.0%	0	0.0%
mmu-mir-193-3p	11	79525511	79525532	+	edit	20	0	0.0%	0	0.0%	0	0.0%
mmu-mir-193-3p	11	79525511	79525532	+	edit	21	0	0.0%	0	0.0%	0	0.0%
mmu-mir-193-3p	11	79525511	79525532	+	edit	22	0	0.0%	0	0.0%	0	0.0%
EDITED							0	0.0%	0	0.0%	3	6.0%
CANONICAL							109	100.0%	72	100.0%	47	94.0%
TOTAL							109		72		50	

miRNA	Chr	Start	Stop	Strand	Type	Position	P7 reads	P7%	P10 reads	P10%	P14 reads	P14%
mmu-mir-199a-1-3p	9	21300942	21300963	-	5' Edit	5'	6	0.0%	0	0.0%	0	0.0%
mmu-mir-199a-1-3p	9	21300942	21300963	-	edit	1	0	0.0%	0	0.0%	0	0.0%
mmu-mir-199a-1-3p	9	21300942	21300963	-	edit	2	0	0.0%	0	0.0%	0	0.0%
mmu-mir-199a-1-3p	9	21300942	21300963	-	edit	3	0	0.0%	0	0.0%	0	0.0%
mmu-mir-199a-1-3p	9	21300942	21300963	-	edit	4	16	0.0%	3	0.0%	0	0.0%
mmu-mir-199a-1-3p	9	21300942	21300963	-	edit	5	6	0.0%	0	0.0%	0	0.0%
mmu-mir-199a-1-3p	9	21300942	21300963	-	edit	6	13	0.0%	0	0.0%	0	0.0%
mmu-mir-199a-1-3p	9	21300942	21300963	-	edit	7	3	0.0%	0	0.0%	0	0.0%
mmu-mir-199a-1-3p	9	21300942	21300963	-	edit	8	130	0.2%	18	0.1%	19	0.1%
mmu-mir-199a-1-3p	9	21300942	21300963	-	edit	9	0	0.0%	0	0.0%	0	0.0%
mmu-mir-199a-1-3p	9	21300942	21300963	-	edit	10	0	0.0%	0	0.0%	0	0.0%
mmu-mir-199a-1-3p	9	21300942	21300963	-	edit	11	0	0.0%	0	0.0%	0	0.0%
mmu-mir-199a-1-3p	9	21300942	21300963	-	edit	12	0	0.0%	0	0.0%	0	0.0%
mmu-mir-199a-1-3p	9	21300942	21300963	-	edit	13	0	0.0%	0	0.0%	0	0.0%
mmu-mir-199a-1-3p	9	21300942	21300963	-	edit	14	12	0.0%	4	0.0%	0	0.0%
mmu-mir-199a-1-3p	9	21300942	21300963	-	edit	15	98	0.1%	269	0.9%	21	0.1%
mmu-mir-199a-1-3p	9	21300942	21300963	-	edit	16	136	0.2%	21	0.1%	23	0.1%
mmu-mir-199a-1-3p	9	21300942	21300963	-	edit	17	69	0.1%	18	0.1%	17	0.1%
mmu-mir-199a-1-3p	9	21300942	21300963	-	edit	18	8	0.0%	4	0.0%	0	0.0%
mmu-mir-199a-1-3p	9	21300942	21300963	-	edit	19	101	0.1%	41	0.1%	11	0.1%
mmu-mir-199a-1-3p	9	21300942	21300963	-	edit	20	0	0.0%	0	0.0%	0	0.0%
mmu-mir-199a-1-3p	9	21300942	21300963	-	edit	21	0	0.0%	0	0.0%	0	0.0%
mmu-mir-199a-1-3p	9	21300942	21300963	-	edit	22	7	0.0%	0	0.0%	0	0.0%
EDITED							605	0.8%	377	1.2%	90	0.5%
CANONICAL							77201	99.2%	29801	98.8%	16413	99.5%
TOTAL							77806		30178		16503	

miRNA	Chr	Start	Stop	Strand	Type	Position	P7 reads	P7%	P10 reads	P10%	P14 reads	P14%
mmu-mir-199a-1-5p	9	21300981	21301003	-	edit	1	0	0.0%	0	0.0%	0	0.0%
mmu-mir-199a-1-5p	9	21300981	21301003	-	edit	2	0	0.0%	0	0.0%	0	0.0%
mmu-mir-199a-1-5p	9	21300981	21301003	-	edit	3	0	0.0%	0	0.0%	0	0.0%
mmu-mir-199a-1-5p	9	21300981	21301003	-	edit	4	0	0.0%	0	0.0%	0	0.0%
mmu-mir-199a-1-5p	9	21300981	21301003	-	edit	5	0	0.0%	0	0.0%	0	0.0%
mmu-mir-199a-1-5p	9	21300981	21301003	-	edit	6	0	0.0%	0	0.0%	0	0.0%
mmu-mir-199a-1-5p	9	21300981	21301003	-	edit	7	0	0.0%	0	0.0%	0	0.0%
mmu-mir-199a-1-5p	9	21300981	21301003	-	edit	8	0	0.0%	0	0.0%	0	0.0%
mmu-mir-199a-1-5p	9	21300981	21301003	-	edit	9	0	0.0%	0	0.0%	0	0.0%
mmu-mir-199a-1-5p	9	21300981	21301003	-	edit	10	0	0.0%	0	0.0%	0	0.0%
mmu-mir-199a-1-5p	9	21300981	21301003	-	edit	11	0	0.0%	0	0.0%	0	0.0%
mmu-mir-199a-1-5p	9	21300981	21301003	-	edit	12	0	0.0%	0	0.0%	0	0.0%
mmu-mir-199a-1-5p	9	21300981	21301003	-	edit	13	0	0.0%	0	0.0%	0	0.0%
mmu-mir-199a-1-5p	9	21300981	21301003	-	edit	14	0	0.0%	0	0.0%	0	0.0%
mmu-mir-199a-1-5p	9	21300981	21301003	-	edit	15	0	0.0%	0	0.0%	0	0.0%
mmu-mir-199a-1-5p	9	21300981	21301003	-	edit	16	0	0.0%	0	0.0%	0	0.0%
mmu-mir-199a-1-5p	9	21300981	21301003	-	edit	17	**11**	**3.2%**	**7**	**4.7%**	**4**	**4.7%**
mmu-mir-199a-1-5p	9	21300981	21301003	-	edit	18	0	0.0%	0	0.0%	0	0.0%
mmu-mir-199a-1-5p	9	21300981	21301003	-	edit	19	0	0.0%	0	0.0%	0	0.0%
mmu-mir-199a-1-5p	9	21300981	21301003	-	edit	20	0	0.0%	0	0.0%	0	0.0%
mmu-mir-199a-1-5p	9	21300981	21301003	-	edit	21	0	0.0%	0	0.0%	0	0.0%
mmu-mir-199a-1-5p	9	21300981	21301003	-	edit	22	0	0.0%	0	0.0%	0	0.0%
EDITED							11	3.2%	7	4.7%	4	4.7%
CANONICAL							331	96.8%	142	95.3%	82	95.3%
TOTAL							342		149		86	

miRNA	Chr	Start	Stop	Strand	Type	Position	P7 reads	P7%	P10 reads	P10%	P14 reads	P14%
mmu-mir-199a-2-3p	1	164148014	164148035	+	3' Edit	3'	58	0.1%	0	0.0%	15	0.1%
mmu-mir-199a-2-3p	1	164148014	164148035	+	Indel	19	16	0.0%	3	0.0%	0	0.0%
mmu-mir-199a-2-3p	1	164148014	164148035	+	Indel	20	15	0.0%	0	0.0%	3	0.0%
mmu-mir-199a-2-3p	1	164148014	164148035	+	Indel	21	0	0.0%	0	0.0%	0	0.0%
mmu-mir-199a-2-3p	1	164148014	164148035	+	Indel	22	13	0.0%	0	0.0%	0	0.0%
mmu-mir-199a-2-3p	1	164148014	164148035	+	edit	1	0	0.0%	0	0.0%	0	0.0%
mmu-mir-199a-2-3p	1	164148014	164148035	+	edit	2	0	0.0%	0	0.0%	0	0.0%
mmu-mir-199a-2-3p	1	164148014	164148035	+	edit	3	0	0.0%	0	0.0%	0	0.0%
mmu-mir-199a-2-3p	1	164148014	164148035	+	edit	4	16	0.0%	3	0.0%	0	0.0%
mmu-mir-199a-2-3p	1	164148014	164148035	+	edit	5	6	0.0%	0	0.0%	0	0.0%
mmu-mir-199a-2-3p	1	164148014	164148035	+	edit	6	13	0.0%	0	0.0%	0	0.0%
mmu-mir-199a-2-3p	1	164148014	164148035	+	edit	7	3	0.0%	0	0.0%	0	0.0%
mmu-mir-199a-2-3p	1	164148014	164148035	+	edit	8	130	0.2%	18	0.1%	19	0.1%
mmu-mir-199a-2-3p	1	164148014	164148035	+	edit	9	0	0.0%	0	0.0%	0	0.0%
mmu-mir-199a-2-3p	1	164148014	164148035	+	edit	10	0	0.0%	0	0.0%	0	0.0%
mmu-mir-199a-2-3p	1	164148014	164148035	+	edit	11	0	0.0%	0	0.0%	0	0.0%
mmu-mir-199a-2-3p	1	164148014	164148035	+	edit	12	0	0.0%	0	0.0%	0	0.0%
mmu-mir-199a-2-3p	1	164148014	164148035	+	edit	13	0	0.0%	0	0.0%	0	0.0%
mmu-mir-199a-2-3p	1	164148014	164148035	+	edit	14	12	0.0%	4	0.0%	3	0.0%
mmu-mir-199a-2-3p	1	164148014	164148035	+	edit	15	99	0.1%	268	0.9%	21	0.1%
mmu-mir-199a-2-3p	1	164148014	164148035	+	edit	16	136	0.2%	21	0.1%	23	0.1%
mmu-mir-199a-2-3p	1	164148014	164148035	+	edit	17	68	0.1%	19	0.1%	17	0.1%
mmu-mir-199a-2-3p	1	164148014	164148035	+	edit	18	8	0.0%	4	0.0%	0	0.0%
mmu-mir-199a-2-3p	1	164148014	164148035	+	edit	19	102	0.1%	41	0.1%	11	0.1%
mmu-mir-199a-2-3p	1	164148014	164148035	+	edit	20	0	0.0%	0	0.0%	0	0.0%
mmu-mir-199a-2-3p	1	164148014	164148035	+	edit	21	3	0.0%	0	0.0%	0	0.0%
mmu-mir-199a-2-3p	1	164148014	164148035	+	edit	22	11	0.0%	0	0.0%	0	0.0%
EDITED							709	0.9%	380	1.3%	111	0.7%
CANONICAL							76979	99.1%	29732	98.7%	16386	99.3%
TOTAL							77688		30112		16497	

miRNA	Chr	Start	Stop	Strand	Type	Position	P7 reads	P7%	P10 reads	P10%	P14 reads	P14%
mmu-mir-199a-2-5p	1	164147975	164147997	+	edit	1	0	0.0%	0	0.0%	0	0.0%
mmu-mir-199a-2-5p	1	164147975	164147997	+	edit	2	0	0.0%	0	0.0%	0	0.0%
mmu-mir-199a-2-5p	1	164147975	164147997	+	edit	3	0	0.0%	0	0.0%	0	0.0%
mmu-mir-199a-2-5p	1	164147975	164147997	+	edit	4	0	0.0%	0	0.0%	0	0.0%
mmu-mir-199a-2-5p	1	164147975	164147997	+	edit	5	0	0.0%	0	0.0%	0	0.0%
mmu-mir-199a-2-5p	1	164147975	164147997	+	edit	6	0	0.0%	0	0.0%	0	0.0%
mmu-mir-199a-2-5p	1	164147975	164147997	+	edit	7	0	0.0%	0	0.0%	0	0.0%
mmu-mir-199a-2-5p	1	164147975	164147997	+	edit	8	0	0.0%	0	0.0%	0	0.0%
mmu-mir-199a-2-5p	1	164147975	164147997	+	edit	9	0	0.0%	0	0.0%	0	0.0%
mmu-mir-199a-2-5p	1	164147975	164147997	+	edit	10	0	0.0%	0	0.0%	0	0.0%
mmu-mir-199a-2-5p	1	164147975	164147997	+	edit	11	0	0.0%	0	0.0%	0	0.0%
mmu-mir-199a-2-5p	1	164147975	164147997	+	edit	12	0	0.0%	0	0.0%	0	0.0%
mmu-mir-199a-2-5p	1	164147975	164147997	+	edit	13	0	0.0%	0	0.0%	0	0.0%
mmu-mir-199a-2-5p	1	164147975	164147997	+	edit	14	0	0.0%	0	0.0%	0	0.0%
mmu-mir-199a-2-5p	1	164147975	164147997	+	edit	15	0	0.0%	0	0.0%	0	0.0%
mmu-mir-199a-2-5p	1	164147975	164147997	+	edit	16	0	0.0%	0	0.0%	0	0.0%
mmu-mir-199a-2-5p	1	164147975	164147997	+	edit	17	**11**	**3.4%**	**7**	**4.9%**	**4**	**5.1%**
mmu-mir-199a-2-5p	1	164147975	164147997	+	edit	18	0	0.0%	0	0.0%	0	0.0%
mmu-mir-199a-2-5p	1	164147975	164147997	+	edit	19	0	0.0%	0	0.0%	0	0.0%
mmu-mir-199a-2-5p	1	164147975	164147997	+	edit	20	0	0.0%	0	0.0%	0	0.0%
mmu-mir-199a-2-5p	1	164147975	164147997	+	edit	21	0	0.0%	0	0.0%	0	0.0%
mmu-mir-199a-2-5p	1	164147975	164147997	+	edit	22	0	0.0%	0	0.0%	0	0.0%
EDITED							11	3.4%	7	4.9%	4	5.1%
CANONICAL							311	96.6%	136	95.1%	74	94.9%
TOTAL							322		143		78	

miRNA	Chr	Start	Stop	Strand	Type	Position	P7 reads	P7%	P10 reads	P10%	P14 reads	P14%
mmu-mir-199b-3p	2	32174044	32174065	+	3' Edit	3'	5	0.0%	0	0.0%	0	0.0%
mmu-mir-199b-3p	2	32174044	32174065	+	edit	1	0	0.0%	0	0.0%	0	0.0%
mmu-mir-199b-3p	2	32174044	32174065	+	edit	2	0	0.0%	0	0.0%	0	0.0%
mmu-mir-199b-3p	2	32174044	32174065	+	edit	3	0	0.0%	0	0.0%	0	0.0%
mmu-mir-199b-3p	2	32174044	32174065	+	edit	4	16	0.0%	3	0.0%	0	0.0%
mmu-mir-199b-3p	2	32174044	32174065	+	edit	5	6	0.0%	0	0.0%	0	0.0%
mmu-mir-199b-3p	2	32174044	32174065	+	edit	6	13	0.0%	0	0.0%	0	0.0%
mmu-mir-199b-3p	2	32174044	32174065	+	edit	7	3	0.0%	0	0.0%	0	0.0%
mmu-mir-199b-3p	2	32174044	32174065	+	edit	8	130	0.2%	18	0.1%	19	0.1%
mmu-mir-199b-3p	2	32174044	32174065	+	edit	9	0	0.0%	0	0.0%	0	0.0%
mmu-mir-199b-3p	2	32174044	32174065	+	edit	10	0	0.0%	0	0.0%	0	0.0%
mmu-mir-199b-3p	2	32174044	32174065	+	edit	11	0	0.0%	0	0.0%	0	0.0%
mmu-mir-199b-3p	2	32174044	32174065	+	edit	12	0	0.0%	0	0.0%	0	0.0%
mmu-mir-199b-3p	2	32174044	32174065	+	edit	13	0	0.0%	0	0.0%	0	0.0%
mmu-mir-199b-3p	2	32174044	32174065	+	edit	14	12	0.0%	4	0.0%	0	0.0%
mmu-mir-199b-3p	2	32174044	32174065	+	edit	15	98	0.1%	269	0.9%	21	0.1%
mmu-mir-199b-3p	2	32174044	32174065	+	edit	16	136	0.2%	21	0.1%	23	0.1%
mmu-mir-199b-3p	2	32174044	32174065	+	edit	17	69	0.1%	18	0.1%	17	0.1%
mmu-mir-199b-3p	2	32174044	32174065	+	edit	18	8	0.0%	4	0.0%	0	0.0%
mmu-mir-199b-3p	2	32174044	32174065	+	edit	19	101	0.1%	41	0.1%	11	0.1%
mmu-mir-199b-3p	2	32174044	32174065	+	edit	20	0	0.0%	0	0.0%	0	0.0%
mmu-mir-199b-3p	2	32174044	32174065	+	edit	21	0	0.0%	0	0.0%	0	0.0%
mmu-mir-199b-3p	2	32174044	32174065	+	edit	22	7	0.0%	0	0.0%	0	0.0%
EDITED							604	0.8%	377	1.3%	90	0.5%
CANONICAL							76833	99.2%	29698	98.7%	16354	99.5%
TOTAL							77437		30075		16444	

miRNA	Chr	Start	Stop	Strand	Type	Position	P7 reads	P7%	P10 reads	P10%	P14 reads	P14%
mmu-mir-200a-3p	4	155429020	155429041	-	edit	1	0	0.0%	0	0.0%	0	0.0%
mmu-mir-200a-3p	4	155429020	155429041	-	edit	2	0	0.0%	0	0.0%	0	0.0%
mmu-mir-200a-3p	4	155429020	155429041	-	edit	3	0	0.0%	0	0.0%	0	0.0%
mmu-mir-200a-3p	4	155429020	155429041	-	edit	4	0	0.0%	0	0.0%	0	0.0%
mmu-mir-200a-3p	4	155429020	155429041	-	edit	5	0	0.0%	0	0.0%	0	0.0%
mmu-mir-200a-3p	4	155429020	155429041	-	edit	6	0	0.0%	0	0.0%	0	0.0%
mmu-mir-200a-3p	4	155429020	155429041	-	edit	7	0	0.0%	0	0.0%	0	0.0%
mmu-mir-200a-3p	4	155429020	155429041	-	edit	8	0	0.0%	0	0.0%	0	0.0%
mmu-mir-200a-3p	4	155429020	155429041	-	edit	9	0	0.0%	0	0.0%	0	0.0%
mmu-mir-200a-3p	4	155429020	155429041	-	edit	10	0	0.0%	0	0.0%	0	0.0%
mmu-mir-200a-3p	4	155429020	155429041	-	edit	11	0	0.0%	0	0.0%	0	0.0%
mmu-mir-200a-3p	4	155429020	155429041	-	edit	12	0	0.0%	0	0.0%	0	0.0%
mmu-mir-200a-3p	4	155429020	155429041	-	edit	13	0	0.0%	0	0.0%	0	0.0%
mmu-mir-200a-3p	4	155429020	155429041	-	edit	14	0	0.0%	0	0.0%	0	0.0%
mmu-mir-200a-3p	4	155429020	155429041	-	edit	15	0	0.0%	4	1.2%	0	0.0%
mmu-mir-200a-3p	4	155429020	155429041	-	edit	16	0	0.0%	0	0.0%	0	0.0%
mmu-mir-200a-3p	4	155429020	155429041	-	edit	17	0	0.0%	0	0.0%	0	0.0%
mmu-mir-200a-3p	4	155429020	155429041	-	edit	18	0	0.0%	0	0.0%	0	0.0%
mmu-mir-200a-3p	4	155429020	155429041	-	edit	19	0	0.0%	4	1.3%	0	0.0%
mmu-mir-200a-3p	4	155429020	155429041	-	edit	20	0	0.0%	0	0.0%	0	0.0%
mmu-mir-200a-3p	4	155429020	155429041	-	edit	21	0	0.0%	0	0.0%	0	0.0%
mmu-mir-200a-3p	4	155429020	155429041	-	edit	22	0	0.0%	0	0.0%	0	0.0%
EDITED							0	0.0%	8	2.5%	0	0.0%
CANONICAL							284	100.0%	296	97.5%	247	100.0%
TOTAL							284		304		247	

miRNA	Chr	Start	Stop	Strand	Type	Position	P7 reads	P7%	P10 reads	P10%	P14 reads	P14%
mmu-mir-202-3p	7	147143594	147143615	-	edit	1	0	0.0%	0	0.0%	0	0.0%
mmu-mir-202-3p	7	147143594	147143615	-	edit	2	0	0.0%	0	0.0%	0	0.0%
mmu-mir-202-3p	7	147143594	147143615	-	edit	3	0	0.0%	0	0.0%	0	0.0%
mmu-mir-202-3p	7	147143594	147143615	-	edit	4	0	0.0%	0	0.0%	0	0.0%
mmu-mir-202-3p	7	147143594	147143615	-	edit	5	0	0.0%	0	0.0%	0	0.0%
mmu-mir-202-3p	7	147143594	147143615	-	edit	6	0	0.0%	0	0.0%	0	0.0%
mmu-mir-202-3p	7	147143594	147143615	-	edit	7	0	0.0%	0	0.0%	0	0.0%
mmu-mir-202-3p	7	147143594	147143615	-	edit	8	0	0.0%	0	0.0%	0	0.0%
mmu-mir-202-3p	7	147143594	147143615	-	edit	9	0	0.0%	0	0.0%	0	0.0%
mmu-mir-202-3p	7	147143594	147143615	-	edit	10	0	0.0%	0	0.0%	0	0.0%
mmu-mir-202-3p	7	147143594	147143615	-	edit	11	0	0.0%	0	0.0%	0	0.0%
mmu-mir-202-3p	7	147143594	147143615	-	edit	12	0	0.0%	0	0.0%	0	0.0%
mmu-mir-202-3p	7	147143594	147143615	-	edit	13	0	0.0%	0	0.0%	0	0.0%
mmu-mir-202-3p	7	147143594	147143615	-	edit	14	0	0.0%	0	0.0%	0	0.0%
mmu-mir-202-3p	7	147143594	147143615	-	edit	15	0	0.0%	0	0.0%	0	0.0%
mmu-mir-202-3p	7	147143594	147143615	-	edit	16	0	0.0%	0	0.0%	0	0.0%
mmu-mir-202-3p	7	147143594	147143615	-	edit	17	0	0.0%	0	0.0%	0	0.0%
mmu-mir-202-3p	7	147143594	147143615	-	edit	18	0	0.0%	0	0.0%	0	0.0%
mmu-mir-202-3p	7	147143594	147143615	-	edit	19	9	0.9%	21	3.8%	0	0.0%
mmu-mir-202-3p	7	147143594	147143615	-	edit	20	0	0.0%	0	0.0%	0	0.0%
mmu-mir-202-3p	7	147143594	147143615	-	edit	21	0	0.0%	0	0.0%	0	0.0%
mmu-mir-202-3p	7	147143594	147143615	-	edit	22	0	0.0%	0	0.0%	0	0.0%
EDITED							9	0.9%	21	3.8%	0	0.0%
CANONICAL							985	99.1%	539	96.3%	227	100.0%
TOTAL							994		560		227	

miRNA	Chr	Start	Stop	Strand	Type	Position	P7 reads	P7%	P10 reads	P10%	P14 reads	P14%
mmu-mir-202-5p	7	147143631	147143651	-	edit	1	0	0.0%	0	0.0%	0	0.0%
mmu-mir-202-5p	7	147143631	147143651	-	edit	2	0	0.0%	0	0.0%	0	0.0%
mmu-mir-202-5p	7	147143631	147143651	-	edit	3	0	0.0%	0	0.0%	0	0.0%
mmu-mir-202-5p	7	147143631	147143651	-	edit	4	0	0.0%	0	0.0%	0	0.0%
mmu-mir-202-5p	7	147143631	147143651	-	edit	5	0	0.0%	0	0.0%	0	0.0%
mmu-mir-202-5p	7	147143631	147143651	-	edit	6	0	0.0%	0	0.0%	0	0.0%
mmu-mir-202-5p	7	147143631	147143651	-	edit	7	0	0.0%	0	0.0%	0	0.0%
mmu-mir-202-5p	7	147143631	147143651	-	edit	8	0	0.0%	0	0.0%	0	0.0%
mmu-mir-202-5p	7	147143631	147143651	-	edit	9	0	0.0%	0	0.0%	0	0.0%
mmu-mir-202-5p	7	147143631	147143651	-	edit	10	0	0.0%	0	0.0%	0	0.0%
mmu-mir-202-5p	7	147143631	147143651	-	edit	11	0	0.0%	0	0.0%	0	0.0%
mmu-mir-202-5p	7	147143631	147143651	-	edit	12	0	0.0%	0	0.0%	0	0.0%
mmu-mir-202-5p	7	147143631	147143651	-	edit	13	0	0.0%	0	0.0%	0	0.0%
mmu-mir-202-5p	7	147143631	147143651	-	edit	14	0	0.0%	0	0.0%	0	0.0%
mmu-mir-202-5p	7	147143631	147143651	-	edit	15	0	0.0%	0	0.0%	0	0.0%
mmu-mir-202-5p	7	147143631	147143651	-	edit	16	24	0.1%	15	0.0%	10	0.1%
mmu-mir-202-5p	7	147143631	147143651	-	edit	17	0	0.0%	0	0.0%	0	0.0%
mmu-mir-202-5p	7	147143631	147143651	-	edit	18	5	0.0%	4	0.0%	0	0.0%
mmu-mir-202-5p	7	147143631	147143651	-	edit	19	0	0.0%	0	0.0%	0	0.0%
mmu-mir-202-5p	7	147143631	147143651	-	edit	20	0	0.0%	0	0.0%	0	0.0%
mmu-mir-202-5p	7	147143631	147143651	-	edit	21	0	0.0%	0	0.0%	0	0.0%
mmu-mir-202-5p	7	147143631	147143651	-	edit	22	0	0.0%	0	0.0%	0	0.0%
EDITED							29	0.1%	19	0.1%	10	0.1%
CANONICAL							25730	99.9%	30465	99.9%	14298	99.9%
TOTAL							25759		30484		14308	

miRNA	Chr	Start	Stop	Strand	Type	Position	P7 reads	P7%	P10 reads	P10%	P14 reads	P14%
mmu-mir-203-3p	12	113369138	113369159	+	edit	1	0	0.0%	0	0.0%	0	0.0%
mmu-mir-203-3p	12	113369138	113369159	+	edit	2	0	0.0%	0	0.0%	0	0.0%
mmu-mir-203-3p	12	113369138	113369159	+	edit	3	0	0.0%	0	0.0%	0	0.0%
mmu-mir-203-3p	12	113369138	113369159	+	edit	4	0	0.0%	0	0.0%	0	0.0%
mmu-mir-203-3p	12	113369138	113369159	+	edit	5	0	0.0%	0	0.0%	0	0.0%
mmu-mir-203-3p	12	113369138	113369159	+	edit	6	0	0.0%	0	0.0%	0	0.0%
mmu-mir-203-3p	12	113369138	113369159	+	edit	7	0	0.0%	0	0.0%	0	0.0%
mmu-mir-203-3p	12	113369138	113369159	+	edit	8	0	0.0%	0	0.0%	0	0.0%
mmu-mir-203-3p	12	113369138	113369159	+	edit	9	0	0.0%	0	0.0%	0	0.0%
mmu-mir-203-3p	12	113369138	113369159	+	edit	10	0	0.0%	0	0.0%	0	0.0%
mmu-mir-203-3p	12	113369138	113369159	+	edit	11	0	0.0%	0	0.0%	0	0.0%
mmu-mir-203-3p	12	113369138	113369159	+	edit	12	0	0.0%	0	0.0%	0	0.0%
mmu-mir-203-3p	12	113369138	113369159	+	edit	13	0	0.0%	0	0.0%	0	0.0%
mmu-mir-203-3p	12	113369138	113369159	+	edit	14	0	0.0%	0	0.0%	0	0.0%
mmu-mir-203-3p	12	113369138	113369159	+	edit	15	0	0.0%	0	0.0%	0	0.0%
mmu-mir-203-3p	12	113369138	113369159	+	edit	16	0	0.0%	3	0.5%	7	1.2%
mmu-mir-203-3p	12	113369138	113369159	+	edit	17	0	0.0%	0	0.0%	0	0.0%
mmu-mir-203-3p	12	113369138	113369159	+	edit	18	0	0.0%	0	0.0%	0	0.0%
mmu-mir-203-3p	12	113369138	113369159	+	edit	19	0	0.0%	6	0.9%	4	0.7%
mmu-mir-203-3p	12	113369138	113369159	+	edit	20	0	0.0%	0	0.0%	0	0.0%
mmu-mir-203-3p	12	113369138	113369159	+	edit	21	0	0.0%	0	0.0%	0	0.0%
mmu-mir-203-3p	12	113369138	113369159	+	edit	22	0	0.0%	0	0.0%	0	0.0%
EDITED							0	0.0%	9	1.4%	11	1.9%
CANONICAL							509	100.0%	651	98.6%	546	98.1%
TOTAL							509		660		557	

miRNA	Chr	Start	Stop	Strand	Type	Position	P7 reads	P7%	P10 reads	P10%	P14 reads	P14%
mmu-mir-210-5p	7	148407306	148407327	-	edit	1	0	0.0%	0	0.0%	0	0.0%
mmu-mir-210-5p	7	148407306	148407327	-	edit	2	0	0.0%	0	0.0%	0	0.0%
mmu-mir-210-5p	7	148407306	148407327	-	edit	3	0	0.0%	0	0.0%	0	0.0%
mmu-mir-210-5p	7	148407306	148407327	-	edit	4	0	0.0%	0	0.0%	0	0.0%
mmu-mir-210-5p	7	148407306	148407327	-	edit	5	0	0.0%	0	0.0%	0	0.0%
mmu-mir-210-5p	7	148407306	148407327	-	edit	6	0	0.0%	0	0.0%	0	0.0%
mmu-mir-210-5p	7	148407306	148407327	-	edit	7	0	0.0%	0	0.0%	0	0.0%
mmu-mir-210-5p	7	148407306	148407327	-	edit	8	0	0.0%	0	0.0%	0	0.0%
mmu-mir-210-5p	7	148407306	148407327	-	edit	9	0	0.0%	0	0.0%	0	0.0%
mmu-mir-210-5p	7	148407306	148407327	-	edit	10	0	0.0%	0	0.0%	0	0.0%
mmu-mir-210-5p	7	148407306	148407327	-	edit	11	0	0.0%	0	0.0%	0	0.0%
mmu-mir-210-5p	7	148407306	148407327	-	edit	12	0	0.0%	0	0.0%	0	0.0%
mmu-mir-210-5p	7	148407306	148407327	-	edit	13	0	0.0%	0	0.0%	0	0.0%
mmu-mir-210-5p	7	148407306	148407327	-	edit	14	0	0.0%	0	0.0%	0	0.0%
mmu-mir-210-5p	7	148407306	148407327	-	edit	15	0	0.0%	0	0.0%	0	0.0%
mmu-mir-210-5p	7	148407306	148407327	-	edit	16	0	0.0%	0	0.0%	0	0.0%
mmu-mir-210-5p	7	148407306	148407327	-	edit	17	0	0.0%	0	0.0%	0	0.0%
mmu-mir-210-5p	7	148407306	148407327	-	edit	18	0	0.0%	0	0.0%	0	0.0%
mmu-mir-210-5p	7	148407306	148407327	-	edit	19	12	1.4%	11	2.7%	7	4.0%
mmu-mir-210-5p	7	148407306	148407327	-	edit	20	0	0.0%	0	0.0%	0	0.0%
mmu-mir-210-5p	7	148407306	148407327	-	edit	21	0	0.0%	0	0.0%	0	0.0%
mmu-mir-210-5p	7	148407306	148407327	-	edit	22	0	0.0%	0	0.0%	0	0.0%
EDITED							12	1.4%	11	2.7%	7	4.0%
CANONICAL							874	98.6%	397	97.3%	158	96.0%
TOTAL							886		408		165	

miRNA	Chr	Start	Stop	Strand	Type	Position	P7 reads	P7%	P10 reads	P10%	P14 reads	P14%
mmu-mir-221-5p	X	18723433	18723455	-	edit	2	0	0.0%	0	0.0%	0	0.0%
mmu-mir-221-5p	X	18723433	18723455	-	edit	3	0	0.0%	0	0.0%	0	0.0%
mmu-mir-221-5p	X	18723433	18723455	-	edit	4	0	0.0%	0	0.0%	0	0.0%
mmu-mir-221-5p	X	18723433	18723455	-	edit	5	0	0.0%	0	0.0%	0	0.0%
mmu-mir-221-5p	X	18723433	18723455	-	edit	6	0	0.0%	0	0.0%	0	0.0%
mmu-mir-221-5p	X	18723433	18723455	-	edit	7	0	0.0%	0	0.0%	0	0.0%
mmu-mir-221-5p	X	18723433	18723455	-	edit	8	0	0.0%	0	0.0%	0	0.0%
mmu-mir-221-5p	X	18723433	18723455	-	edit	9	0	0.0%	0	0.0%	0	0.0%
mmu-mir-221-5p	X	18723433	18723455	-	edit	10	0	0.0%	0	0.0%	0	0.0%
mmu-mir-221-5p	X	18723433	18723455	-	edit	11	0	0.0%	0	0.0%	0	0.0%
mmu-mir-221-5p	X	18723433	18723455	-	edit	12	0	0.0%	0	0.0%	0	0.0%
mmu-mir-221-5p	X	18723433	18723455	-	edit	13	0	0.0%	0	0.0%	0	0.0%
mmu-mir-221-5p	X	18723433	18723455	-	edit	14	0	0.0%	0	0.0%	0	0.0%
mmu-mir-221-5p	X	18723433	18723455	-	edit	15	9	0.4%	3	0.6%	5	1.1%
mmu-mir-221-5p	X	18723433	18723455	-	edit	16	3	0.1%	0	0.0%	0	0.0%
mmu-mir-221-5p	X	18723433	18723455	-	edit	17	0	0.0%	0	0.0%	0	0.0%
mmu-mir-221-5p	X	18723433	18723455	-	edit	18	0	0.0%	0	0.0%	0	0.0%
mmu-mir-221-5p	X	18723433	18723455	-	edit	19	5	0.2%	0	0.0%	0	0.0%
mmu-mir-221-5p	X	18723433	18723455	-	edit	20	25	1.2%	3	0.5%	0	0.0%
mmu-mir-221-5p	X	18723433	18723455	-	edit	21	0	0.0%	0	0.0%	0	0.0%
mmu-mir-221-5p	X	18723433	18723455	-	edit	22	0	0.0%	0	0.0%	0	0.0%
EDITED							42	2.1%	6	1.1%	5	1.1%
CANONICAL							1998	97.9%	552	98.9%	461	98.9%
TOTAL							2040		558		466	

miRNA	Chr	Start	Stop	Strand	Type	Position	P7 reads	P7%	P10 reads	P10%	P14 reads	P14%
mmu-mir-222-5p	X	18724029	18724049	-	edit	1	0	0.0%	0	0.0%	0	0.0%
mmu-mir-222-5p	X	18724029	18724049	-	edit	2	0	0.0%	0	0.0%	0	0.0%
mmu-mir-222-5p	X	18724029	18724049	-	edit	3	0	0.0%	0	0.0%	0	0.0%
mmu-mir-222-5p	X	18724029	18724049	-	edit	4	0	0.0%	0	0.0%	0	0.0%
mmu-mir-222-5p	X	18724029	18724049	-	edit	5	0	0.0%	0	0.0%	0	0.0%
mmu-mir-222-5p	X	18724029	18724049	-	edit	6	0	0.0%	0	0.0%	0	0.0%
mmu-mir-222-5p	X	18724029	18724049	-	edit	7	0	0.0%	0	0.0%	0	0.0%
mmu-mir-222-5p	X	18724029	18724049	-	edit	8	0	0.0%	0	0.0%	0	0.0%
mmu-mir-222-5p	X	18724029	18724049	-	edit	9	0	0.0%	0	0.0%	0	0.0%
mmu-mir-222-5p	X	18724029	18724049	-	edit	10	0	0.0%	0	0.0%	0	0.0%
mmu-mir-222-5p	X	18724029	18724049	-	edit	11	0	0.0%	0	0.0%	0	0.0%
mmu-mir-222-5p	X	18724029	18724049	-	edit	12	0	0.0%	0	0.0%	0	0.0%
mmu-mir-222-5p	X	18724029	18724049	-	edit	13	0	0.0%	0	0.0%	0	0.0%
mmu-mir-222-5p	X	18724029	18724049	-	edit	14	0	0.0%	0	0.0%	0	0.0%
mmu-mir-222-5p	X	18724029	18724049	-	edit	15	0	0.0%	0	0.0%	0	0.0%
mmu-mir-222-5p	X	18724029	18724049	-	edit	16	0	0.0%	0	0.0%	0	0.0%
mmu-mir-222-5p	X	18724029	18724049	-	edit	17	0	0.0%	0	0.0%	0	0.0%
mmu-mir-222-5p	X	18724029	18724049	-	edit	18	0	0.0%	0	0.0%	0	0.0%
mmu-mir-222-5p	X	18724029	18724049	-	edit	19	0	0.0%	0	0.0%	0	0.0%
mmu-mir-222-5p	X	18724029	18724049	-	edit	20	0	0.0%	0	0.0%	0	0.0%
mmu-mir-222-5p	X	18724029	18724049	-	edit	21	7	1.9%	0	0.0%	0	0.0%
mmu-mir-222-5p	X	18724029	18724049	-	edit	22	0	0.0%	0	0.0%	0	0.0%
EDITED							7	1.9%	0	0.0%	0	0.0%
CANONICAL							364	98.1%	125	100.0%	70	100.0%
TOTAL							371		125		70	

miRNA	Chr	Start	Stop	Strand	Type	Position	P7 reads	P7%	P10 reads	P10%	P14 reads	P14%
mmu-mir-296-5p	2	174092594	174092614	-	edit	1	0	0.0%	0	0.0%	0	0.0%
mmu-mir-296-5p	2	174092594	174092614	-	edit	2	0	0.0%	0	0.0%	0	0.0%
mmu-mir-296-5p	2	174092594	174092614	-	edit	3	0	0.0%	0	0.0%	0	0.0%
mmu-mir-296-5p	2	174092594	174092614	-	edit	4	0	0.0%	0	0.0%	0	0.0%
mmu-mir-296-5p	2	174092594	174092614	-	edit	5	0	0.0%	0	0.0%	0	0.0%
mmu-mir-296-5p	2	174092594	174092614	-	edit	6	0	0.0%	0	0.0%	0	0.0%
mmu-mir-296-5p	2	174092594	174092614	-	edit	7	0	0.0%	0	0.0%	0	0.0%
mmu-mir-296-5p	2	174092594	174092614	-	edit	8	0	0.0%	0	0.0%	0	0.0%
mmu-mir-296-5p	2	174092594	174092614	-	edit	9	0	0.0%	0	0.0%	0	0.0%
mmu-mir-296-5p	2	174092594	174092614	-	edit	10	0	0.0%	0	0.0%	0	0.0%
mmu-mir-296-5p	2	174092594	174092614	-	edit	11	0	0.0%	0	0.0%	0	0.0%
mmu-mir-296-5p	2	174092594	174092614	-	edit	12	0	0.0%	0	0.0%	0	0.0%
mmu-mir-296-5p	2	174092594	174092614	-	edit	13	0	0.0%	0	0.0%	0	0.0%
mmu-mir-296-5p	2	174092594	174092614	-	edit	14	0	0.0%	0	0.0%	0	0.0%
mmu-mir-296-5p	2	174092594	174092614	-	edit	15	0	0.0%	0	0.0%	0	0.0%
mmu-mir-296-5p	2	174092594	174092614	-	edit	16	0	0.0%	0	0.0%	0	0.0%
mmu-mir-296-5p	2	174092594	174092614	-	edit	17	0	0.0%	0	0.0%	0	0.0%
mmu-mir-296-5p	2	174092594	174092614	-	edit	18	0	0.0%	0	0.0%	0	0.0%
mmu-mir-296-5p	2	174092594	174092614	-	edit	19	4	0.9%	0	0.0%	0	0.0%
mmu-mir-296-5p	2	174092594	174092614	-	edit	20	0	0.0%	0	0.0%	0	0.0%
mmu-mir-296-5p	2	174092594	174092614	-	edit	21	0	0.0%	0	0.0%	0	0.0%
mmu-mir-296-5p	2	174092594	174092614	-	edit	22	0	0.0%	0	0.0%	0	0.0%
EDITED							4	0.9%	0	0.0%	0	0.0%
CANONICAL							381	99.1%	136	100.0%	107	100.0%
TOTAL							385		136		107	

miRNA	Chr	Start	Stop	Strand	Type	Position	P7 reads	P7%	P10 reads	P10%	P14 reads	P14%
mmu-mir-298-5p	2	174093054	174093076	-	Indel	22	0	0.0%	4	0.1%	0	0.0%
mmu-mir-298-5p	2	174093054	174093076	-	edit	1	0	0.0%	0	0.0%	0	0.0%
mmu-mir-298-5p	2	174093054	174093076	-	edit	2	0	0.0%	0	0.0%	0	0.0%
mmu-mir-298-5p	2	174093054	174093076	-	edit	3	0	0.0%	0	0.0%	0	0.0%
mmu-mir-298-5p	2	174093054	174093076	-	edit	4	0	0.0%	0	0.0%	0	0.0%
mmu-mir-298-5p	2	174093054	174093076	-	edit	5	0	0.0%	0	0.0%	0	0.0%
mmu-mir-298-5p	2	174093054	174093076	-	edit	6	0	0.0%	0	0.0%	0	0.0%
mmu-mir-298-5p	2	174093054	174093076	-	edit	7	0	0.0%	0	0.0%	0	0.0%
mmu-mir-298-5p	2	174093054	174093076	-	edit	8	0	0.0%	0	0.0%	0	0.0%
mmu-mir-298-5p	2	174093054	174093076	-	edit	9	0	0.0%	0	0.0%	0	0.0%
mmu-mir-298-5p	2	174093054	174093076	-	edit	10	0	0.0%	0	0.0%	0	0.0%
mmu-mir-298-5p	2	174093054	174093076	-	edit	11	0	0.0%	0	0.0%	0	0.0%
mmu-mir-298-5p	2	174093054	174093076	-	edit	12	0	0.0%	0	0.0%	0	0.0%
mmu-mir-298-5p	2	174093054	174093076	-	edit	13	6	0.1%	12	0.2%	18	0.4%
mmu-mir-298-5p	2	174093054	174093076	-	edit	14	0	0.0%	0	0.0%	0	0.0%
mmu-mir-298-5p	2	174093054	174093076	-	edit	15	0	0.0%	0	0.0%	4	0.1%
mmu-mir-298-5p	2	174093054	174093076	-	edit	16	28	0.2%	11	0.2%	15	0.3%
mmu-mir-298-5p	2	174093054	174093076	-	edit	17	6	0.1%	0	0.0%	0	0.0%
mmu-mir-298-5p	2	174093054	174093076	-	edit	18	9	0.1%	0	0.0%	5	0.1%
mmu-mir-298-5p	2	174093054	174093076	-	edit	19	7	0.1%	7	0.1%	6	0.1%
mmu-mir-298-5p	2	174093054	174093076	-	edit	20	4	0.0%	0	0.0%	0	0.0%
mmu-mir-298-5p	2	174093054	174093076	-	edit	21	16	0.1%	6	0.1%	12	0.2%
mmu-mir-298-5p	2	174093054	174093076	-	edit	22	23	0.2%	11	0.2%	20	0.4%
EDITED							98	0.8%	50	0.9%	79	1.7%
CANONICAL							11498	99.2%	5248	99.1%	4534	98.3%
TOTAL							11596		5298		4613	

miRNA	Chr	Start	Stop	Strand	Type	Position	P7 reads	P7%	P10 reads	P10%	P14 reads	P14%
mmu-mir-320-5p	14	70843364	70843385	+	3' Edit	3'	35	0.1%	0	0.0%	10	0.1%
mmu-mir-320-5p	14	70843364	70843385	+	3' Indel	3'	16	0.0%	0	0.0%	5	0.0%
mmu-mir-320-5p	14	70843364	70843385	+	Indel	22	12	0.0%	0	0.0%	6	0.1%
mmu-mir-320-5p	14	70843364	70843385	+	edit	1	0	0.0%	0	0.0%	0	0.0%
mmu-mir-320-5p	14	70843364	70843385	+	edit	2	0	0.0%	0	0.0%	0	0.0%
mmu-mir-320-5p	14	70843364	70843385	+	edit	3	0	0.0%	0	0.0%	0	0.0%
mmu-mir-320-5p	14	70843364	70843385	+	edit	4	15	0.0%	12	0.1%	0	0.0%
mmu-mir-320-5p	14	70843364	70843385	+	edit	5	0	0.0%	0	0.0%	0	0.0%
mmu-mir-320-5p	14	70843364	70843385	+	edit	6	46	0.1%	14	0.1%	16	0.2%
mmu-mir-320-5p	14	70843364	70843385	+	edit	7	0	0.0%	0	0.0%	0	0.0%
mmu-mir-320-5p	14	70843364	70843385	+	edit	8	38	0.1%	15	0.1%	0	0.0%
mmu-mir-320-5p	14	70843364	70843385	+	edit	9	0	0.0%	0	0.0%	0	0.0%
mmu-mir-320-5p	14	70843364	70843385	+	edit	10	0	0.0%	0	0.0%	0	0.0%
mmu-mir-320-5p	14	70843364	70843385	+	edit	11	0	0.0%	0	0.0%	0	0.0%
mmu-mir-320-5p	14	70843364	70843385	+	edit	12	0	0.0%	0	0.0%	0	0.0%
mmu-mir-320-5p	14	70843364	70843385	+	edit	13	0	0.0%	0	0.0%	0	0.0%
mmu-mir-320-5p	14	70843364	70843385	+	edit	14	3	0.0%	0	0.0%	0	0.0%
mmu-mir-320-5p	14	70843364	70843385	+	edit	15	129	0.3%	49	0.2%	26	0.2%
mmu-mir-320-5p	14	70843364	70843385	+	edit	16	4	0.0%	9	0.0%	0	0.0%
mmu-mir-320-5p	14	70843364	70843385	+	edit	17	8	0.0%	3	0.0%	0	0.0%
mmu-mir-320-5p	14	70843364	70843385	+	edit	18	5	0.0%	0	0.0%	0	0.0%
mmu-mir-320-5p	14	70843364	70843385	+	edit	19	306	0.6%	65	0.3%	67	0.6%
mmu-mir-320-5p	14	70843364	70843385	+	edit	20	94	0.2%	40	0.2%	34	0.3%
mmu-mir-320-5p	14	70843364	70843385	+	edit	21	23	0.0%	4	0.0%	5	0.0%
mmu-mir-320-5p	14	70843364	70843385	+	edit	22	30	0.1%	7	0.0%	11	0.1%
EDITED							762	1.5%	218	1.0%	179	1.7%
CANONICAL							48550	98.5%	20699	99.0%	10643	98.3%
TOTAL							49312		20917		10822	

miRNA	Chr	Start	Stop	Strand	Type	Position	P7 reads	P7%	P10 reads	P10%	P14 reads	P14%
mmu-mir-322-5p	X	50407446	50407466	-	5' Edit	5'	19	2.2%	0	0.0%	0	0.0%
mmu-mir-322-5p	X	50407483	50407504	-	edit	1	0	0.0%	0	0.0%	0	0.0%
mmu-mir-322-5p	X	50407483	50407504	-	edit	2	0	0.0%	0	0.0%	0	0.0%
mmu-mir-322-5p	X	50407483	50407504	-	edit	3	0	0.0%	0	0.0%	0	0.0%
mmu-mir-322-5p	X	50407483	50407504	-	edit	4	0	0.0%	0	0.0%	0	0.0%
mmu-mir-322-5p	X	50407483	50407504	-	edit	5	0	0.0%	0	0.0%	0	0.0%
mmu-mir-322-5p	X	50407483	50407504	-	edit	6	21	2.4%	0	0.0%	6	2.3%
mmu-mir-322-5p	X	50407483	50407504	-	edit	7	3	0.4%	0	0.0%	0	0.0%
mmu-mir-322-5p	X	50407483	50407504	-	edit	8	0	0.0%	0	0.0%	0	0.0%
mmu-mir-322-5p	X	50407483	50407504	-	edit	9	0	0.0%	0	0.0%	0	0.0%
mmu-mir-322-5p	X	50407483	50407504	-	edit	10	0	0.0%	0	0.0%	0	0.0%
mmu-mir-322-5p	X	50407483	50407504	-	edit	11	0	0.0%	0	0.0%	0	0.0%
mmu-mir-322-5p	X	50407483	50407504	-	edit	12	0	0.0%	0	0.0%	0	0.0%
mmu-mir-322-5p	X	50407483	50407504	-	edit	13	20	2.3%	6	1.3%	5	2.0%
mmu-mir-322-5p	X	50407483	50407504	-	edit	14	19	2.2%	3	0.7%	5	2.0%
mmu-mir-322-5p	X	50407483	50407504	-	edit	15	21	2.4%	3	0.7%	0	0.0%
mmu-mir-322-5p	X	50407483	50407504	-	edit	16	34	4.0%	10	2.3%	9	3.5%
mmu-mir-322-5p	X	50407483	50407504	-	edit	17	**32**	**3.7%**	**36**	**8.3%**	**8**	**3.1%**
mmu-mir-322-5p	X	50407483	50407504	-	edit	18	4	0.5%	0	0.0%	0	0.0%
mmu-mir-322-5p	X	50407483	50407504	-	edit	19	0	0.0%	0	0.0%	0	0.0%
mmu-mir-322-5p	X	50407483	50407504	-	edit	20	0	0.0%	0	0.0%	0	0.0%
mmu-mir-322-5p	X	50407483	50407504	-	edit	21	5	0.6%	0	0.0%	0	0.0%
mmu-mir-322-5p	X	50407483	50407504	-	edit	22	0	0.0%	0	0.0%	0	0.0%
EDITED							158	18.4%	58	13.2%	33	12.9%
CANONICAL							699	81.6%	378	86.8%	223	87.1%
TOTAL							857		436		256	

miRNA	Chr	Start	Stop	Strand	Type	Position	P7 reads	P7%	P10 reads	P10%	P14 reads	P14%
mmu-mir-323-3p	12	110950768	110950788	+	edit	1	0	0.0%	0	0.0%	0	0.0%
mmu-mir-323-3p	12	110950768	110950788	+	edit	2	0	0.0%	0	0.0%	0	0.0%
mmu-mir-323-3p	12	110950768	110950788	+	edit	3	0	0.0%	0	0.0%	0	0.0%
mmu-mir-323-3p	12	110950768	110950788	+	edit	4	0	0.0%	0	0.0%	0	0.0%
mmu-mir-323-3p	12	110950768	110950788	+	edit	5	0	0.0%	0	0.0%	0	0.0%
mmu-mir-323-3p	12	110950768	110950788	+	edit	6	0	0.0%	0	0.0%	0	0.0%
mmu-mir-323-3p	12	110950768	110950788	+	edit	7	0	0.0%	0	0.0%	0	0.0%
mmu-mir-323-3p	12	110950768	110950788	+	edit	8	0	0.0%	0	0.0%	0	0.0%
mmu-mir-323-3p	12	110950768	110950788	+	edit	9	0	0.0%	0	0.0%	0	0.0%
mmu-mir-323-3p	12	110950768	110950788	+	edit	10	0	0.0%	0	0.0%	0	0.0%
mmu-mir-323-3p	12	110950768	110950788	+	edit	11	0	0.0%	0	0.0%	0	0.0%
mmu-mir-323-3p	12	110950768	110950788	+	edit	12	0	0.0%	0	0.0%	0	0.0%
mmu-mir-323-3p	12	110950768	110950788	+	edit	13	0	0.0%	0	0.0%	0	0.0%
mmu-mir-323-3p	12	110950768	110950788	+	edit	14	4	0.9%	0	0.0%	0	0.0%
mmu-mir-323-3p	12	110950768	110950788	+	edit	15	0	0.0%	0	0.0%	0	0.0%
mmu-mir-323-3p	12	110950768	110950788	+	edit	16	0	0.0%	0	0.0%	0	0.0%
mmu-mir-323-3p	12	110950768	110950788	+	edit	17	0	0.0%	0	0.0%	0	0.0%
mmu-mir-323-3p	12	110950768	110950788	+	edit	18	6	1.4%	0	0.0%	0	0.0%
mmu-mir-323-3p	12	110950768	110950788	+	edit	19	0	0.0%	0	0.0%	0	0.0%
mmu-mir-323-3p	12	110950768	110950788	+	edit	20	0	0.0%	0	0.0%	0	0.0%
mmu-mir-323-3p	12	110950768	110950788	+	edit	21	0	0.0%	0	0.0%	0	0.0%
mmu-mir-323-3p	12	110950768	110950788	+	edit	22	0	0.0%	0	0.0%	0	0.0%
EDITED							10	2.3%	0	0.0%	0	0.0%
CANONICAL							422	97.7%	99	100.0%	37	100.0%
TOTAL							432		99		37	

miRNA	Chr	Start	Stop	Strand	Type	Position	P7 reads	P7%	P10 reads	P10%	P14 reads	P14%
mmu-mir-328-5p	8	107832279	107832300	-	edit	1	0	0.0%	0	0.0%	0	0.0%
mmu-mir-328-5p	8	107832279	107832300	-	edit	2	0	0.0%	0	0.0%	0	0.0%
mmu-mir-328-5p	8	107832279	107832300	-	edit	3	0	0.0%	0	0.0%	0	0.0%
mmu-mir-328-5p	8	107832279	107832300	-	edit	4	0	0.0%	0	0.0%	0	0.0%
mmu-mir-328-5p	8	107832279	107832300	-	edit	5	0	0.0%	0	0.0%	0	0.0%
mmu-mir-328-5p	8	107832279	107832300	-	edit	6	0	0.0%	0	0.0%	0	0.0%
mmu-mir-328-5p	8	107832279	107832300	-	edit	7	0	0.0%	0	0.0%	0	0.0%
mmu-mir-328-5p	8	107832279	107832300	-	edit	8	0	0.0%	0	0.0%	0	0.0%
mmu-mir-328-5p	8	107832279	107832300	-	edit	9	0	0.0%	0	0.0%	0	0.0%
mmu-mir-328-5p	8	107832279	107832300	-	edit	10	0	0.0%	0	0.0%	0	0.0%
mmu-mir-328-5p	8	107832279	107832300	-	edit	11	0	0.0%	0	0.0%	0	0.0%
mmu-mir-328-5p	8	107832279	107832300	-	edit	12	0	0.0%	0	0.0%	0	0.0%
mmu-mir-328-5p	8	107832279	107832300	-	edit	13	0	0.0%	0	0.0%	0	0.0%
mmu-mir-328-5p	8	107832279	107832300	-	edit	14	0	0.0%	0	0.0%	0	0.0%
mmu-mir-328-5p	8	107832279	107832300	-	edit	15	0	0.0%	3	3.3%	0	0.0%
mmu-mir-328-5p	8	107832279	107832300	-	edit	16	0	0.0%	0	0.0%	0	0.0%
mmu-mir-328-5p	8	107832279	107832300	-	edit	17	0	0.0%	0	0.0%	0	0.0%
mmu-mir-328-5p	8	107832279	107832300	-	edit	18	0	0.0%	0	0.0%	0	0.0%
mmu-mir-328-5p	8	107832279	107832300	-	edit	19	0	0.0%	0	0.0%	0	0.0%
mmu-mir-328-5p	8	107832279	107832300	-	edit	20	0	0.0%	0	0.0%	0	0.0%
mmu-mir-328-5p	8	107832279	107832300	-	edit	21	0	0.0%	0	0.0%	0	0.0%
mmu-mir-328-5p	8	107832279	107832300	-	edit	22	0	0.0%	0	0.0%	0	0.0%
EDITED							0	0.0%	3	3.3%	0	0.0%
CANONICAL							116	100.0%	89	96.7%	53	100.0%
TOTAL							116		92		53	

miRNA	Chr	Start	Stop	Strand	Type	Position	P7 reads	P7%	P10 reads	P10%	P14 reads	P14%
mmu-mir-330-3p	7	19766874	19766896	+	edit	1	0	0.0%	0	0.0%	0	0.0%
mmu-mir-330-3p	7	19766874	19766896	+	edit	2	0	0.0%	0	0.0%	0	0.0%
mmu-mir-330-3p	7	19766874	19766896	+	edit	3	0	0.0%	0	0.0%	0	0.0%
mmu-mir-330-3p	7	19766874	19766896	+	edit	4	0	0.0%	0	0.0%	0	0.0%
mmu-mir-330-3p	7	19766874	19766896	+	edit	5	0	0.0%	0	0.0%	0	0.0%
mmu-mir-330-3p	7	19766874	19766896	+	edit	6	0	0.0%	0	0.0%	0	0.0%
mmu-mir-330-3p	7	19766874	19766896	+	edit	7	0	0.0%	0	0.0%	0	0.0%
mmu-mir-330-3p	7	19766874	19766896	+	edit	8	0	0.0%	0	0.0%	0	0.0%
mmu-mir-330-3p	7	19766874	19766896	+	edit	9	0	0.0%	0	0.0%	0	0.0%
mmu-mir-330-3p	7	19766874	19766896	+	edit	10	0	0.0%	0	0.0%	0	0.0%
mmu-mir-330-3p	7	19766874	19766896	+	edit	11	0	0.0%	0	0.0%	0	0.0%
mmu-mir-330-3p	7	19766874	19766896	+	edit	12	0	0.0%	0	0.0%	0	0.0%
mmu-mir-330-3p	7	19766874	19766896	+	edit	13	0	0.0%	0	0.0%	0	0.0%
mmu-mir-330-3p	7	19766874	19766896	+	edit	14	0	0.0%	0	0.0%	0	0.0%
mmu-mir-330-3p	7	19766874	19766896	+	edit	15	0	0.0%	0	0.0%	0	0.0%
mmu-mir-330-3p	7	19766874	19766896	+	edit	16	0	0.0%	0	0.0%	0	0.0%
mmu-mir-330-3p	7	19766874	19766896	+	edit	17	0	0.0%	0	0.0%	0	0.0%
mmu-mir-330-3p	7	19766874	19766896	+	edit	18	0	0.0%	0	0.0%	0	0.0%
mmu-mir-330-3p	7	19766874	19766896	+	edit	19	0	0.0%	3	2.4%	0	0.0%
mmu-mir-330-3p	7	19766874	19766896	+	edit	20	0	0.0%	0	0.0%	0	0.0%
mmu-mir-330-3p	7	19766874	19766896	+	edit	21	0	0.0%	0	0.0%	0	0.0%
mmu-mir-330-3p	7	19766874	19766896	+	edit	22	0	0.0%	0	0.0%	0	0.0%
EDITED							0	0.0%	3	2.4%	0	0.0%
CANONICAL							216	100.0%	124	97.6%	53	100.0%
TOTAL							216		127		53	

miRNA	Chr	Start	Stop	Strand	Type	Position	P7 reads	P7%	P10 reads	P10%	P14 reads	P14%
mmu-mir-337-5p	12	110824025	110824045	+	edit	1	0	0.0%	0	0.0%	0	0.0%
mmu-mir-337-5p	12	110824025	110824045	+	edit	2	0	0.0%	0	0.0%	0	0.0%
mmu-mir-337-5p	12	110824025	110824045	+	edit	3	0	0.0%	0	0.0%	0	0.0%
mmu-mir-337-5p	12	110824025	110824045	+	edit	4	0	0.0%	0	0.0%	0	0.0%
mmu-mir-337-5p	12	110824025	110824045	+	edit	5	0	0.0%	0	0.0%	0	0.0%
mmu-mir-337-5p	12	110824025	110824045	+	edit	6	0	0.0%	0	0.0%	0	0.0%
mmu-mir-337-5p	12	110824025	110824045	+	edit	7	0	0.0%	0	0.0%	0	0.0%
mmu-mir-337-5p	12	110824025	110824045	+	edit	8	0	0.0%	0	0.0%	0	0.0%
mmu-mir-337-5p	12	110824025	110824045	+	edit	9	0	0.0%	0	0.0%	0	0.0%
mmu-mir-337-5p	12	110824025	110824045	+	edit	10	0	0.0%	0	0.0%	0	0.0%
mmu-mir-337-5p	12	110824025	110824045	+	edit	11	0	0.0%	0	0.0%	0	0.0%
mmu-mir-337-5p	12	110824025	110824045	+	edit	12	0	0.0%	0	0.0%	0	0.0%
mmu-mir-337-5p	12	110824025	110824045	+	edit	13	0	0.0%	0	0.0%	0	0.0%
mmu-mir-337-5p	12	110824025	110824045	+	edit	14	0	0.0%	0	0.0%	0	0.0%
mmu-mir-337-5p	12	110824025	110824045	+	edit	15	0	0.0%	0	0.0%	0	0.0%
mmu-mir-337-5p	12	110824025	110824045	+	edit	16	0	0.0%	0	0.0%	0	0.0%
mmu-mir-337-5p	12	110824025	110824045	+	edit	17	0	0.0%	0	0.0%	0	0.0%
mmu-mir-337-5p	12	110824025	110824045	+	edit	18	**3**	**0.7%**	**9**	**5.3%**	0	0.0%
mmu-mir-337-5p	12	110824025	110824045	+	edit	19	0	0.0%	0	0.0%	0	0.0%
mmu-mir-337-5p	12	110824025	110824045	+	edit	20	0	0.0%	0	0.0%	0	0.0%
mmu-mir-337-5p	12	110824025	110824045	+	edit	21	0	0.0%	0	0.0%	0	0.0%
mmu-mir-337-5p	12	110824025	110824045	+	edit	22	0	0.0%	0	0.0%	0	0.0%
EDITED							3	0.7%	9	5.3%	0	0.0%
CANONICAL							403	99.3%	151	94.7%	47	100.0%
TOTAL							406		160		47	

miRNA	Chr	Start	Stop	Strand	Type	Position	P7 reads	P7%	P10 reads	P10%	P14 reads	P14%
mmu-mir-340-5p	11	49883222	49883243	+	3' Edit	3'	0	0.0%	0	0.0%	3	0.1%
mmu-mir-340-5p	11	49883222	49883243	+	Indel	22	0	0.0%	0	0.0%	3	0.1%
mmu-mir-340-5p	11	49883222	49883243	+	edit	1	0	0.0%	0	0.0%	0	0.0%
mmu-mir-340-5p	11	49883222	49883243	+	edit	2	0	0.0%	0	0.0%	0	0.0%
mmu-mir-340-5p	11	49883222	49883243	+	edit	3	0	0.0%	0	0.0%	0	0.0%
mmu-mir-340-5p	11	49883222	49883243	+	edit	4	0	0.0%	0	0.0%	0	0.0%
mmu-mir-340-5p	11	49883222	49883243	+	edit	5	0	0.0%	0	0.0%	0	0.0%
mmu-mir-340-5p	11	49883222	49883243	+	edit	6	0	0.0%	0	0.0%	0	0.0%
mmu-mir-340-5p	11	49883222	49883243	+	edit	7	0	0.0%	0	0.0%	0	0.0%
mmu-mir-340-5p	11	49883222	49883243	+	edit	8	7	0.1%	0	0.0%	0	0.0%
mmu-mir-340-5p	11	49883222	49883243	+	edit	9	0	0.0%	0	0.0%	0	0.0%
mmu-mir-340-5p	11	49883222	49883243	+	edit	10	0	0.0%	0	0.0%	0	0.0%
mmu-mir-340-5p	11	49883222	49883243	+	edit	11	0	0.0%	0	0.0%	0	0.0%
mmu-mir-340-5p	11	49883222	49883243	+	edit	12	0	0.0%	0	0.0%	0	0.0%
mmu-mir-340-5p	11	49883222	49883243	+	edit	13	0	0.0%	0	0.0%	0	0.0%
mmu-mir-340-5p	11	49883222	49883243	+	edit	14	0	0.0%	0	0.0%	0	0.0%
mmu-mir-340-5p	11	49883222	49883243	+	edit	15	0	0.0%	0	0.0%	3	0.1%
mmu-mir-340-5p	11	49883222	49883243	+	edit	16	0	0.0%	0	0.0%	0	0.0%
mmu-mir-340-5p	11	49883222	49883243	+	edit	17	0	0.0%	0	0.0%	0	0.0%
mmu-mir-340-5p	11	49883222	49883243	+	edit	18	0	0.0%	0	0.0%	0	0.0%
mmu-mir-340-5p	11	49883222	49883243	+	edit	19	3	0.0%	0	0.0%	0	0.0%
mmu-mir-340-5p	11	49883222	49883243	+	edit	20	0	0.0%	0	0.0%	0	0.0%
mmu-mir-340-5p	11	49883222	49883243	+	edit	21	0	0.0%	0	0.0%	0	0.0%
mmu-mir-340-5p	11	49883222	49883243	+	edit	22	0	0.0%	5	0.1%	0	0.0%
EDITED							10	0.2%	5	0.1%	9	0.2%
CANONICAL							6713	99.8%	4471	99.9%	3665	99.8%
TOTAL							6723		4476		3674	

miRNA	Chr	Start	Stop	Strand	Type	Position	P7 reads	P7%	P10 reads	P10%	P14 reads	P14%
mmu-mir-341-5p	12	110849766	110849786	+	3' Edit	3'	3	0.1%	0	0.0%	0	0.0%
mmu-mir-341-5p	12	110849766	110849786	+	edit	1	0	0.0%	0	0.0%	0	0.0%
mmu-mir-341-5p	12	110849766	110849786	+	edit	2	0	0.0%	0	0.0%	0	0.0%
mmu-mir-341-5p	12	110849766	110849786	+	edit	3	0	0.0%	0	0.0%	0	0.0%
mmu-mir-341-5p	12	110849766	110849786	+	edit	4	0	0.0%	0	0.0%	0	0.0%
mmu-mir-341-5p	12	110849766	110849786	+	edit	5	0	0.0%	0	0.0%	0	0.0%
mmu-mir-341-5p	12	110849766	110849786	+	edit	6	0	0.0%	0	0.0%	0	0.0%
mmu-mir-341-5p	12	110849766	110849786	+	edit	7	0	0.0%	0	0.0%	0	0.0%
mmu-mir-341-5p	12	110849766	110849786	+	edit	8	0	0.0%	0	0.0%	0	0.0%
mmu-mir-341-5p	12	110849766	110849786	+	edit	9	0	0.0%	0	0.0%	0	0.0%
mmu-mir-341-5p	12	110849766	110849786	+	edit	10	0	0.0%	0	0.0%	0	0.0%
mmu-mir-341-5p	12	110849766	110849786	+	edit	11	0	0.0%	0	0.0%	0	0.0%
mmu-mir-341-5p	12	110849766	110849786	+	edit	12	0	0.0%	0	0.0%	0	0.0%
mmu-mir-341-5p	12	110849766	110849786	+	edit	13	0	0.0%	0	0.0%	0	0.0%
mmu-mir-341-5p	12	110849766	110849786	+	edit	14	0	0.0%	0	0.0%	0	0.0%
mmu-mir-341-5p	12	110849766	110849786	+	edit	15	0	0.0%	0	0.0%	0	0.0%
mmu-mir-341-5p	12	110849766	110849786	+	edit	16	0	0.0%	0	0.0%	0	0.0%
mmu-mir-341-5p	12	110849766	110849786	+	edit	17	0	0.0%	0	0.0%	0	0.0%
mmu-mir-341-5p	12	110849766	110849786	+	edit	18	0	0.0%	0	0.0%	0	0.0%
mmu-mir-341-5p	12	110849766	110849786	+	edit	19	0	0.0%	0	0.0%	0	0.0%
mmu-mir-341-5p	12	110849766	110849786	+	edit	20	0	0.0%	0	0.0%	0	0.0%
mmu-mir-341-5p	12	110849766	110849786	+	edit	21	0	0.0%	0	0.0%	0	0.0%
mmu-mir-341-5p	12	110849766	110849786	+	edit	22	0	0.0%	0	0.0%	0	0.0%
EDITED							3	0.1%	0	0.0%	0	0.0%
CANONICAL							2419	99.9%	931	100.0%	211	100.0%
TOTAL							2422		931		211	

miRNA	Chr	Start	Stop	Strand	Type	Position	P7 reads	P7%	P10 reads	P10%	P14 reads	P14%
mmu-mir-342-3p	12	109896890	109896912	+	edit	1	0	0.0%	0	0.0%	0	0.0%
mmu-mir-342-3p	12	109896890	109896912	+	edit	2	0	0.0%	0	0.0%	0	0.0%
mmu-mir-342-3p	12	109896890	109896912	+	edit	3	0	0.0%	0	0.0%	0	0.0%
mmu-mir-342-3p	12	109896890	109896912	+	edit	4	0	0.0%	0	0.0%	0	0.0%
mmu-mir-342-3p	12	109896890	109896912	+	edit	5	0	0.0%	0	0.0%	0	0.0%
mmu-mir-342-3p	12	109896890	109896912	+	edit	6	0	0.0%	0	0.0%	0	0.0%
mmu-mir-342-3p	12	109896890	109896912	+	edit	7	0	0.0%	0	0.0%	0	0.0%
mmu-mir-342-3p	12	109896890	109896912	+	edit	8	0	0.0%	0	0.0%	0	0.0%
mmu-mir-342-3p	12	109896890	109896912	+	edit	9	0	0.0%	0	0.0%	0	0.0%
mmu-mir-342-3p	12	109896890	109896912	+	edit	10	0	0.0%	0	0.0%	0	0.0%
mmu-mir-342-3p	12	109896890	109896912	+	edit	11	0	0.0%	0	0.0%	0	0.0%
mmu-mir-342-3p	12	109896890	109896912	+	edit	12	0	0.0%	0	0.0%	0	0.0%
mmu-mir-342-3p	12	109896890	109896912	+	edit	13	0	0.0%	0	0.0%	0	0.0%
mmu-mir-342-3p	12	109896890	109896912	+	edit	14	0	0.0%	0	0.0%	0	0.0%
mmu-mir-342-3p	12	109896890	109896912	+	edit	15	0	0.0%	0	0.0%	0	0.0%
mmu-mir-342-3p	12	109896890	109896912	+	edit	16	0	0.0%	0	0.0%	0	0.0%
mmu-mir-342-3p	12	109896890	109896912	+	edit	17	0	0.0%	0	0.0%	0	0.0%
mmu-mir-342-3p	12	109896890	109896912	+	edit	18	0	0.0%	0	0.0%	0	0.0%
mmu-mir-342-3p	12	109896890	109896912	+	edit	19	9	1.5%	5	0.5%	5	0.8%
mmu-mir-342-3p	12	109896890	109896912	+	edit	20	0	0.0%	0	0.0%	0	0.0%
mmu-mir-342-3p	12	109896890	109896912	+	edit	21	3	0.5%	0	0.0%	0	0.0%
mmu-mir-342-3p	12	109896890	109896912	+	edit	22	0	0.0%	0	0.0%	0	0.0%
EDITED							12	2.0%	5	0.5%	5	0.8%
CANONICAL							591	98.0%	977	99.5%	620	99.2%
TOTAL							603		982		625	

miRNA	Chr	Start	Stop	Strand	Type	Position	P7 reads	P7%	P10 reads	P10%	P14 reads	P14%
mmu-mir-342-5p	12	109896848	109896869	+	3' Edit	3'	0	0.0%	0	0.0%	3	1.7%
mmu-mir-342-5p	12	109896848	109896869	+	edit	1	0	0.0%	0	0.0%	0	0.0%
mmu-mir-342-5p	12	109896848	109896869	+	edit	2	0	0.0%	0	0.0%	0	0.0%
mmu-mir-342-5p	12	109896848	109896869	+	edit	3	0	0.0%	0	0.0%	0	0.0%
mmu-mir-342-5p	12	109896848	109896869	+	edit	4	0	0.0%	0	0.0%	0	0.0%
mmu-mir-342-5p	12	109896848	109896869	+	edit	5	0	0.0%	0	0.0%	0	0.0%
mmu-mir-342-5p	12	109896848	109896869	+	edit	6	3	0.7%	0	0.0%	0	0.0%
mmu-mir-342-5p	12	109896848	109896869	+	edit	7	0	0.0%	0	0.0%	0	0.0%
mmu-mir-342-5p	12	109896848	109896869	+	edit	8	0	0.0%	0	0.0%	0	0.0%
mmu-mir-342-5p	12	109896848	109896869	+	edit	9	0	0.0%	0	0.0%	0	0.0%
mmu-mir-342-5p	12	109896848	109896869	+	edit	10	0	0.0%	0	0.0%	0	0.0%
mmu-mir-342-5p	12	109896848	109896869	+	edit	11	0	0.0%	0	0.0%	0	0.0%
mmu-mir-342-5p	12	109896848	109896869	+	edit	12	0	0.0%	0	0.0%	0	0.0%
mmu-mir-342-5p	12	109896848	109896869	+	edit	13	0	0.0%	0	0.0%	0	0.0%
mmu-mir-342-5p	12	109896848	109896869	+	edit	14	0	0.0%	0	0.0%	0	0.0%
mmu-mir-342-5p	12	109896848	109896869	+	edit	15	4	0.8%	0	0.0%	0	0.0%
mmu-mir-342-5p	12	109896848	109896869	+	edit	16	0	0.0%	0	0.0%	0	0.0%
mmu-mir-342-5p	12	109896848	109896869	+	edit	17	0	0.0%	5	1.4%	0	0.0%
mmu-mir-342-5p	12	109896848	109896869	+	edit	18	0	0.0%	0	0.0%	0	0.0%
mmu-mir-342-5p	12	109896848	109896869	+	edit	19	0	0.0%	0	0.0%	0	0.0%
mmu-mir-342-5p	12	109896848	109896869	+	edit	20	0	0.0%	0	0.0%	0	0.0%
mmu-mir-342-5p	12	109896848	109896869	+	edit	21	0	0.0%	8	2.3%	3	1.7%
mmu-mir-342-5p	12	109896848	109896869	+	edit	22	0	0.0%	0	0.0%	0	0.0%
EDITED							7	1.5%	13	3.7%	6	3.3%
CANONICAL							441	98.5%	342	96.3%	175	96.7%
TOTAL							448		355		181	

miRNA	Chr	Start	Stop	Strand	Type	Position	P7 reads	P7%	P10 reads	P10%	P14 reads	P14%
mmu-mir-345-3p	12	110075237	110075258	+	edit	1	0	0.0%	0	0.0%	0	0.0%
mmu-mir-345-3p	12	110075237	110075258	+	edit	2	0	0.0%	0	0.0%	0	0.0%
mmu-mir-345-3p	12	110075237	110075258	+	edit	3	0	0.0%	0	0.0%	0	0.0%
mmu-mir-345-3p	12	110075237	110075258	+	edit	4	0	0.0%	0	0.0%	0	0.0%
mmu-mir-345-3p	12	110075237	110075258	+	edit	5	0	0.0%	0	0.0%	0	0.0%
mmu-mir-345-3p	12	110075237	110075258	+	edit	6	0	0.0%	0	0.0%	0	0.0%
mmu-mir-345-3p	12	110075237	110075258	+	edit	7	0	0.0%	0	0.0%	0	0.0%
mmu-mir-345-3p	12	110075237	110075258	+	edit	8	0	0.0%	0	0.0%	0	0.0%
mmu-mir-345-3p	12	110075237	110075258	+	edit	9	0	0.0%	0	0.0%	0	0.0%
mmu-mir-345-3p	12	110075237	110075258	+	edit	10	0	0.0%	0	0.0%	0	0.0%
mmu-mir-345-3p	12	110075237	110075258	+	edit	11	0	0.0%	0	0.0%	0	0.0%
mmu-mir-345-3p	12	110075237	110075258	+	edit	12	0	0.0%	0	0.0%	0	0.0%
mmu-mir-345-3p	12	110075237	110075258	+	edit	13	0	0.0%	0	0.0%	0	0.0%
mmu-mir-345-3p	12	110075237	110075258	+	edit	14	6	0.5%	0	0.0%	0	0.0%
mmu-mir-345-3p	12	110075237	110075258	+	edit	15	0	0.0%	0	0.0%	0	0.0%
mmu-mir-345-3p	12	110075237	110075258	+	edit	16	0	0.0%	0	0.0%	0	0.0%
mmu-mir-345-3p	12	110075237	110075258	+	edit	17	0	0.0%	0	0.0%	0	0.0%
mmu-mir-345-3p	12	110075237	110075258	+	edit	18	0	0.0%	0	0.0%	0	0.0%
mmu-mir-345-3p	12	110075237	110075258	+	edit	19	0	0.0%	0	0.0%	0	0.0%
mmu-mir-345-3p	12	110075237	110075258	+	edit	20	0	0.0%	0	0.0%	0	0.0%
mmu-mir-345-3p	12	110075237	110075258	+	edit	21	0	0.0%	0	0.0%	0	0.0%
mmu-mir-345-3p	12	110075237	110075258	+	edit	22	0	0.0%	0	0.0%	0	0.0%
EDITED							6	0.5%	0	0.0%	0	0.0%
CANONICAL							997	99.5%	331	100.0%	242	100.0%
TOTAL							1003		331		242	

miRNA	Chr	Start	Stop	Strand	Type	Position	P7 reads	P7%	P10 reads	P10%	P14 reads	P14%
mmu-mir-351-5p	X	50406492	50406515	-	edit	1	0	0.0%	0	0.0%	0	0.0%
mmu-mir-351-5p	X	50406492	50406515	-	edit	2	0	0.0%	0	0.0%	0	0.0%
mmu-mir-351-5p	X	50406492	50406515	-	edit	3	0	0.0%	0	0.0%	0	0.0%
mmu-mir-351-5p	X	50406492	50406515	-	edit	4	0	0.0%	0	0.0%	0	0.0%
mmu-mir-351-5p	X	50406492	50406515	-	edit	5	0	0.0%	0	0.0%	0	0.0%
mmu-mir-351-5p	X	50406492	50406515	-	edit	6	0	0.0%	0	0.0%	0	0.0%
mmu-mir-351-5p	X	50406492	50406515	-	edit	7	0	0.0%	0	0.0%	0	0.0%
mmu-mir-351-5p	X	50406492	50406515	-	edit	8	0	0.0%	0	0.0%	0	0.0%
mmu-mir-351-5p	X	50406492	50406515	-	edit	9	0	0.0%	0	0.0%	0	0.0%
mmu-mir-351-5p	X	50406492	50406515	-	edit	10	0	0.0%	0	0.0%	0	0.0%
mmu-mir-351-5p	X	50406492	50406515	-	edit	11	0	0.0%	0	0.0%	0	0.0%
mmu-mir-351-5p	X	50406492	50406515	-	edit	12	0	0.0%	0	0.0%	0	0.0%
mmu-mir-351-5p	X	50406492	50406515	-	edit	13	0	0.0%	0	0.0%	0	0.0%
mmu-mir-351-5p	X	50406492	50406515	-	edit	14	0	0.0%	0	0.0%	0	0.0%
mmu-mir-351-5p	X	50406492	50406515	-	edit	15	4	0.3%	0	0.0%	0	0.0%
mmu-mir-351-5p	X	50406492	50406515	-	edit	16	0	0.0%	0	0.0%	0	0.0%
mmu-mir-351-5p	X	50406492	50406515	-	edit	17	3	0.3%	0	0.0%	0	0.0%
mmu-mir-351-5p	X	50406492	50406515	-	edit	18	0	0.0%	0	0.0%	0	0.0%
mmu-mir-351-5p	X	50406492	50406515	-	edit	19	7	0.6%	3	0.6%	0	0.0%
mmu-mir-351-5p	X	50406492	50406515	-	edit	20	0	0.0%	0	0.0%	0	0.0%
mmu-mir-351-5p	X	50406492	50406515	-	edit	21	13	1.0%	0	0.0%	0	0.0%
mmu-mir-351-5p	X	50406492	50406515	-	edit	22	0	0.0%	0	0.0%	0	0.0%
EDITED							27	2.2%	3	0.6%	0	0.0%
CANONICAL							1170	97.8%	509	99.4%	174	100.0%
TOTAL							1197		512		174	

miRNA	Chr	Start	Stop	Strand	Type	Position	P7 reads	P7%	P10 reads	P10%	P14 reads	P14%
mmu-mir-376b-3p	12	110961718	110961738	+	edit	1	0	0.0%	0	0.0%	0	0.0%
mmu-mir-376b-3p	12	110961718	110961738	+	edit	2	0	0.0%	0	0.0%	0	0.0%
mmu-mir-376b-3p	12	110961718	110961738	+	edit	3	0	0.0%	0	0.0%	0	0.0%
mmu-mir-376b-3p	12	110961718	110961738	+	edit	4	0	0.0%	0	0.0%	0	0.0%
mmu-mir-376b-3p	12	110961718	110961738	+	edit	5	0	0.0%	0	0.0%	0	0.0%
mmu-mir-376b-3p	12	110961718	110961738	+	edit	6	**23**	**11.6%**	**4**	**13.8%**	0	0.0%
mmu-mir-376b-3p	12	110961718	110961738	+	edit	7	0	0.0%	0	0.0%	0	0.0%
mmu-mir-376b-3p	12	110961718	110961738	+	edit	8	0	0.0%	0	0.0%	0	0.0%
mmu-mir-376b-3p	12	110961718	110961738	+	edit	9	0	0.0%	0	0.0%	0	0.0%
mmu-mir-376b-3p	12	110961718	110961738	+	edit	10	0	0.0%	0	0.0%	0	0.0%
mmu-mir-376b-3p	12	110961718	110961738	+	edit	11	0	0.0%	0	0.0%	0	0.0%
mmu-mir-376b-3p	12	110961718	110961738	+	edit	12	0	0.0%	0	0.0%	0	0.0%
mmu-mir-376b-3p	12	110961718	110961738	+	edit	13	0	0.0%	0	0.0%	0	0.0%
mmu-mir-376b-3p	12	110961718	110961738	+	edit	14	0	0.0%	0	0.0%	0	0.0%
mmu-mir-376b-3p	12	110961718	110961738	+	edit	15	0	0.0%	0	0.0%	0	0.0%
mmu-mir-376b-3p	12	110961718	110961738	+	edit	16	0	0.0%	0	0.0%	0	0.0%
mmu-mir-376b-3p	12	110961718	110961738	+	edit	17	0	0.0%	0	0.0%	0	0.0%
mmu-mir-376b-3p	12	110961718	110961738	+	edit	18	0	0.0%	0	0.0%	0	0.0%
mmu-mir-376b-3p	12	110961718	110961738	+	edit	19	0	0.0%	0	0.0%	0	0.0%
mmu-mir-376b-3p	12	110961718	110961738	+	edit	20	0	0.0%	0	0.0%	0	0.0%
mmu-mir-376b-3p	12	110961718	110961738	+	edit	21	0	0.0%	0	0.0%	0	0.0%
mmu-mir-376b-3p	12	110961718	110961738	+	edit	22	0	0.0%	0	0.0%	0	0.0%
EDITED							23	11.6%	4	13.8%	0	0.0%
CANONICAL							175	88.4%	25	86.2%	11	100.0%
TOTAL							198		29		11	

miRNA	Chr	Start	Stop	Strand	Type	Position	P7 reads	P7%	P10 reads	P10%	P14 reads	P14%
mmu-mir-378-3p	18	61557492	61557512	-	edit	1	0	0.0%	0	0.0%	0	0.0%
mmu-mir-378-3p	18	61557492	61557512	-	edit	2	0	0.0%	0	0.0%	0	0.0%
mmu-mir-378-3p	18	61557492	61557512	-	edit	3	0	0.0%	0	0.0%	0	0.0%
mmu-mir-378-3p	18	61557492	61557512	-	edit	4	0	0.0%	0	0.0%	0	0.0%
mmu-mir-378-3p	18	61557492	61557512	-	edit	5	0	0.0%	0	0.0%	0	0.0%
mmu-mir-378-3p	18	61557492	61557512	-	edit	6	0	0.0%	0	0.0%	0	0.0%
mmu-mir-378-3p	18	61557492	61557512	-	edit	7	0	0.0%	0	0.0%	0	0.0%
mmu-mir-378-3p	18	61557492	61557512	-	edit	8	0	0.0%	0	0.0%	0	0.0%
mmu-mir-378-3p	18	61557492	61557512	-	edit	9	0	0.0%	0	0.0%	0	0.0%
mmu-mir-378-3p	18	61557492	61557512	-	edit	10	0	0.0%	0	0.0%	0	0.0%
mmu-mir-378-3p	18	61557492	61557512	-	edit	11	0	0.0%	0	0.0%	0	0.0%
mmu-mir-378-3p	18	61557492	61557512	-	edit	12	0	0.0%	0	0.0%	0	0.0%
mmu-mir-378-3p	18	61557492	61557512	-	edit	13	0	0.0%	0	0.0%	0	0.0%
mmu-mir-378-3p	18	61557492	61557512	-	edit	14	0	0.0%	0	0.0%	0	0.0%
mmu-mir-378-3p	18	61557492	61557512	-	edit	15	9	0.4%	0	0.0%	0	0.0%
mmu-mir-378-3p	18	61557492	61557512	-	edit	16	10	0.4%	9	1.3%	3	0.5%
mmu-mir-378-3p	18	61557492	61557512	-	edit	17	0	0.0%	0	0.0%	0	0.0%
mmu-mir-378-3p	18	61557492	61557512	-	edit	18	0	0.0%	0	0.0%	0	0.0%
mmu-mir-378-3p	18	61557492	61557512	-	edit	19	0	0.0%	4	0.6%	0	0.0%
mmu-mir-378-3p	18	61557492	61557512	-	edit	20	0	0.0%	0	0.0%	0	0.0%
mmu-mir-378-3p	18	61557492	61557512	-	edit	21	0	0.0%	0	0.0%	0	0.0%
mmu-mir-378-3p	18	61557492	61557512	-	edit	22	0	0.0%	0	0.0%	0	0.0%
EDITED							19	0.8%	13	1.9%	3	0.5%
CANONICAL							2316	99.2%	654	98.1%	594	99.5%
TOTAL							2335		667		597	

miRNA	Chr	Start	Stop	Strand	Type	Position	P7 reads	P7%	P10 reads	P10%	P14 reads	P14%
mmu-mir-379-5p	12	110947275	110947295	+	3' Edit	3'	27	0.6%	0	0.0%	7	0.9%
mmu-mir-379-5p	12	110947275	110947295	+	Indel	21	3	0.1%	0	0.0%	0	0.0%
mmu-mir-379-5p	12	110947275	110947295	+	edit	1	0	0.0%	0	0.0%	0	0.0%
mmu-mir-379-5p	12	110947275	110947295	+	edit	2	0	0.0%	0	0.0%	0	0.0%
mmu-mir-379-5p	12	110947275	110947295	+	edit	3	0	0.0%	0	0.0%	0	0.0%
mmu-mir-379-5p	12	110947275	110947295	+	edit	4	0	0.0%	0	0.0%	0	0.0%
mmu-mir-379-5p	12	110947275	110947295	+	edit	5	0	0.0%	0	0.0%	0	0.0%
mmu-mir-379-5p	12	110947275	110947295	+	edit	6	0	0.0%	0	0.0%	0	0.0%
mmu-mir-379-5p	12	110947275	110947295	+	edit	7	0	0.0%	0	0.0%	0	0.0%
mmu-mir-379-5p	12	110947275	110947295	+	edit	8	0	0.0%	0	0.0%	0	0.0%
mmu-mir-379-5p	12	110947275	110947295	+	edit	9	0	0.0%	0	0.0%	0	0.0%
mmu-mir-379-5p	12	110947275	110947295	+	edit	10	0	0.0%	0	0.0%	0	0.0%
mmu-mir-379-5p	12	110947275	110947295	+	edit	11	0	0.0%	0	0.0%	0	0.0%
mmu-mir-379-5p	12	110947275	110947295	+	edit	12	0	0.0%	0	0.0%	0	0.0%
mmu-mir-379-5p	12	110947275	110947295	+	edit	13	0	0.0%	0	0.0%	0	0.0%
mmu-mir-379-5p	12	110947275	110947295	+	edit	14	0	0.0%	0	0.0%	0	0.0%
mmu-mir-379-5p	12	110947275	110947295	+	edit	15	0	0.0%	0	0.0%	0	0.0%
mmu-mir-379-5p	12	110947275	110947295	+	edit	16	0	0.0%	0	0.0%	0	0.0%
mmu-mir-379-5p	12	110947275	110947295	+	edit	17	0	0.0%	0	0.0%	0	0.0%
mmu-mir-379-5p	12	110947275	110947295	+	edit	18	0	0.0%	0	0.0%	0	0.0%
mmu-mir-379-5p	12	110947275	110947295	+	edit	19	0	0.0%	4	0.2%	0	0.0%
mmu-mir-379-5p	12	110947275	110947295	+	edit	20	0	0.0%	0	0.0%	0	0.0%
mmu-mir-379-5p	12	110947275	110947295	+	edit	21	4	0.1%	5	0.3%	4	0.5%
mmu-mir-379-5p	12	110947275	110947295	+	edit	22	0	0.0%	0	0.0%	0	0.0%
EDITED							34	0.8%	9	0.5%	11	1.5%
CANONICAL							4497	99.2%	1794	99.5%	738	98.5%
TOTAL							4531		1803		749	

miRNA	Chr	Start	Stop	Strand	Type	Position	P7 reads	P7%	P10 reads	P10%	P14 reads	P14%
mmu-mir-382-3p	12	110972029	110972050	+	edit	1	0	0.0%	0	0.0%	0	0.0%
mmu-mir-382-3p	12	110972029	110972050	+	edit	2	0	0.0%	0	0.0%	0	0.0%
mmu-mir-382-3p	12	110972029	110972050	+	edit	3	0	0.0%	0	0.0%	0	0.0%
mmu-mir-382-3p	12	110972029	110972050	+	edit	4	0	0.0%	0	0.0%	0	0.0%
mmu-mir-382-3p	12	110972029	110972050	+	edit	5	0	0.0%	0	0.0%	0	0.0%
mmu-mir-382-3p	12	110972029	110972050	+	edit	6	0	0.0%	0	0.0%	0	0.0%
mmu-mir-382-3p	12	110972029	110972050	+	edit	7	0	0.0%	0	0.0%	0	0.0%
mmu-mir-382-3p	12	110972029	110972050	+	edit	8	0	0.0%	0	0.0%	0	0.0%
mmu-mir-382-3p	12	110972029	110972050	+	edit	9	0	0.0%	0	0.0%	0	0.0%
mmu-mir-382-3p	12	110972029	110972050	+	edit	10	0	0.0%	0	0.0%	0	0.0%
mmu-mir-382-3p	12	110972029	110972050	+	edit	11	0	0.0%	0	0.0%	0	0.0%
mmu-mir-382-3p	12	110972029	110972050	+	edit	12	0	0.0%	0	0.0%	0	0.0%
mmu-mir-382-3p	12	110972029	110972050	+	edit	13	0	0.0%	3	0.9%	0	0.0%
mmu-mir-382-3p	12	110972029	110972050	+	edit	14	0	0.0%	0	0.0%	0	0.0%
mmu-mir-382-3p	12	110972029	110972050	+	edit	15	0	0.0%	0	0.0%	0	0.0%
mmu-mir-382-3p	12	110972029	110972050	+	edit	16	0	0.0%	0	0.0%	0	0.0%
mmu-mir-382-3p	12	110972029	110972050	+	edit	17	8	0.7%	0	0.0%	0	0.0%
mmu-mir-382-3p	12	110972029	110972050	+	edit	18	0	0.0%	0	0.0%	0	0.0%
mmu-mir-382-3p	12	110972029	110972050	+	edit	19	0	0.0%	0	0.0%	0	0.0%
mmu-mir-382-3p	12	110972029	110972050	+	edit	20	0	0.0%	0	0.0%	0	0.0%
mmu-mir-382-3p	12	110972029	110972050	+	edit	21	0	0.0%	0	0.0%	0	0.0%
mmu-mir-382-3p	12	110972029	110972050	+	edit	22	0	0.0%	0	0.0%	0	0.0%
EDITED							8	0.7%	3	0.9%	0	0.0%
CANONICAL							1131	99.3%	349	99.1%	200	100.0%
TOTAL							1139		352		200	

miRNA	Chr	Start	Stop	Strand	Type	Position	P7 reads	P7%	P10 reads	P10%	P14 reads	P14%
mmu-mir-382-5p	12	110971991	110972012	+	edit	1	0	0.0%	0	0.0%	0	0.0%
mmu-mir-382-5p	12	110971991	110972012	+	edit	2	0	0.0%	0	0.0%	0	0.0%
mmu-mir-382-5p	12	110971991	110972012	+	edit	3	0	0.0%	0	0.0%	0	0.0%
mmu-mir-382-5p	12	110971991	110972012	+	edit	4	0	0.0%	0	0.0%	0	0.0%
mmu-mir-382-5p	12	110971991	110972012	+	edit	5	0	0.0%	0	0.0%	0	0.0%
mmu-mir-382-5p	12	110971991	110972012	+	edit	6	0	0.0%	0	0.0%	0	0.0%
mmu-mir-382-5p	12	110971991	110972012	+	edit	7	0	0.0%	0	0.0%	0	0.0%
mmu-mir-382-5p	12	110971991	110972012	+	edit	8	0	0.0%	0	0.0%	0	0.0%
mmu-mir-382-5p	12	110971991	110972012	+	edit	9	0	0.0%	0	0.0%	0	0.0%
mmu-mir-382-5p	12	110971991	110972012	+	edit	10	0	0.0%	0	0.0%	0	0.0%
mmu-mir-382-5p	12	110971991	110972012	+	edit	11	0	0.0%	0	0.0%	0	0.0%
mmu-mir-382-5p	12	110971991	110972012	+	edit	12	0	0.0%	0	0.0%	0	0.0%
mmu-mir-382-5p	12	110971991	110972012	+	edit	13	0	0.0%	0	0.0%	0	0.0%
mmu-mir-382-5p	12	110971991	110972012	+	edit	14	0	0.0%	0	0.0%	0	0.0%
mmu-mir-382-5p	12	110971991	110972012	+	edit	15	22	0.8%	8	0.9%	0	0.0%
mmu-mir-382-5p	12	110971991	110972012	+	edit	16	0	0.0%	0	0.0%	0	0.0%
mmu-mir-382-5p	12	110971991	110972012	+	edit	17	0	0.0%	0	0.0%	0	0.0%
mmu-mir-382-5p	12	110971991	110972012	+	edit	18	0	0.0%	0	0.0%	0	0.0%
mmu-mir-382-5p	12	110971991	110972012	+	edit	19	3	0.1%	0	0.0%	0	0.0%
mmu-mir-382-5p	12	110971991	110972012	+	edit	20	0	0.0%	0	0.0%	0	0.0%
mmu-mir-382-5p	12	110971991	110972012	+	edit	21	0	0.0%	0	0.0%	0	0.0%
mmu-mir-382-5p	12	110971991	110972012	+	edit	22	3	0.1%	0	0.0%	0	0.0%
EDITED							28	1.0%	8	0.9%	0	0.0%
CANONICAL							2846	99.0%	902	99.1%	397	100.0%
TOTAL							2874		910		397	

miRNA	Chr	Start	Stop	Strand	Type	Position	P7 reads	P7%	P10 reads	P10%	P14 reads	P14%
mmu-mir-409-3p	12	110981414	110981435	+	edit	1	0	0.0%	0	0.0%	0	0.0%
mmu-mir-409-3p	12	110981414	110981435	+	edit	2	0	0.0%	0	0.0%	0	0.0%
mmu-mir-409-3p	12	110981414	110981435	+	edit	3	0	0.0%	0	0.0%	0	0.0%
mmu-mir-409-3p	12	110981414	110981435	+	edit	4	0	0.0%	0	0.0%	0	0.0%
mmu-mir-409-3p	12	110981414	110981435	+	edit	5	0	0.0%	0	0.0%	0	0.0%
mmu-mir-409-3p	12	110981414	110981435	+	edit	6	0	0.0%	0	0.0%	0	0.0%
mmu-mir-409-3p	12	110981414	110981435	+	edit	7	0	0.0%	0	0.0%	0	0.0%
mmu-mir-409-3p	12	110981414	110981435	+	edit	8	0	0.0%	0	0.0%	0	0.0%
mmu-mir-409-3p	12	110981414	110981435	+	edit	9	0	0.0%	0	0.0%	0	0.0%
mmu-mir-409-3p	12	110981414	110981435	+	edit	10	0	0.0%	0	0.0%	0	0.0%
mmu-mir-409-3p	12	110981414	110981435	+	edit	11	0	0.0%	0	0.0%	0	0.0%
mmu-mir-409-3p	12	110981414	110981435	+	edit	12	0	0.0%	0	0.0%	0	0.0%
mmu-mir-409-3p	12	110981414	110981435	+	edit	13	0	0.0%	0	0.0%	0	0.0%
mmu-mir-409-3p	12	110981414	110981435	+	edit	14	0	0.0%	0	0.0%	0	0.0%
mmu-mir-409-3p	12	110981414	110981435	+	edit	15	0	0.0%	0	0.0%	0	0.0%
mmu-mir-409-3p	12	110981414	110981435	+	edit	16	0	0.0%	0	0.0%	0	0.0%
mmu-mir-409-3p	12	110981414	110981435	+	edit	17	0	0.0%	0	0.0%	0	0.0%
mmu-mir-409-3p	12	110981414	110981435	+	edit	18	0	0.0%	0	0.0%	0	0.0%
mmu-mir-409-3p	12	110981414	110981435	+	edit	19	7	1.9%	0	0.0%	0	0.0%
mmu-mir-409-3p	12	110981414	110981435	+	edit	20	0	0.0%	0	0.0%	0	0.0%
mmu-mir-409-3p	12	110981414	110981435	+	edit	21	0	0.0%	0	0.0%	0	0.0%
mmu-mir-409-3p	12	110981414	110981435	+	edit	22	0	0.0%	0	0.0%	0	0.0%
EDITED							7	1.9%	0	0.0%	0	0.0%
CANONICAL							370	98.1%	89	100.0%	28	100.0%
TOTAL							377		89		28	

miRNA	Chr	Start	Stop	Strand	Type	Position	P7 reads	P7%	P10 reads	P10%	P14 reads	P14%
mmu-mir-411-5p	12	110948400	110948420	+	edit	1	0	0.0%	0	0.0%	0	0.0%
mmu-mir-411-5p	12	110948400	110948420	+	edit	2	0	0.0%	0	0.0%	0	0.0%
mmu-mir-411-5p	12	110948400	110948420	+	edit	3	0	0.0%	0	0.0%	0	0.0%
mmu-mir-411-5p	12	110948400	110948420	+	edit	4	0	0.0%	0	0.0%	0	0.0%
mmu-mir-411-5p	12	110948400	110948420	+	edit	5	9	0.7%	0	0.0%	0	0.0%
mmu-mir-411-5p	12	110948400	110948420	+	edit	6	0	0.0%	0	0.0%	0	0.0%
mmu-mir-411-5p	12	110948400	110948420	+	edit	7	0	0.0%	0	0.0%	0	0.0%
mmu-mir-411-5p	12	110948400	110948420	+	edit	8	0	0.0%	0	0.0%	0	0.0%
mmu-mir-411-5p	12	110948400	110948420	+	edit	9	0	0.0%	0	0.0%	0	0.0%
mmu-mir-411-5p	12	110948400	110948420	+	edit	10	0	0.0%	0	0.0%	0	0.0%
mmu-mir-411-5p	12	110948400	110948420	+	edit	11	0	0.0%	0	0.0%	0	0.0%
mmu-mir-411-5p	12	110948400	110948420	+	edit	12	0	0.0%	0	0.0%	0	0.0%
mmu-mir-411-5p	12	110948400	110948420	+	edit	13	0	0.0%	0	0.0%	0	0.0%
mmu-mir-411-5p	12	110948400	110948420	+	edit	14	0	0.0%	0	0.0%	0	0.0%
mmu-mir-411-5p	12	110948400	110948420	+	edit	15	0	0.0%	0	0.0%	0	0.0%
mmu-mir-411-5p	12	110948400	110948420	+	edit	16	0	0.0%	0	0.0%	0	0.0%
mmu-mir-411-5p	12	110948400	110948420	+	edit	17	0	0.0%	0	0.0%	0	0.0%
mmu-mir-411-5p	12	110948400	110948420	+	edit	18	0	0.0%	0	0.0%	0	0.0%
mmu-mir-411-5p	12	110948400	110948420	+	edit	19	0	0.0%	0	0.0%	0	0.0%
mmu-mir-411-5p	12	110948400	110948420	+	edit	20	0	0.0%	0	0.0%	0	0.0%
mmu-mir-411-5p	12	110948400	110948420	+	edit	21	0	0.0%	0	0.0%	0	0.0%
mmu-mir-411-5p	12	110948400	110948420	+	edit	22	0	0.0%	0	0.0%	0	0.0%
EDITED							9	0.7%	0	0.0%	0	0.0%
CANONICAL							1289	99.3%	473	100.0%	185	100.0%
TOTAL							1298		473		185	

miRNA	Chr	Start	Stop	Strand	Type	Position	P7 reads	P7%	P10 reads	P10%	P14 reads	P14%
mmu-mir-423-3p	11	76891588	76891610	-	edit	1	0	0.0%	0	0.0%	0	0.0%
mmu-mir-423-3p	11	76891588	76891610	-	edit	2	0	0.0%	0	0.0%	0	0.0%
mmu-mir-423-3p	11	76891588	76891610	-	edit	3	0	0.0%	0	0.0%	0	0.0%
mmu-mir-423-3p	11	76891588	76891610	-	edit	4	0	0.0%	0	0.0%	0	0.0%
mmu-mir-423-3p	11	76891588	76891610	-	edit	5	0	0.0%	0	0.0%	0	0.0%
mmu-mir-423-3p	11	76891588	76891610	-	edit	6	0	0.0%	0	0.0%	0	0.0%
mmu-mir-423-3p	11	76891588	76891610	-	edit	7	0	0.0%	0	0.0%	0	0.0%
mmu-mir-423-3p	11	76891588	76891610	-	edit	8	0	0.0%	0	0.0%	0	0.0%
mmu-mir-423-3p	11	76891588	76891610	-	edit	9	0	0.0%	0	0.0%	0	0.0%
mmu-mir-423-3p	11	76891588	76891610	-	edit	10	0	0.0%	0	0.0%	0	0.0%
mmu-mir-423-3p	11	76891588	76891610	-	edit	11	0	0.0%	0	0.0%	0	0.0%
mmu-mir-423-3p	11	76891588	76891610	-	edit	12	0	0.0%	0	0.0%	0	0.0%
mmu-mir-423-3p	11	76891588	76891610	-	edit	13	0	0.0%	0	0.0%	0	0.0%
mmu-mir-423-3p	11	76891588	76891610	-	edit	14	0	0.0%	0	0.0%	0	0.0%
mmu-mir-423-3p	11	76891588	76891610	-	edit	15	0	0.0%	0	0.0%	0	0.0%
mmu-mir-423-3p	11	76891588	76891610	-	edit	16	4	0.6%	7	1.6%	0	0.0%
mmu-mir-423-3p	11	76891588	76891610	-	edit	17	0	0.0%	0	0.0%	0	0.0%
mmu-mir-423-3p	11	76891588	76891610	-	edit	18	3	0.5%	0	0.0%	0	0.0%
mmu-mir-423-3p	11	76891588	76891610	-	edit	19	0	0.0%	0	0.0%	0	0.0%
mmu-mir-423-3p	11	76891588	76891610	-	edit	20	3	0.5%	0	0.0%	0	0.0%
mmu-mir-423-3p	11	76891588	76891610	-	edit	21	0	0.0%	0	0.0%	0	0.0%
mmu-mir-423-3p	11	76891588	76891610	-	edit	22	0	0.0%	0	0.0%	0	0.0%
EDITED							10	1.6%	7	1.6%	0	0.0%
CANONICAL							627	98.4%	430	98.4%	266	100.0%
TOTAL							637		437		266	

miRNA	Chr	Start	Stop	Strand	Type	Position	P7 reads	P7%	P10 reads	P10%	P14 reads	P14%
mmu-mir-423-5p	11	76891624	76891646	-	5' Edit	5'	9	0.0%	0	0.0%	9	0.1%
mmu-mir-423-5p	11	76891624	76891646	-	5' Indel	5'	9	0.0%	0	0.0%	0	0.0%
mmu-mir-423-5p	11	76891624	76891646	-	Indel	21	17	0.0%	3	0.0%	9	0.1%
mmu-mir-423-5p	11	76891624	76891646	-	Indel	22	15	0.0%	0	0.0%	10	0.1%
mmu-mir-423-5p	11	76891624	76891646	-	Indel	23	0	0.0%	0	0.0%	3	0.0%
mmu-mir-423-5p	11	76891624	76891646	-	edit	1	0	0.0%	0	0.0%	0	0.0%
mmu-mir-423-5p	11	76891624	76891646	-	edit	2	0	0.0%	0	0.0%	0	0.0%
mmu-mir-423-5p	11	76891624	76891646	-	edit	3	0	0.0%	0	0.0%	0	0.0%
mmu-mir-423-5p	11	76891624	76891646	-	edit	4	5	0.0%	0	0.0%	4	0.0%
mmu-mir-423-5p	11	76891624	76891646	-	edit	5	11	0.0%	9	0.0%	3	0.0%
mmu-mir-423-5p	11	76891624	76891646	-	edit	6	4	0.0%	0	0.0%	0	0.0%
mmu-mir-423-5p	11	76891624	76891646	-	edit	7	0	0.0%	0	0.0%	0	0.0%
mmu-mir-423-5p	11	76891624	76891646	-	edit	8	27	0.1%	11	0.0%	10	0.1%
mmu-mir-423-5p	11	76891624	76891646	-	edit	9	6	0.0%	10	0.0%	0	0.0%
mmu-mir-423-5p	11	76891624	76891646	-	edit	10	0	0.0%	0	0.0%	0	0.0%
mmu-mir-423-5p	11	76891624	76891646	-	edit	11	0	0.0%	0	0.0%	0	0.0%
mmu-mir-423-5p	11	76891624	76891646	-	edit	12	0	0.0%	0	0.0%	0	0.0%
mmu-mir-423-5p	11	76891624	76891646	-	edit	13	29	0.1%	44	0.2%	27	0.2%
mmu-mir-423-5p	11	76891624	76891646	-	edit	14	0	0.0%	0	0.0%	0	0.0%
mmu-mir-423-5p	11	76891624	76891646	-	edit	15	121	0.3%	65	0.3%	102	0.7%
mmu-mir-423-5p	11	76891624	76891646	-	edit	16	50	0.1%	66	0.3%	20	0.1%
mmu-mir-423-5p	11	76891624	76891646	-	edit	17	98	0.3%	303	1.3%	59	0.4%
mmu-mir-423-5p	11	76891624	76891646	-	edit	18	31	0.1%	24	0.1%	5	0.0%
mmu-mir-423-5p	11	76891624	76891646	-	edit	19	296	0.8%	366	1.6%	109	0.7%
mmu-mir-423-5p	11	76891624	76891646	-	edit	20	394	1.1%	201	0.9%	250	1.6%
mmu-mir-423-5p	11	76891624	76891646	-	edit	21	190	0.5%	44	0.2%	98	0.6%
mmu-mir-423-5p	11	76891624	76891646	-	edit	22	57	0.2%	12	0.1%	24	0.2%
mmu-mir-423-5p	11	76891624	76891646	-	edit	23	5	0.0%	0	0.0%	0	0.0%
EDITED							1373	3.7%	1158	5.0%	742	4.8%
CANONICAL							35295	96.3%	21790	95.0%	14764	95.2%
TOTAL							36668		22948		15506	

miRNA	Chr	Start	Stop	Strand	Type	Position	P7 reads	P7%	P10 reads	P10%	P14 reads	P14%
mmu-mir-425-5p	9	108471120	108471142	+	edit	1	0	0.0%	0	0.0%	0	0.0%
mmu-mir-425-5p	9	108471120	108471142	+	edit	2	0	0.0%	0	0.0%	0	0.0%
mmu-mir-425-5p	9	108471120	108471142	+	edit	3	0	0.0%	0	0.0%	0	0.0%
mmu-mir-425-5p	9	108471120	108471142	+	edit	4	0	0.0%	0	0.0%	0	0.0%
mmu-mir-425-5p	9	108471120	108471142	+	edit	5	0	0.0%	0	0.0%	0	0.0%
mmu-mir-425-5p	9	108471120	108471142	+	edit	6	0	0.0%	0	0.0%	0	0.0%
mmu-mir-425-5p	9	108471120	108471142	+	edit	7	0	0.0%	0	0.0%	0	0.0%
mmu-mir-425-5p	9	108471120	108471142	+	edit	8	0	0.0%	0	0.0%	0	0.0%
mmu-mir-425-5p	9	108471120	108471142	+	edit	9	0	0.0%	0	0.0%	0	0.0%
mmu-mir-425-5p	9	108471120	108471142	+	edit	10	0	0.0%	0	0.0%	0	0.0%
mmu-mir-425-5p	9	108471120	108471142	+	edit	11	0	0.0%	0	0.0%	0	0.0%
mmu-mir-425-5p	9	108471120	108471142	+	edit	12	0	0.0%	0	0.0%	0	0.0%
mmu-mir-425-5p	9	108471120	108471142	+	edit	13	0	0.0%	0	0.0%	0	0.0%
mmu-mir-425-5p	9	108471120	108471142	+	edit	14	0	0.0%	0	0.0%	0	0.0%
mmu-mir-425-5p	9	108471120	108471142	+	edit	15	0	0.0%	0	0.0%	0	0.0%
mmu-mir-425-5p	9	108471120	108471142	+	edit	16	0	0.0%	0	0.0%	0	0.0%
mmu-mir-425-5p	9	108471120	108471142	+	edit	17	4	5.8%	0	0.0%	0	0.0%
mmu-mir-425-5p	9	108471120	108471142	+	edit	18	0	0.0%	0	0.0%	0	0.0%
mmu-mir-425-5p	9	108471120	108471142	+	edit	19	0	0.0%	0	0.0%	0	0.0%
mmu-mir-425-5p	9	108471120	108471142	+	edit	20	0	0.0%	0	0.0%	0	0.0%
mmu-mir-425-5p	9	108471120	108471142	+	edit	21	0	0.0%	0	0.0%	0	0.0%
mmu-mir-425-5p	9	108471120	108471142	+	edit	22	0	0.0%	0	0.0%	0	0.0%
EDITED							4	5.8%	0	0.0%	0	0.0%
CANONICAL							65	94.2%	44	100.0%	60	100.0%
TOTAL							69		44		60	

miRNA	Chr	Start	Stop	Strand	Type	Position	P7 reads	P7%	P10 reads	P10%	P14 reads	P14%
mmu-mir-433-3p	12	110829991	110830012	+	Indel	20	3	0.1%	0	0.0%	0	0.0%
mmu-mir-433-3p	12	110829991	110830012	+	edit	1	0	0.0%	0	0.0%	0	0.0%
mmu-mir-433-3p	12	110829991	110830012	+	edit	2	0	0.0%	0	0.0%	0	0.0%
mmu-mir-433-3p	12	110829991	110830012	+	edit	3	0	0.0%	0	0.0%	0	0.0%
mmu-mir-433-3p	12	110829991	110830012	+	edit	4	0	0.0%	0	0.0%	0	0.0%
mmu-mir-433-3p	12	110829991	110830012	+	edit	5	0	0.0%	0	0.0%	0	0.0%
mmu-mir-433-3p	12	110829991	110830012	+	edit	6	0	0.0%	0	0.0%	0	0.0%
mmu-mir-433-3p	12	110829991	110830012	+	edit	7	0	0.0%	0	0.0%	0	0.0%
mmu-mir-433-3p	12	110829991	110830012	+	edit	8	4	0.1%	0	0.0%	0	0.0%
mmu-mir-433-3p	12	110829991	110830012	+	edit	9	0	0.0%	0	0.0%	0	0.0%
mmu-mir-433-3p	12	110829991	110830012	+	edit	10	0	0.0%	0	0.0%	0	0.0%
mmu-mir-433-3p	12	110829991	110830012	+	edit	11	0	0.0%	0	0.0%	0	0.0%
mmu-mir-433-3p	12	110829991	110830012	+	edit	12	0	0.0%	0	0.0%	0	0.0%
mmu-mir-433-3p	12	110829991	110830012	+	edit	13	0	0.0%	0	0.0%	0	0.0%
mmu-mir-433-3p	12	110829991	110830012	+	edit	14	0	0.0%	0	0.0%	0	0.0%
mmu-mir-433-3p	12	110829991	110830012	+	edit	15	17	0.6%	4	0.5%	0	0.0%
mmu-mir-433-3p	12	110829991	110830012	+	edit	16	0	0.0%	0	0.0%	0	0.0%
mmu-mir-433-3p	12	110829991	110830012	+	edit	17	0	0.0%	0	0.0%	0	0.0%
mmu-mir-433-3p	12	110829991	110830012	+	edit	18	0	0.0%	0	0.0%	0	0.0%
mmu-mir-433-3p	12	110829991	110830012	+	edit	19	10	0.3%	0	0.0%	0	0.0%
mmu-mir-433-3p	12	110829991	110830012	+	edit	20	0	0.0%	0	0.0%	0	0.0%
mmu-mir-433-3p	12	110829991	110830012	+	edit	21	0	0.0%	0	0.0%	0	0.0%
mmu-mir-433-3p	12	110829991	110830012	+	edit	22	0	0.0%	0	0.0%	0	0.0%
EDITED							34	1.2%	4	0.5%	0	0.0%
CANONICAL							2883	98.8%	760	99.5%	280	100.0%
TOTAL							2917		764		280	

miRNA	Chr	Start	Stop	Strand	Type	Position	P7 reads	P7%	P10 reads	P10%	P14 reads	P14%
mmu-mir-449a-5p	13	113827757	113827778	+	3' Edit	3'	0	0.0%	0	0.0%	3	0.6%
mmu-mir-449a-5p	13	113827757	113827778	+	3' Indel	3'	0	0.0%	0	0.0%	19	3.7%
mmu-mir-449a-5p	13	113827757	113827778	+	edit	1	0	0.0%	0	0.0%	0	0.0%
mmu-mir-449a-5p	13	113827757	113827778	+	edit	2	0	0.0%	0	0.0%	0	0.0%
mmu-mir-449a-5p	13	113827757	113827778	+	edit	3	0	0.0%	0	0.0%	0	0.0%
mmu-mir-449a-5p	13	113827757	113827778	+	edit	4	0	0.0%	0	0.0%	0	0.0%
mmu-mir-449a-5p	13	113827757	113827778	+	edit	5	0	0.0%	0	0.0%	0	0.0%
mmu-mir-449a-5p	13	113827757	113827778	+	edit	6	0	0.0%	0	0.0%	0	0.0%
mmu-mir-449a-5p	13	113827757	113827778	+	edit	7	0	0.0%	0	0.0%	0	0.0%
mmu-mir-449a-5p	13	113827757	113827778	+	edit	8	0	0.0%	0	0.0%	0	0.0%
mmu-mir-449a-5p	13	113827757	113827778	+	edit	9	0	0.0%	0	0.0%	0	0.0%
mmu-mir-449a-5p	13	113827757	113827778	+	edit	10	0	0.0%	0	0.0%	0	0.0%
mmu-mir-449a-5p	13	113827757	113827778	+	edit	11	0	0.0%	0	0.0%	0	0.0%
mmu-mir-449a-5p	13	113827757	113827778	+	edit	12	0	0.0%	0	0.0%	0	0.0%
mmu-mir-449a-5p	13	113827757	113827778	+	edit	13	0	0.0%	0	0.0%	0	0.0%
mmu-mir-449a-5p	13	113827757	113827778	+	edit	14	0	0.0%	0	0.0%	0	0.0%
mmu-mir-449a-5p	13	113827757	113827778	+	edit	15	0	0.0%	0	0.0%	0	0.0%
mmu-mir-449a-5p	13	113827757	113827778	+	edit	16	0	0.0%	0	0.0%	0	0.0%
mmu-mir-449a-5p	13	113827757	113827778	+	edit	17	0	0.0%	0	0.0%	0	0.0%
mmu-mir-449a-5p	13	113827757	113827778	+	edit	18	0	0.0%	0	0.0%	0	0.0%
mmu-mir-449a-5p	13	113827757	113827778	+	edit	19	0	0.0%	0	0.0%	0	0.0%
mmu-mir-449a-5p	13	113827757	113827778	+	edit	20	0	0.0%	0	0.0%	0	0.0%
mmu-mir-449a-5p	13	113827757	113827778	+	edit	21	0	0.0%	0	0.0%	0	0.0%
mmu-mir-449a-5p	13	113827757	113827778	+	edit	22	0	0.0%	0	0.0%	0	0.0%
EDITED							0	0.0%	0	0.0%	22	4.3%
CANONICAL							41	100.0%	27	100.0%	493	95.7%
TOTAL							41		27		515	

miRNA	Chr	Start	Stop	Strand	Type	Position	P7 reads	P7%	P10 reads	P10%	P14 reads	P14%
mmu-mir-449c-5p	13	113826216	113826236	+	3' Edit	3'	0	0.0%	0	0.0%	27	2.8%
mmu-mir-449c-5p	13	113826216	113826236	+	edit	1	0	0.0%	0	0.0%	0	0.0%
mmu-mir-449c-5p	13	113826216	113826236	+	edit	2	0	0.0%	0	0.0%	0	0.0%
mmu-mir-449c-5p	13	113826216	113826236	+	edit	3	0	0.0%	0	0.0%	0	0.0%
mmu-mir-449c-5p	13	113826216	113826236	+	edit	4	0	0.0%	0	0.0%	0	0.0%
mmu-mir-449c-5p	13	113826216	113826236	+	edit	5	0	0.0%	0	0.0%	0	0.0%
mmu-mir-449c-5p	13	113826216	113826236	+	edit	6	0	0.0%	0	0.0%	0	0.0%
mmu-mir-449c-5p	13	113826216	113826236	+	edit	7	0	0.0%	0	0.0%	0	0.0%
mmu-mir-449c-5p	13	113826216	113826236	+	edit	8	0	0.0%	0	0.0%	0	0.0%
mmu-mir-449c-5p	13	113826216	113826236	+	edit	9	0	0.0%	0	0.0%	0	0.0%
mmu-mir-449c-5p	13	113826216	113826236	+	edit	10	0	0.0%	0	0.0%	0	0.0%
mmu-mir-449c-5p	13	113826216	113826236	+	edit	11	0	0.0%	0	0.0%	3	0.3%
mmu-mir-449c-5p	13	113826216	113826236	+	edit	12	0	0.0%	0	0.0%	0	0.0%
mmu-mir-449c-5p	13	113826216	113826236	+	edit	13	0	0.0%	0	0.0%	0	0.0%
mmu-mir-449c-5p	13	113826216	113826236	+	edit	14	0	0.0%	0	0.0%	0	0.0%
mmu-mir-449c-5p	13	113826216	113826236	+	edit	15	0	0.0%	0	0.0%	0	0.0%
mmu-mir-449c-5p	13	113826216	113826236	+	edit	16	0	0.0%	0	0.0%	0	0.0%
mmu-mir-449c-5p	13	113826216	113826236	+	edit	17	0	0.0%	0	0.0%	4	0.4%
mmu-mir-449c-5p	13	113826216	113826236	+	edit	18	0	0.0%	0	0.0%	0	0.0%
mmu-mir-449c-5p	13	113826216	113826236	+	edit	19	0	0.0%	0	0.0%	10	1.0%
mmu-mir-449c-5p	13	113826216	113826236	+	edit	20	0	0.0%	0	0.0%	0	0.0%
mmu-mir-449c-5p	13	113826216	113826236	+	edit	21	0	0.0%	0	0.0%	0	0.0%
mmu-mir-449c-5p	13	113826216	113826236	+	edit	22	0	0.0%	0	0.0%	0	0.0%
EDITED							0	0.0%	0	0.0%	44	4.6%
CANONICAL							64	100.0%	43	100.0%	912	95.4%
TOTAL							64		43		956	

miRNA	Chr	Start	Stop	Strand	Type	Position	P7 reads	P7%	P10 reads	P10%	P14 reads	P14%
mmu-mir-451-5p	11	77886688	77886709	+	Indel	20	4	1.0%	0	0.0%	0	0.0%
mmu-mir-451-5p	11	77886688	77886709	+	edit	1	0	0.0%	0	0.0%	0	0.0%
mmu-mir-451-5p	11	77886688	77886709	+	edit	2	0	0.0%	0	0.0%	0	0.0%
mmu-mir-451-5p	11	77886688	77886709	+	edit	3	0	0.0%	0	0.0%	0	0.0%
mmu-mir-451-5p	11	77886688	77886709	+	edit	4	0	0.0%	0	0.0%	0	0.0%
mmu-mir-451-5p	11	77886688	77886709	+	edit	5	0	0.0%	0	0.0%	0	0.0%
mmu-mir-451-5p	11	77886688	77886709	+	edit	6	0	0.0%	0	0.0%	0	0.0%
mmu-mir-451-5p	11	77886688	77886709	+	edit	7	0	0.0%	0	0.0%	0	0.0%
mmu-mir-451-5p	11	77886688	77886709	+	edit	8	0	0.0%	0	0.0%	0	0.0%
mmu-mir-451-5p	11	77886688	77886709	+	edit	9	0	0.0%	0	0.0%	0	0.0%
mmu-mir-451-5p	11	77886688	77886709	+	edit	10	0	0.0%	0	0.0%	0	0.0%
mmu-mir-451-5p	11	77886688	77886709	+	edit	11	0	0.0%	0	0.0%	0	0.0%
mmu-mir-451-5p	11	77886688	77886709	+	edit	12	0	0.0%	0	0.0%	0	0.0%
mmu-mir-451-5p	11	77886688	77886709	+	edit	13	0	0.0%	0	0.0%	0	0.0%
mmu-mir-451-5p	11	77886688	77886709	+	edit	14	0	0.0%	0	0.0%	0	0.0%
mmu-mir-451-5p	11	77886688	77886709	+	edit	15	0	0.0%	0	0.0%	0	0.0%
mmu-mir-451-5p	11	77886688	77886709	+	edit	16	0	0.0%	0	0.0%	0	0.0%
mmu-mir-451-5p	11	77886688	77886709	+	edit	17	0	0.0%	0	0.0%	0	0.0%
mmu-mir-451-5p	11	77886688	77886709	+	edit	18	0	0.0%	0	0.0%	0	0.0%
mmu-mir-451-5p	11	77886688	77886709	+	edit	19	0	0.0%	0	0.0%	3	1.9%
mmu-mir-451-5p	11	77886688	77886709	+	edit	20	0	0.0%	0	0.0%	0	0.0%
mmu-mir-451-5p	11	77886688	77886709	+	edit	21	0	0.0%	0	0.0%	0	0.0%
mmu-mir-451-5p	11	77886688	77886709	+	edit	22	0	0.0%	0	0.0%	0	0.0%
EDITED							4	1.0%	0	0.0%	3	1.9%
CANONICAL							396	99.0%	103	100.0%	155	98.1%
TOTAL							400		103		158	

miRNA	Chr	Start	Stop	Strand	Type	Position	P7 reads	P7%	P10 reads	P10%	P14 reads	P14%
mmu-mir-465a-3p	X	64092238	64092259	-	Indel	22	0	0.0%	0	0.0%	4	0.3%
mmu-mir-465a-3p	X	64092238	64092259	-	edit	1	0	0.0%	0	0.0%	0	0.0%
mmu-mir-465a-3p	X	64092238	64092259	-	edit	2	0	0.0%	0	0.0%	0	0.0%
mmu-mir-465a-3p	X	64092238	64092259	-	edit	3	0	0.0%	0	0.0%	0	0.0%
mmu-mir-465a-3p	X	64092238	64092259	-	edit	4	0	0.0%	0	0.0%	0	0.0%
mmu-mir-465a-3p	X	64092238	64092259	-	edit	5	0	0.0%	0	0.0%	0	0.0%
mmu-mir-465a-3p	X	64092238	64092259	-	edit	6	0	0.0%	0	0.0%	0	0.0%
mmu-mir-465a-3p	X	64092238	64092259	-	edit	7	0	0.0%	0	0.0%	0	0.0%
mmu-mir-465a-3p	X	64092238	64092259	-	edit	8	0	0.0%	0	0.0%	0	0.0%
mmu-mir-465a-3p	X	64092238	64092259	-	edit	9	0	0.0%	0	0.0%	0	0.0%
mmu-mir-465a-3p	X	64092238	64092259	-	edit	10	0	0.0%	0	0.0%	0	0.0%
mmu-mir-465a-3p	X	64092238	64092259	-	edit	11	0	0.0%	0	0.0%	0	0.0%
mmu-mir-465a-3p	X	64092238	64092259	-	edit	12	0	0.0%	0	0.0%	0	0.0%
mmu-mir-465a-3p	X	64092238	64092259	-	edit	13	0	0.0%	0	0.0%	0	0.0%
mmu-mir-465a-3p	X	64092238	64092259	-	edit	14	0	0.0%	0	0.0%	0	0.0%
mmu-mir-465a-3p	X	64092238	64092259	-	edit	15	0	0.0%	0	0.0%	0	0.0%
mmu-mir-465a-3p	X	64092238	64092259	-	edit	16	0	0.0%	3	0.2%	0	0.0%
mmu-mir-465a-3p	X	64092238	64092259	-	edit	17	0	0.0%	33	1.7%	4	0.2%
mmu-mir-465a-3p	X	64092238	64092259	-	edit	18	0	0.0%	0	0.0%	0	0.0%
mmu-mir-465a-3p	X	64092238	64092259	-	edit	19	4	0.6%	11	0.6%	15	0.9%
mmu-mir-465a-3p	X	64092238	64092259	-	edit	20	0	0.0%	0	0.0%	0	0.0%
mmu-mir-465a-3p	X	64092238	64092259	-	edit	21	0	0.0%	0	0.0%	0	0.0%
mmu-mir-465a-3p	X	64092238	64092259	-	edit	22	0	0.0%	0	0.0%	0	0.0%
EDITED							4	0.6%	47	2.5%	23	1.4%
CANONICAL							720	99.4%	1856	97.5%	1589	98.6%
TOTAL							724		1903		1612	

miRNA	Chr	Start	Stop	Strand	Type	Position	P7 reads	P7%	P10 reads	P10%	P14 reads	P14%
mmu-mir-465a-5p	X	64092274	64092296	-	edit	1	0	0.0%	0	0.0%	0	0.0%
mmu-mir-465a-5p	X	64092274	64092296	-	edit	2	0	0.0%	0	0.0%	0	0.0%
mmu-mir-465a-5p	X	64092274	64092296	-	edit	3	0	0.0%	0	0.0%	0	0.0%
mmu-mir-465a-5p	X	64092274	64092296	-	edit	4	0	0.0%	0	0.0%	0	0.0%
mmu-mir-465a-5p	X	64092274	64092296	-	edit	5	0	0.0%	0	0.0%	0	0.0%
mmu-mir-465a-5p	X	64092274	64092296	-	edit	6	0	0.0%	0	0.0%	0	0.0%
mmu-mir-465a-5p	X	64092274	64092296	-	edit	7	0	0.0%	0	0.0%	0	0.0%
mmu-mir-465a-5p	X	64092274	64092296	-	edit	8	0	0.0%	0	0.0%	0	0.0%
mmu-mir-465a-5p	X	64092274	64092296	-	edit	9	0	0.0%	0	0.0%	0	0.0%
mmu-mir-465a-5p	X	64092274	64092296	-	edit	10	0	0.0%	0	0.0%	0	0.0%
mmu-mir-465a-5p	X	64092274	64092296	-	edit	11	0	0.0%	0	0.0%	0	0.0%
mmu-mir-465a-5p	X	64092274	64092296	-	edit	12	0	0.0%	0	0.0%	0	0.0%
mmu-mir-465a-5p	X	64092274	64092296	-	edit	13	0	0.0%	0	0.0%	0	0.0%
mmu-mir-465a-5p	X	64092274	64092296	-	edit	14	0	0.0%	0	0.0%	0	0.0%
mmu-mir-465a-5p	X	64092274	64092296	-	edit	15	0	0.0%	0	0.0%	4	0.4%
mmu-mir-465a-5p	X	64092274	64092296	-	edit	16	0	0.0%	0	0.0%	0	0.0%
mmu-mir-465a-5p	X	64092274	64092296	-	edit	17	0	0.0%	0	0.0%	0	0.0%
mmu-mir-465a-5p	X	64092274	64092296	-	edit	18	0	0.0%	18	1.6%	0	0.0%
mmu-mir-465a-5p	X	64092274	64092296	-	edit	19	0	0.0%	0	0.0%	4	0.4%
mmu-mir-465a-5p	X	64092274	64092296	-	edit	20	0	0.0%	0	0.0%	0	0.0%
mmu-mir-465a-5p	X	64092274	64092296	-	edit	21	0	0.0%	0	0.0%	7	0.7%
mmu-mir-465a-5p	X	64092274	64092296	-	edit	22	0	0.0%	0	0.0%	0	0.0%
EDITED							0	0.0%	18	1.6%	15	1.4%
CANONICAL							488	100.0%	1095	98.4%	1028	98.6%
TOTAL							488		1113		1043	

miRNA	Chr	Start	Stop	Strand	Type	Position	P7 reads	P7%	P10 reads	P10%	P14 reads	P14%
mmu-mir-465b-1-3p	X	64082387	64082408	-	Indel	22	0	0.0%	0	0.0%	4	0.3%
mmu-mir-465b-1-3p	X	64082387	64082408	-	edit	1	0	0.0%	0	0.0%	0	0.0%
mmu-mir-465b-1-3p	X	64082387	64082408	-	edit	2	0	0.0%	0	0.0%	0	0.0%
mmu-mir-465b-1-3p	X	64082387	64082408	-	edit	3	0	0.0%	0	0.0%	0	0.0%
mmu-mir-465b-1-3p	X	64082387	64082408	-	edit	4	0	0.0%	0	0.0%	0	0.0%
mmu-mir-465b-1-3p	X	64082387	64082408	-	edit	5	0	0.0%	0	0.0%	0	0.0%
mmu-mir-465b-1-3p	X	64082387	64082408	-	edit	6	0	0.0%	0	0.0%	0	0.0%
mmu-mir-465b-1-3p	X	64082387	64082408	-	edit	7	0	0.0%	0	0.0%	0	0.0%
mmu-mir-465b-1-3p	X	64082387	64082408	-	edit	8	0	0.0%	0	0.0%	0	0.0%
mmu-mir-465b-1-3p	X	64082387	64082408	-	edit	9	0	0.0%	0	0.0%	0	0.0%
mmu-mir-465b-1-3p	X	64082387	64082408	-	edit	10	0	0.0%	0	0.0%	0	0.0%
mmu-mir-465b-1-3p	X	64082387	64082408	-	edit	11	0	0.0%	0	0.0%	0	0.0%
mmu-mir-465b-1-3p	X	64082387	64082408	-	edit	12	0	0.0%	0	0.0%	0	0.0%
mmu-mir-465b-1-3p	X	64082387	64082408	-	edit	13	0	0.0%	0	0.0%	0	0.0%
mmu-mir-465b-1-3p	X	64082387	64082408	-	edit	14	0	0.0%	0	0.0%	0	0.0%
mmu-mir-465b-1-3p	X	64082387	64082408	-	edit	15	0	0.0%	0	0.0%	0	0.0%
mmu-mir-465b-1-3p	X	64082387	64082408	-	edit	16	0	0.0%	3	0.2%	0	0.0%
mmu-mir-465b-1-3p	X	64082387	64082408	-	edit	17	0	0.0%	33	1.7%	4	0.2%
mmu-mir-465b-1-3p	X	64082387	64082408	-	edit	18	0	0.0%	0	0.0%	0	0.0%
mmu-mir-465b-1-3p	X	64082387	64082408	-	edit	19	4	0.6%	11	0.6%	15	0.9%
mmu-mir-465b-1-3p	X	64082387	64082408	-	edit	20	0	0.0%	0	0.0%	0	0.0%
mmu-mir-465b-1-3p	X	64082387	64082408	-	edit	21	0	0.0%	0	0.0%	0	0.0%
mmu-mir-465b-1-3p	X	64082387	64082408	-	edit	22	0	0.0%	0	0.0%	0	0.0%
EDITED							4	0.6%	47	2.5%	23	1.4%
CANONICAL							718	99.4%	1855	97.5%	1588	98.6%
TOTAL							722		1902		1611	

miRNA	Chr	Start	Stop	Strand	Type	Position	P7 reads	P7%	P10 reads	P10%	P14 reads	P14%
mmu-mir-465b-1-5p	X	64082424	64082445	-	edit	1	0	0.0%	0	0.0%	0	0.0%
mmu-mir-465b-1-5p	X	64082424	64082445	-	edit	2	0	0.0%	0	0.0%	0	0.0%
mmu-mir-465b-1-5p	X	64082424	64082445	-	edit	3	0	0.0%	0	0.0%	0	0.0%
mmu-mir-465b-1-5p	X	64082424	64082445	-	edit	4	0	0.0%	0	0.0%	0	0.0%
mmu-mir-465b-1-5p	X	64082424	64082445	-	edit	5	0	0.0%	0	0.0%	0	0.0%
mmu-mir-465b-1-5p	X	64082424	64082445	-	edit	6	0	0.0%	0	0.0%	0	0.0%
mmu-mir-465b-1-5p	X	64082424	64082445	-	edit	7	0	0.0%	0	0.0%	0	0.0%
mmu-mir-465b-1-5p	X	64082424	64082445	-	edit	8	0	0.0%	0	0.0%	0	0.0%
mmu-mir-465b-1-5p	X	64082424	64082445	-	edit	9	0	0.0%	0	0.0%	0	0.0%
mmu-mir-465b-1-5p	X	64082424	64082445	-	edit	10	0	0.0%	0	0.0%	0	0.0%
mmu-mir-465b-1-5p	X	64082424	64082445	-	edit	11	0	0.0%	0	0.0%	0	0.0%
mmu-mir-465b-1-5p	X	64082424	64082445	-	edit	12	0	0.0%	0	0.0%	0	0.0%
mmu-mir-465b-1-5p	X	64082424	64082445	-	edit	13	0	0.0%	0	0.0%	0	0.0%
mmu-mir-465b-1-5p	X	64082424	64082445	-	edit	14	0	0.0%	0	0.0%	0	0.0%
mmu-mir-465b-1-5p	X	64082424	64082445	-	edit	15	0	0.0%	0	0.0%	3	0.6%
mmu-mir-465b-1-5p	X	64082424	64082445	-	edit	16	0	0.0%	0	0.0%	0	0.0%
mmu-mir-465b-1-5p	X	64082424	64082445	-	edit	17	0	0.0%	0	0.0%	0	0.0%
mmu-mir-465b-1-5p	X	64082424	64082445	-	edit	18	0	0.0%	0	0.0%	0	0.0%
mmu-mir-465b-1-5p	X	64082424	64082445	-	edit	19	0	0.0%	0	0.0%	0	0.0%
mmu-mir-465b-1-5p	X	64082424	64082445	-	edit	20	0	0.0%	0	0.0%	0	0.0%
mmu-mir-465b-1-5p	X	64082424	64082445	-	edit	21	0	0.0%	0	0.0%	0	0.0%
mmu-mir-465b-1-5p	X	64082424	64082445	-	edit	22	0	0.0%	0	0.0%	0	0.0%
EDITED							0	0.0%	0	0.0%	3	0.6%
CANONICAL							234	100.0%	552	100.0%	615	99.4%
TOTAL							234		552		618	

miRNA	Chr	Start	Stop	Strand	Type	Position	P7 reads	P7%	P10 reads	P10%	P14 reads	P14%
mmu-mir-465b-2-3p	X	64088949	64088970	-	Indel	22	0	0.0%	0	0.0%	4	0.3%
mmu-mir-465b-2-3p	X	64088949	64088970	-	edit	1	0	0.0%	0	0.0%	0	0.0%
mmu-mir-465b-2-3p	X	64088949	64088970	-	edit	2	0	0.0%	0	0.0%	0	0.0%
mmu-mir-465b-2-3p	X	64088949	64088970	-	edit	3	0	0.0%	0	0.0%	0	0.0%
mmu-mir-465b-2-3p	X	64088949	64088970	-	edit	4	0	0.0%	0	0.0%	0	0.0%
mmu-mir-465b-2-3p	X	64088949	64088970	-	edit	5	0	0.0%	0	0.0%	0	0.0%
mmu-mir-465b-2-3p	X	64088949	64088970	-	edit	6	0	0.0%	0	0.0%	0	0.0%
mmu-mir-465b-2-3p	X	64088949	64088970	-	edit	7	0	0.0%	0	0.0%	0	0.0%
mmu-mir-465b-2-3p	X	64088949	64088970	-	edit	8	0	0.0%	0	0.0%	0	0.0%
mmu-mir-465b-2-3p	X	64088949	64088970	-	edit	9	0	0.0%	0	0.0%	0	0.0%
mmu-mir-465b-2-3p	X	64088949	64088970	-	edit	10	0	0.0%	0	0.0%	0	0.0%
mmu-mir-465b-2-3p	X	64088949	64088970	-	edit	11	0	0.0%	0	0.0%	0	0.0%
mmu-mir-465b-2-3p	X	64088949	64088970	-	edit	12	0	0.0%	0	0.0%	0	0.0%
mmu-mir-465b-2-3p	X	64088949	64088970	-	edit	13	0	0.0%	0	0.0%	0	0.0%
mmu-mir-465b-2-3p	X	64088949	64088970	-	edit	14	0	0.0%	0	0.0%	0	0.0%
mmu-mir-465b-2-3p	X	64088949	64088970	-	edit	15	0	0.0%	0	0.0%	0	0.0%
mmu-mir-465b-2-3p	X	64088949	64088970	-	edit	16	0	0.0%	3	0.2%	0	0.0%
mmu-mir-465b-2-3p	X	64088949	64088970	-	edit	17	0	0.0%	33	1.7%	4	0.2%
mmu-mir-465b-2-3p	X	64088949	64088970	-	edit	18	0	0.0%	0	0.0%	0	0.0%
mmu-mir-465b-2-3p	X	64088949	64088970	-	edit	19	4	0.6%	11	0.6%	15	0.9%
mmu-mir-465b-2-3p	X	64088949	64088970	-	edit	20	0	0.0%	0	0.0%	0	0.0%
mmu-mir-465b-2-3p	X	64088949	64088970	-	edit	21	0	0.0%	0	0.0%	0	0.0%
mmu-mir-465b-2-3p	X	64088949	64088970	-	edit	22	0	0.0%	0	0.0%	0	0.0%
EDITED							4	0.6%	47	2.5%	19	1.2%
CANONICAL							718	99.4%	1855	97.5%	1588	98.8%
TOTAL							722		1902		1607	

miRNA	Chr	Start	Stop	Strand	Type	Position	P7 reads	P7%	P10 reads	P10%	P14 reads	P14%
mmu-mir-465b-2-5p	X	64088986	64089007	-	edit	1	0	0.0%	0	0.0%	0	0.0%
mmu-mir-465b-2-5p	X	64088986	64089007	-	edit	2	0	0.0%	0	0.0%	0	0.0%
mmu-mir-465b-2-5p	X	64088986	64089007	-	edit	3	0	0.0%	0	0.0%	0	0.0%
mmu-mir-465b-2-5p	X	64088986	64089007	-	edit	4	0	0.0%	0	0.0%	0	0.0%
mmu-mir-465b-2-5p	X	64088986	64089007	-	edit	5	0	0.0%	0	0.0%	0	0.0%
mmu-mir-465b-2-5p	X	64088986	64089007	-	edit	6	0	0.0%	0	0.0%	0	0.0%
mmu-mir-465b-2-5p	X	64088986	64089007	-	edit	7	0	0.0%	0	0.0%	0	0.0%
mmu-mir-465b-2-5p	X	64088986	64089007	-	edit	8	0	0.0%	0	0.0%	0	0.0%
mmu-mir-465b-2-5p	X	64088986	64089007	-	edit	9	0	0.0%	0	0.0%	0	0.0%
mmu-mir-465b-2-5p	X	64088986	64089007	-	edit	10	0	0.0%	0	0.0%	0	0.0%
mmu-mir-465b-2-5p	X	64088986	64089007	-	edit	11	0	0.0%	0	0.0%	0	0.0%
mmu-mir-465b-2-5p	X	64088986	64089007	-	edit	12	0	0.0%	0	0.0%	0	0.0%
mmu-mir-465b-2-5p	X	64088986	64089007	-	edit	13	0	0.0%	0	0.0%	0	0.0%
mmu-mir-465b-2-5p	X	64088986	64089007	-	edit	14	0	0.0%	0	0.0%	0	0.0%
mmu-mir-465b-2-5p	X	64088986	64089007	-	edit	15	0	0.0%	0	0.0%	3	0.6%
mmu-mir-465b-2-5p	X	64088986	64089007	-	edit	16	0	0.0%	0	0.0%	0	0.0%
mmu-mir-465b-2-5p	X	64088986	64089007	-	edit	17	0	0.0%	0	0.0%	0	0.0%
mmu-mir-465b-2-5p	X	64088986	64089007	-	edit	18	0	0.0%	0	0.0%	0	0.0%
mmu-mir-465b-2-5p	X	64088986	64089007	-	edit	19	0	0.0%	0	0.0%	0	0.0%
mmu-mir-465b-2-5p	X	64088986	64089007	-	edit	20	0	0.0%	0	0.0%	0	0.0%
mmu-mir-465b-2-5p	X	64088986	64089007	-	edit	21	0	0.0%	0	0.0%	0	0.0%
mmu-mir-465b-2-5p	X	64088986	64089007	-	edit	22	0	0.0%	0	0.0%	0	0.0%
EDITED							0	0.0%	0	0.0%	3	0.6%
CANONICAL							234	100.0%	552	100.0%	615	99.4%
TOTAL							234		552		618	

miRNA	Chr	Start	Stop	Strand	Type	Position	P7 reads	P7%	P10 reads	P10%	P14 reads	P14%
mmu-mir-465c-1-3p	X	64079140	64079161	-	Indel	22	0	0.0%	0	0.0%	4	0.3%
mmu-mir-465c-1-3p	X	64079140	64079161	-	edit	1	0	0.0%	0	0.0%	0	0.0%
mmu-mir-465c-1-3p	X	64079140	64079161	-	edit	2	0	0.0%	0	0.0%	0	0.0%
mmu-mir-465c-1-3p	X	64079140	64079161	-	edit	3	0	0.0%	0	0.0%	0	0.0%
mmu-mir-465c-1-3p	X	64079140	64079161	-	edit	4	0	0.0%	0	0.0%	0	0.0%
mmu-mir-465c-1-3p	X	64079140	64079161	-	edit	5	0	0.0%	0	0.0%	0	0.0%
mmu-mir-465c-1-3p	X	64079140	64079161	-	edit	6	0	0.0%	0	0.0%	0	0.0%
mmu-mir-465c-1-3p	X	64079140	64079161	-	edit	7	0	0.0%	0	0.0%	0	0.0%
mmu-mir-465c-1-3p	X	64079140	64079161	-	edit	8	0	0.0%	0	0.0%	0	0.0%
mmu-mir-465c-1-3p	X	64079140	64079161	-	edit	9	0	0.0%	0	0.0%	0	0.0%
mmu-mir-465c-1-3p	X	64079140	64079161	-	edit	10	0	0.0%	0	0.0%	0	0.0%
mmu-mir-465c-1-3p	X	64079140	64079161	-	edit	11	0	0.0%	0	0.0%	0	0.0%
mmu-mir-465c-1-3p	X	64079140	64079161	-	edit	12	0	0.0%	0	0.0%	0	0.0%
mmu-mir-465c-1-3p	X	64079140	64079161	-	edit	13	0	0.0%	0	0.0%	0	0.0%
mmu-mir-465c-1-3p	X	64079140	64079161	-	edit	14	0	0.0%	0	0.0%	0	0.0%
mmu-mir-465c-1-3p	X	64079140	64079161	-	edit	15	0	0.0%	0	0.0%	0	0.0%
mmu-mir-465c-1-3p	X	64079140	64079161	-	edit	16	0	0.0%	3	0.2%	0	0.0%
mmu-mir-465c-1-3p	X	64079140	64079161	-	edit	17	0	0.0%	33	1.7%	4	0.2%
mmu-mir-465c-1-3p	X	64079140	64079161	-	edit	18	0	0.0%	0	0.0%	0	0.0%
mmu-mir-465c-1-3p	X	64079140	64079161	-	edit	19	4	0.6%	11	0.6%	15	0.9%
mmu-mir-465c-1-3p	X	64079140	64079161	-	edit	20	0	0.0%	0	0.0%	0	0.0%
mmu-mir-465c-1-3p	X	64079140	64079161	-	edit	21	0	0.0%	0	0.0%	0	0.0%
mmu-mir-465c-1-3p	X	64079140	64079161	-	edit	22	0	0.0%	0	0.0%	0	0.0%
EDITED							4	0.6%	47	2.5%	23	1.4%
CANONICAL							717	99.4%	1856	97.5%	1587	98.6%
TOTAL							721		1903		1610	

miRNA	Chr	Start	Stop	Strand	Type	Position	P7 reads	P7%	P10 reads	P10%	P14 reads	P14%
mmu-mir-465c-1-5p	X	64079179	64079200	-	5' Edit	5'	0	0.0%	0	0.0%	6	0.2%
mmu-mir-465c-1-5p	X	64079179	64079200	-	edit	1	0	0.0%	0	0.0%	0	0.0%
mmu-mir-465c-1-5p	X	64079179	64079200	-	edit	2	0	0.0%	0	0.0%	0	0.0%
mmu-mir-465c-1-5p	X	64079179	64079200	-	edit	3	0	0.0%	0	0.0%	0	0.0%
mmu-mir-465c-1-5p	X	64079179	64079200	-	edit	4	0	0.0%	0	0.0%	0	0.0%
mmu-mir-465c-1-5p	X	64079179	64079200	-	edit	5	0	0.0%	0	0.0%	0	0.0%
mmu-mir-465c-1-5p	X	64079179	64079200	-	edit	6	0	0.0%	0	0.0%	0	0.0%
mmu-mir-465c-1-5p	X	64079179	64079200	-	edit	7	0	0.0%	0	0.0%	0	0.0%
mmu-mir-465c-1-5p	X	64079179	64079200	-	edit	8	0	0.0%	0	0.0%	0	0.0%
mmu-mir-465c-1-5p	X	64079179	64079200	-	edit	9	0	0.0%	0	0.0%	0	0.0%
mmu-mir-465c-1-5p	X	64079179	64079200	-	edit	10	0	0.0%	0	0.0%	0	0.0%
mmu-mir-465c-1-5p	X	64079179	64079200	-	edit	11	0	0.0%	0	0.0%	0	0.0%
mmu-mir-465c-1-5p	X	64079179	64079200	-	edit	12	0	0.0%	0	0.0%	0	0.0%
mmu-mir-465c-1-5p	X	64079179	64079200	-	edit	13	0	0.0%	0	0.0%	0	0.0%
mmu-mir-465c-1-5p	X	64079179	64079200	-	edit	14	0	0.0%	0	0.0%	0	0.0%
mmu-mir-465c-1-5p	X	64079179	64079200	-	edit	15	5	0.4%	23	0.7%	24	0.9%
mmu-mir-465c-1-5p	X	64079179	64079200	-	edit	16	0	0.0%	0	0.0%	3	0.1%
mmu-mir-465c-1-5p	X	64079179	64079200	-	edit	17	0	0.0%	0	0.0%	0	0.0%
mmu-mir-465c-1-5p	X	64079179	64079200	-	edit	18	0	0.0%	0	0.0%	0	0.0%
mmu-mir-465c-1-5p	X	64079179	64079200	-	edit	19	0	0.0%	3	0.1%	12	0.4%
mmu-mir-465c-1-5p	X	64079179	64079200	-	edit	20	0	0.0%	0	0.0%	0	0.0%
mmu-mir-465c-1-5p	X	64079179	64079200	-	edit	21	0	0.0%	0	0.0%	0	0.0%
mmu-mir-465c-1-5p	X	64079179	64079200	-	edit	22	0	0.0%	0	0.0%	0	0.0%
EDITED							5	0.4%	27	0.8%	45	1.6%
CANONICAL							1229	99.6%	3357	99.2%	2724	98.4%
TOTAL							1234		3384		2769	

miRNA	Chr	Start	Stop	Strand	Type	Position	P7 reads	P7%	P10 reads	P10%	P14 reads	P14%
mmu-mir-465c-2-3p	X	64085702	64085723	-	Indel	22	0	0.0%	0	0.0%	4	0.3%
mmu-mir-465c-2-3p	X	64085702	64085723	-	edit	1	0	0.0%	0	0.0%	0	0.0%
mmu-mir-465c-2-3p	X	64085702	64085723	-	edit	2	0	0.0%	0	0.0%	0	0.0%
mmu-mir-465c-2-3p	X	64085702	64085723	-	edit	3	0	0.0%	0	0.0%	0	0.0%
mmu-mir-465c-2-3p	X	64085702	64085723	-	edit	4	0	0.0%	0	0.0%	0	0.0%
mmu-mir-465c-2-3p	X	64085702	64085723	-	edit	5	0	0.0%	0	0.0%	0	0.0%
mmu-mir-465c-2-3p	X	64085702	64085723	-	edit	6	0	0.0%	0	0.0%	0	0.0%
mmu-mir-465c-2-3p	X	64085702	64085723	-	edit	7	0	0.0%	0	0.0%	0	0.0%
mmu-mir-465c-2-3p	X	64085702	64085723	-	edit	8	0	0.0%	0	0.0%	0	0.0%
mmu-mir-465c-2-3p	X	64085702	64085723	-	edit	9	0	0.0%	0	0.0%	0	0.0%
mmu-mir-465c-2-3p	X	64085702	64085723	-	edit	10	0	0.0%	0	0.0%	0	0.0%
mmu-mir-465c-2-3p	X	64085702	64085723	-	edit	11	0	0.0%	0	0.0%	0	0.0%
mmu-mir-465c-2-3p	X	64085702	64085723	-	edit	12	0	0.0%	0	0.0%	0	0.0%
mmu-mir-465c-2-3p	X	64085702	64085723	-	edit	13	0	0.0%	0	0.0%	0	0.0%
mmu-mir-465c-2-3p	X	64085702	64085723	-	edit	14	0	0.0%	0	0.0%	0	0.0%
mmu-mir-465c-2-3p	X	64085702	64085723	-	edit	15	0	0.0%	0	0.0%	0	0.0%
mmu-mir-465c-2-3p	X	64085702	64085723	-	edit	16	0	0.0%	3	0.2%	0	0.0%
mmu-mir-465c-2-3p	X	64085702	64085723	-	edit	17	0	0.0%	33	1.7%	4	0.2%
mmu-mir-465c-2-3p	X	64085702	64085723	-	edit	18	0	0.0%		0.0%	0	0.0%
mmu-mir-465c-2-3p	X	64085702	64085723	-	edit	19	4	0.6%	11	0.6%	15	0.9%
mmu-mir-465c-2-3p	X	64085702	64085723	-	edit	20	0	0.0%	0	0.0%	0	0.0%
mmu-mir-465c-2-3p	X	64085702	64085723	-	edit	21	0	0.0%	0	0.0%	0	0.0%
mmu-mir-465c-2-3p	X	64085702	64085723	-	edit	22	0	0.0%	0	0.0%	0	0.0%
EDITED							4	0.6%	47	2.5%	23	1.4%
CANONICAL							717	99.4%	1856	97.5%	1587	98.6%
TOTAL							721		1903		1610	

miRNA	Chr	Start	Stop	Strand	Type	Position	P7 reads	P7%	P10 reads	P10%	P14 reads	P14%
mmu-mir-465c-2-5p	X	64085741	64085762	-	5' Edit	5'	0	0.0%	0	0.0%	6	0.2%
mmu-mir-465c-2-5p	X	64085741	64085762	-	edit	1	0	0.0%	0	0.0%	0	0.0%
mmu-mir-465c-2-5p	X	64085741	64085762	-	edit	2	0	0.0%	0	0.0%	0	0.0%
mmu-mir-465c-2-5p	X	64085741	64085762	-	edit	3	0	0.0%	0	0.0%	0	0.0%
mmu-mir-465c-2-5p	X	64085741	64085762	-	edit	4	0	0.0%	0	0.0%	0	0.0%
mmu-mir-465c-2-5p	X	64085741	64085762	-	edit	5	0	0.0%	0	0.0%	0	0.0%
mmu-mir-465c-2-5p	X	64085741	64085762	-	edit	6	0	0.0%	0	0.0%	0	0.0%
mmu-mir-465c-2-5p	X	64085741	64085762	-	edit	7	0	0.0%	0	0.0%	0	0.0%
mmu-mir-465c-2-5p	X	64085741	64085762	-	edit	8	0	0.0%	0	0.0%	0	0.0%
mmu-mir-465c-2-5p	X	64085741	64085762	-	edit	9	0	0.0%	0	0.0%	0	0.0%
mmu-mir-465c-2-5p	X	64085741	64085762	-	edit	10	0	0.0%	0	0.0%	0	0.0%
mmu-mir-465c-2-5p	X	64085741	64085762	-	edit	11	0	0.0%	0	0.0%	0	0.0%
mmu-mir-465c-2-5p	X	64085741	64085762	-	edit	12	0	0.0%	0	0.0%	0	0.0%
mmu-mir-465c-2-5p	X	64085741	64085762	-	edit	13	0	0.0%	0	0.0%	0	0.0%
mmu-mir-465c-2-5p	X	64085741	64085762	-	edit	14	0	0.0%	0	0.0%	0	0.0%
mmu-mir-465c-2-5p	X	64085741	64085762	-	edit	15	5	0.4%	23	0.7%	24	0.9%
mmu-mir-465c-2-5p	X	64085741	64085762	-	edit	16	0	0.0%	0	0.0%	3	0.1%
mmu-mir-465c-2-5p	X	64085741	64085762	-	edit	17	0	0.0%	0	0.0%	0	0.0%
mmu-mir-465c-2-5p	X	64085741	64085762	-	edit	18	0	0.0%	0	0.0%	0	0.0%
mmu-mir-465c-2-5p	X	64085741	64085762	-	edit	19	0	0.0%	3	0.1%	12	0.4%
mmu-mir-465c-2-5p	X	64085741	64085762	-	edit	20	0	0.0%	0	0.0%	0	0.0%
mmu-mir-465c-2-5p	X	64085741	64085762	-	edit	21	0	0.0%	0	0.0%	0	0.0%
mmu-mir-465c-2-5p	X	64085741	64085762	-	edit	22	0	0.0%	0	0.0%	0	0.0%
EDITED							5	0.4%	27	0.8%	45	1.6%
CANONICAL							1229	99.6%	3357	99.2%	2724	98.4%
TOTAL							1234		3384		2769	

miRNA	Chr	Start	Stop	Strand	Type	Position	P7 reads	P7%	P10 reads	P10%	P14 reads	P14%
mmu-mir-470-5p	X	64067170	64067192	-	edit	1	0	0.0%	0	0.0%	0	0.0%
mmu-mir-470-5p	X	64067170	64067192	-	edit	2	0	0.0%	0	0.0%	0	0.0%
mmu-mir-470-5p	X	64067170	64067192	-	edit	3	0	0.0%	0	0.0%	0	0.0%
mmu-mir-470-5p	X	64067170	64067192	-	edit	4	0	0.0%	0	0.0%	0	0.0%
mmu-mir-470-5p	X	64067170	64067192	-	edit	5	0	0.0%	0	0.0%	0	0.0%
mmu-mir-470-5p	X	64067170	64067192	-	edit	6	0	0.0%	0	0.0%	0	0.0%
mmu-mir-470-5p	X	64067170	64067192	-	edit	7	0	0.0%	0	0.0%	0	0.0%
mmu-mir-470-5p	X	64067170	64067192	-	edit	8	0	0.0%	0	0.0%	0	0.0%
mmu-mir-470-5p	X	64067170	64067192	-	edit	9	0	0.0%	3	0.1%	8	0.1%
mmu-mir-470-5p	X	64067170	64067192	-	edit	10	0	0.0%	0	0.0%	0	0.0%
mmu-mir-470-5p	X	64067170	64067192	-	edit	11	0	0.0%	0	0.0%	0	0.0%
mmu-mir-470-5p	X	64067170	64067192	-	edit	12	0	0.0%	0	0.0%	0	0.0%
mmu-mir-470-5p	X	64067170	64067192	-	edit	13	0	0.0%	0	0.0%	4	0.1%
mmu-mir-470-5p	X	64067170	64067192	-	edit	14	0	0.0%	0	0.0%	0	0.0%
mmu-mir-470-5p	X	64067170	64067192	-	edit	15	3	0.1%	11	0.2%	11	0.2%
mmu-mir-470-5p	X	64067170	64067192	-	edit	16	3	0.1%	3	0.1%	4	0.1%
mmu-mir-470-5p	X	64067170	64067192	-	edit	17	0	0.0%	0	0.0%	0	0.0%
mmu-mir-470-5p	X	64067170	64067192	-	edit	18	0	0.0%	10	0.2%	3	0.0%
mmu-mir-470-5p	X	64067170	64067192	-	edit	19	22	1.1%	47	0.8%	123	1.9%
mmu-mir-470-5p	X	64067170	64067192	-	edit	20	0	0.0%	6	0.1%	5	0.1%
mmu-mir-470-5p	X	64067170	64067192	-	edit	21	9	0.4%	51	0.9%	46	0.7%
mmu-mir-470-5p	X	64067170	64067192	-	edit	22	0	0.0%	0	0.0%	5	0.1%
mmu-mir-470-5p	X	64067170	64067192	-	edit	23	0	0.0%	0	0.0%	5	0.1%
EDITED							37	1.8%	131	2.2%	213	3.3%
CANONICAL							2090	98.2%	5744	97.8%	6308	96.7%
TOTAL							2127		5875		6521	

miRNA	Chr	Start	Stop	Strand	Type	Position	P7 reads	P7%	P10 reads	P10%	P14 reads	P14%
mmu-mir-483-5p	7	149840873	149840894	-	edit	1	0	0.0%	0	0.0%	0	0.0%
mmu-mir-483-5p	7	149840873	149840894	-	edit	2	0	0.0%	0	0.0%	0	0.0%
mmu-mir-483-5p	7	149840873	149840894	-	edit	3	0	0.0%	0	0.0%	0	0.0%
mmu-mir-483-5p	7	149840873	149840894	-	edit	4	0	0.0%	0	0.0%	0	0.0%
mmu-mir-483-5p	7	149840873	149840894	-	edit	5	0	0.0%	0	0.0%	0	0.0%
mmu-mir-483-5p	7	149840873	149840894	-	edit	6	0	0.0%	0	0.0%	0	0.0%
mmu-mir-483-5p	7	149840873	149840894	-	edit	7	0	0.0%	0	0.0%	0	0.0%
mmu-mir-483-5p	7	149840873	149840894	-	edit	8	0	0.0%	0	0.0%	0	0.0%
mmu-mir-483-5p	7	149840873	149840894	-	edit	9	0	0.0%	0	0.0%	0	0.0%
mmu-mir-483-5p	7	149840873	149840894	-	edit	10	0	0.0%	0	0.0%	0	0.0%
mmu-mir-483-5p	7	149840873	149840894	-	edit	11	0	0.0%	0	0.0%	0	0.0%
mmu-mir-483-5p	7	149840873	149840894	-	edit	12	0	0.0%	0	0.0%	0	0.0%
mmu-mir-483-5p	7	149840873	149840894	-	edit	13	0	0.0%	0	0.0%	0	0.0%
mmu-mir-483-5p	7	149840873	149840894	-	edit	14	0	0.0%	0	0.0%	0	0.0%
mmu-mir-483-5p	7	149840873	149840894	-	edit	15	0	0.0%	0	0.0%	0	0.0%
mmu-mir-483-5p	7	149840873	149840894	-	edit	16	0	0.0%	0	0.0%	0	0.0%
mmu-mir-483-5p	7	149840873	149840894	-	edit	17	0	0.0%	0	0.0%	0	0.0%
mmu-mir-483-5p	7	149840873	149840894	-	edit	18	0	0.0%	0	0.0%	0	0.0%
mmu-mir-483-5p	7	149840873	149840894	-	edit	19	0	0.0%	0	0.0%	0	0.0%
mmu-mir-483-5p	7	149840873	149840894	-	edit	20	0	0.0%	0	0.0%	0	0.0%
mmu-mir-483-5p	7	149840873	149840894	-	edit	21	0	0.0%	0	0.0%	0	0.0%
mmu-mir-483-5p	7	149840873	149840894	-	edit	22	4	0.6%	0	0.0%	0	0.0%
EDITED							4	0.6%	0	0.0%	0	0.0%
CANONICAL							646	99.4%	112	100.0%	66	100.0%
TOTAL							650		112		66	

miRNA	Chr	Start	Stop	Strand	Type	Position	P7 reads	P7%	P10 reads	P10%	P14 reads	P14%
mmu-mir-485-3p	12	110973156	110973177	+	edit	1	0	0.0%	0	0.0%	0	0.0%
mmu-mir-485-3p	12	110973156	110973177	+	edit	2	0	0.0%	0	0.0%	0	0.0%
mmu-mir-485-3p	12	110973156	110973177	+	edit	3	0	0.0%	0	0.0%	0	0.0%
mmu-mir-485-3p	12	110973156	110973177	+	edit	4	0	0.0%	0	0.0%	0	0.0%
mmu-mir-485-3p	12	110973156	110973177	+	edit	5	0	0.0%	0	0.0%	0	0.0%
mmu-mir-485-3p	12	110973156	110973177	+	edit	6	0	0.0%	0	0.0%	0	0.0%
mmu-mir-485-3p	12	110973156	110973177	+	edit	7	0	0.0%	0	0.0%	0	0.0%
mmu-mir-485-3p	12	110973156	110973177	+	edit	8	0	0.0%	0	0.0%	0	0.0%
mmu-mir-485-3p	12	110973156	110973177	+	edit	9	0	0.0%	0	0.0%	0	0.0%
mmu-mir-485-3p	12	110973156	110973177	+	edit	10	0	0.0%	0	0.0%	0	0.0%
mmu-mir-485-3p	12	110973156	110973177	+	edit	11	0	0.0%	0	0.0%	0	0.0%
mmu-mir-485-3p	12	110973156	110973177	+	edit	12	0	0.0%	0	0.0%	0	0.0%
mmu-mir-485-3p	12	110973156	110973177	+	edit	13	0	0.0%	0	0.0%	0	0.0%
mmu-mir-485-3p	12	110973156	110973177	+	edit	14	0	0.0%	0	0.0%	0	0.0%
mmu-mir-485-3p	12	110973156	110973177	+	edit	15	0	0.0%	0	0.0%	0	0.0%
mmu-mir-485-3p	12	110973156	110973177	+	edit	16	5	0.4%	0	0.0%	0	0.0%
mmu-mir-485-3p	12	110973156	110973177	+	edit	17	0	0.0%	0	0.0%	0	0.0%
mmu-mir-485-3p	12	110973156	110973177	+	edit	18	0	0.0%	0	0.0%	0	0.0%
mmu-mir-485-3p	12	110973156	110973177	+	edit	19	3	0.2%	0	0.0%	0	0.0%
mmu-mir-485-3p	12	110973156	110973177	+	edit	20	5	0.4%	0	0.0%	0	0.0%
mmu-mir-485-3p	12	110973156	110973177	+	edit	21	0	0.0%	0	0.0%	0	0.0%
mmu-mir-485-3p	12	110973156	110973177	+	edit	22	4	0.3%	0	0.0%	0	0.0%
EDITED							17	1.3%	0	0.0%	0	0.0%
CANONICAL							1266	98.7%	254	100.0%	113	100.0%
TOTAL							1283		254		113	

miRNA	Chr	Start	Stop	Strand	Type	Position	P7 reads	P7%	P10 reads	P10%	P14 reads	P14%
mmu-mir-485-5p	12	110973120	110973141	+	edit	1	0	0.0%	0	0.0%	0	0.0%
mmu-mir-485-5p	12	110973120	110973141	+	edit	2	0	0.0%	0	0.0%	0	0.0%
mmu-mir-485-5p	12	110973120	110973141	+	edit	3	0	0.0%	0	0.0%	0	0.0%
mmu-mir-485-5p	12	110973120	110973141	+	edit	4	0	0.0%	0	0.0%	0	0.0%
mmu-mir-485-5p	12	110973120	110973141	+	edit	5	0	0.0%	0	0.0%	0	0.0%
mmu-mir-485-5p	12	110973120	110973141	+	edit	6	0	0.0%	0	0.0%	0	0.0%
mmu-mir-485-5p	12	110973120	110973141	+	edit	7	0	0.0%	0	0.0%	0	0.0%
mmu-mir-485-5p	12	110973120	110973141	+	edit	8	0	0.0%	0	0.0%	0	0.0%
mmu-mir-485-5p	12	110973120	110973141	+	edit	9	0	0.0%	0	0.0%	0	0.0%
mmu-mir-485-5p	12	110973120	110973141	+	edit	10	0	0.0%	0	0.0%	0	0.0%
mmu-mir-485-5p	12	110973120	110973141	+	edit	11	0	0.0%	0	0.0%	0	0.0%
mmu-mir-485-5p	12	110973120	110973141	+	edit	12	0	0.0%	0	0.0%	0	0.0%
mmu-mir-485-5p	12	110973120	110973141	+	edit	13	21	0.8%	0	0.0%	0	0.0%
mmu-mir-485-5p	12	110973120	110973141	+	edit	14	0	0.0%	0	0.0%	0	0.0%
mmu-mir-485-5p	12	110973120	110973141	+	edit	15	**73**	**2.8%**	**72**	**10.4%**	**13**	**4.8%**
mmu-mir-485-5p	12	110973120	110973141	+	edit	16	0	0.0%	0	0.0%	0	0.0%
mmu-mir-485-5p	12	110973120	110973141	+	edit	17	0	0.0%	0	0.0%	0	0.0%
mmu-mir-485-5p	12	110973120	110973141	+	edit	18	0	0.0%	0	0.0%	0	0.0%
mmu-mir-485-5p	12	110973120	110973141	+	edit	19	12	0.4%	15	2.2%	0	0.0%
mmu-mir-485-5p	12	110973120	110973141	+	edit	20	0	0.0%	0	0.0%	0	0.0%
mmu-mir-485-5p	12	110973120	110973141	+	edit	21	0	0.0%	0	0.0%	0	0.0%
mmu-mir-485-5p	12	110973120	110973141	+	edit	22	0	0.0%	0	0.0%	0	0.0%
EDITED							105	4.0%	87	12.5%	13	4.8%
CANONICAL							2492	96.0%	608	87.5%	259	95.2%
TOTAL							2597		695		272	

miRNA	Chr	Start	Stop	Strand	Type	Position	P7 reads	P7%	P10 reads	P10%	P14 reads	P14%
mmu-mir-503-5p	X	50407204	50407226	-	5' Edit	5'	20	0.2%	0	0.0%	0	0.0%
mmu-mir-503-5p	X	50407204	50407226	-	5' Indel	5'	18	0.2%	0	0.0%	9	0.6%
mmu-mir-503-5p	X	50407204	50407226	-	edit	1	0	0.0%	0	0.0%	0	0.0%
mmu-mir-503-5p	X	50407204	50407226	-	edit	2	0	0.0%	0	0.0%	0	0.0%
mmu-mir-503-5p	X	50407204	50407226	-	edit	3	0	0.0%	0	0.0%	0	0.0%
mmu-mir-503-5p	X	50407204	50407226	-	edit	4	3	0.0%	0	0.0%	0	0.0%
mmu-mir-503-5p	X	50407204	50407226	-	edit	5	0	0.0%	0	0.0%	0	0.0%
mmu-mir-503-5p	X	50407204	50407226	-	edit	6	0	0.0%	0	0.0%	0	0.0%
mmu-mir-503-5p	X	50407204	50407226	-	edit	7	0	0.0%	0	0.0%	0	0.0%
mmu-mir-503-5p	X	50407204	50407226	-	edit	8	6	0.1%	0	0.0%	0	0.0%
mmu-mir-503-5p	X	50407204	50407226	-	edit	9	4	0.0%	0	0.0%	0	0.0%
mmu-mir-503-5p	X	50407204	50407226	-	edit	10	0	0.0%	0	0.0%	0	0.0%
mmu-mir-503-5p	X	50407204	50407226	-	edit	11	0	0.0%	0	0.0%	0	0.0%
mmu-mir-503-5p	X	50407204	50407226	-	edit	12	0	0.0%	0	0.0%	0	0.0%
mmu-mir-503-5p	X	50407204	50407226	-	edit	13	31	0.3%	10	0.2%	7	0.5%
mmu-mir-503-5p	X	50407204	50407226	-	edit	14	0	0.0%	0	0.0%	0	0.0%
mmu-mir-503-5p	X	50407204	50407226	-	edit	15	9	0.1%	5	0.1%	0	0.0%
mmu-mir-503-5p	X	50407204	50407226	-	edit	16	149	1.4%	80	1.7%	22	1.5%
mmu-mir-503-5p	X	50407204	50407226	-	edit	17	3	0.0%	20	0.4%	0	0.0%
mmu-mir-503-5p	X	50407204	50407226	-	edit	18	88	0.8%	58	1.2%	24	1.6%
mmu-mir-503-5p	X	50407204	50407226	-	edit	19	139	1.3%	53	1.1%	30	2.0%
mmu-mir-503-5p	X	50407204	50407226	-	edit	20	5	0.0%	3	0.1%	0	0.0%
mmu-mir-503-5p	X	50407204	50407226	-	edit	21	50	0.5%	26	0.5%	16	1.1%
mmu-mir-503-5p	X	50407204	50407226	-	edit	22	0	0.0%	0	0.0%	0	0.0%
EDITED							525	4.8%	254	5.3%	108	7.3%
CANONICAL							10470	95.2%	4502	94.7%	1373	92.7%
TOTAL							10995		4756		1481	

miRNA	Chr	Start	Stop	Strand	Type	Position	P7 reads	P7%	P10 reads	P10%	P14 reads	P14%
mmu-mir-532-5p	X	6825582	6825603	-	edit	1	0	0.0%	0	0.0%	0	0.0%
mmu-mir-532-5p	X	6825582	6825603	-	edit	2	0	0.0%	0	0.0%	0	0.0%
mmu-mir-532-5p	X	6825582	6825603	-	edit	3	0	0.0%	0	0.0%	0	0.0%
mmu-mir-532-5p	X	6825582	6825603	-	edit	4	0	0.0%	0	0.0%	0	0.0%
mmu-mir-532-5p	X	6825582	6825603	-	edit	5	0	0.0%	0	0.0%	0	0.0%
mmu-mir-532-5p	X	6825582	6825603	-	edit	6	0	0.0%	0	0.0%	0	0.0%
mmu-mir-532-5p	X	6825582	6825603	-	edit	7	0	0.0%	0	0.0%	0	0.0%
mmu-mir-532-5p	X	6825582	6825603	-	edit	8	0	0.0%	0	0.0%	0	0.0%
mmu-mir-532-5p	X	6825582	6825603	-	edit	9	0	0.0%	0	0.0%	0	0.0%
mmu-mir-532-5p	X	6825582	6825603	-	edit	10	0	0.0%	0	0.0%	0	0.0%
mmu-mir-532-5p	X	6825582	6825603	-	edit	11	0	0.0%	0	0.0%	0	0.0%
mmu-mir-532-5p	X	6825582	6825603	-	edit	12	0	0.0%	0	0.0%	0	0.0%
mmu-mir-532-5p	X	6825582	6825603	-	edit	13	0	0.0%	0	0.0%	0	0.0%
mmu-mir-532-5p	X	6825582	6825603	-	edit	14	0	0.0%	0	0.0%	0	0.0%
mmu-mir-532-5p	X	6825582	6825603	-	edit	15	0	0.0%	0	0.0%	0	0.0%
mmu-mir-532-5p	X	6825582	6825603	-	edit	16	0	0.0%	0	0.0%	0	0.0%
mmu-mir-532-5p	X	6825582	6825603	-	edit	17	0	0.0%	0	0.0%	0	0.0%
mmu-mir-532-5p	X	6825582	6825603	-	edit	18	0	0.0%	0	0.0%	0	0.0%
mmu-mir-532-5p	X	6825582	6825603	-	edit	19	**48**	**6.3%**	**39**	**10.8%**	**52**	**13.8%**
mmu-mir-532-5p	X	6825582	6825603	-	edit	20	0	0.0%	0	0.0%	0	0.0%
mmu-mir-532-5p	X	6825582	6825603	-	edit	21	0	0.0%	0	0.0%	0	0.0%
mmu-mir-532-5p	X	6825582	6825603	-	edit	22	0	0.0%	0	0.0%	0	0.0%
EDITED							48	6.3%	39	10.8%	52	13.8%
CANONICAL							712	93.7%	323	89.2%	325	86.2%
TOTAL							760		362		377	

miRNA	Chr	Start	Stop	Strand	Type	Position	P7 reads	P7%	P10 reads	P10%	P14 reads	P14%
mmu-mir-541-5p	12	110980632	110980656	+	edit	1	0	0.0%	0	0.0%	0	0.0%
mmu-mir-541-5p	12	110980632	110980656	+	edit	2	0	0.0%	0	0.0%	0	0.0%
mmu-mir-541-5p	12	110980632	110980656	+	edit	3	0	0.0%	0	0.0%	0	0.0%
mmu-mir-541-5p	12	110980632	110980656	+	edit	4	0	0.0%	0	0.0%	0	0.0%
mmu-mir-541-5p	12	110980632	110980656	+	edit	5	0	0.0%	0	0.0%	0	0.0%
mmu-mir-541-5p	12	110980632	110980656	+	edit	6	0	0.0%	0	0.0%	0	0.0%
mmu-mir-541-5p	12	110980632	110980656	+	edit	7	0	0.0%	0	0.0%	0	0.0%
mmu-mir-541-5p	12	110980632	110980656	+	edit	8	4	0.1%	0	0.0%	0	0.0%
mmu-mir-541-5p	12	110980632	110980656	+	edit	9	0	0.0%	0	0.0%	0	0.0%
mmu-mir-541-5p	12	110980632	110980656	+	edit	10	0	0.0%	0	0.0%	0	0.0%
mmu-mir-541-5p	12	110980632	110980656	+	edit	11	0	0.0%	0	0.0%	0	0.0%
mmu-mir-541-5p	12	110980632	110980656	+	edit	12	0	0.0%	0	0.0%	0	0.0%
mmu-mir-541-5p	12	110980632	110980656	+	edit	13	0	0.0%	0	0.0%	0	0.0%
mmu-mir-541-5p	12	110980632	110980656	+	edit	14	0	0.0%	0	0.0%	0	0.0%
mmu-mir-541-5p	12	110980632	110980656	+	edit	15	25	0.4%	0	0.0%	4	0.6%
mmu-mir-541-5p	12	110980632	110980656	+	edit	16	0	0.0%	0	0.0%	0	0.0%
mmu-mir-541-5p	12	110980632	110980656	+	edit	17	0	0.0%	0	0.0%	0	0.0%
mmu-mir-541-5p	12	110980632	110980656	+	edit	18	0	0.0%	0	0.0%	0	0.0%
mmu-mir-541-5p	12	110980632	110980656	+	edit	19	44	0.8%	9	0.6%	5	0.7%
mmu-mir-541-5p	12	110980632	110980656	+	edit	20	6	0.1%	0	0.0%	0	0.0%
mmu-mir-541-5p	12	110980632	110980656	+	edit	21	5	0.1%	11	0.7%	0	0.0%
mmu-mir-541-5p	12	110980632	110980656	+	edit	22	16	0.3%	0	0.0%	0	0.0%
mmu-mir-541-5p	12	110980632	110980656	+	edit	23	3	0.1%	0	0.0%	0	0.0%
EDITED							102	1.8%	20	1.3%	9	1.3%
CANONICAL							5528	98.2%	1539	98.7%	716	98.7%
TOTAL							5630		1559		725	

miRNA	Chr	Start	Stop	Strand	Type	Position	P7 reads	P7%	P10 reads	P10%	P14 reads	P14%
mmu-mir-543-5p	12	110955514	110955535	+	edit	1	0	0.0%	0	0.0%	0	0.0%
mmu-mir-543-5p	12	110955514	110955535	+	edit	2	0	0.0%	0	0.0%	0	0.0%
mmu-mir-543-5p	12	110955514	110955535	+	edit	3	0	0.0%	0	0.0%	0	0.0%
mmu-mir-543-5p	12	110955514	110955535	+	edit	4	0	0.0%	0	0.0%	0	0.0%
mmu-mir-543-5p	12	110955514	110955535	+	edit	5	0	0.0%	0	0.0%	0	0.0%
mmu-mir-543-5p	12	110955514	110955535	+	edit	6	0	0.0%	0	0.0%	0	0.0%
mmu-mir-543-5p	12	110955514	110955535	+	edit	7	0	0.0%	0	0.0%	0	0.0%
mmu-mir-543-5p	12	110955514	110955535	+	edit	8	0	0.0%	0	0.0%	0	0.0%
mmu-mir-543-5p	12	110955514	110955535	+	edit	9	0	0.0%	0	0.0%	0	0.0%
mmu-mir-543-5p	12	110955514	110955535	+	edit	10	0	0.0%	0	0.0%	0	0.0%
mmu-mir-543-5p	12	110955514	110955535	+	edit	11	0	0.0%	0	0.0%	0	0.0%
mmu-mir-543-5p	12	110955514	110955535	+	edit	12	0	0.0%	0	0.0%	0	0.0%
mmu-mir-543-5p	12	110955514	110955535	+	edit	13	0	0.0%	0	0.0%	0	0.0%
mmu-mir-543-5p	12	110955514	110955535	+	edit	14	0	0.0%	0	0.0%	0	0.0%
mmu-mir-543-5p	12	110955514	110955535	+	edit	15	11	0.8%	0	0.0%	0	0.0%
mmu-mir-543-5p	12	110955514	110955535	+	edit	16	0	0.0%	0	0.0%	0	0.0%
mmu-mir-543-5p	12	110955514	110955535	+	edit	17	0	0.0%	0	0.0%	0	0.0%
mmu-mir-543-5p	12	110955514	110955535	+	edit	18	0	0.0%	0	0.0%	0	0.0%
mmu-mir-543-5p	12	110955514	110955535	+	edit	19	3	0.2%	0	0.0%	0	0.0%
mmu-mir-543-5p	12	110955514	110955535	+	edit	20	0	0.0%	0	0.0%	0	0.0%
mmu-mir-543-5p	12	110955514	110955535	+	edit	21	0	0.0%	0	0.0%	0	0.0%
mmu-mir-543-5p	12	110955514	110955535	+	edit	22	0	0.0%	0	0.0%	0	0.0%
EDITED							14	1.0%	0	0.0%	0	0.0%
CANONICAL							1359	99.0%	286	100.0%	91	100.0%
TOTAL							1373		286		91	

miRNA	Chr	Start	Stop	Strand	Type	Position	P7 reads	P7%	P10 reads	P10%	P14 reads	P14%
mmu-mir-598-5p	14	64346073	64346094	+	edit	1	0	0.0%	0	0.0%	0	0.0%
mmu-mir-598-5p	14	64346073	64346094	+	edit	2	0	0.0%	0	0.0%	0	0.0%
mmu-mir-598-5p	14	64346073	64346094	+	edit	3	0	0.0%	0	0.0%	0	0.0%
mmu-mir-598-5p	14	64346073	64346094	+	edit	4	0	0.0%	0	0.0%	0	0.0%
mmu-mir-598-5p	14	64346073	64346094	+	edit	5	0	0.0%	0	0.0%	0	0.0%
mmu-mir-598-5p	14	64346073	64346094	+	edit	6	0	0.0%	0	0.0%	0	0.0%
mmu-mir-598-5p	14	64346073	64346094	+	edit	7	0	0.0%	0	0.0%	0	0.0%
mmu-mir-598-5p	14	64346073	64346094	+	edit	8	0	0.0%	0	0.0%	0	0.0%
mmu-mir-598-5p	14	64346073	64346094	+	edit	9	0	0.0%	0	0.0%	0	0.0%
mmu-mir-598-5p	14	64346073	64346094	+	edit	10	0	0.0%	0	0.0%	0	0.0%
mmu-mir-598-5p	14	64346073	64346094	+	edit	11	0	0.0%	0	0.0%	0	0.0%
mmu-mir-598-5p	14	64346073	64346094	+	edit	12	0	0.0%	0	0.0%	0	0.0%
mmu-mir-598-5p	14	64346073	64346094	+	edit	13	0	0.0%	0	0.0%	0	0.0%
mmu-mir-598-5p	14	64346073	64346094	+	edit	14	0	0.0%	0	0.0%	0	0.0%
mmu-mir-598-5p	14	64346073	64346094	+	edit	15	0	0.0%	0	0.0%	4	0.7%
mmu-mir-598-5p	14	64346073	64346094	+	edit	16	0	0.0%	0	0.0%	0	0.0%
mmu-mir-598-5p	14	64346073	64346094	+	edit	17	0	0.0%	0	0.0%	0	0.0%
mmu-mir-598-5p	14	64346073	64346094	+	edit	18	0	0.0%	0	0.0%	0	0.0%
mmu-mir-598-5p	14	64346073	64346094	+	edit	19	3	0.5%	0	0.0%	0	0.0%
mmu-mir-598-5p	14	64346073	64346094	+	edit	20	0	0.0%	0	0.0%	0	0.0%
mmu-mir-598-5p	14	64346073	64346094	+	edit	21	0	0.0%	0	0.0%	0	0.0%
mmu-mir-598-5p	14	64346073	64346094	+	edit	22	0	0.0%	0	0.0%	0	0.0%
EDITED							3	0.5%	0	0.0%	4	0.7%
CANONICAL							616	99.5%	574	100.0%	575	99.3%
TOTAL							619		574		579	

miRNA	Chr	Start	Stop	Strand	Type	Position	P7 reads	P7%	P10 reads	P10%	P14 reads	P14%
mmu-mir-668-5p	12	110972984	110973007	+	edit	1	0	0.0%	0	0.0%	0	0.0%
mmu-mir-668-5p	12	110972984	110973007	+	edit	2	0	0.0%	0	0.0%	0	0.0%
mmu-mir-668-5p	12	110972984	110973007	+	edit	3	0	0.0%	0	0.0%	0	0.0%
mmu-mir-668-5p	12	110972984	110973007	+	edit	4	0	0.0%	0	0.0%	0	0.0%
mmu-mir-668-5p	12	110972984	110973007	+	edit	5	0	0.0%	0	0.0%	0	0.0%
mmu-mir-668-5p	12	110972984	110973007	+	edit	6	0	0.0%	0	0.0%	0	0.0%
mmu-mir-668-5p	12	110972984	110973007	+	edit	7	0	0.0%	0	0.0%	0	0.0%
mmu-mir-668-5p	12	110972984	110973007	+	edit	8	0	0.0%	0	0.0%	0	0.0%
mmu-mir-668-5p	12	110972984	110973007	+	edit	9	0	0.0%	0	0.0%	0	0.0%
mmu-mir-668-5p	12	110972984	110973007	+	edit	10	0	0.0%	0	0.0%	0	0.0%
mmu-mir-668-5p	12	110972984	110973007	+	edit	11	0	0.0%	0	0.0%	0	0.0%
mmu-mir-668-5p	12	110972984	110973007	+	edit	12	0	0.0%	0	0.0%	0	0.0%
mmu-mir-668-5p	12	110972984	110973007	+	edit	13	0	0.0%	0	0.0%	0	0.0%
mmu-mir-668-5p	12	110972984	110973007	+	edit	14	0	0.0%	0	0.0%	0	0.0%
mmu-mir-668-5p	12	110972984	110973007	+	edit	15	0	0.0%	0	0.0%	0	0.0%
mmu-mir-668-5p	12	110972984	110973007	+	edit	16	0	0.0%	0	0.0%	0	0.0%
mmu-mir-668-5p	12	110972984	110973007	+	edit	17	3	1.5%	3	3.1%	0	0.0%
mmu-mir-668-5p	12	110972984	110973007	+	edit	18	0	0.0%	0	0.0%	0	0.0%
mmu-mir-668-5p	12	110972984	110973007	+	edit	19	**7**	**3.5%**	**8**	**8.2%**	**0**	**0.0%**
mmu-mir-668-5p	12	110972984	110973007	+	edit	20	0	0.0%	0	0.0%	0	0.0%
mmu-mir-668-5p	12	110972984	110973007	+	edit	21	0	0.0%	0	0.0%	0	0.0%
mmu-mir-668-5p	12	110972984	110973007	+	edit	22	0	0.0%	0	0.0%	0	0.0%
EDITED							10	5.0%	11	11.3%	0	0.0%
CANONICAL							192	95.0%	86	88.7%	16	100.0%
TOTAL							202		97		16	

miRNA	Chr	Start	Stop	Strand	Type	Position	P7 reads	P7%	P10 reads	P10%	P14 reads	P14%
mmu-mir-669c-5p	2	10430946	10430967	+	3' Edit	3'	64	0.6%	0	0.0%	34	0.4%
mmu-mir-669c-5p	2	10430946	10430967	+	Indel	22	4	0.0%	9	0.1%	6	0.1%
mmu-mir-669c-5p	2	10430946	10430967	+	edit	1	0	0.0%	0	0.0%	0	0.0%
mmu-mir-669c-5p	2	10430946	10430967	+	edit	2	0	0.0%	0	0.0%	0	0.0%
mmu-mir-669c-5p	2	10430946	10430967	+	edit	3	0	0.0%	0	0.0%	0	0.0%
mmu-mir-669c-5p	2	10430946	10430967	+	edit	4	0	0.0%	0	0.0%	0	0.0%
mmu-mir-669c-5p	2	10430946	10430967	+	edit	5	0	0.0%	0	0.0%	0	0.0%
mmu-mir-669c-5p	2	10430946	10430967	+	edit	6	3	0.0%	0	0.0%	0	0.0%
mmu-mir-669c-5p	2	10430946	10430967	+	edit	7	0	0.0%	0	0.0%	0	0.0%
mmu-mir-669c-5p	2	10430946	10430967	+	edit	8	12	0.1%	11	0.1%	6	0.1%
mmu-mir-669c-5p	2	10430946	10430967	+	edit	9	0	0.0%	0	0.0%	0	0.0%
mmu-mir-669c-5p	2	10430946	10430967	+	edit	10	11	0.1%	0	0.0%	0	0.0%
mmu-mir-669c-5p	2	10430946	10430967	+	edit	11	0	0.0%	0	0.0%	0	0.0%
mmu-mir-669c-5p	2	10430946	10430967	+	edit	12	21	0.2%	14	0.1%	10	0.1%
mmu-mir-669c-5p	2	10430946	10430967	+	edit	13	0	0.0%	7	0.1%	0	0.0%
mmu-mir-669c-5p	2	10430946	10430967	+	edit	14	0	0.0%	0	0.0%	0	0.0%
mmu-mir-669c-5p	2	10430946	10430967	+	edit	15	15	0.2%	72	0.6%	12	0.1%
mmu-mir-669c-5p	2	10430946	10430967	+	edit	16	0	0.0%	0	0.0%	0	0.0%
mmu-mir-669c-5p	2	10430946	10430967	+	edit	17	11	0.1%	13	0.1%	0	0.0%
mmu-mir-669c-5p	2	10430946	10430967	+	edit	18	0	0.0%	0	0.0%	0	0.0%
mmu-mir-669c-5p	2	10430946	10430967	+	edit	19	41	0.4%	19	0.2%	12	0.2%
mmu-mir-669c-5p	2	10430946	10430967	+	edit	20	19	0.2%	16	0.1%	28	0.3%
mmu-mir-669c-5p	2	10430946	10430967	+	edit	21	84	0.8%	63	0.6%	31	0.4%
mmu-mir-669c-5p	2	10430946	10430967	+	edit	22	81	0.8%	41	0.4%	93	1.1%
EDITED							364	3.7%	264	2.4%	232	2.8%
CANONICAL							9480	96.3%	10842	97.6%	7987	97.2%
TOTAL							9844		11106		8219	

miRNA	Chr	Start	Stop	Strand	Type	Position	P7 reads	P7%	P10 reads	P10%	P14 reads	P14%
mmu-mir-672-5p	X	101311567	101311589	-	5' Indel	5'	7	0.1%	0	0.0%	0	0.0%
mmu-mir-672-5p	X	101311567	101311589	-	Indel	23	21	0.3%	9	0.1%	9	0.3%
mmu-mir-672-5p	X	101311567	101311589	-	edit	1	0	0.0%	0	0.0%	0	0.0%
mmu-mir-672-5p	X	101311567	101311589	-	edit	2	0	0.0%	0	0.0%	0	0.0%
mmu-mir-672-5p	X	101311567	101311589	-	edit	3	0	0.0%	0	0.0%	0	0.0%
mmu-mir-672-5p	X	101311567	101311589	-	edit	4	0	0.0%	0	0.0%	0	0.0%
mmu-mir-672-5p	X	101311567	101311589	-	edit	5	0	0.0%	0	0.0%	0	0.0%
mmu-mir-672-5p	X	101311567	101311589	-	edit	6	0	0.0%	0	0.0%	0	0.0%
mmu-mir-672-5p	X	101311567	101311589	-	edit	7	0	0.0%	0	0.0%	0	0.0%
mmu-mir-672-5p	X	101311567	101311589	-	edit	8	3	0.0%	0	0.0%	0	0.0%
mmu-mir-672-5p	X	101311567	101311589	-	edit	9	0	0.0%	0	0.0%	0	0.0%
mmu-mir-672-5p	X	101311567	101311589	-	edit	10	0	0.0%	0	0.0%	0	0.0%
mmu-mir-672-5p	X	101311567	101311589	-	edit	11	0	0.0%	0	0.0%	0	0.0%
mmu-mir-672-5p	X	101311567	101311589	-	edit	12	0	0.0%	0	0.0%	0	0.0%
mmu-mir-672-5p	X	101311567	101311589	-	edit	13	0	0.0%	0	0.0%	0	0.0%
mmu-mir-672-5p	X	101311567	101311589	-	edit	14	0	0.0%	0	0.0%	0	0.0%
mmu-mir-672-5p	X	101311567	101311589	-	edit	15	4	0.0%	4	0.1%	4	0.1%
mmu-mir-672-5p	X	101311567	101311589	-	edit	16	6	0.1%	8	0.1%	0	0.0%
mmu-mir-672-5p	X	101311567	101311589	-	edit	17	15	0.2%	13	0.2%	12	0.3%
mmu-mir-672-5p	X	101311567	101311589	-	edit	18	6	0.1%	6	0.1%	7	0.2%
mmu-mir-672-5p	X	101311567	101311589	-	edit	19	51	0.6%	28	0.4%	17	0.5%
mmu-mir-672-5p	X	101311567	101311589	-	edit	20	9	0.1%	9	0.1%	4	0.1%
mmu-mir-672-5p	X	101311567	101311589	-	edit	21	5	0.1%	0	0.0%	0	0.0%
mmu-mir-672-5p	X	101311567	101311589	-	edit	22	18	0.2%	4	0.1%	10	0.3%
mmu-mir-672-5p	X	101311567	101311589	-	edit	23	3	0.0%	0	0.0%	0	0.0%
EDITED							147	1.8%	80	1.2%	63	1.8%
CANONICAL							7961	98.2%	6722	98.8%	3428	98.2%
TOTAL							8108		6802		3491	

miRNA	Chr	Start	Stop	Strand	Type	Position	P7 reads	P7%	P10 reads	P10%	P14 reads	P14%
mmu-mir-674-5p	2	117010887	117010908	+	edit	1	0	0.0%	0	0.0%	0	0.0%
mmu-mir-674-5p	2	117010887	117010908	+	edit	2	0	0.0%	0	0.0%	0	0.0%
mmu-mir-674-5p	2	117010887	117010908	+	edit	3	0	0.0%	0	0.0%	0	0.0%
mmu-mir-674-5p	2	117010887	117010908	+	edit	4	0	0.0%	0	0.0%	0	0.0%
mmu-mir-674-5p	2	117010887	117010908	+	edit	5	0	0.0%	0	0.0%	0	0.0%
mmu-mir-674-5p	2	117010887	117010908	+	edit	6	0	0.0%	0	0.0%	0	0.0%
mmu-mir-674-5p	2	117010887	117010908	+	edit	7	0	0.0%	0	0.0%	0	0.0%
mmu-mir-674-5p	2	117010887	117010908	+	edit	8	0	0.0%	0	0.0%	0	0.0%
mmu-mir-674-5p	2	117010887	117010908	+	edit	9	0	0.0%	0	0.0%	0	0.0%
mmu-mir-674-5p	2	117010887	117010908	+	edit	10	0	0.0%	0	0.0%	0	0.0%
mmu-mir-674-5p	2	117010887	117010908	+	edit	11	0	0.0%	0	0.0%	0	0.0%
mmu-mir-674-5p	2	117010887	117010908	+	edit	12	0	0.0%	0	0.0%	0	0.0%
mmu-mir-674-5p	2	117010887	117010908	+	edit	13	0	0.0%	0	0.0%	0	0.0%
mmu-mir-674-5p	2	117010887	117010908	+	edit	14	0	0.0%	0	0.0%	0	0.0%
mmu-mir-674-5p	2	117010887	117010908	+	edit	15	0	0.0%	0	0.0%	0	0.0%
mmu-mir-674-5p	2	117010887	117010908	+	edit	16	0	0.0%	0	0.0%	0	0.0%
mmu-mir-674-5p	2	117010887	117010908	+	edit	17	0	0.0%	0	0.0%	0	0.0%
mmu-mir-674-5p	2	117010887	117010908	+	edit	18	0	0.0%	0	0.0%	0	0.0%
mmu-mir-674-5p	2	117010887	117010908	+	edit	19	8	0.8%	0	0.0%	0	0.0%
mmu-mir-674-5p	2	117010887	117010908	+	edit	20	0	0.0%	0	0.0%	0	0.0%
mmu-mir-674-5p	2	117010887	117010908	+	edit	21	0	0.0%	0	0.0%	0	0.0%
mmu-mir-674-5p	2	117010887	117010908	+	edit	22	0	0.0%	0	0.0%	0	0.0%
EDITED							8	0.8%	0	0.0%	0	0.0%
CANONICAL							938	99.2%	296	100.0%	193	100.0%
TOTAL							946		296		193	

miRNA	Chr	Start	Stop	Strand	Type	Position	P7 reads	P7%	P10 reads	P10%	P14 reads	P14%
mmu-mir-676-3p	X	97576490	97576510	+	3' Indel	3'	7	0.6%	0	0.0%	3	0.6%
mmu-mir-676-3p	X	97576490	97576510	+	edit	1	0	0.0%	0	0.0%	0	0.0%
mmu-mir-676-3p	X	97576490	97576510	+	edit	2	0	0.0%	0	0.0%	0	0.0%
mmu-mir-676-3p	X	97576490	97576510	+	edit	3	0	0.0%	0	0.0%	0	0.0%
mmu-mir-676-3p	X	97576490	97576510	+	edit	4	0	0.0%	0	0.0%	0	0.0%
mmu-mir-676-3p	X	97576490	97576510	+	edit	5	0	0.0%	0	0.0%	0	0.0%
mmu-mir-676-3p	X	97576490	97576510	+	edit	6	0	0.0%	0	0.0%	0	0.0%
mmu-mir-676-3p	X	97576490	97576510	+	edit	7	0	0.0%	0	0.0%	0	0.0%
mmu-mir-676-3p	X	97576490	97576510	+	edit	8	0	0.0%	0	0.0%	0	0.0%
mmu-mir-676-3p	X	97576490	97576510	+	edit	9	0	0.0%	0	0.0%	0	0.0%
mmu-mir-676-3p	X	97576490	97576510	+	edit	10	0	0.0%	0	0.0%	0	0.0%
mmu-mir-676-3p	X	97576490	97576510	+	edit	11	0	0.0%	0	0.0%	0	0.0%
mmu-mir-676-3p	X	97576490	97576510	+	edit	12	0	0.0%	0	0.0%	0	0.0%
mmu-mir-676-3p	X	97576490	97576510	+	edit	13	0	0.0%	0	0.0%	0	0.0%
mmu-mir-676-3p	X	97576490	97576510	+	edit	14	0	0.0%	0	0.0%	0	0.0%
mmu-mir-676-3p	X	97576490	97576510	+	edit	15	7	0.6%	0	0.0%	4	0.7%
mmu-mir-676-3p	X	97576490	97576510	+	edit	16	0	0.0%	0	0.0%	0	0.0%
mmu-mir-676-3p	X	97576490	97576510	+	edit	17	0	0.0%	0	0.0%	0	0.0%
mmu-mir-676-3p	X	97576490	97576510	+	edit	18	0	0.0%	0	0.0%	0	0.0%
mmu-mir-676-3p	X	97576490	97576510	+	edit	19	7	0.6%	0	0.0%	0	0.0%
mmu-mir-676-3p	X	97576490	97576510	+	edit	20	0	0.0%	0	0.0%	0	0.0%
mmu-mir-676-3p	X	97576490	97576510	+	edit	21	0	0.0%	0	0.0%	0	0.0%
mmu-mir-676-3p	X	97576490	97576510	+	edit	22	0	0.0%	0	0.0%	0	0.0%
EDITED							21	1.8%	0	0.0%	7	1.4%
CANONICAL							1160	98.2%	665	100.0%	468	98.6%
TOTAL							1181		665		475	

miRNA	Chr	Start	Stop	Strand	Type	Position	P7 reads	P7%	P10 reads	P10%	P14 reads	P14%
mmu-mir-708-5p	7	103397960	103397982	+	edit	1	0	0.0%	0	0.0%	0	0.0%
mmu-mir-708-5p	7	103397960	103397982	+	edit	2	0	0.0%	0	0.0%	0	0.0%
mmu-mir-708-5p	7	103397960	103397982	+	edit	3	0	0.0%	0	0.0%	0	0.0%
mmu-mir-708-5p	7	103397960	103397982	+	edit	4	0	0.0%	0	0.0%	0	0.0%
mmu-mir-708-5p	7	103397960	103397982	+	edit	5	0	0.0%	0	0.0%	0	0.0%
mmu-mir-708-5p	7	103397960	103397982	+	edit	6	0	0.0%	0	0.0%	0	0.0%
mmu-mir-708-5p	7	103397960	103397982	+	edit	7	0	0.0%	0	0.0%	0	0.0%
mmu-mir-708-5p	7	103397960	103397982	+	edit	8	0	0.0%	0	0.0%	0	0.0%
mmu-mir-708-5p	7	103397960	103397982	+	edit	9	0	0.0%	0	0.0%	0	0.0%
mmu-mir-708-5p	7	103397960	103397982	+	edit	10	0	0.0%	0	0.0%	0	0.0%
mmu-mir-708-5p	7	103397960	103397982	+	edit	11	0	0.0%	0	0.0%	0	0.0%
mmu-mir-708-5p	7	103397960	103397982	+	edit	12	0	0.0%	0	0.0%	0	0.0%
mmu-mir-708-5p	7	103397960	103397982	+	edit	13	0	0.0%	0	0.0%	0	0.0%
mmu-mir-708-5p	7	103397960	103397982	+	edit	14	0	0.0%	0	0.0%	0	0.0%
mmu-mir-708-5p	7	103397960	103397982	+	edit	15	3	0.5%	0	0.0%	0	0.0%
mmu-mir-708-5p	7	103397960	103397982	+	edit	16	0	0.0%	0	0.0%	0	0.0%
mmu-mir-708-5p	7	103397960	103397982	+	edit	17	0	0.0%	0	0.0%	0	0.0%
mmu-mir-708-5p	7	103397960	103397982	+	edit	18	0	0.0%	0	0.0%	0	0.0%
mmu-mir-708-5p	7	103397960	103397982	+	edit	19	8	1.3%	0	0.0%	0	0.0%
mmu-mir-708-5p	7	103397960	103397982	+	edit	20	0	0.0%	0	0.0%	0	0.0%
mmu-mir-708-5p	7	103397960	103397982	+	edit	21	0	0.0%	0	0.0%	0	0.0%
mmu-mir-708-5p	7	103397960	103397982	+	edit	22	0	0.0%	0	0.0%	0	0.0%
EDITED							11	1.8%	0	0.0%	0	0.0%
CANONICAL							590	98.2%	192	100.0%	95	100.0%
TOTAL							601		192		95	

miRNA	Chr	Start	Stop	Strand	Type	Position	P7 reads	P7%	P10 reads	P10%	P14 reads	P14%
mmu-mir-742-3p	X	64033551	64033572	-	Indel	22	0	0.0%	0	0.0%	**8**	**6.8%**
mmu-mir-742-3p	X	64033551	64033572	-	edit	1	0	0.0%	0	0.0%	0	0.0%
mmu-mir-742-3p	X	64033551	64033572	-	edit	2	0	0.0%	0	0.0%	0	0.0%
mmu-mir-742-3p	X	64033551	64033572	-	edit	3	0	0.0%	0	0.0%	0	0.0%
mmu-mir-742-3p	X	64033551	64033572	-	edit	4	0	0.0%	0	0.0%	0	0.0%
mmu-mir-742-3p	X	64033551	64033572	-	edit	5	0	0.0%	0	0.0%	0	0.0%
mmu-mir-742-3p	X	64033551	64033572	-	edit	6	0	0.0%	0	0.0%	0	0.0%
mmu-mir-742-3p	X	64033551	64033572	-	edit	7	0	0.0%	0	0.0%	0	0.0%
mmu-mir-742-3p	X	64033551	64033572	-	edit	8	0	0.0%	0	0.0%	0	0.0%
mmu-mir-742-3p	X	64033551	64033572	-	edit	9	0	0.0%	0	0.0%	0	0.0%
mmu-mir-742-3p	X	64033551	64033572	-	edit	10	0	0.0%	0	0.0%	0	0.0%
mmu-mir-742-3p	X	64033551	64033572	-	edit	11	0	0.0%	0	0.0%	0	0.0%
mmu-mir-742-3p	X	64033551	64033572	-	edit	12	0	0.0%	0	0.0%	0	0.0%
mmu-mir-742-3p	X	64033551	64033572	-	edit	13	0	0.0%	0	0.0%	0	0.0%
mmu-mir-742-3p	X	64033551	64033572	-	edit	14	0	0.0%	0	0.0%	0	0.0%
mmu-mir-742-3p	X	64033551	64033572	-	edit	15	0	0.0%	0	0.0%	0	0.0%
mmu-mir-742-3p	X	64033551	64033572	-	edit	16	0	0.0%	0	0.0%	0	0.0%
mmu-mir-742-3p	X	64033551	64033572	-	edit	17	0	0.0%	0	0.0%	0	0.0%
mmu-mir-742-3p	X	64033551	64033572	-	edit	18	0	0.0%	0	0.0%	0	0.0%
mmu-mir-742-3p	X	64033551	64033572	-	edit	19	0	0.0%	0	0.0%	0	0.0%
mmu-mir-742-3p	X	64033551	64033572	-	edit	20	0	0.0%	0	0.0%	0	0.0%
mmu-mir-742-3p	X	64033551	64033572	-	edit	21	0	0.0%	0	0.0%	0	0.0%
mmu-mir-742-3p	X	64033551	64033572	-	edit	22	0	0.0%	0	0.0%	0	0.0%
EDITED							0	0.0%	0	0.0%	8	6.8%
CANONICAL							25	100.0%	74	100.0%	109	93.2%
TOTAL							25		74		117	

miRNA	Chr	Start	Stop	Strand	Type	Position	P7 reads	P7%	P10 reads	P10%	P14 reads	P14%
mmu-mir-743a-5p	X	64029935	64029956	-	5' Edit	5'	0	0.0%	0	0.0%	5	0.8%
mmu-mir-743a-5p	X	64029935	64029956	-	edit	1	0	0.0%	0	0.0%	0	0.0%
mmu-mir-743a-5p	X	64029935	64029956	-	edit	2	0	0.0%	0	0.0%	0	0.0%
mmu-mir-743a-5p	X	64029935	64029956	-	edit	3	0	0.0%	0	0.0%	0	0.0%
mmu-mir-743a-5p	X	64029935	64029956	-	edit	4	0	0.0%	0	0.0%	0	0.0%
mmu-mir-743a-5p	X	64029935	64029956	-	edit	5	0	0.0%	0	0.0%	0	0.0%
mmu-mir-743a-5p	X	64029935	64029956	-	edit	6	0	0.0%	0	0.0%	0	0.0%
mmu-mir-743a-5p	X	64029935	64029956	-	edit	7	0	0.0%	0	0.0%	0	0.0%
mmu-mir-743a-5p	X	64029935	64029956	-	edit	8	0	0.0%	0	0.0%	0	0.0%
mmu-mir-743a-5p	X	64029935	64029956	-	edit	9	0	0.0%	0	0.0%	0	0.0%
mmu-mir-743a-5p	X	64029935	64029956	-	edit	10	0	0.0%	0	0.0%	0	0.0%
mmu-mir-743a-5p	X	64029935	64029956	-	edit	11	0	0.0%	0	0.0%	0	0.0%
mmu-mir-743a-5p	X	64029935	64029956	-	edit	12	0	0.0%	0	0.0%	0	0.0%
mmu-mir-743a-5p	X	64029935	64029956	-	edit	13	0	0.0%	0	0.0%	0	0.0%
mmu-mir-743a-5p	X	64029935	64029956	-	edit	14	0	0.0%	0	0.0%	0	0.0%
mmu-mir-743a-5p	X	64029935	64029956	-	edit	15	0	0.0%	0	0.0%	0	0.0%
mmu-mir-743a-5p	X	64029935	64029956	-	edit	16	0	0.0%	0	0.0%	0	0.0%
mmu-mir-743a-5p	X	64029935	64029956	-	edit	17	0	0.0%	9	2.1%	0	0.0%
mmu-mir-743a-5p	X	64029935	64029956	-	edit	18	0	0.0%	0	0.0%	0	0.0%
mmu-mir-743a-5p	X	64029935	64029956	-	edit	19	0	0.0%	0	0.0%	11	1.7%
mmu-mir-743a-5p	X	64029935	64029956	-	edit	20	0	0.0%	0	0.0%	0	0.0%
mmu-mir-743a-5p	X	64029935	64029956	-	edit	21	0	0.0%	0	0.0%	0	0.0%
mmu-mir-743a-5p	X	64029935	64029956	-	edit	22	0	0.0%	0	0.0%	0	0.0%
EDITED							0	0.0%	9	2.1%	16	2.5%
CANONICAL							173	100.0%	399	97.9%	634	97.5%
TOTAL							173		408		650	

miRNA	Chr	Start	Stop	Strand	Type	Position	P7 reads	P7%	P10 reads	P10%	P14 reads	P14%
mmu-mir-743b-3p	X	64030441	64030462	-	Indel	22	0	0.0%	0	0.0%	32	0.5%
mmu-mir-743b-3p	X	64030441	64030462	-	edit	1	0	0.0%	0	0.0%	0	0.0%
mmu-mir-743b-3p	X	64030441	64030462	-	edit	2	0	0.0%	0	0.0%	0	0.0%
mmu-mir-743b-3p	X	64030441	64030462	-	edit	3	0	0.0%	0	0.0%	0	0.0%
mmu-mir-743b-3p	X	64030441	64030462	-	edit	4	0	0.0%	0	0.0%	0	0.0%
mmu-mir-743b-3p	X	64030441	64030462	-	edit	5	0	0.0%	0	0.0%	0	0.0%
mmu-mir-743b-3p	X	64030441	64030462	-	edit	6	0	0.0%	0	0.0%	0	0.0%
mmu-mir-743b-3p	X	64030441	64030462	-	edit	7	0	0.0%	0	0.0%	0	0.0%
mmu-mir-743b-3p	X	64030441	64030462	-	edit	8	0	0.0%	3	0.1%	6	0.1%
mmu-mir-743b-3p	X	64030441	64030462	-	edit	9	0	0.0%	0	0.0%	0	0.0%
mmu-mir-743b-3p	X	64030441	64030462	-	edit	10	0	0.0%	0	0.0%	0	0.0%
mmu-mir-743b-3p	X	64030441	64030462	-	edit	11	0	0.0%	0	0.0%	0	0.0%
mmu-mir-743b-3p	X	64030441	64030462	-	edit	12	0	0.0%	0	0.0%	0	0.0%
mmu-mir-743b-3p	X	64030441	64030462	-	edit	13	0	0.0%	0	0.0%	0	0.0%
mmu-mir-743b-3p	X	64030441	64030462	-	edit	14	0	0.0%	0	0.0%	0	0.0%
mmu-mir-743b-3p	X	64030441	64030462	-	edit	15	3	0.3%	6	0.2%	11	0.2%
mmu-mir-743b-3p	X	64030441	64030462	-	edit	16	0	0.0%	0	0.0%	0	0.0%
mmu-mir-743b-3p	X	64030441	64030462	-	edit	17	4	0.4%	56	1.7%	16	0.2%
mmu-mir-743b-3p	X	64030441	64030462	-	edit	18	0	0.0%	0	0.0%	0	0.0%
mmu-mir-743b-3p	X	64030441	64030462	-	edit	19	3	0.3%	3	0.1%	23	0.3%
mmu-mir-743b-3p	X	64030441	64030462	-	edit	20	0	0.0%	0	0.0%	0	0.0%
mmu-mir-743b-3p	X	64030441	64030462	-	edit	21	0	0.0%	0	0.0%	0	0.0%
mmu-mir-743b-3p	X	64030441	64030462	-	edit	22	0	0.0%	0	0.0%	0	0.0%
EDITED							10	0.9%	68	2.0%	88	1.3%
CANONICAL							1046	99.1%	3238	98.0%	6815	98.7%
TOTAL							1056		3306		6903	

miRNA	Chr	Start	Stop	Strand	Type	Position	P7 reads	P7%	P10 reads	P10%	P14 reads	P14%
mmu-mir-743b-5p	X	64030477	64030497	-	edit	1	0	0.0%	0	0.0%	0	0.0%
mmu-mir-743b-5p	X	64030477	64030497	-	edit	2	0	0.0%	0	0.0%	0	0.0%
mmu-mir-743b-5p	X	64030477	64030497	-	edit	3	0	0.0%	0	0.0%	0	0.0%
mmu-mir-743b-5p	X	64030477	64030497	-	edit	4	0	0.0%	0	0.0%	0	0.0%
mmu-mir-743b-5p	X	64030477	64030497	-	edit	5	0	0.0%	0	0.0%	0	0.0%
mmu-mir-743b-5p	X	64030477	64030497	-	edit	6	0	0.0%	0	0.0%	0	0.0%
mmu-mir-743b-5p	X	64030477	64030497	-	edit	7	0	0.0%	0	0.0%	0	0.0%
mmu-mir-743b-5p	X	64030477	64030497	-	edit	8	0	0.0%	0	0.0%	0	0.0%
mmu-mir-743b-5p	X	64030477	64030497	-	edit	9	0	0.0%	0	0.0%	0	0.0%
mmu-mir-743b-5p	X	64030477	64030497	-	edit	10	0	0.0%	0	0.0%	0	0.0%
mmu-mir-743b-5p	X	64030477	64030497	-	edit	11	0	0.0%	0	0.0%	0	0.0%
mmu-mir-743b-5p	X	64030477	64030497	-	edit	12	0	0.0%	0	0.0%	0	0.0%
mmu-mir-743b-5p	X	64030477	64030497	-	edit	13	0	0.0%	0	0.0%	0	0.0%
mmu-mir-743b-5p	X	64030477	64030497	-	edit	14	0	0.0%	0	0.0%	0	0.0%
mmu-mir-743b-5p	X	64030477	64030497	-	edit	15	0	0.0%	0	0.0%	0	0.0%
mmu-mir-743b-5p	X	64030477	64030497	-	edit	16	0	0.0%	0	0.0%	0	0.0%
mmu-mir-743b-5p	X	64030477	64030497	-	edit	17	0	0.0%	0	0.0%	4	1.0%
mmu-mir-743b-5p	X	64030477	64030497	-	edit	18	0	0.0%	0	0.0%	0	0.0%
mmu-mir-743b-5p	X	64030477	64030497	-	edit	19	0	0.0%	0	0.0%	0	0.0%
mmu-mir-743b-5p	X	64030477	64030497	-	edit	20	0	0.0%	0	0.0%	0	0.0%
mmu-mir-743b-5p	X	64030477	64030497	-	edit	21	0	0.0%	0	0.0%	0	0.0%
mmu-mir-743b-5p	X	64030477	64030497	-	edit	22	0	0.0%	0	0.0%	0	0.0%
EDITED							0	0.0%	0	0.0%	4	1.0%
CANONICAL							62	100.0%	287	100.0%	351	99.0%
TOTAL							62		287		355	

miRNA	Chr	Start	Stop	Strand	Type	Position	P7 reads	P7%	P10 reads	P10%	P14 reads	P14%
mmu-mir-744-5p	11	65548301	65548322	-	5' Edit	5'	3	0.0%	0	0.0%	0	0.0%
mmu-mir-744-5p	11	65548301	65548322	-	edit	1	0	0.0%	0	0.0%	0	0.0%
mmu-mir-744-5p	11	65548301	65548322	-	edit	2	0	0.0%	0	0.0%	0	0.0%
mmu-mir-744-5p	11	65548301	65548322	-	edit	3	0	0.0%	0	0.0%	0	0.0%
mmu-mir-744-5p	11	65548301	65548322	-	edit	4	0	0.0%	7	0.1%	0	0.0%
mmu-mir-744-5p	11	65548301	65548322	-	edit	5	0	0.0%	0	0.0%	0	0.0%
mmu-mir-744-5p	11	65548301	65548322	-	edit	6	0	0.0%	0	0.0%	0	0.0%
mmu-mir-744-5p	11	65548301	65548322	-	edit	7	0	0.0%	0	0.0%	0	0.0%
mmu-mir-744-5p	11	65548301	65548322	-	edit	8	8	0.1%	12	0.2%	0	0.0%
mmu-mir-744-5p	11	65548301	65548322	-	edit	9	0	0.0%	0	0.0%	0	0.0%
mmu-mir-744-5p	11	65548301	65548322	-	edit	10	0	0.0%	0	0.0%	0	0.0%
mmu-mir-744-5p	11	65548301	65548322	-	edit	11	0	0.0%	0	0.0%	0	0.0%
mmu-mir-744-5p	11	65548301	65548322	-	edit	12	0	0.0%	0	0.0%	0	0.0%
mmu-mir-744-5p	11	65548301	65548322	-	edit	13	0	0.0%	0	0.0%	0	0.0%
mmu-mir-744-5p	11	65548301	65548322	-	edit	14	0	0.0%	0	0.0%	0	0.0%
mmu-mir-744-5p	11	65548301	65548322	-	edit	15	29	0.4%	17	0.3%	8	0.3%
mmu-mir-744-5p	11	65548301	65548322	-	edit	16	10	0.1%	42	0.6%	5	0.2%
mmu-mir-744-5p	11	65548301	65548322	-	edit	17	18	0.3%	139	2.1%	11	0.4%
mmu-mir-744-5p	11	65548301	65548322	-	edit	18	0	0.0%	0	0.0%	0	0.0%
mmu-mir-744-5p	11	65548301	65548322	-	edit	19	20	0.3%	97	1.5%	14	0.5%
mmu-mir-744-5p	11	65548301	65548322	-	edit	20	0	0.0%	0	0.0%	0	0.0%
mmu-mir-744-5p	11	65548301	65548322	-	edit	21	11	0.2%	4	0.1%	0	0.0%
mmu-mir-744-5p	11	65548301	65548322	-	edit	22	0	0.0%	0	0.0%	0	0.0%
EDITED							99	1.4%	317	4.8%	38	1.5%
CANONICAL							7147	98.6%	6279	95.2%	2511	98.5%
TOTAL							7246		6596		2549	

miRNA	Chr	Start	Stop	Strand	Type	Position	P7 reads	P7%	P10 reads	P10%	P14 reads	P14%
mmu-mir-871-5p	X	64063647	64063669	-	edit	1	0	0.0%	0	0.0%	0	0.0%
mmu-mir-871-5p	X	64063647	64063669	-	edit	2	0	0.0%	0	0.0%	0	0.0%
mmu-mir-871-5p	X	64063647	64063669	-	edit	3	0	0.0%	0	0.0%	0	0.0%
mmu-mir-871-5p	X	64063647	64063669	-	edit	4	0	0.0%	0	0.0%	0	0.0%
mmu-mir-871-5p	X	64063647	64063669	-	edit	5	0	0.0%	0	0.0%	0	0.0%
mmu-mir-871-5p	X	64063647	64063669	-	edit	6	0	0.0%	0	0.0%	0	0.0%
mmu-mir-871-5p	X	64063647	64063669	-	edit	7	0	0.0%	0	0.0%	0	0.0%
mmu-mir-871-5p	X	64063647	64063669	-	edit	8	0	0.0%	0	0.0%	0	0.0%
mmu-mir-871-5p	X	64063647	64063669	-	edit	9	0	0.0%	0	0.0%	0	0.0%
mmu-mir-871-5p	X	64063647	64063669	-	edit	10	0	0.0%	0	0.0%	0	0.0%
mmu-mir-871-5p	X	64063647	64063669	-	edit	11	0	0.0%	0	0.0%	0	0.0%
mmu-mir-871-5p	X	64063647	64063669	-	edit	12	0	0.0%	0	0.0%	0	0.0%
mmu-mir-871-5p	X	64063647	64063669	-	edit	13	0	0.0%	0	0.0%	0	0.0%
mmu-mir-871-5p	X	64063647	64063669	-	edit	14	0	0.0%	0	0.0%	0	0.0%
mmu-mir-871-5p	X	64063647	64063669	-	edit	15	0	0.0%	0	0.0%	0	0.0%
mmu-mir-871-5p	X	64063647	64063669	-	edit	16	0	0.0%	0	0.0%	0	0.0%
mmu-mir-871-5p	X	64063647	64063669	-	edit	17	0	0.0%	3	2.1%	0	0.0%
mmu-mir-871-5p	X	64063647	64063669	-	edit	18	0	0.0%	0	0.0%	0	0.0%
mmu-mir-871-5p	X	64063647	64063669	-	edit	19	0	0.0%	0	0.0%	0	0.0%
mmu-mir-871-5p	X	64063647	64063669	-	edit	20	0	0.0%	0	0.0%	0	0.0%
mmu-mir-871-5p	X	64063647	64063669	-	edit	21	0	0.0%	0	0.0%	0	0.0%
mmu-mir-871-5p	X	64063647	64063669	-	edit	22	0	0.0%	0	0.0%	0	0.0%
EDITED							0	0.0%	3	2.1%	0	0.0%
CANONICAL							58	100.0%	138	97.9%	130	100.0%
TOTAL							58		141		130	

miRNA	Chr	Start	Stop	Strand	Type	Position	P7 reads	P7%	P10 reads	P10%	P14 reads	P14%
mmu-mir-872-5p	4	94331858	94331878	+	edit	1	0	0.0%	0	0.0%	0	0.0%
mmu-mir-872-5p	4	94331858	94331878	+	edit	2	0	0.0%	0	0.0%	0	0.0%
mmu-mir-872-5p	4	94331858	94331878	+	edit	3	0	0.0%	0	0.0%	0	0.0%
mmu-mir-872-5p	4	94331858	94331878	+	edit	4	0	0.0%	0	0.0%	0	0.0%
mmu-mir-872-5p	4	94331858	94331878	+	edit	5	0	0.0%	0	0.0%	0	0.0%
mmu-mir-872-5p	4	94331858	94331878	+	edit	6	0	0.0%	0	0.0%	0	0.0%
mmu-mir-872-5p	4	94331858	94331878	+	edit	7	0	0.0%	0	0.0%	0	0.0%
mmu-mir-872-5p	4	94331858	94331878	+	edit	8	0	0.0%	0	0.0%	0	0.0%
mmu-mir-872-5p	4	94331858	94331878	+	edit	9	0	0.0%	0	0.0%	0	0.0%
mmu-mir-872-5p	4	94331858	94331878	+	edit	10	0	0.0%	0	0.0%	0	0.0%
mmu-mir-872-5p	4	94331858	94331878	+	edit	11	0	0.0%	0	0.0%	0	0.0%
mmu-mir-872-5p	4	94331858	94331878	+	edit	12	0	0.0%	0	0.0%	0	0.0%
mmu-mir-872-5p	4	94331858	94331878	+	edit	13	0	0.0%	0	0.0%	0	0.0%
mmu-mir-872-5p	4	94331858	94331878	+	edit	14	0	0.0%	0	0.0%	0	0.0%
mmu-mir-872-5p	4	94331858	94331878	+	edit	15	3	0.2%	0	0.0%	0	0.0%
mmu-mir-872-5p	4	94331858	94331878	+	edit	16	0	0.0%	0	0.0%	0	0.0%
mmu-mir-872-5p	4	94331858	94331878	+	edit	17	0	0.0%	0	0.0%	0	0.0%
mmu-mir-872-5p	4	94331858	94331878	+	edit	18	0	0.0%	0	0.0%	0	0.0%
mmu-mir-872-5p	4	94331858	94331878	+	edit	19	0	0.0%	3	0.3%	3	0.3%
mmu-mir-872-5p	4	94331858	94331878	+	edit	20	0	0.0%	0	0.0%	0	0.0%
mmu-mir-872-5p	4	94331858	94331878	+	edit	21	0	0.0%	0	0.0%	0	0.0%
mmu-mir-872-5p	4	94331858	94331878	+	edit	22	0	0.0%	0	0.0%	0	0.0%
EDITED							3	0.2%	3	0.3%	3	0.3%
CANONICAL							1613	99.8%	963	99.7%	870	99.7%
TOTAL							1616		966		873	

miRNA	Chr	Start	Stop	Strand	Type	Position	P7 reads	P7%	P10 reads	P10%	P14 reads	P14%
mmu-mir-877-5p	17	36097740	36097759	-	edit	1	0	0.0%	0	0.0%	0	0.0%
mmu-mir-877-5p	17	36097740	36097759	-	edit	2	0	0.0%	0	0.0%	0	0.0%
mmu-mir-877-5p	17	36097740	36097759	-	edit	3	0	0.0%	0	0.0%	0	0.0%
mmu-mir-877-5p	17	36097740	36097759	-	edit	4	0	0.0%	0	0.0%	0	0.0%
mmu-mir-877-5p	17	36097740	36097759	-	edit	5	0	0.0%	0	0.0%	0	0.0%
mmu-mir-877-5p	17	36097740	36097759	-	edit	6	0	0.0%	0	0.0%	0	0.0%
mmu-mir-877-5p	17	36097740	36097759	-	edit	7	0	0.0%	0	0.0%	0	0.0%
mmu-mir-877-5p	17	36097740	36097759	-	edit	8	0	0.0%	0	0.0%	0	0.0%
mmu-mir-877-5p	17	36097740	36097759	-	edit	9	0	0.0%	0	0.0%	0	0.0%
mmu-mir-877-5p	17	36097740	36097759	-	edit	10	0	0.0%	0	0.0%	0	0.0%
mmu-mir-877-5p	17	36097740	36097759	-	edit	11	0	0.0%	0	0.0%	0	0.0%
mmu-mir-877-5p	17	36097740	36097759	-	edit	12	0	0.0%	0	0.0%	0	0.0%
mmu-mir-877-5p	17	36097740	36097759	-	edit	13	0	0.0%	0	0.0%	0	0.0%
mmu-mir-877-5p	17	36097740	36097759	-	edit	14	0	0.0%	0	0.0%	0	0.0%
mmu-mir-877-5p	17	36097740	36097759	-	edit	15	0	0.0%	0	0.0%	0	0.0%
mmu-mir-877-5p	17	36097740	36097759	-	edit	16	0	0.0%	0	0.0%	0	0.0%
mmu-mir-877-5p	17	36097740	36097759	-	edit	17	4	0.7%	7	2.0%	0	0.0%
mmu-mir-877-5p	17	36097740	36097759	-	edit	18	0	0.0%	0	0.0%	0	0.0%
mmu-mir-877-5p	17	36097740	36097759	-	edit	19	0	0.0%	0	0.0%	0	0.0%
mmu-mir-877-5p	17	36097740	36097759	-	edit	20	0	0.0%	0	0.0%	0	0.0%
mmu-mir-877-5p	17	36097740	36097759	-	edit	21	0	0.0%	0	0.0%	0	0.0%
mmu-mir-877-5p	17	36097740	36097759	-	edit	22	0	0.0%	0	0.0%	0	0.0%
EDITED							4	0.7%	7	2.0%	0	0.0%
CANONICAL							497	99.3%	349	98.0%	297	100.0%
TOTAL							501		356		297	

miRNA	Chr	Start	Stop	Strand	Type	Position	P7 reads	P7%	P10 reads	P10%	P14 reads	P14%
mmu-mir-878-3p	X	64054693	64054714	-	edit	1	0	0.0%	0	0.0%	0	0.0%
mmu-mir-878-3p	X	64054693	64054714	-	edit	2	0	0.0%	0	0.0%	0	0.0%
mmu-mir-878-3p	X	64054693	64054714	-	edit	3	0	0.0%	0	0.0%	0	0.0%
mmu-mir-878-3p	X	64054693	64054714	-	edit	4	0	0.0%	0	0.0%	0	0.0%
mmu-mir-878-3p	X	64054693	64054714	-	edit	5	0	0.0%	0	0.0%	0	0.0%
mmu-mir-878-3p	X	64054693	64054714	-	edit	6	0	0.0%	0	0.0%	0	0.0%
mmu-mir-878-3p	X	64054693	64054714	-	edit	7	0	0.0%	0	0.0%	0	0.0%
mmu-mir-878-3p	X	64054693	64054714	-	edit	8	0	0.0%	0	0.0%	0	0.0%
mmu-mir-878-3p	X	64054693	64054714	-	edit	9	0	0.0%	0	0.0%	0	0.0%
mmu-mir-878-3p	X	64054693	64054714	-	edit	10	0	0.0%	0	0.0%	0	0.0%
mmu-mir-878-3p	X	64054693	64054714	-	edit	11	0	0.0%	0	0.0%	0	0.0%
mmu-mir-878-3p	X	64054693	64054714	-	edit	12	0	0.0%	0	0.0%	0	0.0%
mmu-mir-878-3p	X	64054693	64054714	-	edit	13	0	0.0%	0	0.0%	0	0.0%
mmu-mir-878-3p	X	64054693	64054714	-	edit	14	0	0.0%	0	0.0%	0	0.0%
mmu-mir-878-3p	X	64054693	64054714	-	edit	15	0	0.0%	0	0.0%	3	1.7%
mmu-mir-878-3p	X	64054693	64054714	-	edit	16	0	0.0%	0	0.0%	0	0.0%
mmu-mir-878-3p	X	64054693	64054714	-	edit	17	0	0.0%	0	0.0%	0	0.0%
mmu-mir-878-3p	X	64054693	64054714	-	edit	18	0	0.0%	0	0.0%	0	0.0%
mmu-mir-878-3p	X	64054693	64054714	-	edit	19	0	0.0%	0	0.0%	9	5.0%
mmu-mir-878-3p	X	64054693	64054714	-	edit	20	0	0.0%	0	0.0%	0	0.0%
mmu-mir-878-3p	X	64054693	64054714	-	edit	21	0	0.0%	0	0.0%	0	0.0%
mmu-mir-878-3p	X	64054693	64054714	-	edit	22	0	0.0%	0	0.0%	0	0.0%
EDITED							0	0.0%	0	0.0%	12	6.6%
CANONICAL							26	100.0%	118	100.0%	169	93.4%
TOTAL							26		118		181	

miRNA	Chr	Start	Stop	Strand	Type	Position	P7 reads	P7%	P10 reads	P10%	P14 reads	P14%
mmu-mir-878-5p	X	64054729	64054750	-	Indel	22	0	0.0%	0	0.0%	3	0.7%
mmu-mir-878-5p	X	64054729	64054750	-	edit	1	0	0.0%	0	0.0%	0	0.0%
mmu-mir-878-5p	X	64054729	64054750	-	edit	2	0	0.0%	0	0.0%	0	0.0%
mmu-mir-878-5p	X	64054729	64054750	-	edit	3	0	0.0%	0	0.0%	0	0.0%
mmu-mir-878-5p	X	64054729	64054750	-	edit	4	0	0.0%	0	0.0%	0	0.0%
mmu-mir-878-5p	X	64054729	64054750	-	edit	5	0	0.0%	0	0.0%	0	0.0%
mmu-mir-878-5p	X	64054729	64054750	-	edit	6	0	0.0%	0	0.0%	0	0.0%
mmu-mir-878-5p	X	64054729	64054750	-	edit	7	0	0.0%	0	0.0%	0	0.0%
mmu-mir-878-5p	X	64054729	64054750	-	edit	8	0	0.0%	0	0.0%	0	0.0%
mmu-mir-878-5p	X	64054729	64054750	-	edit	9	0	0.0%	0	0.0%	0	0.0%
mmu-mir-878-5p	X	64054729	64054750	-	edit	10	0	0.0%	0	0.0%	0	0.0%
mmu-mir-878-5p	X	64054729	64054750	-	edit	11	0	0.0%	0	0.0%	0	0.0%
mmu-mir-878-5p	X	64054729	64054750	-	edit	12	0	0.0%	0	0.0%	0	0.0%
mmu-mir-878-5p	X	64054729	64054750	-	edit	13	0	0.0%	0	0.0%	0	0.0%
mmu-mir-878-5p	X	64054729	64054750	-	edit	14	0	0.0%	0	0.0%	0	0.0%
mmu-mir-878-5p	X	64054729	64054750	-	edit	15	0	0.0%	0	0.0%	0	0.0%
mmu-mir-878-5p	X	64054729	64054750	-	edit	16	0	0.0%	0	0.0%	3	0.7%
mmu-mir-878-5p	X	64054729	64054750	-	edit	17	0	0.0%	0	0.0%	0	0.0%
mmu-mir-878-5p	X	64054729	64054750	-	edit	18	0	0.0%	0	0.0%	0	0.0%
mmu-mir-878-5p	X	64054729	64054750	-	edit	19	0	0.0%	0	0.0%	0	0.0%
mmu-mir-878-5p	X	64054729	64054750	-	edit	20	0	0.0%	0	0.0%	0	0.0%
mmu-mir-878-5p	X	64054729	64054750	-	edit	21	0	0.0%	0	0.0%	0	0.0%
mmu-mir-878-5p	X	64054729	64054750	-	edit	22	0	0.0%	0	0.0%	0	0.0%
EDITED							0	0.0%	0	0.0%	6	1.4%
CANONICAL							124	100.0%	310	100.0%	422	98.6%
TOTAL							124		310		428	

miRNA	Chr	Start	Stop	Strand	Type	Position	P7 reads	P7%	P10 reads	P10%	P14 reads	P14%
mmu-mir-880-5p	X	64053715	64053736	-	Indel	22	0	0.0%	0	0.0%	4	0.6%
mmu-mir-880-5p	X	64053715	64053736	-	edit	1	0	0.0%	0	0.0%	0	0.0%
mmu-mir-880-5p	X	64053715	64053736	-	edit	2	0	0.0%	0	0.0%	0	0.0%
mmu-mir-880-5p	X	64053715	64053736	-	edit	3	0	0.0%	0	0.0%	0	0.0%
mmu-mir-880-5p	X	64053715	64053736	-	edit	4	0	0.0%	0	0.0%	0	0.0%
mmu-mir-880-5p	X	64053715	64053736	-	edit	5	0	0.0%	0	0.0%	0	0.0%
mmu-mir-880-5p	X	64053715	64053736	-	edit	6	0	0.0%	0	0.0%	0	0.0%
mmu-mir-880-5p	X	64053715	64053736	-	edit	7	0	0.0%	0	0.0%	0	0.0%
mmu-mir-880-5p	X	64053715	64053736	-	edit	8	0	0.0%	0	0.0%	0	0.0%
mmu-mir-880-5p	X	64053715	64053736	-	edit	9	0	0.0%	0	0.0%	0	0.0%
mmu-mir-880-5p	X	64053715	64053736	-	edit	10	0	0.0%	0	0.0%	0	0.0%
mmu-mir-880-5p	X	64053715	64053736	-	edit	11	0	0.0%	0	0.0%	0	0.0%
mmu-mir-880-5p	X	64053715	64053736	-	edit	12	0	0.0%	0	0.0%	0	0.0%
mmu-mir-880-5p	X	64053715	64053736	-	edit	13	0	0.0%	0	0.0%	0	0.0%
mmu-mir-880-5p	X	64053715	64053736	-	edit	14	0	0.0%	0	0.0%	0	0.0%
mmu-mir-880-5p	X	64053715	64053736	-	edit	15	0	0.0%	0	0.0%	0	0.0%
mmu-mir-880-5p	X	64053715	64053736	-	edit	16	0	0.0%	0	0.0%	0	0.0%
mmu-mir-880-5p	X	64053715	64053736	-	edit	17	0	0.0%	19	2.5%	4	0.5%
mmu-mir-880-5p	X	64053715	64053736	-	edit	18	0	0.0%	0	0.0%	0	0.0%
mmu-mir-880-5p	X	64053715	64053736	-	edit	19	4	2.1%	0	0.0%	9	1.3%
mmu-mir-880-5p	X	64053715	64053736	-	edit	20	0	0.0%	0	0.0%	0	0.0%
mmu-mir-880-5p	X	64053715	64053736	-	edit	21	0	0.0%	0	0.0%	0	0.0%
mmu-mir-880-5p	X	64053715	64053736	-	edit	22	0	0.0%	0	0.0%	0	0.0%
EDITED							4	2.1%	19	2.5%	17	2.4%
CANONICAL							191	97.9%	731	97.5%	685	97.6%
TOTAL							195		750		702	

miRNA	Chr	Start	Stop	Strand	Type	Position	P7 reads	P7%	P10 reads	P10%	P14 reads	P14%
mmu-mir-881-3p	X	64055129	64055150	-	Indel	22	0	0.0%	8	0.2%	21	0.4%
mmu-mir-881-3p	X	64055129	64055150	-	edit	1	0	0.0%	0	0.0%	0	0.0%
mmu-mir-881-3p	X	64055129	64055150	-	edit	2	0	0.0%	0	0.0%	0	0.0%
mmu-mir-881-3p	X	64055129	64055150	-	edit	3	0	0.0%	0	0.0%	0	0.0%
mmu-mir-881-3p	X	64055129	64055150	-	edit	4	4	0.2%	0	0.0%	3	0.1%
mmu-mir-881-3p	X	64055129	64055150	-	edit	5	0	0.0%	0	0.0%	0	0.0%
mmu-mir-881-3p	X	64055129	64055150	-	edit	6	0	0.0%	0	0.0%	0	0.0%
mmu-mir-881-3p	X	64055129	64055150	-	edit	7	0	0.0%	0	0.0%	0	0.0%
mmu-mir-881-3p	X	64055129	64055150	-	edit	8	3	0.2%	4	0.1%	9	0.2%
mmu-mir-881-3p	X	64055129	64055150	-	edit	9	0	0.0%	0	0.0%	0	0.0%
mmu-mir-881-3p	X	64055129	64055150	-	edit	10	0	0.0%	0	0.0%	0	0.0%
mmu-mir-881-3p	X	64055129	64055150	-	edit	11	0	0.0%	0	0.0%	0	0.0%
mmu-mir-881-3p	X	64055129	64055150	-	edit	12	0	0.0%	0	0.0%	0	0.0%
mmu-mir-881-3p	X	64055129	64055150	-	edit	13	0	0.0%	0	0.0%	0	0.0%
mmu-mir-881-3p	X	64055129	64055150	-	edit	14	0	0.0%	0	0.0%	0	0.0%
mmu-mir-881-3p	X	64055129	64055150	-	edit	15	0	0.0%	0	0.0%	6	0.1%
mmu-mir-881-3p	X	64055129	64055150	-	edit	16	0	0.0%	0	0.0%	0	0.0%
mmu-mir-881-3p	X	64055129	64055150	-	edit	17	6	0.3%	60	1.5%	20	0.4%
mmu-mir-881-3p	X	64055129	64055150	-	edit	18	0	0.0%	0	0.0%	0	0.0%
mmu-mir-881-3p	X	64055129	64055150	-	edit	19	0	0.0%	3	0.1%	5	0.1%
mmu-mir-881-3p	X	64055129	64055150	-	edit	20	0	0.0%	0	0.0%	0	0.0%
mmu-mir-881-3p	X	64055129	64055150	-	edit	21	0	0.0%	0	0.0%	0	0.0%
mmu-mir-881-3p	X	64055129	64055150	-	edit	22	0	0.0%	0	0.0%	0	0.0%
EDITED							13	0.7%	75	1.8%	64	1.1%
CANONICAL							1735	99.3%	4020	98.2%	5516	98.9%
TOTAL							1748		4095		5580	

**miRNA**	**Chr**	**Start**	**Stop**	**Strand**	**Type**	**Position**	**P7 reads**	**P7%**	**P10 reads**	**P10%**	**P14 reads**	**P14%**
mmu-mir-883a-3p	X	64033943	64033964	-	edit	1	0	0.0%	0	0.0%	0	0.0%
mmu-mir-883a-3p	X	64033943	64033964	-	edit	2	0	0.0%	0	0.0%	0	0.0%
mmu-mir-883a-3p	X	64033943	64033964	-	edit	3	0	0.0%	0	0.0%	0	0.0%
mmu-mir-883a-3p	X	64033943	64033964	-	edit	4	0	0.0%	0	0.0%	0	0.0%
mmu-mir-883a-3p	X	64033943	64033964	-	edit	5	0	0.0%	0	0.0%	0	0.0%
mmu-mir-883a-3p	X	64033943	64033964	-	edit	6	0	0.0%	0	0.0%	0	0.0%
mmu-mir-883a-3p	X	64033943	64033964	-	edit	7	0	0.0%	0	0.0%	0	0.0%
mmu-mir-883a-3p	X	64033943	64033964	-	edit	8	0	0.0%	0	0.0%	0	0.0%
mmu-mir-883a-3p	X	64033943	64033964	-	edit	9	0	0.0%	0	0.0%	0	0.0%
mmu-mir-883a-3p	X	64033943	64033964	-	edit	10	0	0.0%	0	0.0%	0	0.0%
mmu-mir-883a-3p	X	64033943	64033964	-	edit	11	0	0.0%	0	0.0%	0	0.0%
mmu-mir-883a-3p	X	64033943	64033964	-	edit	12	0	0.0%	0	0.0%	0	0.0%
mmu-mir-883a-3p	X	64033943	64033964	-	edit	13	0	0.0%	0	0.0%	0	0.0%
mmu-mir-883a-3p	X	64033943	64033964	-	edit	14	0	0.0%	0	0.0%	0	0.0%
mmu-mir-883a-3p	X	64033943	64033964	-	edit	15	0	0.0%	3	0.1%	6	0.1%
mmu-mir-883a-3p	X	64033943	64033964	-	edit	16	0	0.0%	0	0.0%	11	0.2%
mmu-mir-883a-3p	X	64033943	64033964	-	edit	17	0	0.0%	3	0.1%	5	0.1%
mmu-mir-883a-3p	X	64033943	64033964	-	edit	18	0	0.0%	35	1.1%	13	0.3%
mmu-mir-883a-3p	X	64033943	64033964	-	edit	19	0	0.0%	0	0.0%	0	0.0%
mmu-mir-883a-3p	X	64033943	64033964	-	edit	20	11	0.8%	10	0.3%	28	0.6%
mmu-mir-883a-3p	X	64033943	64033964	-	edit	21	0	0.0%	0	0.0%	0	0.0%
mmu-mir-883a-3p	X	64033943	64033964	-	edit	22	0	0.0%	0	0.0%	0	0.0%
EDITED							11	0.8%	51	1.6%	63	1.3%
CANONICAL							1348	99.2%	3082	98.4%	4615	98.7%
TOTAL							1359		3133		4678	

miRNA	Chr	Start	Stop	Strand	Type	Position	P7 reads	P7%	P10 reads	P10%	P14 reads	P14%
mmu-mir-883b-5p	X	64043111	64043132	-	edit	1	0	0.0%	0	0.0%	0	0.0%
mmu-mir-883b-5p	X	64043111	64043132	-	edit	2	0	0.0%	0	0.0%	0	0.0%
mmu-mir-883b-5p	X	64043111	64043132	-	edit	3	0	0.0%	0	0.0%	0	0.0%
mmu-mir-883b-5p	X	64043111	64043132	-	edit	4	0	0.0%	0	0.0%	0	0.0%
mmu-mir-883b-5p	X	64043111	64043132	-	edit	5	0	0.0%	0	0.0%	0	0.0%
mmu-mir-883b-5p	X	64043111	64043132	-	edit	6	0	0.0%	0	0.0%	0	0.0%
mmu-mir-883b-5p	X	64043111	64043132	-	edit	7	0	0.0%	0	0.0%	0	0.0%
mmu-mir-883b-5p	X	64043111	64043132	-	edit	8	0	0.0%	0	0.0%	0	0.0%
mmu-mir-883b-5p	X	64043111	64043132	-	edit	9	0	0.0%	0	0.0%	0	0.0%
mmu-mir-883b-5p	X	64043111	64043132	-	edit	10	0	0.0%	0	0.0%	0	0.0%
mmu-mir-883b-5p	X	64043111	64043132	-	edit	11	0	0.0%	0	0.0%	0	0.0%
mmu-mir-883b-5p	X	64043111	64043132	-	edit	12	0	0.0%	0	0.0%	0	0.0%
mmu-mir-883b-5p	X	64043111	64043132	-	edit	13	0	0.0%	0	0.0%	0	0.0%
mmu-mir-883b-5p	X	64043111	64043132	-	edit	14	0	0.0%	0	0.0%	0	0.0%
mmu-mir-883b-5p	X	64043111	64043132	-	edit	15	0	0.0%	0	0.0%	0	0.0%
mmu-mir-883b-5p	X	64043111	64043132	-	edit	16	0	0.0%	0	0.0%	0	0.0%
mmu-mir-883b-5p	X	64043111	64043132	-	edit	17	0	0.0%	4	1.2%	0	0.0%
mmu-mir-883b-5p	X	64043111	64043132	-	edit	18	0	0.0%	0	0.0%	0	0.0%
mmu-mir-883b-5p	X	64043111	64043132	-	edit	19	0	0.0%	0	0.0%	0	0.0%
mmu-mir-883b-5p	X	64043111	64043132	-	edit	20	0	0.0%	0	0.0%	0	0.0%
mmu-mir-883b-5p	X	64043111	64043132	-	edit	21	3	1.8%	3	0.9%	8	2.9%
mmu-mir-883b-5p	X	64043111	64043132	-	edit	22	0	0.0%	0	0.0%	0	0.0%
EDITED							3	1.8%	7	2.1%	8	2.9%
CANONICAL							163	98.2%	332	97.9%	268	97.1%
TOTAL							166		339		276	
