# Supplementary material for: Analysis of MicroRNA Expression in the Prepubertal Testis
Source: PLoS One. 2010 Dec 29;5(12):e15317. doi: 10.1371/journal.pone.0015317 (PMC3012074; doi:10.1371/journal.pone.0015317)
Supplement: Table S4 — 5′ cleavage variants of miRNAs during prepubertal testicular development. 5′ variants are generally highest at P7 in the juvenile testis but represent a small fraction of total reads. (PDF) [file pone.0015317.s004.pdf]

5' variants

miRNA	P7 canonical	P7 variant	P7 % variant	P10 canonical	P10 variant	P10 % variant	P14 canonical	P14 variant	P14 % variant
mmu-let-7a-1-5p	356489	1595	0.4%	193610	0	0.00%	103860	696	0.67%
mmu-let-7d-5p	121532	219	0.2%	60054	0	0.00%	35740	92	0.26%
mmu-let-7f-1-5p	607029	1028	0.2%	352131	0	0.00%	225350	400	0.18%
mmu-mir-21-5p	6659	17	0.3%	4952	0	0.00%	3182	10	0.31%
mmu-mir-25-5p	30958	618	2.0%	13809	0	0.00%	12435	405	3.25%
mmu-mir-30d-5p	7116	9	0.1%	5031	0	0.00%	3302	13	0.39%
mmu-mir-34c-5p	2719	0	0.0%	2069	0	0.00%	27073	4	0.01%
mmu-mir-101a-3p	7466	0	0.0%	7364	0	0.00%	4625	4	0.08%
mmu-mir-107-5p	39174	23	0.1%	26183	0	0.00%	17078	18	0.10%
mmu-mir-143-5p	10776	4	0.0%	9027	0	0.00%	6303	4	0.06%
mmu-mir-181d-5p	23294	23	0.1%	6626	0	0.00%	4197	4	0.10%
mmu-mir-185-5p	6476	7	0.1%	5028	0	0.00%	2595	0	0.00%
mmu-mir-199a-1-3p	77201	6	0.0%	29801	0	0.00%	16413	0	0.00%
mmu-mir-322-5p	699	19	2.7%	378	0	0.00%	223	0	0.00%
mmu-mir-423-5p	35295	9	0.0%	21790	0	0.00%	14764	9	0.06%
mmu-mir-465c-1-5p	1229	0	0.0%	3357	0	0.00%	2724	6	0.22%
mmu-mir-465c-2-5p	1229	0	0.0%	3357	0	0.00%	2724	6	0.22%
mmu-mir-503-5p	10470	20	0.2%	4502	0	0.00%	1373	0	0.00%
mmu-mir-743a-5p	173	0	0.0%	399	0	0.00%	634	5	0.79%
mmu-mir-744-5p	7147	3	0.0%	6279	0	0.00%	2511	0	0.00%
