# Supplementary material for: Analysis of MicroRNA Expression in the Prepubertal Testis
Source: PLoS One. 2010 Dec 29;5(12):e15317. doi: 10.1371/journal.pone.0015317 (PMC3012074; doi:10.1371/journal.pone.0015317)
Supplement: Table S5 — Internal editing of miRNAs during prepubertal testicular development. Most internal editing events are highest at P14 in the juvenile testis, but other patterns are detected less frequently. Increased editing is associated with declining levels of the miRNA in 55% of cases. Uridine was the most common base affected. The affected positions are bolded within the mature miRNA sequence. (PDF) [file pone.0015317.s005.pdf]

Editing of miRNAs

miRNA	P7 total	P7 % variant	P10 total	P10 % variant	P14 total	P14 % variant	miRNA	editing	position	nucleotide	miRNA sequence
mmu-let-7b-3p	802	26.0%	287	18.2%	238	15.0%	decreasing	decreasing	15	G	CUAUACAACCUACU**G**CCUUCCC
mmu-mir-34b-5p	66	15.2%	41	9.8%	599	12.9%	increasing	decreasing	11	A	AGGCAGUGUA**A**UUAGCUGAUUGU
mmu-mir-98-5p	2113	8.3%	1347	5.8%	912	10.6%	decreasing	increasing	19	U	UGAGGUAGUAAGUUGUAU**U**GUU
mmu-mir-99b-3p	1819	5.4%	556	5.5%	286	10.1%	decreasing	increasing	19	U	CAAGCUCGUGUCUGUGGG**U**CCG
mmu-mir-181a-1-5p	9486	6.9%	3982	5.3%	2162	9.6%	decreasing	increasing	19	U	AACAUUCAACGCUGUCGG**U**GAGU
mmu-mir-181a-2-5p	9400	6.9%	4004	5.3%	2143	9.8%	decreasing	increasing	19	U	AACAUUCAACGCUGUCGG**U**GAGU
mmu-mir-181b-1-5p	5488	7.9%	1846	6.7%	986	9.9%	decreasing	increasing	19	U	AACAUUCAUUGCUGUCGG**U**GGGU
mmu-mir-181b-2-5p	11749	7.3%	3977	6.9%	2118	9.5%	decreasing	increasing	19	U	AACAUUCAUUGCUGUCGG**U**GGGU
mmu-mir-199a-1-5p	342	3.2%	149	4.7%	86	4.7%	decreasing	increasing	17	G	UAGGUAGUUUCAUGUU**G**UUGGG
mmu-mir-199a-2-5p	322	3.4%	143	4.9%	78	5.1%	decreasing	increasing	17	G	UAGGUAGUUUCAUGUU**G**UUGGG
mmu-mir-322-5p	857	3.7%	436	8.3%	256	3.1%	decreasing	peak	6, 13-17	G/U	CAGCA**G**CAAUUCA**UGUU**UUGGA
mmu-mir-337-5p	406	0.7%	160	5.3%	47	0.0%	decreasing	peak	18	A	GAACGGCGUCAUGCAGG**A**GUU
mmu-mir-376b-3p	198	11.6%	29	13.8%	11	0.0%	decreasing	decreasing	6	A	AUCAU**A**GAGGAACAUCCACUU
mmu-mir-485-5p	2597	2.8%	695	10.4%	272	4.8%	decreasing	peak	15	A	AGAGGCUGGCCGUG**A**UGAAUUC
mmu-mir-503-5p	10995	4.8%	4756	5.3%	1481	7.3%	decreasing	increasing	16, 19	U	UAGCAGCGGGAACAG**U**AC**U**GCAG
mmu-mir-532-5p	760	6.3%	362	10.8%	377	13.8%	decreasing	increasing	19	C	CAUGCCUUGAGUGUAGGA**C**CGU
mmu-mir-668-5p	202	3.5%	97	8.2%	16	0.0%	decreasing	peak	19	A	UGUCACUCGGCUCGGCCC**A**CUACC
mmu-mir-742-3p	25	0.0%	74	0.0%	117	6.8%	increasing	increasing	22	A	GAAAGCCACCAUGCUGGGUAA**A**
