# Supplementary material for: Analysis of MicroRNA Expression in the Prepubertal Testis
Source: PLoS One. 2010 Dec 29;5(12):e15317. doi: 10.1371/journal.pone.0015317 (PMC3012074; doi:10.1371/journal.pone.0015317)
Supplement: Table S6 — 3′ nuclotide addition to miRNAs during prepubertal testicular development. 3′ nucleotide addition increased over juvenile testis development (61% of cases), but the portion of modified reads represent a small fraction of total reads. (PDF) [file pone.0015317.s006.pdf]

3' nucleotide addition

miRNAs	P7 canonical	P7 variant	P7 % variant	P10 canonical	P10 variant	P10 % variant	P14 canonical	P14 variant	P14 % variant
mmu-let-7a-2-5p	333447	92	0.03%	185125	0	0.00%	98375	30	0.03%
mmu-let-7b-5p	549565	120	0.02%	410791	0	0.00%	176895	59	0.03%
mmu-let-7c-1-5p	699775	232	0.03%	492893	0	0.00%	241358	82	0.03%
mmu-let-7c-2-5p	792556	5169	0.65%	527611	0	0.00%	275775	1791	0.65%
mmu-let-7e-5p	308133	954	0.31%	97522	0	0.00%	45983	160	0.35%
mmu-let-7f-2-5p	779320	2385	0.31%	437857	0	0.00%	301811	1243	0.41%
mmu-mir-10b-5p	3215	7	0.22%	1301	0	0.00%	689	3	0.44%
mmu-mir-17-3p	792	10	1.26%	464	0	0.00%	293	3	1.02%
mmu-mir-24-1-3p	1248	5	0.40%	912	0	0.00%	398	0	0.00%
mmu-mir-24-2-3p	1247	4	0.32%	911	0	0.00%	397	0	0.00%
mmu-mir-26a-1-5p	6209	6	0.10%	4204	0	0.00%	2217	0	0.00%
mmu-mir-26a-2-5p	6270	59	0.94%	4259	0	0.00%	2249	33	1.47%
mmu-mir-26b-5p	3401	3	0.09%	2145	0	0.00%	1141	5	0.44%
mmu-mir-30a-5p	7565	6	0.08%	5788	0	0.00%	5882	7	0.12%
mmu-mir-99a-5p	11936	3	0.03%	6245	0	0.00%	3743	3	0.08%
mmu-mir-99b-3p	1672	17	0.99%	509	0	0.00%	251	0	0.00%
mmu-mir-103-1-5p	54312	54	0.10%	38098	0	0.00%	32978	45	0.14%
mmu-mir-103-2-5p	56517	390	0.69%	39005	0	0.00%	34378	479	1.39%
mmu-mir-127-3p	27461	136	0.50%	10153	0	0.00%	3109	19	0.61%
mmu-mir-140-3p	83969	90	0.11%	45691	0	0.00%	23075	59	0.26%
mmu-mir-181a-1-5p	8252	11	0.13%	3368	0	0.00%	1808	3	0.17%
mmu-mir-181b-1-5p	5002	5	0.09%	1713	0	0.00%	883	0	0.00%
mmu-mir-191-5p	9954	0	0.00%	5513	0	0.00%	9206	3	0.03%
mmu-mir-199a-2-3p	76979	58	0.07%	29732	0	0.00%	16386	15	0.09%
mmu-mir-199b-3p	76833	5	0.01%	29698	0	0.00%	16354	0	0.00%
mmu-mir-214-3p	1151	3	0.26%	397	0	0.00%	249	0	0.00%
mmu-mir-320-5p	48550	35	0.07%	20699	0	0.00%	10643	10	0.09%
mmu-mir-340-5p	6713	0	0.00%	4471	0	0.00%	3665	3	0.08%
mmu-mir-341-5p	2419	3	0.12%	931	0	0.00%	211	0	0.00%
mmu-mir-342-5p	441	0	0.00%	342	0	0.00%	175	3	1.71%
mmu-mir-379-5p	4497	27	0.60%	1794	0	0.00%	738	7	0.95%
mmu-mir-449a-5p	41	0	0.00%	27	0	0.00%	493	3	0.61%
mmu-mir-449c-5p	64	0	0.00%	43	0	0.00%	912	27	2.96%
mmu-mir-669c-5p	9480	64	0.67%	10842	0	0.00%	7987	34	0.42%
