# Supplementary material for: Analysis of MicroRNA Expression in the Prepubertal Testis
Source: PLoS One. 2010 Dec 29;5(12):e15317. doi: 10.1371/journal.pone.0015317 (PMC3012074; doi:10.1371/journal.pone.0015317)

Class	Number	Alignment with hairpin sequence	Reads	Run
high confidence	1	GTAGACACAGTTGTCAAAGCCTGGTCACGCAGCATCTTGCGCCTCACGAGGGACTGGCACAAGGGACTCCACAAGCTTGTTCTGTTTTGGCATGGCGATGTTACCTCTCGCAAGCTGTGAGCCTTTGGGCATCAGGCATCCCAGCGTGCTTGGAAGCATGAGCTCTGGGCTGAGGCCCCCTGTCTTCTAC		
		(((((.((((..(((..((((..(......(((.((.(((.((.((((.(((((((((.((((((.(((.(((.((((((...(...((((((....))))))...)..))))))))))))))))))).))..)))...))))..))))...))..))).))))))..))))..)))...))))..)))))		
		…TGTTACCTCTCGCAAGCTGTGAGCCTT***…***		
		…TGTTACCTCTCGCAAGCTGTGAGCCTT…	7	P7-1 +/-
		…	7	P7-1 +/-

		…TGTTACCTCTCGCAAGCTGTGAGCCTT…	14	P10-1 +/-
		…	14	P10-1 +/-

		…TGTTACCTCTCGCAAGCTGTGAGCCTT…	18	P10-2 +/-
		…	18	P10-2 +/-

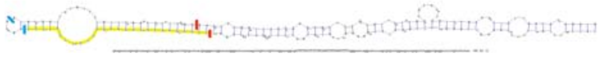

Class	Number	Alignment with hairpin sequence	Reads	Run
high confidence	2	CTGCTCTAGGTCTTAACTGTCCGTCTCAGAACTCACGGTGTCACCTGACTCTGAGCCACCCTGTCATGTGTATTTGTGATGAGTCAAAACCTTAATAAATGGCATTGAGAAGGGAAGTATATGTCACAAGACAGGAGCACCGGGATGGGACAGCCAGTGAGGTACGTGGACAG		
		(((.((((.(((((.((((.(.((((((....((.((((((..((((.((.((((((..((((.(((.((((((((((..((((.......)))))))))).)))))))..))))..))......)))..)).)))).)))))).)))))))).).)))).))).)).)))))))		
		…TAATAAATGGCATTGAGAAGGGAAGT***…***		
		…TAACTGTCCGTCTCAGAACTCACGGTG…	13	P7-1 +/-
		…TAACTGTCCGTCTCAGAACTCACGGTGTC…	12	P7-1 +/-
		…TAACTGTCCGTCTCAGAACTCACGGTGT…	5	P7-1 +/-
		…TAATAAATGGCATTGAGAAGGGAAGT…	12	P7-1 +/-
		…	42	P7-1 +/-

		…TAACTGTCCGTCTCAGAACTCACGGTG…	7	P10-1 +/-
		…TAACTGTCCGTCTCAGAACTCACGGT…	5	P10-1 +/-
		…TAACTGTCCGTCTCAGAACTCACGGTGT…	5	P10-1 +/-
		…TAATAAATGGCATTGAGAAGGGAAGT…	14	P10-1 +/-
		…	31	P10-1 +/-

		…TAACTGTCCGTCTCAGAACTCACGGTGTC…	5	P10-2 +/-
		…TAATAAATGGCATTGAGAAGGGAAGT…	8	P10-2 +/-
		…TATGTCACAAGACAGGAGCACCGGGAT…	6	P10-2 +/-
		…	19	P10-2 +/-

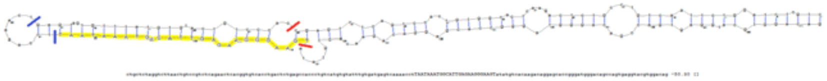

Class	Number	Alignment with hairpin sequence	Reads	Run
non-candidate	3	ATGGCACTTCCATCATGGAGTCACTTGTGCTAAGGTCTCAGAGGCCTACTGAGTGGTTGTTATTGACTATCTTGTTAATAACAAGACTATTAACAGAGGTGGCTAAGAATTGACGGAGAAGTGTAAGGCCTTGAGAGACAAATCACGTGCTGGGGTCCTTGGTTAGTGTGAT		
		((.(((((.(((....(((.((...((((.....(((((.((((((((((...(.((((...(((.((((((((((((((.......)))))))).)))))).)))....)))).)...)))...))))))))..)))))....))))).....)).))).))).)))))).))		
		…TAACAGAGGTGGCTAAGAATTGACGGA***…***		
		…TAACAGAGGTGGCTAAGAATTGACGGA…	8	P7-1 +/-
		…	8	P7-1 +/-

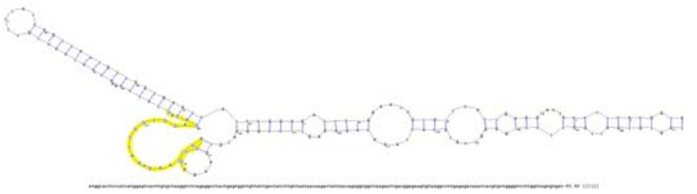

Class	Number	Alignment with hairpin sequence	Reads	Run
potential	4	ACCCATCTGAGACTGAGCTGGAAGCTTTGTCCTTGCTGGCCAGCATGCATAGGACCTGTAGGTGCAGGCGGCTGTGCGGCACAGCACCACTTCTCTGGACCCTGCATGGTACTGTGTCAAGCAAGATGTGCCTACTAGGGCTGGTGAGATGGCTCAGCTGTTGAGGGT		
		((((.((..((.((((((((...(((..(((((.(..(((((((.((((((.(((((((((.((((.((..((((......))))..)).).))).).))))))).))).))))).)).))....)).)))..).))))).)))....)))))))))...))))))		
		…TGCATGGTACTGTGTCAAGCAAGATGT***…***		
		…TGCATAGGACCTGTAGGTGCAGGCGGC…	6	P7-1 +/-
		…TGCATAGGACCTGTAGGTGCAGGCGGCT…	5	P7-1 +/-
		…TGCATGGTACTGTGTCAAGCAAGATGT…	31	P7-1 +/-
		…TGCATGGTACTGTGTCAAGCAAGATGTG…	12	P7-1 +/-
		…TGCATGGTACTGTGTCAAGCAAGATG…	8	P7-1 +/-
		…	62	P7-1 +/-

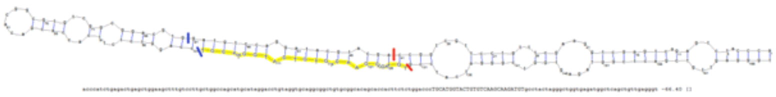

Class	Number	Alignment with hairpin sequence	Reads	Run
high confidence	5	TGCTCATTTGGGGCTGTCTTTATGTTGTTGCTGAGAAAATGACAACTGGATATGATGACTGATTACCTGAGAAATAATTGATGAAATCTCAAGAAAATTCCTCTAGATAGTCAAGTTCTGATCCAGCTATGTCAACTCAAAGCAGCAACCTTGATTGCCCTCTGAGTA		
		(((((...(((((.(((.....((((((((..((...(((((.((((((.....(((((((((.(((((.(((..((((.(....)))))....))).))).)).))))).))))...))))))...)))))..))..)))))))))...))).))))).))))))		
		…GGATATGATGACTGATTACCTGAGA***…***		
		…GGATATGATGACTGATTACCTGAGA…	19	P7-1 +/-
		…TGGATATGATGACTGATTACCTGAGA…	15	P7-1 +/-
		…TGGATATGATGACTGATTACCTGAGAAA…	8	P7-1 +/-
		…TGGATATGATGACTG…	5	P7-1 +/-
		…	47	P7-1 +/-

		…TGGATATGATGACTGATTACCTGAGA…	17	P7-3 -/-
		…GGATATGATGACTGATTACCTGAGA…	10	P7-3 -/-
		…TGGATATGATGACTGA…	7	P7-3 -/-
		…TGGATATGATGACTGATTACCTGAGAAA…	6	P7-3 -/-
		…GGATATGATGACTGATTACCTGAGAAA…	5	P7-3 -/-
		…TGGATATGATGACT…	5	P7-3 -/-
		…TGGATATGATGACTG…	5	P7-3 -/-
		…	55	P7-3 -/-

		…GGATATGATGACTGATTACCTGAGA…	22	P7-4 -/-
		…TGGATATGATGACTGATTACCTGAGA…	13	P7-4 -/-
		…TGGATATGATGACTGATTACCTGAGAAA…	9	P7-4 -/-
		…TGGATATGATGACTGA…	8	P7-4 -/-
		…TGGATATGATGACTG…	7	P7-4 -/-
		…	59	P7-4 -/-

		…GGATATGATGACTGATTACCTGAGA…	10	P10-1 +/-
		…TGGATATGATGACTGATTACCTGAGA…	10	P10-1 +/-
		…TGGATATGATGACTG…	5	P10-1 +/-
		…	25	P10-1 +/-

		…TGGATATGATGACTGATTACCTGAGA…	10	P10-2 +/-
		…TGGATATGATGACTGA…	7	P10-2 +/-
		…GGATATGATGACTGATTACCTGAGA…	5	P10-2 +/-
		…TGGATATGATGACTGATTACCTGAGAAA…	5	P10-2 +/-
		…	27	P10-2 +/-

		…TGGATATGATGACTGATTACCTGAGA…	17	P10-3 -/-
		…GGATATGATGACTGATTACCTGAGA…	12	P10-3 -/-
		…TGGATATGATGACTGATTACCTGAGAAA…	10	P10-3 -/-
		…TGGATATGATGACTG…	9	P10-3 -/-
		…	48	P10-3 -/-

		…GGATATGATGACTGATTACCTGAGA…	20	P10-4 -/-
		…TGGATATGATGACTGATTACCTGAGA…	17	P10-4 -/-
		…TGGATATGATGACTGATTACCTGAGAAA…	13	P10-4 -/-
		…TGGATATGATGACTGA…	8	P10-4 -/-
		…TGGATATGATGACTG…	7	P10-4 -/-
		…TGGATATGATGACT…	5	P10-4 -/-
		…	70	P10-4 -/-

		…GGATATGATGACTGATTACCTGAGA…	15	P14-1 +/-
		…TGGATATGATGACTGATTACCTGAGAAA…	15	P14-1 +/-
		…TGGATATGATGACTGA…	8	P14-1 +/-
		…TGGATATGATGACT…	7	P14-1 +/-
		…TGGATATGATGACTG…	7	P14-1 +/-
		…TGGATATGATGACTGATTACCTGAGA…	5	P14-1 +/-
		…	57	P14-1 +/-

		…GGATATGATGACTGATTACCTGAGA…	9	P14-3 -/-
		…TGGATATGATGACTG…	9	P14-3 -/-
		…TGGATATGATGACTGATTACCTGAGA…	9	P14-3 -/-
		…TGGATATGATGACTGA…	6	P14-3 -/-
		…	33	P14-3 -/-

		…GGATATGATGACTGATTACCTGAGA…	13	P14-4 -/-
		…TGGATATGATGACTGATTACCTGAGA…	12	P14-4 -/-
		…TGGATATGATGACTGATTACCTGAGAAA…	9	P14-4 -/-

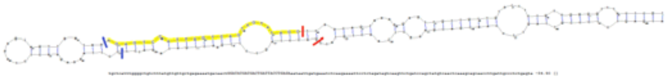

```
..........................................TGGATATGATGACTG...........................................................  7 P14-4 -/-
..........................................TGGATATGATGACTGATTACCTGA..................................................  6 P14-4 -/-
..........................................TGGATATGATGACTGA..........................................................  5 P14-4 -/-
.....................................................................................................................  52 P14-4 -/-
----------------------------------------------------------------------------------------------------
```

high confidence

```
6 GCAGCATCAGGGAGGTTGAGAACCACTGCTCTGTAGGCCTCCTGGTTGAATTCAGGCTGTCTCGAGTAAGGATGGTTTCTCCTAACTGTCCCAAGTCTAGAAGAGTAGAAAGCGATCAGGGTGGAGGAGGGAGAGTTCCCGAGTTCTCTGTGGCTGT
  ((((..(((((((.(((.((((.....((((.(...((((((((((..(((..(((.(((.((.(..((((((((......))))))))..).)).))).)))..))).))).)))))))))).))...).)))).)))).))).)))))))..)))))
  **************************************************************************************TAGAAGAGTAGAAAGCGATCAGGGTG***********************************  5 P7-1 +/-
  ......................................................................................TAGAAGAGTAGAAAGCGATCAGGGTG...................................  5 P7-1 +/-
  ...................................................................................................................................................
  ----------------------------------------------------------------------------------------------------
```

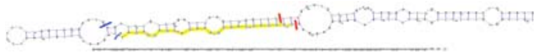

high confidence

```
7 GCATATCTGGCTCTCACCGACAGGGTTCAGTAGTGTTGGTGTCTCCACAGAGCGTGGAACATGATGAAGCCAGTGACCTTGAGGTGAGACGTGCTTCAAATTCATGCATCTTGGAGAACGGACACCAGCTGGATGCAGGTAGGAGCCTATGGC
  .((((...(((((..(((....(.(((((((.(((((.((.((((((.((((((((((......(((((((..(.(((....))).)...).))))))..))))))).)))))))))))).))))).)..))))))).).)))..))))))))..
  *******************************************************************************************TTCATGCATCTTGGAGAACGGACACC*************************  5 P10-1 +/-
  .......................................................................................TTCATGCATCTTGGAGAACGGACACC.........................  5 P10-1 +/-
  ............................................................................................................................................
  ----------------------------------------------------------------------------------------------------
  .......................................................................................TTCATGCATCTTGGAGAACGGACACC.........................  5 P10-2 +/-
  ............................................................................................................................................  5 P10-2 +/-
  ----------------------------------------------------------------------------------------------------
```

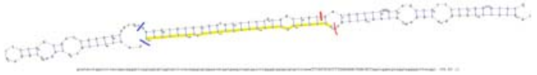

high confidence

```
8 GCACGGTCCGAGATGGCTGGCGGTAAGGCTGGAAAGGACTCCGGAAAGGCCAAGACAAAGGCGGTTTCCCGCTCGCAGCGAGCCGGCTTGTAGTTCCCTTTGGGCTGTATTCCATCGACACCTGAAATCTAGGACAACCAGCCACGGACGTGT
  (((((.((((...((((((..((....(((((.(((..((.((((((.(((((((.(((.(((((...(((.....)))))))))).)))..))).....)))).))...))))...)).))).....))))).))..)))))))))))))))
  ..........................TGGAAAGGACTCCGGAAAGGCCAAGAC.....................................................................................  6 P7-1 +/-
  ..........................................................................................................................................  6 P7-1 +/-
  ----------------------------------------------------------------------------------------------------
  **************************TGGAAAGGACTCCGGAAAGGCCAAGAC*************************************************************************************
  ..........................TGGAAAGGACTCCGGAAAGGCCAAGAC.....................................................................................  8 P10-1 +/-
  ..........................................................................................................................................  8 P10-1 +/-
  ----------------------------------------------------------------------------------------------------
  ..........................TGGAAAGGACTCCGGAAAGGCCAAGAC.....................................................................................  6 P10-2 +/-
  ..........................................................................................................................................  6 P10-2 +/-
  ----------------------------------------------------------------------------------------------------
```

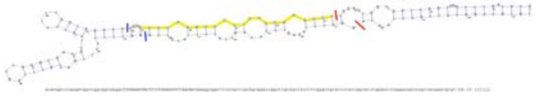

high confidence

```
9 GACGTGAGAGAGGTTGCTGGGTGTGTGAATAAACAGAGTAGTAATCGGAAGCCTCAGGAGTTGGGTCTTGAGTTCCTTACACTGTAGACTAGCACCTGACAATGCCAGATTCTTATTTTACCATTTAAAACTCAGTACCCCTGTCTAATGTC
  (((((.(((.(((.((((((((...(((((....((((((((.((((((.....(((((.((((((.(((..(((.......)))..))))))))).)))))).....)).))))).))))))))...)))))..))))))))..))).))).)))))
  **********************TGAATAAACAGAGTAGTAATCGGAAGCCT**********************************************************************************  7 P7-1 +/-
  ......................TGAATAAACAGAGTAGTAATCGGAAGCCT..................................................................................  7 P7-1 +/-
  .....................................................................................................................................
  ----------------------------------------------------------------------------------------------------
```

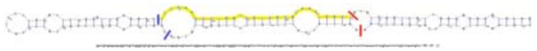

non-candidate

```
10 GTTTGCCAGCCAGGTACAGACTCCTCAAAGGTAGCAGACTTGCTGATGGTTGCTGCTGTAGGAGCCCTCAGTTTCTTCCTGGGCTGAGTCTACAGTAGTAGGCTGAGTCAGGGCGTCGCAGGGCCTGTAGGGAGCACCTGTGCTGGGGAGC
   ((((.((((((((((...(.(((((...((((.((.(((.(.((((((.((((((((((((((((((.............)))))...))))))))))))).)...))))).).)))))...))))...))))))))))).))))).))))
   ******************************************************************************************TACAGTAGTAGGCTGAGTCAGGGCGTC********************************
   .....................TCAAAGGTAGCAGACTTGCTGATGGTT................................................................................................  6 P7-1 +/-
   .........................................................................................TACAGTAGTAGGCTGAGTCAGGGCGTC............................  24 P7-1 +/-
   ..................................................................................................................................................  30 P7-1 +/-
   ----------------------------------------------------------------------------------------------------
   .....................TCAAAGGTAGCAGACTTGCTGATGGTT................................................................................................  15 P10-1 +/-
   .........................................................................................TACAGTAGTAGGCTGAGTCAGGGCGTC............................  8 P10-1 +/-
   ..................................................................................................................................................  23 P10-1 +/-
   ----------------------------------------------------------------------------------------------------
   .....................TCAAAGGTAGCAGACTTGCTGATGGTT................................................................................................  13 P10-2 +/-
   .........................................................................................TACAGTAGTAGGCTGAGTCAGGGCGTC............................  8 P10-2 +/-
   ..................................................................................................................................................  21 P10-2 +/-
   ----------------------------------------------------------------------------------------------------
```

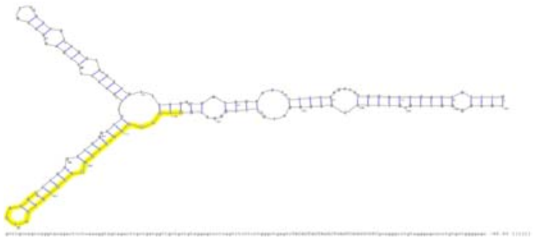

high confidence

```
11 ATGGATCTGCAGCTGCTTGGCTTACTTTTTGATTTGTGTGAGCAAAACCGGCGCCTTTCTCCACCACTTTACCTTTAGCTTGTGTTTCAGTATTGAAAGTCTGCCTTTTTGTACACAACATGTGGTTAGGTCTCACTGCTGTTTAATCCAT
   ((((((..(((((.....((((((((...((..((((((..((((((..((((.(((((...(((((..............))).....))...)))))..)))).)))))))))))))))...)).)))))).....)))))...))))))
   ***********************TTTGATTTGTGTGAGCAAAACCGGCGCCTT*********************************************************************************  6 P14-1 +/-
   .......................TTTGATTTGTGTGAGCAAAACCGGCGCCTT.................................................................................  5 P14-1 +/-
   .........................TGATTTGTGTGAGCAAAACCGGCGCCTTT................................................................................  11 P14-1 +/-
   ......................................................................................................................................
   ----------------------------------------------------------------------------------------------------
```

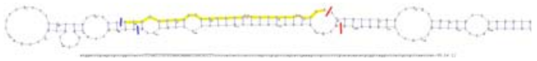

potential

```
12 CAATGTTTGTAGGCAAGTCCACTGCTGTCACTGTCCTTTGTCTCCCATAGCCCCTTCTGAGCTCTCCTGTGCCCTTGAGCTTTGGGGCTATTTGGAGTGTAGAATGGGTGTTTTGTGAAACTGGACCAGTCTGCCTTGCCATGAGCTGTTG
   ((((((..(.((((((..((..((((.....((((...((.((.((.(((((((((((.(((((.....(((((........)))))).....))))).))))).))).))).)).)).)).))))))))).)).)))))))..))).))))
   ******************************************************************************************TTTGGAGTGTAGAATGGGTGTTTTGT*******************************  5 P7-1 +/-
   ..........................................................................................TTTGGAGTGTAGAATGGGTGTTTTGT...............................  5 P7-1 +/-
   ...................................................................................................................................................
   ----------------------------------------------------------------------------------------------------
   ..........................................................................................TTTGGAGTGTAGAATGGGTGTTTTGT...............................  6 P10-1 +/-
   ...................................................................................................................................................  6 P10-1 +/-
   ----------------------------------------------------------------------------------------------------
   ..........................................................................................TTTGGAGTGTAGAATGGGTGTTTTGT...............................  5 P10-2 +/-
   ...................................................................................................................................................  5 P10-2 +/-
   ----------------------------------------------------------------------------------------------------
```

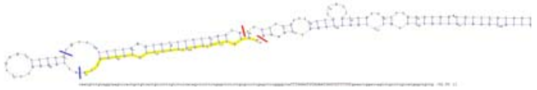

non-candidate

```
13 GCCAAATCCCCCTTGATTTGGAAGCCCCTTGAGTTATAAAAGTCTTGTCTTCCTGCGTCCAGTGCTAATCTCTCTAACCCAGCCTTAGAAGAAGGCAGTAAATGTACACGCAAAAAGCTTGTTTTAATTGCTTCCTTTAATTGATTTGGT
   ((((((((....((((...((((((......(((((...((((.((((.....(((((..(.((((..(((.((((((.......))))))).)))).)..)))))...))))...))))...)))))))))))).)))).))))))))
   *************************************************************************************TAGAAGAAGGCAGTAAATGTACACGCA***********************************  7 P14-1 +/-
   .....................................................................................TAGAAGAAGGCAGTAAATGTACACGCA...................................  7 P14-1 +/-
   .....................................................................................TAGAAGAAGGCAGTAAATGTACACGCAA..................................  14 P14-1 +/-
   .................................................................................................................................................
   ----------------------------------------------------------------------------------------------------
```

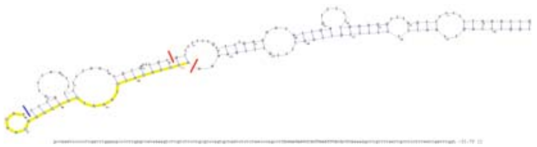

high confidence

```
14 GGGGCACTGCTGCCATTCTGGGCACCCGAGTGTCTTCTCTATGATCAAAAGTCCTGGAACGCGTTATGAATCCTAGTTACGTATGGCTTTTGGATTGGTTCTTGGTAAACTGAAGATTATTTTTGTGGTGAATGAATTAAACAGGCCCC
   (((((.(((.....((((....((((((((.((((((......((((.(((((.((((.((..((((((((....))).)))))..)))))))))))).....))))).....)))))).....)))).)))))...)))).....))))))))
   ***************************************************************************************TTGGATTGGTTCTTGGTAAACTGAAGA*********************************  5 P7-1 +/-
   ..........................................TGATCAAAAGTCCTGGAACGCGTTAT..................................................................................  12 P7-1 +/-
   ........................................................................................TTGGATTGGTTCTTGGTAAACTGAAGA.................................  8 P7-1 +/-
   ........................................................................................TTGGATTGGTTCTTGGTAAACTGAAGATT...............................  25 P7-1 +/-
   ......................................................................................................................................................
   ----------------------------------------------------------------------------------------------------
```

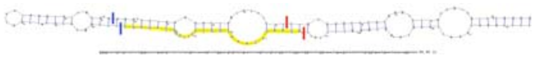

high confidence

```
15 CCGGGAAGACCGTTGTCTGGCATGGACTGAGCAGATGTGGAATAGGAGAAGGCAGAGGTAAAAAGTGGTATTTGGATATTCCTTGGCTGAATCTCCTGTAGCACCATCACCTTCTCTGGTTGGAGCACAGAGGTCTGACTCCCAGG
   ..((((((((....((((((.(.(((((((.((.(((((.((((((((.(((.((((.......................)))).)))...)))))))))....))).)).)).))).)))).).)).)))))))))...)))).. 
   ***************************TGAGCAGATGTGGAATAGGAGAAGGC**************************************************************************************  8 P7-1 +/-
   ...........................TGAGCAGATGTGGAATAGGAGAAGGC..............................................................................................  5 P7-1 +/-
   ......................TGGACTGAGCAGATGTGGAATAGGAGA..................................................................................................  13 P7-1 +/-
   ...................................................................................................................................................
   ----------------------------------------------------------------------------------------------------
```

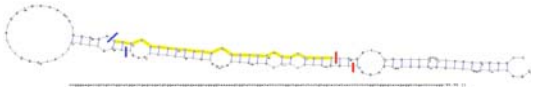

high confidence

```
16  GTCTTTGTAAATTCTCTGTTTAGCTCTGTATCCCATTTTTTGATTGGTTTTAGAAGCTTCTTGAGTTCTTTATTTATATTGGAGATAGATAGATAGATGATAGATAGATAAATAAATGGATACTGGATGAGGGTTAGTGAAGAT
    ((((..(.(((((((..(((((.....((((((.((((....(((...((((....(((((.....((((((.......))))))).....))).)).....)))))..)))....))))))))))))))..))))))).)..)))))
    ***TAGATAGATAAATAAATGGATACTGGAT***
    ...TAGATAGATAAATAAATGGATACTGGAT...                          7 P7-1 +/-
    ...                                                          7 P7-1 +/-
    ---
    ...TAGATAGATAAATAAATGGATACTGGAT...                          5 P10-1 +/-
    ...                                                          5 P10-1 +/-
    ---
```

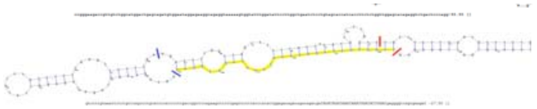

non-candidate

```
17  CATGAAGGTCATGGGTCTGTGTGTCTATGAAGTGAGTCTGTCTACTGTGAGCTGATCGTAAGAACTCTGCTGTGCTGCATCCCACTAGGCCAGCAGCTTATGTTCAAGGGGACGATGATTATGTGACATGATTTGTGACTCTGTG
    .....((((((..(((...((((((((((.....(((.(((..((.(((((....(((((....((((((.(((...........)))))))))))))))))))..))))))..))))).)))))))..)))))).))...
    ***TGTTCAAGGGGACGATGATTATGTGA***
    ...TGTTCAAGGGGACGATGATTATGTGA...                            7 P7-1 +/-
    ...                                                          7 P7-1 +/-
    ---
```

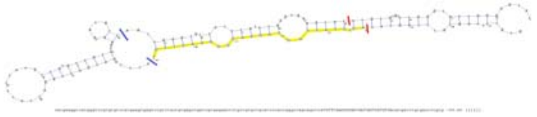

potential

```
18  AGCAGGATGAGCGTCTTGGTTTCTCAGCTCAGGAGTGTGTTGGGGTTTGATTGCTTCTGATACGGGTCACCCGGACACATCAGTGGTAGGACAGGACCAGTAGCTCCCAGCAGCCAGGGAGGCCAAGGCCATCTCCTTCCTCCT
    ((.((((.(((.(((((((((((((.((((.(((((...((((..((((.(((((.(((((.((((...))))).....))))).)))))..)))).))))..))))).)..).)))..)))))))))))...)))..)))).))
    ***TGGGGTTTGATTGCTTCTGATACGGGT***
    ...TGGGGTTTGATTGCTTCTGATACGGGT...                           5 P10-1 +/-
    ...                                                          5 P10-1 +/-
    ---
```

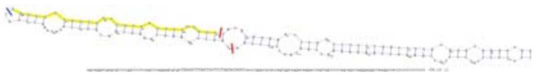

high confidence

```
19  GGCGCAGCTCCATGAGCCTGTGAGTGGCCAGGGTGGGGCTGGGTGGTGCAGGCAGGAGAGCCATTGATCTATTGTGGGCTCTACTCCCTGCCCCAGCCAGAGCACCCCATTGGCTACCCACAGGTACACAGCAGCCAGCGTC
    ((((.((((..((.(((((((.((((((((((((...((((.(((.(((((.((.((((((.............)))))).)).))))).))).))))..)))))...))))))).))))))).))..).)))..)))))
    ***GAGCACCCCATTGGCTACCCACA***
    ...GGTGGTGCAGGCAGGAGAGC...                                   6 P7-1 +/-
    ...GGTGGTGCAGGCAGGAGAGCC...                                  5 P7-1 +/-
    ...GGTGGTGCAGGCAGGAGAGCCA...                                 5 P7-1 +/-
    ...GAGCACCCCATTGGCTACCCACA...                                8 P7-1 +/-
    ...GAGCACCCCATTGGCTACCCACAG...                               8 P7-1 +/-
    ...                                                          32 P7-1 +/-
    ---
    ...GGTGGTGCAGGCAGGAGAGC...                                   5 P7 +/-
    ...                                                          5 P7-2 +/-
    ---
    ...GGTGGTGCAGGCAGGAGAGC...                                   17 P7-3 -/-
    ...GGTGGTGCAGGCAGGAGAGCC...                                  11 P7-3 -/-
    ...GGTGGTGCAGGCAGGAGAGCCA...                                 11 P7-3 -/-
    ...GGTGGTGCAGGCAGGAGAGCCAT...                                6 P7-3 -/-
    ...GAGCACCCCATTGGCTACCCACA...                                22 P7-3 -/-
    ...GAGCACCCCATTGGCTACCCACAG...                               9 P7-3 -/-
    ...GAGCACCCCATTGGCTACCCAC...                                 5 P7-3 -/-
    ...                                                          81 P7-3 -/-
    ---
    ...GGTGGTGCAGGCAGGAGAGC...                                   12 P7-4 -/-
    ...GGTGGTGCAGGCAGGAGAGCC...                                  10 P7-4 -/-
    ...GGTGGTGCAGGCAGGAGAGCCA...                                 9 P7-4 -/-
    ...GAGCACCCCATTGGCTACCCACA...                                17 P7-4 -/-
    ...GAGCACCCCATTGGCTACCCACAG...                               9 P7-4 -/-
    ...GAGCACCCCATTGGCTACCCAC...                                 5 P7-4 -/-
    ...                                                          62 P7-4 -/-
    ---
    ...GGTGGTGCAGGCAGGAGAGC...                                   5 P10-3 -/-
    ...                                                          5 P10-3 -/-
    ---
    ...GAGCACCCCATTGGCTACCCACA...                                6 P10-4 -/-
    ...                                                          6 P10-4 -/-
    ---
    ...GAGCACCCCATTGGCTACCCACA...                                5 P14-4 -/-
    ...                                                          5 P14-4 -/-
    ---
```

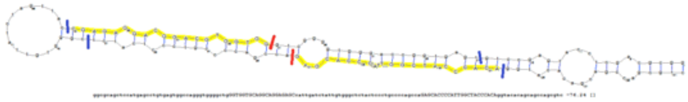

high confidence

```
20  GCGGTTTTTGGTTTTTGGTTTTGGTTTTTGGTTTTTGGTTTATGGTTTTGGAGACAGGGCTTCTCTGTGTAGCCCTAACTGTCCTGGAATTCACTCTATAGAGCAGGCTGGCCTCGAACTCATAAATCCACCTGCCTCTGC
    ((((.....(((...(((...((((((..((((((((.((((((((((..(.(((((((((.(.....).)))))....)))))..)))......)))))))).))))..))))..))))).))......)))...))).))))
    ***GAGCAGGCTGGCCTCGAACTC***
    ...GAGCAGGCTGGCCTCGAACTC...                                  4868 P7-3 -/-
    ...GAGCAGGCTGGCCTCGAACTCA...                                 2221 P7-3 -/-
    ...GAGCAGGCTGGCCTCGAACT...                                   1093 P7-3 -/-
    ...AGCAGGCTGGCCTCGAACTC...                                   651 P7-3 -/-
    ...AGCAGGCTGGCCTCGAACTCA...                                  387 P7-3 -/-
    ...TAGAGCAGGCTGGCCTCGAACT...                                 344 P7-3 -/-
    ...TAGAGCAGGCTGGCCTCGAA...                                   286 P7-3 -/-
    ...GAGCAGGCTGGCCTCGAAC...                                    282 P7-3 -/-
    ...TAGAGCAGGCTGGCCTCGA...                                    282 P7-3 -/-
    ...TAGAGCAGGCTGGCCTCGAAC...                                  187 P7-3 -/-
    ...GAGCAGGCTGGCCTCGAACTCAT...                                125 P7-3 -/-
    ...AGAGCAGGCTGGCCTCGAACTC...                                 97 P7-3 -/-
    ...AGCAGGCTGGCCTCGAACT...                                    94 P7-3 -/-
    ...AGAGCAGGCTGGCCTCGAACT...                                  75 P7-3 -/-
    ...GAGCAGGCTGGCCTCGAA...                                     55 P7-3 -/-
    ...AGAGCAGGCTGGCCTCGAAC...                                   31 P7-3 -/-
    ...AGAGCAGGCTGGCCTCGAACTCA...                                31 P7-3 -/-
    ...TAGAGCAGGCTGGCCTCG...                                     22 P7-3 -/-
    ...AGAGCAGGCTGGCCTCGAA...                                    19 P7-3 -/-
    ...TAGAGCAGGCTGGCCTCGAACTC...                                19 P7-3 -/-
    ...AGCAGGCTGGCCTCGAACTCAT...                                 17 P7-3 -/-
    ...GCAGGCTGGCCTCGAACTCA...                                   16 P7-3 -/-
    ...AGCAGGCTGGCCTCGAAC...                                     10 P7-3 -/-
    ...GAGCAGGCTGGCCTCGA...                                      10 P7-3 -/-
    ...GCAGGCTGGCCTCGAACTC...                                    10 P7-3 -/-
    ...GAGCAGGCTGGCCTCGAACTCATA...                               7 P7-3 -/-
    ...AGCAGGCTGGCCTCGAA...                                      6 P7-3 -/-
    ...AGAGCAGGCTGGCCTCGA...                                     5 P7-3 -/-
    ...                                                          11250 P7-3 -/-
    ---
    ...GAGCAGGCTGGCCTCGAACTC...                                  1780 P7-4 -/-
    ...GAGCAGGCTGGCCTCGAACTCA...                                 724 P7-4 -/-
    ...GAGCAGGCTGGCCTCGAACT...                                   390 P7-4 -/-
    ...AGCAGGCTGGCCTCGAACTC...                                   178 P7-4 -/-
    ...AGCAGGCTGGCCTCGAACTCA...                                  120 P7-4 -/-
    ...TAGAGCAGGCTGGCCTCGA...                                    97 P7-4 -/-
    ...GAGCAGGCTGGCCTCGAAC...                                    96 P7-4 -/-
    ...TAGAGCAGGCTGGCCTCGAA...                                   90 P7-4 -/-
    ...TAGAGCAGGCTGGCCTCGAACT...                                 89 P7-4 -/-
    ...TAGAGCAGGCTGGCCTCGAAC...                                  75 P7-4 -/-
    ...GAGCAGGCTGGCCTCGAACTCAT...                                41 P7-4 -/-
    ...AGAGCAGGCTGGCCTCGAACTC...                                 27 P7-4 -/-
    ...AGAGCAGGCTGGCCTCGAACT...                                  26 P7-4 -/-
    ...AGCAGGCTGGCCTCGAACT...                                    18 P7-4 -/-
    ...GAGCAGGCTGGCCTCGAA...                                     16 P7-4 -/-
    ...AGAGCAGGCTGGCCTCGAAC...                                   15 P7-4 -/-
    ...AGAGCAGGCTGGCCTCGAACTCA...                                15 P7-4 -/-
    ...GCAGGCTGGCCTCGAACTC...                                    12 P7-4 -/-
    ...TAGAGCAGGCTGGCCTCG...                                     12 P7-4 -/-
    ...TAGAGCAGGCTGGCCTCGAACTC...                                10 P7-4 -/-
    ...GAGCAGGCTGGCCTCGA...                                      9 P7-4 -/-
    ...AGCAGGCTGGCCTCGAACTCAT...                                 6 P7-4 -/-
    ...GAGCAGGCTGGCCTCGAACTCATA...                               6 P7-4 -/-
    ...                                                          3852 P7-4 -/-
    ---
    ...GAGCAGGCTGGCCTCGAACTC...                                  6930 P10-3 -/-
    ...GAGCAGGCTGGCCTCGAACTCA...                                 1990 P10-3 -/-
    ...GAGCAGGCTGGCCTCGAACT...                                   1643 P10-3 -/-
    ...AGCAGGCTGGCCTCGAACTC...                                   799 P10-3 -/-
```

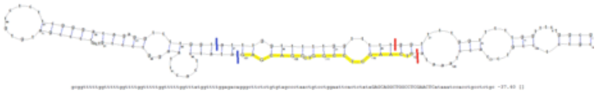

```
TAGAGCAGGCTGGCCTCGAACT            460   P10-3 -/-
GAGCAGGCTGGCCTCGAAC               449   P10-3 -/-
TAGAGCAGGCTGGCCTCGAA              413   P10-3 -/-
AGCAGGCTGGCCTCGAACTCA             331   P10-3 -/-
TAGAGCAGGCTGGCCTCGA               324   P10-3 -/-
TAGAGCAGGCTGGCCTCGAAC             299   P10-3 -/-
AGCAGGCTGGCCTCGAACT               114   P10-3 -/-
GAGCAGGCTGGCCTCGAACTCAT           110   P10-3 -/-
AGAGCAGGCTGGCCTCGAACT              89   P10-3 -/-
GAGCAGGCTGGCCTCGAA                 80   P10-3 -/-
AGAGCAGGCTGGCCTCGAACTC             79   P10-3 -/-
AGAGCAGGCTGGCCTCGAAC               43   P10-3 -/-
AGAGCAGGCTGGCCTCGAACTCA            39   P10-3 -/-
TAGAGCAGGCTGGCCTCG                 22   P10-3 -/-
TAGAGCAGGCTGGCCTCGAACTC            22   P10-3 -/-
AGAGCAGGCTGGCCTCGAA                18   P10-3 -/-
AGCAGGCTGGCCTCGAACTCAT             17   P10-3 -/-
GCAGGCTGGCCTCGAA                   14   P10-3 -/-
GCAGGCTGGCCTCGAACTCA               13   P10-3 -/-
AGCAGGCTGGCCTCGAA                  12   P10-3 -/-
AGAGCAGGCTGGCCTCGA                 11   P10-3 -/-
GAGCAGGCTGGCCTCG                    9   P10-3 -/-
GCAGGCTGGCCTCGAAC                   8   P10-3 -/-
GCAGGCTGGCCTCGAACTC                 7   P10-3 -/-
GAGCAGGCTGGCCTC                     6   P10-3 -/-
GAGCAGGCTGGCCTCGA                   6   P10-3 -/-
AGCAGGCTGGCCTCGAAC                  5   P10-3 -/-
GAGCAGGCTGGCCTCGAACTCATA            5   P10-3 -/-
                                14367   P10-3 -/-

GAGCAGGCTGGCCTCGAACTC            6144   P10-4 -/-
GAGCAGGCTGGCCTCGAACTCA           2086   P10-4 -/-
GAGCAGGCTGGCCTCGAACT             1491   P10-4 -/-
AGCAGGCTGGCCTCGAACTC              718   P10-4 -/-
GAGCAGGCTGGCCTCGAAC               367   P10-4 -/-
AGCAGGCTGGCCTCGAACTCA             358   P10-4 -/-
TAGAGCAGGCTGGCCTCGAACT            285   P10-4 -/-
TAGAGCAGGCTGGCCTCGAA              272   P10-4 -/-
TAGAGCAGGCTGGCCTCGA               206   P10-4 -/-
TAGAGCAGGCTGGCCTCGAAC             186   P10-4 -/-
GAGCAGGCTGGCCTCGAACTCAT           119   P10-4 -/-
AGAGCAGGCTGGCCTCGAACT             110   P10-4 -/-
AGCAGGCTGGCCTCGAACT                90   P10-4 -/-
AGAGCAGGCTGGCCTCGAACTC             85   P10-4 -/-
GAGCAGGCTGGCCTCGAA                 67   P10-4 -/-
AGAGCAGGCTGGCCTCGAAC               45   P10-4 -/-
AGAGCAGGCTGGCCTCGAACTCA            37   P10-4 -/-
AGCAGGCTGGCCTCGAACTCAT             19   P10-4 -/-
TAGAGCAGGCTGGCCTCGAACTC            19   P10-4 -/-
AGAGCAGGCTGGCCTCGAA                17   P10-4 -/-
GCAGGCTGGCCTCGAACTCA               16   P10-4 -/-
AGCAGGCTGGCCTCGAAC                 14   P10-4 -/-
TAGAGCAGGCTGGCCTCG                 13   P10-4 -/-
AGCAGGCTGGCCTCGAA                   9   P10-4 -/-
GCAGGCTGGCCTCGAACTC                 9   P10-4 -/-
GAGCAGGCTGGCCTCGA                   8   P10-4 -/-
GAGCAGGCTGGCCTCGAACTCATA            8   P10-4 -/-
GCAGGCTGGCCTCGAAC                   8   P10-4 -/-
GCAGGCTGGCCTCGAA                    6   P10-4 -/-
AGAGCAGGCTGGCCTCGA                  5   P10-4 -/-
GAGCAGGCTGGCCTCG                    5   P10-4 -/-
                                12822   P10-4 -/-
```

non-candidate

```
21 ATAGATTCTGTCAAATCAAGAAGAAATAACTGGTCACCAGTTGCTTGTTAGTGTCTCTGGGCTAAGCAGTGCTTAATCACATTGACTAACCTCAGCGAGTCTGTGGATGAGTAATCTCTACTGGAACAAAGGTTTCTGT
   ((((..((......((.((.(((.((.(((.(((..(((..(((((((((.(((..(((..(((((...)))))..).))..)))....)).)))))))))..))).))).)).))).)).)).....))..)))))
   TGCTTGTTAGTGTCTCTGGGCTAAGCAG           5   P7-1 +/-
                                          5   P7-1 +/-
```

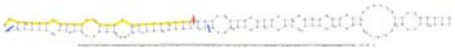

potential

```
22 GTAGTGATTAAGAAGAGACCAGCAGGCTGGAGAGATGGCTCAGCTGTTAAAGGCTAGGCTCACAACCAAAAATATAAGAGTTCAGTTCCCAGCACCCACGGCTGTCTCTCCAGCCACCTTTTTTGCCTTATACCACAC
   ...(((..((((..((((..((..(((((((((((((((..((((.....((((..((((.................))))..))))..))))......)))))))))))))))..)).))))..))))...)))..
   CACGGCTGTCTCTCCAGCCACCTTTTT            5   P14-1 +/-
                                          5   P14-1 +/-
```

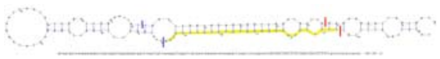

high confidence

```
23 TGTCCTGCTTGAGTTCCAGTCCTGACTTCCTTGGTGATGAACAGCAGTATGGAAGGGTAAGCCGAATAAATCCTTTCCTCCCCAAACTTGCTTCTTGGTCATGATGTTTGTGCAGGAATAGAAACCCTGGCTAAGACA
   .(((..(((....(((...(((((((.....(.(((((.((.((((((.(((..(((.(((..((.....))))).)))..))).(((.(((..((.....))))).)))..))).)).)))).)).))))).).....)).)))))...)))......)))...))).
   TTGGTCATGATGTTTGTGCAGGAATA
   TTGGTGATGAACAGCAGTATGGAAGGGTA          6   P10-1 +/-
   TTGGTCATGATGTTTGTGCAGGAATA            26   P10-1 +/-
   TTGGTCATGATGTTTGTGCAGGAATAGA          24   P10-1 +/-
   TTGGTCATGATGTTTGTGCAGGAATAG           15   P10-1 +/-
   TTGGTCATGATGTTTGTGCAGGAAT              7   P10-1 +/-
                                         78   P10-1 +/-
```

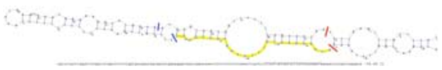

potential

```
24 GCCTGGCCTGCTTTGCCAACATGCCTGTGGAGAGCGAGCTGGAGCGAGGCGCTCGGATGAAGTCTGTGGGCATCCTCTCTCCATCCTACACCCTGCTCTACCGCTACATGCACTTTTTGGAGAACCAGGTTCGGT
   (((..(((((.((..((((((((...((((((((((((.((((.((((.(((((.(.(....)).))))))..)))).)))))..(.(.(....)).))))))..)))).))))))..))......)))))).)))).)))).......))))..)).))))))..)))
   TGCCAACATGCCTGTGGAGAGCGAGC
   TGCCAACATGCCTGTGGAGAGCGAGC             8   P7-1 +/-
                                          8   P7-1 +/-
   TGCCAACATGCCTGTGGAGAGCGAGC             8   P10-1 +/-
                                          8   P10-1 +/-
   TGCCAACATGCCTGTGGAGAGCGAGC             5   P10-2 +/-
                                          5   P10-2 +/-
```

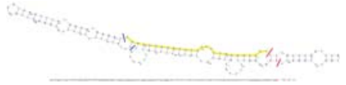

potential

```
25 GAGGTCTTCTGCAGGTCTATGCTGATGCTTCTCTGGTTTTCCCCTTGCTGGTGGCTGAGACATTCGCCCAAAAGGCAGATGCCTTCAGAGCTGAGAAGAATGAAGACTAAGAAGATTGGTAAAGACTGAGGCTTT
   ((((((((.....(((((.(((..((.(((((.((((((((..(((.(((((..(((((.(((..(((.....)))..)))..)))))).(((((.(((..(((.....)))..)))..)))))).))).)))))...)))))))))))))))..))).)))))))))))))
   TGAAGACTAAGAAGATTGGTAAAGACTG
   TGAAGACTAAGAAGATTGGTAAAGACTG           6   P10-1 +/-
                                          6   P10-1 +/-
```

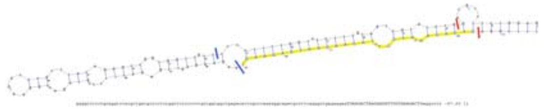

```
non-candidate   26  GCTGGGCAGCCCAGGTATAGTTAGTTTTAGGCTTGTAGGTTTACTTCCTAGTTTCTTTAAACACACCTGATGGTAAAGAACACATTGAAAGTACTGCAGGTTGCAGTTGACAGTACCCACATGGTCTGCAAAAGC
                        (((..((((.((((((((.((((..(...(((((((((...(((((.(..((((((((......(((....))))))))).))...).))))))))))))))..)..)))).)))))....))).))))...)))
                        ***...***TGGTAAAGAACACATTGAAAGTACTGCA***...***
                        ......TGGTAAAGAACACATTGAAAGTACTGCA......            6  P7-1 +/-
                        ......TGGTAAAGAACACATTGAAAGTACTGCA......            6  P7-1 +/-
```

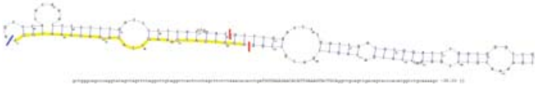

```
non-candidate   27  GCTGGCGTTGTGGTGGGCGGCAGGTGGGATCTTGAGTAAATGGTTGCGCTTCCCCTCTACAGGACACAGAATGTTCTTGGGGAGAAGGGTCGTCGGATCAGAGAGTTGACCGCAGTTGTCCAGAAGCGCTTTGGC
                        ((((((((....((((((((..((((..((((..((...(((..((.((((.((((...((((((.....)))))).)))).)))).))..))).))..))))......)))).))))))))..))))))..)))
                        ***...***TGTTCTTGGGGAGAAGGGTCGTCGGA***...***
                        ......TGTTCTTGGGGAGAAGGGTCGTCGGA......              11  P7-1 +/-
                        ......TGTTCTTGGGGAGAAGGGTCGTCGGAT......             8  P7-1 +/-
                        ......TGTTCTTGGGGAGAAGGGTCGTCGG......               6  P7-1 +/-
                                                                            25  P7-1 +/-
```

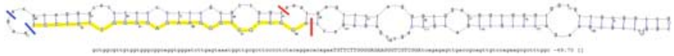

```
potential       28  AGTACAGTGGTAGGCAGATGACCACTGACTGTCATCTTTTCTGTGGTGGCACTGGGCATGCTGGGCCACTGGTGAAGCCTGCTCCAGCACGTGTCACACTGGATGAGTACAGTGGTAGGCAGATGACCACTGACT
                        (((.(((((((..((.....(((((((((..((((((.....(((.(((((((((((((.(((..(((...)))..))).))).)))....))))))))).)))))))).)))))))..)).....))))))))))
                        ***...***TGGATGAGTACAGTGGTAGGCAGATG***...***
                        ......TGGATGAGTACAGTGGTAGGCAGATGA......             5  P7-1 +/-
                        ......TGGATGAGTACAGTGGTAGGCAGATGACC......           5  P7-1 +/-
                                                                            10  P7-1 +/-
                        ......TGGATGAGTACAGTGGTAGGCAGATG......              5  P10-1 +/-
                                                                            5  P10-1 +/-
```

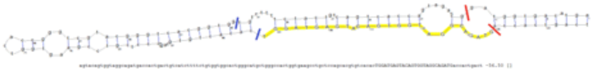

```
non-candidate   29  AGAAGACTCCTCACACACTTGCTTGGCAGGGGAGAGTCCATGATCAAGGAGCTGGTCTTTCCTGGGTAATGCTTATTCTTTGCAGTCTGGATGTGCTTGACTGAGTAATTTTTGGTAGTGGGGGAATGTATTCT
                        ((((.(((((((.((((((((((..(((((.(...(((((.((((((((((..(((.((.(....).)).)))..))))))))..))))))))).).)))).)..)))).....))).))))))))..)).))))
                        ***...***TTATTCTTTGCAGTCTGGATGTGCTT***...***
                        ......TTATTCTTTGCAGTCTGGATGTGCTT......              12  P7-1 +/-
                        ......TGCTTATTCTTTGCAGTCTGGATGTG......              5  P7-1 +/-
                                                                            17  P7-1 +/-
                        ......TTATTCTTTGCAGTCTGGATGTGCTT......              22  P10-1 +/-
                                                                            22  P10-1 +/-
                        ......TTATTCTTTGCAGTCTGGATGTGCTT......              8  P10-2 +/-
                                                                            8  P10-2 +/-
```

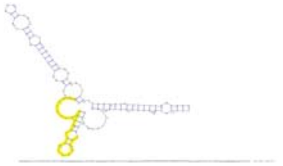

```
high confidence 30  GGCATTCTGGGTTTTGTTTGTTTGTTTGTTTGTTTGTTTGTTTGTTTTGAGACAGGGTTTCTCTGCATAGCCTTGGCTGTCCTGGAACTCACTCTGTAGACGAGGCTGACCTCGAACTCAGAAATCCACCTGCC
                        ((((...((((((((....(((((...(((.((...((((((((....(((...(((((((...(.(((((....))))).).))))))).)))..))))))))))).)))..)))))...))))))))..))))
                        ***...***TCACTCTGTAGACGAGGCTGAC***...***
                        ......TCACTCTGTAGACGAGGCTGAC......                  5  P7-1 +/-
                                                                            5  P7-1 +/-
                        ......TCACTCTGTAGACGAGGCTGAC......                  5  P10-2 +/-
                                                                            5  P10-2 +/-
```

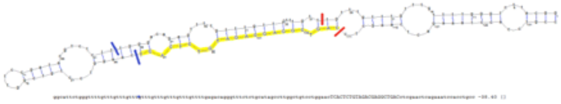

```
high confidence 31  AGTATTACTGTGGGGCCACCTTCTCTCAGCAGCTGGCATCTGTACTTGTGGTATATCTGGCACTGCCAAGTGGTGCAGGACCTCCCAGGGTGCAGTGGCAAGTGTGGCAGCTACTGGTCCTTTCCTCTGGTGCT
                        (((((((..(.(((((((....((.(((.((..((.((.((((((((.(((....(((.((((..(...)..)))).)))...))))))))))))).))..))))).))....)))))))...)..)))))))
                        ***...***TCTCTCAGCAGCTGGCATCTGTACTT***...***
                        ......TCTCTCAGCAGCTGGCATCTGTACTT......              9  P7-1 +/-
                        ......TCTCAGCAGCTGGCATCTGTACTTGT......              6  P7-1 +/-
                        ......TTCTCTCAGCAGCTGGCATCTGTACTT......             6  P7-1 +/-
                                                                            21  P7-1 +/-
                        ......TCTCTCAGCAGCTGGCATCTGTACTT......              16  P10-1 +/-
                        ......TCTCAGCAGCTGGCATCTGTACTTG......               8  P10-1 +/-
                                                                            24  P10-1 +/-
```

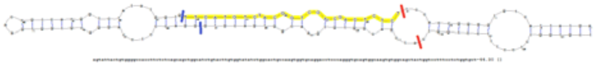

```
high confidence 32  TCTGCACTGGTAGTCTTGGGTTCTACAAGAGAGCAGTCTGAGCAAGCCAGGGGAAGCAAGCCAGTAAAGAAACATCCCTCCGTGGCCTCTGCATCAGTTCCTGCTTCTTGACCTGCTTGAGATCCAGTCCCGA
                        ((.(.(((((..((((..(((....((((.(((((.(((((((.((((((((...................)))))...))))...))).))))...)))))))))....))).)))))))))).).))
                        ***...***TGGGTTCTACAAGAGAGCAGTCTGAGCA***...***
                        ......TGGGTTCTACAAGAGAGCAGTCTGAGCA......            7  P10-1 +/-
                                                                            7  P10-1 +/-
                        ......TGGGTTCTACAAGAGAGCAGTCTGAGC......             5  P10-2 +/-
                                                                            5  P10-2 +/-
                        ......TGGGTTCTACAAGAGAGCAGTCTGAGCA......            5  P14-1 +/-
                                                                            5  P14-1 +/-
```

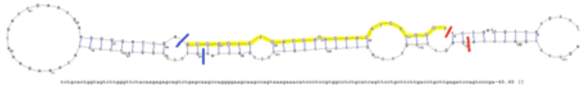

```
potential       33  AGTTCCACAGTGCCAGATAGCCAAGAATGATCTTTTAGCTGGAGTCTGTACCCTGAAGGGAGATTGGTCACAGTGCTCTGGGTCTGGAATGTTTCTCAGCCATTAGTTCATGCCAGAAGTCTCACTGGGAACT
                        (((((.(((((..((((..(.....((((.((..(.((((((((((..((((.....((((((((....)))).)))))))))..)))....))).)))).)..)).))))....)..))))))))))))))
                        ***...***TGATCTTTTAGCTGGAGTCTGTACCCTG***...***
                        ......TGATCTTTTAGCTGGAGTCTGTACCCTG......            7  P14-1 +/-
                        ......TGATCTTTTAGCTGGAGTCTGTACCCT......             5  P14-1 +/-
                        ......TGATCTTTTAGCTGGAGTCTGTACCCTGA......           5  P14-1 +/-
                                                                            17  P14-1 +/-
```

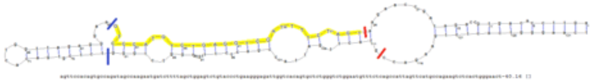

```
high confidence 34  GCTGCAGGCCAGTACAAACACAGGTGGAGCTGGAGCAGGGACAGAGGAGCTCAGGTAGAGGACGTGGCCATTCTTGTACTCAGCAGCTAGGCTCTTGGCCTCCCTGGGTGTAGAACATTGGTAGATAAAGC
                        (((....((((((....((((.((.((((((((((((..((...(..(((..((((.((.((......)).))))))..)))..)..))..)))).))).)))))))..)))).....))))))......)))
                        ***...***TACAAACACAGGTGGAGCTGGAGCAG***...***
                        ......TACAAACACAGGTGGAGCTGGAGCAG......              31  P7-1 +/-
                        ......TACAAACACAGGTGGAGCTGGAGCAGG......             11  P7-1 +/-
                        ......TACAAACACAGGTGGAGCTGGAGCA......               8  P7-1 +/-
                                                                            50  P7-1 +/-
                        ......TACAAACACAGGTGGAGCTGGAGCA......               17  P10-1 +/-
                        ......TACAAACACAGGTGGAGCTGGAGCAG......              11  P10-1 +/-
                                                                            28  P10-1 +/-
                        ......TACAAACACAGGTGGAGCTGGAGCA......               9  P10-2 +/-
                        ......TACAAACACAGGTGGAGCTGGAGCAG......              8  P10-2 +/-
                                                                            17  P10-2 +/-
```

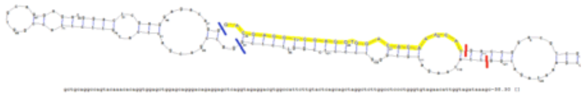

```
high confidence 35  CTTTCCTGTGAGATTATGGTACTGTGCAGCAAGCGGGCAGAAGACACTGGGAGGCTCGTAGGTGGTCACTGAATCCCAGACGTCAGCTTCACCCATTGCAGGGTTGCATGGAGAAATTAGTCTTGGGATGG
                        ((.((((..(((((((.....(((((((((..(((((.((..(((.((((((......(((.......)))..))))))..)))..))...)))...))...))))))))).....)))))))))))).))
                        ***...***TGAGATTATGGTACTGTGCAGCAAGC***...***
                        ......TGAGATTATGGTACTGTGCAGCAAGC......              10  P7-1 +/-
                                                                            10  P7-1 +/-
```

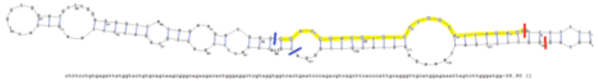

high confidence

```
36 GGAGCAGGTGGTGAAGCGGCAGGATCTCTTCATTGTCAGCAAGCTGTGGTGCACGTTCCATGACAAGAGCATGGTGAAAGGAGCCTTCCAGAAGACACTGAGCGACCTGCAGCTGGACTACCTGGATCTCT
   ((((((((((..(((.(((((.((.((.((.((((......(((.(((...(...(((((.(....))))))).....)..)))...))).)))).)))).))))))).))).)))))))...))))
   **************************************************************************************************TGAGCGACCTGCAGCTGGACTACCTGG******
   ..................................................................................................TGAGCGACCTGCAGCTGGACTACCTGG......
   ...................................................................................................................................
   ----------------------------------------------------------------------------------------------------
   ..................................................................................................TGAGCGACCTGCAGCTGGACTACCTG.......
   ...................................................................................................................................
   ----------------------------------------------------------------------------------------------------
   ..................................................................................................TGAGCGACCTGCAGCTGGACTACCTGG......
   ...................................................................................................................................
   ----------------------------------------------------------------------------------------------------
   ..................................................................................................TGAGCGACCTGCAGCTGGACTACCTG.......
   ...................................................................................................................................
   ----------------------------------------------------------------------------------------------------
```

7 P7-1 +/-
7 P7-1 +/-

5 P10-1 +/-
5 P10-1 +/-

6 P10-2 +/-
6 P10-2 +/-

5 P14-1 +/-
5 P14-1 +/-

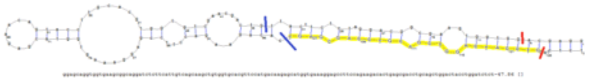

high confidence

```
37 CAAGCTGTAATAAGAACTTGCCCAATAATACTGGACATAGCGTGGAAACCAGAGCAGGGTGGCTAGAATTTAAACCCTCTCTGTGGTCTTTGTTTTCAGTCCACGTGTGTGGTTGAGTGTGTGAAGCTTG
   ((((((...(((...((((..(((...(((((((((..((((.(((...(((((.(((((.............)))))))))).)...))).)))).....))))).)))).))).)))).))).))))))
   ****************************TACTGGACATAGCGTGGAAACCAGAGC**************************************************************************
   ............................TACTGGACATAGCGTGGAAACCAGAGC..........................................................................
   .....................................................................................................................................
   ----------------------------------------------------------------------------------------------------
   ............................TACTGGACATAGCGTGGAAACCAGAGC..........................................................................
   .....................................................................................................................................
   ----------------------------------------------------------------------------------------------------
```

48 P7-1 +/-
48 P7-1 +/-

19 P10-1 +/-
19 P10-1 +/-

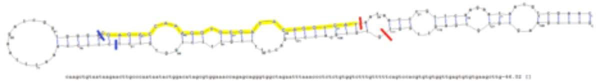

high confidence

```
38 GTATACGAACTGCTTGCTCTTTCTGGCTTTGCCTTCCCTCCATTCTCCTGGGGATGCTACTCCCTTTGTCTTGGGTAGTTCTGCGAGAGACTGCTGGGGACAGGCATGGGAGAAAGCTAGGGCCTTGTGC
   ...((((...((((...((((((..((.((((((((((.((.((((.((.(((.(((((((.........)))))))))).)).)))).))..))))).))))).)).))))))...)))).))))).
   ****************************************************************************TGCGAGAGACTGCTGGGGACAGGCAT**************************
   ............................................................................TGCGAGAGACTGCTGGGGACAGGCAT..........................
   ............................................................................TGCGAGAGACTGCTGGGGACAGGCATG.........................
   ...................................................................................................................................
   ----------------------------------------------------------------------------------------------------
   ............................................................................TGCGAGAGACTGCTGGGGACAGGCAT..........................
   ...................................................................................................................................
   ----------------------------------------------------------------------------------------------------
   ...TACGAACTGCTTGCTCTTTCTGGCTTT.....................................................................................................
   ............................................................................TGCGAGAGACTGCTGGGGACAGGCAT..........................
   ...................................................................................................................................
   ----------------------------------------------------------------------------------------------------
   ............................................................................TGCGAGAGACTGCTGGGGACAGGCAT..........................
   ...................................................................................................................................
   ----------------------------------------------------------------------------------------------------
```

18 P7-1 +/-
10 P7-1 +/-
28 P7-1 +/-

31 P10-1 +/-
31 P10-1 +/-

5 P10-2 +/-
40 P10-2 +/-
45 P10-2 +/-

7 P14-1 +/-
7 P14-1 +/-

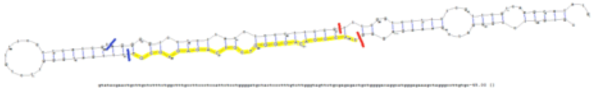

high confidence

```
39 GGCGCGCTTTGAGGCTGACCTGAAGGACAGGATGGATGGAAGTGACTTTGAGAGCACTGGAGCACGGAGCTTGAAAAGCAAGTTGGCTGCCATTCTGTTTACTAGTGATTGTAGCTTTGAGGTCTCGTC
   ((((.((((..((((((.((((..(((((((((((.(.((..((.((((..((((.(((.....))).)))).))))))..)).)...)))))))))))..))).)....)))))).))).).))))
   *******************TGAAGGACAGGATGGATGGAAGTGA**************************************************************************************
   ...................TGAAGGACAGGATGGATGGAAGTGAC.............................................................................
   ..................................................................................................................................
   ----------------------------------------------------------------------------------------------------
   ...................TGAAGGACAGGATGGATGGAAGTGA..............................................................................
   ..................................................................................................................................
   ----------------------------------------------------------------------------------------------------
```

7 P7-1 +/-
7 P7-1 +/-

8 P10-1 +/-
8 P10-1 +/-

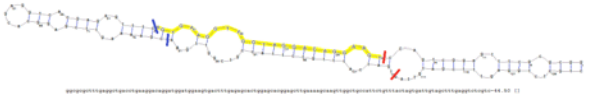

high confidence

```
40 GCCCTGGGGCTAAAGGAAGGAAGGAAAGGATCAATGGATGCCCTCAGCATGGGCTGTGTGTTCAGCATGGCTGGCTTCTCTCCTGGACTGTTCTTTCTTGGTCTCCTCTGCCTTTTATTTACCCTGGGC
   ((((.(((...(((((.((((((((((((((....(((.....(((((....((((......))))...)))))......)))......))))))))))....))))...)))))......))).))))
   ****TGGGGCTAAAGGAAGGAAGGAAAGGAT*********************************************************************************************
   ....TGGGGCTAAAGGAAGGAAGGAAAGGAT.............................................................................................
   ....TGGGGCTAAAGGAAGGAAGGAAAGGA..............................................................................................
   ....TGGGGCTAAAGGAAGGAAGGAAAGGATC............................................................................................
   ...................................................................................................................................
   ----------------------------------------------------------------------------------------------------
```

11 P7-1 +/-
6 P7-1 +/-
5 P7-1 +/-
22 P7-1 +/-

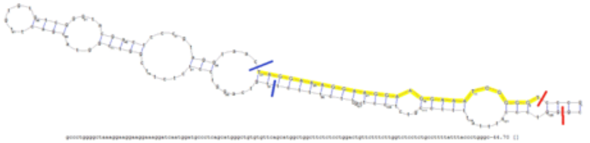

high confidence

```
41 GGGCTGTTTTATGCCATGAATTGTTTTGTGTGTGTGGTTTCAGGTATCTGGGACCAGTTGCTCATCAGCTCTAAGCCTCATCCCAGAAAGACCTCTACACTTCAAACAGTTCGGATGCAGAGGAGCCC
   (((((.(((..(((..((((((((((.(...((((((....((((.(((((((..((..(((...........))))).)))))))...)))))))))))..)))))))))))...)))))).)))))
   ***************************************************************************************************TACACTTCAAACAGTTCGGATGCAGAGG*****
   ...................................................................................................TACACTTCAAACAGTTCGGATGCAGAGG.....
   ........................................................................................................TTCAAACAGTTCGGATGCAGAGGAGC...
   .....................................................................................................................................
   ----------------------------------------------------------------------------------------------------
```

6 P7-1 +/-
5 P7-1 +/-
11 P7-1 +/-

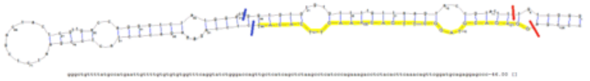

potential

```
42 TCTGGGCTGGTAGTCTTGAGTTTTATAAGAGAGCAAGCTGAGCAAGACAGGAGAGGCAAGCCAGTAACATCCCTCCATGACCTCTGCATCAGCTCCTGCTCCCTGACCTGCTTGAGTTCCAGTTCTGA
   ((.(((((((....((..(((......((.(((((((((((((((((.((((((.((..............)))))).))..)).))).)))))).))))).))......)))..))..))))))).))
   **************************TAAGAGAGCAAGCTGAGCAAGACAGGA*******************************************************************************
   ..........................TAAGAGAGCAAGCTGAGCAAGACAGGA.......................................................................
   ..................................................................................................................................
   ----------------------------------------------------------------------------------------------------
```

5 P10-2 +/-
5 P10-2 +/-

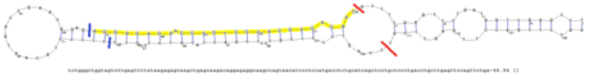

high confidence

```
43 ATGGTTCCAGATCTGTGGGAGTCCCTTTTCTGGGTAAGCAGCGTGGCTGGCCCCGGGGCCTACATACCTCGATGCCAGAGCACATGGGAACTGCCTCCTTTGAAAATTCACTTGCAGACTGGACTGT
   (((((.((((.((((..((.(....(((((.(((...((((((((.(((((..(((((........)))))..)))))....))))....)))).))).)))))..).))..)))))))))))))))
   ****************************************************************************************************TGAAAATTCACTTGCAGACTGGACTGT*
   ....................................................................................................TGAAAATTCACTTGCAGACTGGACTGT.
   .................................................................................................................................
   ----------------------------------------------------------------------------------------------------
   ....................................................................................................TGAAAATTCACTTGCAGACTGGACTGT.
   .................................................................................................................................
   ----------------------------------------------------------------------------------------------------
```

5 P7-1 +/-
5 P7-1 +/-

5 P10-2 +/-
5 P10-2 +/-

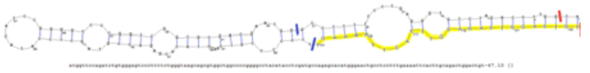

high confidence

```
44 CTTGGTCTAGATAGTTTTAGGACAGCCAGCAGTATGTAGAGAGACCAACCATAAAAGAAAGAAATTGGAAGGTAGTTTTAAAAAGTGTGCCAAATGGTAAGTTAGGAACATCTTATTTAGAAACAGG
   ((((.((((((((((((((..(((.(((((((.(((.(((((((..(((..(((..............)))..))).))))))....))))))....)))).))).)))))....)))))))))..))))
   ****************TAGGACAGCCAGCAGTATGTAGAGAGACC*************************************************************************************
   ................TAGGACAGCCAGCAGTATGTAGAGAGACC.....................................................................................
   ..................................................................................................................................
   ----------------------------------------------------------------------------------------------------
```

6 P7-1 +/-
6 P7-1 +/-

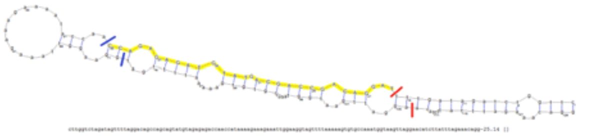

potential

```
45 GACCCAGTGTTCTCTGAAGAGATGTCTGCTGCTCAGGGCATGCTGTGTGTGGGGCAGGTGCTGGGCATCTGAGTAACAGGCAGAAGCCATTAGGAAGGAGCAGCTCCCTTTTATGAGCCAGGGTC
   (((((.(.((((...((((.((.(.(((((.(((..(((...((((.(((....(((((((...)))))))....))).)))).)))).....).)).))))))))).)))).))))).)))))
   ************TCTGAAGAGATGTCTGCTGCTCAGGGC**************************************************************************************
   ............TCTGAAGAGATGTCTGCTGCTCAGGGC......................................................................................
   .............................................................................................................................
   ------------------------------------------------------------------------------------------
```

6 P10-1 +/-
6 P10-1 +/-

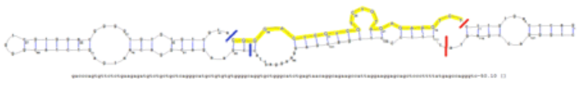

potential

```
46 TCTTCATTTCCCCATGTTCATTCTTGCAACCCGGACATTTCCTGAGTCAGACCCTGTTGCCTTTCCTCACTGGGAACCTGTCAGATGCTAGTTTGCCTTTCAGATGGATGTGGAGGGAGGGAAGA
   (((((.((((((((((((((((...((((.(..(.(((...((((..(((.(((.((...........)).)))...)))))))))))..).))))......)))))))))))).))))).)))))
   **************************************************************************************************TTCAGATGGATGTGGAGGGAGGGAAG**
   ..................................................................................................TTCAGATGGATGTGGAGGGAGGGAAG..
   ..................................................................................................TTCAGATGGATGTGGAGGGAGGGAA...
   ..............................................................................................................................
   ------------------------------------------------------------------------------------------
```

6 P7-1 +/-
5 P7-1 +/-
11 P7-1 +/-

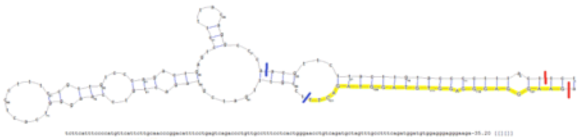

non-candidate

```
47 GGCAGGTGGTGTCACCACAAACCTGGTGACATCACTGAAGATTCTTGGGCTCTCCTGGATACAAGACATTGTCCAGGGAAGGGACTACTAATGATGTTACTGGGAACTGCAATGTCCTACCTGCT
   (((((((((.(.((...((..((..(((((((((..(.((.(((((....((.((((((((........))))))))))))))))).)...)))))))))..))...))...)).)))))))))
   **************************TGACATCACTGAAGATTCTTGGGCTCT************************************************************************
   ..........................TGACATCACTGAAGATTCTTGGGCTCT........................................................................
   ..........................TGACATCACTGAAGATTCTTGGGC...........................................................................
   ..........................TGACATCACTGAAGATTCTTGGGCTC.........................................................................
   .............................................................................................................................
   ------------------------------------------------------------------------------------------
```

16 P14-1 +/-
9 P14-1 +/-
9 P14-1 +/-
34 P14-1 +/-

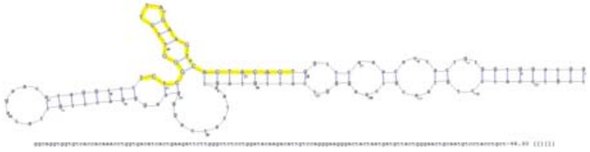

non-candidate

```
48 GAGGCCCTGGCCCTAGCAGGGAGGTGACAGGTCTACAAAGCTCTGTGCATGGCTCTTCTGGAGGAAGCCCCAGCCATGTGGTTTCCAGTGCTTGGCAATACATCTGGGTTTAGGGCCATTGCCTC
   (((((..((((((((((..(((.(((....(((....((((.((((((((((((((((.....))))....)))))))))).....))).)))))))..))).))).)..).))))))))..)))))
   *********************************TACAAAGCTCTGTGCATGGCTCTTCTG*****************************************************************
   .................................TACAAAGCTCTGTGCATGGCTCTTCTG.................................................................
   .................................TACAAAGCTCTGTGCATGGCTCTTCT..................................................................
   .............................................................................................................................
   ------------------------------------------------------------------------------------------
   .................................TACAAAGCTCTGTGCATGGCTCTTCT..................................................................
   .............................................................................................................................
   ------------------------------------------------------------------------------------------
```

8 P7-1 +/-
5 P7-1 +/-
13 P7-1 +/-

5 P10-2 +/-
5 P10-2 +/-

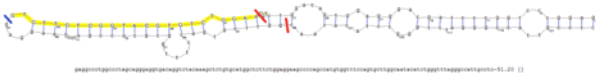

non-candidate

```
49 ACTTGCGTGGATGCACCTCTGCATGTGGAGGCTGGAGATGTGTCAAGTCAGCCCTGAAGGCAGGGCCACCTTAGTCAACATAGCAGGGTCTCTGTCACATCCAGAGCTCACCAATACAGCTAGT
   (((.((.(((.((...(((((.(((((((((((.(..(((((.((((...((((((....))))))...))).).).))))..)..)))))))...))))).)))))..)))))......)).)))
   *******************TGCATGTGGAGGCTGGAGATGTGTCA*******************************************************************************
   ...................TGCATGTGGAGGCTGGAGATGTGTCA...............................................................................
   ............................................................................................................................
   ------------------------------------------------------------------------------------------
   ...................TGCATGTGGAGGCTGGAGATGTGTC................................................................................
   ............................................................................................................................
   ------------------------------------------------------------------------------------------
```

6 P7-1 +/-
6 P7-1 +/-

5 P10-1 +/-
5 P10-1 +/-

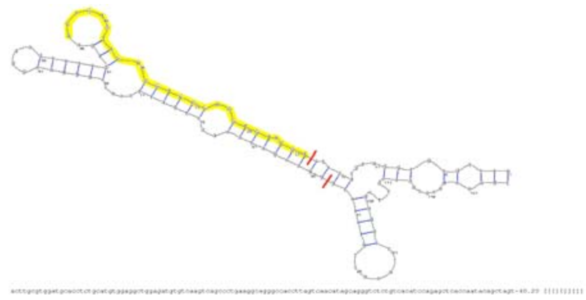

potential

```
50 TCTGTCTGTTTGATGATTTTCATCTGCATGTGTTCTTGCCTGCTCCCAAACTCGCCTTTGATCTTCAGGAAGTAAATTGTTAGTAAGCATTATATGTGCATAGTGGAAAGTCAAGCTTTAAGA
   (((....(((((((..(((((((.((((((((((...(((.((((..((((((..(((..........))).)))..)))..)))).)))..)))))))))..))))))))))))))))....)))
   ******************************************************************************************TATGTGCATAGTGGAAAGTCAAGCTT******
   ..........................................................................................TATGTGCATAGTGGAAAGTCAAGCTT......
   ...........................................................................................................................
   ------------------------------------------------------------------------------------------
```

8 P7-1 +/-
8 P7-1 +/-

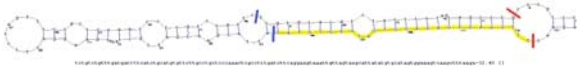

high confidence

```
51 AGAAGTGAATGCTGAGAGAGCTGCTTCAGACAGTGAAGGACAGACGGTGCCTCTTTTGTATGCTGTGTATCCTTGGCCTGCCTGCCTGCCTGCCTGGGTGGCTCCGCTGGATGCTTCT
   ((((((..((.(...(.((((..((((((.(((.(.(((.(((.(((.(((......((((.....))))....)))))).)))))).)))).)))...)))..)))))...).))))))))
   ************************TTCAGACAGTGAAGGACAGACGGTGC********************************************************************
   ........................TTCAGACAGTGAAGGACAGACGGTGC....................................................................
   ..............................................................................................................................
   ------------------------------------------------------------------------------------------
   ........................TTCAGACAGTGAAGGACAGACGGTG.....................................................................
   ..............................................................................................................................
   ------------------------------------------------------------------------------------------
```

6 P7-1 +/-
6 P7-1 +/-

5 P10-1 +/-
5 P10-1 +/-

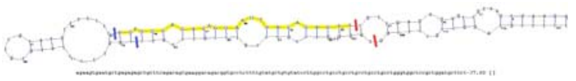

non-candidate

```
52 CGAGCTGAGTCTGACTTTCACAGCCCATGCTGTCATCTGTGGCACAAGTGGGTGTGAGTACGTCCTGCATGCACTAACGGTGCAGAACATGGCATGGTGACTAGAAAGTAAAACTCCTTTTG
   ((((..((((...((((((....((((((((((..((((((.(...((((.((((..(.....)..)))).))))...).))))))..))))))))))).)....))))))...))))..))))
   ***************************TGCTGTCATCTGTGGCACAAGTGGGT*********************************************************************
   ...........................TGCTGTCATCTGTGGCACAAGTGGGT.....................................................................   5 P7-1 +/-
   ..........................................................................................................................   5 P7-1 +/-
   -----------------------------------------------------------------------------------------------------------
```

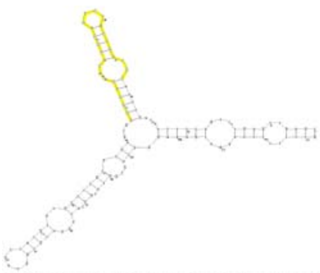

potential

```
53 CTCGAGGCATGTTTGTTTGTTGTACAGGTCCTGGGCATCTCTCGTTCCAGTCAAGTTGACAAAAGCAAGACCAGCCAGCATGGGAAAACTAGGCCTGAGCTCAGAAAAGGGTGCAGACGAG
   ((((..((((.(((.((((..((.(((((.(((((...((((..(((...(((..(((......)))..)))....)))..))))...)))))))))).)).)))).))).))))...))))
   *********TGTTTGTTTGTTGTACAGGTCCTGGGC***************************************************************************
   .........TGTTTGTTTGTTGTACAGGTCCTGGGC...........................................................................   247 P14-1 +/-
   .........TGTTTGTTTGTTGTACAGGTCCTGGG............................................................................    45 P14-1 +/-
   .........TGTTTGTTTGTTGTACAGGTCCTGG.............................................................................    38 P14-1 +/-
   .........TGTTTGTTTGTTGTACAGGTCCTGGGCA..........................................................................    14 P14-1 +/-
   .............TGTTTGTTGTACAGGTCCTGGGCATC........................................................................    13 P14-1 +/-
   .........TGTTTGTTTGTTGTACAGGTCCTGGGCATC........................................................................    12 P14-1 +/-
   .............TGTTTGTTGTACAGGTCCTGGGCATCT.......................................................................    10 P14-1 +/-
   .............TGTTTGTTGTACAGGTCCTGGGCAT.........................................................................     9 P14-1 +/-
   ............TTGTTTGTTGTACAGGTCCTGGGCA..........................................................................     6 P14-1 +/-
   ...............................................................................................................   394 P14-1 +/-
   ---------------------------------------------------------------------------------------------------
```

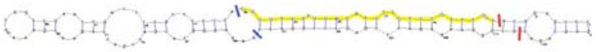

high confidence

```
54 GGTGTCTGTGTCCCTGTGAATGGAAAACAAGTTAAACCTATTTATTAGTATTTCATCTAACCCTAAAGTTTGAGAGTTCTTTAAAGGTTTGACGTTTGGTGGGCAGGCTCACAAGGCCCC
   ((.(((((((..(((((..((.(((.....(((((((((..(((..((..(((((.((........))..)))))...)).))))))))))))).))).))..)))))..))).)))))).
   ********************************************************************************TTAAAGGTTTGACGTTTGGTGGGCAG**************
   ................................................................................TTAAAGGTTTGACGTTTGGTGGGCAG..............   6 P7-1 +/-
   ........................................................................................................................   6 P7-1 +/-
   ---------------------------------------------------------------------------------------------------------
```

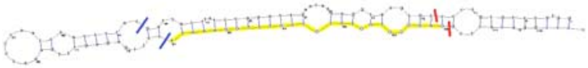

potential

```
55 GAGGGCATTGGATCCCCTGGAACTAGAATTACAGGTTGTGAGCTGCCATGCAGGTACTGGGAATTGAACCTGGATCCTCAAGAAGAGCAGCCTGTGCTCTTAACCACTGAACCATTTCTC
   (((((...(((.((...((((....(((..((((((((((...((..(.((.(((..(..((.......))..)..))))).).)).))))))))))).)))...)))..)).))).)))))
   *****************TGGAACTAGAATTACAGGTTGTGAGCTGC**************************************************************************
   .................TGGAACTAGAATTACAGGTTGTGAGCTGC..........................................................................   5 P7-1 +/-
   ........................................................................................................................   5 P7-1 +/-
   ---------------------------------------------------------------------------------------------------------
```

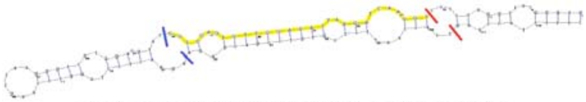

high confidence

```
56 TGACACTATGGGAGAAATCTTTTAGGAACTATTGGGACAGATTCAGGCTTTTATGTACTTCTTACTACAGTTGTAAAATGAGATCTCGTCTCGGTGCCTGGATGATTCTTCTCATGTTA
   ......(((((((..((((.((((((...(((((((((((((((.((.....((((.........))))...))....))).)))).))))))))))))))).)))).))))))))...
   ***********************TAGGAACTATTGGGACAGATTCAGGCT*********************************************************************
   .......................TAGGAACTATTGGGACAGATTCAGGCT.....................................................................   6 P7-1 +/-
   .......................................................................................................................   6 P7-1 +/-
   ---------------------------------------------------------------------------------------------------------
   .......................TAGGAACTATTGGGACAGATTCAGGCT.....................................................................   5 P10-1 +/-
   .......................................................................................................................   5 P10-1 +/-
   ---------------------------------------------------------------------------------------------------------
```

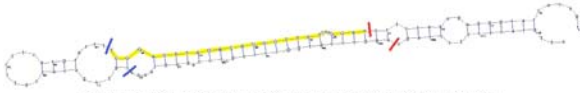

high confidence

```
57 TTGCAATAGAGGCCTCAGAACGAGTGTTTTCTTTAGAGCCTGCCCTGAGGGTGAGGAAGGCCGGCCTATTCAGCATGTGGGTTTTCTGAGAGGAGAGCTGGTCTGAGCAGTTACTGTGA
   (..((.(((..((.(((((.(.(((.(((((((.((((((..(.((((((((..((....)).)))).))))...)..)))))))..)))))))..))).))))))))..))).))..)
   ************************************************************************************TCTGAGAGGAGAGCTGGTCTGAGCAG*********
   ....................................................................................TCTGAGAGGAGAGCTGGTCTGAGCAG.........   6 P7-1 +/-
   .......................................................................................................................   6 P7-1 +/-
   ---------------------------------------------------------------------------------------------------------
```

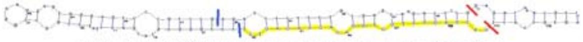

non-candidate

```
58 CGGATTTTGAGGTCATGGAACAGCTGGATGCAGATGGTGGCCCTCTCCGCACGCGCTCTGGAGAAGCAGCGGCCCGAGACATAGTGCAGTTCGTGCCTTATCGACGATTTCAGAATGTG
   ((.(((((((((((.(.((...((((((((((.(((..((((((((((((....))...)))))....).))))).....)))..)))).)))).))....)).).)))))))))).))
   *******TGAGGTCATGGAACAGCTGGATGCAGA*************************************************************************************
   .......TGAGGTCATGGAACAGCTGGATGCAGA.....................................................................................   5 P10-1 +/-
   .......................................................................................................................   5 P10-1 +/-
   ---------------------------------------------------------------------------------------------------------
```

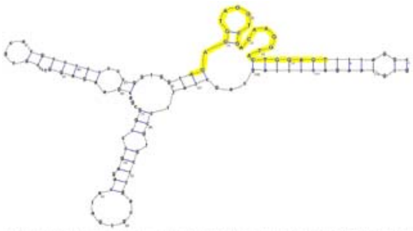

non-candidate

59
```
GCCAGCTAGCATGACATACACAGCAGTGAAAAACAGAGACGCTACCTCAGACAAGCTTGGAAGGAAGGAAGGAAGGACTGGGGTTGTCCTCTGACCTCTCTGTATGCTGCTGCACGGGC
(((.((.((((.(.((((((.((.((.(.....(((((((((..(((((((.(...(((...........)))...).))))))))))).))))).))))).)))))))))))))...)))
*****************************************************************************************TCTGACCTCTCTGTATGCTGCTGCACGG***
.........................................................................................TCTGACCTCTCTGTATGCTGCTGCACGG...
.......................................................................................................................
-------------------------------------------------------------------------------------------------------
.........................................................................................TCTGACCTCTCTGTATGCTGCTGCACGG...
...........................................................................................TGACCTCTCTGTATGCTGCTGCACGG...
.......................................................................................................................
-------------------------------------------------------------------------------------------------------
```

7 P7-1 +/-
7 P7-1 +/-

14 P10-1 +/-
8 P10-1 +/-
22 P10-1 +/-

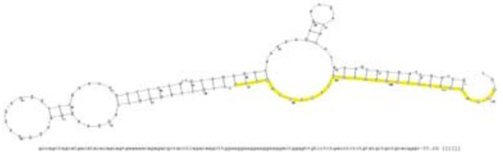

high confidence

60
```
GTTCTATGTTTTGTTTTGTTTTTTCATAGCTGAGAGATTGCTTCTGTATGTATACAATAAGGTAACATTGAAACAGAACATAAACTTTGAGACTTAAGGAAAAGCAGCTGACTTAGAAC
(((((.(((..((..((((((((..((((.((((..((.((((((((((.(((......)))))))....)))))))....)))).)).))..))))))))))).))).))))))
*************************************************************************************TGAGACTTAAGGAAAAGCAGCTGACTTA*****
.....................................................................................TGAGACTTAAGGAAAAGCAGCTGACTTA.....
.....................................................................................TGAGACTTAAGGAAAAGCAGCTGACTT......
.....................................................................................................................
-------------------------------------------------------------------------------------------------------
.....................................................................................TGAGACTTAAGGAAAAGCAGCTGACTTA.....
.....................................................................................TGAGACTTAAGGAAAAGCAGCTGACTT......
.....................................................................................................................
-------------------------------------------------------------------------------------------------------
.....................................................................................TGAGACTTAAGGAAAAGCAGCTGACTTA.....
.....................................................................................TGAGACTTAAGGAAAAGCAGCTGACTT......
.....................................................................................................................
-------------------------------------------------------------------------------------------------------
```

18 P7-1 +/-
5 P7-1 +/-
23 P7-1 +/-

12 P10-1 +/-
6 P10-1 +/-
18 P10-1 +/-

13 P10-2 +/-
6 P10-2 +/-
19 P10-2 +/-

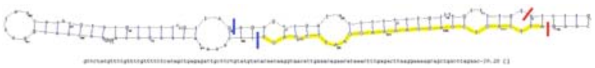

potential

61
```
AGGCTTGTCCTGCAGATGACCCTCTCTAGGCACGAGGAGTCTCCAGGTTTCCAGCAAGGGCAGAAACCGTGTGGTAGGGCCTCCTTGTGTACACAGAGAAAGATATCCAGGCCAAGCCT
((((((.((((..((((.(..(((((..(((((((((((((((((((((..((....)).))))))...)))..))).))))))))))....)))))..).)))))))).)))))))
*********************TCTCTAGGCACGAGGAGTCTCCAGGTT**********************************************************************
.....................TCTCTAGGCACGAGGAGTCTCCAGGTT......................................................................
.....................................................................................................................
-------------------------------------------------------------------------------------------------------
```

8 P14-1 +/-
8 P14-1 +/-

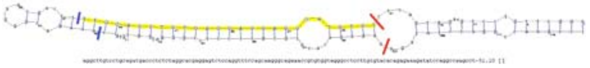

high confidence

62
```
GGCAGTGTTCTAGAGATAGCCAAGGTTGTACCTTTTACTGTGGCATGACTTGGTAATGTGTAAGAGTTACCCAGGTGGTACTGGTTTTGAAGGTATGACGGAATCATGCAGAACAGCC
(((..((((((.(.(((..((...((..(((((((.((((((.(((..((.((((((........)))))).))))).))).)))...)))))))..)))).))).)..)))))))))
*****************************************************************************TACTGGTTTTGAAGGTATGACGGAAT**************
.............................................................................TACTGGTTTTGAAGGTATGACGGAAT..............
.............................................................................TACTGGTTTTGAAGGTATGACGGAATC.............
................................................................................TGGTTTTGAAGGTATGACGGAATCATG..........
.....................................................................................................................
-------------------------------------------------------------------------------------------------------
.............................................................................TACTGGTTTTGAAGGTATGACGGAAT..............
.....................................................................................................................
-------------------------------------------------------------------------------------------------------
.............................................................................TACTGGTTTTGAAGGTATGACGGAAT..............
................................................................................TGGTTTTGAAGGTATGACGGAATCAT...........
.....................................................................................................................
-------------------------------------------------------------------------------------------------------
```

10 P7-1 +/-
5 P7-1 +/-
5 P7-1 +/-
20 P7-1 +/-

6 P10-1 +/-
6 P10-1 +/-

5 P10-2 +/-
5 P10-2 +/-
10 P10-2 +/-

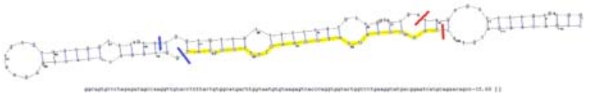

non-candidate

63
```
GAGCTTTGCATTTAACACACCAGTGCTGTAAACCCTCAGGACGGTAGCAGAGTCAACACAGCCTTTGCATTGTGGCAAATTGGGGTTTTGCTTCCTTTGTGGTTAGAGACAGATGTTT
((((((((..((((((.(((.((.(..(((((.((((((.(((((.((((((.(......).)))))))))))).....)))))).))))).))))..)))..)))))))))..))))).))))
**********************TGCTGTAAACCCTCAGGACGGTAGCAG*******************************************************************
......................TGCTGTAAACCCTCAGGACGGTAGCAG...................................................................
.....................................................................................................................
-------------------------------------------------------------------------------------------------------
......................TGCTGTAAACCCTCAGGACGGTAGCAG...................................................................
.....................................................................................................................
-------------------------------------------------------------------------------------------------------
```

6 P7-1 +/-
6 P7-1 +/-

5 P10-2 +/-
5 P10-2 +/-

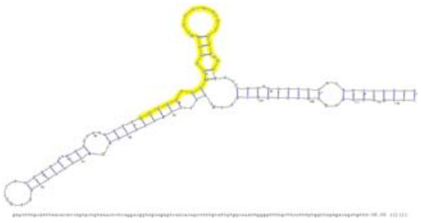

potential

64
```
GGACTGTGGAGAAAAGATTTGACTGATAGATGTTGGGACTTAGGCAAAGCTTTTGAAAGGCCTCTAACCTAAACTCCCACCCAGCCTGTCAGGCCCGCTTTGATATTCATTCAGTCT
(((((((((..((((......(((((((.((.(((((.(((((....((((.....))))......))))).))))).)).)))))))....)))).....)))))..))))))
*****************TGACTGATAGATGTTGGGACTTAGGCA***********************************************************************
.................TGACTGATAGATGTTGGGACTTAGGCA.......................................................................
.....................TGATAGATGTTGGGACTTAGGCAAAGC...................................................................
.....................TGATAGATGTTGGGACTTAGGCAAAG....................................................................
...................................................................................................................
-------------------------------------------------------------------------------------------------------
.....................TGATAGATGTTGGGACTTAGGCAAAGC...................................................................
.................TGACTGATAGATGTTGGGACTTAGGCA.......................................................................
.....................TGATAGATGTTGGGACTTAGGCAAAG....................................................................
...................................................................................................................
-------------------------------------------------------------------------------------------------------
```

9 P7-1 +/-
9 P7-1 +/-
5 P7-1 +/-
23 P7-1 +/-

8 P10-1 +/-
7 P10-1 +/-
5 P10-1 +/-
20 P10-1 +/-

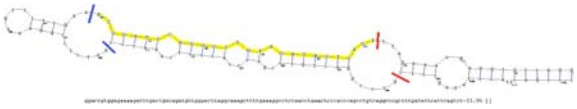

high confidence

65
```
GCCTGTGCAGTCCTGGTTTGATGACATGGCAGATACAGAGTTGCTGAACCTGATTCCAGTCTTCGAGGAGCTAAGTGGAACTGATGATGTCTACACCAGCCTTGGGCAGCTGCGGGC
(((((((.((((.((((((..(((((.((((.(((..((((.(((((.(((....)))..))).)).)))).)))...))).).)))))..))..)))).)))).)).))))))
************TGGTTTGATGACATGGCAGATACAGA*****************************************************************************
............TGGTTTGATGACATGGCAGATACAGA.............................................................................
............TGGTTTGATGACATGGCAGATACAGAG............................................................................
.................TGATGACATGGCAGATACAGAGTTGC........................................................................
...................................................................................................................
-------------------------------------------------------------------------------------------------------
```

8 P7-1 +/-
8 P7-1 +/-
5 P7-1 +/-
21 P7-1 +/-

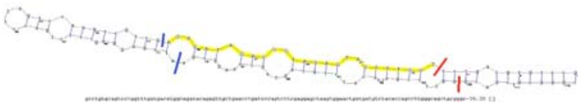

potential

66
```
AGAATGAGATGGGAGGTTTCTGGGAAAGGCATCTGATTCTCATCTCCTGCTCCAACCGGCGAGGGAGGGGCGGGGAGAGAGGGTGGTGAGTGACAGAGGAACTGTTCGCTCATTCT
((((((((.((((.((((((((.(..(...((((...(((((.((.((((((((..((.....))..))))))))))))))...))))..)..)..))))))).))))))))))))
****TGAGATGGGAGGTTTCTGGGAAAGGCA**********************************************************************************
....TGAGATGGGAGGTTTCTGGGAAAGGCA..................................................................................
.................................................................................................................
-------------------------------------------------------------------------------------------------------
....TGAGATGGGAGGTTTCTGGGAAAGGCA..................................................................................
.................................................................................................................
-------------------------------------------------------------------------------------------------------
```

9 P7-1 +/-
9 P7-1 +/-

8 P10-2 +/-
8 P10-2 +/-

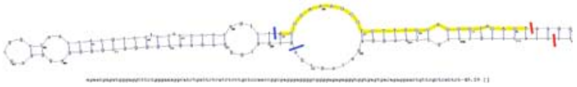

```
high confidence   67  GTATCTATTAACTAGAGAACTGGTCAGGTCTGAGGTGGGATGCTGAGGACTGTGATGTATCACTGGACAGTCATTTCATGTCTCTGAGCATGCTTCTTCATAGTAAAGATAGAGAC
                      ...((((((.((((((((((((.(((((...((.(((..(((((....(.((((....)))).)..))).)))..))).)))))))).)).).))))))..))))...))))))...
                      *******TAACTAGAGAACTGGTCAGGTCTGAGGT*************************************************************************************
                      .......TAACTAGAGAACTGGTCAGGTCTGAGGT.....................................................................................   13  P14-1 +/-
                      .......TAACTAGAGAACTGGTCAGGTCTGAGG......................................................................................    8  P14-1 +/-
                      .......TAACTAGAGAACTGGTCAGGTCTGAGGTG....................................................................................    5  P14-1 +/-
                      ........................................................................................................................   26  P14-1 +/-
                      ------------------------------------------------------------------------------------------------------
```

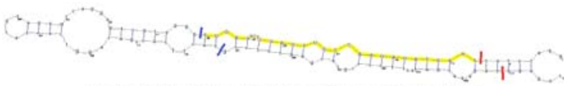

```
high confidence   68  GATTCCAAAGTCAGAAATGACAAATGACAAGTTTTCTGGTGTCCTTGCGGTGGTAGCCCTTTAAGGATGGAATTAGAAATAATGTGATGGGTCTGACCTGTAACTTTATGGAGTC
                      ((((((((((((((....(((..((.(((...(((((((((((((((.(((....)))...)))))))...)))))))))...))).))..)))....)))..)))).)))))))
                      ***************************************************************TAAGGATGGAATTAGAAATAATGTGATGG**************************
                      ...............................................................TAAGGATGGAATTAGAAATAATGTGATGG..........................    8  P14-1 +/-
                      .....................................................................................................................    8  P14-1 +/-
                      ------------------------------------------------------------------------------------------------------
```

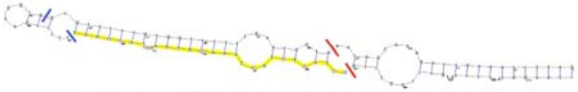

```
high confidence   69  GGGGAATTGAATATGGTCACTGCCTTAGGTACACTGGAGGGACAGGACCAAAAGTCAAGGGTCCCAGGCTCCCTTTAATACAAGGTGAACAGTAGACCATTAGTAGAGTGTCCCT
                      (((((.((....((((((((((((((...(((...(((((((((.(((((..........)))))...).))))))))).))).)))....)))).))))))).....))...)))))
                      *****************************************************************************TAATACAAGGTGAACAGTAGACCATT**************
                      .............................................................................TAATACAAGGTGAACAGTAGACCATT..............   34  P7-1 +/-
                      .............................................................................TAATACAAGGTGAACAGTAGACCATTAG............   20  P7-1 +/-
                      ................................................................................TACAAGGTGAACAGTAGACCATTAGTAG.........   17  P7-1 +/-
                      ................................................................................TACAAGGTGAACAGTAGACCATTAGTAGA........   11  P7-1 +/-
                      .............................................................................TAATACAAGGTGAACAGTAGACCATTA.............   10  P7-1 +/-
                      .............................................................................TAATACAAGGTGAACAGTAGACCAT...............    9  P7-1 +/-
                      ................................................................................TACAAGGTGAACAGTAGACCATTAGTA..........    5  P7-1 +/-
                      ...........................................................................TTTAATACAAGGTGAACAGTAGACCAT...............    5  P7-1 +/-
                      .....................................................................................................................  111  P7-1 +/-
                      ------------------------------------------------------------------------------------------------------
                      .............................................................................TAATACAAGGTGAACAGTAGACCATT..............   31  P10-1 +/-
                      .............................................................................TAATACAAGGTGAACAGTAGACCATTA.............   19  P10-1 +/-
                      .............................................................................TAATACAAGGTGAACAGTAGACCAT...............   16  P10-1 +/-
                      .............................................................................TAATACAAGGTGAACAGTAGACCATTAG............   15  P10-1 +/-
                      ................................................................................TACAAGGTGAACAGTAGACCATTAGTA..........   12  P10-1 +/-
                      .....................................................................................................................   93  P10-1 +/-
                      ------------------------------------------------------------------------------------------------------
                      .............................................................................TAATACAAGGTGAACAGTAGACCATTAG............    9  P10-2 +/-
                      .............................................................................TAATACAAGGTGAACAGTAGACCATT..............    8  P10-2 +/-
                      ................................................................................TACAAGGTGAACAGTAGACCATTAGTA..........    8  P10-2 +/-
                      .............................................................................TAATACAAGGTGAACAGTAGACCAT...............    7  P10-2 +/-
                      .............................................................................TAATACAAGGTGAACAGTAGACCATTA.............    7  P10-2 +/-
                      .....................................................................................................................   39  P10-2 +/-
                      ------------------------------------------------------------------------------------------------------
                      .............................................................................TAATACAAGGTGAACAGTAGACCATT..............    9  P14-1 +/-
                      ................................................................................TACAAGGTGAACAGTAGACCATTAGTA..........    8  P14-1 +/-
                      .............................................................................TAATACAAGGTGAACAGTAGACCATTAG............    7  P14-1 +/-
                      .....................................................................................................................   24  P14-1 +/-
                      ------------------------------------------------------------------------------------------------------
```

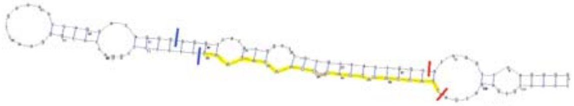

```
high confidence   70  AACTTAAGCTCTTTCCCGGTTTTGGGGGTTTTGTTTTTTTAAATCGCTTAGGGGTATTCTCTGGTGTTTTAGGAGCTGGTGACCTTTAAGCGCTGGATTGGAGGAGGAGGAGTT
                      (((((...(((((((((((((((((((((((.....((((((((.((((.((((.....)))))))).))))))))).....)))))))))).)))))...)))))))...)))))
                      *********************************************************************************************TAAGCGCTGGATTGGAGGAGGAGGA****
                      .............................................................................................TAAGCGCTGGATTGGAGGAGGAGGA....   15  P7-1 +/-
                      ............................................................................................TTAAGCGCTGGATTGGAGGAGGAGGA....   12  P7-1 +/-
                      ............................................................................................TTAAGCGCTGGATTGGAGGAGGAGG.....   11  P7-1 +/-
                      .............................................................................................TAAGCGCTGGATTGGAGGAGGAGGAG...    8  P7-1 +/-
                      ..........................................................................................................................   46  P7-1 +/-
                      ------------------------------------------------------------------------------------------------------
```

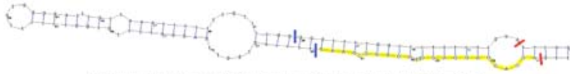

```
potential         71  GAGGTGCTGTGTTACGTGTGCTGGAGGATGAAGTAAGGAGTGATCCATCGGCTAAGTGTTTTCCCACTGTGCTGACACTCACATTGCTGACAGTGCACGTTTTCACAGCACCTC
                      ((((((((((..(((((..(((..((....(((...(((((.....(((((..((((......))))..))))))))))..))).))..)))..)))))...))))))))))
                      ******************GCTGGAGGATGAAGTAAGGAGTGA*******************************************************************************
                      ..................GCTGGAGGATGAAGTAAGGAGTGA..............................................................................   21  P7-3 -/-
                      ..................GCTGGAGGATGAAGTAAGGAG.................................................................................    8  P7-3 -/-
                      ..................GCTGGAGGATGAAGTAAGGAGT................................................................................    5  P7-3 -/-
                      .............................................................CCACTGTGCTGACACTCACATTGCTGACA.....................    5  P7-3 -/-
                      .....................................................................................................................   39  P7-3 -/-
                      ------------------------------------------------------------------------------------------------------
                      ..................GCTGGAGGATGAAGTAAGGAGTGA..............................................................................   18  P7-4 -/-
                      ..................GCTGGAGGATGAAGTAAGGAG.................................................................................    7  P7-4 -/-
                      .............................................................CCACTGTGCTGACACTCACATTGCTGACA.....................    5  P7-4 -/-
                      .....................................................................................................................   30  P7-4 -/-
                      ------------------------------------------------------------------------------------------------------
                      ..................GCTGGAGGATGAAGTAAGGAG.................................................................................    7  P14-4 -/-
                      .............................................................CCACTGTGCTGACACTCACATTGCTGACA.....................    5  P14-4 -/-
                      .....................................................................................................................   12  P14-4 -/-
                      ------------------------------------------------------------------------------------------------------
```

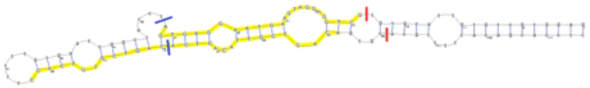

```
high confidence   72  TCTGGATTAGGATGAAAGTGGTAGAGCTTGCATATTTAATCTGGCCTTTTGTGACATGTTTGAAAAGAATATAAGATGCATATTAAGCCAAACCAGTTTTTTCTCTCCCAGA
                      ((((...((((.((((.((((...(((((.((((...((((...(((((..((....))..)))))......))))..)))))))))...)))).)))).)))).)))...)))))
                      ***********TGAAAGTGGTAGAGCTTGCATATTTAATC*********************************************************************************
                      ...........TGAAAGTGGTAGAGCTTGCATATTTAATC.................................................................................    7  P7-1 +/-
                      .....................................................................................................................    7  P7-1 +/-
                      ------------------------------------------------------------------------------------------------------
```

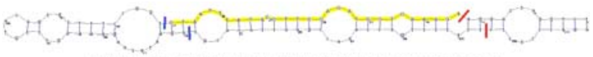

```
potential         73  GGTGTGTCTTCAGCCCCTTGCCATGGGATCTTAGTCATGCAAGTGAGTTTTTTAGAATGCAGATTCTCACCTGCTGCCAGCTTCTTGGATCTGGCACTGTGTTCGAGGCCACC
                      ((((.(((((.(((.(..(((((...(((((.(((((.(((.(((((..(((........)))..))))).)))))...)))....)))))))))..).))).)))))))))
                      **************************************************************************************TTGGATCTGGCACTGTGTTCGAGGC*****
                      ......................................................................................TTGGATCTGGCACTGTGTTCGAGGC.....    5  P14-1 +/-
                      ......................................................................................TTGGATCTGGCACTGTGTTCGAGGCC....    5  P14-1 +/-
                      .....................................................................................................................   10  P14-1 +/-
                      ------------------------------------------------------------------------------------------------------
```

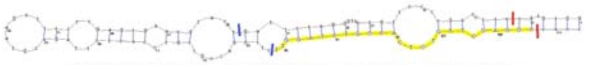

```
high confidence   74  CACAAAGCTTCCCTGCCTACCATCCTCTGACTGATGGTCTCTCAGATGGCTCAGGACAGGCCATGGGCACTCTTGGGGCCAGGTCAGCCCTAGGTGGCTGGCAGGGCAGAGTG
                      ......(((((((((((..(((.(((((((((..(((((((..(((..((((((((.....)).)))))..))).))))))))))))))....))))))..)))))))..)))).
                      *************************TGACTGATGGTCTCTCAGATGGCTCA***************************************************************
                      .........................TGACTGATGGTCTCTCAGATGGCTCA...............................................................    5  P10-1 +/-
                      .....................................................................................................................    5  P10-1 +/-
                      ------------------------------------------------------------------------------------------------------
                      .........................TGACTGATGGTCTCTCAGATGGCTCA...............................................................   10  P10-2 +/-
                      .....................................................................................................................   10  P10-2 +/-
                      ------------------------------------------------------------------------------------------------------
```

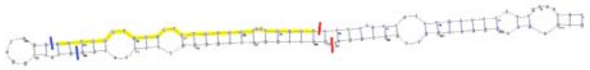

potential

```
75 GCCGGTTGTGAGTGACGATGACAGTGAGGAGGAGCAGGAGGAGGTAAGAGCGCTGCAGCCATCCTCGACTTAGCCAGTCCCATGTCCAGGCCACTCTGACGACAGACTCGGC
   ((((((((((((.(...((((.((.(((.(.(((((..((((...(.((......))).))))..))).))).))))))))..)..))))).))))).....))))
   ********TGAGTGACGATGACAGTGAGGAGGAGC**********************************************************************
   ........TGAGTGACGATGACAGTGAGGAGGAGC......................................................................
   .........................................................................................................
   ---------------------------------------------------------------------------------------------
```

```
5 P7-1 +/-
5 P7-1 +/-
```

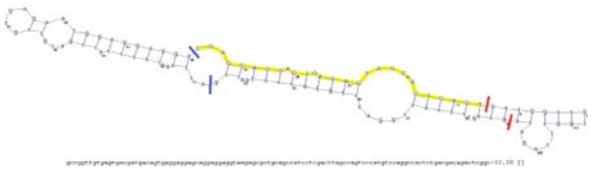

high confidence plus star

```
76 TGTGTTACCCATCAGTAGCAATGGCACAGTTATTTCTTTGGTCATGTCTACTGTCCATGCAGACAGTGCTGTGAGACGCTGAGCCAGTAGGGTACTGGTTGGGAACTGCGCA
   .((((..((((((((((.(..((((.((((..((((..((((..(((((.(.......).)))))..)))).)))).)))).))))....).))))))).))))....)))).
   ***********************************************************************TGTGAGACGCTGAGCCAGTAGGGTACT****************
   ..............TAGCAATGGCACAGTTATTTCTTTGGT..........................................................................
   ..............TAGCAATGGCACAGTTATTTCTTTGGTC.........................................................................
   .......................................................................TGTGAGACGCTGAGCCAGTAGGGTACT................
   .......................................................................TGTGAGACGCTGAGCCAGTAGGGTACTGGT.............
   ....................................................................TGCTGTGAGACGCTGAGCCAGTAGGGT...................
   .......................................................................TGTGAGACGCTGAGCCAGTAGGGTAC.................
   ....................................................................TGCTGTGAGACGCTGAGCCAGTAGGGTA..................
   ..................................................................................................................
   ----------------------------------------------------------------------------------------------------
```

```
6 P7-1 +/-
5 P7-1 +/-
21 P7-1 +/-
18 P7-1 +/-
9 P7-1 +/-
7 P7-1 +/-
6 P7-1 +/-
72 P7-1 +/-
```

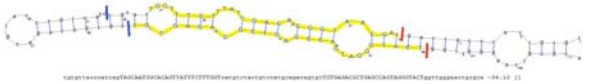

high confidence

```
77 GCCCTGAGCCTTGATGCAGGATGTACTGTAGACAGATGATGGGCCGGGCATCTCATGCTCGCTTGTTCCCTTCATCTTGATCTCTGTGGTTTTCTGTAATGATTTCTGTGGC
   ((((.(((..(((.((((((((...((..(((((((((((.(((.((((((((...))))))))......))).)))))).))...)))))..)).)))))))).))).))).).)))
   *************TGCAGGATGTACTGTAGACAGATGATGG**********************************************************************
   .............TGCAGGATGTACTGTAGACAGATGATGG......................................................................
   ..............................................................................................................
   ----------------------------------------------------------------------------------------------------
```

```
5 P7-1 +/-
5 P7-1 +/-
```

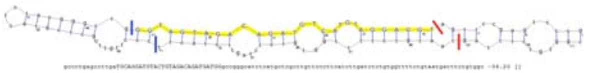

high confidence

```
78 TCTGGAGACTTAAAAGTCCTCAACCAGGGCTCTGGCATCTGGCAATGGCATTTCTTTATGTGACATCCCATAGCTAAAGGCAAGAAGTTGGGGACTGGGTTCAGTTTTTGGA
   ((..((((((.((.((((((((((......(((.((...((((.((((.((.((.......)).)).)))).))))...)).))).))))))))))...)).))))))..))
   **************************************************************************TAAAGGCAAGAAGTTGGGGACTGGGTT*************
   ..........................................................................TAAAGGCAAGAAGTTGGGGACTGGGTT.............
   ......................................................................TAGCTAAAGGCAAGAAGTTGGGGACTG.................
   ..................................................................................................................
   ----------------------------------------------------------------------------------------------------
```

```
12 P14-1 +/-
7 P14-1 +/-
19 P14-1 +/-
```

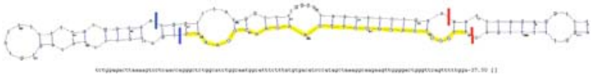

high confidence

```
79 ACTGTCTGTCTTGAATTCTGAAGGGGAACCAAGCCGGTGGGACCCTCCGATGAAACAGGACCCCAGCCGGTTTGATGTCAGTTTCCCAAACAGTGTAATTAAGAGAGACAGT
   (((((((.((((((...(((..((((((((((((((((((..(((..........)))..)))).)))))))))......)))))))...)))......)))))).)))))))
   ***********TGAATTCTGAAGGGGAACCAAGCCGG*****************************************************************************
   ...........TGAATTCTGAAGGGGAACCAAGCCGG.............................................................................
   ..................................................................................................................
   ----------------------------------------------------------------------------------------------------
```

```
5 P10-1 +/-
5 P10-1 +/-
```

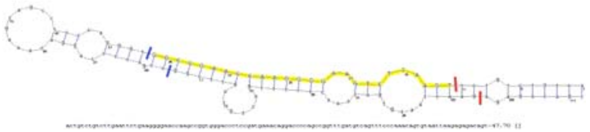

potential

```
80 GCTTTTCCCACTTTCTCCTCTGTAAGTTTCAGTGTCTCTGGTTTTATGTGGAGTTCCTTGATCTACTTAGACTTGAGCGTTGTACAAGGAGATAAGAATGGGTCAATTAGC
   (((...((((((((((((((.((((.....(((((.(((.(((((...(((((.........)))))..))))).))))))))))))))))).))).)))).....)))
   *******************************************************************************TACAAGGAGATAAGAATGGGTCAATTA***
   ...............................................................................TACAAGGAGATAAGAATGGGTCAATTA...
   ...............................................................................TACAAGGAGATAAGAATGGGTCAATTAGC.
   ...............................................................................TACAAGGAGATAAGAATGGGTCAATTAG..
   ...............................................................................TACAAGGAGATAAGAATGGGTCAATT....
   ..............................................................................................................
   ----------------------------------------------------------------------------------------------------
   ...............................................................................TACAAGGAGATAAGAATGGGTCAATTA...
   ..............................................................................................................
   ----------------------------------------------------------------------------------------------------
   ...............................................................................TACAAGGAGATAAGAATGGGTCAATTA...
   ..............................................................................................................
   ----------------------------------------------------------------------------------------------------
   ...............................................................................TACAAGGAGATAAGAATGGGTCAATTA...
   ...............................................................................TACAAGGAGATAAGAATGGGTCAATT....
   ..............................................................................................................
   ----------------------------------------------------------------------------------------------------
```

```
49 P7-1 +/-
13 P7-1 +/-
11 P7-1 +/-
10 P7-1 +/-
83 P7-1 +/-

6 P7-2 +/-
6 P7-2 +/-

21 P10-1 +/-
21 P10-1 +/-

17 P10-2 +/-
5 P10-2 +/-
22 P10-2 +/-
```

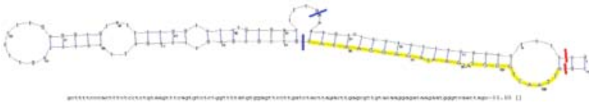

high confidence

```
81 AGGCTTAGGCAAATTCTTAGGACAAGGGTAGAAGGCAGCCATATTCTTTGTCAAAACATAACAAGAATAAGTCTTTAGCCAGTTGTTAATAATTGTTATACCCTGCAGCCT
   ((((((((..(((..(((.(((((.(((.((((((.....((((((((((....)))....))))))).)))))).))).)))))..))).)))....)))).)))))
   ***********AATTCTTAGGACAAGGGTAGAAGGCA*************************************************************************
   ...........AATTCTTAGGACAAGGGTAGAAGGCA.........................................................................
   ..............................................................................................................
   ----------------------------------------------------------------------------------------------------
```

```
5 P10-1 +/-
5 P10-1 +/-
```

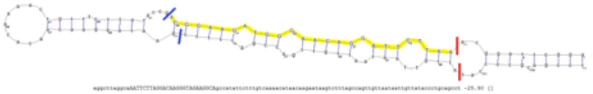

potential

```
82 GAATGCTGCAGAAATCCAGGTAGAAAATCCTTCCATCAATGACAGGTCAAGACGTGTCGAATTGACTTGTAAGATGGTGATCCTCAGCTCCCGGAGTGTCTGTTCAAATTC
   ((((...(((((..(((.((..((..(((...((((....(((((((((..((...))..)))))))))..)))))).)))..))....)).)))...)))))....))))
   *****************************************************************TGACTTGTAAGATGGTGATCCTCAGC*******************
   .................................................................TGACTTGTAAGATGGTGATCCTCAGC...................
   ..............................................................................................................
   ----------------------------------------------------------------------------------------------------
```

```
5 P14-1 +/-
5 P14-1 +/-
```

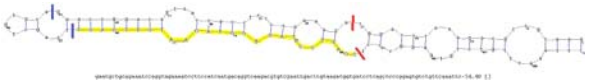

non-candidate

```
83 TTCGTGTGTGCTCACCCAAGGGGAACCTGTGTCTATGTTGGCTTTAGGTGAAATATCCTAAAACTCGAAATAGCACTCAGGCTCTGGAGGTCTCAATGATGCACACATGGG
   .(((((((((((((((...((((..((((.((((((.((((.(((((((........)))))).).))).)))).)).))))))))...)))......)).))))))))).
   *********************************************************TCCTAAAACTCGAAATAGCACTCAGGCT*************************
   .........................................................TCCTAAAACTCGAAATAGCACTCAGGCT.........................
   ..............................................................................................................
   ----------------------------------------------------------------------------------------------------
```

```
5 P14-1 +/-
5 P14-1 +/-
```

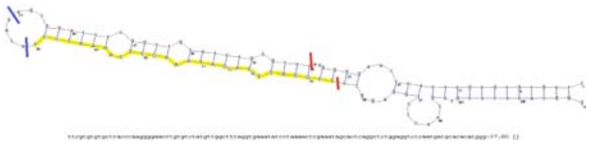

```
high confidence   84  TGTGGTACTTCTGGCCATCGGCATGTTCTTCACTGCCTGGTTCTTCGTGTATCCTTTATTTGCAGCCAGGAGGCAGAATTAGTGACCATGGAAGCGAGAGAGAAACCACA
                      .((((..((((.(((((.(((((((((.(.((.((((((((...((.(((.....))).)).))))))))))).)))))..))).)))))...)).)))....))))).
                      **TGGTACTTCTGGCCATCGGCATGTTCTT**********************************************************************************
                      ..TGGTACTTCTGGCCATCGGCATGTTCTT..................................................................................   6 P10-2 +/-
                      ................................................................................................................   6 P10-2 +/-
                      ----------------------------------------------------------------------------------------------------------
```

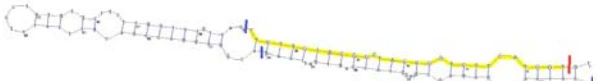

```
high confidence   85  ATCCAAGTCATCTGTCAGATAAAGTGAGTTTGGTTTTGAGGTGACAGCTCAGTGTGGCCCAGGTTGGTCTCAAACGCCAGGCCTCAGTGGGTCTTCCCAGTTGACTGGAT
                      ((((.((((.(((..((((..(.((((((((((((((((...(((((..(......)..))))).))))))).)))))).)))).)..))))...))).)))))))))
                      *******************TAAAGTGAGTTTGGTTTTGAGGTGA*********************************************************************
                      ...................TAAAGTGAGTTTGGTTTTGAGGTGACAGC.................................................................   9 P7-1 +/-
                      ................................................................................................................   9 P7-1 +/-
                      ----------------------------------------------------------------------------------------------------------
                      ...................TAAAGTGAGTTTGGTTTTGAGGTGACAGC.................................................................   6 P10-1 +/-
                      ................................................................................................................   6 P10-1 +/-
                      ----------------------------------------------------------------------------------------------------------
                      ...................TAAAGTGAGTTTGGTTTTGAGGTGA.....................................................................   10 P10-2 +/-
                      ................................................................................................................   10 P10-2 +/-
                      ----------------------------------------------------------------------------------------------------------
                      ...................TAAAGTGAGTTTGGTTTTGAGGTGACAGC.................................................................   7 P14-1 +/-
                      ................................................................................................................   7 P14-1 +/-
                      ----------------------------------------------------------------------------------------------------------
```

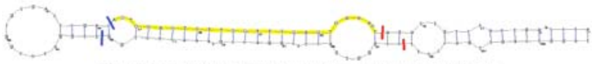

```
non-candidate     86  GGATCCCGAAAGTACAATACTTGGCATCTCAAGTGAAGTGTGGGGGAAGGGCCATCAGCTGCTAATGCCAGGCACACAGTGAGAGTTCTGTGTCTGTATTGATGGGGTCC
                      (((((((..(((((((((..(((.(((((.(((..((.(((.(..(((((.....))).))..).))).)).)))..))))))))..)))).))))))..))))))))
                      *******************************************************************************TGAGAGTTCTGTGTCTGTATTGATGGG*****
                      ...............................................................................TGAGAGTTCTGTGTCTGTATTGATGGG.....   10 P7-1 +/-
                      ................................................................................................................   10 P7-1 +/-
                      ----------------------------------------------------------------------------------------------------------
                      ...............................................................................TGAGAGTTCTGTGTCTGTATTGATGGG.....   5 P10-2 +/-
                      ................................................................................................................   5 P10-2 +/-
                      ----------------------------------------------------------------------------------------------------------
```

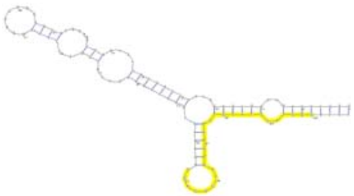

```
non-candidate     87  AGAAATGCAGGGCAGAGCCTCAGTGACCTAGAACTCTGTGCACCTGGCTGGCACAGACTGCAGTCCAGGTTGGGTGGTTCTGGTCTTGTGTAGTTTGCCTCATCACTTCT
                      ((((.((..((((((((..(((.((((.((((((.(.((.(((((((((.((.....))))).)))))))).).))))))))))))).)).)))))))...)).))))
                      *****************************TAGAACTCTGTGCACCTGGCTGGCAC**********************************************************
                      .............................TAGAACTCTGTGCACCTGGCTGGCACA.........................................................   7 P10-1 +/-
                      ................................................................................................................   7 P10-1 +/-
                      ----------------------------------------------------------------------------------------------------------
                      .............................TAGAACTCTGTGCACCTGGCTGGCAC..........................................................   11 P10-2 +/-
                      .............................TAGAACTCTGTGCACCTGGCTGGCACA.........................................................   7 P10-2 +/-
                      ................................................................................................................   18 P10-2 +/-
                      ----------------------------------------------------------------------------------------------------------
```

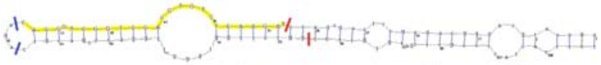

```
high confidence   88  GAGATGGCTGTCATGGGATAGAAGGAAATGCAGATGAGGCAGAGGGGCCTAGGGAAGCCCACTGGGCTGCTGATTTAGAAGCAGGCACTTCTATCCTATGATGGGTACTC
                      (((.((.((((((((((((((((....(((...((((.(((...((((((((.....)).))))))).))).))))...)))....))))))))))))))))).)))))
                      *************TGGGATAGAAGGAAATGCAGATGAGGCA************************************************************************
                      .............TGGGATAGAAGGAAATGCAGATGAGGCA........................................................................   39 P14-1 +/-
                      .............TGGGATAGAAGGAAATGCAGATGAGGC.........................................................................   21 P14-1 +/-
                      .............TGGGATAGAAGGAAATGCAGATGAGG..........................................................................   10 P14-1 +/-
                      ..................TAGAAGGAAATGCAGATGAGGCAGA......................................................................   9 P14-1 +/-
                      ..................TAGAAGGAAATGCAGATGAGGCAGAG.....................................................................   8 P14-1 +/-
                      ..................TAGAAGGAAATGCAGATGAGGCAGAGG....................................................................   6 P14-1 +/-
                      ..................TAGAAGGAAATGCAGATGAGGCAGAGGG...................................................................   5 P14-1 +/-
                      ................................................................................................................   98 P14-1 +/-
                      ----------------------------------------------------------------------------------------------------------
```

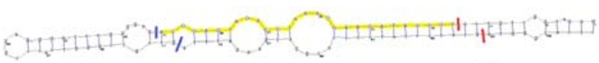

```
non-candidate     89  GTTGAGCTGTCCCAGGTAAAGGCTGGGCTTAGACGTGGCCTTTGGGTGTGGAATGCACTTCCGTTTGTAACCGCCATCTAACCCTGGCCTTTGACAGGTGCGTTCTGAC
                      ...((((.(..((...(((((((..((.(((((..((((..(((.(..(((((.....)))))..).)))..)))))))))).))..)))))))...))..)))))....
                      ***************GTAAAGGCTGGGCTTAGACGTGG***************************************************************************
                      ...............GTAAAGGCTGGGCTTAGACGTGG...........................................................................   1954 P7-4 -/-
                      ...............GTAAAGGCTGGGCTTAGACGTG............................................................................   346 P7-4 -/-
                      ...............GTAAAGGCTGGGCTTAGACGTGGC..........................................................................   83 P7-4 -/-
                      .........................................................................CATCTAACCCTGGCCTTTGACA..................   5 P7-4 -/-
                      ................................................................................................................   2388 P7-4 -/-
                      ----------------------------------------------------------------------------------------------------------
                      ...............GTAAAGGCTGGGCTTAGACGTGG...........................................................................   370 P10-1 +/-
                      ...............GTAAAGGCTGGGCTTAGACGTG............................................................................   154 P10-1 +/-
                      ...............GTAAAGGCTGGGCTTAGACGTGGC..........................................................................   11 P10-1 +/-
                      .........................................................................CATCTAACCCTGGCCTTTGACA..................   5 P10-1 +/-
                      ................................................................................................................   540 P10-1 +/-
                      ----------------------------------------------------------------------------------------------------------
                      ...............GTAAAGGCTGGGCTTAGACGTGG...........................................................................   895 P10-4 -/-
                      ...............GTAAAGGCTGGGCTTAGACGTG............................................................................   338 P10-4 -/-
                      ...............GTAAAGGCTGGGCTTAGACGTGGC..........................................................................   48 P10-4 -/-
                      .........................................................................CATCTAACCCTGGCCTTTGACA..................   5 P10-4 -/-
                      ................................................................................................................   1286 P10-4 -/-
                      ----------------------------------------------------------------------------------------------------------
```

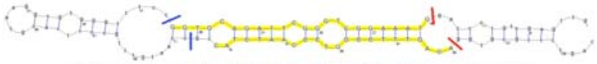

```
potential         90  GGTGGGGGGTTTGGAGAGTTAAAATTTGGTATTGAGTAGCATTTATGTCTAACAAGCATGAATAATTAGCCTTAGGTGATTGGGTTTTGCGGACTAATGATTCTTCACC
                      ((((((((((...((.((.((((((..((.(((((..(((((((((.......))))))))).....)))))))...)).)))))))))...))...)))))))))))
                      ********************************************************************GCCTTAGGTGATTGGGTTTTGCGGAC*****************
                      ....................................................................GCCTTAGGTGATTGGGTTTTGCGGAC.................   5 P7-4 -/-
                      ................................................................................................................   5 P7-4 -/-
                      ----------------------------------------------------------------------------------------------------------
```

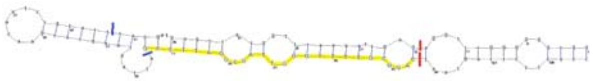

```
high confidence   91  CTGATGGAGTTGAGGCTGTAAACAAACCACCGGGGACTGCGGCTTTCAGTTAAAGGTCACTGTTTGCTGTTTGTCCTCCCAGGAGTGGTTTTGGCCAGCATCTCCTCAG
                      ((((.((((.((.(((((.....((((((((((((((.(((((...((((.........))))...)))))..)))))).....).))))))))))))..)).))))))))
                      ****************************ACCGGGGACTGCGGCTTTCAGT***************************************************************
                      ............................ACCGGGGACTGCGGCTTTCAGT...............................................................   7 P7-4 -/-
                      ................................................................................................................   7 P7-4 -/-
                      ----------------------------------------------------------------------------------------------------------
                      ............................ACCGGGGACTGCGGCTTTCAGT...............................................................   5 P10-4 -/-
                      ................................................................................................................   5 P10-4 -/-
                      ----------------------------------------------------------------------------------------------------------
                      ............................ACCGGGGACTGCGGCTTTCAGT...............................................................   5 P14-3 -/-
                      ................................................................................................................   5 P14-3 -/-
                      ----------------------------------------------------------------------------------------------------------
```

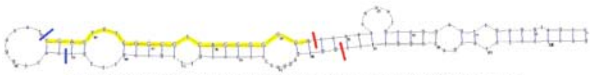

high confidence

```
92 GCTCTCGTCTACATCTGGCTCCACTCTCTCATTGGCTGTCTGCCGCCCCTATTTGGTTGGTCATCGGTGGAGTTTGATGAGTTCAAGTGGATGTGTGTGGCCGCGTGGC
   ((((.((.((((((...((((((((..(((((..(...((..((((((((.....))..))....)))..))..)..)))))....))))))).))))))))).)).).)))
   *****************************************************************************TGAGTTCAAGTGGATGTGTGTGGCC********
   .............................................................................TGAGTTCAAGTGGATGTGTGTGGCC........
   .............................................................................................................
   ---------------------------------------------------------------------------------------------------------
```

6 P7-1 +/-
6 P7-1 +/-

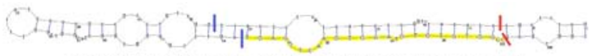

potential

```
93 CTTGGCCAAGTATGCTGACCCTGTGACTGATCTCCTGGACCGGTGCGGGTGTTCCGGGCCCGCCTGTTCCGAGAGGCTTGTGTGTTCCACCAGGGCTGCTATGTCAAG
   (((((...(((.((.(.((((((((.(..(((.((((.(((.(((((.(......).)))))))).)))).)))..).))......)).)))))).)))))))))))
   *********************TGTGACTGATCTCCTGGACCGGTGC**************************************************************
   .....................TGTGACTGATCTCCTGGACCGGTGC..............................................................
   .....................TGTGACTGATCTCCTGGACCGGTGCGG............................................................
   ............................................................................................................
   --------------------------------------------------------------------------------------------------------
   .....................TGTGACTGATCTCCTGGACCGGTGC..............................................................
   .....................TGTGACTGATCTCCTGGACCGGTGCGG............................................................
   ............................................................................................................
   --------------------------------------------------------------------------------------------------------
```

6 P10-1 +/-
5 P10-1 +/-
11 P10-1 +/-

14 P10-2 +/-
6 P10-2 +/-
20 P10-2 +/-

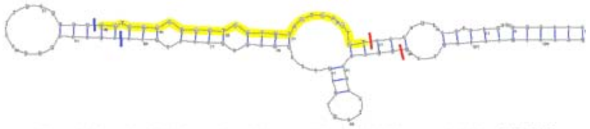

high confidence

```
94 CTGCAGAGTAAATCTGACTAGAATCATGGCATGGGAGGCAGCAGCTTTTCTGTCCAAGGACTGCAGTAGCTGCTCATGAGCTGTGAGGACCTGGTGGGTATACAGGCAG
   ((((...(((.((((.(((((..(((((((.(.(..((((((.(((.....(((....)))...))).))))))..).))))))))....))))))))).)))..))))
   **************TGACTAGAATCATGGCATGGGAGGCA*********************************************************************
   ..............TGACTAGAATCATGGCATGGGAGGCA.....................................................................
   ..............TGACTAGAATCATGGCATGGGAGGCAGC...................................................................
   ..............TGACTAGAATCATGGCATGGGAGGCAG....................................................................
   ..............TGACTAGAATCATGGCATGGGAGGC......................................................................
   ............TCTGACTAGAATCATGGCATGGGAGGC......................................................................
   .............................................................................................................
   ---------------------------------------------------------------------------------------------------------
```

16 P7-1 +/-
16 P7-1 +/-
10 P7-1 +/-
8 P7-1 +/-
7 P7-1 +/-
57 P7-1 +/-

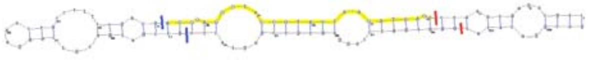

potential

```
95 TTCTGTCATTCTGCTCTGTTCTTTCCTCTGAAATGTCTGTATGCAGACCACATCGCCCTGATCATAGAACTTCTGGGGAAGGTGCCTCGCAAGCTCATTGTGGCAGGA
   ((((((((...(((.(((.((((((((.(((...(((((...(((..(.....)..)))...)))))..))).))))))))......))))))....)))))))))
   **********************************************************TGATCATAGAACTTCTGGGGAAGGTGCC**********************
   ..........................................................TGATCATAGAACTTCTGGGGAAGGTGCC......................
   ............................................................................................................
   --------------------------------------------------------------------------------------------------------
```

7 P7-1 +/-
7 P7-1 +/-

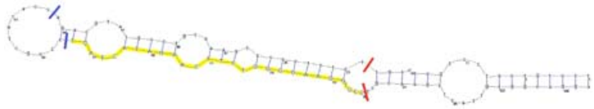

high confidence

```
96 GATGAGGGCTGAGTTGGTTCCTCTTAGAGCTTCCTTGTTGTCCTGAGGCAGAGTTCCTGGGTGATCTTGAGTTTGCTGTGTAGAGGATGGCTCCTAATTTTTCACATC
   ((.(((....(((((...((((((((.(((...((((..(((((.(((.......))).)).))).)..))))...))).)).))))))).)))))....))).))....
   *******************************************************************TGAGTTTGCTGTGTAGAGGATGGCTCC**************
   ...................................................................TGAGTTTGCTGTGTAGAGGATGGCTCC..............
   ............................................................................................................
   --------------------------------------------------------------------------------------------------------
```

5 P10-2 +/-
5 P10-2 +/-

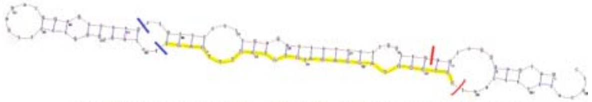

high confidence

```
97 AGGGACAATGGTTTCCATGTCAGCGACAAACGCACTTCAGGTAGTTCTTGGACCTGATAGCTTTAAGATTGCGGAGGAGCGAGAAGGTAGCTGAGGGAGCCTGTGCCT
   (((.(((..(((((((...(((((.((...(((.((((..(((((.((((((.(.....).)))))))))))).)))).)))......)).)))))))))))))))).)))
   ***************************************************************TAAGATTGCGGAGGAGCGAGAAGGTA*******************
   ...............................................................TAAGATTGCGGAGGAGCGAGAAGGTA...................
   ...............................................................TAAGATTGCGGAGGAGCGAGAAGGT....................
   ............................................................................................................
   --------------------------------------------------------------------------------------------------------
   ...............................................................TAAGATTGCGGAGGAGCGAGAAGGTA...................
   ...............................................................TAAGATTGCGGAGGAGCGAGAAGGT....................
   ............................................................................................................
   --------------------------------------------------------------------------------------------------------
   ...............................................................TAAGATTGCGGAGGAGCGAGAAGGTA...................
   ...............................................................TAAGATTGCGGAGGAGCGAGAAGGT....................
   ............................................................................................................
   --------------------------------------------------------------------------------------------------------
```

37 P7-1 +/-
8 P7-1 +/-
45 P7-1 +/-

13 P10-1 +/-
6 P10-1 +/-
19 P10-1 +/-

12 P10-2 +/-
10 P10-2 +/-
22 P10-2 +/-

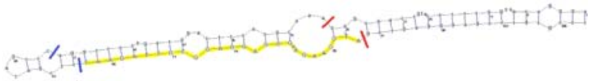

non-candidate

```
98 GCCATATTATCACTGTGGAATCCAGACTGAGCAGACAGAGCTCCCACCTCCATGAGGCTCTCCTGGCCGTGCTCAGTCTTGGCATTCATTCTGTGAGGGAGATTGGT
   ((((..((.((((.(..((((((((((((((((.(((((..((((......)).))..)).)))..).)))))))))).))).))))...).))))..))...))))
   *************TGTGGAATCCAGACTGAGCAGACAGAGC******************************************************************
   .............TGTGGAATCCAGACTGAGCAGACAGA....................................................................
   ...........................................................................................................
   -------------------------------------------------------------------------------------------------------
   .............TGTGGAATCCAGACTGAGCAGACAGAGC..................................................................
   ...........................................................................................................
   -------------------------------------------------------------------------------------------------------
   .............TGTGGAATCCAGACTGAGCAGACAGAGC..................................................................
   ...........................................................................................................
   -------------------------------------------------------------------------------------------------------
```

5 P7-1 +/-
5 P7-1 +/-

6 P10-1 +/-
6 P10-1 +/-

8 P10-2 +/-
8 P10-2 +/-

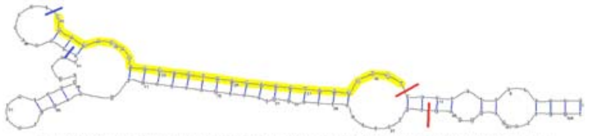

high confidence

```
99 TGCTCTTGCAGAGTACCCAAGTTTGGATTTCAGTAACCATGTCAGGTGTCTCTCTCGCCACCCTTCCATGGTGCAAATGAATGTTCTGGCGGATGCTCTCAAGAGCA
   .(((((((.((((((.((..((..((((((((...((((((..(((((.(......).)).)))..)))))))....)))).)))).)))).)))))))))))))).
   *****************************************************************************TGAATGTTCTGGCGGATGCTCTCAAG*****
   .............................................................................TGAATGTTCTGGCGGATGCTCTCAAG.....
   ...........................................................................................................
   -------------------------------------------------------------------------------------------------------
```

6 P7-1 +/-
6 P7-1 +/-

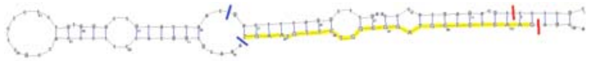

high confidence

```
100 GCTGGACAAGCTGATTGAAGGAACACACCGCCTCCCCTGGCAGCAGAGCCCTCGGTGTCTCTGCCCCTTTTGGGATTGTGGTGGCATTTCTCGGAGCTTGTCACGGC
    ((((((((((((..((((.((((..((((((.((((..((..((((((((....).).)))))))..))...)))).)))))))...))))))))))))))).))))
    **TGGACAAGCTGATTGAAGGAACACACCGCC*****************************************************************************
    ..TGGACAAGCTGATTGAAGGAACACACCGCC...     46 P7-1 +/-
    ..TGGACAAGCTGATTGAAGGAACACACC...        24 P7-1 +/-
    ..TGGACAAGCTGATTGAAGGAACACACCG...       12 P7-1 +/-
    ..TGGACAAGCTGATTGAAGGAACACACCGC...       9 P7-1 +/-
    ...TGGGATTGTGGTGGCATTTCTCGGA...         34 P7-1 +/-
    ...TGGGATTGTGGTGGCATTTCTCGGAG...         6 P7-1 +/-
    ...TGGGATTGTGGTGGCATTTCTCGGAGC...        6 P7-1 +/-
    ...                                    137 P7-1 +/-
    ----------------------------------------
    ..TGGACAAGCTGATTGAAGGAACACACC...        22 P10-1 +/-
    ..TGGACAAGCTGATTGAAGGAACACACCGCC...     19 P10-1 +/-
    ..TGGACAAGCTGATTGAAGGAACACACCG...       14 P10-1 +/-
    ...                                     79 P10-1 +/-
    ...TGGGATTGTGGTGGCATTTCTCGGA...         24 P10-1 +/-
    ----------------------------------------
    ..TGGACAAGCTGATTGAAGGAACACACC...        22 P10-2 +/-
    ..TGGACAAGCTGATTGAAGGAACACACCGCC...     14 P10-2 +/-
    ....GACAAGCTGATTGAAGGAACACACCGCC...      5 P10-2 +/-
    ...TGGGATTGTGGTGGCATTTCTCGGA...         24 P10-2 +/-
    ...                                     65 P10-2 +/-
    ----------------------------------------
    ..TGGACAAGCTGATTGAAGGAACACACC...         8 P14-1 +/-
    ...TGGGATTGTGGTGGCATTTCTCGGA...          8 P14-1 +/-
    ...                                     16 P14-1 +/-
    ----------------------------------------
```

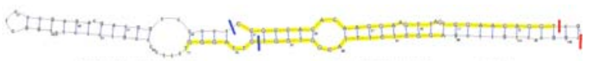

high confidence

```
101 CTTTTCTGGGTCATGGGAACAGTAGCTTGCAGTGACTGGCTCCAGGGTGAGGCCTAGGACCTTACCCTGGAGGGACTGCTGCAGAGGACTGGGACCTCCCAGAAAAG
    ((((((((((....((...((((...((((((......(((((((((((((....).)))))))))))))).....))))))...))))...)).))))))))))
    ***************************************************************TACCCTGGAGGGACTGCTGCAGAGGACT****************
    ...TACCCTGGAGGGACTGCTGCAGAGGACT...       5 P7-1 +/-
    ...                                      5 P7-1 +/-
    ----------------------------------------
```

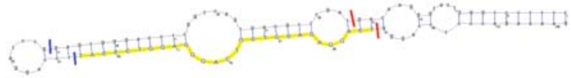

potential

```
102 GCCAGGCTGAACTGGAAGTGGCAAGTGTTGAGAACCAAGGTTTGGCCAAGTGTGATGGCTCACACCTGTGACTTTGGTACTTGAGAAGCTGGGACAGGAGGATGGC
    ((((..((...(((.....(((...(.(..((.(((((((((....((.((((((....)))))).)).))))))))).))..).).)))....)))..)).))))
    ***************************************************************TGTGACTTTGGTACTTGAGAAGCTGG***************
    ...TGTGACTTTGGTACTTGAGAAGCTGG...        28 P7-1 +/-
    ...TGACTTTGGTACTTGAGAAGCTGGGA...         8 P7-1 +/-
    ...TGTGACTTTGGTACTTGAGAAGCTG...          6 P7-1 +/-
    ...TGTGACTTTGGTACTTGAGAAGCTGGG...        5 P7-1 +/-
    ...                                     47 P7-1 +/-
    ----------------------------------------
    ...TGTGACTTTGGTACTTGAGAAGCTGG...         6 P7-2 +/-
    ...                                      6 P7-2 +/-
    ----------------------------------------
    ...TGTGACTTTGGTACTTGAGAAGCTGG...        31 P10-1 +/-
    ...TGTGACTTTGGTACTTGAGAAGCTG...         13 P10-1 +/-
    ...TGACTTTGGTACTTGAGAAGCTGGGAC...       11 P10-1 +/-
    ...TGACTTTGGTACTTGAGAAGCTGGGA...         5 P10-1 +/-
    ...                                     60 P10-1 +/-
    ----------------------------------------
    ...TGTGACTTTGGTACTTGAGAAGCTGG...        39 P10-2 +/-
    ...TGACTTTGGTACTTGAGAAGCTGGGA...         5 P10-2 +/-
    ...TGACTTTGGTACTTGAGAAGCTGGGAC...        5 P10-2 +/-
    ...TGTGACTTTGGTACTTGAGAAGCTG...          5 P10-2 +/-
    ...                                     54 P10-2 +/-
    ----------------------------------------
    ...TGTGACTTTGGTACTTGAGAAGCTGG...        15 P14-1 +/-
    ...                                     15 P14-1 +/-
    ----------------------------------------
```

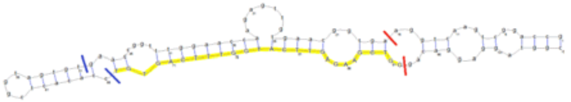

potential

```
103 GAGGCCCAGTTCACTGCAGGGGTCTCTAAACACTCTTGTGACCTGCATTTGTCTGGTGATGGGCTGTCATAGTGGTGTGATCTTCAGGATGATCTGGCACTGCCTC
    ((((((((.(((((...((((((.....(((((.((((((((..((..(..((....))..))).)))))))).))))))))))..)).))).)))))....)))))
    **********************************************************TGGGCTGTCATAGTGGTGTGATCTTC**********************
    ...TGGGCTGTCATAGTGGTGTGATCTTC...         8 P7-1 +/-
    ...                                      8 P7-1 +/-
    ----------------------------------------
    ...TGGGCTGTCATAGTGGTGTGATCTTC...        10 P10-1 +/-
    ...                                     10 P10-1 +/-
    ----------------------------------------
    ...TGGGCTGTCATAGTGGTGTGATCTTC...         9 P10-2 +/-
    ...                                      9 P10-2 +/-
    ----------------------------------------
    ...TGGGCTGTCATAGTGGTGTGATCTTC...         5 P14-1 +/-
    ...                                      5 P14-1 +/-
    ----------------------------------------
```

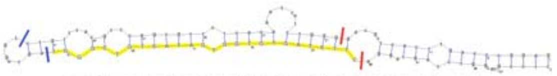

high confidence

```
104 TGATATAGTGAGAATGCTTGCTGGTCTGTTGTGATGGAGTGCTTTTTCAAATGGACAATGTGCTCCTTAGCCTTGCAGATTAGAGAGACATTCCTCAATGGATCA
    .(((....((((((((((..(((((((((.((...((((..(((.(((....))).)).)..))))...))...)))))))))..)).)))).)))).....))).
    *************TGCTTGCTGGTCTGTTGTGATGGAG*********************************************************************
    .............TGCTTGCTGGTCTGTTGTGATGGAG...    5 P7-1 +/-
    ...                                          5 P7-1 +/-
    ----------------------------------------
    .............TGCTTGCTGGTCTGTTGTGATGGAG...    5 P10-1 +/-
    ...                                          5 P10-1 +/-
    ----------------------------------------
    .............TGCTTGCTGGTCTGTTGTGATGGAG...    6 P10-2 +/-
    ...                                          6 P10-2 +/-
    ----------------------------------------
```

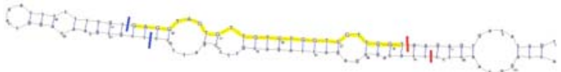

potential

```
105 GCTGACAAATTAGTTATTCACATGGGGACTTGGAAGGCACTTTCTTAAAAAATTAACAAAGTGAGCCTATTCTATCCAGGACAGAAAATGACATAGTTTGTTGC
    ((.((((((((((((((....((....(((((((((((((((.((((....)))).)))))..))))......)))))).))...)))))).)))))))))))
    ***************************************************************TTCTATCCAGGACAGAAAATGACATA**********
    ...TTCTATCCAGGACAGAAAATGACATA...         30 P14-1 +/-
    ...TATCCAGGACAGAAAATGACATAGTTTGTT...     20 P14-1 +/-
    ...TATCCAGGACAGAAAATGACATAGTTTGT...      18 P14-1 +/-
    ...TATCCAGGACAGAAAATGACATAGTTTG...       13 P14-1 +/-
    ...TTCTATCCAGGACAGAAAATGACATAGTTT...      8 P14-1 +/-
    ...TCCAGGACAGAAAATGACATAGTTTGTT...        5 P14-1 +/-
    ...                                      94 P14-1 +/-
    ----------------------------------------
```

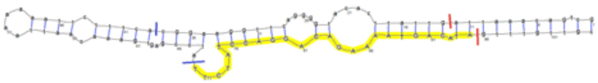

high confidence

```
106 TCAGCAAAGCTTTTGTTGTTGGGTTTTTTAGTTGTTGTTTGTTTTCCTGTTAGATTATCATGGCACAACTTGAGGACATGGGCATGTGGCTGAGGTGCTGA
    (((((.((((.....((((.(((((((.((((((.((..(..((..((......))..)).)..)))))))).)))))).).))))...)))).....))))))
    ************************************************************TGGCACAACTTGAGGACATGGGCATG****************
    ...TGGCACAACTTGAGGACATGGGCATG...          7 P10-2 +/-
    ...                                       7 P10-2 +/-
    ----------------------------------------
```

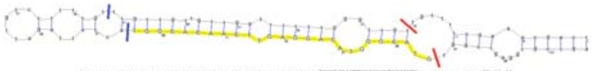

high confidence

```
107 CCTTTCCCCACTTGTGGTCATGGTGTTTTTACCACAGCAATGGAAACCCTAACTAAGACATTGCTTTGGAAAGACCTGATCATGAGTAGAAGCAGTAGGAAGG
    (((((..(.(((.((.((((((((((((((.(((.(((((((...............))))))).)))))))))))...))))))).)).))).)..))))))
    ***************************************************************TGGAAAGACCTGATCATGAGTAGAAGC***********
    ...TGGAAAGACCTGATCATGAGTAGAAGC...         7 P7-1 +/-
    ...                                       7 P7-1 +/-
    ----------------------------------------
```

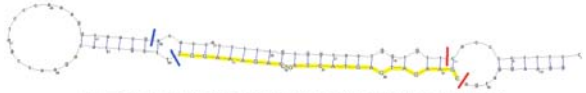

non-candidate

```
108 GGTCTGTCCCCAGTAAGCTCACCTGCCTGGGTTCTCTGCCTCCTCTGACCTGCTTGGTTGAGTCCTAGAACGCAGGCCTGGTTGCCCCTATGGGGACTCGGCT
    ((((.(((((((....((..(((.(((((.(((((..(.(((....((((.....))))))).)..))))).)))))..))).)).....)))))))..))))
    *****************************************************TTGGTTGAGTCCTAGAACGCAGGCC*************************
    .....................................................TTGGTTGAGTCCTAGAACGCAGGCC.........................   17 P14-1 +/-
    .......................................................................................................   17 P14-1 +/-
    -------------------------------------------------------------------------------------------------------
```

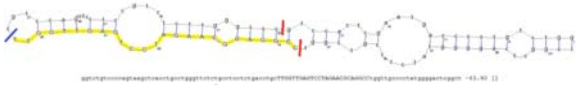

potential

```
109 GCTGACCTTGGGACCCTGGTGAAAGTGAAGGACTGAAAGCCCTGCGCTCCATTTGGAAGGAGCAATGGGCTCTGCTTGACTAGAAGGTCCCTTTAGTGTTGGC
    ((..((((.((((((((((..((((...(((......((((...(((((.........)))))...)))))))))))).)))))..)))))))...)).))..))
    ********TGGGACCCTGGTGAAAGTGAAGGACT*********************************************************************
    ........TGGGACCCTGGTGAAAGTGAAGGACT.....................................................................   7 P10-2 +/-
    .......................................................................................................   7 P10-2 +/-
    -------------------------------------------------------------------------------------------------------
```

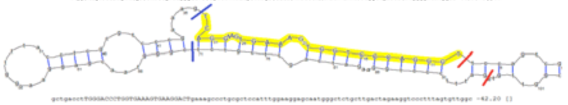

potential

```
110 TGTCAGCCATTTCTAGTAGACTGGCTCTTGATGGTTTGTGCTGTGCACCCCTGCCCACCATAATGCAATCTTCTCTGCTTGTCTGCCTTGGGGACAGTTGATA
    ((((((..(..((((((((.(((....(((.(((((((.((.(((....))).)).)))))).)).)))......))).))))))..)))..)..)))))))
    *************TAGTAGACTGGCTCTTGATGGTTTGT****************************************************************
    .............TAGTAGACTGGCTCTTGATGGTTTGT................................................................   16 P7-1 +/-
    ..........TTCTAGTAGACTGGCTCTTGATGGTT...................................................................   9 P7-1 +/-
    .............TAGTAGACTGGCTCTTGATGGTTTG.................................................................   7 P7-1 +/-
    .......................................................................................................   32 P7-1 +/-
    -------------------------------------------------------------------------------------------------------
    .............TAGTAGACTGGCTCTTGATGGTTTGT................................................................   5 P7-2 +/-
    .......................................................................................................   5 P7-2 +/-
    -------------------------------------------------------------------------------------------------------
    .............TAGTAGACTGGCTCTTGATGGTTTGT................................................................   17 P10-1 +/-
    .............TAGTAGACTGGCTCTTGATGGTTTG.................................................................   13 P10-1 +/-
    ..........TTCTAGTAGACTGGCTCTTGATGGTT...................................................................   13 P10-1 +/-
    .......................................................................................................   43 P10-1 +/-
    -------------------------------------------------------------------------------------------------------
    .............TAGTAGACTGGCTCTTGATGGTTTGT................................................................   30 P10-2 +/-
    .............TAGTAGACTGGCTCTTGATGGTTTG.................................................................   14 P10-2 +/-
    ..........TTCTAGTAGACTGGCTCTTGATGGTT...................................................................   10 P10-2 +/-
    .........TTTCTAGTAGACTGGCTCTTGATGGTT...................................................................   5 P10-2 +/-
    .......................................................................................................   59 P10-2 +/-
    -------------------------------------------------------------------------------------------------------
    .............TAGTAGACTGGCTCTTGATGGTTTGT................................................................   63 P14-2 +/-
    .............TAGTAGACTGGCTCTTGATGGTTTG.................................................................   8 P14-2 +/-
    .......................................................................................................   71 P14-2 +/-
    -------------------------------------------------------------------------------------------------------
    .............TAGTAGACTGGCTCTTGATGGTTTGT................................................................   168 P14-1 +/-
    .............TAGTAGACTGGCTCTTGATGGTTTG.................................................................   23 P14-1 +/-
    ..........TTCTAGTAGACTGGCTCTTGATGGTT...................................................................   11 P14-1 +/-
    .........TTTCTAGTAGACTGGCTCTTGATGGTT...................................................................   7 P14-1 +/-
    .............TAGTAGACTGGCTCTTGATGGTTTGTG...............................................................   5 P14-1 +/-
    .............TAGTAGACTGGCTCTTGATGGTTTGTGCT.............................................................   5 P14-1 +/-
    ..........TTCTAGTAGACTGGCTCTTGATGGT....................................................................   5 P14-1 +/-
    ..........TTCTAGTAGACTGGCTCTTGATGGTTT..................................................................   5 P14-1 +/-
    ..........TTCTAGTAGACTGGCTCTTGATGGTTTGT................................................................   5 P14-1 +/-
    .......................................................................................................   234 P14-1 +/-
    -------------------------------------------------------------------------------------------------------
```

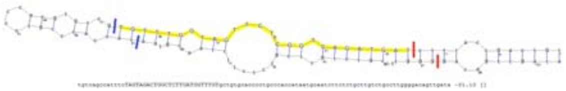

high confidence

```
111 CCAGCTCGAGGACCTATGGGTGTCTTACCTGGGATTTGTTTACAATCTAACACCGCTTGTCGAGGAATTCAAAGGTAAGGGACACACGGTAGGGAAGGGCTGG
    ((((((.....(((((.(((((((((((.((((((.((.((((.(.......).)))).)).)))))))..))))).))))))).).))))...)))))))
    ****************TGGGTGTCTTACCTGGGATTTGTTTAC************************************************************
    ................TGGGTGTCTTACCTGGGATTTGTTTAC............................................................   8 P10-1 +/-
    .......................................................................................................   8 P10-1 +/-
    -------------------------------------------------------------------------------------------------------
```

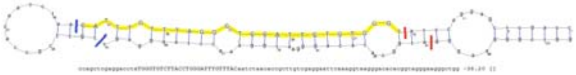

high confidence

```
112 AGGGCAGAGTATGAGACAGTGGACCTAGTTGGGGTGCTGAGGCAAACACTTAAGCTTCCTGCCTGCCTGCACACTGAGAATGTAACTGGCTCGCTCATCCCT
    ((((..((((..(((.((((..((....((.((((((.((((((...............))))).)..)))).)).))...)).)))).)))))))..))))
    **************************************************************************TGAGAATGTAACTGGCTCGCTCATCCC**
    ..........................................................................TGAGAATGTAACTGGCTCGCTCATCCC..   12 P14-1 +/-
    ......................................................................................................   12 P14-1 +/-
    ------------------------------------------------------------------------------------------------------
```

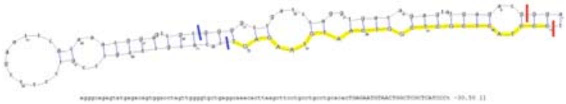

potential

```
113 GACTGCCAGCAGCTACTGGACATTTGCAGGACACTGGAGGAATTGGGCTTTCAGCTCAGCGTGGCAGTGTCTTGAGACAGTGTTCACATTGGCCTGGTTGTC
    (((.((((..((((.((((((((..((((((((((......((((((.....))))))).....))))))))))....))))))))...))))))))).)))
    *************TACTGGACATTTGCAGGACACTGGAGGA*************************************************************
    .............TACTGGACATTTGCAGGACACTGGAGGA.............................................................   7 P7-1 +/-
    ......................................................................................................   7 P7-1 +/-
    ------------------------------------------------------------------------------------------------------
```

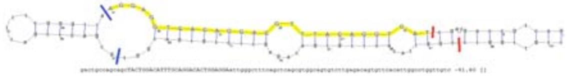

high confidence

```
114 TCCCTGCTGGTCTCTTTTCTGACAACTCTTTCAGATTAAGTCAGTGGCAAAACAGACCCCTTATTTCCTGAAGGTCACAGAACAGGCAGACTCAATAGGGA
    (((((.((((((((((((((......(((((((...((((.((.(((......).)).)).)))).)))))))...))))).))).)))).)).))))))
    ********************************************************************TGAAGGTCACAGAACAGGCAGACTCAA******
    ....................................................................TGAAGGTCACAGAACAGGCAGACTCAA......   7 P14-1 +/-
    ....................................................................TGAAGGTCACAGAACAGGCAGACTCAAT.....   7 P14-1 +/-
    .....................................................................................................   14 P14-1 +/-
    -----------------------------------------------------------------------------------------------------
```

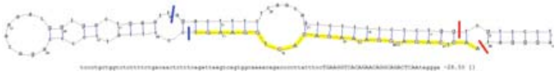

high confidence

```
115 CCTCCATCAATGGCCTTGGCCTTCTTCGTAGTACATACTTTTCCACTCAGGATGACGTGGACCTGTATACGGATTCAGAACCCAGGGCCACCTACCGGAGG
    (((((.....((((((((..((((((((.((((......((((((.....)).)))))..))))))))))....))))).)))))))))......)))))
    ****************TGGCCTTCTTCGTAGTACATACTTTTCC*********************************************************
    ................TGGCCTTCTTCGTAGTACATACTTTTCC.........................................................   5 P10-1 +/-
    .....................................................................................................   5 P10-1 +/-
    -----------------------------------------------------------------------------------------------------
```

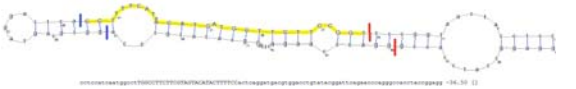

high confidence

```
116 GCATATATCCTTTAGTAGACTCTAAATCTTTTCTGGTGCAGAGCAGATTGGAGCCTTCTGAATTAAAAGAGAGACTGGGTTTAATTCAAGAGGATGTTGC
    (((.(((((((((..(((((((....((((((.((((.((((((.........))..)))).)))).))))))....))))))).....)))))))))))
    **********************************************************TGAATTAAAAGAGAGACTGGGTTTAA****************
    ..........................................................TGAATTAAAAGAGAGACTGGGTTTAA................   5 P10-1 +/-
    ....................................................................................................   5 P10-1 +/-
    ----------------------------------------------------------------------------------------------------
    ..........................................................TGAATTAAAAGAGAGACTGGGTTTAA................   7 P10-2 +/-
    ....................................................................................................   7 P10-2 +/-
    ----------------------------------------------------------------------------------------------------
```

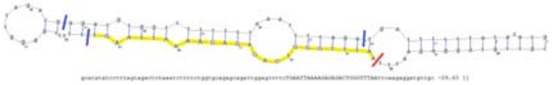

high confidence

117
```
GGGTTCCCACGCTTCTCTGAGAATCGAGTAGAGACAAGTTGATTGTGGTCTGCATGCTCTCTCCTTTGCCCGTCCTCGGAAACCTGCCTGTCTGGGACCT
((((((((.((...((((((...((.(((((((((.((.(((....))).)).)).)))))).....)).))..)))))).....)).))...)))))))
***************TCTGAGAATCGAGTAGAGACAAGTTG***********************************************************
...............TCTGAGAATCGAGTAGAGACAAGTTG...........................................................
....................................................................................................
---------------------------------------------------------------------------------------------------------
```
7 P10-1 +/-
7 P10-1 +/-

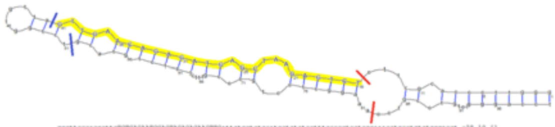

high confidence

118
```
ATAGTCGTCGTCGTCTTCTGGGATGGACAGGAGTGGGCACATTAGAATGATTTCCTGGTGGGTAGTTTCGGGTTTGTATCCAGAAGAGAAAATGGGTAT
(((.((((..((.((((((((((..(((..(((...((.((((((.........)))))).))...)))..)))..).)))))))))))..)))).)))
*TAGTCGTCGTCGTCTTCTGGGATGGA************************************************************************
.TAGTCGTCGTCGTCTTCTGGGATGGA........................................................................
.TAGTCGTCGTCGTCTTCTGGGATGG.........................................................................
...................................................................................................
---------------------------------------------------------------------------------------------------------
```
25 P14-1 +/-
7 P14-1 +/-
32 P14-1 +/-

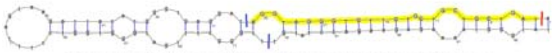

non-candidate

119
```
CTGTTGTCCTGAGATCCATCTTGTTTGAATACGGGCATCTTCTGACACAGAGAAGAGGTAATAATGTCAGTCACAGTTACAGGGGATTAAGGATGCAG
((((.(((((..(((((..(((((.(((..(((.((.((((((.......)))))).))....)))...))).....))))))))))).)))))))))
******************TCTTGTTTGAATACGGGCATCTTCTG******************************************************
..................TCTTGTTTGAATACGGGCATCTTCTG......................................................
..................................................................................................
---------------------------------------------------------------------------------------------------------
..................TCTTGTTTGAATACGGGCATCTTCTG......................................................
..................................................................................................
---------------------------------------------------------------------------------------------------------
..................TCTTGTTTGAATACGGGCATCTTCTG......................................................
..................................................................................................
---------------------------------------------------------------------------------------------------------
```
5 P10-1 +/-
5 P10-1 +/-
10 P10-2 +/-
10 P10-2 +/-
5 P14-1 +/-
5 P14-1 +/-

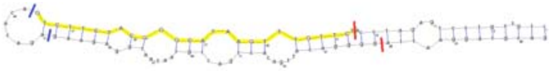

high confidence

120
```
CGATTCTTGTTTGCGTGGGGAGGAGGGGAGGGGATGGGCCAAGTTCCCTATGCCTGGAACGCCCTTCCCCCCCTTCTTCACCTGGCGAACTCCTACTCG
(((.....((((((..((((((((((.(((((.((((...(((((.........))))))))).)))))))))))))).))..))))))......)))
**********************************************************ACGCCCTTCCCCCCCTTCTTCA*******************
..........................................................ACGCCCTTCCCCCCCTTCTTCA...................
..........................................................ACGCCCTTCCCCCCCTTCTTC....................
...................................................................................................
---------------------------------------------------------------------------------------------------------
..........................................................ACGCCCTTCCCCCCCTTCTTC....................
...................................................................................................
---------------------------------------------------------------------------------------------------------
..........................................................ACGCCCTTCCCCCCCTTCTTCA...................
...................................................................................................
---------------------------------------------------------------------------------------------------------
..........................................................ACGCCCTTCCCCCCCTTCTTCA...................
...................................................................................................
---------------------------------------------------------------------------------------------------------
..........................................................ACGCCCTTCCCCCCCTTCTTC....................
...................................................................................................
---------------------------------------------------------------------------------------------------------
```
13 P7-3 -/-
8 P7-3 -/-
21 P7-3 -/-
5 P7-2 +/-
5 P7-2 +/-
5 P10-2 +/-
5 P10-2 +/-
7 P10-3 -/-
7 P10-3 -/-
5 P10-4 -/-
5 P10-4 -/-

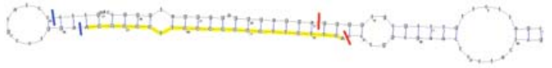

non-candidate

121
```
AAGCTATTGCATTCCATTTTTTTGAGGCTCATTTCTTCAGCTTTAAATTGAGCTGAAGAGATTGCCTGCCTCCTTGTGGCCATTATGCAACATTGCTT
((((..((((((.((((......(((((.((((((((((((((......)))))))))))).))...)))))...)))).....))))))....))))
************************************************TGAGCTGAAGAGATTGCCTGCCTCCTT***********************
................................................TGAGCTGAAGAGATTGCCTGCCTCCTT.......................
..................................................................................................
---------------------------------------------------------------------------------------------------------
................................................TGAGCTGAAGAGATTGCCTGCCTCCTT.......................
..................................................................................................
---------------------------------------------------------------------------------------------------------
```
7 P7-1 +/-
7 P7-1 +/-
7 P10-2 +/-
7 P10-2 +/-

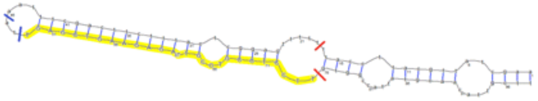

high confidence

122
```
GGAAATACAGAAACTGTGGCTCATAGTGCCAGGTTCCAGAAGATTCTATGGTTGCCCATGGTATCTGAGAGCTAGAATGACCCATGTCTGTGTTTGC
(.((((((((....((((.((((....(..((((((((.....((((((....))))))..)))).))))).).)))).)))).))))))))).)
**********************************************************TGGTATCTGAGAGCTAGAATGACCCATG***********
..........................................................TGGTATCTGAGAGCTAGAATGACCCATG...........
..........................................................TGGTATCTGAGAGCTAGAATGACCCAT............
.................................................................................................
---------------------------------------------------------------------------------------------------------
```
7 P7-1 +/-
6 P7-1 +/-
13 P7-1 +/-

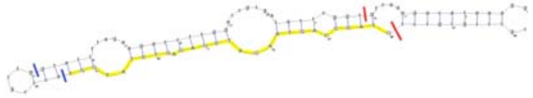

high confidence

123
```
GGGATGGTCCTAACACTTGGTCTACTCCTAAGTCTTATGCATAGAGTTCCAGTTTTCCAGCTTAGGACTCAGGAGTGCTTGGAGTGGTGGTTGTTCC
((((..(.((...(((((.(..(((((((.(((((((.((...(((........)))..)).))))))).)))))))..).)))))..)))..))))
**************************************************************TAGGACTCAGGAGTGCTTGGAGTGGT*********
..............................................................TAGGACTCAGGAGTGCTTGGAGTGGT.........
.................................................................................................
---------------------------------------------------------------------------------------------------------
..............................................................TAGGACTCAGGAGTGCTTGGAGTGGT.........
.............................................................TTAGGACTCAGGAGTGCTTGGAGTGG..........
.................................................................................................
---------------------------------------------------------------------------------------------------------
..............................................................TAGGACTCAGGAGTGCTTGGAGTGGT.........
.................................................................................................
---------------------------------------------------------------------------------------------------------
```
16 P10-1 +/-
16 P10-1 +/-
6 P10-2 +/-
6 P10-2 +/-
12 P10-2 +/-
5 P14-1 +/-
5 P14-1 +/-

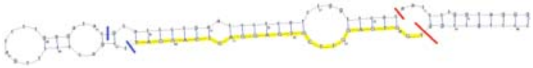

potential

124
```
GTTGTGTGACAGGGCAGTTTAGGATCCCCGGAAGTGCAAAGAAGGCCGCAAGAACTTCGTTTTCGCATCCAGAGGCCTGACTCTCCCTCCGACCAAC
((((.((...((((.((.(((((..(((.(((.(((.(((((((..(.....)..)))).))).))).))).).)))))))..))))))...))))))
*******************TAGGATCCCCGGAAGTGCAAAGAAGGC**************************************************
...................TAGGATCCCCGGAAGTGCAAAGAAGGC..................................................
...................TAGGATCCCCGGAAGTGCAAAGAAGGCC.................................................
................................................................................................
---------------------------------------------------------------------------------------------------------
...................TAGGATCCCCGGAAGTGCAAAGAAGGC..................................................
...................TAGGATCCCCGGAAGTGCAAAGAAGGCC.................................................
................................................................................................
---------------------------------------------------------------------------------------------------------
...................TAGGATCCCCGGAAGTGCAAAGAAGGC..................................................
...................TAGGATCCCCGGAAGTGCAAAGAAGGCC.................................................
................................................................................................
---------------------------------------------------------------------------------------------------------
```
8 P7-1 +/-
7 P7-1 +/-
15 P7-1 +/-
16 P10-1 +/-
10 P10-1 +/-
26 P10-1 +/-
13 P10-2 +/-
5 P10-2 +/-
18 P10-2 +/-

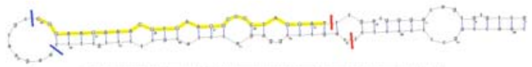

high confidence

125
```
GGCTGAAAAGGCTAGACTCCTGTGAGGTCCTTGCTGTGGAGGGCCCTCCTCAAGGGTCCCAGTTTGAGGAACTCAGCAGATGTTTGTGGGTCAGCC
(((((....((.((((..(((((((.(((((((((....(((((((.....))))))))))))..))))).)))).)))..))))))...))))))
**************************************************************TTTGAGGAACTCAGCAGATGTTTGTG********
..............................................................TTTGAGGAACTCAGCAGATGTTTGTG........
................................................................................................
---------------------------------------------------------------------------------------------------------
```
6 P10-1 +/-
6 P10-1 +/-

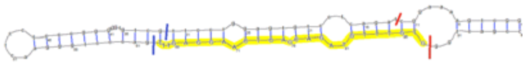

non-candidate

126
```
GGAAGGTGGGTGGGTTTGTGTTTCCGTGTTGGCAGATCTGGAGTAACGTGAGCTCTGGCAATAATGGCAGGCAGGCAGGTCACACAGAGGCCTTCT
((((((..((((..((((((((((((((((.((((.((.(.......).)).)))).)))).)))).))))..))))..).)))....)))))))
******************TGTTTCCGTGTTGGCAGATCTGGAG*****************************************************
................TGTGTTTCCGTGTTGGCAGATCTGGAG....................................................
................TGTGTTTCCGTGTTGGCAGATCTGGA.....................................................
................................................................................................
---------------------------------------------------------------------------------------------------------
..................TGTTTCCGTGTTGGCAGATCTGGAG.....................................................
................TGTGTTTCCGTGTTGGCAGATCTGGA.....................................................
................................................................................................
---------------------------------------------------------------------------------------------------------
```
8 P7-1 +/-
7 P7-1 +/-
15 P7-1 +/-
8 P10-1 +/-
5 P10-1 +/-
13 P10-1 +/-

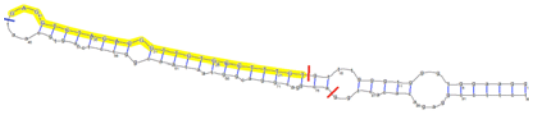

potential

```
127 CCATAGTCCCCTTGGACCTAGTTGACCTTGCAGGGCCGAGGGCAAAATACTGTCCCTCTTCTCCTTGCCCACGTTAATGTAGCCTCCAGGTATGG
    ((((.....((((((.((((((((...((((((..((((((((......)).)))))....))))))....)))))).))).)))))))))))
    ***********TTGGACCTAGTTGACCTTGCAGGGCC**********************************************************
    ...........TTGGACCTAGTTGACCTTGCAGGGCC..........................................................
    ...............................................................................................
    --------------------------------------------------------------------------------------------------------------
```

5 P14-1 +/-
5 P14-1 +/-

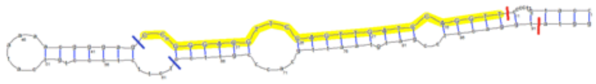

non-candidate

```
128 GGTCTCAGGGCTCAGTTCAGTTTGTCTCTGGAGGAAGGGCAGTGTGGGTTTTGGCCTCTGTGGGCTCTAAAGAAAGGGTGACTGGGTCCTGGGCC
    ((((.((((((((((((.((.(((.(((.(((((.....((((...((((....))))))))...))))).))).))).))))))))))))))))
    ***************************************************TGGCCTCTGTGGGCTCTAAAGAAAGGGT****************
    ...................................................TGGCCTCTGTGGGCTCTAAAGAAAGGGT................
    ...............................................................................................
    --------------------------------------------------------------------------------------------------------------
```

5 P10-1 +/-
5 P10-1 +/-

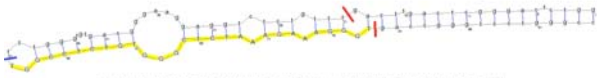

high confidence

```
129 GGGTGAGAGTAGGGGATGATGAGGACTGTTTTTTTTTCTTTTAAATTTTGCTTTGAGAGGCAGGCTTCAGATCTTTTGGTTCTCTTCTCTAACCC
    ((((.((((.(((((((.(.((((.(((....(((.(((((((((......)))))))))).)))...))).)))).).))))))).))))).))))
    *****************************************************TGAGAGGCAGGCTTCAGATCTTTTGGT***************
    .....................................................TGAGAGGCAGGCTTCAGATCTTTTGGTT..............
    .....................................................TGAGAGGCAGGCTTCAGATCTTTTGG................
    ...............................................................................................
    --------------------------------------------------------------------------------------------------------------
    .....................................................TGAGAGGCAGGCTTCAGATCTTTTGGTT..............
    ...............................................................................................
    --------------------------------------------------------------------------------------------------------------
    .....................................................TGAGAGGCAGGCTTCAGATCTTTTGG................
    ...............................................................................................
    --------------------------------------------------------------------------------------------------------------
    .....................................................TGAGAGGCAGGCTTCAGATCTTTTGGT...............
    ...............................................................................................
    --------------------------------------------------------------------------------------------------------------
```

8 P7-1 +/-
6 P7-1 +/-
14 P7-1 +/-

7 P10-1 +/-
7 P10-1 +/-

6 P10-2 +/-
6 P10-2 +/-

9 P14-1 +/-
9 P14-1 +/-

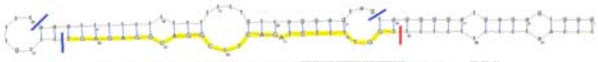

high confidence

```
130 GGCACTGTGAGATTTGTTCATGATCTTCTGACTTACCCGTTGCAAAGGAAGCCAGATGCCGTATCAGAAAGAGTCAAGGCAGTCTCGCCAGTGCT
    ((((((((((((((.(((..(((((((((((..(((.((((((.......))..))))..))))))).)))).))).)))))))))).)))))))
    *******TGAGATTTGTTCATGATCTTCTGACTT**************************************************************
    .......TGAGATTTGTTCATGATCTTCTGACTT..............................................................
    .......TGAGATTTGTTCATGATCTTCTGACTTACC...........................................................
    .......TGAGATTTGTTCATGATCTTCTGACTTA.............................................................
    ...............................................................................................
    --------------------------------------------------------------------------------------------------------------
```

7 P14-1 +/-
7 P14-1 +/-
5 P14-1 +/-
19 P14-1 +/-

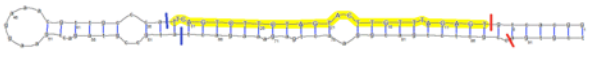

high confidence plus star

```
131 AGTCTACATAGGGAGCTTGAGGACAGCCTAGGCTACATTGTGAGACCCTGTCTCAACAACAAAGATATAGACTGTTTTGTGCTCTCCTTAAGACT
    (((((....(((((((...(((((((.(((..((...(((((((((...))))).))))...))...))).))))))))..)))).)))..)))))
    *************************************************************TAGACTGTTTTGTGCTCTCCTTAAGACT*
    ....TACATAGGGAGCTTGAGGACAGCCTA.................................................................
    .............................................................TAGACTGTTTTGTGCTCTCCTTAAGACT.
    ...............................................................................................
    --------------------------------------------------------------------------------------------------------------
    .............................................................TAGACTGTTTTGTGCTCTCCTTAAGACT.
    ...............................................................................................
    --------------------------------------------------------------------------------------------------------------
    .............................................................TAGACTGTTTTGTGCTCTCCTTAAGACT.
    ...............................................................................................
    --------------------------------------------------------------------------------------------------------------
    ....TACATAGGGAGCTTGAGGACAGCCTA.................................................................
    .............................................................TAGACTGTTTTGTGCTCTCCTTAAGACT.
    ...............................................................................................
    --------------------------------------------------------------------------------------------------------------
```

5 P7-1 +/-
9 P7-1 +/-
14 P7-1 +/-

7 P7-2 +/-
7 P7-2 +/-

12 P10-1 +/-
12 P10-1 +/-

5 P10-2 +/-
8 P10-2 +/-
13 P10-2 +/-

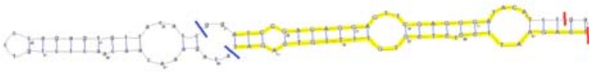

potential

```
132 CCTCCCGATCCTGTACTGACTGTAATGCATATTAGCCAGGTGATGCACTTTAGCTACCCTGGACAATGCTGTCAAGTGTGCTGGGAAGAAAGG
    (((..(..(((((((((((((......((((.....(((((.(..((......))..))))))...)))).))))...))))).)))).)..)))
    ........TCCTGTACTGACTGTAATGCATATTAGCC..........................................................
    ........TCCTGTACTGACTGTAATGCATATTAGCCA.........................................................
    ........TCCTGTACTGACTGTAATGCATATTAGC...........................................................
    ...............................................................................................
    --------------------------------------------------------------------------------------------------------------
    ********TCCTGTACTGACTGTAATGCATATTAGCC**********************************************************
    ........TCCTGTACTGACTGTAATGCATATTAGC...........................................................
    ...............................................................................................
    --------------------------------------------------------------------------------------------------------------
```

21 P7-1 +/-
13 P7-1 +/-
10 P7-1 +/-
44 P7-1 +/-

10 P10-1 +/-
10 P10-1 +/-

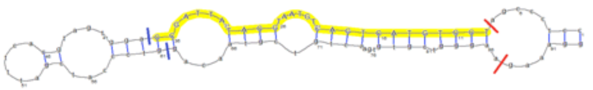

high confidence

```
133 CTTGTTGGTGGTTTTCTTAAATCTCTTTTATTTCTAGGATAAGGATGCTAAATTCCGCCTGATTCTGATAGAGAGCAGAATTCACCGATTGG
    ((.((((((((..((((....(((((.(((..((.(((....(((........))).)))))...))).)))))..)))).)))))))).))
    ****************************************************************TGATAGAGAGCAGAATTCACCGATTG**
    ................................................................TGATAGAGAGCAGAATTCACCGATTG..
    ...............................................................................................
    --------------------------------------------------------------------------------------------------------------
    ................................................................TGATAGAGAGCAGAATTCACCGATTG..
    ...............................................................................................
    --------------------------------------------------------------------------------------------------------------
    ................................................................TGATAGAGAGCAGAATTCACCGATTG..
    ...............................................................................................
    --------------------------------------------------------------------------------------------------------------
    ................................................................TGATAGAGAGCAGAATTCACCGATTG..
    ...............................................................................................
    --------------------------------------------------------------------------------------------------------------
```

8 P7-1 +/-
8 P7-1 +/-

17 P10-1 +/-
17 P10-1 +/-

16 P10-2 +/-
16 P10-2 +/-

8 P14-1 +/-
8 P14-1 +/-

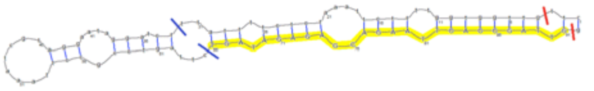

potential

```
134 TCTGGAACTCACCCCAGGTAGCAGTCTGACTGAGAGCATGGAGAAAACAAATGCTTTTAGACTGACTGACTATTAAACCGGGTGAACCCAGA
    ((((...((((((..((((((((((.(.((((((((((..........)))))))))).).))))).))))).....))))))..)))))
    **TGGAACTCACCCCAGGTAGCAGTCTGA*******************************************************************
    ..TGGAACTCACCCCAGGTAGCAGTCTGA...................................................................
    ...............................................................................................
    --------------------------------------------------------------------------------------------------------------
```

8 P10-2 +/-
8 P10-2 +/-

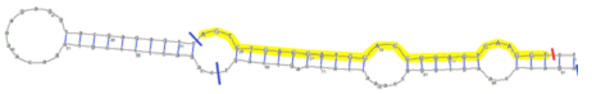

high confidence

```
135 TACTGAGCCACACTCCCTGTCCCTGAAAACTATTTTCTAATCATGACCTTTTAAATTTTAGAATGTAAGGGACTGGAGAGATGGCTTAGTG
    ((((((((((.((((..((((((....((....((((((.................)))))).)).)))))).)))).).))))))))))
    ****************************************************************TGTAAGGGACTGGAGAGATGGCT******
    ................................................................TGTAAGGGACTGGAGAGATGGC.......
    ...............................................................................................
    --------------------------------------------------------------------------------------------------------------
    ................................................................TGTAAGGGACTGGAGAGATGGCT......
    ...............................................................................................
    --------------------------------------------------------------------------------------------------------------
    ................................................................TGTAAGGGACTGGAGAGATGGCT......
    ...............................................................................................
    --------------------------------------------------------------------------------------------------------------
```

6 P7-1 +/-
6 P7-1 +/-

8 P10-1 +/-
8 P10-1 +/-

6 P10-2 +/-
6 P10-2 +/-

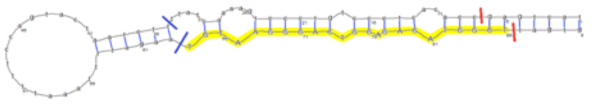

potential

```
136 TGCAGAATGAACATGAGCTGGAGCAGTGAGGTCTCGTAACACTAGAGAGAACCGTCCCTCCTGCCCAACCCGGCTCCCTGTCGGCTCTGCA
    .(((((.(((....(((((((.((((.((((((((...........))))......))))))))).....)))))))....))).)..))))).
    *******TGAACATGAGCTGGAGCAGTGAGGTC***************************************************************
    .......TGAACATGAGCTGGAGCAGTGAGGTC...............................................................
    ...............................................................................................
    --------------------------------------------------------------------------------------------------------------
```

5 P10-1 +/-
5 P10-1 +/-

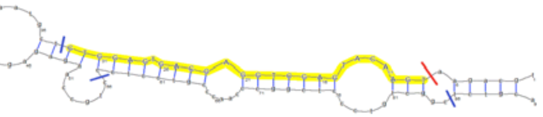

high confidence

137
```
GGGAATTAGATGTTACCACCACTGTTTTCAATGCGTCCCAGATATGACACCATGATCCTGAGATGGGAAAAGGAGGTAGCGTTAGGTCTCC
((..((..((((((((.((....((((....((((.(((.((((....))))...))).)))).))))))).)))))))))..))..))
**********************************************************TGAGATGGGAAAAGGAGGTAGCGTTA********
..........................................................TGAGATGGGAAAAGGAGGTAGCGTTA........
..........................................................TGAGATGGGAAAAGGAGGTAGCGTT.........
...........................................................................................
-------------------------------------------------------------------------------------------------------
```

6 P14-1 +/-
5 P14-1 +/-
11 P14-1 +/-

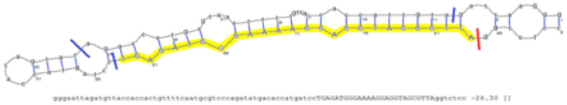

potential

138
```
GGTGGGCATGTTGATCGGCGTTCTTGGTTATGACAGACCTCCTGGGAGACAGGAAGGCTGTGGCTTCCAGGGAGTGGGTGGCAGGCTCGCT
(((((((.((((.(((.((.((((((((...(.((((.(((((((.....)))).))))))).)...))))))))).))))))).)))))))
********TGTTGATCGGCGTTCTTGGTTATG************************************************************
........TGTTGATCGGCGTTCTTGGTTATG............................................................
........TGTTGATCGGCGTTCTTGGTTATGA...........................................................
...........................................................................................
-------------------------------------------------------------------------------------------------------
........TGTTGATCGGCGTTCTTGGTTATG............................................................
...........................................................................................
-------------------------------------------------------------------------------------------------------
........TGTTGATCGGCGTTCTTGGTTATG............................................................
...........................................................................................
-------------------------------------------------------------------------------------------------------
```

13 P7-1 +/-
5 P7-1 +/-
18 P7-1 +/-

6 P10-1 +/-
6 P10-1 +/-

6 P10-2 +/-
6 P10-2 +/-

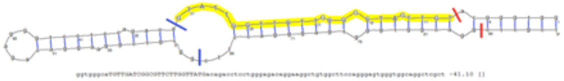

high confidence

139
```
GGAGGCCAGCCCTAATGGGTAGACTGAGCCTGGCTGACTTGAAACCTCCCCTGGGCTTTGTCTGCCTCCACAGAATGGGGTTGGCCCTCC
((((((((((((((.(((((((((.(((((..(..((.........))..)..))))).))))))))....))...)))))))))).)))))
***************TGGGTAGACTGAGCCTGGCTGA*****************************************************
...............TGGGTAGACTGAGCCTGGCTGA.....................................................
..........................................................................................
-------------------------------------------------------------------------------------------------------
...............TGGGTAGACTGAGCCTGGCTGA.....................................................
..........................................................................................
-------------------------------------------------------------------------------------------------------
```

7 P7-3 -/-
7 P7-3 -/-

10 P10-3 -/-
10 P10-3 -/-

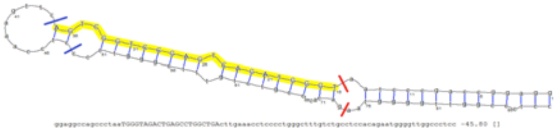

high confidence

140
```
GTTCTCTGTGCTGTTTCTAGAAGTAGGGGCTGTGGCTGAGAACAGGCCTCTGAGAGCCTGCTGTTTACTCTGGACTGCAGCATAGACCAC
....(((((((((.((((((((((((..(((.((((.......)))).)..))..))))))).....))))))..))))))))))....
*************************************************************TGTTTACTCTGGACTGCAGCATAGACC***
.............................................................TGTTTACTCTGGACTGCAGCATAGACC...
..........................................................................................
-------------------------------------------------------------------------------------------------------
```

6 P7-1 +/-
6 P7-1 +/-

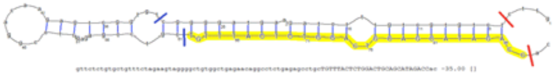

high confidence

141
```
GGGAACATCTGGTCTCCAGCCTTTTAGGTGCATGATAGACCTGATGTGGTAATGCATGCCTTTAATTCTAGGCTGAGGCAGGTGGATCTC
((((.((((((.((((.(((((.(((((.(((((....(((......)))....))))).)))))....))))))))))))))))..))))
**************************************************************TAATTCTAGGCTGAGGCAGGTGGATC***
..............................................................TAATTCTAGGCTGAGGCAGGTGGATC...
..........................................................................................
-------------------------------------------------------------------------------------------------------
```

8 P7-1 +/-
8 P7-1 +/-

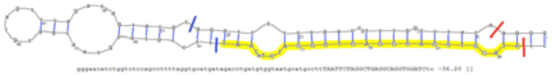

potential

142
```
TCTGCATGGTCAGATGTTTTAGGTTGGGAAGAAAGCTTAAGCTCAGAAAGCTTTAAGCCTGTGACGGGCCTGAGGCTCCTGCTGGGTAGG
.((((..((.(((..((((((((((.(..(.(..((((((((((.....))).)))))).).)..).)))))))))))..)))))..)))).
******TGGTCAGATGTTTTAGGTTGGGAAGA**********************************************************
......TGGTCAGATGTTTTAGGTTGGGAAGA..........................................................
..........................................................................................
-------------------------------------------------------------------------------------------------------
```

5 P10-1 +/-
5 P10-1 +/-

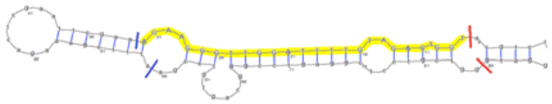

high confidence plus star

143
```
GGAATAGATGTGATAACGCTCCCAGAGGCTGGCTTCGGTGGCAGGCATTTTGTTAGTCTCCTGGACCTGCTGTTTTCAGAGCTAGGACC
((..(((.(.((((.(((((..((((((((((((...((((.....))))..)))))))))).))))....)).))).))).).)))...))
****TAGATGTGATAACGCTCCCAGAGGCTGG*********************************************************
....TAGATGTGATAACGCTCCCAGAGGCTGG.........................................................
....TAGATGTGATAACGCTCCCAGAGGCTGGC........................................................
.........................................................................................
-------------------------------------------------------------------------------------------------------
....................................................TTAGTCTCCTGGACCTGCTGTTTTCA...........
.........................................................................................
-------------------------------------------------------------------------------------------------------
```

20 P7-1 +/-
6 P7-1 +/-
26 P7-1 +/-

5 P10-2 +/-
5 P10-2 +/-

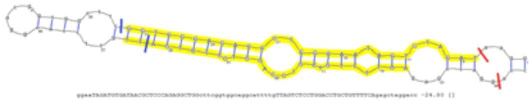

potential

144
```
GTCATAATAAACAAGGGATGCCTTCGAAGATGCAGTCTTCCCAGACCCGGGTCTGTAGTAGGATCTTTGAGTTTCTTGCGGTTTATGGC
(((((((....((((..((....((((((((.(...((...(((((....))))).))..).))))))))))..))))....)))))))
**********************************************************TAGGATCTTTGAGTTTCTTGCGGTTT******
..........................................................TAGGATCTTTGAGTTTCTTGCGGTTT......
..........................................................TAGGATCTTTGAGTTTCTTGCGGTT.......
.......................................................TAGTAGGATCTTTGAGTTTCTTGCGGTT.......
.........................................................................................
-------------------------------------------------------------------------------------------------------
..........................................................TAGGATCTTTGAGTTTCTTGCGGTTT......
.......................................................TAGTAGGATCTTTGAGTTTCTTGCGGTT.......
..........................................................TAGGATCTTTGAGTTTCTTGCGGTT.......
.........................................................................................
-------------------------------------------------------------------------------------------------------
..........................................................TAGGATCTTTGAGTTTCTTGCGGTTT......
.......................................................TAGTAGGATCTTTGAGTTTCTTGCGGTT.......
..........................................................TAGGATCTTTGAGTTTCTTGCGGTT.......
.......................................................TAGTAGGATCTTTGAGTTTCTTGCGGT........
.........................................................................................
-------------------------------------------------------------------------------------------------------
.......................................................TAGTAGGATCTTTGAGTTTCTTGCGGTT.......
..........................................................TAGGATCTTTGAGTTTCTTGCGGTTT......
.........................................................................................
-------------------------------------------------------------------------------------------------------
```

36 P7-1 +/-
24 P7-1 +/-
16 P7-1 +/-
76 P7-1 +/-

23 P10-1 +/-
18 P10-1 +/-
15 P10-1 +/-
56 P10-1 +/-

23 P10-2 +/-
20 P10-2 +/-
16 P10-2 +/-
6 P10-2 +/-
65 P10-2 +/-

9 P14-1 +/-
7 P14-1 +/-
16 P14-1 +/-

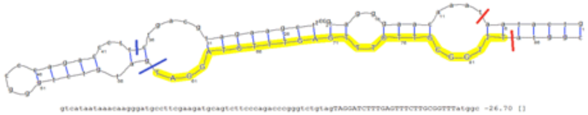

high confidence

145
```
GCATCACAGGGTGTTGTGAGGATAATGCATGTATACAGTAATTGCTCAATAAAGGTGGCTGCTGTTGTTATAAATCCCTGCCTGGTGC
((((((((((..((((((.((((..(((.((.(((..(.(((....))).)..))))))))))))).))))))..)))))..))))))
****************TGAGGATAATGCATGTATACAGTAATT*********************************************
................TGAGGATAATGCATGTATACAGTAATT.............................................
..............TGTGAGGATAATGCATGTATACAGTAATT.............................................
..............TGTGAGGATAATGCATGTATACAGTAAT..............................................
........................................................................................
-------------------------------------------------------------------------------------------------------
```

9 P14-1 +/-
7 P14-1 +/-
5 P14-1 +/-
21 P14-1 +/-

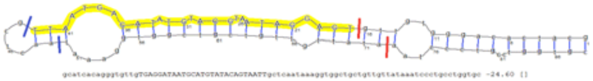

```
high confidence   146 TCTGTTTGGTTTGGATTTGTGAAAGTCTCACTGTGTAGTTCTGGCTAGCCTTGAGCTCACTTTGTAGAACAGGCTAGACTGAAACAGG
                      .((((((((((((.(((((.....(((((..(((.(((((.((....))..))))))))..)).))))))))))))))).))))))).
                      ********************************************************TCACTTTGTAGAACAGGCT*************
                      ........................................................TCACTTTGTAGAACAGGCT.............     25  P10-1 +/-
                      ........................................................TCACTTTGTAGAACAGGC..............     15  P10-1 +/-
                      ........................................................TCACTTTGTAGAACAGGCTAGA.........     10  P10-1 +/-
                      ........................................................TCACTTTGTAGAACAGGCTAGAC........      7  P10-1 +/-
                      .......................................................CTCACTTTGTAGAACAGGCT.............      6  P10-1 +/-
                      ........................................................TCACTTTGTAGAACAGGCTAG..........      5  P10-1 +/-
                      ........................................................................................     68  P10-1 +/-
                      ----------------------------------------------------------------------------------------------------
                      ........................................................TCACTTTGTAGAACAGGCT.............     71  P10-2 +/-
                      ........................................................TCACTTTGTAGAACAGGC..............     38  P10-2 +/-
                      ........................................................TCACTTTGTAGAACAGGCTAG..........     20  P10-2 +/-
                      ........................................................TCACTTTGTAGAACAGGCTA...........     14  P10-2 +/-
                      ........................................................TCACTTTGTAGAACAGGCTAGA.........     10  P10-2 +/-
                      ........................................................TCACTTTGTAGAACAGGCTAGAC........     10  P10-2 +/-
                      .......................................................CTCACTTTGTAGAACAGGCT.............      5  P10-2 +/-
                      ........................................................................................    168  P10-2 +/-
                      ----------------------------------------------------------------------------------------------------
                      ........................................................TCACTTTGTAGAACAGGCT.............     34  P14-1 +/-
                      ........................................................TCACTTTGTAGAACAGGC..............     20  P14-1 +/-
                      ........................................................TCACTTTGTAGAACAGGCTAG..........      9  P14-1 +/-
                      ........................................................TCACTTTGTAGAACAGGCTA...........      6  P14-1 +/-
                      ........................................................TCACTTTGTAGAACAGGCTAGA.........      5  P14-1 +/-
                      ........................................................................................     74  P14-1 +/-
                      ----------------------------------------------------------------------------------------------------
```

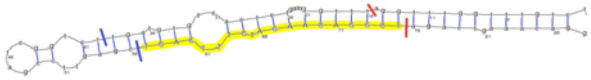


```
non-candidate     147 CTGCCATCCTACACTTAGATTGTAGGCCCATTGGAAGGGTTAGCGTCTTGACCCCAATGAGCCTACAGTCTAAGTGTGGGATGGCGG
                      (((((((((((((((((((((((((.((((((...((((((....)))))))))))).)))))))))))))))))))))))))))
                      ***************TAGATTGTAGGCCCATTGGAAGG*************************************************
                      ...............TAGATTGTAGGCCCATTGGAAGG.................................................      7  P7-3 -/-
                      .......................................................................................      7  P7-3 -/-
                      ----------------------------------------------------------------------------------------------------
```

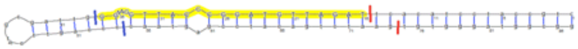


```
high confidence   148 CACAATTTAACTGACAACTGAGTTGAAGGCAAAGGTAGTAATGTCTGACTTAGCTTTGCCTTCAGCTTAGTCGTCAGTTAAATTGTG
                      ((((((((((((((.((((((((((((((((((.((((........)).)).)))))))))))))))))).)))))))))))))))
                      ***************AACTGAGTTGAAGGCAAAGGT***************************************************
                      ...............AACTGAGTTGAAGGCAAAGGT...................................................      6  P7-1 +/-
                      .......................................................................................      6  P7-1 +/-
                      ----------------------------------------------------------------------------------------------------
                      ...............AACTGAGTTGAAGGCAAAGGT...................................................      5  P7-4 -/-
                      .......................................................................................      5  P7-4 -/-
                      ----------------------------------------------------------------------------------------------------
```

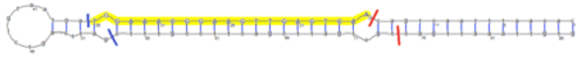


```
high confidence   149 GAGCCCTGTGCACAAGCTTTCTCATCTGTGACACTCGAGATGGTTTCAGGGAGTGGACACTGGAGAGAGAGCTTTTGCAAGTCGCTC
                      ((((....((((.((((((((((.((((((.(((((..((.....))...)))))..))).))))))))))))).)))).....))))
                      *****************************************************TGGACACTGGAGAGAGAGCTTTT***********
                      .....................................................TGGACACTGGAGAGAGAGCTTTT...........     54  P7-1 +/-
                      .....................................................TGGACACTGGAGAGAGAGCTTT............     28  P7-1 +/-
                      .....................................................TGGACACTGGAGAGAGAGCTT.............      8  P7-1 +/-
                      .......................................................................................     90  P7-1 +/-
                      ----------------------------------------------------------------------------------------------------
                      .....................................................TGGACACTGGAGAGAGAGCTTTT...........     31  P7-2 +/-
                      .....................................................TGGACACTGGAGAGAGAGCTTT............     16  P7-2 +/-
                      .......................................................................................     47  P7-2 +/-
                      ----------------------------------------------------------------------------------------------------
                      .....................................................TGGACACTGGAGAGAGAGCTTTT...........     81  P7-3 -/-
                      .....................................................TGGACACTGGAGAGAGAGCTTT............     49  P7-3 -/-
                      .....................................................TGGACACTGGAGAGAGAGCTT.............     14  P7-3 -/-
                      .......................................................................................    144  P7-3 -/-
                      ----------------------------------------------------------------------------------------------------
                      .....................................................TGGACACTGGAGAGAGAGCTTTT...........    102  P7-4 -/-
                      .....................................................TGGACACTGGAGAGAGAGCTTT............     76  P7-4 -/-
                      .....................................................TGGACACTGGAGAGAGAGCTT.............     11  P7-4 -/-
                      .......................................................................................    189  P7-4 -/-
                      ----------------------------------------------------------------------------------------------------
                      .....................................................TGGACACTGGAGAGAGAGCTTT............     20  P10-1 +/-
                      .....................................................TGGACACTGGAGAGAGAGCTTTT...........     19  P10-1 +/-
                      .......................................................................................     39  P10-1 +/-
                      ----------------------------------------------------------------------------------------------------
                      .....................................................TGGACACTGGAGAGAGAGCTTTT...........     17  P10-2 +/-
                      .....................................................TGGACACTGGAGAGAGAGCTTT............      8  P10-2 +/-
                      .......................................................................................     25  P10-2 +/-
                      ----------------------------------------------------------------------------------------------------
                      .....................................................TGGACACTGGAGAGAGAGCTTTT...........     59  P10-3 -/-
                      .....................................................TGGACACTGGAGAGAGAGCTTT............     45  P10-3 -/-
                      .....................................................TGGACACTGGAGAGAGAGCTT.............     11  P10-3 -/-
                      .......................................................................................    115  P10-3 -/-
                      ----------------------------------------------------------------------------------------------------
                      .....................................................TGGACACTGGAGAGAGAGCTTTT...........     52  P10-4 -/-
                      .....................................................TGGACACTGGAGAGAGAGCTTT............     41  P10-4 -/-
                      .....................................................TGGACACTGGAGAGAGAGCTT.............      6  P10-4 -/-
                      .......................................................................................     99  P10-4 -/-
                      ----------------------------------------------------------------------------------------------------
                      .....................................................TGGACACTGGAGAGAGAGCTTTT...........     14  P14-1 +/-
                      .....................................................TGGACACTGGAGAGAGAGCTTT............      8  P14-1 +/-
                      .......................................................................................     22  P14-1 +/-
                      ----------------------------------------------------------------------------------------------------
                      .....................................................TGGACACTGGAGAGAGAGCTTTT...........     22  P14-3 -/-
                      .....................................................TGGACACTGGAGAGAGAGCTTT............     14  P14-3 -/-
                      .......................................................................................     36  P14-3 -/-
                      ----------------------------------------------------------------------------------------------------
                      .....................................................TGGACACTGGAGAGAGAGCTTTT...........     29  P14-4 -/-
                      .....................................................TGGACACTGGAGAGAGAGCTTT............     20  P14-4 -/-
                      .....................................................TGGACACTGGAGAGAGAGCTT.............      6  P14-4 -/-
                      .......................................................................................     55  P14-4 -/-
                      ----------------------------------------------------------------------------------------------------
```

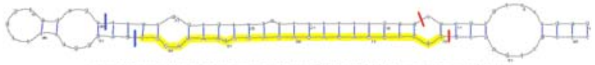


```
non-candidate     150 TGGCTGGCTGCTAGATTGTAAGCTCATGAGGGCAGAAACCTCATTCCTTTCTGTATTCTCATTGCTGAACACATAGCTGCTGCTCA
                      .(((.(((.((((...(((.(((..(((((((((((((.........)))))))..)))))).)))..)))..)))).)))))).. 
                      ***********TAGATTGTAAGCTCATGAGGGCAGAAACC**********************************************
                      ...........TAGATTGTAAGCTCATGAGGGCAGAAACC..............................................      6  P10-1 +/-
                      ......................................................................................      6  P10-1 +/-
                      ----------------------------------------------------------------------------------------------------
```

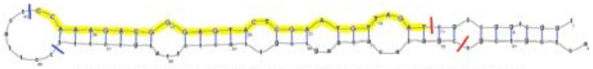


```
high confidence   151 GGGCCAGCCTATCTGGGTTCATGCAGTGTAGTCTCTTTCCTTCATTGGGCTCAGGGGACCTTTTGACTGTGTGCAAGGGCTGGGCT
                      ((.(((((((...((....(((((((...((((((((((......)))...)))))))......)))))))))).))))))).))
                      ***********TCTGGGTTCATGCAGTGTAGTCTCTTT************************************************
                      ...........TCTGGGTTCATGCAGTGTAGTCTCTTT................................................     13  P14-1 +/-
                      .............................................................TTTGACTGTGTGCAAGGGCTGG...      6  P14-1 +/-
                      ......................................................................................     19  P14-1 +/-
                      ----------------------------------------------------------------------------------------------------
```

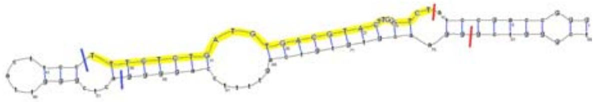


```
high confidence   152 TTGCCATCTAAATGTGCAACTCTTGGGAGAGCTTCCAGGGCAGATGCCTGGGCTCTGTGAGGAGTGGGGCTGCAGGAGTTGGGTAA
                      .((((..((...(((((.((((((.(.(((((..(((((.(....))))))))))).).))))))...)).)))..))...)))).
                      ******TCTAAATGTGCAACTCTTGGGAGAGCTT****************************************************
                      ......TCTAAATGTGCAACTCTTGGGAGAGCTT....................................................      9  P14-1 +/-
                      ........TAAATGTGCAACTCTTGGGAGAGCTTC...................................................      6  P14-1 +/-
                      ......................................................................................     15  P14-1 +/-
                      ----------------------------------------------------------------------------------------------------
```

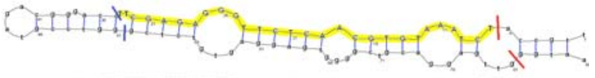

potential

```
153 AGCCCCAGACTGTGGCACTGGAAAAGATGAGGCTATGTAGCCAAAGCCCAAATGACGTCTGAGTGGCACAATCAGCCTGGGCGCT
    ((((((((.(((((.(((((((..(..((.((((.((....)).)))))))..)....))).)))).)))....)).))))).)))
    **********TGTGGCACTGGAAAAGATGAGGCTAT*************************************************
    ..........TGTGGCACTGGAAAAGATGAGGCTAT.................................................   6 P10-2 +/-
    .....................................................................................   6 P10-2 +/-
    ----------------------------------------------------------------------------------------------------
```

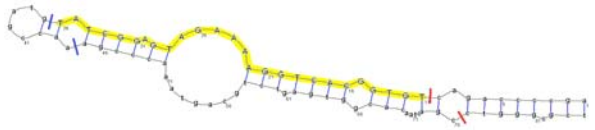

potential

```
154 ACGCTGTTCTGGCTGAACTATCTCAGAGACTTTTGGTCGTCTTTGGTCTTCTGTCCCCTGTGCCAGTTCAGGCTGAACTGGTGT
    ((((((((.(.(((((((..(.(((.(((....(..((....))..)....)))..))).)..))))))).).)))).)))))
    ********CTGGCTGAACTATCTCAGAGACTTT***************************************************
    ........CTGGCTGAACTATCTCAGAGACTTT...................................................   5 P14-1 +/-
    ....................................................................................   5 P14-1 +/-
    ----------------------------------------------------------------------------------------------------
```

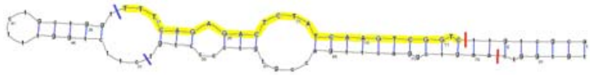

high confidence

```
155 TGGAGCAGAAGGGCAAAAGCTCGCTTGATCTTGATTTTCAGTACAAGTCCAGATCATGAAATCAGGGCCTTTTTCCTCTGTCCA
    .(((.((((.(((.(((.((((.(.((((((.(((((.......))))).)))))).)......)))).))).)))))))))).
    ******AGAAGGGCAAAAGCTCGCTTGATCTTGA**************************************************
    ......AGAAGGGCAAAAGCTCGCTTGATCTTGA..................................................   5 P7-2 +/-
    ....................................................................................   5 P7-2 +/-
    ----------------------------------------------------------------------------------------------------
```

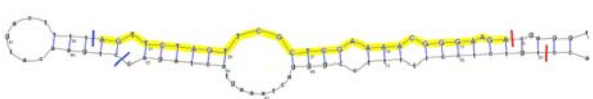

potential

```
156 TAGACAGGGTTTCTCTCTGTATCCCTGGCTGTTTTAGGACTTGCTCTATAGAGCAGGCTGGCCTCGAACTGAGAGATCCGTCTG
    (((((.(((((((((...((.((...((((......((.(((((((....)))))))))))))..)))).))))))))))))))
    **************************************************GAGCAGGCTGGCCTCGAACT**************
    ----------------------------------------------------------------------------------------------------
    ..................................................GAGCAGGCTGGCCTCGAACT..............   861 P14-1 +/-
    ...............................................TAGAGCAGGCTGGCCTCGAA.................   258 P14-1 +/-
    ..................................................GAGCAGGCTGGCCTCGAAC...............   225 P14-1 +/-
    ...............................................TAGAGCAGGCTGGCCTCGA..................   211 P14-1 +/-
    ...............................................TAGAGCAGGCTGGCCTCGAACT...............   210 P14-1 +/-
    ...............................................TAGAGCAGGCTGGCCTCGAAC................   125 P14-1 +/-
    ...................................................AGCAGGCTGGCCTCGAACT..............   68 P14-1 +/-
    ................................................AGAGCAGGCTGGCCTCGAACT...............   51 P14-1 +/-
    ..................................................GAGCAGGCTGGCCTCGAA................   39 P14-1 +/-
    ................................................AGAGCAGGCTGGCCTCGAAC................   18 P14-1 +/-
    ................................................AGAGCAGGCTGGCCTCGAA.................   15 P14-1 +/-
    ...............................................TAGAGCAGGCTGGCCTCG...................   15 P14-1 +/-
    ..................................................GAGCAGGCTGGCCTCGA.................   9 P14-1 +/-
    ...................................................AGCAGGCTGGCCTCGAAC...............   7 P14-1 +/-
    ...................................................AGCAGGCTGGCCTCGAA................   5 P14-1 +/-
    ..................................................GAGCAGGCTGGCCTCGAACTG.............   5 P14-1 +/-
    ....................................................................................   2122 P14-1 +/-
    ----------------------------------------------------------------------------------------------------
    ..................................................GAGCAGGCTGGCCTCGAACT..............   1211 P14-3 -/-
    ...............................................TAGAGCAGGCTGGCCTCGAA.................   309 P14-3 -/-
    ..................................................GAGCAGGCTGGCCTCGAAC...............   298 P14-3 -/-
    ...............................................TAGAGCAGGCTGGCCTCGA..................   276 P14-3 -/-
    ...............................................TAGAGCAGGCTGGCCTCGAAC................   190 P14-3 -/-
    ...............................................TAGAGCAGGCTGGCCTCGAACT...............   100 P14-3 -/-
    ..................................................GAGCAGGCTGGCCTCGAA................   57 P14-3 -/-
    ................................................AGAGCAGGCTGGCCTCGAACT...............   46 P14-3 -/-
    ...................................................AGCAGGCTGGCCTCGAACT..............   37 P14-3 -/-
    ................................................AGAGCAGGCTGGCCTCGAAC................   29 P14-3 -/-
    ................................................AGAGCAGGCTGGCCTCGAA.................   19 P14-3 -/-
    ...............................................TAGAGCAGGCTGGCCTCG...................   15 P14-3 -/-
    ................................................AGAGCAGGCTGGCCTCGA..................   11 P14-3 -/-
    ..................................................GAGCAGGCTGGCCTCGAANN..............   8 P14-3 -/-
    ...................................................AGCAGGCTGGCCTCGAAC...............   7 P14-3 -/-
    ..................................................GAGCAGGCTGGCCTCG..................   7 P14-3 -/-
    ...................................................AGCAGGCTGGCCTCGAA................   6 P14-3 -/-
    ..................................................GAGCAGGCTGGCCTCGAACTG.............   5 P14-3 -/-
    ....................................................GCAGGCTGGCCTCGAA................   5 P14-3 -/-
    ....................................................................................   2636 P14-3 -/-
```

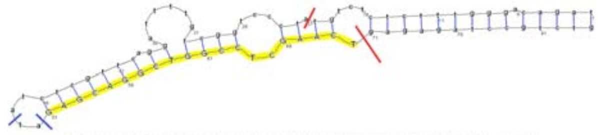

potential

```
157 TCGGCTCCTGCTTCTTGACCTGCTTGAGTTCCAGTCCTGACTTCCTTGGTGATGAACAGCAAGCAGTATGAAAGTATAAGCCGA
    ((((((..(((((.(((..(((((((.((((..((((..(.....)..).))))))))..)))))))..))))))))..))))))
    *********************************************TTGGTGATGAACAGCAAGCAGTATGAAAGT*********
    .............................................TTGGTGATGAACAGCAAGCAGTATGAAAGT.........   15 P7-1 +/-
    ..............................................TGGTGATGAACAGCAAGCAGTATGAAAGT.........   6 P7-1 +/-
    ....................................................................................   21 P7-1 +/-
    ----------------------------------------------------------------------------------------------------
    ..............................................TGGTGATGAACAGCAAGCAGTATGAAAGT.........   7 P10-1 +/-
    .............................................TTGGTGATGAACAGCAAGCAGTATGAAAGT.........   7 P10-1 +/-
    ....................................................................................   14 P10-1 +/-
    ----------------------------------------------------------------------------------------------------
```

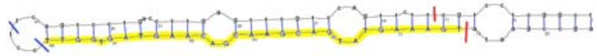

high confidence

```
158 GTTCTTGGCCAAGGATGAGAACTCTAACCTGACAGGATACGCTGCTGTGATGGGTTAAAGGATTTACCTGAGGCCAACAGAAC
    (((((((((.(((.((((..((.((((((((((((.(.....).))))..)))))))).))..))))))).)))))..)))))
    ******GGCCAAGGATGAGAACTCTAACCTGAC**************************************************
    ......GGCCAAGGATGAGAACTCTAACCTGAC..................................................   5 P7-1 +/-
    ...................................................................................   5 P7-1 +/-
    ----------------------------------------------------------------------------------------------------
    ......GGCCAAGGATGAGAACTCTAACCTGAC..................................................   5 P10-4 -/-
    ...................................................................................   5 P10-4 -/-
    ----------------------------------------------------------------------------------------------------
```

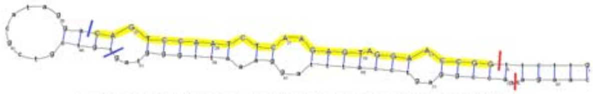

high confidence

```
159 GCATGTTCCCACCGTCTGTAAATCTGTCCCAGCCGTGCTGCAGTCACAGTACTGGAACTAGAGCACTGCGGATGTGATCATGT
    ((((.((.(((((((..((...(((((.(((...((((((......))))))))).)).))).))..)).)))).)).)).)))))
    *****************************************************TGGAACTAGAGCACTGCGGATGTGATC*****
    .....................................................TGGAACTAGAGCACTGCGGATGTGATC.....   7 P7-1 +/-
    ...................................................................................   7 P7-1 +/-
    ----------------------------------------------------------------------------------------------------
```

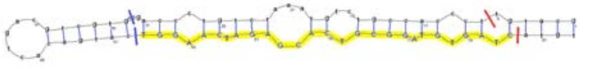

high confidence

```
160 GCAGCAGAACACCTCTTTTTCAGGAGCTGGCGCCATGCTCAGCCATGCCTGGTAGTCTCTGTGGAAAGAGCTGGCTTGCTGT
    ((((((..((.((((((((((((..(((...((((.((........)).))))))))..))).))))))))).))..)))))))
    ************************************************TGGTAGTCTCTGTGGAAAGAGCTGGC********
    ................................................TGGTAGTCTCTGTGGAAAGAGCTGGC........   7 P7-1 +/-
    ..................................................................................   7 P7-1 +/-
    ----------------------------------------------------------------------------------------------------
    ................................................TGGTAGTCTCTGTGGAAAGAGCTGGC........   11 P10-1 +/-
    ..................................................................................   11 P10-1 +/-
    ----------------------------------------------------------------------------------------------------
```

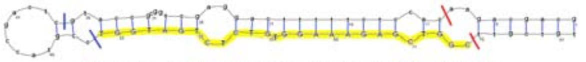

high confidence

161
```
CTGCTTCTGTTTCGCTTCTGCTTCAGACTCAAGAGCAACAGGATGTTCTAGAGAACCTGAAGCATGAACTGGCCACCAGCAG
(((((..((..(((.(((((((((((.(((.((((((......)))))).)))...)))))))).))).))).))..)))))
************************************************TAGAGAACCTGAAGCATGAACTGGCC********
................................................TAGAGAACCTGAAGCATGAACTGGCC........
................................................TAGAGAACCTGAAGCATGAACTGGCCA.......
................................................TAGAGAACCTGAAGCATGAACTGGCCAC......
..................................................................................
----------------------------------------------------------------------------------------------------
................................................TAGAGAACCTGAAGCATGAACTGGCC........
................................................TAGAGAACCTGAAGCATGAACTGGCCA.......
..................................................................................
----------------------------------------------------------------------------------------------------
................................................TAGAGAACCTGAAGCATGAACTGGCC........
................................................TAGAGAACCTGAAGCATGAACTGGCCA.......
..................................................................................
----------------------------------------------------------------------------------------------------
................................................TAGAGAACCTGAAGCATGAACTGGCC........
..................................................................................
----------------------------------------------------------------------------------------------------
```

22 P7-1 +/-
11 P7-1 +/-
10 P7-1 +/-
43 P7-1 +/-

37 P10-1 +/-
7 P10-1 +/-
44 P10-1 +/-

33 P10-2 +/-
6 P10-2 +/-
39 P10-2 +/-

15 P14-1 +/-
15 P14-1 +/-

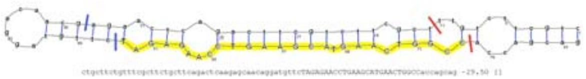

non-candidate

162
```
CTGAGCACTTGGGAAGCAGAGGCAGGGGGGTTTCTGAGTTGGAGGCCAGCCTGGTCTACAGAGTGAGTTCCAGGACAGCCAG
(((.((.(((((((..((.(((((((..(((((((.....)))))))..))).))))......))..)))))))...)))))
***************************************************TGGTCTACAGAGTGAGTTCCAGGACA*****
.........TGGGAAGCAGAGGCAGGGGGGTTTCT...............................................
...................................................TGGTCTACAGAGTGAGTTCCAGGACA.....
...................................................TGGTCTACAGAGTGAGTTCCAGGACAG....
..................................................................................
----------------------------------------------------------------------------------------------------
```

6 P10-1 +/-
26 P10-1 +/-
5 P10-1 +/-
37 P10-1 +/-

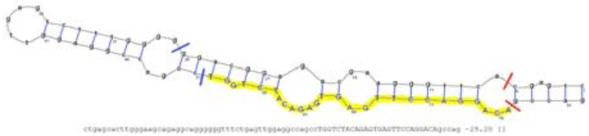

potential

163
```
GAATAAGCTGGGTATGGTGGCTCCTGCCTTTATTTCCAGCCCTTGGGAAGCAGAGGCAGGTGAGTTCAGGGCCAGCTTGGTC
((.((((((((.(.(((...(.((((((((((.((((((...)))))))..))))))))).)...))).).)))))))).))
*******************************************TGGGAAGCAGAGGCAGGTGAGTT****************
...........................................TGGGAAGCAGAGGCAGGTGAGTT................
..................................................................................
----------------------------------------------------------------------------------------------------
```

8 P10-2 +/-
8 P10-2 +/-

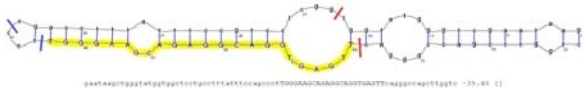

potential

164
```
GTGAGGTTCATAAGTGGTCTTGGCTCTTTTCTCTGTAGCAAGACTCCTCAGCCTGAGACTCAGGACAGCTTGCAGACCTCAT
((((((((..(((((.((((((..((((....(((.((........)))))...)))).)))))).)))))..))))))))
*****************************************************TGAGACTCAGGACAGCTTGCAGACCTC**
.....................................................TGAGACTCAGGACAGCTTGCAGACCTC..
..................................................................................
----------------------------------------------------------------------------------------------------
.....................................................TGAGACTCAGGACAGCTTGCAGACCTC..
..................................................................................
----------------------------------------------------------------------------------------------------
```

6 P10-1 +/-
6 P10-1 +/-

8 P10-2 +/-
8 P10-2 +/-

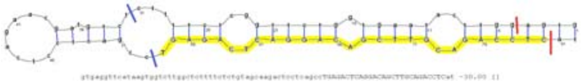

high confidence

165
```
GGCACATTTGCAACTAGACGCTGAGGACAGAGGGCAGGCTTCAGGCCAAATGTCCCTACTCCTCGGATGATCTGGTCAAGCC
(((....(((..(((((((((((((((...(((((((((.....)))...)).)))))..)))))))).)).))))))))))
********TGCAACTAGACGCTGAGGACAGAGGGC***********************************************
........TGCAACTAGACGCTGAGGACAGAGGGC...............................................
..................................................................................
----------------------------------------------------------------------------------------------------
```

5 P10-1 +/-
5 P10-1 +/-

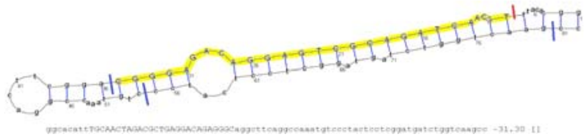

high confidence

166
```
TCAGGGTATGGGGTGGGCTTAGCACTTTAGTGGATTGAGACATTATTCCATTCTGAAGGTGTCGTGTGTTCTGTGCTCCTGA
((((((((((..((.((...((((((((((((.(((....))).))))))...))))))).)).))..)))))).)))))
*********************************************TTCCATTCTGAAGGTGTCGTGTGTTC***********
.............................................TTCCATTCTGAAGGTGTCGTGTGTTC...........
..................................................................................
----------------------------------------------------------------------------------------------------
.............................................TTCCATTCTGAAGGTGTCGTGTGTT............
.............................................TTCCATTCTGAAGGTGTCGTGTGTTC...........
..................................................................................
----------------------------------------------------------------------------------------------------
```

5 P7-1 +/-
5 P7-1 +/-

6 P10-2 +/-
5 P10-2 +/-
11 P10-2 +/-

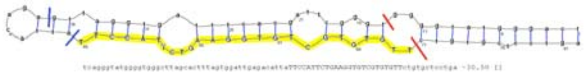

non-candidate

167
```
AGGGTAAAGGAACCATGGCAGGAGCTGTGTACAAGAGGAGCCTCTTCCTCTTCCGGTTCAGCACAGGTTACCTCCCGCCCT
((((..((((((.((((..(((((.(...(((((((....)))))))))))))).)).))))))).)))...)))))
***************TGGCAGGAGCTGTGTACAAGAGGAGCC***************************************
...............TGGCAGGAGCTGTGTACAAGAGGAGCC.......................................
.................................................................................
----------------------------------------------------------------------------------------------------
```

6 P10-1 +/-
6 P10-1 +/-

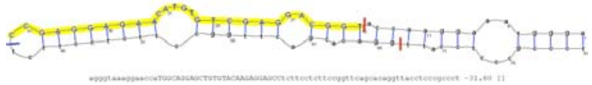

high confidence

168
```
TGGTGAGGAGAGGTGGAAGTATGGAAGGGGGTGGCTTTTCCAGCCAACTCCCCCTTATATTTATGGTACCTTTCCTTCCCA
.((..((((((((((.(((((((..((((((((((((.....)))))...)))))))))))))....))))))))))..)).
**GTGAGGAGAGGTGGAAGTATGGAAGG*****************************************************
..GTGAGGAGAGGTGGAAGTATGGAAGG.....................................................
.................................................................................
----------------------------------------------------------------------------------------------------
```

6 P7-1 +/-
6 P7-1 +/-

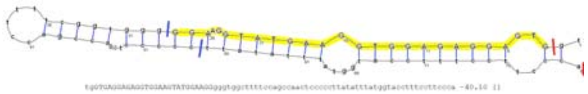

potential

169
```
GGTTCTCTCTGCTTCACTTTTAGCTGTCCCTGTGGCTCTGCCCAGGGCTGCAAGAACGTTGACGTGGAGCGGTCCACGCC
(((.....(((((((((..(((((.((.(.((..(((((....)))))..)).).))))))).))))))))).....)))
************************************************TGCAAGAACGTTGACGTGGAGCGGTC******
................................................TGCAAGAACGTTGACGTGGAGCGGTC......
................................................................................
----------------------------------------------------------------------------------------------------
```

5 P7-1 +/-
5 P7-1 +/-

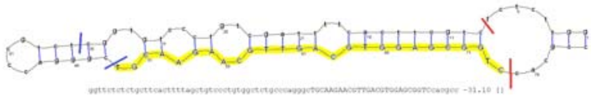

non-candidate

```
170 TGTGCCTATGTGCTGTAATACTGGGAGGCAAAGGAAAGGGCCATGGCGTCCCTTTCTGTTACCAGGAGCATCTAGGCAGG
    ..((((((.((((((((((..((((.((...((......))...)).))))....))))))....)))))).)))))).. 
    ********TGTGCTGTAATACTGGGAGGCAAAGGA*********************************************
    ........TGTGCTGTAATACTGGGAGGCAAAGGA.............................................    6 P7-1 +/-
    ................................................................................    6 P7-1 +/-
    ----------------------------------------------------------------------------------------------------
```

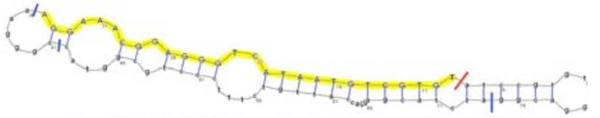

tgtgcctaTGTGCTGTAATACTGGGAGGCAAAGGAaagggccatggcgtccctttctgttaccaggagcatctaggcagg -31.40 []

potential

```
171 TCAGCTCCTGCTTTCTGAACTGCTTGAGTTCCAGTCCTGACTTCCTTGGTGATGAACAGCAGTATGGAAGGGTAAGCTGA
    (((((....((((((((.((((((...((((..((((..(.....)..).)))))))))))))).))))))))...))))))
    *********************************************TTGGTGATGAACAGCAGTATGGAAGGGTA******
    .............................................TTGGTGATGAACAGCAGTATGGAAGGGTA......    6 P10-1 +/-
    ................................................................................    6 P10-1 +/-
    ----------------------------------------------------------------------------------------------------
```

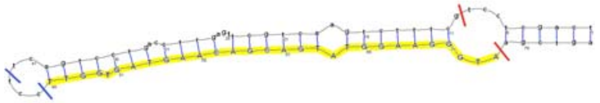

tcagctcctgctttctgaactgcttgagttccagtcctgacttccTTGGTGATGAACAGCAGTATGGAAGGGTAagctga -25.70 []

high confidence

```
172 AGTCCTGGAACTCGCTCTGTAGACCAGGCTATCCCCAAACTCAGAGATTCGCCTGCCTCTGCCTTCTGAGTGCTGGGACT
    (((((..(.(((((....(((((.(((((.(((.(........).)))..)))))..)))))....))))).)..)))))
    ***********TCGCTCTGTAGACCAGGCTATC***********************************************
    ...........TCGCTCTGTAGACCAGGCTATC...............................................   12 P7-1 +/-
    ...........TCGCTCTGTAGACCAGGCTA.................................................    7 P7-1 +/-
    ............CGCTCTGTAGACCAGGCTATC...............................................    5 P7-1 +/-
    ................................................................................   24 P7-1 +/-
    ----------------------------------------------------------------------------------------------------
    ...........TCGCTCTGTAGACCAGGCTATC...............................................    9 P10-1 +/-
    ................................................................................    9 P10-1 +/-
    ----------------------------------------------------------------------------------------------------
    ...........TCGCTCTGTAGACCAGGCTATC...............................................   10 P10-2 +/-
    ...........TCGCTCTGTAGACCAGGCTAT................................................    5 P10-2 +/-
    ................................................................................   15 P10-2 +/-
    ----------------------------------------------------------------------------------------------------
```

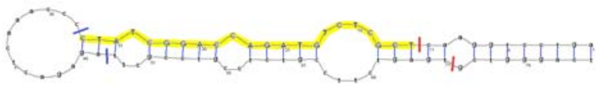

agtcctggaacTCGCTCTGTAGACCAGGCTATCcccaaactcagagattcgcctgcctctgccttctgagtgctgggact -28.30 []

high confidence

```
173 AGGGCCTCCTCCTTCACAGGCCTGGGGAGGTATTCAGAGTCGCAGGCCTTTAGGCCTTCTGAAGCGTAGGAAAGGTTCT
    (((((((((((((((.(((((((..(((((...(......)...))))))))))))..)))))).).)))))..))))))
    **************************************************TAGGCCTTCTGAAGCGTAGGAAAGGTT***
    ..................................................TAGGCCTTCTGAAGCGTAGGAAAGGTT...    5 P7-1 +/-
    ...............................................................................    5 P7-1 +/-
    ----------------------------------------------------------------------------------------------------
    ..................................................TAGGCCTTCTGAAGCGTAGGAAAGGTT...    8 P10-1 +/-
    ...............................................................................    8 P10-1 +/-
    ----------------------------------------------------------------------------------------------------
```

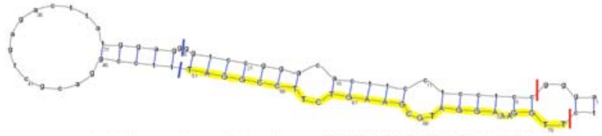

agggcctcctccttcacaggcctggggaggtattcagagtcgcaggcctttTAGGCCTTCTGAAGCGTAGGAAAGGTTct -28.94 []

high confidence

```
174 GCTGCATTCCTGGGACATGTGACAGCAGCCTCCCCTTTCTCTGGGAGGAAGGCTTCTGTCTTGTCTTGGGTGTGATAGC
    ((((((.((..(((((...(((((.(((((((((((......))).)).)))))).))))).)))))..)).))).))))
    **TGCATTCCTGGGACATGTGACAGCAGC**************************************************
    ..TGCATTCCTGGGACATGTGACAGCAGC..................................................    5 P10-1 +/-
    ...............................................................................    5 P10-1 +/-
    ----------------------------------------------------------------------------------------------------
```

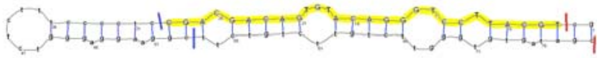

gcTGCATTCCTGGGACATGTGACAGCAGCctcccctttctctgggaggaaggcttctgtcttgtcttgggtgtgatagc -42.00 []

high confidence

```
175 TGTGGCTCAGTGCTTCGAGTCAGCGTTGTGATCTCAGTTCCTCTGTGAACTACGATGCTGAATGAGTCATTCAGCCTCA
    .(.((((.((((.((((..(((((((((((.((.(((.....))).))..)))))))))))).)))).)))).)))).).
    *******************************************TGTGAACTACGATGCTGAATGAGTCA**********
    ...........................................TGTGAACTACGATGCTGAATGAGTCA..........    6 P14-1 +/-
    ...........................................TGTGAACTACGATGCTGAATGAGTCAT.........    6 P14-1 +/-
    ...............................................................................   12 P14-1 +/-
    ----------------------------------------------------------------------------------------------------
```

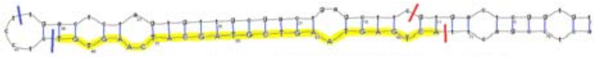

tgtggctcagtgcttcgagtcagcgttgtgatctcagttcctcTGTGAACTACGATGCTGAATGAGTCAttcagcctca -31.10 []

high confidence

```
176 CTGAGTCTCAGTGGTCAGAGCACTCTCTGCAGGCAAGCTCTCCTCTTGCAGGGAAGGTGCACAGAAGTCTGCAGCTCAG
    (((((..(((...((.(.((((((((((((((..........)))))))))..))))).).))...)))..))))))
    *********************************************TTGCAGGGAAGGTGCACAGAAGTCTGC*******
    .............................................TTGCAGGGAAGGTGCACAGAAGTCTGC.......   39 P7-1 +/-
    .............................................TTGCAGGGAAGGTGCACAGAAGTCTG........   13 P7-1 +/-
    ..............................................TGCAGGGAAGGTGCACAGAAGTCTGC.......    7 P7-1 +/-
    ...............................................................................   59 P7-1 +/-
    ----------------------------------------------------------------------------------------------------
    .............................................TTGCAGGGAAGGTGCACAGAAGTCTGC.......   31 P10-1 +/-
    .............................................TTGCAGGGAAGGTGCACAGAAGTCTG........   14 P10-1 +/-
    ..............................................TGCAGGGAAGGTGCACAGAAGTCTGC.......    7 P10-1 +/-
    ...............................................................................   52 P10-1 +/-
    ----------------------------------------------------------------------------------------------------
    .............................................TTGCAGGGAAGGTGCACAGAAGTCTGC.......   30 P10-2 +/-
    .............................................TTGCAGGGAAGGTGCACAGAAGTCTG........    9 P10-2 +/-
    ..............................................TGCAGGGAAGGTGCACAGAAGTCTGC.......    5 P10-2 +/-
    ...............................................................................   44 P10-2 +/-
    ----------------------------------------------------------------------------------------------------
    .............................................TTGCAGGGAAGGTGCACAGAAGTCTGC.......    8 P14-1 +/-
    ...............................................................................    8 P14-1 +/-
    ----------------------------------------------------------------------------------------------------
```

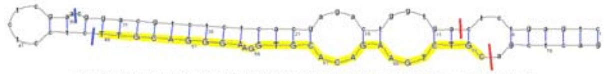

ctgagtctcagtggtcagagcactctctgcaggcaagctctcctcTTGCAGGGAAGGTGCACAGAAGTCTGCagctcag -30.10 []

high confidence

```
177 GCTGATTTTCCTGACTTGTAGGGAACGCAGAGATACACAAGTAGTCTTAAATGCTTCTCTACCAGAGGGAATCTTTAGT
    (((((..(((((..((.((((((((.((((((((((....)).)))))...)))))))))))).)).))))))...)))))
    ********TCCTGACTTGTAGGGAACGCAGAGA**********************************************
    ........TCCTGACTTGTAGGGAACGCAGAGA..............................................   21 P14-1 +/-
    ...........TGACTTGTAGGGAACGCAGAGATACA..........................................   21 P14-1 +/-
    ...........TGACTTGTAGGGAACGCAGAGATAC...........................................   20 P14-1 +/-
    ........TCCTGACTTGTAGGGAACGCAGAGAT.............................................   12 P14-1 +/-
    ...........TGACTTGTAGGGAACGCAGAGATACAC.........................................   12 P14-1 +/-
    ........TCCTGACTTGTAGGGAACGCAGAGATA............................................    5 P14-1 +/-
    ...............................................................................   91 P14-1 +/-
    ----------------------------------------------------------------------------------------------------
```

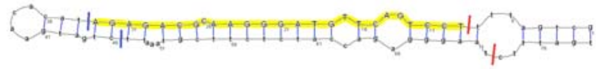

gctgattTCCTGACTTGTAGGGAACGCAGAGAtacacaagtagtcttaaatgcttctctaccagagggaatctttagt -26.60 []

potential

```
178 TCCTGGAATTCACTTTGTAGAGCAGGCTGGTCTTGAACTCACAGAAATTTGCCTGCCTCTGTCTCCCAGGTGCTAGGA
    (((((....(((((.(((((((((((...(((((....)).)))....))))).))))).)....))))))))))
    ********TTCACTTTGTAGAGCAGGCTGG*********************************************
    ........TTCACTTTGTAGAGCAGGCTGG.............................................   21  P7-1 +/-
    ........TTCACTTTGTAGAGCAGGCTGGT............................................    5  P7-1 +/-
    ..........................................................................   26  P7-1 +/-
    ------------------------------------------------------------------------------------------------------
    ........TTCACTTTGTAGAGCAGGCTGG.............................................   47  P7-3 -/-
    ........TTCACTTTGTAGAGCAGGCTGGT............................................   12  P7-3 -/-
    .........TCACTTTGTAGAGCAGGCTGG.............................................   11  P7-3 -/-
    ........TTCACTTTGTAGAGCAGGCTG..............................................   10  P7-3 -/-
    .........TCACTTTGTAGAGCAGGCTG..............................................    6  P7-3 -/-
    ..........................................................................   86  P7-3 -/-
    ------------------------------------------------------------------------------------------------------
    ........TTCACTTTGTAGAGCAGGCTGG.............................................   12  P7-4 -/-
    ........TTCACTTTGTAGAGCAGGCTGGT............................................    5  P7-4 -/-
    ..........................................................................   17  P7-4 -/-
    ------------------------------------------------------------------------------------------------------
    ........TTCACTTTGTAGAGCAGGCTGG.............................................   24  P10-3 -/-
    .........TCACTTTGTAGAGCAGGCTGG.............................................   13  P10-3 -/-
    ........TTCACTTTGTAGAGCAGGCTGGT............................................   12  P10-3 -/-
    ..........................................................................   49  P10-3 -/-
    ------------------------------------------------------------------------------------------------------
    ........TTCACTTTGTAGAGCAGGCTGG.............................................   33  P10-4 -/-
    .........TCACTTTGTAGAGCAGGCTGG.............................................   16  P10-4 -/-
    ........TTCACTTTGTAGAGCAGGCTGGT............................................    6  P10-4 -/-
    ..........................................................................   55  P10-4 -/-
    ------------------------------------------------------------------------------------------------------
    ........TTCACTTTGTAGAGCAGGCTGG.............................................   18  P14-1 +/-
    ........TTCACTTTGTAGAGCAGGCTGGT............................................    5  P14-1 +/-
    ..........................................................................   23  P14-1 +/-
    ------------------------------------------------------------------------------------------------------
    ........TTCACTTTGTAGAGCAGGCTGG.............................................   31  P14-4 -/-
    .........TCACTTTGTAGAGCAGGCTGG.............................................    8  P14-4 -/-
    ........TTCACTTTGTAGAGCAGGCTGGT............................................    8  P14-4 -/-
    ........TTCACTTTGTAGAGCAGGCTG..............................................    5  P14-4 -/-
    ..........................................................................   52  P14-4 -/-
    ------------------------------------------------------------------------------------------------------
```

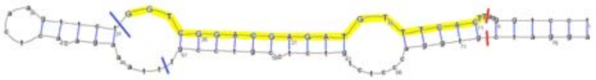

tcctggaaTTCACTTTGTAGAGCAGGCTGGtcttgaactcacagaaatttgcctgcctctgtctcccaggtgctagga -24.20 []

potential

```
179 CTGCCCTGATTTTTATCGATTCTTCTCATCTTCACTTCTTACTAGTAAAGAAGAGATTTGGAGAAGGTCAGGTGACAG
    (((((((((((((.((((....((((.((((.(((.......))).)))).)))).)))).))))))))))).).)))
    **************************************TAGTAAAGAAGAGATTTGGAGAAGGT***********
    ......................................TAGTAAAGAAGAGATTTGGAGAAGGT...........   20  P14-1 +/-
    ......................................TAGTAAAGAAGAGATTTGGAGAAGGTC..........   18  P14-1 +/-
    ......................................TAGTAAAGAAGAGATTTGGAGAAGGTCA.........   17  P14-1 +/-
    ......................................TAGTAAAGAAGAGATTTGGAGAAGGTCAG........    9  P14-1 +/-
    ......................................TAGTAAAGAAGAGATTTGGAGAAGG............    5  P14-1 +/-
    ..........................................................................   69  P14-1 +/-
    ------------------------------------------------------------------------------------------------------
```

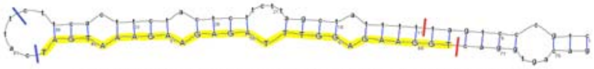

ctgccctgatttttatcgattcttctcatcttcacttcttacTAGTAAAGAAGAGATTTGGAGAAGGTcaggtgacag -24.00 []

non-candidate

```
180 TGGCTGTTCTGTCCCCAGCACTCCCAGCAGCTATACAGGCACATCTACCTGAGCTGTAAGGAGGAGACGAACATCCA
    .((.(((((.(((.....(.((((..((((((...((((........))))))))))..)))))).)))))))).)).
    *****************************************TACCTGAGCTGTAAGGAGGAGACGAAC******
    .........................................TACCTGAGCTGTAAGGAGGAGACGAAC......    9  P7-1 +/-
    .........................................................................    9  P7-1 +/-
    ------------------------------------------------------------------------------------------------------
    .........................................TACCTGAGCTGTAAGGAGGAGACGAAC......    5  P10-1 +/-
    .........................................................................    5  P10-1 +/-
    ------------------------------------------------------------------------------------------------------
```

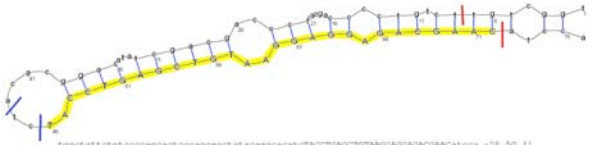

tggctgttctgtccccagcactcccagcagctatacaggcacatcTACCTGAGCTGTAAGGAGGAGACGAACatcca -28.50 []

non-candidate

```
181 ACTGGGCATCTTGTTCTGAGCAATCCCCTGTCTCTGCCCTCCAAGGCAGGAGATATACTCAGGACACTGCTCCTGT
    ...(((((...((((((((..(((.(((((((.((.....))))))))).)))...))))))))).))))).....
    ************************************TCCAAGGCAGGAGATATACTCAGGAC************
    ....................................TCCAAGGCAGGAGATATACTCAGGAC............    6  P10-2 +/-
    ..........................................................................    6  P10-2 +/-
    ------------------------------------------------------------------------------------------------------
```

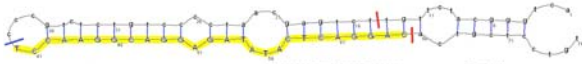

actgggcatcttgttctgagcaatcccctgtctctgccctCCAAGGCAGGAGATATACTCAGGACactgctcctgt -29.70 []

potential

```
182 CCCACCTCCCCCTACATCTTCACCATGGCCAGTTTAGGACTAGGCTATAGGAGGACCAGGAGAGGACTATAGTGGG
    (((((.(((((((...(((((.(.((((((((((...)))).)))))).)))))))..))).).))).....)))))
    ******************************************TATAGGAGGACCAGGAGAGGACTATAGT****
    ..........................................TATAGGAGGACCAGGAGAGGACTATAGT....    9  P7-1 +/-
    ..........................................................................    9  P7-1 +/-
    ------------------------------------------------------------------------------------------------------
```

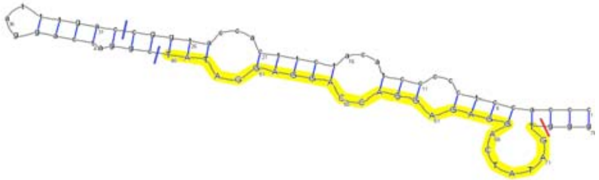

cccacctcccccctacatcttcaccatggccagtttaggactaggcTATAGGAGGACCAGGAGAGGACTATAGTggg -25.61 []

high confidence

```
183 TCTGAACTCATCTGGAGGCCTGACATCGTCCTCTACAACAAGTAAGCCGTGTGGGCCCTGGCAGGTGAGATCAGG
    .((((.((((((((..(((((.((((.((...((......))...)).))))))))))....)))))))).)))).
    TCTGAACTCATCTGGAGGCCTGACATC************************************************
    TCTGAACTCATCTGGAGGCCTGACATC................................................    6  P7-1 +/-
    ..........................................................................    6  P7-1 +/-
    ------------------------------------------------------------------------------------------------------
```

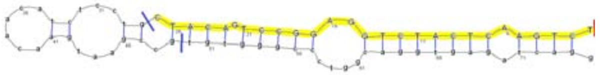

TCTGAACTCATCTGGAGGCCTGACATCgtcctctacaacaagtaagccgtgtgggccctggcaggtgagatcagg -28.20 []

non-candidate

```
184 TGGAAAAGGCAGTGATGTTTCCAAGCACATATCTGACTTATATGTGTGGATGGTAATAATGCTGAGTCTTTTCCG
    .(((((((((((((.((((.(((.((((((((.......))))))))...))).)))).)))))..))))))))).
    **********************************TATATGTGTGGATGGTAATAATGCTGAGT*********
    ..................................TATATGTGTGGATGGTAATAATGCTGAGT.........    5  P7-1 +/-
    ..........................................................................    5  P7-1 +/-
    ------------------------------------------------------------------------------------------------------
    ..................................TATATGTGTGGATGGTAATAATGCTGAGT.........    7  P7-4 -/-
    ..........................................................................    7  P7-4 -/-
    ------------------------------------------------------------------------------------------------------
```

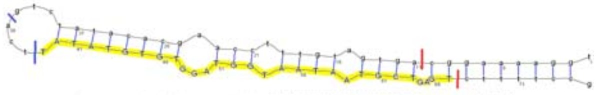

tggaaaaggcagtgatgtttccaagcacatatctgacttTATATGTGTGGATGGTAATAATGCTGAGTcttttccg -26.60 []

high confidence

```
185 TCCCCATCCCAGCTTCCTTCCAGCCCTTGATGTTTTAGTACGCACATAGGCTAGAGAGAGGTTGGGGATGGGGA
    ((((((((((((((.(((.((((..((.(((........))))).)))).))).)))))).))))))))))
    **********************************************TAGGCTAGAGAGAGGTTGGGGA*******
    ..............................................TAGGCTAGAGAGAGGTTGGGGA.......      5  P7-1 +/-
    ..........................................................................      5  P7-1 +/-
    ----------------------------------------------------------------------------------------------------
    ..............................................TAGGCTAGAGAGAGGTTGGGGA.......     16  P7-3 -/-
    ..............................................TAGGCTAGAGAGAGGTTGGGGAT......     11  P7-3 -/-
    ..............................................TAGGCTAGAGAGAGGTTGGG.........      6  P7-3 -/-
    ..........................................................................     33  P7-3 -/-
    ----------------------------------------------------------------------------------------------------
    ..............................................TAGGCTAGAGAGAGGTTGGGGA.......     13  P7-4 -/-
    ..............................................TAGGCTAGAGAGAGGTTGGGGAT......      9  P7-4 -/-
    ..............................................TAGGCTAGAGAGAGGTTGGGG........      5  P7-4 -/-
    ..........................................................................     27  P7-4 -/-
    ----------------------------------------------------------------------------------------------------
    ..............................................TAGGCTAGAGAGAGGTTGGGGA.......      5  P10-3 -/-
    ..........................................................................      5  P10-3 -/-
    ----------------------------------------------------------------------------------------------------
```

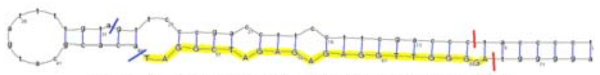

tccccatcccagcttccttccagcccttgatgttttagtacgcacaTAGGCTAGAGAGAGGTTGGGGAtgggga -38.90 []

potential

```
186 AGAATTCAGTTAGAAATTACATTGATTTAAGAAGTGGTAATGGGCTTAGATCGATGTGGTGCTCCTGAGTTCT
    (((((((((...((.((((((((((((((((..((....))...))))))))))))))))..)))))))))))
    ********************************************CTTAGATCGATGTGGTGCT***********
    ................................................GATCGATGTGGTGCTC.........     15  P14-4 -/-
    ............................................CTTAGATCGATGTGGTGCT..........      5  P14-4 -/-
    ............................................CTTAGATCGATGTGGTGCTC.........      5  P14-4 -/-
    .........................................................................     25  P14-4 -/-
    ----------------------------------------------------------------------------------------------------
```

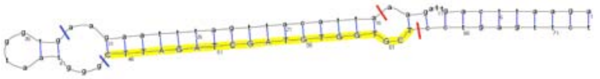

agaattcagttagaaattacattgatttaagaagtggtaatgggCTTAGATCGATGTGGTGCTcctgagttct -27.70 []

high confidence

```
187 TCTGAAAGGGCACAGACCATGGGCGTGAACATTGTGCCGTACCTGAGATGGTCTGTGGACTGCTGCTTCAGA
    ((((((((..((((((((((((((..((.((...))..))..)))...))))))))))..)).....))))))
    TCTGAAAGGGCACAGACCATGGGCGT**********************************************
    TCTGAAAGGGCACAGACCATGGGCGT..............................................      8  P10-1 +/-
    ........................................................................      8  P10-1 +/-
    ----------------------------------------------------------------------------------------------------
    TCTGAAAGGGCACAGACCATGGGCGT..............................................      5  P10-2 +/-
    ........................................................................      5  P10-2 +/-
    ----------------------------------------------------------------------------------------------------
```

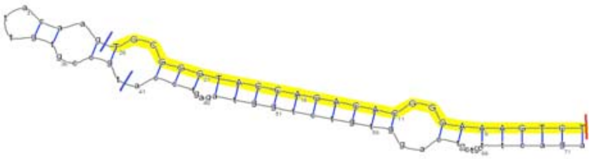

TCTGAAAGGGCACAGACCATGGGCGTgaacattgtgccgtacctgagatggtctgtggactgctgcttcaga -25.20 []

high confidence

```
188 CTCCATGGAGCAGGCGGCGCCCAGTAGTGGTAGAGCCCATCTCTGATGAAGACTGGCACCTATTCTGTGGAG
    ((((((((((.(((.(....(((((..(.((((((.....)))).)).)..)))))).))).))))))))))
    *******************************************TGATGAAGACTGGCACCTATTCTGTG****
    ...........................................TGATGAAGACTGGCACCTATTCTGTG....      5  P10-1 +/-
    ........................................................................      5  P10-1 +/-
    ----------------------------------------------------------------------------------------------------
```

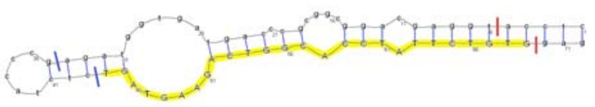

high confidence

```
189 GCCATGGAGAATCTGAAAGGAGGAGGTCAGCATCAGGGTCTGACAGGATTTTCAGGCTTTTTCCAGAGGGC
    (((.((((((((((((((((......(((((.((....))))))))....))))))))..))))))))...)))
    **************************************TCTGACAGGATTTTCAGGCTTTTTCCAGA*****
    ......................................TCTGACAGGATTTTCAGGCTTTTTCCAGA.....      5  P7-1 +/-
    .......................................................................      5  P7-1 +/-
    ----------------------------------------------------------------------------------------------------
```

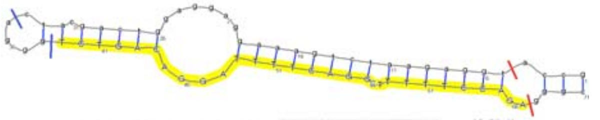

gccatggagaatctgaaaggaggaggtcagcatcagggTCTGACAGGATTTTCAGGCTTTTTCCAGAgggc -25.70 []

high confidence

```
190 GTCTGGCGGCAGTCAGGATACCTGTCTTTCCTGGTAGGAATGAGTTACAGGTGTTTCCTCTGCCGTCACAC
    ((.(((((((((...(((((((((((((((((.....)))..)))..)))))))))))...))))))))).))
    ***TGGCGGCAGTCAGGATACCTGT**********************************************
    ...TGGCGGCAGTCAGGATACCTGT..............................................      7  P7-1 +/-
    .......................................................................      7  P7-1 +/-
    ----------------------------------------------------------------------------------------------------
    ...TGGCGGCAGTCAGGATACCTGT..............................................      5  P7-2 +/-
    .......................................................................      5  P7-2 +/-
    ----------------------------------------------------------------------------------------------------
    ...TGGCGGCAGTCAGGATACCTGT..............................................     18  P7-3 -/-
    .......................................................................     18  P7-3 -/-
    ----------------------------------------------------------------------------------------------------
    ...TGGCGGCAGTCAGGATACCTGT..............................................     15  P7-4 -/-
    .......................................................................     15  P7-4 -/-
    ----------------------------------------------------------------------------------------------------
    ...TGGCGGCAGTCAGGATACCTGT..............................................      9  P10-1 +/-
    .......................................................................      9  P10-1 +/-
    ----------------------------------------------------------------------------------------------------
    ...TGGCGGCAGTCAGGATACCTGT..............................................      7  P10-2 +/-
    .......................................................................      7  P10-2 +/-
    ----------------------------------------------------------------------------------------------------
    ...TGGCGGCAGTCAGGATACCTGT..............................................     14  P10-3 -/-
    .......................................................................     14  P10-3 -/-
    ----------------------------------------------------------------------------------------------------
    ...TGGCGGCAGTCAGGATACCTGT..............................................     14  P10-4 -/-
    .......................................................................     14  P10-4 -/-
    ----------------------------------------------------------------------------------------------------
    ...TGGCGGCAGTCAGGATACCTGT..............................................      8  P14-3 -/-
    .......................................................................      8  P14-3 -/-
    ----------------------------------------------------------------------------------------------------
```

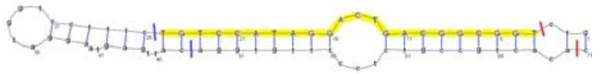

gtcTGGCGGCAGTCAGGATACCTGTctttcctggtaggaatgagttacaggtgtttcctctgccgtcacac -32.10 []

high confidence

```
191 GCTACTCTTTCTGTGTGTCCGAGGCTTCCAGCCTGGAGGTTCTCGGCAGTGTGCAGCATGAGGCCTGAGC
    (((.(((...(((((..((((((((((((.....))))).)))))))..)..)))))...))).....)))
    **TACTCTTTCTGTGTGTCCGAGGCTTC******************************************
    ..TACTCTTTCTGTGTGTCCGAGGCTTC..........................................      5  P10-1 +/-
    ......................................................................      5  P10-1 +/-
    ----------------------------------------------------------------------------------------------------
    ..TACTCTTTCTGTGTGTCCGAGGCTTC..........................................      5  P10-2 +/-
    ......................................................................      5  P10-2 +/-
    ----------------------------------------------------------------------------------------------------
```

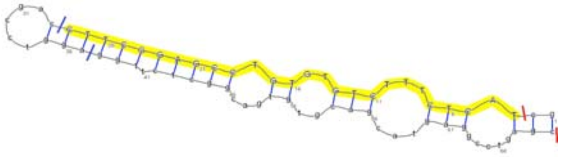

gcTACTCTTTCTGTGTGTCCGAGGCTTCcagcctggaggttctcggcagtgtgcagcatgaggcctgagc -25.20 []

non-candidate 192

```
CCTTTGTTAATCCTCTCACTAGCATCATCTGCACTTAGGAGTTGTGCAGTGAAGAGGATGGCTGAGG
((((.((((.((((((((((.(((.((.((.(.....).)).)))))))))).)))))))))).))))
***********************************TAGGAGTTGTGCAGTGAAGAGGATGG*******
...................................TAGGAGTTGTGCAGTGAAGAGGATGG.......
...................................TAGGAGTTGTGCAGTGAAGAGGATGGC......
...................................................................
------------------------------------------------------------------------------------------
...................................TAGGAGTTGTGCAGTGAAGAGGATGG.......
...................................................................
------------------------------------------------------------------------------------------
...................................TAGGAGTTGTGCAGTGAAGAGGATGG.......
...................................................................
------------------------------------------------------------------------------------------
```

23 P7-1 +/-
9 P7-1 +/-
32 P7-1 +/-
15 P10-1 +/-
15 P10-1 +/-
9 P10-2 +/-
9 P10-2 +/-

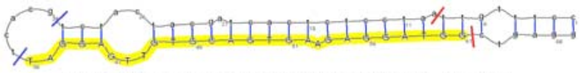

high confidence 193

```
AACTCTTCCCAAGTATGGAACCAGGGGCTTCTGGCAAGCCTGTCCTGGCACTGTGGTGGGACTGGTT
((((..(((((..(((((..((((((((((.....)))))...))))))..))))).))))))..))))
******************************************TCCTGGCACTGTGGTGGGACTGGTT*
..........................................TCCTGGCACTGTGGTGGGACTGGTT.
...................................................................
------------------------------------------------------------------------------------------
```

5 P7-1 +/-
5 P7-1 +/-

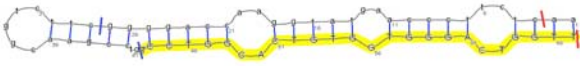

high confidence 194

```
CCCAGCTTGTGCTTAGACCTGTCAGTCAGTGCTCTCACTGACATAGCTGAGCACTCAAAGATGGG
((((.((((((((((...(((((((.((....)).))))))))...))))))))...))).))))
**************************************TGACATAGCTGAGCACTCAAAGATGG**
......................................TGACATAGCTGAGCACTCAAAGATGG..
.................................................................
------------------------------------------------------------------------------------------
```

5 P10-2 +/-
5 P10-2 +/-

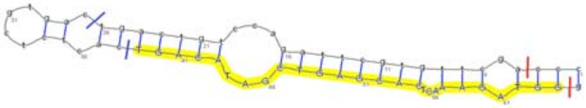

potential 195

```
GTGTCTGAGTGCTTGTCCTGCCCTAGCAGGACTCTGATGGGGAACAGGAGGACTCGCCGGCCAC
(((.(((.(((.((.((((((((((.(((....))).))))))..))))).))..)))))).)))
**********************************TGATGGGGAACAGGAGGACTCGCCGGC****
..................................TGATGGGGAACAGGAGGACTCGCCGGC....
................................................................
------------------------------------------------------------------------------------------
..................................TGATGGGGAACAGGAGGACTCGCCGGC....
................................................................
------------------------------------------------------------------------------------------
```

24 P7-1 +/-
24 P7-1 +/-
5 P10-1 +/-
5 P10-1 +/-

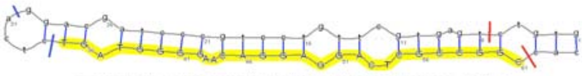

high confidence 196

```
TGGGTGAGGGGAGCTAGGTAGAAAGCCAGAGGCATATGTGGTTTTCACCTTCCTCCCAGCCCA
.((((...(((((..((((.((((((((.(......).))))))))))))..)))))).)))).
******AGGGGAGCTAGGTAGAAAGCCA************************************
......AGGGGAGCTAGGTAGAAAGCCA....................................
................................................................
------------------------------------------------------------------------------------------
```

5 P7-4 -/-
5 P7-4 -/-

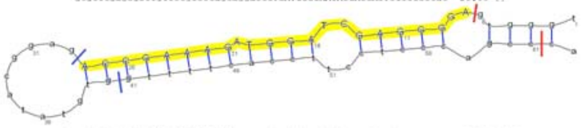

non-candidate 197

```
CTGAAGGACGTCTGCCTCCCTTGTTCTTGCTGAGAACAGGGGAACAGAGGGGCTTGTTTCAG
((((((.(.((((..(((((((((((((...))))))))))))).)))).)..))...)))))
******************************TGAGAACAGGGGAACAGAGGGGCTT*******
..............................TGAGAACAGGGGAACAGAGGGGCTT.......
..............................................................
------------------------------------------------------------------------------------------
..............................TGAGAACAGGGGAACAGAGGGGCTT.......
..............................................................
------------------------------------------------------------------------------------------
```

7 P7-1 +/-
7 P7-1 +/-
8 P10-2 +/-
8 P10-2 +/-

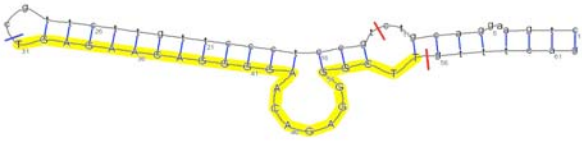

high confidence 198

```
GTGGGAAGTCTGACCTCTGCTGAGACATGCTGGGCATGGCAGCTGTGAGGTACCTCCCAC
((((((.((...(((((.((((...(((((...))))).))))...))))))).))))))
*TGGGAAGTCTGACCTCTGCTGAGACATG*******************************
.TGGGAAGTCTGACCTCTGCTGAGACAT................................
............................................................
------------------------------------------------------------------------------------------
.TGGGAAGTCTGACCTCTGCTGAGACATG...............................
............................................................
------------------------------------------------------------------------------------------
.TGGGAAGTCTGACCTCTGCTGAGACAT................................
............................................................
------------------------------------------------------------------------------------------
```

5 P7-1 +/-
5 P7-1 +/-
5 P10-1 +/-
5 P10-1 +/-
5 P10-2 +/-
5 P10-2 +/-

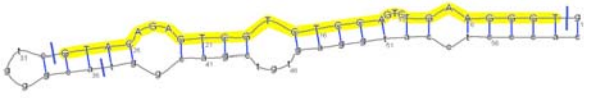

Supplement: Table S7 — Hairpin candidate evaluation. All testicular small RNA reads not identified as known miRNAs were analyzed for 1) their ability to form a stem loop structure, 2) minimum free energy less than -20 kcal/mol, 3) strong clean signal in the specific region of the 15–25 nt reference hairpin, 4) signals should not fall in the loop, 5) predicted Drosha and Dicer cut sites must be able to yield a mature miR sequence that matches the read, 6) stable 5′ end (±1 nt), 7) highly variable 3′ end, 8) presence of star sequence at lower copy number and matching miR with 3′ 2 nucleotide overhang, 9) does not map to rRNA, tRNA, snoRNA, or snRNA. Those matching all nine criteria were designated high-confidence novel miRNAs with star sequences. Those reads that passed all but criteria 6–8 were designated high-confidence miRNA without star sequence. Those that also did not pass criteria 5 were designated as potential miRNAs and the reads that failed criteria 1–4 were identified as non-candidates. Manually curated Dicer and Drosha cleavage sites on the hairpins were marked in blue and red lines. (PDF) [file pone.0015317.s007.pdf]
