# Supplementary material for: Analysis of MicroRNA Expression in the Prepubertal Testis
Source: PLoS One. 2010 Dec 29;5(12):e15317. doi: 10.1371/journal.pone.0015317 (PMC3012074; doi:10.1371/journal.pone.0015317)
Supplement: Table S8 — Description of novel testicular candidate miRNAs. Hairpin candidates were mapped to the mouse genome (mm9) using the UCSC Blat tool. Those that overlapped with other genomic elements were identified including repetitive elements (LINE, SINE, LTR). Conservation of the SEED sequence with syntenic sequences on the rat (R.n.) and human (H.s.) was assessed through the UCSC genome browser (Y, yes; N, no; M, mutated). (PDF) [file pone.0015317.s008.pdf]

Novel miRNA Candidates

	Mature sequence	Chr	Start	Stop	Str	Class	length	R.n. SEED	H.s. SEED	Location	Type of RNA	Gene (Gene Symbol)
1	UGUUACCUCUCGCAAGCUGUGAGCCUU	10	120849742	120849768	-	potential	27	Y	M	exon	mRNA	Ras association (RalGDS/AF-6) domain family member 3 (Rassf3)
2	UAAUAAAUGGCAUUGAGAAGGGAAGU	8	106878673	106878698	-	high confidence	26	M	M	exon	mRNA	CKLF-like MARVEL transmembrane domain containing 4 (Cmtm4)
3	UAACAGAGGUGGCUAAGAAUUGACGGA	17	36133218	36133244	+	non-candidate	27	M	N	intron	ncRNA	RIKEN cDNA A930015D03 gene (A930015D03Rik)
4	UGCAUGGUACUGUGUCAAGCAAGAUGU	15	20850719	20850745	+	potential	27	N	N	intergenic		LINE
4	UGCAUGGUACUGUGUCAAGCAAGAUGU	8	72286149	72286175	-	potential	27	Y	N	exon	mRNA	RIKEN cDNA D330038O06 gene (D330038O06Rik), LINE similarity
5	GGAUAUGAUGACUGAUUACCUGAGA	1	88248637	88248661	-	high confidence	25	Y	Y	intron	mRNA	nucleolin (Ncl)
6	UAGAAGAGUAGAAAGCGAUCAGGGUG	18	75592958	75592983	-	high confidence	26	M	M	exon	mRNA	predicted gene 672 (Gm672)
7	UUCAUGCAUCUUGGAGAACGGACACC	11	72217431	72217456	-	high confidence	26	Y	M	exon	mRNA	smoothelin-like 2 (Smtnl2)
8	UGGAAAGGACUCCGGAAAGGCCAAGAC	12	84961975	84962001	-	high confidence	27	N	N	intergenic		
8	UGGAAAGGACUCCGGAAAGGCCAAGAC	6	116123772	116123798	+	high confidence	27	N	N	intron	mRNA	transmembrane and coiled coil domains 1 (Tmcc1)
8	UGGAAAGGACUCCGGAAAGGCCAAGAC	3	137528111	137528137	+	high confidence	27	Y	Y	exon	mRNA	H2A histone family, member Z (H2afz)
9	UGAAUAAACAGAGUAGUAAUCGGAAGCCU	2	163980331	163980359	+	high confidence	29	M	M	exon	mRNA	serine/threonine kinase 4 (Stk4)
10	UACAGUAGUAGGCUGAGUCAGGGCGUC	11	105918017	105918043	+	non-candidate	27	M	M	exon	mRNA	DDB1 and CUL4 associated factor 7 (Dcaf7)
11	UUUGAUUUGUGUGAGCAAAACCGGCGCCUU	5	113783887	113783916	+	high confidence	30	Y	N	intergenic		
12	UUUGGAGUGUAGAAUGGGUGUUUUGU	10	60895112	60895137	-	potential	26	Y	M	intergenic		
13	UAGAAGAAGGCAGUAAAUGUACACGCA	17	27476263	27476289	+	non-candidate	27	Y	N	intergenic		LTR
14	UUGGAUUGGUUCUUGGUAAACUGAAGA	8	106877883	106877909	-	high confidence	27	Y	Y	intergenic		
15	UGAGCAGAUGUGGAAUAGGAGAAGGC	7	48296769	48296794	-	high confidence	26	Y	N	intron	mRNA	predicted gene 4884 (Gm4884)
15	UGAGCAGAUGUGGAAUAGGAGAAGGC	7	48411559	48411584	-	high confidence	26	Y	N	intergenic		
15	UGAGCAGAUGUGGAAUAGGAGAAGGC	7	48542163	48542188	-	high confidence	26	Y	N	exon	mRNA	predicted gene 5592 (Gm5592)
15	UGAGCAGAUGUGGAAUAGGAGAAGGC	7	46477859	46477884	+	high confidence	26	Y	N	intergenic		
15	UGAGCAGAUGUGGAAUAGGAGAAGGC	7	46621028	46621053	+	high confidence	26	Y	N	intergenic		
15	UGAGCAGAUGUGGAAUAGGAGAAGGC	7	46666051	46666076	+	high confidence	26	Y	N	exon	mRNA	predicted gene 5114 (Gm5114)
15	UGAGCAGAUGUGGAAUAGGAGAAGGC	7	39234704	39234729	+	high confidence	26	Y	N	intergenic		
15	UGAGCAGAUGUGGAAUAGGAGAAGGC	7	39355242	39355267	+	high confidence	26	Y	N	intergenic		
16	UAGAUAGAUAAAUAAAUGGAUACUGGAU	3	19799839	19799866	+	high confidence	28	N	N	intergenic		
17	UGUUCAAGGGGACGAUGAUUAUGUGA	1	95715748	95715773	+	non-candidate	26	Y	M	exon	mRNA	inhibitor of growth family, member 5 (Ing5)
18	UGGGGUUUGAUUGCUUCUGAUACGGGU	4	138451203	138451229	-	potential	27	M	M	intergenic		
19	GAGCACCCCAUUGGCUACCCACA	7	108030821	108030843	-	high confidence	23	Y	Y	intron	mRNA	(Arhgef17)
20	GAGCAGGCUGGCCUCGAACUC					high confidence	21					
21	UGCUUGUUAGUGUCUCUGGGCUAAGCAG	4	119300406	119300433	+	non-candidate	28	Y	M	exon		forkhead box J3 (Foxj3)
22	CACGGCUGUCUCUCCAGCCACCUUUUU	18	17002943	17002969	-	potential	27	N	N	intergenic		SINE
22	CACGGCUGUCUCUCCAGCCACCUUUUU	2	27257492	27257518	+	potential	27	N	N	intron	mRNA	vav 2 oncogene (Vav2), LTR
22	CACGGCUGUCUCUCCAGCCACCUUUUU	16	33047750	33047776	+	potential	27	N	N		mRNA	IQ motif containing G (Iqcg), SINE
22	CACGGCUGUCUCUCCAGCCACCUUUUU	6	128710095	128710121	-	potential	27	Y	N	intergenic		SINE
22	CACGGCUGUCUCUCCAGCCACCUUUUU	6	128748879	128748905	-	potential	27	Y	N	intergenic		SINE
22	CACGGCUGUCUCUCCAGCCACCUUUUU	6	128789140	128789166	+	potential	27	Y	N	intergenic		SINE
23	UUGGUCAUGAUGUUUGUGCAGGAAUA	2	41589326	41589351	-	high confidence	26	N	N	intron	mRNA	low density lipoprotein-related protein 1B (deleted in tumors) (Lrp1b), LTR
24	UGCCAACAUGCCUGUGGAGAGCGAGC	6	40372819	40372844	-	potential	26	Y	Y	intron	mRNA	RIKEN cDNA E330009J07 gene (E330009J07Rik)
25	UGAAGACUAAGAAGAUUGGUAAAGACUG	8	87598919	87598946	+	potential	28	M	M	exon	mRNA	deoxyhypusine synthase (Dhps)
25	UGAAGACUAAGAAGAUUGGUAAAGACUG					potential	28			exon/Intergenic	mRNA	WD repeat domain containing 83 (Wdr83)
26	UGGUAAAGAACACAUUGAAAGUACUGCA	X	158850826	158850853	-	non-candidate	28	Y	M	exon	mRNA	RALBP1 associated Eps domain containing protein 2 (Reps2)
27	UGUUCUUGGGGAGAAGGGUCGUCGGA	7	106630830	106630855	-	non-candidate	26	Y	M	exon	mRNA	ribosomal protein S3 (Rps3)
28	UGGAUGAGUACAGUGGUAGGCAGAUG	5	140277933	140277958	+	potential	26	N	N	intron	mRNA	v-maf musculoaponeurotic fibrosarcoma oncogene family, protein K (avian) (Mafk)
29	UUAUUCUUUGCAGUCUGGAUGUGCUU	1	120578456	120578481	+	non-candidate	26	N	N	exon	mRNA	transcription factor CP2-like 1 (Tcfcp2l1), snRNA
30	UCACUCUGUAGACGAGGCUGAC	4	150054118	150054139	-	high confidence	22	N	N	intergenic		SINE
30	UCACUCUGUAGACGAGGCUGAC	14	10535490	10535511	-	high confidence	22	N	N	intron	mRNA	fragile histidine triad gene (Fhit), SINE
30	UCACUCUGUAGACGAGGCUGAC	16	53627361	53627382	+	high confidence	22	N	N	intergenic		SINE
30	UCACUCUGUAGACGAGGCUGAC	13	55341940	55341961	+	high confidence	22	N	N	intron	mRNA	nuclear receptor-binding SET-domain protein 1 (Nsd1), SINE
30	UCACUCUGUAGACGAGGCUGAC	15	81081200	81081221	-	high confidence	22	Y	N	intergenic		SINE
31	UCUCUCAGCAGCUGGCAUCUGUACUU	1	34870956	34870981	-	high confidence	26	Y	M	exon	mRNA	family with sequence similarity 168, member B (Fam168b), transcript variant 2
32	UGGGUUCUACAAGAGAGCAGUCUGAGCA	2	177169180	177169207	-	high confidence	28	N	N	intergenic		LTR
32	UGGGUUCUACAAGAGAGCAGUCUGAGCA	2	177507800	177507827	+	high confidence	28	N	N	intergenic		LTR
33	UGAUCUUUUAGCUGGAGUCUGUACCCUG	14	24906769	24906796	+	potential	28	M	N	intergenic		
34	UACAAACACAGGUGGAGCUGGAGCAG	11	60369681	60369706	+	high confidence	26	M	M	intergenic		
35	UGAGAUUAUGGUACUGUGCAGCAAGC	2	163978012	163978037	+	high confidence	26	Y	M	exon	mRNA	serine/threonine kinase 4 (Stk4)
36	UGAGCGACCUGCAGCUGGACUACCUGG	12	13462461	13462487	-	high confidence	27	N	N	intron	mRNA	neuroblastoma amplified sequence (Nbas)
36	UGAGCGACCUGCAGCUGGACUACCUGG	11	40487043	40487069	-	high confidence	27	N	N	intergenic		
36	UGAGCGACCUGCAGCUGGACUACCUGG	1	55501553	55501579	-	high confidence	27	N	N	intron	mRNA	phospholipase C-like 1 (Plcl1)
36	UGAGCGACCUGCAGCUGGACUACCUGG	6	34261957	34261983	-	high confidence	27	Y	Y	exon	mRNA	aldo-keto reductase family 1, member B3 (aldose reductase) (Akr1b3)
36	UGAGCGACCUGCAGCUGGACUACCUGG					high confidence	27			exon	ncRNA	predicted gene 6644 (Gm6644)
37	UACUGGACAUAGCGUGGAAACCAGAGC	10	75503676	75503702	+	high confidence	27	M	N	exon	mRNA	zinc finger protein 280B (Zfp280b)
37	UACUGGACAUAGCGUGGAAACCAGAGC	X	5496810	5496836	-	high confidence	27	N	N	intergenic		
38	UGCGAGAGACUGCUGGGGACAGGCAU	2	26762102	26762127	-	high confidence	26	Y	Y	exon	mRNA	mediator complex subunit 22 (Med22), transcript variant 1
38	UGCGAGAGACUGCUGGGGACAGGCAU					high confidence	26	Y	Y	intron	mRNA	mediator complex subunit 22 (Med22), transcript variant 2
39	UGAAGGACAGGAUGGAUGGAAGUGA	1	95709039	95709063	+	high confidence	25	Y	Y	exon	mRNA	inhibitor of growth family, member 5 (Ing5),
40	UGGGGCUAAAGGAAGGAAGGAAAGGAU	6	128134016	128134042	-	high confidence	27	N	N	intergenic		
41	UACACUUCAAACAGUUCGGAUGCAGAGG	10	82824844	82824871	+	high confidence	28	M	M	intron	mRNA	DNA segment, Chr 10, Wayne State University 102, expressed (D10Wsu102e)
42	UAAGAGAGCAAGCUGAGCAAGACAGGA	6	71933932	71933958	+	potential	27	N	N	intergenic		LTR
43	UGAAAAUUCACUUGCAGACUGGACUGU	13	12709375	12709401	-	high confidence	27	M	N	intron	ncRNA	G protein-coupled receptor 137B, pseudogene (Gpr137b-ps)
43	UGAAAAUUCACUUGCAGACUGGACUGU	13	13452948	13452974	-	high confidence	27	M	N	intron	ncRNA	G protein-coupled receptor 137B (Gpr137b)
44	UAGGACAGCCAGCAGUAUGUAGAGAGACC	1	4780647	4780675	-	high confidence	29	Y	N	intergenic		
45	UCUGAAGAGAUGUCUGCUGCUCAGGGC	16	17260573	17260599	+	potential	27	Y	M	exon	mRNA	hypermethylated in cancer 2 (Hic2)
46	UUCAGAUGGAUGUGGAGGGAGGGAAG	11	97255938	97255963	+	potential	26	M	M	exon	mRNA	suppressor of cytokine signaling 7 (Socs7)

47	UGACAUCACUGAAGAUUCUUGGGCUCU	14	45560552	45560578	+	non-candidate	27	N	N	intergenic		
47	UGACAUCACUGAAGAUUCUUGGGCUCU	14	20459859	20459885	-	non-candidate	27	Y	N	intergenic		
48	UACAAAGCUCUGUGCAUGGCUCUUCUG	11	79510938	79510964	+	non-candidate	27	M	M	intergenic		
49	UGCAUGUGGAGGCUGGAGAUGUGUCA	8	72352999	72353024	+	non-candidate	26	N	N	exon	mRNA	lysophosphatidic acid receptor 2 (Lpar2)
50	UAUGUGCAUAGUGGAAAGUCAAGCUU	7	6581544	6581569	-	potential	26	N	N	intergenic		LTR
51	UUCAGACAGUGAAGGACAGACGGUGC	1	127136179	127136204	+	high confidence	26	N	M	exon	mRNA	transmembrane protein 194 (Tmem194), transcript variant 1
52	UGCUGUCAUCUGUGGCACAAGUGGGU	13	51246455	51246480	+	non-candidate	26	M	M	exon	mRNA	spindlin 1 (Spin1), transcript variant 1
52	UGCUGUCAUCUGUGGCACAAGUGGGU	11	8239558	8239583	-	non-candidate	26	N	N	intergenic		
53	UGUUUGUUUGUUGUACAGGUCCUGGGC	17	27487398	27487424	+	potential	27	M	N	intergenic		LTR
54	UUAAAGGUUUGACGUUUGGUGGGCAG	1	75503034	75503059	+	high confidence	26	M	N	exon	mRNA	zinc finger protein 280B (Zfp280b)
55	UGGAACUAGAAUUACAGGUUGUGAGCUGC	11	5683022	5683050	+	potential	29	Y	N	intron	ncRNA	RIKEN cDNA 2210015D19 gene (2210015D19Rik) SINE
56	UAGGAACUAUUGGGACAGAUUCAGGCU	1	77210313	77210339	+	high confidence	27	Y	M	exon	mRNA	haloacid dehalogenase-like hydrolase domain containing 2 (Hdhd2), transcript variant 1
57	UCUGAGAGGAGAGCUGGUCUGAGCAG	1	105919634	105919659	+	high confidence	26	Y	M	exon	mRNA	DDB1 and CUL4 associated factor 7 (Dcaf7)
58	UGAGGUCAUGGAACAGCUGGAUGCAGA	2	155899617	155899643	-	non-candidate	27	M	M	exon	mRNA	copine I (Cpne1), transcript variant 2
59	UCUGACCUCUCUGUAUGCUGCUGCACGG	2	31654657	31654684	+	non-candidate	28	Y	N	intron	mRNA	c-abl oncogene 1, receptor tyrosine kinase (Abl1), transcript variant 1
60	UGAGACUUAAGGAAAAGCAGCUGACUUA	1	30876816	30876843	+	high confidence	28	M	Y	exon	mRNA	zinc finger and BTB domain containing 44 (Zbtb44), transcript variant a
61	UCUCUAGGCACGAGGAGUCUCCAGGUU	17	66502266	66502292	-	potential	27	M	N	intergenic		
61	UCUCUAGGCACGAGGAGUCUCCAGGUU	17	66558591	66558617	+	potential	27	Y	N	intergenic		
62	UACUGGUUUUGAAGGUAUGACGGAAU	2	174992649	174992674	-	high confidence	26	N	N	intergenic		LTR
63	UGCUGUAAACCCUCAGGACGGUAGCAG	4	45409335	45409361	-	non-candidate	27	M	M	exon	mRNA	mitochondrial carrier triple repeat 1 (Mcart1), nuclear gene encoding mitochondrial protein
63	UGCUGUAAACCCUCAGGACGGUAGCAG	7	135302351	135302377	+	non-candidate	27	N	N	intergenic		
64	UGACUGAUAGAUGUUGGGACUUAGGCA	8	112254677	112254703	-	potential	27	Y	Y	exon	mRNA	ataxin 1-like (Atxn1l)
65	UGGUUUGAUGACAUGGCAGAUACAGA	10	129850258	129850283	+	high confidence	26	N	N	intergenic		
65	UGGUUUGAUGACAUGGCAGAUACAGA	10	126433432	126433457	+	high confidence	26	N	Y	exon	mRNA	CTD (carboxy-terminal domain, RNA polymerase II, polypeptide A) small phosphatase 2 (Ctdsp2), transcript variant a
66	UGAGAUGGGAGGUUUCUGGGAAAGGCA	11	97255148	97255174	+	potential	27	M	M	exon	mRNA	suppressor of cytokine signaling 7 (Socs7)
67	UAACUAGAGAACUGGUCAGGUCUGAGGU	2	92402706	92402733	+	high confidence	28	M	M	intergenic		
68	UAAGGAUGGAAUUAGAAAUAAUGUGAUGG	9	67585842	67585870	+	high confidence	29	M	N	intergenic		
69	UAAUACAAGGUGAACAGUAGACCAUU	1	120578955	120578980	+	high confidence	26	N	N	exon	mRNA	transcription factor CP2-like 1 (Tcfcp2l1)
70	UAAGCGCUGGAUUGGAGGAGGAGGA	7	6610919	6610943	-	high confidence	25	Y	N	intergenic		
71	GCUGGAGGAUGAAGUAAGGAGUGA	8	112363200	112363223	+	potential	24	Y	M	intron	mRNA	adaptor protein complex AP-1, gamma 1 subunit (Ap1g1)
72	UGAAAGUGGUAGAGCUUGCAUAUUUAAUC	16	31962155	31962183	-	high confidence	29	M	Y	exon	mRNA	SUMO/sentrin specific peptidase 5 (Senp5)
73	UUGGAUCUGGCACUGUGUUCGAGGC	15	72935119	72935143	-	potential	25	M	M	exon	mRNA	eukaryotic translation initiation factor 2C, 2 (Eif2c2)
74	UGACUGAUGGUCUCUCAGAUGGCUCA	11	60040841	60040866	-	high confidence	26	M	M	exon	mRNA	target of myb1-like 2 (chicken) (Tom1l2), transcript variant 2
75	UGAGUGACGAUGACAGUGAGGAGGAGC	9	21505640	21505666	+	potential	27	Y	Y	exon	mRNA	SWI/SNF related, matrix associated, actin dependent regulator of chromatin, subfamily a, member 4 (Smarca4), transcript variant 1
76	UGUGAGACGCUGAGCCAGUAGGGUACU	1	119734913	119734939	+	high confidence plus star	27	Y	M	intergenic		
77	UGCAGGAUGUACUGUAGACAGAUGAUGG	5	25195814	25195841	-	high confidence	28	N	N	exon	mRNA	X-ray repair complementing defective repair in Chinese hamster cells 2 (Xrcc2)
78	UAAAGGCAAGAAGUUGGGGACUGGGUU	5	150595127	150595153	-	high confidence	27	M	N	intergenic		
79	UGAAUUCUGAAGGGGAACCAAGCCGG	4	141033165	141033190	-	high confidence	26	M	M	exon	mRNA	SPEN homolog, transcriptional regulator (Drosophila) (Spen)
80	UACAAGGAGAUAAGAAUGGGUCAAUUA	7	14691434	14691460	-	potential	27	N	N	intergenic		LINE
80	UACAAGGAGAUAAGAAUGGGUCAAUUA	3	17736121	17736147	-	potential	27	N	N	intergenic		LINE
80	UACAAGGAGAUAAGAAUGGGUCAAUUA	13	65383446	65383472	+	potential	27	N	N	intron	mRNA	zinc finger protein 369 (Zfp369) LINE
80	UACAAGGAGAUAAGAAUGGGUCAAUUA	13	65400660	65400686	+	potential	27	N	N	intergenic		LINE
81	AAUUCUUAGGACAAGGGUAGAAGGCA	X	124798934	124798959	+	high confidence	26	N	N	intergenic		LTR
81	AAUUCUUAGGACAAGGGUAGAAGGCA	1	134886608	134886633	-	high confidence	26	Y	N	exon	mRNA	transformed mouse 3T3 cell double minute 4 (Mdm4)
82	UGACUUGUAAGAUGGUGAUCCUCAGC	14	20454819	20454844	-	potential	26	N	N	intergenic		LTR
82	UGACUUGUAAGAUGGUGAUCCUCAGC	Y	56811628	56811653	+	potential	26	N	N	intergenic		LTR
82	UGACUUGUAAGAUGGUGAUCCUCAGC	Y	12347907	12347932	+	potential	26	N	N	intergenic		LTR
82	UGACUUGUAAGAUGGUGAUCCUCAGC	X	5135673	5135698	+	potential	26	N	N	intergenic		LTR
83	UCCUAAAACUCGAAAUAGCACUCAGGCU	10	85221705	85221732	+	non-candidate	28	N	N	intergenic		
84	UGGUACUUCUGGCCAUCGGCAUGUUCUU	19	10280978	10281005	+	high confidence	28	Y	M	exon	mRNA	RIKEN cDNA 1810006K21 gene (1810006K21Rik), transcript variant 1
85	UAAAGUGAGUUUGGUUUUGAGGUGA	4	134970329	134970353	-	high confidence	25	Y	N	exon	mRNA	regulator of calcineurin 3 (Rcan3)
86	UGAGAGUUCUGUGUCUGUAUUGAUGGG	15	7144899	7144925	+	non-candidate	27	Y	M	exon	mRNA	leukemia inhibitory factor receptor (Lifr), transcript variant 1
87	UAGAACUCUGUGCACCUGGCUGGCAC	11	119007268	119007293	-	non-candidate	26	Y	M	exon	mRNA	TBC1 domain family, member 16 (Tbc1d16)
88	UGGGAUAGAAGGAAAUGCAGAUGAGGCA	8	94716597	94716624	+	high confidence	28	M	M	intergenic		
89	GUAAAGGCUGGGCUUAGACGUGG	1	186646345	186646367	-	non-candidate	23	Y	M	exon	mRNA	MOCO sulphurase C-terminal domain containing 2 (Mosc2)
89	GUAAAGGCUGGGCUUAGACGUGG					non-candidate	23					mmu-miR-1981
89	GUAAAGGCUGGGCUUAGACGUGG					non-candidate	23					mmu-miR-1971
90	GCCUUAGGUGAUUGGGUUUUGCGGAC	M	16008	16033	-	potential	26	M	N	intergenic		
90	GCCUUAGGUGAUUGGGUUUUGCGGAC	12	98299971	98299996	-	potential	26	N	N	intergenic		
91	ACCGGGGACUGCGGCUUUCAGU	7	133723020	133723041	-	high confidence	22	Y	M	intron	mRNA	ceroid lipofuscinosis, neuronal 3, juvenile (Batten, Spielmeyer-Vogt disease) (Cln3), transcript variant 1
92	UGAGUUCAAGUGGAUGUGUGUGGCC	1	167240541	167240565	+	high confidence	25	Y	Y	exon	mRNA	G protein-coupled receptor 161 (Gpr161)
93	UGUGACUGAUCUCCUGGACCGGUGC	10	129850068	129850092	+	potential	25	N	N	intergenic		
93	UGUGACUGAUCUCCUGGACCGGUGC	10	126433093	126433117	+	potential	25	N	Y	exon	mRNA	CTD (carboxy-terminal domain, RNA polymerase II, polypeptide A) small phosphatase 2 (Ctdsp2), transcript variant a
94	UGACUAGAAUCAUGGCAUGGGAGGCA	1	34870610	34870635	-	high confidence	26	Y	M	exon	mRNA	family with sequence similarity 168, member B (Fam168b), transcript variant 2
95	UGAUCAUAGAACUUCUGGGGAAGGUGCC	17	28728199	28728226	-	potential	28	Y	Y	exon	mRNA	serine/arginine-rich protein specific kinase 1 (Srpk1)
96	UGAGUUUGCUGUGUAGAGGAUGGCUCC	1	191695054	191695080	+	high confidence	27	M	N	exon	mRNA	protein tyrosine phosphatase, non-receptor type 14 (Ptpn14)
97	UAAGAUUGCGGAGGAGCGAGAAGGUA	11	69208832	69208857	-	high confidence	26	M	M			
97	UAAGAUUGCGGAGGAGCGAGAAGGUA	11	28239851	28239876	-	high confidence	26	N	N	intergenic		
98	UGUGGAAUCCAGACUGAGCAGACAGAGC	14	27916166	27916193	+	non-candidate	28	M	M	exon	mRNA	interleukin 17 receptor D (Il17rd)
99	UGAAUGUUCUGGCGGAUGCUCUCAAG	2	152011383	152011408	-	high confidence	26	N	N	intergenic		
99	UGAAUGUUCUGGCGGAUGCUCUCAAG	11	6073004	6073029	-	high confidence	26	N	N	intron	mRNA	NudC domain containing 3 (Nudcd3)
99	UGAAUGUUCUGGCGGAUGCUCUCAAG	11	6355873	6355898	-	high confidence	26	N	N	Intergenic		
99	UGAAUGUUCUGGCGGAUGCUCUCAAG	6	131563716	131563741	+	high confidence	26	N	N	Intergenic		

99	UGAAUGUUCUGGCGGAUGCUCUCAAG	5	137372523	137372548	+	high confidence	26	N	N	Intergenic		
99	UGAAUGUUCUGGCGGAUGCUCUCAAG	7	125258720	125258745	-	high confidence	26	Y	Y	exon	mRNA	ribosomal protein S15A (Rps15a)
100	UGGACAAGCUGAUUGAAGGAACACACCGCC	9	114416715	114416744	-	high confidence	30	M	M	intergenic		
101	UACCCUGGAGGGACUGCUGCAGAGGACU	11	70798481	70798508	-	high confidence	28	Y	N	exon	mRNA	DEAH (Asp-Glu-Ala-His) box polypeptide 33 (Dhx33)
102	UGUGACUUUGGUACUUGAGAAGCUGG	6	47821195	47821220	+	potential	26	M	N	intergenic		SINE
103	UGGGCUGUCAUAGUGGUGUGAUCUUC	6	128136274	128136299	-	potential	26	M	N	intergenic		Transposon
104	UGCUUGCUGGUCUGUUGUGAUGGAG	1	167254946	167254970	+	high confidence	25	Y	M	intergenic		
105	UUCUAUCCAGGACAGAAAAUGACAUA	15	59087405	59087430	-	potential	26	M	M	intergenic		
106	UGGCACAACUUGAGGACAUGGGCAUG	2	164132500	164132525	+	high confidence	26	N	N	intergenic		
106	UGGCACAACUUGAGGACAUGGGCAUG	2	120333227	120333252	-	high confidence	26	Y	Y	exon	mRNA	zinc finger protein 106 (Zfp106)
107	UGGAAAGACCUGAUCAUGAGUAGAAGC	7	86049491	86049517	+	high confidence	27	M	M	intron	mRNA	apoptosis enhancing nuclease (Aen), transcript variant 1
108	UUGGUUGAGUCCUAGAACGCAGGCC	17	27474712	27474736	+	non-candidate	25	M	N	intergenic		
109	UGGGACCCUGGUGAAAGUGAAGGACU	15	72931610	72931635	-	potential	26	Y	M	intergenic		
110	UAGUAGACUGGCUCUUGAUGGUUUGU	15	83186036	83186061	-	potential	26	Y	N	exon	ncRNA	RIKEN cDNA 1700001L05 gene (1700001L05Rik)
111	UGGGUGUCUUACCUGGGAUUUGUUUAC	11	28239686	28239712	-	high confidence	27	N	N	intergenic		
111	UGGGUGUCUUACCUGGGAUUUGUUUAC	11	69208667	69208693	-	high confidence	27	Y	M	exon	mRNA	cytochrome b5 domain containing 1 (Cyb5d1)
112	UGAGAAUGUAACUGGCUCGCUCAUCCC	14	24923943	24923969	+	high confidence	27	M	M	intergenic		
113	UACUGGACAUUUGCAGGACACUGGAGGA	6	120837486	120837513	+	potential	28	M	M	exon	mRNA	BCL2-like 13 (apoptosis facilitator) (Bcl2l13), nuclear gene encoding mitochondrial protein
114	UGAAGGUCACAGAACAGGCAGACUCAA	12	99649011	99649037	-	high confidence	27	M	M	intergenic		
115	UGGCCUUCUUCGUAGUACAUACUUUUCC	2	167309426	167309453	-	high confidence	28	M	M	exon	mRNA	spermatogenesis associated 2 (Spata2)
116	UGAAUUAAAAGAGAGACUGGGUUUAA	17	23954443	23954468	+	high confidence	26	Y	M	exon	mRNA	serine/arginine repetitive matrix 2 (Srrm2)
117	UCUGAGAAUCGAGUAGAGACAAGUUG	10	60226266	60226291	-	high confidence	26	Y	M	exon	mRNA	unc-5 homolog B (C. elegans) (Unc5b)
118	UAGUCGUCGUCGUCUUCUGGGAUGGA	10	75303672	75303697	+	high confidence	26	M	M	intergenic		
119	UCUUGUUUGAAUACGGGCAUCUUCUG	6	128398496	128398521	+	non-candidate	26	Y	N	intron	ncRNA	predicted gene 10069 (Gm10069), transcript variant 2
120	ACGCCCUUCCCCCCCUUCUUCA	15	84781975	84781996	-	high confidence	22	Y	Y	intron	mRNA	RIKEN cDNA 5031439G07 gene (5031439G07Rik)
121	UGAGCUGAAGAGAUUGCCUGCCUCCUU	4	115807742	115807768	-	non-candidate	27	Y	M	intergenic		
122	UGGUAUCUGAGAGCUAGAAUGACCCAUG	2	180759598	180759625	-	high confidence	28	Y	M	intron	mRNA	cholinergic receptor, nicotinic, alpha polypeptide 4 (Chrna4)
123	UAGGACUCAGGAGUGCUUGGAGUGGU	1	158577746	158577771	+	high confidence	26	N	N	exon	mRNA	v-abl Abelson murine leukemia viral oncogene homolog 2 (arg, Abelson-related gene) (Abl2), transcript variant 2
124	UAGGAUCCCCGGAAGUGCAAAGAAGGC	11	60591401	60591427	+	potential	27	Y	M	exon	mRNA	Smith-Magenis syndrome chromosome region, candidate 8 homolog (human) (Smcr8), transcript variant 1
125	UUUGAGGAACUCAGCAGAUGUUUGUG	15	66936224	66936249	-	high confidence	26	Y	N	exon	mRNA	ST3 beta-galactoside alpha-2,3-sialyltransferase 1 (St3gal1)
126	UGUUUCCGUGUUGGCAGAUCUGGAG	18	33362063	33362087	-	non-candidate	25	M	M	exon	mRNA	StAR-related lipid transfer (START) domain containing 4 (Stard4)
127	UUGGACCUAGUUGACCUUGCAGGGCC	15	74478391	74478416	+	potential	26	Y	N	intergenic		
128	UGGCCUCUGUGGGCUCUAAAGAAAGGGU	8	119734798	119734825	+	non-candidate	28	M	M	intergenic		
129	UGAGAGGCAGGCUUCAGAUCUUUUGGU	9	44102003	44102029	-	high confidence	27	Y	N	intergenic		SINE
130	UGAGAUUUGUUCAUGAUCUUCUGACUU	15	59091457	59091483	-	high confidence	27	M	M	intergenic		LTR
131	UAGACUGUUUUGUGCUCUCCUUAAGACU	11	60370988	60371015	+	high confidence plus star	28	Y	N	exon	mRNA	alkB, alkylation repair homolog 5 (E. coli) (Alkbh5)
132	UCCUGUACUGACUGUAAUGCAUAUUAGCC	11	95823266	95823294	-	potential	29	Y	Y	exon	mRNA	insulin-like growth factor 2 mRNA binding protein 1 (Igf2bp1)
133	UGAUAGAGAGCAGAAUUCACCGAUUG	7	123475940	123475965	-	high confidence	26	M	M	exon	mRNA	ribosomal protein S13 (Rps13)
133	UGAUAGAGAGCAGAAUUCACCGAUUG	8	122126151	122126176	-	high confidence	26	N	N	intron	mRNA	TATA box binding protein (Tbp)-associated factor, RNA polymerase I, C (Taf1c
133	UGAUAGAGAGCAGAAUUCACCGAUUG	7	95679034	95679059	-	high confidence	26	N	N	intergenic		
133	UGAUAGAGAGCAGAAUUCACCGAUUG	8	89571968	89571993	+	high confidence	26	N	N	intergenic		
134	UGGAACUCACCCCAGGUAGCAGUCUGA	6	92137382	92137408	-	potential	27	M	M	intron	mRNA	zinc finger, FYVE domain containing 20 (Zfyve20)
135	UGUAAGGGACUGGAGAGAUGGCU	17	37133722	37133744	-	high confidence	23	Y	N	intergenic		SINE
136	UGAACAUGAGCUGGAGCAGUGAGGUC	5	38591872	38591897	-	potential	26	M	Y	exon	mRNA	zinc finger and BTB domain containing 49 (Zbtb49)
137	UGAGAUGGGAAAAGGAGGUAGCGUUA	15	78493609	78493634	-	high confidence	26	M	M	intergenic		
138	UGUUGAUCGGCGUUCUUGGUUAUG	7	6594138	6594161	-	potential	24	N	N	intergenic		
139	UGGGUAGACUGAGCCUGGCUGA	4	133904334	133904355	+	high confidence	22	Y	M	intron	mRNA	solute carrier family 30 (zinc transporter), member 2 (Slc30a2)
140	UGUUUACUCUGGACUGCAGCAUAGACC	11	59270213	59270239	+	high confidence	27	M	N	exon	mRNA	jumonji domain containing 4 (Jmjd4)
141	UAAUUCUAGGCUGAGGCAGGUGGAUC	2	39271345	39271370	-	high confidence	26	N	N	intergenic		SINE
141	UAAUUCUAGGCUGAGGCAGGUGGAUC	12	37104097	37104122	-	high confidence	26	N	N	intergenic		SINE
141	UAAUUCUAGGCUGAGGCAGGUGGAUC	3	92447656	92447681	+	high confidence	26	N	N	intergenic		SINE
141	UAAUUCUAGGCUGAGGCAGGUGGAUC	2	156215243	156215268	-	high confidence	26	Y	N	exon	ncRNA	RIKEN cDNA 2900097C17 gene (2900097C17Rik)
142	UGGUCAGAUGUUUUAGGUUGGGAAGA	2	33308031	33308056	-	potential	26	M	M	exon	mRNA	zinc finger and BTB domain containing 43 (Zbtb43), transcript variant 2
143	UAGAUGUGAUAACGCUCCCAGAGGCUGG	1	133924908	133924935	+	high confidence plus star	28	M	M	intron	mRNA	major facilitator superfamily domain containing 4 (Mfsd4), transcript variant a
144	UAGGAUCUUUGAGUUUCUUGCGGUUU	7	71033578	71033603	-	potential	26	Y	M	exon	mRNA	Kruppel-like factor 13 (Klf13)
145	UGAGGAUAAUGCAUGUAUACAGUAAUU	2	92433180	92433206	+	high confidence	27	M	M	intergenic		
146	UCACUUUGUAGAACAGGCU	18	55030866	55030888	+	high confidence	19	N	N	intergenic		SINE
147	UAGAUUGUAGGCCCAUUGGAAGG					non-candidate	23			No Match		
148	AACUGAGUUGAAGGCAAAGGU	15	40862391	40862411	+	high confidence	21	M	M	intron	mRNA	zinc finger protein, multitype 2 (Zfpm2)
149	UGGACACUGGAGAGAGAGCUUUU	4	58453895	58453917	-	high confidence	23	M	Y	intron	mRNA	lysophosphatidic acid receptor 1 (Lpar1), transcript variant 1
150	UAGAUUGUAAGCUCAUGAGGGCAGAAACC	11	69204515	69204543	-	non-candidate	29	M	M	intergenic		LINE
150	UAGAUUGUAAGCUCAUGAGGGCAGAAACC	11	28236498	28236526	-	non-candidate	29	N	N	intergenic		LINE
151	UCUGGGUUCAUGCAGUGUAGUCUCUUU	4	61881864	61881890	-	high confidence	27	M	N	intergenic		
152	UCUAAAUGUGCAACUCUUGGGAGAGCUU	17	27471454	27471481	+	high confidence	28	M	M	intergenic		
153	UGUGGCACUGGAAAAGAUGAGGCUAU	11	60598019	60598044	+	potential	26	M	M	exon	mRNA	Smith-Magenis syndrome chromosome region, candidate 8 homolog (human) (Smcr8), transcript variant 1
154	CUGGCUGAACUAUCUCAGAGACUUU	1	157929636	157929660	+	potential	25	M	M	intron	mRNA	family with sequence similarity 163, member A (Fam163a)
155	AGAAGGGCAAAAGCUCGCUUGAUCUUGA	X	70271990	70272017	+	high confidence	28	M	N	intron	mRNA	zinc finger protein 185 (Zfp185), transcript variant 2
155	AGAAGGGCAAAAGCUCGCUUGAUCUUGA	6	49186560	49186587	-	high confidence	28	N	N	intergenic	rRNA	
155	AGAAGGGCAAAAGCUCGCUUGAUCUUGA	9	123370959	123370986	+	high confidence	28	N	N	exon	mRNA	leucyl-tRNA synthetase, mitochondrial (Lars2), nuclear gene encoding mitochondrial protein
155	AGAAGGGCAAAAGCUCGCUUGAUCUUGA	12	79451966	79451993	+	high confidence	28	N	N	intron	mRNA	gephyrin (Gphn), transcript variant 2
156	GAGCAGGCUGGCCUCGAACU	1	167121331	167121357	-	potential	20	M	N	intron	mRNA	SFT2 domain containing 2 (Sft2d2) SINE
157	UUGGUGAUGAACAGCAAGCAGUAUGAAAGU	2	177169056	177169085	-	potential	30	N	N	intergenic		LTR
157	UUGGUGAUGAACAGCAAGCAGUAUGAAAGU	2	177096391	177096420	-	potential	30	N	N	intergenic		LTR
157	UUGGUGAUGAACAGCAAGCAGUAUGAAAGU	2	176698905	176698934	-	potential	30	N	N	intergenic		LTR

157	UUGGUGAUGAACAGCAAGCAGUAUGAAAGU	2	175454561	175454590	-	potential	30	N	N	intergenic		LTR
157	UUGGUGAUGAACAGCAAGCAGUAUGAAAGU	2	177507922	177507951	+	potential	30	N	N	intergenic		LTR
157	UUGGUGAUGAACAGCAAGCAGUAUGAAAGU	2	174789279	174789308	+	potential	30	N	N	intergenic		LTR
158	GGCCAAGGAUGAGAACUCUAACCUGAC	5	23216852	23216878	-	high confidence	27	Y	N	exon	ncRNA	expressed sequence AI506816 (AI506816)
159	UGGAACUAGAGCACUGCGGAUGUGAUC	11	60601516	60601542	+	high confidence	27	M	M	exon	mRNA	Smith-Magenis syndrome chromosome region, candidate 8 homolog (human) (Smcr8), transcript variant 1
160	UGGUAGUCUCUGUGGAAAGAGCUGGC	18	65792365	65792390	+	high confidence	26	Y	M	intron	mRNA	zinc finger protein 532 (Zfp532)
161	UAGAGAACCUGAAGCAUGAACUGGCC	5	135907570	135907595	-	high confidence	26	Y	M	exon	mRNA	huntingtin interacting protein 1 (Hip1)
162	UGGUCUACAGAGUGAGUUCCAGGACA					non-candidate	26			No Match		
163	UGGGAAGCAGAGGCAGGUGAGUU	5	36796598	36796620	-	potential	23	M	N	intergenic		SINE
163	UGGGAAGCAGAGGCAGGUGAGUU	8	86354809	86354831	+	potential	23	N	N	intergenic		SINE
164	UGAGACUCAGGACAGCUUGCAGACCUC	2	25702331	25702357	-	potential	27	M	M	exon	mRNA	spermatogenesis and oogenesis specific basic helix-loop-helix 1 (Sohlh1)
165	UGCAACUAGACGCUGAGGACAGAGGGC	2	32088489	32088515	+	high confidence	27	Y	M	exon	mRNA	HLA-B associated transcript 2-like (Bat2l), transcript variant 2
166	UUCCAUUCUGAAGGUGUCGUGUGUU	4	101137643	101137667	+	high confidence	25	Y	M	exon	mRNA	adenylate kinase 3-like 1 (Ak3l1), nuclear gene encoding mitochondrial protein
167	UGGCAGGAGCUGUGUACAAGAGGAGCC	4	126016998	126017024	-	non-candidate	27	Y	M	intergenic		
168	GUGAGGAGAGGUGGAAGUAUGGAAGG	X	20273594	20273619	+	high confidence	26	Y	M	intron	mRNA	cyclin-dependent kinase 16 (Cdk16)
169	UGCAAGAACGUUGACGUGGAGCGGUC	6	28371332	28371357	-	potential	26	M	M	exon	mRNA	golgi coiled coil 1 (Gcc1)
170	UGUGCUGUAAUACUGGGAGGCAAAGGA	11	75390798	75390824	+	non-candidate	27	Y	M	intergenic		
171	UUGGUGAUGAACAGCAGUAUGGAAGGGUA	9	103087934	103087962	-	potential	29	N	N	intergenic		LTR
171	UUGGUGAUGAACAGCAGUAUGGAAGGGUA	9	94413229	94413257	-	potential	29	N	N	intergenic		LTR
171	UUGGUGAUGAACAGCAGUAUGGAAGGGUA	16	72185919	72185947	-	potential	29	N	N	intergenic		LTR
171	UUGGUGAUGAACAGCAGUAUGGAAGGGUA	3	72616258	72616286	+	potential	29	N	N	intergenic		LTR
171	UUGGUGAUGAACAGCAGUAUGGAAGGGUA	17	93157323	93157351	+	potential	29	N	N	intergenic		LTR
171	UUGGUGAUGAACAGCAGUAUGGAAGGGUA	13	11255396	11255424	+	potential	29	N	N	intergenic		LTR
172	UCGCUCUGUAGACCAGGCUAUC	19	6240651	6240671	-	high confidence	22	Y	M	intergenic		SINE
173	UAGGCCUUCUGAAGCGUAGGAAAGGUU	1	136654781	136654807	-	high confidence	27	M	M	intergenic		protein phosphatase 1, regulatory (inhibitor) subunit 12B (Ppp1r12b)
174	UGCAUUCCUGGGACAUGUGACAGCAGC	10	60896036	60896062	-	high confidence	27	M	M	exon	mRNA	eukaryotic translation initiation factor 4E binding protein 2 (Eif4ebp2)
175	UGUGAACUACGAUGCUGAAUGAGUCA	7	80264650	80264675	+	high confidence	26	M	N	intergenic		SINE
176	UUGCAGGGAAGGUGCACAGAAGUCUGC	7	6577615	6577641	-	high confidence	27	N	N	intergenic		LINE
176	UUGCAGGGAAGGUGCACAGAAGUCUGC	14	108191759	108191785	-	high confidence	27	N	N	intergenic		LINE
177	UCCUGACUUGUAGGGAACGCAGAGA	14	24914718	24914742	+	high confidence	25	M	N	intergenic		
178	UUCACUUUGUAGAGCAGGCUGG	4	92132716	92132737	-	potential	22	N	N	intergenic		SINE
178	UUCACUUUGUAGAGCAGGCUGG	17	71873925	71873946	-	potential	22	N	N	intron	mRNA	NDC80 homolog, kinetochore complex component (S. cerevisiae) (Ndc80) SINE
178	UUCACUUUGUAGAGCAGGCUGG	9	90875686	90875707	+	potential	22	N	N	intergenic		SINE
178	UUCACUUUGUAGAGCAGGCUGG	6	132325485	132325506	+	potential	22	N	N	intergenic		SINE
178	UUCACUUUGUAGAGCAGGCUGG	18	77823831	77823852	+	potential	22	N	N	intergenic		SINE
178	UUCACUUUGUAGAGCAGGCUGG	17	44298061	44298082	+	potential	22	N	N	intergenic		SINE
178	UUCACUUUGUAGAGCAGGCUGG	3	121847510	121847531	-	potential	22	Y	N	intron	mRNA	ATP-binding cassette, sub-family A (ABC1), member 4 (Abca4) SINE
178	UUCACUUUGUAGAGCAGGCUGG	9	43485813	43485834	+	potential	22	Y	N	intergenic		SINE
179	UAGUAAAGAAGAGAUUUGGAGAAGGU	9	67543485	67543510	-	potential	26	Y	N	intergenic		
180	UACCUGAGCUGUAAGGAGGAGACGAAC	12	3976823	3976849	-	non-candidate	27	M	M	exon	mRNA	EFR3 homolog B (S. cerevisiae) (Efr3b)
181	UCCAAGGCAGGAGAUAUACUCAGGAC	8	72353146	72353171	+	non-candidate	26	M	N	exon	mRNA	lysophosphatidic acid receptor 2 (Lpar2)
182	UAUAGGAGGACCAGGAGAGGACUAUAGU	12	88033485	88033512	+	potential	28	M	M	exon	mRNA	vasohibin 1 (Vash1)
183	UCUGAACUCAUCUGGAGGCCUGACAUC	2	180768639	180768665	-	high confidence	27	Y	Y	exon	mRNA	cholinergic receptor, nicotinic, alpha polypeptide 4 (Chrna4)
184	UAUAUGUGUGGAUGGUAAUAAUGCUGAGU	4	41150099	41150127	-	non-candidate	29	Y	Y	intron	mRNA	ubiquitin-associated protein 2 (Ubap2)
185	UAGGCUAGAGAGAGGUUGGGGA	7	6756349	6756370	+	high confidence	22	Y	N	intron	mRNA	ubiquitin specific peptidase 29 (Usp29)
186	CUUAGAUCGAUGUGGUGCU	12	37542836	37542855	+	potential	19			intergenic		
187	UCUGAAAGGGCACAGACCAUGGGCGU	9	63496617	63496642	-	high confidence	26	M	M	exon	mRNA	MAD homolog 3 (Drosophila) (Smad3)
188	UGAUGAAGACUGGCACCUAUUCUGUG	3	87725937	87725962	+	high confidence	26	Y	Y	exon	mRNA	mitochondrial ribosomal protein L24 (Mrpl24), nuclear gene encoding mitochondrial protein
189	UCUGACAGGAUUUUCAGGCUUUUUCCAGA	10	82847050	82847078	+	high confidence	29	N	N	intergenic		LTR
190	UGGCGGCAGUCAGGAUACCUGU	11	117863593	117863614	+	high confidence	22	M	M	intron	mRNA	phosphatidylglycerophosphate synthase 1 (Pgs1)
191	UACUCUUUCUGUGUGUCCGAGGCUUC	8	119735479	119735504	+	high confidence	26	M	N	intergenic		
192	UAGGAGUUGUGCAGUGAAGAGGAUGG	12	37103490	37103515	-	non-candidate	26	N	N	intergenic		
192	UAGGAGUUGUGCAGUGAAGAGGAUGG	2	156214636	156214661	-	non-candidate	26	Y	M	exon	ncRNA	RIKEN cDNA 2900097C17 gene (2900097C17Rik)
193	UCCUGGCACUGUGGUGGGACUGGUU	9	119349051	119349075	+	high confidence	25	M	M	intergenic		
194	UGACAUAGCUGAGCACUCAAAGAUGG	9	3156891	3156916	+	high confidence	26	N	N	intergenic		
194	UGACAUAGCUGAGCACUCAAAGAUGG	6	128132922	128132947	-	high confidence	26	Y	N	intergenic		
195	UGAUGGGGAACAGGAGGACUCGCCGGC	11	69209684	69209710	+	potential	27	Y	M	exon	mRNA	LSM domain containing 1 (Lsmd1)
196	AGGGGAGCUAGGUAGAAAGCCA	19	4623929	4623950	-	high confidence	22	Y	M	intron	mRNA	RCE1 homolog, prenyl protein peptidase (S. cerevisiae) (Rce1)
197	UGAGAACAGGGGAACAGAGGGGCUU	15	102044485	102044509	+	non-candidate	25	Y	Y	exon	mRNA	zinc finger protein 740 (Zfp740)
198	UGGGAAGUCUGACCUCUGCUGAGACAUG	9	43952272	43952299	-	high confidence	28	M	M	intergenic		
