# Supplementary material for: Analysis of MicroRNA Expression in the Prepubertal Testis
Source: PLoS One. 2010 Dec 29;5(12):e15317. doi: 10.1371/journal.pone.0015317 (PMC3012074; doi:10.1371/journal.pone.0015317)
Supplement: Table S10 — Comparison of predominance of 5p vs. 3p between P14 and adult testes. The expression of 5p versus 3p mature miRNAs were compared between this study and adult testes. Those miRNAs which shifted from a high to low 5p/3p ratio or vice-versa are shown with the abundant mature miRNA at each time-point. (PDF) [file pone.0015317.s010.pdf]

miRNAs shifting strand preference between P14 and adult testis

miRNA	P14	Adult
mmu-mir-1-2	5p	3p
mmu-mir-17	3p	5p
mmu-mir-19b-1	5p	3p
mmu-mir-19b-2	5p	3p
mmu-mir-23a	5p	3p
mmu-mir-29b-2	5p	3p
mmu-mir-30b	3p	5p
mmu-mir-30c-2	3p	5p
mmu-mir-25	5p	3p
mmu-mir-28	3p	5p
mmu-mir-92a-1	5p	3p
mmu-mir-92b	5p	3p
mmu-mir-101b	5p	3p
mmu-mir-103-1	5p	3p
mmu-mir-103-2	5p	3p
mmu-mir-107	5p	3p
mmu-mir-126	5p	3p
mmu-mir-128-1	5p	3p
mmu-mir-128-2	5p	3p
mmu-mir-130a	5p	3p
mmu-mir-133a-2	5p	3p
mmu-mir-133b	5p	3p
mmu-mir-139	3p	5p
mmu-mir-140	3p	5p
mmu-mir-143	5p	3p
mmu-mir-144	5p	3p
mmu-mir-145	5p	3p
mmu-mir-147	5p	3p
mmu-mir-148b	5p	3p
mmu-mir-150	3p	5p
mmu-mir-151	3p	5p
mmu-mir-152	5p	3p
mmu-mir-153	5p	3p
mmu-mir-154	5p	3p
mmu-mir-193	3p	5p
mmu-mir-202	5p	3p
mmu-mir-206	5p	3p
mmu-mir-210	5p	3p
mmu-mir-218-2	3p	5p
mmu-mir-219-2	5p	3p
mmu-mir-221	5p	3p
mmu-mir-222	5p	3p
mmu-mir-223	5p	3p
mmu-mir-297a-3	3p	5p
mmu-mir-297a-4	3p	5p
mmu-mir-297c	3p	5p
mmu-mir-301a	5p	3p
mmu-mir-320	5p	3p
mmu-mir-322	3p	5p
mmu-mir-326	5p	3p
mmu-mir-328	5p	3p
mmu-mir-330	3p	5p

miRNAs shifting strand preference between P14 and adult testis

mmu-mir-341	5p	3p
mmu-mir-344-1	5p	3p
mmu-mir-344-2	5p	3p
mmu-mir-345	3p	5p
mmu-mir-350	5p	3p
mmu-mir-365-1	5p	3p
mmu-mir-365-2	5p	3p
mmu-mir-369	5p	3p
mmu-mir-370	5p	3p
mmu-mir-375	5p	3p
mmu-mir-376b	3p	5p
mmu-mir-377	5p	3p
mmu-mir-381	5p	3p
mmu-mir-382	5p	3p
mmu-mir-410	5p	3p
mmu-mir-429	5p	3p
mmu-mir-455	3p	5p
mmu-mir-463	5p	3p
mmu-mir-465a	3p	5p
mmu-mir-465c-1	5p	3p
mmu-mir-465c-2	5p	3p
mmu-mir-466c	5p	3p
mmu-mir-466d	5p	3p
mmu-mir-466f-3	5p	3p
mmu-mir-467b	3p	5p
mmu-mir-483	5p	3p
mmu-mir-485	5p	3p
mmu-mir-487b	5p	3p
mmu-mir-488	5p	3p
mmu-mir-494	5p	3p
mmu-mir-495	5p	3p
mmu-mir-500	5p	3p
mmu-mir-511	5p	3p
mmu-mir-540	5p	3p
mmu-mir-542	3p	5p
mmu-mir-543	5p	3p
mmu-mir-547	5p	3p
mmu-mir-582	3p	5p
mmu-mir-598	5p	3p
mmu-mir-615	5p	3p
mmu-mir-652	5p	3p
mmu-mir-665	5p	3p
mmu-mir-666	5p	3p
mmu-mir-667	5p	3p
mmu-mir-668	5p	3p
mmu-mir-669f	5p	3p
mmu-mir-674	5p	3p
mmu-mir-741	5p	3p
mmu-mir-743a	5p	3p
mmu-mir-871	5p	3p
mmu-mir-878	5p	3p
mmu-mir-880	5p	3p
