# Supplementary material for: Analysis of MicroRNA Expression in the Prepubertal Testis
Source: PLoS One. 2010 Dec 29;5(12):e15317. doi: 10.1371/journal.pone.0015317 (PMC3012074; doi:10.1371/journal.pone.0015317)
Supplement: Checklist S1 — The ARRIVE Checklist. (DOC) [file pone.0015317.s011.doc]

**The ARRIVE Checklist**

**TITLE**

**1 Provide as accurate and concise a description of the content of the article as possible.**

Analysis of MicroRNA Expression in the Prepubertal Testis

**ABSTRACT**

**2 Provide an accurate summary of the background, research objectives (including details of the species or strain of animal used), key methods, principal findings, and conclusions of the study.**

Only thirteen microRNAs are conserved between *D. melanogaster* and the mouse; however, conditional loss of miRNA function through mutation of Dicer causes defects in proliferation of premeiotic germ cells in both species. This highlights the potentially important, but uncharacterized, role of miRNAs during early spermatogenesis. The goal of this study was to characterize on postnatal day 7, 10, and 14 the content and editing of murine testicular miRNAs, which predominantly arise from spermatogonia and spermatocytes, in contrast to prior descriptions of miRNAs in the adult mouse testis which largely reflects the content of spermatids. Previous studies have shown miRNAs to be abundant in the mouse testis by postnatal day 14; however, through Next Generation Sequencing of testes from a B6;129 background we found abundant earlier expression of miRNAs and describe shifts in the miRNA signature during this period. We detected robust expression of miRNAs encoded on the X chromosome in postnatal day 14 testes, consistent with prior studies showing their resistance to meiotic sex chromosome inactivation. Unexpectedly, we also found a similar positional enrichment for most miRNAs on chromosome 2 at postnatal day 14 and for those on chromosome 12 at postnatal day 7. We quantified *in vivo* developmental changes in three types of miRNA variation including 5’ heterogeneity, editing, and 3’ nucleotide addition. We identified eleven putative novel pubertal testis miRNAs whose developmental expression suggests a possible role in early male germ cell development. These studies provide a foundation for interpretation of miRNA changes associated with testicular pathology and identification of novel components of the miRNA editing machinery in the testis.

**INTRODUCTION**

**Background**

**3 a. Include sufficient scientific background (including relevant references to previous work) to understand the motivation and context for the study, and explain the experimental approach and rationale.**

**b. Explain how and why the animal species and model being used can address the scientific objectives and, where appropriate, the study’s relevance to human biology.**

We selected the mouse model because multiple studies of mammalian small RNAs have been performed in the mouse testis, allowing a productive comparison to pubertal spermatogenesis. There are a significant number of genetic mutants in the miRNA and piRNA pathways uniquely in the mouse. In particular, we utilized GASZ null mice lacking piRNAs and displaying elevated miRNA reads to evaluate the likelihood of potential novel miRNAs. Due to the limited accessibility of human pubertal testicular material, studies in model organisms are necessary to develop an understanding of pubertal spermatogenesis in humans. We expect that a description of the normal pubertal testicular miRNAome will provide the necessary background to evaluate the mechanistic role of miRNA alterations to human infertility involving early blocks to spermatogenesis or testicular cancer. Alteration of some miRNAs in these pathologic states might alternatively result from an arrest of developmental progression and our studies could potentially aid distinguishing these from therapeutic targets.

**Objectives**

**4 Clearly describe the primary and any secondary objectives of the study, or specific hypotheses being tested.**

- To comprehensively determine the miRNAs and their edited isoforms in the pubertal mouse testis and identify systems level patterns to their expression.
- To contrast the miRNA complement and editing during pubertal spermatogenesis to published studies in the adult testis.
- To determine the developmental timing of several distinct types of miRNA editing *in vivo* in order to assess whether these processes may be developmentally regulated or coordinated with each other.
- To identify novel testicular miRNAs, and evaluate their potential for contraceptive targeting or contribution to human male infertility.
- To determine what developmental trends in miRNAs or their processing are consistent with their potential involvement in the translational control of the many germ cell expressed mRNAs associated with nuage RNA granules.

**METHODS**

**Ethical statement**

**5 Indicate the nature of the ethical review permissions, relevant licenses (e.g. Animal [Scientific Procedures] Act 1986), and national or institutional guidelines for the care and use of animals, that cover the research.**

These studies were carried out in accordance with the NIH Guide for the Care and Use of Laboratory Animals under Baylor College of Medicine IACUC approved protocol AN-716.

**Study design**

**6 For each experiment, give brief details of the study design, including:**

**a. The number of experimental and control groups.**

**b. Any steps taken to minimise the effects of subjective bias when allocating animals to treatment (e.g., randomisation procedure) and when assessing results (e.g., if done, describe who was blinded and when).**

**c. The experimental unit (e.g. a single animal, group, or cage of animals).**

**A time-line diagram or flow chart can be useful to illustrate how complex study designs were carried out.**

a. Two GASZ^+/-^ mice from two different litters were collected with litter-matched GASZ null controls.

b-c. The small RNA complement of each animal was individually sequenced and the number of corresponding reads across both animals were summed to reduce inter-animal variation. Due to the low copy number of many of the novel miRNAs, these were assessed from individual animals.

**Experimental procedures**

**7 For each experiment and each experimental group, including controls, provide precise details of all procedures carried out. For example:**

**a. How (e.g., drug formulation and dose, site and route of administration, anaesthesia and analgesia used [including monitoring], surgical procedure, method of euthanasia). Provide details of any specialist equipment used, including supplier(s).**

**b. When (e.g., time of day).**

**c. Where (e.g., home cage, laboratory, water maze).**

**d. Why (e.g., rationale for choice of specific anaesthetic, route of administration, drug dose used).**

All mice were sacrificed at 6pm under inhaled anaesthetic isofluorane for five minutes followed by cervical dislocation as per approved animal protocol. Testes were frozen in liquid nitrogen until RNA isolation procedure was performed.

**Experimental animals**

**8 a. Provide details of the animals used, including species, strain, sex, developmental stage (e.g., mean or median age plus age range), and weight (e.g., mean or median weight plus weight range).**

**b. Provide further relevant information such as the source of animals, international strain nomenclature, genetic modification status (e.g. knock-out or transgenic), genotype, health/immune status, drug- or test naıve, previous procedures, etc.**

Testes were collected from *M. musculus* heterozygous or homozygous for mutant Gasz allele, *Gasz^tm1Zuk^*, at postnatal day 7, 10, and 14. Mice were generated from a mating colony by intercrossing heterozygous sires and homozygous dams of B6;129 mixed background (129S7/AB2.2 × C57BL6/J).

**Housing and husbandry**

**9 Provide details of:**

**a. Housing (e.g., type of facility, e.g., specific pathogen free (SPF); type of cage or housing; bedding material; number of cage companions; tank shape and material etc. for fish).**

**b. Husbandry conditions (e.g., breeding programme, light/dark cycle, temperature, quality of water etc. for fish, type of food, access to food and water, environmental enrichment).**

**c. Welfare-related assessments and interventions that were carried out before, during, or after the experiment.**

1. Housing- Barrier (SPF):type of cage- ventilated (microisolator); bedding material-1/4 corn cob; number of cage companion 590 cages
2. Husbandry condition.; light/dark cycle (12hrs/12hrs) 7am-7pm; temperature 70^o^F ± 2 ^o^F; quality of water- acidified; environmental enrichment- nestlet for singly housed and breeding; Food Harland Teklad 2920(regular diet), 2919 (breeder chow), mating cages were maintained as trios (one male and two females)
3. Not applicable

**Sample size**

**10 a. Specify the total number of animals used in each experiment and the number of animals in each experimental group.**

**b. Explain how the number of animals was decided. Provide details of any sample size calculation used.**

**c. Indicate the number of independent replications of each experiment, if relevant.**

Due to the expense of Next Generation Sequencing, two animals were sequenced at each time-point and all miRNAs were normalized to the total miRNA reads to reduce inter-animal variation.

**Allocating animals to experimental groups**

**11 a. Give full details of how animals were allocated to experimental groups, including randomisation or matching if done.**

**b. Describe the order in which the animals in the different experimental groups were treated and assessed.**

RNA was extracted from all pups of two litters for each age. Two samples which passed the RNA quality check were selected for sequencing. All samples from the same age were sequenced simultaneously.

**Experimental outcomes**

**12 Clearly define the primary and secondary experimental outcomes assessed (e.g., cell death, molecular markers, behavioural changes).**

As the studies are primarily descriptive the validity of the RNAs described was assessed through quality control prior to sequencing and comparison to other studies of testicular miRNAs.

**Statistical methods**

**13 a. Provide details of the statistical methods used for each analysis.**

**b. Specify the unit of analysis for each dataset (e.g. single animal, group of animals, single neuron).**

**c. Describe any methods used to assess whether the data met the assumptions of the statistical approach.**

No statistical analysis was used in these studies.

**RESULTS**

**Baseline data
14 For each experimental group, report relevant characteristics and health status of animals (e.g., weight, microbiological status, and drug- or test-naıve) before treatment or testing (this information can often be tabulated).**

All animals analyzed were in good health.

**Numbers analysed**

**15 a. Report the number of animals in each group included in each analysis. Report absolute numbers (e.g. 10/20, not 50%).**

**b. If any animals or data were not included in the analysis, explain why.**

Please refer to #6.

**Outcomes and estimation**

**16 Report the results for each analysis carried out, with a measure of precision (e.g., standard error or confidence interval).**

**Adverse events**

**17 a. Give details of all important adverse events in each experimental group.**

**b. Describe any modifications to the experimental protocols made to reduce adverse events.**

There were no adverse events.

**DISCUSSION**

**Interpretation/scientific implications**

**18 a. Interpret the results, taking into account the study objectives and hypotheses, current theory, and other relevant studies in the literature.**

**b. Comment on the study limitations including any potential sources of bias, any limitations of the animal model, and the imprecision associated with the results.**

**c. Describe any implications of your experimental methods or findings for the replacement, refinement, or reduction (the 3Rs) of the use of animals in research.**

There might be strain-specific differences in the miRNA complement of the mouse testis, which were not assessed in this study. Due to the lack of cell lines which reiterate the male germ cell differentiation program, an *in vitro* study design is not currently feasible.

**Generalisability/translation**

**19 Comment on whether, and how, the findings of this study are likely to translate to other species or systems, including any relevance to human biology.**

This study does not imply that mouse and human testicular miRNAs will be identical. A subset of miRNAs are rodent- or primate-specific which limits the generalizability of the findings of this study in the mouse to modeling human spermatogenesis. However, due to the difficulty of getting sufficient human testicular biopsies, it is unlikely at the current time that a similar analysis in human samples will be performed, although an analysis using non-human primates is feasible. For the majority of miRNAs conserved between mouse and human, their developmental pattern of expression may be similar.

**Funding**

**20 List all funding sources (including grant number) and the role of the funder(s) in the study.**

This research was supported in part by NIH-NICHD 5R01HD057880 (to M.M.M), NIH Epigenomics Roadmap Initiative grant from NIH-NIDA 5U01DA025956 and NIH-NHGRI 5R01HG4009 (to A.M.), and NIH-NICHD 5T32HD007165 (to G.M.B.). We also thank the Cullen Foundation for their generous support of the University of Houston’s Institute for Molecular Design (IMD) Illumina/Solexa Sequencer. The funders had no role in study design, data collection and analysis, decision to publish, or preparation of the manuscript.
